# A 2cM genome-wide scan of European Holstein cattle affected by classical BSE

**DOI:** 10.1186/1471-2156-11-20

**Published:** 2010-03-29

**Authors:** Brenda M Murdoch, Michael L Clawson, William W Laegreid, Paul Stothard, Matthew Settles, Stephanie McKay, Aparna Prasad, Zhiquan Wang, Stephen S Moore, John L Williams

**Affiliations:** 1 Department of Agricultural, Food and Nutritional Science, University of Alberta, Edmonton, Canada; 2 USDA, ARS, US Meat Animal Research Center, Clay Center, Nebraska, USA; 3 Department of Veterinary Pathobiology, University of Illinois, Urbana, Illinois, USA; 4 Department of Animal Science, Washington State University, Pullman, Washington, USA; 5 Divisions of Animal Sciences, University of Missouri, Columbia, Missouri, USA; 6 Parco Tecnologico Padano, Via Einstein, Polo Universitario, Lodi, 26900 Italy

## Abstract

**Background:**

Classical bovine spongiform encephalopathy (BSE) is an acquired prion disease that is invariably fatal in cattle and has been implicated as a significant human health risk. Polymorphisms that alter the prion protein of sheep or humans have been associated with variations in transmissible spongiform encephalopathy susceptibility or resistance. In contrast, there is no strong evidence that non-synonymous mutations in the bovine prion gene (*PRNP*) are associated with classical BSE disease susceptibility. However, two bovine *PRNP *insertion/deletion polymorphisms, one within the promoter region and the other in intron 1, have been associated with susceptibility to classical BSE. These associations do not explain the full extent of BSE susceptibility, and loci outside of *PRNP *appear to be associated with disease incidence in some cattle populations. To test for associations with BSE susceptibility, we conducted a genome wide scan using a panel of 3,072 single nucleotide polymorphism (SNP) markers on 814 animals representing cases and control Holstein cattle from the United Kingdom BSE epidemic.

**Results:**

Two sets of BSE affected Holstein cattle were analyzed in this study, one set with known family relationships and the second set of paired cases with controls. The family set comprises half-sibling progeny from six sires. The progeny from four of these sires had previously been scanned with microsatellite markers. The results obtained from the current analysis of the family set yielded both some supporting and new results compared with those obtained in the earlier study. The results revealed 27 SNPs representing 18 chromosomes associated with incidence of BSE disease. These results confirm a region previously reported on chromosome 20, and identify additional regions on chromosomes 2, 14, 16, 21 and 28. This study did not identify a significant association near the *PRNP *in the family sample set. The only association found in the *PRNP *region was in the case-control sample set and this was not significant after multiple test correction. The genome scan of the case-control animals did not identify any associations that passed a stringent genome-wide significance threshold.

**Conclusions:**

Several regions of the genome are statistically associated with the incidence of classical BSE in European Holstein cattle. Further investigation of loci on chromosomes 2, 14, 16, 20, 21 and 28 will be required to uncover any biological significance underlying these marker associations.

## Background

Transmissible spongiform encephalopathies (TSEs) are fatal neurodegenerative diseases that have been identified in a number of mammalian species (humans, cattle, sheep, mice, etc.) [1]. One of the common characteristics of these diseases is the accumulation of abnormally folded prion protein within the central nervous system. The prion protein is a glycosyl-phosphatidylinositol (GPI) anchored protein that has a native form (PrP^c^) for which the secondary structure consists mainly of alpha-helices. The disease-associated or misfolded form, PrP^Res^, has a substantial increase in the beta-pleated sheets content and reduction of the alpha-helices in comparison to the native form [2]. This altered confirmation is associated with an increased resistance to digestion with proteinase K [3]. Furthermore, the presence of PrP^Res ^behaves like a seed that promotes the conversion of further native PrP^c ^to PrP^Res ^via a mechanism that is to date not completely understood [4].

Specific *PRNP *alleles of non-synonymous polymorphisms in humans and sheep are associated with acquired TSE susceptibility [5-8]. This is not the case in cattle, although the deletion alleles of a 23 base pair insertion/deletion (InDel) polymorphism in the bovine *PRNP *promoter region and of a 12 base pair InDel within intron 1 have been associated with incidence of classical bovine spongiform encephalopathy (BSE) [9-11]. Both of these polymorphisms are contained in a block of high linkage disequilibrium (LD) within *PRNP *that appears conserved in many cattle populations, and is entirely outside of the coding region [12]. Thus, the full extent of *PRNP *association with classical BSE is currently not known. Previous genetic studies of BSE cattle have identified putative loci other than *PRNP *(located on chromosome 13 at 47.2 Mb) that are associated with incidence of disease [13,14]. These studies carried out low density whole-genome scans with microsatellite markers approximately every 20 cM in female European Holstein cattle which contracted BSE and unaffected half-sib controls.

The objective of this study was to test loci throughout the bovine genome for an association with classical BSE using markers at 2 cM resolution. This resolution is approximately a ten-fold improvement over previous genome scans of BSE samples. Two animal sample sets were used, allowing for two analysis approaches: a case-control association study and family based sib-transmission disequilibrium test (sib-TDT) study [13,14]. In both sample sets the cattle used were female European Holstein cattle which contracted BSE in the late 1980's and early 1990's (family analysis) and in the mid-1990's (case-control analysis) and were identified on commercial farms where the most likely source of disease was through consumption of contaminated feed during the United Kingdom's BSE epidemic. Although the United Kingdom imposed a ruminant feed ban of meat and bone meal in June 1988, it wasn't until August 1996 that a total ban on bovine meat and bone meal was implemented [15].

## Results

The panel of SNP markers used in this study consisted of 3,072 SNPs dispersed across the genome at an approximate average interval of 2 cM [16]. Of these, an average 2,853 passed quality control measures and had an average genotyping success rate of 0.99. The samples used in this study came from two sets of animals with different relatedness that allowed for the use of several statistical analyses to test the association between SNP genotypes with classical BSE incidence. By using both offspring from six sires (sib-TDT) and (case-control) Holstein animals, this study examined within-breed and within-family SNP association with disease incidence.

### Family based association testing for BSE incidence

The related sample set (N = 481) was comprised of six paternal half-sib sire families of which 4 were scanned previously with microsatellites [13,14]. Samples were not available from either the sires or the dams; therefore, the sib-TDT analysis method [17] was used. Additionally, PLINK software [18] was used to establish an empirical p value and determine significance. The results for this sib-TDT analysis (412 animals across 2,827 SNPs after quality control) identified 46 SNPs that passed the Bonferroni correction with a p < 0.05 and 27 SNPs that passed the Bonferroni correction with a p < 0.01 (Table 1). In addition, a 10,000,000 permutation test was performed on this data using the PLINK "max T" to establish genome wide empirical p values. The genome wide 10,000,000 permutation identified 31 SNPs with an empirical permutation of p < 0.01. This group of SNPs included the 27 SNPs which passed the Bonferroni at p < 0.01 plus four additional SNPs. Many of these SNPs are located in very close proximity [16] to one another and are potentially in LD in the population.

**Table 1:** The results of sib-TDT model analysis using the large family sample set.

		Location		unadjusted	permuted	
SNP ID	CHR	bp	Gene	p value	p value	Bonferroni
AAFC02065030	2	37,055,124		1.93E-08	3.61E-05	5.45E-05
rs29020694	4	90,418,076		1.87E-06	4.05E-03	5.29E-03
AAFC02132123_1	4	117,126,683	LOC100138299	9.38E-07	1.98E-03	2.65E-03
AAFC02132123_2	4	117,126,810	LOC100138299	3.38E-06	7.33E-03	9.55E-03
AAFC02012009	5	30,554,866	LOC507184	3.53E-06	7.69E-03	9.98E-03
rs29003193	5	66,501,993		3.12E-08	5.79E-05	8.82E-05
rs29012226	5	67,701,052	ANKS1B	6.63E-07	1.38E-03	1.87E-03
SCAFFOLD106936	6	98,767,642		6.35E-07	1.32E-03	1.80E-03
rs29016161	7	24,604,791		2.45E-06	5.31E-03	6.93E-03
rs29017305	9	62,946,258	BACH2	2.30E-06	4.97E-03	6.49E-03
rs29013631	10	38,725,566		2.25E-06	4.87E-03	6.36E-03
rs29022366	12	80,076,729	LOC786668	2.74E-06	5.95E-03	7.75E-03
rs29010388	14	4,145,186		4.20E-07	8.56E-04	1.19E-03
AAFC02138417	15	70,632,844		3.31E-06	7.19E-03	9.36E-03
rs29010371	16	63,558,950	FAM129A	1.06E-06	2.26E-03	3.00E-03
rs29009572	17^#^	37,575,810		1.29E-06	2.76E-03	3.65E-03
rs29021871	17	44,213,230		1.06E-06	2.25E-03	2.99E-03
CART	20^#^	4,922,252	CART	3.57E-08	6.75E-05	1.01E-04
rs29018531	20	38,814,738		1.32E-06	2.82E-03	3.72E-03
AAFC02028192	21	5,702,403	MEF2A	1.24E-07	2.49E-04	3.49E-04
rs29022862	21	12,595,937		1.85E-07	3.73E-04	5.22E-04
rs29009825	21	24,827,395		1.90E-06	4.11E-03	5.38E-03
rs29012664	22	42,415,179	FHIT	5.30E-07	1.10E-03	1.50E-03
NW_930303_1	24^#^	29,286,364		1.02E-07	2.05E-04	2.90E-04
NW_930303_2	24^#^	29,286,314		3.06E-07	6.17E-04	8.64E-04
SCAFFOLD176855	28	26,871,628	SLC29A3	1.03E-06	2.18E-03	2.90E-03
SCAFFOLD68962	X	7,641,750		3.15E-06	6.86 E-03	8.91E-03

Locations were determined by blast to bovine sequence version 4.0. All other locations denoted by ^# ^were either determined from bovine sequence version 2.0, 4.1 or the Maryland sequence assembly. Permuted p values reported here are from a 10,000,000 genome-wide SNP permutation.

### Case-control association testing for BSE incidence

The case-control samples were comprised of 149 BSE case and 184 control animals. The control samples include a least one animal collected from the same farm as each of the BSE cases as well as the controls for, and 15 BSE negative animals. The genotyping data on these animals was analyzed for an association with disease status using the case-control allelic test within the PLINK software [18]. This analysis (320 animal across 2,872 SNPs after quality control) revealed 20 SNPs with a p < 0.01, 14 SNPs with a p < 0.005 and 6 SNPs with a p < 0.001. In order to determine the number of these SNPs that may have occurred by chance, an empirical p-value for each single SNP and across all SNPs (genome wide) was calculated using the max (T) permutation procedure with 10,000 permutations. Following correction for the false discovery rate no significant associations at p ≤ 0.05 genome-wide significance were identified in this data set. This was consistent with the use of Bonferroni multi-test correction on this data set, where again none of the SNPs achieved significance of p ≤ 0.05. To assess significance a threshold was set at p ≤ 1.7 × 10^-5 ^(Bonferroni calculation). Twenty SNPs were identified with p < 0.01 (Table 2), where four SNPs had a p ≤ 5 × 10^-4 ^and one SNP had a p ≤ 1 × 10^-4^. Thus a single SNP had a suggestive association with BSE incidence on chromosome 14 (p = 7.25 × 10^-5^). SNPs which did not quite reach this threshold but had a p ≤ 5 × 10^-4 ^were found on chromosomes 4, 10, 14, and 15. The number of loci on each chromosome, the identity of these specific SNPs and their corresponding p-values are reported in Table 2.

**Table 2:** PLINK case-control association results of the case-control sample set.

		Location		unadjusted
SNP ID	CHR	bp	Gene	p value
rs29013431	2	15,312,400		5.99E-03
rs29012194	2	26,095,592		6.28E-03
rs29016537	2	29,654,789		1.30E-03
rs29020907	4	59,252,723	IMP2	4.97E-04
rs29024570	10	4,359,586		4.30E-04
AAFC02107025	10	21,153,203	DHRS1	4.73E-03
rs29024728	13	28,618,368		3.34E-03
PRNP08	13	47,214,453	PRNP	4.38E-03
rs29021171	14	10,106,755		3.84E-03
SCAFFOLD51887	14	43,984,153		7.25E-05*
rs29014819	15	35,713,980		7.17E-04
rs29014820	15	35,714,095		6.05E-04
rs29014821	15	35,714,113		4.66E-04
AAFC02014662	16	65,943,749		7.95E-03
AAFC02012500_1	21	11,820,092	MCTP2	1.82E-03
AAFC02012500_2	21	11,820,201	MCTP2	3.93E-03
rs29026011	21	13,254,954	LOC618464	3.00E-03
rs29019629	21	33,209,686	CSPG4	2.01E-03
rs29011202	24	18,493,192		9.08E-03
AJ496776	28	31,495,721		5.74E-03

All locations determined by blast to bovine sequence version 4.0. Threshold of significance is p ≤ 1.7 × 10^-5 ^and suggestive significance p ≤ 10^-4 ^is denoted as *.

This data set was also subjected to a best fit model test where the standard allelic, trend, dominance, recessive and genotypic association tests were performed, and the test with the lowest p value was reported. All of the SNPs identified above were also identified in the best fit model as either allelic or trend, however additional SNPs with recessive, dominant and genotype associations were also identified. In the best fit model there were a total of fourteen SNPs with a recessive mode of action, seven dominant SNPs and eight genotypic SNPs with a p < 0.01 (see Additional file 1). Using the same thresholds as described above, (i.e. significant with a p ≤ 10^-5 ^and suggestive with a p ≤ 10^-4^), one SNP on chromosome 14 had a suggestive association with BSE incidence.

## Discussion

### Classical BSE as a phenotype

Clinical presentation of BSE disease is a difficult phenotype to test for genetic associations. Animals that have developed BSE are clearly susceptible, however, those which are clinically healthy are difficult to assess. In the present study, clinically healthy animals were used as controls, however, these animals may have been incubating disease or may not have ingested enough infectious agent to become symptomatic, or alternatively, they may in fact have been resistant to disease. Therefore, the analysis was performed with the realization that phenotypic noise in the controls will have reduced the power to detect associations. Another consideration is that classical BSE is a complex trait which may be more consistent with interactive and possibly subtle effects of multiple contributing loci. Therefore, multiple testing corrections applied to results such as these may be overly prone to type II errors (i.e. discarding real associations). Consequently, it is important to examine the results for supporting evidence of associations between disease and genetic loci, as discussed below.

### PRNP gene

*PRNP *variation was not exhaustively tested for an association with classical BSE as the focus of this study was primarily genome wide. Over 380 polymorphisms are known to reside throughout the coding and non-coding regions of *PRNP *[12]. Of these, 13 *PRNP *haplotype tagging SNPs (htSNPs) were used in the scans of which 8 were informative for both data sets. The htSNPs used in this study capture a large portion of *PRNP *haplotype variation observed in a diverse assemblage of U.S cattle, spanning the promoter region into the last exon, however, they do not capture all of it. Additionally, the two InDels previously identified [9-11] as having allele associations with classical BSE were not genotyped in this study and thus no information is available for this sample set and the InDels *PRNP *haplotypes. One SNP (PRNP08) had a *p *value of 4.38 × 10^-3 ^for an association with classical BSE but did not pass multiple test corrections. Consequently, no significant associations of *PRNP *variation (the 8 informative htSNPs) with classical BSE were identified in this single marker analysis. Given that the single marker analysis of the informative htSNPs did not capture all of the htSNPs in the *PRNP *region or the two InDels it should not be considered an exhaustive analysis of the *PRNP *gene region.

### Family-based analysis

Family-based analyses, although in general being less powerful than case-control studies [19], offer robustness to non-random mating. The transmission/disequilibrium test developed in 1993 by Spielman [17] is intended to test for linkage between complex diseases and genetic markers. The sib-TDT approach used here does not reconstruct parental genotypes in their absence, but uses marker data from unaffected half siblings instead [17]. The DFAM analysis model fits the structure of the related half-sibling sample set and has been utilized in other species as well [20]. The study presented here used a much larger number of markers than the previous studies and used an analysis approach that is robust to population stratification [21]. The advantage this analysis has over the case-control approach is that with 302 affected animals it has twice the number of affected individuals, and thus, a higher study power and likelihood of detecting markers associated with disease loci.

Many of the samples included in the family sample set used in this study were also used by Hernández-Sánchez *et al*. [13] and Zhang *et al*. [14], while the case-control sample set was analyzed here for the first time. Hernández-Sánchez *et al*. [13] also used a TDT approach, however, their analysis method requires heterozygous parents to allow the parent of origin of alleles to be unequivocally determined and as a result many animals had to be disregarded in their analysis. In addition, progeny with the same genotype as the predicted genotype of the sires or progeny that were themselves homozygous were excluded. As a result, although the TDT method used by Hernández- Sánchez *et al*. [13] has the potential to be powerful, the use of this approach with microsatellite marker based data was limited by the number of genotypes which could be used and ranged from 92 (in the case of marker BMS1658) to 210 (in the case of INRA36). The analysis method used to localize QTLs by Zhang *et al*. [14] was a regression approach which does not require the parents to be heterozygous and hence all individuals could be included in the analysis. However, the QTL approach is not robust to population stratification. Moreover, the total number of samples (360) used in the Zhang *et al*. [14] study was smaller than that of Hernández-Sánchez *et al*. [13] (530) as well as this study (412). The two previous analyses of overlapping family samples yielded different results: the TDT analysis of Hernández-Sánchez *et al*. [13] found evidence for associations with BSE incidence on chromosomes 5, 10 and 20, whereas the QTL analysis by Zhang *et al*. [14] identified BSE associated QTL chromosomes 1, 6, 13, 17, 19 and X/Y_ps_. SNPs in the regions matching regions found in previous studies are detailed in Table 3. With regards to the family data this study offers a similar power to that of the TDT analysis by Hernández-Sánchez *et al*. [13], however uses half-sib controls as opposed to inferring the sires genotype. In addition, due to necessary genotypic restrictions (only heterozygous genotypes can be used) of the TDT method, the approach used here allowed for a greater number of animals to be included in the analysis. Although the total number of animals used in this analysis includes two smaller families (6 families versus 4 families in the previous studies [13,14]) this analysis method does not infer sire genotypes and therefore the inclusion of the two smaller families do not reduce the overall power of this analysis.

**Table 3:** Comparison of SNPs observed in this study with locations indentified in other studies.

		Analysis		Location	Previously	Peak
CHR	SNP ID	Method	p-value	Mb	Observed	Location Mb
1	rs29009859	sib-TDT	2.0 × 10^-5^	89.7	[14]	106.5
5	AAFC02012009	sib-TDT	**9.9 × 10^-3^	30.6	No	
5	E25B16-36408-3	sib-TDT	**8.8 × 10^-5^	66.5	No	
5	rs29012226	sib-TDT	**1.9 × 10^-3^	67.7	No	
5	rs29024670	sib-TDT	3.0 × 10^-5^	112.8	[13]	107.0
6	SCAFFOLD106936_12205	sib-TDT	**1.8 × 10^-3^	98.8	No	
10	rs29024570	Case-control	*4.3 × 10^-4^	4.4	No	
10	rs29013631	sib-TDT	**6.5 × 10^-3^	38.7	No	
10	rs29015623	sib-TDT	9.0 × 10^-5^	48.0	[13]	40.0
17	rs29021871	sib-TDT	**3.0 × 10^-3^	44.2	No	
19	rs29027102	sib-TDT	2.0 × 10^-5^	62.5	[14]	53.5
20	rs29018531	sib-TDT	**3.7 × 10^-3^	38.8	[13]	46.0
20	CART-SNP	sib-TDT	**1.0 × 10^-4^	4.9^#^	No	
X	SCAFFOLD68962_9331	sib-TDT	**1.0 × 10^-5^	7.6	No	
X	SCAFFOLD285727_12117	sib-TDT	1.0 × 10^-5^	85.5	[14]	112.5

Sib-TDT p values are reported as unadjusted unless denoted by ** which indicates Bonferroni corrected values that pass 10,000,000 permutations. All case control p-values are reported are as unadjusted and suggested significance is denoted as *. Only SNPs with appreciable or significant p-values located within the confidence intervals of ref# [14] or within 10 cM of ref# [13] are reported here. The location determined by older bovine sequence version 2.0 is denoted by ^#^. All other locations were determined by blast to bovine sequence version 4.0 and are reported in mega bases. The previous studies reported location in cM however for the sake of uniformity the location is reported here, based on marker positions, in Mb.

The sib-TDT analysis in this study identified two significant SNPs on BTA 20 associated with BSE incidence, rs29018531 at 38.8 Mb and a SNP within the cocaine and amphetamine responsive transcript peptide gene, (*CART*). *CART *is not currently on the Btau4.0 bovine sequence assembly but was previously mapped to chromosome 20 at 38.5cR [15]. In addition, the location of *CART *was reported on Btau2.0 as 4.92 Mb as well as the Maryland map as 9.78 Mb. Therefore it is unclear from this study if the observed associations identified here are attributable to one locus or two separate loci. The study of Hernández-Sánchez *et al*. [13] observed an association with marker INRA36 (at 37.9 Mb) on BTA 20 with BSE incidence. This study also identified significant markers on chromosomes 5, 6, 10, 17 and X associated with BSE. The study by Hernández-Sánchez *et al*. [13] also reports associations on BTA 5 and 10 but did not report confidence intervals. The marker identified on BTA 6 in this study (Table 3) is in the same chromosomal region as the marker described by Zhang *et al*. [14] and is within the confidence interval. The precise location of these significant markers can be found in Table 3. Moreover, the significant marker on BTA 6, Scaffold106936 at 98.7 Mb corresponds with the QTL region, 51-72 cM on mouse chromosome 5, previously associated with susceptibility to TSE in mice [22]. Additionally, the homologous region to that identified on BTA 6 was also identified as a QTL modulating scrapie incubation period in sheep [23]. Interestingly, the QTL region on mouse chromosome 5 described by Moreno *et al*. [22] also corresponds to the location of a significant SNP identified on BTA 17, 44.2 Mb. Thus these chromosomal regions identified in the present study are also supported by studies in cattle and other species. Comparative locations were determined by using the National Center for Biotechnology information map viewer of the mouse QTL regions (build 37.1), then the human and Btrna were selected and bovine locations were determined.

In QTL studies in mice Monero *et al*., 2008 [22] and Llyod *et al*., 2002 [24] used a panel of 72 microsatellites to examine 282 F2 and 124 F2 mice respectively. However these mice were inoculated intracerebrally as compared to our study where infectious material was orally ingested through contaminated feed. Comparatively these studies have fewer animals and although they use microsatellite markers the information content is limited due to the fact that both studies only use 2 inline bred strains. In addition, it should be noted that single marker association analysis, as reported in this manuscript differs from QTL analysis. These differences make direct comparison rather difficult. Given that neither this study nor the previous studies, even in other species, can really be considered comprehensive genome scans they suffer from the same shortfalls. In this study there remains the distinct possibility of a type 2 error which fails to identify a genome location that is associated with disease.

### Case-control analysis

In selecting the analysis approach, it is important to match the appropriate model to the data structure to maximize the power. This study used a case-control approach to analyze the paired control with BSE animals. This approach is powerful in its ability to detect loci linked with disease: however, it has been criticized by geneticists for its lack of robustness for population stratification arising from non-random mating or unknown relationships between individuals [19]. The data presented here was examined for stratification and none was observed (see Additional file 2). However, it is likely that some family relationships exist between some of the cases and controls but the extent and the effect was unable to be determined. Despite the power of the case-control approach, this study was limited by the relatively small number of animals used (149 cases and 184 controls) and no significant results were observed. An increase in the number of cases and controls included in this sample set would have a dramatic effect on the power to detect loci associated with disease [19], as would a higher density scan conducted with an increased number of genome-wide markers.

### Shared regions identified in the sib-TDT family and implicated in the case-control analysis

Many of the SNPs included in the panel are in close proximity and are in LD in Holstein [16]. Therefore, it may be more appropriate to consider the results in terms of chromosomal regions instead of individual markers. Linkage disequilibrium will result in the alleles of several closely spaced SNPs being associated with disease status because they all fall on the same haplotype. Thus, it would be expected that several SNPs in LD with a locus involved in disease would show significant associations. Examples of this can be observed with the loci on BTA 15 in Table 2 as well as BTA 4 in Table 1. In addition, if the same regions give significant or a nearly significant association across the different sample sets, this would also increase confidence that the association is real. Chromosome 2 is a good example: the most significant marker in this study, AAFC02065030, with a Bonferroni corrected p = 5.5 × 10^-5 ^in the family based analysis is at 37.1 Mb on this chromosome. In the case-control samples set, three markers were identified on Chromosome 2, one at 15.3 Mb, another at 26.1 Mb, and the last (p = 1.0 × 10^-3^) at 29.7 Mb. From the different analyses methods and across the two sample sets four markers were identified in the chromosomal region from 15.3 Mb-37.1 Mb on BTA2, with the region of 29-37 Mb being significant. This data supports an association between chromosome 2 and BSE disease, however from the data it cannot be determined if this effect is from one locus or several loci spread across the region.

Another chromosomal region which harbours significant markers that were identified in the family-based analyses and was observed but failed to reach significance in the case-control analysis is on chromosome 21. Four SNPs were observed on BTA 21 from the analysis of case-control samples, two SNPs which are located at 11.8 Mb (p = 4.4 × 10^-3 ^and p = 8.3 × 10^-3^) in the *MCTP2 *gene, another at 13.3 Mb (p = 2.5 × 10^-3^) in LOC618464, and an additional SNP at 33.2 Mb (p = 2.0 × 10^-3^) in the gene *CSPG4*. The family based sib-TDT analysis identified three significant SNPs, at 5.7 Mb in the *MEF2 *gene (p = 3.5 × 10^-4^), 12.6 Mb (p = 5.2 × 10^-4^), and 24.83 Mb (p = 5.4 × 10^-3^). When considering both sample sets there appears to be an interval from 11.8 Mb-13.3 Mb with the outer range of approximately 5.7-33.2 Mb for a locus with an effect on BSE. BTA 21 shares conservation of synteny with sheep OAR 16 where QTLs for scrapie susceptibility and scrapie incubation period have been reported [25].

Regions containing loci significantly associated with disease status identified on chromosomes 14, 16 and 28 in the sib-TDT analysis of the family-based data were also observed to be of interest, but failed to reach significance, in the case-control study. On chromosome 14 the marker, rs29021171, at 10.1 Mb had a p = 2.3 × 10^-3 ^in the analysis of case-control animals and is in relative close proximity to, rs29010388, at 4.2 Mb which was identified as significant in the sib-TDT analysis (p = 1.2 × 10^-3^). On chromosome 16 marker AAFC02014662 at 65.9 Mb identified in the case-control (p = 7.95 × 10^-3^) is in close proximity to rs29010371 at 63.5 Mb, which is in the gene *FAM129A*, that was identified as significant (p = 3.0 × 10^-3^) in the sib-TDT analysis. Finally on chromosome 28 marker AJ496776, at 31.5 Mb identified in the case-control sample analysis (p = 9.9 × 10^-3^) is in close proximity to the marker SCAFFOLD176855 (within *SLC29A3 *gene) at 26.9 Mb identified as significant (p = 2.9 × 10^-3^) in the sib-TDT analysis. None of these chromosomal regions have been previously reported as being associated with BSE.

### Candidate genes identified in the case-control and/or sib-TDT family analysis

The SNPs identified from the family samples on chromosomes 4, 5, 9, 12, 16, 20, 21, 22 and 28 are all found within genes; however, the polymorphisms on chromosomes 4 and 12 are in hypothetical genes. The most notable is the polymorphism on chromosome 5, rs29012226, in the ankyrin repeat and sterile alpha motif domain containing 1B gene (*ANKS1B*). This gene is also known as amyloid beta protein precursor (*APP*) intracellular domain associated protein 1 (AIDA-1), and is associated with *APP *binding [26]. It is well known that APP generates beta amyloid and plays a key role in Alzheimers disease [27-30]. Further cellular prion protein and AIDA-1 has been implicated as a receptor for amyloid-β oligomers [31,26], making *ANKS1B *a good candidate gene for further study.

Another candidate gene, which is in close proximity to three SNPs on BTA 2 at ~29.3 Mb, with alleles that associated with BSE incidence is *B3GALT1*. Beta-1,3-galactosyltransferase (*B3GALT1*), is a transferase polypeptide gene involved in the biosynthesis of GPI anchors. The involvement of the GPI anchor, with lipid raft and TSE disease has been investigated [32] and it is thought that the GPI anchor may affect the conformation, or the association of the prion protein with specific membrane domains [33]. An additional potential candidate gene is *CART*, cocaine and amphetamine regulate transcript. This neuropeptide plays a role in a variety of physiological processes, some of which include: promotion of hippocampal neurons by upregulating brain-derived neurotrophic factors [34] and synaptogenesis [35]. In addition, the expression of *CART *has shown to be down regulated in mouse prion disease [36].

The SNP located on BTA 14 (43.9 Mb), associated with BSE incidence in the case-control analysis, is in close proximity to the gene exostoses (multiple) 1, *EXT1*. McCormick et al., [37] showed that *EXT1 *is an endoplasmic reticulum (ER)-resident type II transmembrane glycoprotein whose expression in cells results in the alteration of the synthesis and display of cell surface heparan sulfate glycosaminoglycans (GAGs). The N terminus of PrP contains a GAG-binding motif and it is thought that PrP binding of GAG is important in prion disease [37-39]. Additionally, this region contains another candidate gene *STMN2*, which has been identified in a whole genome association study for genetic risk factors for variant Creutzfeldt-Jakob in humans [40]. Specifically, Mead and others [40] found an association with acquired prion diseases, including vCJD (p = 5.6 × 10(-5)), kuru incubation time (p = 0.017), and resistance to kuru (p = 2.5 × 10(-4)), in a region upstream of *STMN2 *(the gene that encodes *SCG10*). Superior cervical ganglion 10, *SCG10*, is a neuronal growth associated protein and may play a role in neuronal differentiation in modulating membrane interaction with the cytoskeleton during neurite outgrowth. *STMN2 *is at 39.9 Mb on chromosome 14 in cattle, which is in close proximity to *EXT1*, making both *STMN2 *and *EXT1 *are functional and positional gene candidates.

## Conclusions

The large number of SNP markers and the two sets of animals used in this study make it the most comprehensive study to date to test genetic loci for an association with classical BSE in European Holstein cattle. The genome-wide scan of half sib families identified an association between the genetic loci on 18 chromosomes with BSE incidence in European Holstein cattle, including a region on BTA 20 associated with BSE incidence that has been reported in previous studies. The identification of markers at or near statistical significance within the same chromosomal regions in both sets of samples provides independent evidence for the association of those regions and the presence of one or more genes within the regions influencing the incidence of BSE in cattle. However, these results need to be confirmed in additional cattle populations or other species. The data in this study can be made available upon request.

It is worth noting that this study identified a large number of associations with classical BSE disease incidence throughout the bovine genome versus one single major locus with a large effect in the bovine genome. This would make it difficult to select cattle that are genetically resistant to classical BSE, however the results give some insight into gene pathways important during disease progression.

## Methods

### Animal information

This study used two sets of samples from cases and controls, but with different structures. The first sample set consisted of female European Holstein collected in the mid 90's and included 149 BSE case and 184 control animals. The control animals were contemporaries of the BSE cases and collected from the same farms. In addition 15 BSE negative, determined by post-mortem histology, and paired control animals were included in the control set. The second sample set was family based and consisted of 302 BSE affected and 179 unaffected half-sib Holsteins from six sire families. All the BSE affected and unaffected cattle within one family were paternal half sibs from the designated sire but with different dams. No DNA samples were available from any of the sires.

In both sample sets cattle designated as BSE positive were first examined by qualified veterinarians. BSE status was subsequently confirmed post-mortem by histology (by the Veterinary Laboratories Agency, New Haw, Surrey, UK). None of the control animals exhibited any clinical symptoms of disease and were presumed to be free of disease. All of the control animals were age and sex matched from the same calving season and from the same farm as the BSE cases. As such, the control animals are assumed to have been exposed to the same environment.

### DNA isolation and genotyping

Genomic DNA from the case-control was isolated from blood using a high salt phenol/chloroform extraction method as described by Sherman *et al*. [41]. Genomic DNA from the family animal set was isolated from blood samples by phenol and chloroform extraction, as described by Hernández-Sánchez *et al*. [13].

The genotyping panel was comprised of two oligonucleotide pool assays (OPAs) as described by McKay and others in 2007 [16]. Briefly, 5,500 SNP were mapped on the Roslin-Cambridge 3,000 rad bovine-hamster whole genome radiation hybrid panel (WGRH3000) [42] and the minor allele frequency (MAF) was determined on a variety of breeds, including Holstein. Of the original SNPs, 3,072 were selected to give the greatest genome coverage and MAF >0.05 for the genome scan. An Illumina GoldenGate assay [43] was performed using the two custom OPAs and genotypes determined using an Illumina BeadScan (Illumina Inc., San Diego, CA) and the Illumina BeadStudio software. Sequences containing SNPs (see additional file 3) were blasted on the bovine assembly (4.0) to determine the SNP locations [16] and, the location for all except 111 SNPs were determined.

### Data quality control

Quality control analyses were carried out by removing confounding effects prior to the data analysis. For the case-control sample set from the original 3,072 SNPs in the assay, 122 SNPs were excluded based on assay failure, as observed by poor clustering of alleles using the BeadStudio software. The remaining 2,950 SNPs were submitted to the PLINK program [18] where seven duplicated SNPs were removed and 12 SNPs were removed because more than 10% of the samples failed to genotype at that locus (GENO >0.1). Additionally, 13 samples were removed due to low genotyping rate (MIND>0.1). A further 61 SNPs were removed due to a MAF <0.01. The MAFs for each of the SNPs in this data set is provided in additional file 3 and the graphical representation of the MAF for each chromosome is shown in additional file 4. The remaining individuals that were included in the analyses had a mean genotyping rate of 0.995. Population stratification was tested for in this data set and none was observed (data in additional file 2).

For the family sample set, transmission disequilibrium analysis was performed using the sib-TDT application in the PLINK program. Following the removal of SNPs that did not cluster well, 2,904 SNPs were used in the analysis. Of these loci, a further 22 SNPs were removed due to missing data (GENO>0.1), seven duplicated SNPs, and a further 48 SNPs were removed due to low allele frequency (MAF<0.01). The MAFs for each of the SNPs in this data set is provided in additional file 3 and the graphical representation of the MAF for each chromosome is shown as in additional file 4. Of the individuals examined, 5 samples failed and 64 were removed because of low genotyping frequency (MIND>0.1), for the 412 remaining individuals (see Table 4) a genotyping success rate of 0.98 was obtained.

**Table 4:** The sire identities and the number of half sib offspring analyzed in this study verses the number of animal used two previous studies.

Sire	Number of BSE			Number of control		
	**This study**	[13]	[14]	**This study**	[13]	[14]

Barold Rock Seal	33	53	43	11	28	22
Brynhyfryd Cascade	68	93	72	28	56	18
Leighton Workboy	71	88	70	40	44	22
Maybar Juniper	90	124	83	51	44	30
Bowerchalk Polacca	4	0	0	6	0	0
Deri cascade	3	0	0	7	0	0

Total	269	358	268	143	172	92

### Statistical analysis

The PLINK software v1.04 was used to perform the majority of the statistical analysis [18]. The data from the case-control sample set were analyzed using the basic case-control association (χ^2^) test. Whereas the family sample set was analyzed with the DFAM program [18], which is an adjusted family TDT analysis, as described by Spielman [17]. To correct for multiple tests Bonferroni single-step adjusted p-values (BONF) procedures were applied. A permutation test was also used in this study, which was max(T)" permutation with 10,000 and 10,000,000 permutations, a procedure that permutes both a point-wise SNP significance and a genome-wide significance.

## Abbreviations

BSE: bovine spongiform encephalopathy; cM: centimorgans; cR: centirad; ER: endoplasmic reticulum; GAG: glycosaminoglycans; GPI: glycosyl-phosphatidylinositol; htSNP: haplotype tagging single nucleotide polymorphism; InDel: insertion/deletion polymorphism; LD: Linkage disequilibrium; MAF: minor allele frequency; OPA: oligonucleotide pool assay; PrP^c^: native prion protein; PrP^Res^: misfolded prion protein; QTL: quantitative trait loci; Sib-TDT: sibling transmission disequilibrium test; SNP: single nucleotide polymorphism; TSE: transmissible spongiform encephalopathy.

## Authors' contributions

BMM, SSM carried out the DNA isolation and genotyping and JLW designed strategies for sampling infected and control animals and provided the samples. BMM, MS, PS, AP and ZW participated in the bioinformatics and statistical analysis. BMM, SSM, JLW, MLC, and WWL participated in the study design and helped with the draft of the manuscript. All authors read and approved the final manuscript.

## Supplementary Material

Additional file 1 The best fit model analysis results (Genotypic, Recessive, and Dominant) of the case-control sample set. Click here for file

Additional file 2 Multi-dimensional Scaling (MDS) of the multiple family sample set and unrelated sample set. Click here for file

Additional file 3 Sequence information for the SNPs and the minor allele frequencies for the family and case-control sample sets in order of position. Click here for file

Additional file 4 Graphical plots of the MAF versus position for each chromosome for the multiple family sample set and unrelated sample set. Click here for file
