# Supplementary material for: A 2cM genome-wide scan of European Holstein cattle affected by classical BSE
Source: BMC Genet. 2010 Mar 29;11:20. doi: 10.1186/1471-2156-11-20 (PMC2853485; doi:10.1186/1471-2156-11-20)
Supplement: Additional file 1 — The best fit model analysis results (Genotypic, Recessive, and Dominant) of the case-control sample set. [file 1471-2156-11-20-S1.PDF]

Additional Table 1 – The best fit model analysis results (Genotypic, Recessive, and Dominant) of the case-control sample set.

Locations were determined by blast to bovine sequence version 4.0. The location denoted by # was determined by blast to the Maryland sequence assembly. Threshold of suggestive significance $p \leq 10^{-4}$ is denoted as *.

CHR	SNP ID	Location bp	TEST	AFF	UNAFF	CHISQ	DF	p-value
1	rs29012747	35,152,734	REC	16/125	40/137	6.85	1	8.90E-03
1	rs29026512	59,101,518	DOM	113/29	117/60	7.11	1	7.70E-03
3	rs29016848	90,945,586	DOM	80/63	71/106	7.96	1	4.80E-03
4	rs29010270	104,945,421	REC	14/129	5/172	6.87	1	8.80E-03
4	rs29009791	107,076,639	REC	6/137	26/151	9.68	1	1.90E-03
5	rs29002468	12,350,330	REC	20/122	9/167	7.63	1	5.70E-03
5	AF017143	71,118,012	DOM	111/31	114/62	6.82	1	9.10E-03
5	rs29024670	112,776,759	REC	17/125	5/172	10.27	1	1.49E-03
8	rs29012436	70,299,523	DOM	112/31	113/64	7.95	1	4.80E-03
8	rs29027876	70,303,317	DOM	111/32	113/64	7.15	1	7.50E-03
10	rs29018034	61,645,811	GENO	28/56/59	18/96/63	9.32	2	9.40E-03
11	rs29009663	16,684,267	DOM	84/59	131/45	8.84	1	2.90E-03
13	rs29019327	3,111,153	REC	18/125	45/132	8.24	1	4.10E-03
14	rs29012827	10,347,750	GENO	46/32/61	41/78/52	17.12	2	1.90E-04*
14	rs29021189	13,465,428	REC	42/100	29/148	7.93	1	4.90E-03
15	rs29012082	23,647,376	GENO	29/47/66	15/87/75	13.29	2	1.30E-03
15	rs29012083	23,647,575	GENO	35/51/57	23/92/62	10.96	2	4.20E-03
15	rs29012086	23,647,658	GENO	35/51/57	23/92/62	10.96	2	4.20E-03
17	rs29026692	66,954,281	REC	5/136	22/154	8.06	1	4.50E-03
18	rs29016029	41,705,003	GENO	17/74/52	32/62/83	9.26	2	9.70E-03
19	rs29015011	55,229,010	REC	31/112	18/156	7.72	1	5.50E-03
20	rs29021984	56,612,844	REC	14/129	5/172	6.87	1	8.80E-03
20	rs29009836	22,715,561	DOM	89/54	135/42	7.42	1	6.50E-03
21	rs29017681	14,702,969	REC	31/111	18/159	8.24	1	4.10E-03
22	rs29016333	40,957,965	GENO	35/58/50	36/104/37	11.54	2	3.10E-03
23	rs29013434	7,339,117	REC	23/120	51/126	7.21	1	7.30E-03
23	AAFC02076530	40,983,286	GENO	18/83/40	36/70/69	11.29	2	3.50E-03
26#	rs29017074	23,326,504	REC	42/94	31/143	7.24	1	7.10E-03
