# Supplementary material for: A 2cM genome-wide scan of European Holstein cattle affected by classical BSE
Source: BMC Genet. 2010 Mar 29;11:20. doi: 10.1186/1471-2156-11-20 (PMC2853485; doi:10.1186/1471-2156-11-20)

Additional Figure 1 – Multi-dimensional Scaling (MDS) of the multiple family sample set and unrelated sample set.

Multi-dimensional scaling (MDS) of the matrix of genome-wide identity-by-state (IBS) distances was used to provide a two-dimensional projection of the data onto axes representing components of genetic variation. Animals whose genetic ancestry differs significantly appear as outliers on the MDS plot. To avoid confounding the multi-dimensional scaling by extended linkage disequilibrium, the genotype data were reduced to a set in which no pair of SNPs was correlated with r^2 > 0.2. For this set of SNPs, the genome-wide IBS pair-wise identities between each pair of animals were calculated using PLINK (Purcell et al. 2007; Version 1.04). These IBS-relationships were converted into genetic distances by subtracting them from one, and the matrix of pair-wise IBS distances was used as input for multi-dimensional scaling.

MDS plot of family sample set

MSD plot of unrelated samples

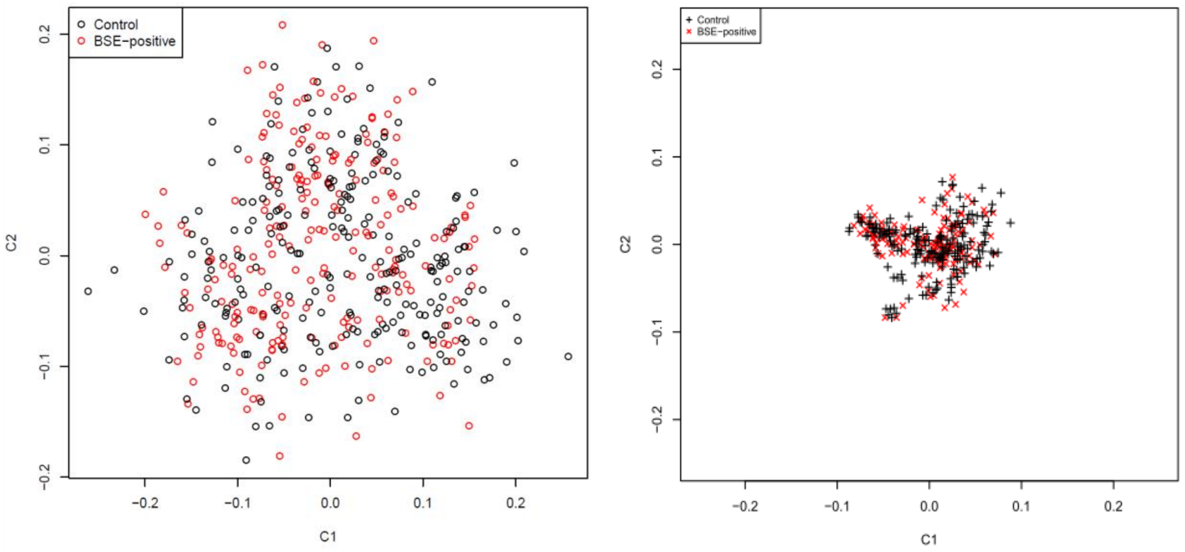

Supplement: Additional file 2 — Multi-dimensional Scaling (MDS) of the multiple family sample set and unrelated sample set. [file 1471-2156-11-20-S2.PDF]
