# Supplementary material for: A 2cM genome-wide scan of European Holstein cattle affected by classical BSE
Source: BMC Genet. 2010 Mar 29;11:20. doi: 10.1186/1471-2156-11-20 (PMC2853485; doi:10.1186/1471-2156-11-20)
Supplement: Additional file 3 — Sequence information for the SNPs and the minor allele frequencies for the family and case-control sample sets in order of position. [file 1471-2156-11-20-S3.PDF]

Additional Table 2 – Sequence information for the SNPs and the minor allele frequencies for the family and case-control sample sets in order of position.

CHR	BP	Name	Family	Case-control	Top Genomic Sequence
1	369242	BES1_Contig627_2082	0.26	0.21	TTTGTCATTCAAGGTGATAAGAATACCTGACTCATTGTATTGGTGTCATGAAATCAAATAGCTTCTTTAAATTCCTAGGCCACAAGACAGTAAATTGAAA[A/G]TAAGAAAGATAGGGAAGAGTGTTTTGAACAGGACAGCTCAATGAGCTCTTGCTTTGATCCATCCTTTTGCTCTGAACACAGAGCCATGACAGATAAGGTG
1	369418	BES1_Contig627_1906	0.26	0.21	TATTCTTATCACCTTGAATGACAAATGAGCAAAGCAAGAAACAGAGTTCAAGTGCTTCACTTCAGGTTGAGTGAACGGATGGTTACCTGTGAGCCTGTGC[A/C]TCACATTTTATCAATACTTTCTATCACTCTTGCTACCAAGAGAAGGAGAACACTGAACAATCACGGCATTCATTAAAGACAACTCCCCTGACCGATTTCT
1	2608864	SCAFFOLD145634_10395	0.21	0.18	GGGACCTCTTAGGAAGAGTGGATTTCCCTTATGGGAAGAGGACTGGAAAGGAAGCCAGTTGTTATGGGACATGGAAGTAGCCAGAGACAAATACTTACTC[A/G]CTTTCCTGAGCCCTTAAGAAATGGGTGGCTCAGATGGTAAAGAATGGTAAAGAATGGTAAAGAATCTGCCTGCAATGCTGGAAACCCGGGTTCAGTCCCT
1	3083187	SCAFFOLD135069_20810	0.29	0.47	CGCCCAGACGGTTGGCCAAACATTACTCTAGGTGTGTCAGGGAGGGTGTTTTCTGGATGAGATCCACATTTGGATCAGTAGACTAAGGAAAGTAGACTGC[A/C]CTTGCTAATGTTGGTGGGCCTCGCTCAATCCACTGAAGGCCTGAGTAGACCCAGAAGGCTGACCCTTCTGAGAGGAAGAGGGAGTTCCTCCTCCCTGATG
1	3083235	SCAFFOLD135069_20858	0.28	0.46	GGGTCTACTCAGGCCTTCAGTGGATTGAGCGAGGCCCACCAACATTAGCAAGGGCAGTCTACTTTCCTTAGTCTACTGATCCAAATGTGGATCTCATCCA[A/G]AAAACACCCTCCCTGACACACCTAGAGTAATGTTTGGCCAACCGTCTGGGCGCCCCCATGTCCTTGTCAAATTGACACATAAGAAAAATGCTCACAGGAT
1	3083367	SCAFFOLD135069_20990	0.29	0.47	GTTTGGCCAACCGTCTGGGCGCCCCCATGTCCTTGTCAAATTGACACATAAGAAAAATGCTCACAGGATGGGAGTGTGTGTCCTCCTCTGGGATCTTCAC[A/G]GTCCCTGGGCTGATCTGGCTCATCTTCTTGGAATTCGCTAATGAGAAGATCCAAGCAGATCCACTTGTGATCTGAGTGTTCATTGCTGCACCGCCTCTCC
1	3083498	SCAFFOLD135069_21121	0.28	0.47	CAGAAGGCACTCAGTATAATGAAATAAAGAGGCTGCACATAAATGTAAAGTAGCGTCTGTCCTTTGTCTGTCAGAATGGATGGGACCTGGCCAGGCATTA[A/C]TATTACGTAGAGAGATGCCCTTGGGAGCTTGGAGAGGCGGTGCAGCAATGAACACTCAGATCACAAGTGGATCTGCTTGGATCTTCTCATTAGCGAATTC
1	3083543	SCAFFOLD135069_21166	0.29	0.47	GTGTTCATTGCTGCACCGCCTCTCCAAGCTCCCAAGGGCATCTCTCTACGTAATATTAATGCCTGGCCAGGTCCCATCCATTCTGACAGACAAAGGACAG[A/G]CGCTACTTTACATTTATGTGCAGCCTCTTTATTTCATTATACTGAGTGCCTTCTGTGGGAAAGGTAGAAGCTGAGGGTCAAGGAGGACCCAGGGCAAGAC
1	3532059	SCAFFOLD15020_24467	0.14	0.04	AGTCTTGCCAGCATCAGGGTCTCTTCCAGTGAGTCGGCTTTTCACATCAGGTGGCCAAAGTATTGGAGCTTCGGCATCACTCCTTCCAATGGAGTACGCA[A/G]TACTTTAAAAAAAGAAAAAAGTCTTTTTCTGTCTTTTTGGGGTGAGAGTATAGCTCTTCAAGAGAACCCTATGAACGTAGGAGATTTGGTTGTAACTCCC
1	5629481	SCAFFOLD155434_15955	0.10	0.16	CATTGGAGAAGCCCCAACCTTTTCTTCAGAGAAGATTGCAAATACCAAAAACCTATAGTGAAGTGAGATATATACAAGCCTGGGGAAGTACCAAGAAAGC[A/G]GAATATGATAGAAACAGGAAGAAGTTATTAAAAGGTGCCAGACCTCAAACAGAAGCAGACTCTTTCAGCATCAGCCAACTTCCTCTCTCCTCCCCAAAGC

1	5800028	SCAFFOLD45728_8109	0.17	0.30	TGGTCCCCTCCCTTCTCCTCACCTGTGACTCAGCAGCTACTGCTTTCACAGACCGCTTCACTGCATTTTTATGCTTTAAACTGGCTCTTCACAGGGACC[A/G]GCCCCTAGCGGCCTATCTTGTCTTGCTTTGGCTGTAGCACATAGATACCGATAGCTCATGTTTCCTAGCTCCTTTTAATGCCATGTTTTCACCATCTTTA
1	6350258	BES10_Contig712_1090	0.14	0.26	GACCCGCCCCTGCTGTGGCTTCGCCATTCCCAGCTAACTTGGTCTTCCCCATGTAAGGGCTCTGGATCCTACTCCTAAAAGCTCTAATTTGGGGATCAGA[A/G]CTTGAAATAGCCCCAAAGGTATCCTGTTTAGCTTGTCATTTAAATAGCTAAGCCCCTGCCATGGCCATTTAAAACCCTCTCACCTTTCATTTAGCTAAGC
1	7967612	SCAFFOLD10253_3415	0.11	0.11	AGTAAATAAAAATGTTATAGGACAAATTCTCAAGTACAGTACTGTGTCTGCAAGCAGGAGAGGTGTCTAAACCAGTGTTATAAGGATAGTAAAAAAATTC[A/G]TCATGGGGGTAAAAATGATCAAAAACCTATAAAGCAGAATCCTTCTTATTTGGGAGGAAAAGATTACACAGTGGAGGGAGGTACATTATGCACACACTAA
1	8066806	SCAFFOLD101174_2389	0.16	0.25	AATACATTCACATTGGTTAGTAGGTAGCACATTGAAGAATACTTTCTATTGTTCGTTAAAAATATATTCAAGCTATGAATTTTGGTTAAAATATCTTAAA[A/G]ACACTGTCTAGTCTTGCATGAACTGGGCAGCAAACTCTAGTGAGATGTGTAGTGCAATATTAGGGTTATATCTCTAAGTTCCTTCCCCCCAAATCATTGA
1	8781962	AJ496763-027.SP6-290	0.17	0.13	ACACAAAAATCGGCAAAATTGTTTCCATCGTTTGCTTTTTGTCTTCTAGAAGAGGGTACTATAGATCTTCATGTGATCTAGGGAACAAGTGCAATAGACG[A/C]AATCTGCGGATCTCCTTGGGTTTCCAAAAGAAAGATAAACAAAAGTGATTGATATGAAGGTTGGGAGTGCTAGACCTATCCAGAAAATTGGTCCAAAGCC
1	8782101	AJ496763-027.SP6-429	0.40	0.38	CATTTATTGGTTGGGAAAATTTGTTGCCAAGCCAAAGCGTCTTCCACATTGTACTCTTTCCGTTCTGCCAATGTCTTAAGAGCTCATTTCTGTCACAGAA[A/G]TTTGCATATGACAGAAGACTACACTTTTATGCTGGAGGGGCTTTGGACCAATTTTCTGGATAGGTCTAGCACTCCCAACCTTCATATCAATCACTTTTGT
1	9842726	SCAFFOLD118127_10627	0.49	0.47	TGAAGGACAAATGACATGCAGCAGAGCTGGGTTCCCCTTCTCAAGACTCCTCTTCAGTTTATATTTCTCTCATTTTGGAGTTCAGTGCCCCTAAAATTAT[A/G]GGGAAATTGCCATATGTGATGTCGGCTGGAAAACGTTTCAAGAGACTTAGGTGGAAATGGTTTAAGTTCCAAGTCTAAATGAGCAGTGAACTGTTAGGCC
1	9842903	SCAFFOLD118127_10804	0.20	0.24	AAATGAGCAGTGAACTGTTAGGCCATCTCTGTTTTTCCTTGCAAAATTTAATAGTCTCCTATAACCCTAGAGCTCTCAATTTCTTTGTTATTCTATAAAA[A/G]AGAAAGGAAATACTTCCTAATGCACTGTTGCTGGCCTCATCCTATTTTTTTAAGGTGGTTGCTGTCTGTCAAAACAAGTTCTTTGCAATTTTTTTTTTTT
1	11260938	SCAFFOLD139165_5836	0.35	0.26	CCACTGGCTGTGTACTCTGTGGATACATTTGTTATTCATCTTGCTCTCCCCGTGAGTAGGGTTATATAAGCTCAACTCAAATAGAAAATTCATCACACTG[A/G]AATCATCACCCTGCACTCAAACCCCACTGTACCACCTGCTTTTGGAGTCTCTGTTATGTAGGCACCAGAGAGATGGATCCCTTGATCATGGGGGGTCTTC
1	14765723	SCAFFOLD100977_3398	0.31	0.25	CCCTAGGGCACAAGGAGCCCACTGATGTCCTTAGAACAGGTCATGCACATGCGGCAGGTGACAAGGCAGAGAACACTGGCATGTGAATCAAAAGTAACAA[A/C]GGGCTATACAGCAAGTAAAGCATCCACGTGGTAAGGACTTCAAATGGAAGACGTCTGTATTCTGTTCCCACGTCTGCTGTTCTAGTTCACTGCTCTGGTG
1	16867516	SCAFFOLD100859_24259	0.24	0.43	ATTAGTCCACCAGACTTAATAAAATGCTTTATCAACTTGAATTAAAACTACTACATTTAGATTTCAGGTATTTTGTTTGTTTGTTTTATTTATTCTGGCT[A/G]TGCTGGGTCATCATTGCTGTACTCAGGCTTTCTCTAGTTGCAGTGCTCAGGCTTCTCAGTGTGGTGATTTCTCTTAACGTGTAACACATGCTTTAGGGCC

1	16957942	SCAFFOLD202527_775	0.24	0.43	GTGTGCATTAAAATAAGAAGAAACACCTGATACAAATTGTCTTCTGAGAATAGTACACCCAAATAACTGAGGTATAAAGTTAATATTAAATACCAATCTC[A/G]ATGTTACAAAAATCACTGGCTTTGCACAGTATATAACTTAAGTTAATTTGCTTCTCTGAGTATAACAAAATCTTGCAGGTAGAGTCATCAGTGACTTATT
1	17205046	SCAFFOLD111013_10074	0.49	0.01	CCGATAGATAAAACATATATCTTACCTCACAATTTTTAAAAAGATGTCTTCTCAGACTATTACAGAGTAAGAACTTTGTTGTGCTGGTAGCCAGAGCACC[A/G]TGCTATATAATATAAATACACCTCTGAGTTTGACTGCATGTATTACATGTAAAACAACGGAAGTGAATCTAACTTTACCTTACCTTACTAAAGTAAGGCT
1	17205133	SCAFFOLD111013_9987	0.31	0.22	TAGCCAGAGCACCATGCTATATAATATAAATACACCTCTGAGTTTGACTGCATGTATTACATGTAAAACAACGGAAGTGAATCTAACTTTACCTTACCTT[A/T]CTAAAGTAAGGCTAATTGTGAAGCGACAGCATCACCAAATGTCAGAAATATTCTTATAATTTTTATTCCCAAATCCTTTGATATCACATTAGAAAATGAA
1	19874286	BES5_Contig533_1201	0.05	0.13	AAAAGGGAAAATATAATGGGAAAGACTGTAATTTTATAGTTCCTGTAAAAATGAGGAAATCACTGCCTTCGCAAAACCCCCAAGTGGTATGGCCCTAAAT[C/G]TGCCCACAGAGAGACCAAGATATATGTTTTTAGCTTTGAGGATGACCCTAGAAACCTACTTGGGGTTTATTTTAGTTTACTCTAACATAACTATAGATCC
1	19877647	SCAFFOLD280362_12193	0.07	0.15	AAAGTAGATCATTATTGGCCAAAAGCACTCCAATTACTTTAGAATTTACAGAGGAAAGAAAGGAAGAAAAGTCCAAATTGTTACTTGGAAAAGATGGTCC[A/C]CATATCTCCCTAAAACTTATATTTTTAGAAAGCTTTGTAATAACTATTCTAGACATAACAGGCATTATTCAAGATTGATATATTTCAGGAGGCAGGTTAT
1	20870762	SCAFFOLD15156_8027	0.23	0.29	TTCAGTCCATGAATTTCTGGCTAAACAGGTCACTGCTAATCCATGGTTCTTCCTATCCAAAAAGTTTCTTCAGACATGCAACCTCTCATATTTTTTTTTC[A/G]GGTATGTAGGATTATTATACTGTTCCTCATCACACACATCACAAAGACTTACAGCATAAGAAGCTGCCTAGGACCTCTTGTTACTTCCTTGCTTCTCCTA
1	21011530	SCAFFOLD101124_11326	0.39	0.45	TGCAATCTTGGAATCTTGCTCATTTCTTTAAAAAAAAAAAACCTATTCTGATCATATTAAAGATCAGAAGTGGGCAAACTATGGCTCACGGGCCAAATCTA[A/G]CTGGCTGTCTACATTTTTGTAAATCAAGATTTACTGGAACACAACTGTACTCATTAAATTATGTACTACCAGTGGAAGCTTTTACACTGTAACGCTAGAG
1	21961798	SCAFFOLD175878_8238	0.49	0.04	GGCCACTTACCAAACTCAGGTGATTCCAGGATGGTTTGGCAACTCCTCCTGAATACATCCTTAACTTCTGTGAAAACCATTGTCCTAAGAACAGTGCTGA[A/C]AAAGATGAAAATATATATTTCACACCCAAGAAAGGCACTAAACTCAGATTGCTTAAACAAAAAAGTAAAGGGCACATATACACGGCAGAGTTGTACACAG
1	22034495	SCAFFOLD240108_5214	0.09	0.11	TTCCTGCCAAGTTCCCTGATGACTGGACTGACAGGAAAGGTGGGCACCCAGCACTTGACACTTCATCGTACCCTGCTCTCGAGAGGCTTATAATTTTGTG[A/C]AGGGGAGCGATCATAGTTCACAAAGGATCAGAGAGCAAGGATGTGGTGCCTGCACTGCTATTTCTGTTTAGTGCGTTTTTCTCTCTCTGGAGTTTCTCAT
1	22034662	SCAFFOLD240108_5381	0.01	0.05	ATGCTTAAAGTACTCTCTGTTCAACAAAGGCTTGTTGACTGAATTTACAGCCTGTGCTGTAACATGAACTGTAGACCGAAGTCCAAATTACAACACATGA[C/G]CTCTATGGCAAGAAAGAGTGAAAAAATAAAGCAACCCTGGACACACGCCAGATGACGGCCGGGCCCATGAGAAACTCCAGAGAGAGAAAAACGCACTAAA
1	24266138	BES1_Contig415_1302	0.23	0.24	ATGAGCTTGGACATCAATAGTTTAAGCTGCAGTGGATTAAAATGATCCTTCTCTTTCATGGTGCCGTGAGGGTAGAATCTGATCATCAGTGAATTCCCAT[A/G]TTGTCAAGACAAACAGACACAGGTGAATATTTCGTGAATAACAGTAAGCACAGGAGCATACACCACCACAAATGCACCTCTCTTTCTCATGCTATTCATC

1	24785995	BES10_Contig599_741	0.49	0.40	GAAAGTGTTTACCCTTTTAAAGTGAGTTTAAGGGTTATATTTGATTGTCTTTAATTAAATATAACAGAAATACATCTTCAACCAATTTGAGCTGAAAAGG[A/G]TTTTTGTTACAAAGCTTATAGAGTATGTCACTGATCCAAGTAGGATGAGCCAACAAGGGAATTCAGACCCCGAATCTTTTTTGTCTTTCATACCTCCTCC
1	24908254	SCAFFOLD171493_3192	0.15	0.22	CACCAAGAGAGCTGAGGCCTAACTGGTTCAGATAGAGTTTCAGTTTGAACTCCGATGCTCCCTGTGTTTGGAGCAGGAGGAGCTCGCGGGTCCTCACACA[A/G]ATTGAGGCTGACTCTGATGCAGCCTGGGTCAGTGGCAGCCCCTAGAGATGGCAGCTGTAGCCCCAGGTGGTAAATATATGACAGCAGCTTGATCTATCGA
1	26843935	SCAFFOLD100386_1610	0.18	0.11	GATTACTCATTTATATATAACATATTATTTATTTATAAATAAATAACAACTAAAACTTAAGCCCTTAATTATCAAACTTTTACAAGTAATCTTTTATTTT[C/G]CAATGTATTTTGAAATATGGATTCTGGAATAAATTATGAAGTAAAAATAATATGGAAATCTATATAATATGGAAACAAGTACAAATGAGTATGTGTAGCT
1	27579797	SCAFFOLD105346_12308	0.14	0.26	TATAAAAGGGATTTTTTGTAACATAAATCTATAAATGTGACAGGATTTTTTATATAGATTAATAGTTAATAGGTAAGTACACATATTCATAAACTCCTAA[A/C]AGTTCTTAAGGGTATATGCATGCATGCTCAGTCATGTTGGACACTTTGCGACCCCATGGACTGTAGCCCTCCAGGCTTTTCTGTCCATGGAATTTTCCAG
1	34347534	BES10_Contig592_452	0.38	0.42	AAAAATAAAATAAAAAAAAAACAAAACCAGAAACATGTTGTTGTTAACAGTGACTGACACAACATCTGTGAAATTTTCAACCAAAAATTTGCTTAGCCCA[A/C]TGCTTTTACTATGCTACTGCCATTGTTTGCTAAATATCCAGACTTCATTCATTTATCAAAATTTCAGCATCTTATAATGCAACCAAATATTTATCTAGTG
1	35152734	SCAFFOLD131987_5638	0.38	0.41	CAATCATGGTACGCTCTAGGGCTAGTTCCACATTTAATGTTAAAAATACTTTTTGTAGGCTACAGTTGCAAAAAAGTAATATGCAATTTTAGTTTACAGA[A/G]AATGGTTAAAATAAGCAAAGCTGCCCATTAATCAAGCCCTCACTCCTGAACAGCAACATGGTAGCTTGTAAGCATGCCTCCAACTTCTTGAGACAAGCAC
1	35153163	SCAFFOLD131987_6067	0.38	0.42	AAGTGAGTGTTTTATTGTGTGAAATGGTACTCTAGGGACTAAATTAGTCTTCCACACTGCCTAACCCAAGGAAAAGCCTGGTGCATTATTAACTGTAAGC[A/G]CAGGGTCAAATTTTACTAGAGAAATGGAATTGGATCAGTCTTCTGATTTTATCTCTAAACTTTGAATATGTAACTCCAGCAATAAAGCATCTCAAAAAGA
1	39379543	SCAFFOLD305427_17567	0.25	0.22	CTGATTGCCTTGAGCCATTATTACAGCTAGCTGTTCATTTCCATTTTAGCCAGCTTGGACCATTGAGTTGCATTAGTATTTCAATTTTACATGAGGTATT[A/G]GAAAAGCCAAGTTGCAGCTCAGGCAAAATGGAATTTTAAAGTGCACAGTAGGCTTTGTAGAAATGTTATGGAGGTCGAAGTTGTTATTGTTGAGAAAGTA
1	41793196	BES7_Contig453_1164	0.16	0.23	ATGTCACTAACAAAAAAAATCAAGCAGAAATGTGCATTAAAATGATATATAACATAACTACATTTACTTAAGAATGTATTGCAATATCAAGCAGCAGATT[C/G]AACAATGCAGAACTGCATCAACCTAACACTAGCAATTTAGTAAAGATAAAGGGAAATAATTTTAAAAACTATAACAAAAAATCTTGAGTTACATATGGGA
1	43944468	SCAFFOLD148828_1609	0.09	0.12	TTATGAAATGAAAGGTGCCTTATATTGGTGTGGTATCAATTTTGTTGTACATGAATGGTGAAATGAGTCATAATATCTGTCTAGATTCAAAGGGTGGAAA[A/G]ATAGACATCTCCTGGTGGAAGAAGCTACGATGTATTATAGCCACTTTTACAATCTAGCAGTCTATCCTTCAGAAAAAATTACCATTTCATCCACGTGTAA
1	44240247	SCAFFOLD60571_5732	0.34	0.40	AAAATCATAAGGAACAGTGATCAAAAGTGGGTTTCAGTTTCTTTCCATGAAATCTTAGTATAGTTTAGCAGAAGCACAAGATATAAGTGGTATTTGGGAA[A/C]CCCCTGTATTCTATAATGTGGTTAATGTTGTCATACTGTCTTATCTAGGAATTTTTCTGTGCCCCTCTAAAGTGTCAGTCGTCACATTTTAAAATGTTTA

1	44640564	SCAFFOLD112638_2979	0.19	0.28	AATTTGGGGAGTCCCTTCCTTCTTGGTCTCCTGGGTGGTCACTGAATGAGTCAGATGTCCTTTTCTCATATCTAATGCATTCTTCTTTTTGTCAGTGACT[A/G]TTGCCATGAAATATAATTATCATTTCTTTTTCCTTTATTCTTGTTGCACACTCATACATTGCAATGCCAATTGACGTTTCAAGGGTAAGTTGTTTGTTTT
1	44640845	SCAFFOLD112638_3260	0.14	0.13	AAGTTTTAATTTAAGAAGATAATTTTCCTTTAACATTTTTTGTTTGAAATCTGATTTACTTGCTCAATGTGCTTTCAGATTTCTAGGTTCCTTTCTGGCC[A/G]AGTTCCTTAACAGCCACTGCTAGAAATATGCTTTGAATTTTGTGTTTTGAGAAATGGATCATTTGTTTAGAACTGCCATATTATTTTGTTAGCAACATAA
1	47153415	SCAFFOLD15708_1407	0.10	0.14	TCAAATTCGATACTGCTGTCATTTGAAAACTAACGATTACACTAATTATTTAATCTTTGTACACTAATGCATATTCAAGTGCATGTGTACAGACACACAC[A/G]TACCCATAAAGTATGTAGAGTGAACATTTTAAGATACATTTTTTTCTTCAAAGATATGTTATAAATGACAATCTGTTTCCATGCTGACAGACTGCTTTGT
1	47153531	SCAFFOLD15708_1291	0.09	0.13	TTGAATATTGACAATTTCATATAGTTCCTATTGCATAAGTGAGTTGAATCTGAAGTGGAAATGGGCGGTGCTGCATAATCCTTTTATGATGAGTAAATGC[A/G]TGGTAAGAGTAAACAACAAAGCAGTCTGTCAGCATGGAAACAGATTGTCATTTATAACATATCTTTGAAGAAAAAAATGTATCTTAAAATGTTCACTCTA
1	47153850	SCAFFOLD15708_972	0.30	0.33	GAGGGGACAGGGAATTAAAGCTTCTGATAACACATGACATTCATCATGTCCGTTCATATTCCACTGACCAAGGCATGGTCAGGGCTCATGTTCATAGAGT[A/G]GGGTGTCATTAGTATGAGCCACAGTATTACTGTTTATAAAGGGCTGCAGCTGCTAAGTCGAGTTAGTCATGTCCGACTCTGTGCGACCCTATAGATGGCA
1	48836494	SCAFFOLD130531_7174	0.26	0.21	TGACAGCCATTAATTTTTCAGTTGGTTTTTGAAATGGGGAACAGAAGGACCTAGGTGATAGGAGGAAGGAGTGGCTGTTGCCAGGAGTGGTTGTTTGAAA[A/T]CCTGATCAGAAGACTTTCACTACTGTAACTTCTAAGGCCAGGCCAATGAGTTCTGGCTAAGTGTTACTCTTGCCTGTAACCCGTTGCTGCTTTTTTCCCC
1	48836636	SCAFFOLD130531_7032	0.22	0.13	ATCACCTAGGTCCTTCTGTTCCCCATTTCAAAAACCAACTGAAAAATTAATGGCTGTCACATTGGGTGGCCCGCAGAAATCCCTCTAGGCTTTAGCTACA[A/G]GTTTTCTGGATTAGCTCTTACTGTGCCATATTGGTTGCTTTTTTTTTTTTTTTTTTTATAATCAGCTCCTGGATCAAGGCATCTGTTTAGTAAATCCACA
1	48836736	SCAFFOLD130531_6932	0.28	0.21	GGTTTTCTGGATTAGCTCTTACTGTGCCATATTGGTTGCTTTTTTTTTTTTTTTTTTTATAATCAGCTCCTGGATCAAGGCATCTGTTTAGTAAATCCAC[A/G]GAGAGGTTGTCCAGCACCTTTGAAGTCATACTGTGTATGGTTGCTGGAGAATGTAAGAAAAGCTTAGAAACAAATGAACAGTACACAGAGAGAGTGCCTA
1	48836871	SCAFFOLD130531_6797	0.27	0.21	GTATGGTTGCTGGAGAATGTAAGAAAAGCTTAGAAACAAATGAACAGTACACAGAGAGAGTGCCTATGTTATTTCTGAGGTTTTTGTTTGGGAGGTAGGT[A/G]TTTTCCTCCAGTTTTGGAATATATTTCGGGGGAAAATGACTAGTTATTCTTGTCCTGAATAAATTTAGTCAGGAAGATAACACATAGAAAAGGAGTGAAG
1	50698307	SCAFFOLD786_9238	0.24	0.21	GATAAGCAGATTGAAGTTTGATAGGAGAAGGGAGGGCTTAGAGGAAAGCATTGAAAGCAGGGACCATATCACAAGAATAAAGGAGGAGCAAGCTGTTCCT[C/G]CAGGAAATAATAGGCATTGAGCTTCATTCTGGAAGACCTTGAGTGTAGATGTGAGAAGACACAAATAAATAAAAATAATGGCAATCACTCATGAGTGCTT
1	50853397	SCAFFOLD134433_935	0.27	0.15	TGTCCACACAACACATGGAAAAAACCTATATTTCCTAGTTTACCTTTCGAACATTTGCCAACCGATTCATCATCAAGGACTCTTCCCGGTCATCATCATC[A/G]TCCAAGTCCAGCATCGGCATTCCAAAAGGGTCGATGATGCTACAGCACCTTGATCCTTCATCCCTCGGATCAAAGGGGTCCACGATGATGGGCTCCGTTC

1	51818942	SCAFFOLD134530_3084	0.45	0.38	TGATACAGGCACACTTTCAAAGAACACATAGTTTAAATAACTGTGTTGATTTTCCCTGTCTCAGACATAGTCTTTAGCATCTACTCTAATCAAACATAAT[A/T]GGATTGTGTATGACTTAAAAGAATGTCTTCTTCATTCTTTTTAATTGACATTTTATAAAGTAACACTCATCAGCCTTTAACAGCAAAGCATGGTATTTAA
1	52539685	SCAFFOLD34530_945	0.47	0.39	CAGCTGACACTGTCAACACTTTTTAAAGATTCTTTTGATGATACCAGACTAACTCCTTTTTTGTTATTGTAATGAATTCATTACTGAATTCATTGGATTA[A/C]TTGATTAAAAATGTCCTATCTTGGTATGAAATTACATTAAATTAATTAAATTACATTAAAATATATCCTATATTATGACAAATTATATGTTACTTCAGTC
1	52539818	SCAFFOLD34530_812	0.40	0.41	AAGTGTCAGAGTGGTTATTCTACTATGATAAAAATTCTATATTCTTTTTAATTTATAATAAGAAATTCCATTATAGTAGCACCCTGACCATAATTGCTAA[A/C]CTTGGACTCAGTTATTTAGTCCATTTTACTCCCAGCTGACACTGTCAACACTTTTTAAAGATTCTTTTGATGATACCAGACTAACTCCTTTTTTGTTATT
1	54371282	SCAFFOLD106669_1083	0.39	0.46	AATTTCTGTGCCATCCTTACTCAGAGGCCACGCTAATCCTCTTGGTATTGCTCCAGTTTTAGCATATGTGTTGCCCACACAAGCCCTCACTACTGATTTT[A/T]TTAACATGGTTCTGAAGGTACATGCGAGTACAATTAGAGAGGAGATTATGAGGTGTATATTATATACACATATATGAAAAAGGTTGTAATATTTGCAAAT
1	57065397	BES2_Contig240_869	0.33	0.28	AATGTGGAATCAGGTCATTATTTGCCAAATGAATAACAAATGTACAGAGGTCATACTAATTGTGATGGAAGGTCAAGTGATGATGAAGATGCCTACCAGC[A/G]TGCACACACATGCTAACAACTGATGCCACTTGGATAATTCCAAGAACCTTGGGGTGGGGTAAGTAGAGATAGCGGATGGTTTTCCTGCCAGTTAAACCGA
1	57962943	SCAFFOLD312466_13255	0.07	0.09	TTTATTTTAACCCAAGTCTGGAAGAAAACAAACAGGGCATTATTTATGCTTCCCTGAAGCATTCGGTCATTGGAATGAACCCAAGACAGGCAACACATGT[A/G]CAAGAAGCACCTACGGAGTATGCGACCATATGTGTGAGGAGCTAAATCCGGTCCTGATCTCCCACCAGTGATCAGCAACTGATCTGTTAGTCAGCACCAC
1	57963228	SCAFFOLD312466_12970	0.03	0.05	TTAAGGAGTAGATGTCAACACCTACAATGAAGAATAGATTATTTCTCTCCATGTATAACCACTCAATTTAGCCTACACTCATGGTTTCCTAGTGGATATG[A/C]ACTATCACATCCTTAAGTTGTAATTAAAGAAATCTTTTTTTTCCTCTCTTTTAGGTTGATGTTCCCCAGTTCTTTAGGAGTGAGCCAACTGAAATCGACA
1	59101518	SCAFFOLD9563_462	0.34	0.47	GGAACTACTGCCTTAGAGTGACGTTTGGAGAAAAACAAAGGAGGGAAATCCTTCTGGCATTAAGTACATAAGAAGGGAGGAGGAGTCTGCAGCCTAGGTA[A/G]TGAAAAGGGAAGCAGACAAGCTAAAGATAAAGCTGGCTTGTTCACAGCTTTCCTTAGGCTTTTACCTTGGTTCTGACTTCTATAGATTTAAGCCAGGCAA
1	59127766	BV105557-406-K	0.33	0.45	TTCCTAAAATTTGGTGCCTTGTCCTCTGAAGTCAAGGTCACTTAATATTAATATTAAAGAGAACATAAAAGATCTGTCTTAAAGTATATCACACCTTTCC[A/C]ATATTTTACTAATACTTTATGAGACAGAAAGGGCCTAATGACACAACAAAAAAATTCAAAACATAGAACAAGCAGTAGGTTCCTATTTTTCTTGTGCAGTT
1	59799327	SCAFFOLD130001_59457	0.46	0.47	CACATTTCAGAATAATTTTGATAACTATTCAAGCCTTAATGAGCTCTTCTAAAACAAGTAAAGCCACTTTCACTCAAAGGCATAGGAGACAATTAGGAAG[A/C]ATTTTTTTCTTGCATTTATCTTTTTGAAAAACAATTGTTGATTGGTTTAGAACTTTAAGCAGTAATTTACTAAGGGATGAAAGGATTCCTAAAGTTTCTA
1	59825081	SCAFFOLD130001_85195	0.24	0.28	ATTAACAGACCTAATAGAAATTGGAAGGAAAGGGAATTCTCTTACGTAAAAATTTGCACAAATCATATCACTCAAAGATAACATAGAGCCTGTAGGCAGC[A/G]TTTATTAAGTTTAGTGGATTTCTGTGAGGCTGACTGAATGTAAAGTTGGGAGCAAAGGAAATGAATGAGTATATAAGGATGCAATCACAACAGCTGAACT

1	59899502	BES10_Contig701_1260	0.19	0.17	GCAAGACACCTTTTTACTCAGAGCTGGTCCTTTCCATCTCCAATGGCTGCACTGTGGGGAAGCCACAGAATGCATTCCAATACAATCCCACAAGCCAGTT[A/G]TACTGGGGATAAGAAGCATCTTTAAATGCAACACTGATTTCATTATTTCTTTAACTCCAAGGAGAGCTTCAAGGATGTTCCTTTCTGCTGACAGCTCTGT
1	61609705	SCAFFOLD211013_2154	0.48	0.40	ATCCTTTAGGATGCTCTTTATTTCTTTTGCTCTACTGTACTTCTATGTGGAATTGTTCACCTCCTTACTAAAGAACTCCTCTTTGTATTTTTTAATGTTT[A/G]GTGTTGCCCTCCTGCTGATAAATTTTCTCAGTTTAGTTTGGAAAAATTGGCTTTGTTCAATCAAAATGTATTAAATATGTAAGTAGAATTTTCAGACAAT
1	63216052	SCAFFOLD15142_17808	0.02	0.07	TTATGTATAATGTTCACACAATCTGAATCATTACATATAACTCAGTTTCTGAAATCAGAATAATTTATTTGCTTCATCTATCTTTAACCTACAAAAAAAA[A/C]CAAACAAACCCAAAACAAAAGGGAATATTTTGCAATAATGCATGAATGACCCCCAATTTGTAAAAGAATTCTTATAAACACTATCATAGAAGTCATTTAT
1	68338491	SCAFFOLD105843_4589	0.21	0.06	GACAACAGGGATAACAACAGCCACCACCTTGTGCTCAACACATCCTCCCCCTAGGGAGAGTGATAAGTGGTTTGCGCAGATTATCCCAACCAGCCTTCAC[A/C]AAAACCCTGCAGAGATTTATGAGGAGGAAGCCAGGGCTCCGAGAAGTGACTTGTCCAAAGGCACACCATCCGTGACTGCGTCAGGATGCCACCCCAGGGG
1	68583890	BES8_Contig467_749	0.20	0.30	CCAGGCCAGTGGTCAGCCTTCCACTTGAGATCCTTGTCCTCACGGCCACCTCCAGAACGCCTCCTGTTCCAACACTAACAGTGTGCGGCCCTTTCTACCC[A/C]CAGCATGTCAACTGCTAAAGTCCCCAGTAACCTCCTGGGAGGGGGAGGCTCCCCAGTTAAAATATGAGCTGCCTCATGTGGCTTTATTGATTCAGTAAAA
1	68666549	SCAFFOLD51684_2891	0.25	0.24	GGTTGCCCAGCTGTAATGAAGTAAGCCTGAAGCTACCATACAGTGTTTTCCAATATGTGCAGAACATCTATCTGCAAAAGAAAAGACTGAAGAAATGAGA[A/G]AAGAATTCCTGGTAACATGTAAGTCCCTGGCTCCACCCAACTAGGAATCTGGCCTATCCTTCAGAATACCCTTTTTTGCTTAAGCAAGTGTGAGTTGTGT
1	74862396	SCAFFOLD40208_19734	0.24	0.31	TCATAAAGGAGAATAAATAACCTATGCTATTAAAACATATTGGTTCTTTGCCTAGCAGACAGATAATATTGATGCAATCCTATTATCATCATCTGCCATC[A/G]TCATTCCCTATTCGGCTTTGTGGGATGTAAATTTCCTTACTAGATGGAAGCCGCTCTGTCTGTTAAATTCCCGATACAAGGCCTTCTGGTAAGCCAGCAC
1	75104987	SCAFFOLD116048_9524	0.11	0.19	CTGTCTTGTTGACCAAATGCTCTGGACTCCCCAGGGCTGAGAGCTATTAGGGAAACAAAAGACTACCCTTTTAATTAGACTCTGCATGTCCTTGCAGGGC[A/G]GGGCGAGTCCGCTGAGTCCCAGTGGTTCTGGTGATTAGATGCAACAGGGGAAAAGTTTAAAACATCTGGGTGTGTTTATACCTCCAAAGCCATGACCGAA
1	76362570	BES10_Contig762_2071	0.01	0.04	GTTTGAACTCAAGCTTTTTGTATCCAGAGTCTGTATTCTTAATCCTCTATCATCCTTCAAGGAAACCTACCAATGATGTGCAATCTACACATACATGAGA[A/G]ACATACTTATTAATTGCTCTTCTTCCTTAATTCCCAGGAAATTTTGTCTCCCAAAGTTGATCTGCAGCAGGACGTATGCCTTCACCTATTGCCCCTCTTA
1	76825793	SCAFFOLD135046_16431	0.30	0.21	TTTTTAAATAGGCATCCTGAATTTGTTTTCATTTCCATTTTTTGATGACATTAGGGATATTTAGTAGAAATGAGGCCAGCTTGGATCTAGATTAATGTAT[A/G]GAAATAGCTTTAAAATTGTCTGGTATAAGACAATTACTTGCATCACTTATTTGATTAATTGTTTTTGTGCTTTTATTAAGAAATTTTGATTAGGACTTTG
1	78280009	BES7_Contig462_688	0.30	0.50	CTGACTAGAAAACAGTCTTTCTATAGCAGTACTGTGATTTCCATCTTCACTGGGGAGTGTGGGGTCAACATTTCATTTCTGATAGTAATAAAAGCAAATA[A/G]TCAAGAATCAGTATTTGCTCTCTTTTAAGGAATTAAAGCTTGAATGATTTTAGTAGTGATAATATAATTATAATGTAACAGTTATAGAGGTGATAATAAA

1	78280163	BES7_Contig462_842	0.24	0.22	ATATTTGTTTTATCTTGTTCCTCTGGACTCCTTCGTGTGGGCTCTTCTCTAAAGAAAAGTCCCAAATTTTAAAAAGAAATCACAACCACACAACAGTTTT[A/G]TTTCTATGTGGTGCATTTCAGATACCATCAACTGGACTTTAAGGCACACAATTCTGACTAGAAAACAGTCTTTCTATAGCAGTACTGTGATTTCCATCTT
1	78280263	BES7_Contig462_942	0.29	0.43	GCCCTTCCTGTGCTTCAGGTACCCATGTTAACGGAACCACAGGATCGATGTGAACAAAGAAAGATATCTTGAAAACCCTAAAGCTCTGTACATGCTTGTG[A/G]TATTTGTTTTATCTTGTTCCTCTGGACTCCTTCGTGTGGGCTCTTCTCTAAAGAAAAGTCCCAAATTTTAAAAAGAAATCACAACCACACAACAGTTTTG
1	79110245	SCAFFOLD145431_11892	0.09	0.18	CGATTGAAACCTACAGGCAGCAGCAGCAACAGCAGCACCAGCATTTACTTCAGAAACAGTGAGTGTATCCCTATGTCATTTTAGCAGATGTGATTAAGAT[A/G]ACTCTGTATTCAGATCGGCCACAGGATGGTTGTTGGCTGATGTAGAAGGTGAGGTTGTGAGACAGCAGAAATTGGTAGGTTCACATGAGCTGGTTTGAGG
1	82924030	SCAFFOLD295051_37454	0.46	0.49	GCAGGTCCTAGCTCTGCGCATAATTATGAGGATTTTAGGAATCGCAGAGATGGGTGGAAGGGGATGGGAGGATGGGAATGAATAGGACTATCCACTGACT[C/G]TCCACCGTCCCTCGGCTCAGAAACTAGAACTTGAGATTGTCTTCGCCAGGGGTCACGAAGTTTGAACTCGCCGACAGCTGCGGGCCGGCACAGTGGGGCC
1	83315142	SCAFFOLD140174_35098	0.14	0.14	CTGTTTTCAGTAAGAATCCAAATGTTCCGAACCTATATATGTGAAGGGCACACTTAATTTTTATTGCTGTTCATGGAGCAAGAAGGTCCACTCTTAGTTT[A/G]TTATAAGTATCTTATGTATCTCAAATCTGAATCAAATGTATAATAACCTTTAACCTGATCATTGTTCTTTTATGCATAAACCTTCATAAATGGCCTAGTT
1	84397541	SCAFFOLD5319_5197	0.47	0.34	AACTTCGGCTCCATTACCCTTTATGGGCTGCAGGGCTGGTGAGGGGAGCCGGAGGGGAGGGTTCTGGTGTGAGTCTGCTTCCGTGAGTAAGGAGCCAAAG[A/G]AACCGTAAAACAGCTTTCTCCCAGCTCGGTAAATTCACACTTTCAGCCCCTAGGCGGGGGATCTCCTCCAGCCGAGGTGAAGGGACGTGCCAAGGCTCCC
1	86159264	BES10_Contig437_406	0.32	0.42	AGATCATGTTCTTTAATCTGCAGAAACATAATTAATATTCACAATAAAAATACCAATACCAAATAACATGTAGGAAACCAGAAACAAAATTCTAAAAAAT[C/G]CCATTAAATACAGTTTGTAACTTGATAAAGATGTAGAACAAGAGTGCTATAATCTTGAATTGCATAAGTAATAGCTAGCTGGCTGCTTCTGACTCCAAAG
1	88005804	SCAFFOLD133267_4665	0.21	0.20	AAACTTCCTCATTAGTGGAAATTCTTTTAAAATACCATGAAGTATTCTCATTGCCTTAAAAATGTAATTTTTAAAGAATATTGTGAGGAAGAAAAATGCC[A/G]CAATAGAGCTGAGAAAGGTTTGTGTATAATTGTGACTTTTTTCCTGGGCCTCAGGCCCATAAATGCCTCTCCTTCCCAGCTAGCACAGTGGTAAAGAACC
1	88482214	SCAFFOLD270040_25442	0.48	0.42	TTGCCAGAGTGCACAATAAATACTCACTAAAGAGCAATTAGAACAAATAACCGTTGAGCATTCAATGAATAGGAAATTTACACATTTCCCTAAAGTAATT[A/T]GCATTCTTTTTCCTTGCTTTGTTTAAATTACCTAAATTACCTTTAATGCTGCCCTTAGTAAAACTGTTTAAAATAAGAGTCGTAAGAATTGGATCTTTCT
1	89733517	SCAFFOLD141856_5022	0.38	0.43	ACGTACTATGCTGCCTTGGGTTGCTTTGCAGCAACTCTTGGGCTTCCCACTTATATAGGTCAGTGCCAACACCATGTTATGGGACTGTTACACAGCCCAA[A/G]GAACTGAACAGAGTCAAAGCTTTATGCTCTTCAATTGCTTCACTAGAACAATTGAAACCATTTTGGAATCAGAAGAATTTCATCCATGTGATAATAATGA
1	90401967	SCAFFOLD105658_14572	0.34	0.31	ACTGTGATGTCACAAACTGGACGTGGCATTCCAAATTCCCACCTACTAATTCAAGGTCCAATGAAACCATCACCTTCTCTGATCTGTTAACACAAGCCTA[A/T]TGCTAAAGTCTGATCTCTTCTGGATAAAATATTTTGTCAATTCTCTCCTTTGGGAATATAACTACAAGGCACATTAACTTTGATGTCTAAGTGAGTATGT

1	90497927	BES4_Contig410_1232	0.10	0.24	AGGAGAGAAGGAAGTACTAGTTCCAGCATTGAACCAAGATTAAGAAAAAACCCAACCAACAAAAAACAGTTATTTCTTTTCCTGAATAATAAAGACATCA[A/T]GTTTGCATATAGAACAGCTTTTGCATTATTGCTCTACGTATAAGAATATATTAAAGAACTCATAACAGACTACACATTACAAACTTAAGGTTCTTTGGTG
1	92010421	SCAFFOLD195008_21951	0.18	0.15	TTCTATTTTTCATTTCAGTGTGCAGAATAGAATCATTCTTGAAATTCAGTACATTTTAAAGATGTTTCCTAGTATATTCATATAAGGAATTTAAAGTGTT[C/G]CTCTTGAATTTTCCCTTTCAAATCAACATGTATTAAGTAACACACTTGAAATTTTTATTAATATTATAGCAATATAATACCAAATGAGAAAAACAGAACA
1	92669977	SCAFFOLD151937_3161	0.37	0.46	AGGCTTGAAGTTTGCAGTGGACTGGTAATAAACTTAAGTAGAAATGAAACAGTGAAACAGCTCAGTCTGAGAAATGTGGCTCAGGTACCAGAAGTAAACA[A/G]AGATACTAAGGAAACAAAGAAGGATTTCTTGCTCAAGAAGGAATCAAACTTGACTAGGAATTAGGACCTGCGAAGTAACTTCATGCCAGCTAGTATGTTA
1	93806079	BES3_Contig167_494	0.16	0.15	GTTATATGATTTAAGTCTTTTTATTCAATGTTATGGACTTGAACAAACTTATAGAACTTAATGATTAAATGATAGAATCTGCCAGCATGAACCTTTGTGG[A/G]AAGTGACTGGTTGGTTTCATTCTAATTCATTTTAGAGAAGTTCTTATCACAGCTAAGCCGGAGAGGGAAAAAAGAGTTTAGAGGATTCCTATTATTTAAA
1	94369684	SCAFFOLD20021_18293	0.39	0.25	ATACTTGCAGGAAAAAGAAAATGACTAATCACCTACTAAAAATGGCAACAGATGCCATAAGCAAAATTTCATTCAACCTGTTAACTTTGGTTCCACTGTA[A/C]GAATCATTTAATTAAAAGTCTTAGTTTTTAAATATGATTTTTTAAAAATAGCATCTGCTTCTAGAATATGAGGAAATGAATTTTATGCTCAATATTTTGA
1	94370042	SCAFFOLD20021_17935	0.38	0.25	AAAAGTTCTAATGGTATAAAATTTACCACATTTGGTTCATATGAAATCATGGTTAATGTTTCATTAAATATTTTTTATCTTTTATATTCTAAATGCATTT[C/G]TGTTAAGGTCCCTGTGATTTTATGTGTGAAAAAACATCAAGTCCAAGGAATAGTAAATCAATTTTATGATTCAAAGCAGTACATTGATAAAATAGCAGAG
1	97260856	SCAFFOLD140039_11354	0.09	0.24	TGGAAGCATCAGTCCCATGACCTAGGGTTCCTGTGAAGGCCAAGTAAGAGGACTGATATGGATACACCTTGGGGCTCCACTGTGGTGCTCCCCCACTGGT[A/G]CAGGGGCTGAAGGCACAATGGGTAATAACAAGCTTTGTAGACAGACGAGTTTGAGTTCAACAGCCTGATGATCTTCACCAATGTAAGCCAAGTAAACGGT
1	97346722	SCAFFOLD280025_30949	0.19	0.15	CCGTGCTGATCTTTACAACCTCCTTGGCAAGATGGACCTTCTCCACTTCGTTTTACAGCAGGAGGCAGGACTGCAGTCTTTCCTGCACATCACAGTTTTC[A/G]CCTTCAGTTTGGAGCACAGTACCTGCACCGTGTCTTTGTTGACTGAGGAGTGAGGCTGAGGGACAAACAAGGAGGATTCACTTCTCCAGGAGGACAAGTG
1	98049934	SCAFFOLD285002_89217	0.48	0.39	GTTTCATAGTGTTTTCATGTTTTAAAAAATAGACCCCTGTGTAAAAGACAAAATTATGTTTACTGTCTGCTAGAATGCTTACTTCAAACTTGTCATAATA[A/G]CTTGTTCCCTTCGTTTCAATAATTCAGTACAACTTCAAAAATATGTGTTTTCTTCTTCTTACAGGTGGTTGATAGTAAAAGATTCCTTTTTACTGTATAT
1	99793772	SCAFFOLD160257_15528	0.27	0.40	TGTAATATTATTTATTACAAGAGTGGTTATTATAAACTGCATATGACTGTGAATCACTTACTGATTAAAAATAGCGAGTTCAAACAGATTCAGTCCATCA[A/G]AAGTCAGCAATGTAACATTCGTTATACAAGGGATTTCATGTTTCCGGCTATCAGAATTTTGTAACTCCATTGTACCAGTGGTTGATGTGGCACTTGATAA
1	100185634	SCAFFOLD280301_21836	0.26	0.19	TGAATACCAGAGATTTGCCTTAAAGGGCCAGGCAATCTGAAACTTTTCAACCAAAACAAGGCACCAGATTACTGAAGGCAGATGATATTTCAAGTTAAAC[A/G]TCAAGTATATTGTCCTTACCATCAGGGATTCTGGCAGGATTAGCAGTGAATAGTGCCTATTATTATATTGATGATAAACCATTTGCATGTTATTAACATT

1	100497613	BES10_Contig745_3284	0.27	0.41	TTTAAGAAGTTCAGTCAGAGAGGAAGACAGGGATGGGGGAGACGCATAAGTTCTTTCTAATTACCAAGTGGATTTAATTAGTAAACAGCAACAAGTCAAG[A/T]CTGGCATTGTTAGACCAAAGGGCGAATTGTCAAGGAGTTGGGCTTCCCAGGTGGCTCCATGGTAAAGAATCCACCAGCCAAGCAGGAGACTCGGGTTCAA
1	100876223	BES6_Contig246_498	0.45	0.46	GCTAGGCTTAATTAACACTTAAACATTAACCTTGAAGATATGTCCTGGGAGGGCCAGAGATGGGGCATAGAACTCCTTGAAAATGGGATGGAGTAATCTC[A/G]AAGAAATGCTGAAATTCTGAGAGAAGCAAAAGCTTTATCTTATGAGCCCCTTTAAGAATCTGCTCCTGCCAACCAGGAAAATTCTGGAAGTCACTACTGA
1	101001400	SCAFFOLD75339_7153	0.43	0.36	TTGTTTAGATAACACCTTCGGACACGAATGCAGTTTGAGCTGTGAGGACTGCATGAATGGAGGCCGATGCCAGGAAGGAAAGAGTGGGTGTTCTTGTCCC[A/G]ATGGCTGGGGAGGCATTCTTTGTAATGAAAGTAAGTAATACTTTGGAGCATGGGTGGGGGGTGATGAATTCTCCTTTTACTGCTGGTGAACTACTGGTGA
1	101604609	SCAFFOLD215053_5265	0.25	0.34	CCTTTGGGTTAAAATATAAACATAGTCCCACTTAAGAAATGAGAACCAAGGACAAGTCTTGGCATTTGTTGAGTCCTTCATAGAACAAGAAAACACATGA[A/G]ATTTTGCTATAATTCAAGAGCCATTCTTGTCAGTGCTTTCAAGGGTAGAAATTAAGCTATATTAAAGCTTGTCTTGGCCCAATTATAGAATGAAAGTGAG
1	106749725	SCAFFOLD202505_4254	0.10	0.09	ATATGAATGATAAAGCATAGATGGAAAGACTGGTTCATAAGGAAGTGCCTGGAGAATATAAAAATGGTGTGAATTCATTTCAGGTTTGGCGAGCTGCCTG[A/C]AACAGCATTGGAATCAGATACACGCAGATGGGGTTCAATTCCTGGGTCAGGAGTATCTCCTGGGCATAGCAACCCACTCCAGTATTCTTGCCTGGAGCCT
1	107399664	SCAFFOLD127873_5753	0.25	0.28	TAGTGCATTTATTTATGATATTCAAACTGATGAGTTAATAGAAGAAACATGTACTGCTAGTTAGTAGGAAATAACCATTTAGTCACTAATTTGCAATGAG[A/G]TCTTAGACAATTTGCAATTTCCTGGAACTCAGTTTTCTAAATTGTAGTGAAAGTGAATATATGTTTGCTAACATCCCTCCCAGACGTTTTACCCAAATTC
1	110232313	SCAFFOLD135366_9491	0.43	0.30	CTATCATAACATTGTTAAACAGCTATATCCCAATGCAAAATAAAAAGTTCAAAAACAACACAAAGATGCTGCCATTTGACGGCCTTTCTGATATTTGTCT[A/T]CTCCTAAATGGTTGAATGAGTACAATTAATTTTACAAGTCTTTTTGTAATCACAAATGATTTTTTTAATTATATAATTTTCAGGAGGAGAAATATTGGTG
1	110232669	SCAFFOLD135366_9135	0.29	0.41	CATGTCCATCACAAACTCTCTAACTATTCCTCACCGCACCCCCCATGTTTCCCCTTGGTAACCATAAATTCATTTTATAAATGGCAGCCTCTTAATCCAT[A/G]CTTGGATATCATAGGGGAAAAGATTGGGTTAATTTTTTCCTCAAATTATATTCCTTAGTTACCTAGAGCTTGAAAGTCCTGGGTGCCCATCTGCCAGGAG
1	110232754	SCAFFOLD135366_9050	0.30	0.41	AGCCTCTTAATCCATACTTGGATATCATAGGGGAAAAGATTGGGTTAATTTTTTCCTCAAATTATATTCCTTAGTTACCTAGAGCTTGAAAGTCCTGGGT[A/G]CCCATCTGCCAGGAGTGCTTTGGAGATTGTTTACAGAAGGGAAGCTGAAGGACGTGACCCCATGGTAGCTTCCAGTTCTGAGTCTGAATCTTAAAAGCAG
1	110567887	SCAFFOLD114762_1113	0.23	0.29	ACATAGTAACGTCATTCATCCCATTTGTGTTTTTATTTCCACTTTCTTAAAATGAGGATCTTGTCCTGTGTGTGCTGTTGTGATGAGCAAATGAGAGTAT[A/G]TGTGAGAACTTGGAAAGCAGTCAGGGAGTTTATACCTCCCTGTAAGGTGTTTGTTAATAGTCTGTAGCAAAAGGGAGAATTGTTGCCTTTGATCAAATTC
1	110568327	SCAFFOLD114762_1553	0.42	0.43	GTAGTAATCTTAGCTGTGGCTTTATCTTAGGTTGTTATTTTTCCTTGTAGTGTTTTTTTTTTATATAATTATCTGTTTTTTAATGATCCCTATTATACCT[A/G]TTTCTCTAAGCTACTCACATCTTTTTTTTTTTCAAGCAGGACAGATACAAATGAAAATTATGATGATTTTTTTTCTTTCTCTTCCAGGGAAGCACAGTAA

1	114871331	SCAFFOLD80515_13456	0.22	0.27	TAAAAAAAATTCAGATAGAATTTCTGGGAATTTTTCTTTAGGAAGAAAATAAGTTTGAAGATGGAGGGTTTGGGTTGGGGGATGGTCAATCAATGTGTTT[A/C]TTCTTTAGTTTGATTTTGTTGTTTCAAATAGAGAAAAACATTTTCAAACAGGATGGTATGACCAATATTTCAGTCCTCCCTAATTTCACCACAGCTGAGT
1	115637893	BES9_Contig289_730	0.21	0.08	TTCAGAGGAAGCAATGCCATTTGTTCAGTGACATTAACACCTGATTGACTTGGCCGACTGAAGAATGACTGGAGCAAGTCAACCTTGTTTAGTTTTCACC[A/G]TTGAAGAATGATGTCTCATCCCATTCATAGAGCTGAACGTGATTCCAGTAACAATAAGAGGCAGAGGAAAGGATTAATTACAGGTGGGGCAGTTTTTCTC
1	116820103	SCAFFOLD111007_1012	0.09	0.17	TTCTATGGGTCATCAATTTAGGCAAGTCAAACTAGGAACGATTTGTCATTCAGCTGGGCTGGCTAAAACAAGTGGGAATGATTACAGTCATATATCATAG[C/G]CCTCAGTTCTCTACTCAGCCTCTCTACATACAAGTTTGGACTTTCTCACTGGATAGTGGTGTCAGAGTAGTCACTTTGGTCAAACTCCAAGAAACTATAC
1	116820290	SCAFFOLD111007_1199	0.34	0.44	GATGACCCATAGAATCATGATCTTACAAAATGGTGGCTCTTTTATGCCACTCAGTTTTTGGAAGGTTTGTTGTGCAGCAGTACATCTTTAGAACAGTTTT[A/C]AATATCTGTGGATATTTTCTTAGTTCTACTTATACTACATTAGCTAATATGATCTCACATTACTTTTCTATCACCTAAACACTGAGGGTTCAGACCTCTA
1	118663240	SCAFFOLD119278_423	0.25	0.27	GGGACGCTTCAGGAGTCTTTCCTCCTAGTTTGATGATCAAACATGGGAGGGAACTACACAGCCATGAGGACGCAGTGGTGGTGGTAGGAGGTGTGATCAC[A/G]CCTGGACCATTGCTGTAACCATCTACCACATCCGGCAACCCCAGCATGGTGACTGCTCTGTCAGCCTGATTCCTGAGGGGCCTTGAGGATCACAGCCTCG
1	121159770	BES7_Contig244_858	0.37	0.32	ATTGTAGAAGTTGTAAATGCACCGCAGTAGGAAGCAGCTTTCCCTGGACTTCAAAGCAAACGGCAGTTAATTATCTAGCTTTCAGGAGCAGTGTGGACCT[A/C]CCGTGACAGCTGTTAGGCGAGGATACACGGGCATCTCCACCACTGAACATCAGCTCGTGTCATTGTTCAGCTTGGATCTCATTGGGAAGAAATGCTTAAG
1	121159979	BES7_Contig244_1067	0.16	0.16	CAAATGTTCACTGCCTCGACCGCGTCAGTTCCACTAACTCCTTTAGATGTTCAGGTTTCTCAAAGGTATTCAAATCACTGAAGCTGCCATGTGAGGTTTT[A/T]CAAGACAGATGTTCTAAAAAAGTGACTTATTTTTTAATCACTAGATTATTAAATAAGTTTGCCAAAAACATGACTATAAGTCATGGATGTTTGCAGCTTT
1	126527215	SCAFFOLD211611_4527	0.46	0.11	TAGTTTAACTCTTAACCCTCTACCATTATACCGTTAAGCTTTTAACTTGATCAAGTCCTTCTTTATAGTTACTGCTTGGCTTTTTGTCATTCTTTTTGTG[A/G]TTGCTTATAAATTCATTTTCTCACATTTTCCTGTGGGTTTTCCCTTTTTTGTGTGTGCACAAAAGCCTATCAATGCAAACAAAAGCTTTGTTTTATATAT
1	128420848	SCAFFOLD251209_4033	0.14	0.21	TTACTTTGCAATGAAATTTTTCTGTATAGAAATTAAAAACTTTTATGAAATAAATTTATTGAACTATTTCTTATTTAACTGCTAGGTCTCATGTCTTAGA[A/C]ACAAAAATGGGCGCTATATAATGCATTTTTAAGAAAACAATATTTATGAAATCCAACTTTGATCTAGATATTTAATAAACTAGTACAATTATTTTATCTT
1	130934215	SCAFFOLD192231_977	0.22	0.12	TGACCTTGAGCCTTAGGGAAACTCTAAACCCAGGCCACTGTGCTCTCCATGGCATTCAGGACAGATGCTAGTCAATTATGTCAGTCATGGCACTCCTTAT[A/T]ACTGAGCTCAGTTCTGCGTCAAAATCCTCCATACATTCCCCAGAGGACTCCACCAATTAAGCCTAACTGGCTTTTGAATGCATGAAAGTTATTTACCATC
1	130936902	SCAFFOLD71663_1538	0.35	0.48	GGTTTCTAGGGGGTAGGCCTCAGACATGATATTTTACTAGCACCTTAAGAGAGAGGCTCAGCTGCTTAAGATGCAGGTTCATTCTTCCTCTGCAAGCATC[A/G]TTGTCACGGCCAGCAAAGAATTATTCCTTGTCTACATGTATTGTTTATTTCCTCTTTCAATAAAGCCTCATGGAACACGGATATTCTTTCATTTCCATTT

1	130937339	SCAFFOLD71663_1975	0.32	0.45	AAAAGTTCTCTTAATTACAACAGAATTAAATAGAGCATTCTTCAGCCTTAACCTTGTTATTTTTCAAGGTGAGTTATTACTAACTCAATTCTAGGATGGC[A/G]TTCAGTAGCCTCTCTTTGTAGCAGCTGTCTGCAGAGCTATTTAGAAGGGATGTATCTCAGGCTAGATGCAGCTGGAGATGGATGGAGTATAACTAGATGA
1	131657378	SCAFFOLD181011_3284	0.35	0.39	GTTTGGACTCTGAGGCAGGCCAGAGAGACAGGTGGTTGAAAAGAGGGTGCCTCATGACACTCCATCCCACCTCCCCTGCCTTCCTAGAAGGCCAGCCTTG[A/G]CTGAGGCATCTGAGGCCAGTTGGATGCCCGACTCCCTGCCTGCTCTCTTCAGCAGCCTCAGCAGTCAGCATTGCCTGGGACTGGTTCTGAAAGGTGGGAG
1	132195905	SCAFFOLD130192_517	0.50	0.43	ATATATTATATATAAATTTTCACATATATAAGTTTCTGGGCTCCTAATTTTGTTTCGTTAATCTATCTGCCTAGTGTTTAAAAGCAAAATTCAACCAAGT[A/G]AATTTGAAGAATTACAATTGGCTTCATTGGGCACTTCATGATTTTGGTAGAACCTCATCTAGCAACTAAAAGAACCTCCAAGGATTTGTACAAAATGGAA
1	132959913	SCAFFOLD255974_14883	0.12	0.12	ATGTAATGACGTAGAGATCCAGGGTACCATCTTTCATGGTACACCTCAGAAGTGACACAGTCCAGACAGGCTACCTGTCTCCTTATTTAAAAAAGGGGGG[A/G]AAAAGGGCTTTCTTTGTTTCAGTTTTAGAGGCAGTAAATACCTTTGCCTTGGAGCTATCCATGGTGCTTGACCCATACTCAGTCTGTCTCTTCCAGCAAT
1	133882238	BES2_Contig431_1345	0.18	0.18	AGGAATTGAGCTTTGAGTGCAGACCCAAGAAAGTTAGTAAACTGCTTTATCTCCTGCTCACCTTTTTCCTGTTCAAAGAAGGGGGGCTGTTCCAGCCTCAC[A/G]TTGGTTGAGAGCAAGCAACCAAAGAAGGGGGCAGTGGGGAGGTCCCAGCAATGACTTCTCAGGCTCCCAGTGATGGACCCTATGTCTGTGGGCCAAAGAA
1	133909843	SCAFFOLD1494_2404	0.50	0.02	CTTAAATAAAGCTAACACTCACAAAAACAAAGGCTACATTCAAGGCCACCCTGACAAAAGTAATAAAAAGATGCAAGATTCCTAACTGTGACATTATTTG[A/G]AAGAGTAAAAGACTGGAAAAAATCCAAATACCTAGCAACAGAAATGACTGAATAAACTATGGTACCTCCATGCAGTGGAGTTTTATCCATTCACAAAAGG
1	136344020	SCAFFOLD40562_2432	0.32	0.24	TTGGATGAAATTGCTGCATCATGTAGTATGCATACAGTCAGTCTTAGGCTGCTTCTTTCTCAGCATAAATAGGCACTCTCAGCCCTCAGCACTCCAGGCT[A/G]GGTCTTCCTCTGGAGTGGTAGGTTCACTGACTTTTGCAAGGGATAAAGAACTTCTGATACAAAGTATAATGAGCTCATGGCATCCTCTTATAAGCTCCTG
1	137000997	SCAFFOLD241332_15465	0.28	0.32	GGGCCACAGAAGGGGAGGTTTTCATTATGTTGATGGGTCACTGAGGGAATGATCGAGCAGTCTGGGGACTCCGAGGCTCCGTGAGAATGTAGTTTAGGAG[A/G]GCTGTGTGAGTGAGGCCCCTCCAGGCTTAGGACAGCCCTCTTCTTGTTCCTGTTCTGCAGTGTCTGGATGGTTGACAGCTTTGTACCTTTTGCTCCATGG
1	137531653	SCAFFOLD141183_2832	0.23	0.28	GTTATGAGGAAGGGTGGAAGGATTGTACCTTAATGGGCTGGTAAGAGAAAGAGAGGTTTTATTGTGTTTTCTTTTATTGACTTTGTTCTGGTTTTAGGCA[A/T]GGAAGCAATAGAGACAAAGAAACTGGACATATGATTACTTGAAGAGAGGTCCAAGCAATGATAGGTGAATGGGCCCAGAAGTCCAGGTAAAGGTATAGTT
1	137926266	SCAFFOLD342104_590	0.47	0.35	TCTTCAGGGAGTCTTGCGAGTCCACAGCATCGAGGCAGCTGGCTTCTAGGAGCCAGGGCCACAGTCAGAGTGGAGGAGACTTTTCAGAGGGAATAAGGAC[A/G]TGGGAGTGTGTGATATTGTGGGTTTGCTTGGGCGTGGGCGAGTGTGACTGTGTGTCCTGTGTGTAGAGGAGTGTGTGTGGTGTGGATGTGGATGTGCAGC
1	139258387	SCAFFOLD46440_2872	0.48	0.44	TTCCCTCTTAACTCCAGCAGCCTTACACGTGTGCCATGCACCCCATTTCTGGCAGACCCTACTAGACCCTACTCTTTGCTCCCACAAGTCTCTGGAGAAA[A/C]CACTGCCCCATCCTAGCAGCCTGTAGCTTCCTGCCCCTAGAAACCAGACATGTCTGGAAGAGGCCAGAGATGTGAGGGAGAGAGCATCTCAGACTTCATT

1	139342300	SCAFFOLD314349_417	0.41	0.49	AAAACATTATTTGCCTTTGGAATAAGGTTTTAATGTTCCAAGATATAGTTATCTCATGAATTATACATTGCTTAACTTTAAAATTAGGTTCATTCCTTGA[A/G]TATATATCAGCCAATGAAGGGGAGAGGTGACTTACAGAAATCAGTAACTCCTACCAAAGGCATCTGCAAACCTGCTTGCTCTTGTTGGCAAAGATATATT
1	142495263	SCAFFOLD95149_21621	0.28	0.23	AGACTGGATGGCCACCGCTGCTCGTTCTGTCTCTGGCGCGTCCATGTCGATGTCGAATTCCTCTTGAACCTTCTTCTGCCCATCTGGAGCAAGAAAAATA[C/G]AGACAGAAGGTGATCACTGAGCAGTGAGCAGGAAGACAGCGGCTCTGGATCAAGCCAGGCTCCAGGCGCCACGTAGCACCTCCCCCAGCACAAGACCTAA
1	142495435	SCAFFOLD95149_21449	0.46	0.47	AATGGGGCCCCAAGGAACAGAGACTGGTACAGAAAGTATCCACGACGCTGAGACTGTGTTTCTGACTTTCCTCATTCCTCCCTGGCAGGGGTAGGACTAG[A/G]GAGTCAGAAGATGGCTGCTTCTGAGCTCCAAGGAAGCCCAGGACATGCTCCACTGGCTTGTACTTTGCAAATTAGGTCTTGTGCTGGGGGAGGTGCTACG
1	142882661	SCAFFOLD51241_2618	0.08	0.09	CTTTCTAGTCCCATTGTTTTGCTCTGGGACAACTTGGAGAACTTTCTCCTTCCCTGTAGAAACTACCAAAAGAGGGCAGTAGGGAGCCATTGCCCTGCT[A/G]TCTTTAATATCCTCTTTGGTTCAACAAGCCCAGAAACGAGTTTCCTCTGCAAGGACACACGTTAGTGTCAATATGCTTTCACAGAAACACAGCTCTCTTG
1	144614382	SCAFFOLD240276_5836	0.27	0.34	GACCACACACCTGCAGACCACAGTCCTGGGGGGACCCTTGCCACAGGTGTGTCCTGTCTAGGGGACCATGTGACAGTGTGGCCTGGGGACTCACGGGGTG[A/G]CCAGGATCAGTGAGAGCCCCAGTTTCCTCCAGCATGCAGGGGGCTGTGTCACAGCCATCCATGGCTGATGCGAGTCCCCAGGGTGGTGTCAAGAGTTGCC
1	144626794	SCAFFOLD134704_1116	0.35	0.50	ACCTGTTAAGGATTTCCAACAGGGAGGCCCCTAGAGCTTCCCTGGTGATCCAGTGGTTAAAAATCCACCTGCCAATGCAAAGAACATAAGTTTGATCCCT[A/G]GTCTGGGAATAGCCCATGTGCCACAGCTACCAAAGCCCATATGTCCTAAAGCCAATGCTCTGCAATAAGAAAAGCCATTGCCAAGAGAAGCCTGCCCACT
1	145384804	SCAFFOLD100330_4459	0.22	0.14	TTGAGGGCCACCTGCAGGCCCCTCATCAGAAGCCCTGAGGGCAGGCACTAGCAGCTGCCCTAATCCCATGACCCTTCTTTAATAGGACGTCCCAGCCCAC[A/G]TCTCCAAAGCTTACCAGGAACAGCAATGTCACCCACAAGTTGTCATGTGCCATCTATCTCTACAAGGGTCAAATCAGGAGCTGCTACAGCTGCTGACCTT
1	146398375	SCAFFOLD256239_3392	0.36	0.43	CAGCAGAGGTTACATTTTTGTGACACAGGACCCAATAAGCAAAGTAAAACGTAGGTGACAGATGGGATGATTGTCAACACTAAGGACGCCTCAGCCTCAC[A/C]GAAACCCCGGAAAATGTCAGCAGAGAGGAGCTGCACGACCACCCGAAGCTTCAACACGAGTCGTGGCCAATGGCCAGGTGGCCCTGGGGCTTCCAGCCAT
1	146398651	SCAFFOLD256239_3668	0.28	0.34	AACGGAACAGAATCAAGGATGCAGAATAAGCTTCCTGCATCTGTGGGAATTGTGTACGTGGGGGAAAGGACATCTCAAATAAGGAGCGAGGGTGGAGCCT[A/G]GAATAATCATATATGGAGTCAGACCACTCCTTACTTCACTCAAAAAATATAAAAAACACATGACGTGACCAAACAGAGGGAACTGGCTAGAGCTTGCTTG
1	146546805	SCAFFOLD125137_2487	0.12	0.11	TTAGGGAGCACTGTCACTAACGATGGAGCCCAGCACAGCTGAACTGAGAATGACCCGGAAACGACACGGCCATGCGTGGTGCCCCGAGTGCCCAGGTCCC[A/G]TGTCCTCGGGTAGCGCAGTGCTGTAGAACTACTACTGACGGAAAGGCAACCCATTCACACCCAAGCTGGGCCAGGGCCAGCCATACCTGCTGCACTGTGA
1	148863139	SCAFFOLD140011_29325	0.39	0.46	TTTGTGGAACAGAATTGTGAAACACAGCAACCTTACTTTGTGCTCTGAAATTTTACTGGCAAGGGTAGGCAGTTTCCTCCTACTCCCACTTTGATGCCTT[A/C]CTTGCATCTGTCTATTAATAGTTTCAATTTGCGAGCCCTCCTTACACTGAGGTAACATGACCTCTTTACTGCCTGTGTGTTCTGGGCAGGGGTTACCTTT

1	150099856	AF440368-538	0.02	0.03	TGACCATGTCAACCTCCAAAACCCTCAAGGCAACAASAGTCAGAAACAGGGGCACAGTTCCCYGGGCTCCCAGACTGAGACCTGGAAATTAAAAGAAGCA[A/G]GTCATAAAGGGAGATGCTTTTTCACCACTCCACACCTGCAATCAGTTCCCTCCTCAATATGAGACCTGGGTTGCCTCAGGGCACAGCAGGCTGGAGGGGA
1	152127697	BES5_Contig610_5337	0.15	0.22	CTTTCATCCCAGCCTTCCCACTCCCGGGAGCCTAAGAGTTTGTAGTCAGCCCAGTGGCTGCAAGCACCTCCTTCTCCCTGCTGGCAGACAGATGTTTTCC[A/G]GCCTCCATGCCCATCAGCCGGAGCACCCTGATTTATAACCCTGAACCCAGCGAACAGGGCCCGCAGAGGCCAGACTATCACCTGGCCTTTGGAATGAATC
1	153015009	SCAFFOLD197372_885	0.42	0.48	GCCTATATTTGCCGAGCCTGAGTTTCTGTTGTCTGCATCCAAGAACCGTCACACATAAAGGACCCGGAGGAACAATCGCCCAACATTGTAGGGGGCGGGG[A/G]GTGCGTATCATGGGAGCAGTAATTCAGCAGTATCATGGAGAAAAGGAAAGATGAGAGAAAAAATTACCTAAAACTAAATCAAGGTCTTGCTGGTGACGTT
1	153019152	SCAFFOLD289544_1115	0.19	0.14	GAGCCATGAAGTGAGCAGATGCTATTGGAAAAATGACGCTGATAGACTCGCTTGACTGGGGTTGCACAAGCCTTCAGTTAGTGAGACCCACAGTATGCGC[A/G]AGGCGCAGGGAAACGAGGCACGCTCGTGGTCCTGGTCGGACTGGGAGACGCAAGCCCTCCTTGGCGTGGGGACTGTCGTGTGTGCTTGACTGAGGTCTGC
1	153517286	SCAFFOLD322842_13209	0.23	0.32	AGTGACAAAGTAAAATCTGTATGCTCCCCTCTGCCCCCCAACCAAAGCCACTCCTTGAGATCTAAGACTCAAGGTCCACGCTGATGCCTGGTCTCTATTT[A/T]CTCCATGGCGTGGCCCACCATACCCCTGCCTGCCCAAACCTCACTGCCCATACTTTCTGCATGCAGACTGATTTCTCATTTGAAAAGCCTTTCCTTTCCT
1	155436054	AJ506786-077.T7-196	0.35	0.42	AGAAAGCGTTTCAAGTTTAACTGGGGAAGACATACCTCCRTAGTAGYTGAACTCATGGTGCATATTCACCCAAGGTTAAATGCTTTAGTGAGGACACAAA[A/G]AGGCCAGTATTGTACGGTTTAGTATGAGCTATCAGAAGAACTTACAGGTTAAAGTTTTAGGCTAAGCTTTGGGAGTCTGCAAACACTATTCATCCCAATG
1	155436054	AJ506786-077.T7-35	0.32	0.42	AGAAAGCGTTTCAAGTTTAACTGGGGAAGACATACCTCCRTAGTAGYTGAACTCATGGTGCATATTCACCCAAGGTTAAATGCTTTAGTGAGGACACAAA[A/G]AGGCCAGTATTGTACGGTTTAGTATGAGCTATCAGAAGAACTTACAGGTTAAAGTTTTAGGCTAAGCTTTGGGAGTCTGCAAACACTATTCATCCCAATG
1	155870122	SCAFFOLD265688_10197	0.30	0.34	CATGCTCAGTAATAATTGTCAACAATGTTTCAAAGAAAAGAAAAAAAAAACTTATTTTCATAATTTTAAGCTTATTTTGCCGAGTAAAGCCCCGATAAAA[A/C]CCCCCTTTTCATTATTTAATGTAGAATTTTGCTTTCCTTTATAAGTGGGTTAATTTAAGTGGCCCTTTCCTATACCAATTGTCTTGGAACAATGAAGCTC
1	157243785	SCAFFOLD146464_5603	0.21	0.21	TTCATTTCCTCACTTGAGCTAGCCACATTTCAGGGCTCAATAACCACATGTGGCTACTGATTACTACACCGGGCAGTGAAGAGATTATAGAACATCCATC[A/G]CAGTTCTGTTTATCCTTGATAACTGGCTTAAATCGGTATTAAATAAATCCAAGATAGAAAGTCAGAGAGATGGGGACACCATATTGAAAAACAGGGTGGG
1	157424324	SCAFFOLD303294_5477	0.24	0.35	TTTCCTCCAAGAACAAACATTAAACCATTTTAACTATTAGAAAAATTACATCTCCTTATGCCCACAGGATATCAGAGCTTAGAAGGAAGATAAAAAAATC[A/C]CAAGTAGCAGGGAACCATATGGAGAGAGTCACAGAGCTTGTGATTCTAATGGTAGCTGCAAACGAGGTCACTTATCCCTCCACGGCCACACTAATTGGTC
1	157424517	SCAFFOLD303294_5284	0.24	0.35	GGAGGAAATACAGTGAGTTGCTTAGGCTTTTATTTCTATTAATCCATGCTAATAGATTATATGAAATATAAATAGAATTATTTGGAGGTTTTCATATTGG[A/G]GCTGATAGAATTCCTGATATAATATGGGTTTAAAGGATGTGTTTGTCTTTGGGGTTTTTGGTGGTTTGTTGAAAGAGATTTACAGGTTCTTTCAAGGCTT

1	159650512	SCAFFOLD2197_3113	0.37	0.37	CTACCATCTAAGCAAGAAACTCATGATCTTTACAGCTTTGGATTCTTCCCAGGTCCTAGGCACTGGCCCACAATCAAATAAAAATCACTGAACCAAAAGA[A/G]ATGCTTATGGAACAATTTCTTTGTATATACATAGGCTCAATATGTAGACGGGCTGCAAATGGCCAGCAGATAGTTTCTTTATCTGGAAAAAGTTCTAAAA
2	1018044	SCAFFOLD120200_997	0.15	0.29	ATAATCTTTCCCTTGTCTGTCTCCCCACTGAAGTCTAAAAGCTATTACCATTTTATCCAAACAGCTGATCATACTGCTTAGTGCACAGAATGTATGTTCA[A/G]TAAAACCTGTTTGCGTGGATGGATAAGGTAATTGAGGGGCAGGTAGATAGGAAATTAAGTTGCTTTTGTTAAAGGCCAGTGTCCTGTAACCTGAAGAATG
2	1233102	BES9_Contig351_698	0.22	0.33	CTTGTTCTTCTAACGGTGTCCTGTCCACCTGAATCCCAGAGTCCTCTCTGCTTAAGGACTGTTGATCATTTTCATCTTCACTTTCTTCAGACCAATTCTG[A/G]TTAAAAGAAGGAAATACACCTTAACCTTAGAGTACCTAACAAATGGACCTATTTAGATGAAAAGATTATGAAAATTTCAGAAGCAAAAACTATATCAAAT
2	1402724	SCAFFOLD150185_12582	0.42	0.42	GTGAAAAAAGTGAAAACACAACACTTCCTAACACTATACAAAGAATAAACTTAAAATGAATTAAAGATCTAAATATGAAGGCAGAAACCTCAAAACTCTT[A/G]GAGCAAAATATAAACAGAGCACTCTCTGACATAAATCACAGCAAAATCCTCATTGACCCATCTCCAAGAGTAATGAAAAGAAAAAAATAAACAAGTTATT
2	1926271	SCAFFOLD141047_7331	0.31	0.38	ATCCTGAGACTAAAAAGTCTGACTACCACTGGTGTATATACTATCTGTTTTCATTAAAAAAAAAAGGCAGGAAAATAAGAATTTATCTTTGTACTAGTTT[A/C]TATATGCATGAAGAAAAACTCACAAAGGATAAATAAACCAGTATGAGTAGTTAAGCAAAGGGAAGGTAGGAAATGGGCAGACAGAAGACCCTTTAGTGAA
2	3921002	SCAFFOLD100952_2333	0.45	0.43	TCACTCACCCCATGACAGTGAGCCTTAGGGAGACTCAGGGTGGAGGAAACCAGAGAACACTCTGTGCTTCCGGTGAATGGGGCTGCCTGCTGTTAGATGC[A/G]TATCTCAGGAAGAATTTCGATGACTCCAGATCTTACATCTTCCTGTACACAGAAAAGCATGAAAAATTATGAACTTGAGATGTCTGTTCTTTGTGATTAG
2	4527399	BES11_Contig456_1216	0.27	0.47	TCAACTACTTTAGGGGCTAAGGAGGTCATCTGTCACTACCTGGGGAGTGGCAACACAGTAGTCTTATTCATTAGACTGTGTTCTCAGTAAAGTACATATA[A/G]CTTTCTACAGCAAAGGATAATTCTCCAAATTTTATGCTCAACTGTATATTATGCTTAAGAGTAAACAATAAATTTATAACAAAACTGACGGGGTTCTTTT
2	5178695	SCAFFOLD135503_8926	0.37	0.47	CCACAGGTTAGAGATGCTGCTTGGTCTGACTCTTGCCGTTAAGTGGCACTGTTTGTTAACCGACTGACTGACCTGAGGGGCTTTGCAAGTTGATAGAACT[A/G]CATGTCTAAGCATCATTTATCCTGCTGCTACTGCTGCTAAGTCACTTCAGTCGTGTCCGACTCTGTGCGACCCCATAGACGGCAGCCCACCAGGCTCCCC
2	5551972	SCAFFOLD265863_5396	0.42	0.40	GGGAAGCCCCTGCTGAATCATGCTCTCTTCTAAATCAGGCTTTGAAAGCAAAGAGCAGCTGGTTTCCTATACCTGTGCCATTGGGCCCCTCCTTCCCCTC[A/G]CCCCCTCTCCTAGGGTCATAGGGCACATCCTGGGCTGTCTGCATATCTTCTCCCCTACATCCCCTCCACCTGGCCTCTAATGTCCACCCACCTTTCTAAG
2	5552633	SCAFFOLD265863_6057	0.41	0.39	GCCCACAACGGACTGGTGTGCAGGTACACGCTTGCTCTGCCCGGGGGACAACAGTGAGCCCGGCGCCTGCCCTTCCCCCGGTGTCTAGTCCTCGCTATCA[A/G]CTAGATGATAATTCCATGTTAGAGTCGAAAAAGCGGTTCTAAGAGAGTAACCACTGATGCTAACAGCTATTATTTATGGAGGGTTAATCATATTTCACAA
2	5735772	SCAFFOLD230069_22382	0.20	0.24	CCAGCTGTAAATAAAGGAGTTCAGTTCAGTTCAGTTCAGTCGCTCAGTCGAGGGAAGTGTAAAAACTTCACCGTGCATAGATGCCTGGGGTGACCTGCCG[A/C]ATGGAACACTGACATACAGGATCAACTGTATATACAATATTTAATATTGACGGAAGATCCTAAGAGCAGAGAGTCACTTTTAGTGGCAGCTGAATTATAA

2	9810425	SCAFFOLD101947_5422	0.21	0.13	CTAGAATATATATACACCATATTCATAACATACCTATGCAAAGATAAAACAAAGAGATTTAGGGAGATGTTTCTAATTAACACAATGCCAACTTTTAAAT[A/G]CATTTTAAACACCCAAATTCACTGTGGGAATTTTCAGGAAGAATTACATGCTCCGTGCTATTTTCAGAATATGAAGTTTAAGCTAGTACATCTGTTAAAT
2	12801683	BES3_Contig475_1491	0.48	0.45	TCAAATCTGTATGTGGCTGCTTGATAAATCTGGCAAGTCAGTGTTTAGTTGGAACCACATATGGAGCAACCTTATGCCAGCCTACATTTTGCCAACAGAC[A/G]TAACAGTTCTGGGTCATTATATTGCCAACTACTCTGACACATTTTTGCCTACATAGGGCCATGGCTAAGACCTGAACACAGCTGAAAGCCCCAAGATATT
2	14869772	SCAFFOLD130639_5676	0.39	0.41	ACAAAGTAGTCCTAATGATGCTAATATGCTGCCAGGGTTGAAAACAACTGAGTCATGTCTTGACAATAAATGAGTGAGTGAATGAATGAGTAACAAAAAA[A/G]CACATACTCACTGTGTGAACTGTTCCTTAAGCTTTTCGAGGGTTAAGTGGCTTTTGTGAATGCCACCTCTTAGAACTTTATAATTCTGGTCTTCCAAAGG
2	14916340	SCAFFOLD150772_2535	0.43	0.46	TATCCAACTGGTGATTTGTGGGACGAAATTTTTCCTGGAAAGATTTCCTTTTAAATTTTAAAAGATAAAAATTTATCACCAGTTTTTAGACCATTTTCTA[A/C]GAAGTCAAAAGAGAAAACGATTGGCTACAATGCTAATGCTGTTAACACGCATGCATGCTATACATACATGGAGTATGTGTGTATTTCTAACTTAAACGAT
2	15277299	SCAFFOLD85870_4758	0.24	0.21	CCAGGTTGGCTGGTCAGACACTGAGTAATGCCAAGTTGGCTTGACTCCTCTGAAATGTATCCAAAATACAATCTCAAATGCTAAACACTGCAGAACCCCC[A/C]AAAAGTCATATGCTATAAAGTATGGGCTTGGTCCACTTAAAAAGGTTATTATTTTCCCGAGAGAGACTATGGTGTCTGTAAGTGGTGGCATGAAAGCAAA
2	15312400	SCAFFOLD10204_22524	0.04	0.10	CACAGTATAGGAATCTGCTGTTCTCCTGTGGAAGAAAGAATGAAGGCATTGCAATCGCCTGGCACATACTACCTCGAGTACGTTGAAGCCCTTAGCTGGT[A/G]GTATGAGAAAAAGTACAAGAAATATTTATTGATGACCTACAAGCTAGGTGATAGAAGAGGATGGGCATAGCTGATACAGATTATGGATACAAAGAACTTA
2	16494665	SCAFFOLD14255_629	0.34	0.28	CTCATTCTCAAGGCTCTGACCTTTAAAGGCATAACACTTTTCCATTCACACAGAGATTAAAAAAATTGCGGAACAGAGAAAAACTTTTGTCGTGTTGGAC[A/G]TTTACAGGAACATCTGTGACCTGACCTGACCTGTTGTAAGAACAAAAGCTTCTGACACCAAAAATTGTGCAACAACCAACCTCACCTTTTCCCTTTGATT
2	16632148	SCAFFOLD145911_8568	0.48	0.05	TCTTGAAAACTCATAGGGGAGTGTCAGTAGGGTTTGTTTGAAAGCAGATGCTGAGACAGAGTTAGGAGCAGAAAAGAGTTATTAGAAGGAAGAGCTCTGG[A/G]TGATAAAAAGAGGAGGAGGAAGAGGACTGGGCAGAGAGAACTTTGAAGATTTTGCCCAGATATGGCAAAATCTTGGTCAGCACACTAGGGCACTGCAGAG
2	18824550	BES3_Contig310_1112	0.47	0.42	TCTGGCCCACTCTGACGGTGATGACATCACGGCAGGTTACATCAAGTTCTACTTCTGGAGGATGAAGGATGTCCTTTGCAATTACAGATTCTGCTAGTTC[A/G]CGTGGCTCTCCCTCTCCCACAATGTTAGCTGCCTTTATACGGAATCTGTACTCATTTCCTTCTATGAGTCCAGGTACCCTAAAGGCACACTGCCTAATGA
2	19253514	SCAFFOLD304919_960	0.40	0.48	AAGGGAGTTGGTTAGGATCAGGGTGCAGATGAGAGTTCAGGGGGAACAGACATCAAAGAAGGTGGTGGCAGAAGCCTGAGGCTGGTAGAACACAGAAAGA[A/G]AGATGGTAGTCATGGCAGCCTAACTTCATAAGATGCAGCTGACATGATAAAGTGGAAAGTCATTTTATAGGCCACAGGCTTGAGGTCCTCTGCTACCCTT
2	19856255	SCAFFOLD45422_13306	0.02	0.03	TACTGGCCACATTGAAACACAATGGAGAGATGGAGATCCTGAGAGCTCCGACTCAAGAAACCCAGACATGGTGGAAGGATGATGTCCTGCTGAGCCAGTG[A/G]CCCCAAAGGTGGCAGCTCCAAGGGGAAGGCAGGCTGGAGTGGGGACGGGTACTGAAAGGAAACAGCTAAGCCAGGAGCAGATGGCATTTATATTAGAATG

2	20235089	SCAFFOLD115993_2831	0.19	0.17	GTAAGATTTAATTCTACATAGTTGATGCATATAAAAACATTCTAAGGGGACCGCTACCATCACCAAACTGCCAAAGGGAGCATGGCATATAAAGAAACCC[A/G]TAATAACTTGATCATGGTCACAAAACTCTTTGAAAACTCCTGATTGTATATTTTCCAATACTTAATGAATAAAGGGTGTTAAGACAACTGAGGTTAAATG
2	20710301	SCAFFOLD220690_10370	0.11	0.13	AGAGATGTTGAGGCTGGATTGTCTCTGTTCACAGGATTGCCTTGCCCTTACAAATATTTATATATGTGTGTCTAAGTAGAAAGATCTCATAAAAGAAAAC[A/G]AGTAAATCAACCTCCAAACTTTCTTGTATGTAAAGGAGGAAAGGCTGGCTGCAAGACAGACTTCCCGACCTGCAAAAGCTGTGATCTCATTTCTGTGTAG
2	21841024	SCAFFOLD105099_16237	0.44	0.43	CTAGTTCATTTTATAAAGCCATTTTGGCTGTTGGCTAGTTTCATTAAAACAGGGCTCATAATTATAGGATGATTAGCTAAAATGTCAGTGTTACTATGAT[A/G]TCTTTCCTGGGGGAAGGACAACAGCTAAGTGCTGGCAGCCCCAGCCTTGCTGCTGTTGCAGTACATGTTCTACTAGAGCATGCCAGAGTCCAGCTGCCCT
2	25285472	SCAFFOLD245835_3838	0.13	0.16	CTCCGCGGAATAGTGAGGAAGGTAATCGGCCTTCAAATCTGCCTGGATTTAATTCCCAGCCGTCCTTTTGGTTACAAAAGACCCCTAGATGGAGAAGGGT[A/G]AGCAGGTTTAGAAAGCCTTGGGAGGTGGGGCTCACCACTGGGGCGCAGCTTGCTGCTTTGGGGGCTCCAGTCACAAACTTAACCTCTTGATAGGTAATGA
2	25316610	SCAFFOLD125747_10620	0.29	0.37	AATGAATCTGCATGTACAGGCTGCTTTGAAAACCAGATCAGTCCCAACAACTGCACTCACAAAACTATGTGAAAAATAGCATAGACAATGAACTTTTCCC[A/G]TTACTCTAATGCATTTCATTATTTAGACGGTTTGTGTCTGCCTCTGAGGAGGAAACTGGCCCCGAGTCTAACAAATATTTGTTCTGATGTGTTAAGGCAG
2	26095592	SCAFFOLD121477_6039	0.40	0.44	CAGGATGTAGCTCAGGATAAAGGACACTTCAAGTAGGCATCTCTGGCCAGCTTCAGTTCTGACTCAGGAGAACGGCACTGTCCTTACAATTTCTTCCTGC[A/G]ATGGACATGGAGGTAAATTTGGAAGGCCTTAGGGAGTGCTTCTTGTTAGAGGATTCATGAGGGTCCCAAGGGCAGAACTAGGTTTGATGGAGCTTGAAAA
2	27273586	SCAFFOLD52046_3051	0.39	0.45	CTTTTAGATTATTTAATACTGTAAGTAATCTTAAATCCAATTTGAAACTATGGCACACATGTTTATCTTCTCTCTTGCATCTAGAAGTGTGAAGAATTAT[A/G]GTATCGTCAGCATTTCTAAAACTATTATGTTATTTGCATTGTGAGCAAATGCTTATAGTTCCTCACTTCTACATTTTAAGCAGAATATACAGTCTTTTGT
2	27788117	G73155-69-R	0.22	0.40	AAAACTAGTAAGTACATAAGTACATATCTACTGGCCTTTGATCTGACTAGTTCCCCAGTCTCAGGTCT[A/G]TTTGCTGTTAATCACCAGTGAGAGAAGGTCCTACCCTATCTTAAGTGGTTCTCATGTCTCCCTCTGGGCCATCCATACTTAAAAGCCCTGGATGTTCCTA
2	29419268	SCAFFOLD20097_26522	0.17	0.33	CTTTGTTCACCTAGGCGATATTAATTTCTCTGAACTGGAATGCATTTATCCTTTCAGGCCAATCTTATCTTAGAGAGAAGCCACTAATTGTGAAATTAAA[A/G]GGTGGTTACTATTCTCCACCAGTGGCAAAACCCACACTTTATTAAAGAACAAACAGAATATTGCTTTATGGGAAATTTACAATAAACCTACCCAAATGGT
2	29419608	SCAFFOLD20097_26182	0.17	0.33	GCGTTTAAATATACACATAAAGGTTAAAAACATTGTTCTCTGTATTTTAAAAATGCCTACAATAACTCATCTTAAAATGTCTACCGAACATAATACCTAT[A/T]ATGCTGTTCTTTGTTTCCACTCTTTATCTTGAAAATGCTTCCATTTTCAAGTTAAACTCTCACTGCATCTTTAAAAATCTTTTTTACAATGTAAAAGTTC
2	29654789	SCAFFOLD310570_1642	0.18	0.10	GTGGAGAAAAGCTATCCTTCTGTCCTGATGCTCTCGCTCTGCAGGCTGCTGATAATCTGTGGCAGAGAGGGACCCCTGCACTGTCACCAGGAACACACTT[A/G]TCCAGGAGGAAGCACACAGGATTTAACGGTTATTAACAAACATTAACGTAACGTAGACCTAAGAAATTATTCCTGTCAATCTGATTCGTAGGGCCCACTT

2	31106089	SCAFFOLD120383_1140	0.15	0.24	GTACAGAGATTATTTCAGAACTGTGATGTATTTCAGAACTGTGATGAAAGCAAAGTGTACACAAGAGGCACAGATGAGAGATGAGGCAGAGAAATTGGTC[A/C]GATTATAGAAATCTTTTCCCCCACCTTAAGCAGTTTGGTCTATATCCTATATTCTGATGACGGGTAAGTGAAGGGTTGTAGGTAGAGAACTACATAGGAA
2	31773763	SCAFFOLD324504_3277	0.21	0.30	TGCCTCTAAACTAGACACTTTAGATCTAAAGTAAAGGACAAACGTACAGTAAAATGATATATCAGAAGCTTTGGGTGTGGGAGGCAGAGCAAGAGAGGGC[A/G]TATCTAAAGAAAAAAATCAAAACCTCTTGCATATTAATCAATTAATTTGGGATATGAGATTACAAATATGGGAATTTTCATTTTGTATTTCCAACTACAT
2	32230599	BES6_Contig218_1114	0.42	0.43	CGCAGCAAATAAAATTGATCATATTTTTACATTATTTATATTTAAACAAGTTTTGGACATATTTTTAGCAAAATACGAAGTATATGTTTTGACAGTAGGC[A/C]GTATGCAGTGTGTTTCCTTGAATCTAGAGAGACACCATATGACACTCAAATTGAATTAAAAATGGAAAAGGAAAAGTGCTTTTAAATAACTGTAAGCTAT
2	32922777	SCAFFOLD150570_5075	0.24	0.31	TAACTTATTTCAGTGTGTGGAATGATTAACTTTATAATACCACTGTTTCTCCATCAGTAATTGCATAAATAGTACCTTTCTTATGCAGATTTTGTACAGG[A/G]GTCTTAATGAACTATATCAATTGGTGAGCTATAAGATCTAATGCAAAAAGTAAATCTGGAAAAAATCAATCTTCAAATTGTGATAACCAAGCTTTGTTGA
2	35431544	BES1_Contig605_744	0.27	0.24	ACATTTCAGATAAGACTTTTCTTACCATCCATTTTTATAAAATTTCCTAAAGTAAATTCTTGAAAGATTCACATTTTGAACTGAACCCTTACATATAATC[A/G]TGAACATTTCTTTCAGAGTTTCACTTTGCTTTTACTTCAGGCTTTGAAATAACCATAAAGTGAACCTTTAATTTCATTTCTAGAACATTCTAGGTTGAAG
2	35431781	BES1_Contig605_981	0.39	0.34	GAAACCCACTCATCACATAAAGGAGAGTGGGCTTTATCCATGACTCTGTAACATGTTTCTCTTCTTAGTTTTCTGTTTCTGTGATCATTATACTCTATTC[A/C]TCCTTTAAATGTTGGTGTTTTTCTGGATCTGGTCTTGATTCTCATTGTTCTTCACTTTCTTGGGGAAATCCACATGGTTTCAAAGACGAGGCCAACTGCC
2	35627523	BES11_Contig473_2661	0.04	0.04	GCTTTGTAAAGTCCAAGCAGATAATAAAATCAAAAATACAATTTCCCCCTGATTATGTTGTACAACAATGGGAGCAGCTTAATTTAATGTGAGTCAAAGG[A/C]AATAAAGCAGTTCTGGCACTTCCACTAATTAATCATCATCCTTCATCTTGCCTTCCATTTAAAATGTCTTCAAAACCATGGTCTTTGTAACCAAATTATT
2	37054932	BES2_Contig411_906	0.32	0.30	CCAGCTTCCAAGGAGGCTATTGGAGAGTCATTTCAATGTCAGGGCAGTGGGAACCCATGCTCCCTTCAACAATAGCTGAATCTCTTCATTGTTCATGGAG[A/G]TTTATTGGCAGGTATCACTGTCTGATAGTTCCCAGATTCTCCAGTATTTATTGCTGAGATGCCAGCATTTCAAATTATTTCCCAGAGCCCAAAGCACTCT
2	37055124	BES2_Contig411_1098	0.30	0.28	AGAATGATTTGTGATGCAAGGAATATGCTTTGACAGCCCAAACCCAAAACACATTCAAAAATTTCCACAGGGATTTTAATGGTGACCTTGATGAGGAAGA[A/G]AAAGCAATCTTTACCTGCTAGAGATATGAGAAACACTGCTTAGAAAGATACACAGACTAAATTTGATATGAAAAGAAATCTGGATTTTACCAGAGTGCTT
2	38768975	SCAFFOLD36022_3810	0.41	0.17	TCCAAGACATTCTCTAAGATCATTCTGGTTTCTTTTCAGATCTGGAGTTCACATCAAACTTCTTACTTCCTGTGATTCTGTGATGGCCATGGTGGGCCTC[A/G]GTTAATGTGGGGTCCTCACTGCCTAGTGTCCCTTTGAGCAGCCTCTCTTCCCCTGCAATTCACCTTCTGTTTCGGGACAGAGAGAATGGCACCGTCTTCC
2	40182188	BES10_Contig758_1293	0.46	0.31	CTACGATAGGATAATCTGTTAGCAGACAACAGGTCAGACTCAACATTTATTTATTTTATACTTTCTCATTATACTGTCCTTGAGTCATACAGAAAGGGAT[A/T]GCAAATGAAGTTGAAGAAGAAGGTAATTACCCGTAATAAGGAGGCCTATTAGACTTTTCCCCACAAGGAAGATTGCACTTTTCTTGTGTCTCCTCCCACC

2	40439143	SCAFFOLD296936_9333	0.20	0.35	TCTTGCTTAGTTACCCTGGAACAGGAGGGGACACAAATGGTGCCCCTGGCCACAGAAACCACCCCACTTGCCAGCACAGGACGCTTCCCTGAGTGTAGAC[C/G]GAGCTCTACACAGCTCTTCAGAAGTCTGAAAAGTTCAAATTGTGTTATACTGTTAACCCACCTTCTCTTTAGACCTGCCCAGAATGTCAGAGGGAGCAAG
2	40439209	SCAFFOLD296936_9399	0.20	0.35	GTATTATGAAATCTGACAACTCTGTGAGCACCCCAACTTGCATCCTACAATGCTCCTAGTTAGAGCTCTTGCTTAGTTACCCTGGAACAGGAGGGGACAC[A/G]AATGGTGCCCCTGGCCACAGAAACCACCCCACTTGCCAGCACAGGACGCTTCCCTGAGTGTAGACGGAGCTCTACACAGCTCTTCAGAAGTCTGAAAAGT
2	40439268	SCAFFOLD296936_9458	0.19	0.35	CCTACTTTCTTGTAGGAAAAATTAAGCATGTAAAAGTGCAAACCAAGCCACTGAGCACTGTATTATGAAATCTGACAACTCTGTGAGCACCCCAACTTGC[A/G]TCCTACAATGCTCCTAGTTAGAGCTCTTGCTTAGTTACCCTGGAACAGGAGGGGACACAAATGGTGCCCCTGGCCACAGAAACCACCCCACTTGCCAGCA
2	41323561	SCAFFOLD290052_5889	0.11	0.10	CTCTGGCAAAACAGCGTGTCTGTTGATTTTCCTGGCCTTTACTCCTAGAAAGACCAAGACAAGCAAAGTCCTCAACCTCACAGATCCCGGAAGCAGCAGG[A/C]GAGATATCTCCGAGGCTCCCCCGCACACCCCGGTGCGCTGCGGGGAGTTGGAAACGGACTCGGTAACTGTGAAGCAGAAAAAGCGCTAGCCACTTTTCAG
2	41325780	SCAFFOLD290052_3670	0.07	0.09	GGGAACGCACATCGCGGCTAGGAGATAGGGGTGCAGCCACTTCCTTACGCCTCTGGCGTGGGGTGGGGCCCCTCTGTCATGCTCCGCGAAGCGTCCTCGG[A/G]CGGAAGCGGGAATTTCCCTAAAGAAGAGCCAAATTAAAGTCGGAGCAAAGATCCTTCGAGCCCCAGCTGTCGTCTCCAGCCGTGCTCAGGAAACGGAAAG
2	47854631	BES5_Contig424_396	0.37	0.34	CCTGTCCTATTCTGCTGCTCACAATAGTGAGACCAGATAAAAGGAGAGATATATTTCACTTCTTAATGAGAGGAACGGCCAAGTCATGTTGCAAAGGACC[A/C]TGGAAGCAATTGCCAGAGCCATCCTTGAAAACGCTCTTTCACATCAGTGTGTCAGTTTCAGTAGGTAAGCCCAGCATTTCTGCGTGAGCTCAGTCCCTTT
2	47870030	BES1_Contig523_1341	0.34	0.20	ACCACATCATATTTTTAGTTTAACCATTTGTTTAAGTTATGTGTCAGGCTTTCTTCTATAGGAGTTGAGAAACTTGATTAATGAAAGCTGGCCTCTGTGG[A/G]AAAAAGGAACCTAGTCATTCCATATGTCATAAAAAGCTTGATTGGAGACTGAACAGAATATAGTTCTTACCTTTGCAGAGGAAAATGTGAAGTTCTTTCC
2	48495347	BES11_Contig450_949	0.50	0.49	TATCTCTTTGGACTGAATGCAGCTCTCAGATGACTGCATGGCTTTATCAACAGCTCCTCTGGATTTATCTCCCTTTTGTTGCTCTGATTTCTGGGTTCAG[A/G]TCTCTTGCTCAGTTTTCACATTTGACAAGCTTCCTGTCTCAGTTTCACCATTGCTTCCACATCTGTTTCTCACTTTTTTCCCCAGAAACAGATGTAGCCA
2	49287570	BES3_Contig368_1159	0.30	0.45	CAAAGGCTGCTCTTGTCCCGGGTGAAGGGCCTTCGGTGACTTGATTAGTGCACAGGCTGGCCTCTGCTGATTTTCAGATGGAGACCATCAGATATCATCC[A/G]CCGCCTCATTAGGCTGACACTGGTCCTGCCCCCTCTTGTAATAATGTCACCAGAGCACTTAATAATCTTGGTAAGTGCTATAAGCATGCACTGCAGAGGT
2	49287802	BES3_Contig368_927	0.18	0.34	ATGGCTTCAGTAGAAACATGCAGGGAGCTCCTATTCCATGTCAAGTACCGTGAAAGTGCCTGTGGGTTCAGGTTGCGGCTCATGAAGGAATAGTTTCTTG[C/G]CTTCTCGAGCCCCTTGTTTTGGATGCTTGCCCTGGAAGAGGAGAGCCCAGCTTTTCCCATGGAACATAACCGTCTCCATTAAACTTATTAGCACTAGAAA
2	49512651	BES11_Contig474_1386	0.25	0.38	AGATTTGCTAATATCTACTTCATGAGTGGTCTTCAGGTGTCATTAATTTTCTCTGTAACCTGTGGAAATAAGGACACATGCTTAAGATCTCAGAGTTAAC[A/G]GAAAAGTCTTAATGAAAACCAGACAATTCTGAAATACAAGAGTGGGTTGCAGAAGGACTGGGTTAGAGATGTCTAAGGTTTAGAGACCAAGCAGACCCTA

2	54059503	BES9_Contig556_2651	0.05	0.13	ACTATTCAGGTTAAAGCCATTTCTTGATCCAATTAAAGCTTTACAGAAAAGCTTAGGTAGATGCATATGTTCTTAAACATAGGCACAATTAGATCTCCCA[A/G]TGGTAGATGAATCAGGCAACTCTTTTATTTGGCAGATTTTTCAAGGAAAAAAAGCATAAAATGACCAGTCATTTCCCTGAGCTCCTCTCTTATTTTAATA
2	54416233	BES8_Contig484_903	0.28	0.47	TCTTAAAACTGAGAAATCTCATGATGTGAAACACTTCCATTGATGCGCTTTATGTCTAAAATAGAAACTTAGGTACTTCCAAGAATAAGCAAAATGCAAA[C/G]TTTCCTTGATTACAGTTACTCAGATATATAAAACAGTATCAATACAATATAAATGAAAAAACAGTGAAAGCTATCCACTACATTCAGAGATCACTTGGAT
2	54689629	SCAFFOLD280727_5591	0.16	0.11	AAAGAGAAAAAGTCAATAACTGTTTCTTTAAAAAAATAATAAATCAAAATGAATTTTAAAACTCTGCTTAGAGGTGAGCAACTGCTATATACTACTAGGT[A/C]TTTTGATAGTTGATTTGTTCCTTTAGTATGCAAATTTTAGGTTTACAAAGATCTACAGCTTTTATTTAAGAAATGTGCCTGAAGGCTAGATTGGTAAGCT
2	55260359	BES5_Contig205_709	0.20	0.22	CATATAAAAGAAAATGGACTGTGGCCACGGAGATAGTGGGGGCAGGGAGAGAGATAGCCAGAGAGATGTTCTGTTCATCAGGAACGCAGCCACCTGAAAA[A/G]TACCAGCTAATTAGCTGTCTGTAGCCACAAAACACAGCAGTTTAGTGCAAGAAGTGAACAATACTTTACCTAACTGAAACTACAGAGCAGACTTAGGTAG
2	55611182	SCAFFOLD11405_254	0.50	0.44	TAAATAAAATCCAAAGCTTGTACTTCCTCAATAAGGGAGCAGATTCTCACTTTAATGCCAGGCAAATGGATTGATATTCTTCAGTGATTACTTCCAGAGC[A/G]AAATATTTAAAAGAGATTACTACGGGAAAATATTTACCACTACCAACCACATAAAACACAGTATTGGAGGCTGACAAAAGGACCTTCATGCATACACGTG
2	55611280	SCAFFOLD11405_352	0.04	0.12	GCAAAATATTTAAAAGAGATTACTACGGGAAAATATTTACCACTACCAACCACATAAAACACAGTATTGGAGGCTGACAAAAGGACCTTCATGCATACAC[A/G]TGTAATCTACAAAGCAAAGAAAGAATGATGTTGACGAGTGGGTAAGTACATTTTGTCAAATTAAGTATTCAACATTTTAAAATAGTCTTGGAACTGCTGC
2	56761629	BES7_Contig295_922	0.24	0.22	TTTATTGTGGATGGAGTTGGTTTCTTTCCAGGAATCAATTCCCATTTTTAAAAGGAGGCCTAGAGTGTCTGGGCTTCAGCCAGATGTTCCTGAGAAGAGG[A/G]TGATTAAAATGGTTTCTAATCCCATGGTGAATATTTTTCTCATAATTCTCGCATTCCACCTTGAAGGATTGAGACAAACCACCCTTCTGGATTATAGATT
2	57983536	BES9_Contig535_933	0.46	0.47	ATGGTGGCTACATATACAGGATTGGACATAGGTATTATTGTCCATATATACCAATTGGACATTGGTTTATAGGTATTTACATAAACAAAACAAATGAGGG[A/G]AGAAATCTCTAAAAGTAATAACTTGTGACATCTGCTTACCAACTTCTAATAACCTTTCTCTACTGATACTTTTACTTGGAGGCAGGTTAGCAAAATTTAT
2	60742654	SCAFFOLD125205_4038	0.48	0.47	TCCTTGGACATTTCCAGGCATTGGACTTTTCCATCTTAGGAGTCTTAACTCCAAATTTTTAGTATCATAGCATAACTGGAATGCATATAATTCATCAGGA[A/C]ACTATGCAGCTCATTTCTGTATTTCATTGCTGCTAAATATTTCAAGAAACTTTTTTTGTTTTCTTCTCTCTTAAGTGAGGCTCCTACTTATAAACCCCTG
2	61663836	SCAFFOLD11521_304	0.29	0.33	AATGTAAGGCCGAGATATTACATTTTTTATTTTTATTTGGAGAGAGGGAGACAAAGAGAAATAGAAAGAAAGGGGGAGGAGAGAGAAAGAGAGAAAGCTC[A/G]GATGACAAGATGAAAATAGAGGCTTCACACTTTCTTCCCTAGGGACACACTTTCTTCACACTTTCTTCCCATCATCCTGCCCCATTTTATTAACACCTGT
2	65736730	SCAFFOLD90014_16630	0.17	0.13	NNNNNNNNNNNNNCACAACATGTCACCCCTCCTCTAGCTGGAGTACAGAGTTTAATAGCAAAGAGTGGAGGTTCTGACGTGAGACTTTCCAGGTTCTACT[A/G]TTTAACCTGCTGCTTGATCTTGGTCAAGCTATCAAACTCCTTTTAGCCTCTATTTCCTCATCTGTCAAGTGGGAGTAACATTTCCTACCTGAAGATAAAA

2	67513136	SCAFFOLD190076_2021	0.13	0.16	TACAAACACAGTTGTTTGAATATATACTGACAGACATTCCACTCCTCAAAATTAAAGAATCATTTTATCTTCAGGGTTTCAATTTTAATTTGAGCTAAAT[C/G]CAAATCGAACTAACCATGGATGTTTATTCCCCTTCTTTTTGGACCAGAAAACAAGAAACAAAAATATGTGTTTTTCGACTGATCCTCACTTCTCCACATG
2	68003253	SCAFFOLD1676_7993	0.15	0.18	ATTAAGTACCATAACCTTCTGAGGAAAACAGATTGATGAATGTGGCTAATCTCACAGGGTAGGGAGCCTGGGCCCTGGGAGCTTCGCAGCTGCTGGCCCT[A/G]GGGATGAATCATACCGGAGCGTGGGCAGGCTGTGGGGCAGGAAGGAAGTGGGTGGGCTGACGCAGAGCACATCTTCTATGGCTTGAACAAATCTGCCCCT
2	68006022	SCAFFOLD1676_5232	0.49	0.48	AGAAGCGAAAAGTGTTACTTGAACCCCCATCAGTGATAACCAAATGGGGTTATAAAGATTGCATGAACTCCAATGAAGGAATCTATTCTCCAGGAATTAA[A/T]AGTAACAGCCTGAAGGAATATCCACCCTGCAAACCCACAGACACAAGGAGCCCCTGCACGGATCCCCACAAGGCATTTGTTTATGACACGGATTCTCATG
2	68006082	SCAFFOLD1676_5172	0.49	0.48	GCATGAACTCCAATGAAGGAATCTATTCTCCAGGAATTAATAGTAACAGCCTGAAGGAATATCCACCCTGCAAACCCACAGACACAAGGAGCCCCTGCAC[A/G]GATCCCCACAAGGCATTTGTTTATGACACGGATTCTCATGACGAGGCTGATGAGGACGCTTCCTCCTTAGCCTCTGTCCAAGCGGTTCCAAACCAGGGCT
2	68006157	SCAFFOLD1676_5097	0.48	0.48	AGTGTTTGCCATTCAGCTCCCAGCCAAAGAGGCTGTCAGAGACACTGTGGGTTAATTTACAACTGTGCAGCCTGCAGCCCTGGTTTGGAACCGCTTGGAC[A/C]GAGGCTAAGGAGGAAGCGTCCTCATCAGCCTCGTCATGAGAATCCGTGTCATAAACAAATGCCTTGTGGGGATCCGTGCAGGGGCTCCTTGTGTCTGTGG
2	69408878	SCAFFOLD32416_1317	0.09	0.03	GGGAAGTGATTTTCAGTCCCAGAAAGTAGCATTCACTTGCCCTTATTCAATACATAAAAGGGCAAACAAAACACTTGATTCCTATGACCTCAAACACTTA[A/C]ACAGAAAGCCACCAGCCATTTTATGGTTTTCTAAACATACTCAAAGGTAGATTGCCAGATTGTAACTCTACCACACTTTAGACTACAGCACGTATTTCCC
2	72842877	BES1_Contig385_257	0.09	0.13	AGAAGGACAGACAACATACCCCTAGAAGAGGTTCAAGTGAACAGACACAAAATAATTGAATGGACACTTTGAACTTCATGAACCAATATAATCTGATGGA[A/G]AGAACAGATTAACATGGCGTATACACTGTTAAGCCTCACCTATAGCAAAATTACTACCTTCATTATAATAATGCAGTCCTAAAATAAGACTGATTTTAAA
2	73940064	BES9_Contig365_495	0.44	0.36	AAGCTCAAGAACCTGCAAGCCAGGCCTGGGATGGCCACATGCACCCTTCCCTGGCTGCAGGGCGACACCACACTTCCATCAGGGATGCCTGGGTCCTGGA[A/G]AAAGATTCTTTGCAAGTTGAAGAGCAGAGCCTTTTAATTGCCATGTCCCCAGTGACATAAAATCTCACAAACAACCTCCATAAATCACACCCTGACAGCT
2	74275820	SCAFFOLD26216_19609	0.03	0.05	ATATTAAATGCCATCTAGTTGCTAACACGATGCATCATTTTCTGCTAAATAATTGATCACGGTGCAGAGATTGTGTTTGTGAAGTTTAAAAAAGTGATTA[A/G]AGTAGGGAACATTTCTGGGAGGGAAGTTGCATCTGTAACCCGGAGCCTGCTCTGCGCACCTTCCCAGGGGGCCGCTGGGCTTCAGGGGATTCTTGCCACA
2	76701090	SCAFFOLD321891_17871	0.22	0.26	CCTTCCACCTTTATTTGCCCTACACCAAACCACTTGACTGCTTTCTCCAGACAAGTTCTCTCTCAGTAAGACACTCTTTCTGAGGCTATGGAAGTAAAGG[A/G]CAGGTGTCACATCTGGTTCCTCGGCGGTAGATGCACCAGAGAGGAGGGGCCGTGTCTCCTTGCCCTGAGTAACTGTGGGGCAGCAGCTGCCGTCTCTCAG
2	76716824	SCAFFOLD135477_4544	0.50	0.49	TCAGGGAGCCCCTGTGCTCTACAGTCGTACTCCCTTCACATTAAAGCAATATTGCCTTAGATAACCTTATGAACTTAGAAGGGGTTGAAATCTTGAATTC[A/T]GTTCAAGTTTTTGATTCCACCTCCTGTCAATGTGTACATGGAGTCTTAACTATAAATGTGTTGAAGTTCACGTTTCTTTTTGTTTGATGCCAGAGCCAGT

2	76927418	SCAFFOLD120474_1790	0.09	0.14	TTTTTTCCACATAGACTTCAGCTTTACAATTCTTTTGAAGAAATTAAAGTATTTTCGATTTTTAAAAATTTCTGATAATTTTAAACATGTTCAAAAGTGG[A/G]GCGAATGGTGTAACATATTCTCATGTACCTATCAACATGCTTCAGCAATTTTCAGTGTTTGTCCATCTTGTTTCACCTTTGAGGAAGTTTTGAGGGTTAG
2	77313656	SCAFFOLD70518_22689	0.15	0.14	GAGAGCAGCCTGTTGTATCAATACGTCCTTGAACCTCAGGGCTGAAGGGACACTCCACACACCCTGGTTGGCTGCCTGCTAAGTGCCTGGCACTGAGGAC[A/C]ACATGTACAGGATGGGGAAGACAGCCCCTGATTGCAAGGAGCTCAGAGCCATCACACAGAGAGGATGTGACTAGAAACAGACCCGCAGTCAGTCATTCTG
2	78322512	BES1_Contig549_1508	0.34	0.27	GGCATTAGCCAGGTACAAGGGAAGCAGTGCTGGTCTCTAAGGGAACAGACTGGGCTCAGGAACAACCCTGTTATTTTACTCTGCATCATTCCAGTGTGGG[A/G]CTGATTCAGCTGGAGTTCTGCACTCACTAGAAGCTTGGTGAGTCACCTGCCTGAGTCTCATTTTCATGGATCTATAAGATGATAGTGACTTCTAGGATTT
2	78410203	BES4_Contig185_644	0.31	0.34	GAGAAGACTGAGGCCAATGAAATCACAGTCGAGGTCAACTCTGAGTGAAGCTTGATCATCCTCTTTCCCACATATGAATCTTTTTTAAGACTCTCTACTA[A/G]AGACATGTTTCTATTTCAAAACTTAGCAACATATTGTCTGGCTCTGACTCTCCTTCCAATTCCTGTCCTTCTAAATCATTTCTGAGAGCTAATTAGGTTG
2	78410418	BES4_Contig185_859	0.02	0.07	CTGCTACTTTGGAAGATAAGGTACACGCCTACATATTGTTCACTTACTGCTGTGCACATCTGTGCTCTATATCCATCTGCAGGGATTTGGAAAATTAACA[A/T]CTCTTTAAAGGCAAAACAGGATTATAATCAAAGGGATGTATTTCTAATGGGTTTTTGGTCTTCGTTCTTGAGGATACAACCTGGACTGCAATTGAAAATG
2	79730013	SCAFFOLD175042_11384	0.46	0.39	GCCATTGATCTTGTCGTTTGACTACATCGGCCCATGACTTTTAGGCCAAAACTCCCAACCCTATTAAACAGATGACCATTGGTTGCAAGGTATCCCAACT[A/G]GAGCCTTCTGCCTAGGGGAGAGCTGAAAGCCATGGATAGGGAGGCACAGGGCATACTGCAAATCGGAGCGAGGCAGCCGCATTCAGAAAGGAACCGCTGT
2	79730332	SCAFFOLD175042_11703	0.45	0.48	AAGTGCGGGGCATTTAATTTAATAGGCAGGAAAGCTGGACACCTGGGGATCCGCCACCTGCACTGTGTATTTAAAGAACCCAGTTCGTCAAACTTGAATA[A/C]ATTGCTGGAGCCATCAGAGTTGCAAGTTCAATTGCTCAACTGCTTCTCTAGGCAGAGAAAGGGAAGTTTTTCCAGCTCTGGAAACTCCTAAATGAAGGAA
2	80814691	SCAFFOLD221392_459	0.33	0.29	TGACTGTTACCCCTTCTGGGAGAGAAGGGCAGATGTTGTCCTCAGGATCCCTGTATCAAGAAGAGTTAAATCACACAAGGACAAAATGGTGTAAAGATTT[A/T]AAACTCTAAAGGGGAAATGAAATTGCAGTGCTTTTGTTTTTCTGTCCTTTATCTTAAGAAATCTTCATTTTATTCTACAGAAATCTTTAGAAATGGCAGT
2	80814700	SCAFFOLD221392_468	0.34	0.29	CCCCTTCTGGGAGAGAAGGGCAGATGTTGTCCTCAGGATCCCTGTATCAAGAAGAGTTAAATCACACAAGGACAAAATGGTGTAAAGATTTAAAACTCTA[A/C]AGGGGAAATGAAATTGCAGTGCTTTTGTTTTTCTGTCCTTTATCTTAAGAAATCTTCATTTTATTCTACAGAAATCTTTAGAAATGGCAGTTCTCCCCAG
2	82664319	SCAFFOLD256846_472	0.28	0.47	TAGTCTTTTTCTCATATGTCTTCTAAGTGGTTGATCTTTTTACAGTGTGTCACTATTGAATTTTATATGTTGACTTTGTAATCAGCCACATTACTGGGTT[A/C]TTTTTTAGTCCTTAATTGTGTTGAGCCAACTCTTGGGATTTCTAGATATGCTGTGTTATATTTGGCAAATGACAATAATTACAAATCTTTTCCAATTTTA
2	83412865	BES1_Contig468_700	0.36	0.41	TCCATGGGAAAACTGTCATCATAGAGCTGGTGAACCTGCTCCAGGTATTTGGACTCAAGCTGCTGAAGCTCATACCACTGGGACATCCTATAGGGAAAGA[C/G]AGCACGGATCATAAATGGAAGCCCACAGCTCCAAAGAAAGCATGTCTAGCATTGTGCTTCTTCTAAATATCTTGCTTAATCAAAAACATAATTTGTCTCA

2	83842904	BES7_Contig241_1853	0.13	0.11	AGAGGACTGACTCATAGGTGGCCACAGGGAACTGCTGCCTGCTGTCTTAAGGGAGTATAAAGCCATGGGTAAACCCACTCTTGTTCTCACCAAGCTGCCA[A/G]AAGTTAGCTTCTTGTACCCAGAAAAGAAGACAAGATCTGCCATTTTGTTCAGTTCAGTTTAGTCGCTTAGTCGTGTCTGACTCTTTCGACTCCATGGACC
2	83879823	BES7_Contig421_544	0.37	0.38	TAGCTCTCAGCCTAAAAATAATAAAATTTATTTTTTAATATATACTTGAACTCTTTGATTAGGTCCACTGGCTTTCCTTTCAGATCTCAGTACTAGTAAG[A/G]TTTTTCTCAATATGATCCAGATTCTATGCCCACTAAACATTCATGGATTCTACAACCCCTCAGGAGCTCCATTTATCCTCCAGAGCTGGCGCTGGTCAGA
2	83879976	BES7_Contig421_391	0.37	0.39	CAAGTATATATTAAAAAATAAATTTTATTATTTTTAGGCTGAGAGCTAGCCTATAAAAAAGATCTAGTGACACATAAAAAATTTGCCAGTAAAAATGAGC[A/C]GTCACTAAAGACATACAGTAAGGTTTTAATTTTTTCCATTTTATTTATTTTTTTGATGTGGACCATTTTTAAAGTCTTTATTGAATTTGTTGTAGTACTG
2	86330390	SCAFFOLD195096_914	0.14	0.23	CCTGCCTTCTCCCACAGTCCAAATATGCTCCTTTTATAGCATTTCCCATGCTGTGAATAATATTACAATCCACCTTGGTGTCTAAGTCAGAAACTCTGAA[A/G]TTCTCTTCTCCCCTCTTTCCCCCATCCCCGAAGCCCTTGCATGCCAAATATTTGAGATTTATGTACTCTTGAACCCTCTTTCTTGAAGTGGTTGCTTTCT
2	87627898	SCAFFOLD109829_3868	0.22	0.24	AAATATAAATGTTCCTTTGTCATGGCTATACAGACAACAAGGTATCAGGAAACTGTCTTGTTATTCATTACCTATATTGCTTAACTTTTGGATTAAACTT[A/T]TTCTTCCATAACTGAATAATATATGCCTATCCTCTCTTGGTGAAAAACAGAGGATTGTGCTTTGTCTTACAATGTCCTCCTATTGAAGAGAAGAATATAC
2	87627946	SCAFFOLD109829_3820	0.25	0.32	TGTGCATCTCTGCTGTGCTGGCAGAATGACAGCTAGTAGAACCAGCAGGTATATTCTTCTCTTCAATAGGAGGACATTGTAAGACAAAGCACAATCCTCT[A/G]TTTTTCACCAAGAGAGGATAGGCATATATTATTCAGTTATGGAAGAATAAGTTTAATCCAAAAGTTAAGCAATATAGGTAATGAATAACAAGACAGTTTC
2	87628041	SCAFFOLD109829_3725	0.24	0.33	AAAGCATATTTTCCTGTAAACCATACTGGAATCTTTCATGGAATGATGTGGAGTATAAATATAGACATACAGACAGAAATTTATGTTGTATATACTGTGC[A/G]TCTCTGCTGTGCTGGCAGAATGACAGCTAGTAGAACCAGCAGGTATATTCTTCTCTTCAATAGGAGGACATTGTAAGACAAAGCACAATCCTCTGTTTTT
2	87639418	AF440377-177	0.08	0.19	GGAAATATTTATAAGTATTATGTAAACTTACTTCCCCTCCCCCTAACAACATTGCTTTTCTCTAGGTTTAGCAAGTTTGCAAAAACATAATAAAAATTAA[A/G]TTTTGAAAGCTATTTTCCTTTAAGAACCTAGGAGGGAAACAAATGTCCTCTCTTCTAAGTCTATGTCTATAGAACATTTAATGGTAGAAGGAGAAGAAAA
2	89062660	BES3_Contig278_695	0.09	0.12	TATTCAAGACAAACAAGCTAAATATCTCCACCCTTCCCTGTGATGCAGCTAAAATACAAATTATAAAACTAAACCAGTAAACATTTTCTACAAGATCACA[A/G]TTGAAGAGCCTGAAAAAGAAAGCTACTGGATTCTCACTGCAGCTGATGAGTGTGAAGTGTTTTGACTTTCAAAATTCATGGATTATAAAAAGCATCAGTG
2	91800274	SCAFFOLD296264_1970	0.50	0.41	AATAAAACTGATGAATTTTATTCAAATAGAAGTTATTCTGAGTAATAATAGAGTTGGAGAACAAGTGGTTCTTCTTCCCAGAGTTTTGGATGCCTAATGG[A/G]TAAACTAGGATTAGAAATAACTACATGACCTGCTTCACAGGATGAAAGATCAAATACAGGTCCTTTTCCAGACATACTTCTTTTCCTTACCTTAGGTTAA
2	91800423	SCAFFOLD296264_2119	0.45	0.48	TCTATTATTACTCAGAATAACTTCTATTTGAATAAAATTCATCAGTTTTATTGTATAAGCCCTGTAAGTTGCCAAGTTTAAAACCAGATGATGTGTAAAT[A/G]TACTTACTGTGAAAGAACCCATAGAATTTAAGATCTATATAGAAATTTACTTACAGAATATTCTTATTACTTTACAGATGAGGAAACTGAGGGCCAAGAA

2	92309638	BES8_Contig415_403	0.47	0.41	TAAATAAGCACTTGATATGACAGTGCTGATCATAATTAAAAAAAATATTTTGTAAGGCACCATGTGGCGAGGCTAAATTCTCTTCTTTGTCTAAAATAAT[A/G]TCTGCCCCACCCTTATTTCCTATGTTCCATAAATTGATACCATGGATGGTATATGGGCAATGGACAATAATGCTGCTTTGTTGTTCTTAGGAGTGAATAC
2	92419324	BES2_Contig368_774	0.20	0.29	AGAGACAGGGCTACCACCGGTGCTAAGCCCCCCAAATAAACGGCCCTCCTCAACCCCCCACCAGCTGCAGTCTATGCGCCCTTATTTCGTGCGGAAGTCA[A/G]GGGCTGGTTCTCTACAGGACTCAGTGCACAGAGGCACTTAATATGTGTAGAATAGTGCTTTCTGCCACACCCCCGATCCCCCACCACCAAGCGACCAGGT
2	94033574	BES4_Contig478_1334	0.02	0.04	ACCATGACTGACATTTCCCTTCAACATCAACCAGTTATTTTAGTTTCTAATTAACTTTAATGAAACCTGCAGTCCCTTTACGCATTAGCTTTGTGAGATA[A/C]AGATAACGTAAAACACAGAGAAAAGATTGGTCATAAGTTTCCACAGCAACCTGCGTTTATACAAGTGATATTTTTCCAAACCAACACAAAAGGAGGCCTT
2	95276320	SCAFFOLD70270_19591	0.47	0.40	CAACCCAGAGTACATCTGGATTCACATAAAAATTGACCCATGTTTTTTCTTTTTAAATCTATTTTTTGTAAAAAGAAATGGTAATTGATGAATAAATTAA[C/G]ATAGAGATTTCTTTCCCTGGGAATTTTAATAAATTATCAGGGTTAGGTCATGGTAGTGGGGAAGAAAATAGCAATAATGCCAATTAATGCTAGATACAGA
2	95762272	BES9_Contig524_1040	0.08	0.02	GAAGGAGTGATTAGGTACAATGTAGACTGACTATTTTCTCTCTTCTGTTTCCCTCTGTTAAGAAATCCAGCTCCAGTTCAGCAATGGTACAGTGACGATT[A/C]CATAGCAATGATTGGTAGCCTGTGAGCTCATGTTGAATGCTTGTTATAGAATTTGAAAGGGCAAAGCATATTTTGTGTACCAGCCCTCAACTATCTCCAT
2	95764841	SCAFFOLD15351_11326	0.44	0.02	ATCTTTTCTTTATTAATGGGGTTGTGAAATTTCATTATTCCGTGCTTTTGCTGGGCTTTTTTTTTTTTTTTTTGGTCACATTCTCTGGGATGGTCACCG[A/G]ATGGTTTTCAGAAGGGAAATTGTTACAGTATTATTCTTCAGTAAATTATTTCCTTTCATATGCTAGTCCTGTCCTGCTCTGAGAGAATGACCCCTGTACA
2	96971145	BES2_Contig197_1122	0.18	0.19	ACCTTTGGCAGCTGTGCTGTAACCAGACATAGCCAGGTGTTAACTGAAATTGAAAATAGCCTTTTCAACAGTCCTAGGTCCTCATTATTTTTAACATCAT[A/T]GTTCATGTTGCTAAAAATATTCCTAAATCTCTCCCTATGGATAATTTGGAAGGGGAGGTATTATATTAAGAAATAGATGGATAATAACCCCTCCCTACTG
2	96971207	BES2_Contig197_1060	0.18	0.19	ATAGGGAGAGATTTAGGAATATTTTTAGCAACATGAACTATGATGTTAAAAATAATGAGGACCTAGGACTGTTGAAAAGGCTATTTTCAATTTCAGTTAA[A/C]ACCTGGCTATGTCTGGTTACAGCACAGCTGCCAAAGGTGAATGTCACACTTCAGAACATAGCATCCCAAATTTCACAATGCTCCCGGAGCTTCAATACTG
2	98678609	BES5_Contig306_381	0.13	0.20	GGCCACCCACTGTGCAGGAGCCATCAGGATAATGGTGCTATTTAAAAAGGAAGCAGGACAGCAGGATGCATGAACTGCAATACAGTGGGTGAAAGCTGTG[A/G]GGTAATCAACAGATAGTCATTCCTCATTGATCAGGCCCTCACTGCGTGCAGAAGAAAATGTGAAGAAGATCAAGTGTGTGGATATCATCAGAGAAATAAA
2	98880148	BES3_Contig215_1033	0.04	0.06	GGAAGTCCAAGTGCTATGAAAACTTTGCCAAAATAAAAAGCTTTTAGATTTCTAGTGCTGGCTGGACACAAACCCGTGCAAGAGTGTTGAAGAATAATGC[A/G]TGCTTGCCAACTGCCAGATACGATTGCTTTTCCCATAATTAATGTCATTCTTGCTGTTTGGAGTACCATTTCCATCCTCTAATAATTTCCACTAATAGAG
2	98897411	SCAFFOLD275028_33882	0.49	0.46	AAATCCTCTGTCCTAGGAATTACCCATGAGGAAGGGCATGCCAACTTTAACCTTGGAGCTTCCTGTGGGTGTTAAAAAGCAGGAGAGAAATGTGAACCTG[A/G]GAAGGTGTTTTTCGCTTGATTTAGAACCTTTCTGAGTTCTTTGGGGAAAAGGCAAATATTGCTGAGAGGCACTGACCCATTGAAGGGAAGAGACATCTGG

2	100663550	SCAFFOLD200710_15727	0.33	0.40	GTAGCTCAGATGGTAAAGCATCTGCCTGCAATGCGGGAGATCCTGATTCAATCCCTGGGTTGGTTGCCTCTTGGCAAAAGTGGGATTCCTGCACCATCAG[A/C]CCTCTGTCTGACAGCACTCATCATGGCTGATTTTGTTCCCAGAGTTTTTATAAGTGCTGGCAGCAAAGTTTCATTTTCAAACACCAAGAACAAAGGCCAT
2	102694242	SCAFFOLD305182_13137	0.50	0.08	GCACAATGCCAAGATACAATCACTGTTAAGTCTGAGCATGACAAAGAAACAGATGGTCATGATCTCTTCTGTAGGGAAGGGGATAAAAAAAGAATAATGA[A/T]ACAAGACACTTAATAGCGAAATTGTTCATGACTGCTGAGATTCTCGGAAGAAAAACAAAATGAAGCTGCTAAGGTAATGAGATAAAAAAAAAAATACATG
2	102926801	SCAFFOLD86037_9839	0.27	0.33	TGTGCATATATGTGTATATATATTATACACACATATATATGAATATATACAGGCACATAGCCATCTAAAAAATGATAGCATAAAGACTGTGTAAGATCTC[A/G]TAAGACAAAGGTAGGGGTTAAGAGGTGTAAGCTATATTTATTAATATATGATAAATAAGCTACAAGGACATAGTGTGCAACACAGAATGTATTTTACAAT
2	102930477	SCAFFOLD86037_6323	0.27	0.33	CTGAGCTGTTCTTGACAATACTTGCCTTGTGCTCCTTCTCCAGCCTGGCATGAGAAACACTTCCTTCTGTAGAGCAGACAGATGCAGGAAGTGCGAGTCA[A/G]TTAACTATTGTTTGCTCTTTCTGCATGGTAAGGAAAAGAAACTTCTTTTTCCTTCCTTTCACTTCCCCCACCCCTTAATTTTTACTTAGCCTGGTTTGCC
2	103202765	BES11_Contig247_1378	0.38	0.39	GGGGAAAAGATTTGAACTATCAATTCATCAAAAAAGAAATGCAAATAGCTTTTTAAATGAAAAAGTTTACCCTTTCTACCAATCAAATGCAAATTAAATT[A/C]TATCTAGAGATATATCATTTTGAGTACAAAAGATAGTCCAAATGAAAAGTCATAATACCCAGTGCTGACAAGCTTGCAGGTAAAAGTGACTCATATACTC
2	103202891	BES11_Contig247_1252	0.28	0.17	AAAGGGTAAACTTTTTCATTTAAAAAGCTATTTGCATTTCTTTTTTGATGAATTGATAGTTCAAATCTTTTCCCCATTTATCAACTGAAATCTCTGAAAC[A/T]GTCACATGGGTCAATTAGAAATGAAGCAACTACTTTATCAACTTAAAAGGAGATTTTCGATCTTATAAGAAGATTAGAAATTATTTTAAAATCATCTCAG
2	103573251	SCAFFOLD175049_6551	0.25	0.23	GTTTAATACTATTAAAAAGAATTAAAAACTTTCCAGAAATCCCTGAATTCAAGACCACTTTGTGATTCAGTTTTACATTGAAGTCCAGACTGACTATATT[A/G]TGAAAAGAAAATAAAAGGTAGCTCAGGGCCCCTTGTCACACACTACCTTGGCAATGGCATAGATGCGGATGCTGGATGGGTCAGCCAGCTCTCTTCTGAG
2	112999439	SCAFFOLD225055_17270	0.40	0.44	AGACCCCTTGAGTATGTGACAGGGCAGTCACACCACAGGTTTATAGAGATGTGACAAAGATGTGATATCTCCATAAGTGTTTGCAAATTAAAGACATTCA[A/G]CCAATCACATGGATGGGTTGCACAGGAAAGTGAATGCATCCAATAACTCAATCAAAATCGCTACTGTTTCCTCTGTTTCCTGTCTGCTGGACCACCTCTG
2	113028601	SCAFFOLD40021_21824	0.15	0.16	TATTTTTCTGGATAACTTATTTAGGGAAAAATAAAATAATAAAATGATCTTAAAATGAAAAGGCCTGAAAATAGCACTCTTATTTAAATTACAATACTTC[A/G]CACTCATCTGAGATTTCTTCATCTTTGCAATAACGCAGCTGTTTTCCTCAGGGTGTTCTCACAAGTTCATAAAGAAAAAAATTGTTATGAGCCTTCCCAA
2	113029112	SCAFFOLD40021_21313	0.29	0.31	CATCAGATGTCCAAGTGTCACATTTTTCTCCACATTCAGTAGAGGGGATATGTCATAAATCAATGGCATTATCCAAGAGTCTTAGTTAAGAAAAAGGGCT[C/G]GAGTGATTGAAGAGTTGGGACTCTGCCAATCCAAAATTCTCTTGGTTCAGAGGCAGTGCTGACTCTTCCTTGCTCTCTCACCTTTGCTGTCAAAGCACTT
2	113502069	SCAFFOLD145989_4929	0.05	0.03	TAAGTGGGTTCCTGTCTTTGTGTATGTGCTACAGGGCATTCACCATTCTCTGGGCATTAGCAAGTGAAAAAAATGCCTTCCTTATTTTTATGTGGCTGAA[A/G]AATGGACCTAGAATTAAATTCAAACAAAGCTTGGTTCCCTGGGCTCTGGATATCTACTTACTGCTCTCATGAAATTCTTTGAGTTCTCAAAATCATGGAT

2	113632777	SCAFFOLD147091_1390	0.35	0.37	TAGGTTCAGTTAAAATCCAGAATGTTGGTTTCATAATTTCATTCCTCACCCAATTTCTCCAGCCTCTTTGTCTTCGGGGTGCCTGGAGACCGTGGACAGC[A/G]CAGAGCCAAGCAGAACCAGTAGCCTGTTCTGGAGATTCTGCTACCCCTCAAGTGCAGTGGGAATTCCTTTCTGCCTGAGTGAAATGTCTAGTCTATAAAA
2	113633123	SCAFFOLD147091_1735	0.40	0.27	GTTTGTTTAAGGCTGTTGCTCAAAAAAAAAAATTCTGAAAAATTAATACAATTATTCTATGCAGCTTAAATTATACCTGGTCTATGGCTTTACTTAGCAG[A/G]AATCACATCAAAATATTTTATTTTATTTCTTAATCATCCCCATCAGTTTAGTCATGATTCTGGGGTAAAAGGAAAACCAGTAAGTAAAGCAGAAAGAAAT
2	114024730	SCAFFOLD1249_643	0.38	0.28	GAGTCAGTGCCTCACAGGGGTCTTCTTTATCCTCTTAGTCTAAGCTACATGACACCCCACATCACCTTGAGCCTATCTTGCTGGCAATGTTTTTAACAAC[A/G]CCAAGTACTTAATTGATTTCCCTCTTGGTTCCCCTACTAGACTCAACGATGAACAGAATCTTAGAAATCTTTTCTTTTTACTGTCTATATCTTCGTGCCT
2	114312649	SCAFFOLD146214_10543	0.33	0.30	TGCTTTGCCTCATATAATCAACAATCCAAAGAACTTCATTTAACTCCATGTTGCAGGCAAAGAGACTGAAGGGCAGTGTGGTTTTAAAAAACTTGCTCAA[C/G]GCCATGCAGTTAGAAAGTGGTAGGTAGAGCTGGGATGAAATGTCCTGAATCCAGGTTCTGATTTTAACCACTACTTCACATCTTCTCTTAACACATTTGC
2	115024545	SCAFFOLD210095_19074	0.35	0.48	TTTAGGGAGCAGGTGAAATTGAACCGGGCTGCAGTTCATCAAACGAAATAAAAACTTGCTGCCAGGGAAGGTTCTGATGTTGTGTGATATTGCATAGGCA[A/G]TTATAAACAACTGTGTCTGATAGCAGTTATCCACTGATATGTGCCTCGTGCCCTACTTCCTTACTACGCAAAATGTGATTGGAAGGCCAGGTGATGCTGG
2	115073815	SCAFFOLD311725_27754	0.49	0.01	AGAGTCTTCTTAACTTAAAAAATAATAATCACATAATTGGGGGTAGCTTGGAGTTCTGTTTTTTGTTTTTTTTTTAAAACCACCAAGAAGGTTCCTTGCC[A/G]TATTAACCCCAGAGAACATAAGTTCATTGGTACCTTTATGCCAACAGCAATTTCTTCAGCTGTTTGAGATAGACGGGATGCACAGTATTTAATTAAAACG
2	115228933	SCAFFOLD60587_8279	0.30	0.39	GACAATAGACTAGCAGTCTTGGATTGCAGTTCAGAACAATAACTAAGATACATTTTCCAAAAAGCAATTTGGCAGTTCGTGTGAAGAGTTCTGAAATAAG[A/C]TTTTCTTCCAGTTCTCCGGAAACAATTAAATATTTAAACTCCAGTTGAAGTACAAAGATAAACAATGAAACAATCAAAATGCATAGTTAAGAAAATGCTT
2	117480271	SCAFFOLD125013_22087	0.30	0.32	GGGTCATGTGCTTCCTATGCTCCCACATTCAGGTACAGTATTCCAAACTGTCATGAAGTTGTATTTGTCAAGGTAGGAAAAGCCAACATACTTTATTTAA[A/C]ACTCTGTTAGCTTTTAAATACTTAAACATACAGCTTCCGTTTTTACTCGCCTCTACCCATCGTAATGTGAGGGGTGGGCCTGCTACAACATTTAGTTTTG
2	117480292	SCAFFOLD125013_22066	0.30	0.32	GTGAGAGGAAGGGGTGGGCCAGGGTCATGTGCTTCCTATGCTCCCACATTCAGGTACAGTATTCCAAACTGTCATGAAGTTGTATTTGTCAAGGTAGGAA[A/G]AGCCAACATACTTTATTTAAAACTCTGTTAGCTTTTAAATACTTAAACATACAGCTTCCGTTTTTACTCGCCTCTACCCATCGTAATGTGAGGGGTGGGC
2	119698805	SCAFFOLD6014_7700	0.22	0.36	ACCAAATTACTTAAAAAAAAAAAGTTAAGTGGTCACACAGAATGAGCTGTGCATCTCTGTCCTGGCCTCCAGCACGCCTGCAAAGCCAAGCAGACCAGCC[A/G]TGAACATTTGCATACGTTAAAAATCACCAACTGCATTATGCCTGCATTGGGCCAGTGAAGAGTCATATAATCAAGCGACTAAAGAAGGTACCACCATCAA
2	119775996	SCAFFOLD22046_4722	0.46	0.47	ACCTGAAATGAAACCCTCTTGCGCAATATAAGATTCAAGTTGGTTTCTTATGGCAAATATGCTCTAATTTAACGTATGGAAAAATTCTGTCCTTTTAAAG[C/G]CTTCTGGAAATTCCATATTAATTTGCCAAGTTCTGTAGATTATTCACAGAAAGGAAATACAAGATGAATCATAGTAGTTCTCCATCGTGTTGTTCTGTGG

2	119892036	AJ496786-032.T7-100	0.24	0.29	AAATATAAACTGTATTCTATATTTAAATGACATTATATAGTTTTACTAGTTTTATAATATATTTTAATATGTTTAACACACCATTTAGTCACATATACAC[A/G]TGTGTGCACATGCATATTATGCACATATATGTATATGTATACATGTGTATATACACGAACATATACAAGCACACGCACACCAAAAGAACTGTACCAAAG
2	121878191	SCAFFOLD126643_8480	0.08	0.14	ATTAGTTTGGTACCTTTGTCACAATAAATGAGCCAATATTGACACATTATTACCAACTAAAGTCTACTCTTTATTCAGGTTTCCTTAGCTTTTTCCTAAC[A/G]TCCTTCTCCTATTACAAGATCCCATATTACAGGTGGCCTTTATGGATTCACAGGCTCTTCTTGGATGTGACAGGTTCTTTCCCAGACTTTCCTTGTCTTG
2	122730626	SCAFFOLD106798_4891	0.14	0.20	GAATATCACAGTCATGGGATTAAGATTTGTAATTATAGGCTATTTAGTTATATAACTTCCTGTCATTGGAAGCAGTGTACAGTTAGTGAAGTTTGGTATA[A/G]TTCAGCTTTTCTTTAGATCTTCCATAGAAAGTCTGTAGACAATTTCTTGTATACCTTAGTAAGATATGTAGGTAACTGTTAATAATATGATGAAGTGAAC
2	124176652	SCAFFOLD266452_1462	0.31	0.35	TACCTTGGACAGAGCCAGGTATGGGAACTGGTCCATGGCCAGGGGTGTCTCAGGTCCCGGCTTGCCAGCCATCTGCCCCTTTAGGATCCAAGCGGCTGTC[A/G]CCGTGGAAACCCCCATCCCTGAAGGTACACTGTGTCAGGTCTGAGTCCCTGGGGCCAGCCTGGGTCAGCCGGCAGCCCAGGATTCCAGGGTTGTCGGGGG
2	124585178	SCAFFOLD76057_3389	0.09	0.19	TAATCCAATCACTGCAGCTTCTGGGAACCCGTTCAAAGGAGACCATCTTCAGCATGGGAAAACTTTATGCATCAAGATGTTCACTAGTTTCTGCTCATTC[A/C]TGCATTCAGCCCACGCTTAGTGGTCACAAGCTCAGAACTGAGCACTGCGCTAGGTCAAGGATTCAGAGGTGAGCGAGACACAGACCTTGTCTTCCAGGAG
2	125174535	SCAFFOLD151694_4517	0.10	0.15	CTGGGAAGGTCCTCTGTGTAGAGTACAACTATTTATCATAGCATACTAGGCTCTGTACTTTCTAGATCCTGTGTGTCTCCGTAGCTCATTTTTCTGTTCA[A/G]TCAATTCAGTTACTCACTCAGTTCCAACTCTGCGGCCTCATGGACTGCAGCACACCAGGTTTCCTTGTCCATCACCAACTCCCAGAACTTGCTCAAACTC
2	126677800	SCAFFOLD5242_24725	0.16	0.24	GGAGAGAAGAGACTGAGGGCTTCATTCATTGAGCCCTGGCCCCGAGACAAATGTCCAGGCTTTATCTTCATGAAGTTTAAAAACAGTTGAAGTCCAGCAC[A/G]GCGATCCGAGAGCAGGTGGTGAAAAGGATGAGCTGGATGTGAGTGCAGGCGTCACCCTGCCTGGACATAGGGTGTGATGCGTGCAGGGCCTGCCCAAATA
2	126784799	SCAFFOLD25500_5689	0.48	0.33	AGGTGTGCCACTTTGGGTCAGAAATCATTTGTGACCCAGGGAATCCTCTGCACAAACCCAGGGAAGTGGGGAAGAAGGTCCTCCAGCCGTTGGGCGTGGA[C/G]GTCCTGCAGTAATAACACCCCAATGACTCAATAAACGGAGGTACTGTCTCAGGATGCCAAGAGCCCAATTTAACCCTCCCTAGAGGGGCCCAGTCCACGA
2	126851875	SCAFFOLD20054_13555	0.04	0.09	GCCTCCAACCATCAGCCCCTCCAGGTCAGAAGTGGGACACTTGAGGGCAAAGGAACTCTTTAATTCTCTGGAATAAGGAGTTGTGTTCTTACACCGAAAT[A/G]CACCTCAACAAAACAAGGGCCACGAGCAGCAGGATGGATGACGGGCCTGTTCTCTGCAAACACATTACAAGCTGAACTGTCCAGCCAGCCCAGAGCTTGT
2	127116677	SCAFFOLD30421_16346	0.48	0.41	GGGCTGCGTCTCTGCCCTGCAAGCAGACCCTGGGAACTCCCAATAAATACCCACCTCCACGCCTCCGGGAACCACTTCCCTCCAGTTTGGCAGCGCACAG[A/G]CCAGATGCTTGCTGCTGCATCTCTCTATATACATGCGTGCGTGTGCACACACACACAGTCATGCACACCCAGGATAGGCAGTAGCAAGGAGGAAAAGCTG
2	127506281	SCAFFOLD115026_11808	0.14	0.25	TGTACAACTTTCATTTCACACCCAGTGGATAAACAGGTGGAGCATTTCATAATGAGCTCCAGCTCTTTCAAATCCATAAACTTCTACGTAGGAAGAAACC[A/G]TAAGAACTCAATAAAGTCACCAATTTTGAGTTTGGGCAGCCACTATGGGGATTCCCTGATGGCTCAGTGGTAAAGAATCTGCCTGCAATGTAGGAGACAC

2	127508327	SCAFFOLD115026_9762	0.23	0.32	ATCAATCAAGAAGAGACTTAAATGGAGAATTATGAGTGAATCATTATCCCATGAACAATTGCAAAACAATGGGTACAAATGTTAGAGTAATGGGAAAGAT[A/G]AATTTACTGGCTCAGCCTGACTGTGAATGACTACTCACTCAGTCAGACAAATATCCTATCATGTGAGGCTCGGAAAATGAAATGATGTTTCTCAAATTCC
2	127508328	SCAFFOLD115026_9761	0.08	0.21	GAATTTGAGAAACATCATTTCATTTTCCGAGCCTCACATGATAGGATATTTGTCTGACTGAGTGAGTAGTCATTCACAGTCAGGCTGAGCCAGTAAATTC[A/G]TCTTTCCCATTACTCTAACATTTGTACCCATTGTTTTGCAATTGTTCATGGGATAATGATTCACTCATAATTCTCCATTTAAGTCTCTTCTTGATTGATC
2	127824393	BES8_Contig556_1629	0.49	0.48	GGCTCTCAGCTCTCCCATGTGTCATTTCTAACTTCCCATAAAAGCCCTTGGCAGAGCGGATTTCAGTCGCTCGAGGGGGATATTCAGGTTCAAGAAATAG[A/C]AAGCTTATACGTAAATACTTGTAAATAAAAAAGCTTATAAACACATTAAATGAACCCAATGGACAGAGCCCCTTTCCTGCCCTGGGAAGGCAGACGCAGT
2	129305805	SCAFFOLD225703_6040	0.44	0.30	ACCGCAGCCAATCAGATGCCTGTTTCCCGGGGAGGCGGGGGAACACCGCCCTCAGGAGCCTGGGGCTCAGCCAATCGGCACCGGAAAGCGCCCGGGGAAT[A/T]CTGGGAGCTGTAGTCAGCCTCCGGCGAATGCTGCAAACTTTTGCAAGAGGCTTTAGTCAGAATGTTGGCAATACCAGATGAGAGTGACAGCCCCGCAGAG
2	129308207	SCAFFOLD225703_8029	0.45	0.32	GGATAATCCAGGATTCATCTGTTATCTCATGTAATTCTCACCACAATATCATGAAGTAGGCATTATGCCCCTATCTGACAGATGAGGATACTGAGGCTCG[A/C]GAGGTGTGTTGGCAAAAGTCAGACAGCTTAGTAATATATTGAGTAAGAATTCAAATGTAGACAGGAATGACTCCAAAGCAGAAACTCCACTGCATCACTG
2	129427386	SCAFFOLD175431_18826	0.35	0.44	CCTTATTCAATTGTATCACTTTGAACATCAGTTTCATGAGAACAGGGACTTCTATCTGTTTTATTCGCCTGTAACTCCATTGTCTAGAGCAGTTCCTGAT[A/G]CGTAGCAGGTGCTTAACAAATACTGGCCAAATGAAAATTACCTTGTAAACCTACCTCATGCATCTAACCAAGGTTTTTACTTTTGTGAGGAGGCTGTGTA
2	129578917	SCAFFOLD280014_15491	0.42	0.30	CAGGCATTTCCTTCCAACCCTACATTTCCGAGAGAAACAGTTCTAGGCATGGGCCTTCTGTCTCTCAGGAGGGGAGGAGCCAGAGTATAGATGAATATGG[A/C]AGTGAATACGGCAGCGACGTCTGTCACAGCTGGAATTCCCTCCCTCATACTGTGTAACTACAACTGAGACCCTGTATCACTTTCAGTGACCTTGGGAAAC
2	130431756	BES10_Contig561_321	0.08	0.08	AGATACAATTCCTGCTTATACCTGGGCTAGAGGAACCTACTCTCCTGTAGGAGGCCCCCACCCCTACCCCACCCCCGAAAACAGTTCTGGGAGAACTGAC[A/G]CTACTTAAGTTCAGCCACTTATAGACTGTGTGACCCTAAACAAGTCCTCTGCCCTCTCTGGGTTCAGAGGATTGAATTAGTCTCTGATCTCTCCAGCTAC
2	131660461	SCAFFOLD195051_23454	0.30	0.30	CTCCGGGCTTCAGTTTCTATGCAGATAAAACAATGATCACACTAATCCCAGCTGAGGACCTTGAGGGGTGAGGAGCAAGTCACAGCCGGACAGCGGGTAT[C/G]AGGTCTAACCTTAACTCCACTGGCTTCAGTTTGGTCCCCAAACGCACGCATAGGGCTCAGACCTTGAGGGCTGGATCCAAGGCCTGGCTGGGACACCACC
2	133058384	SCAFFOLD13026_1235	0.26	0.28	CATATATATATATATATATATATAGCCGGTAGCATAAAAATGGAGTGGGAAGAGCCTGGAGGCTAGGAGACCAATTATTAGATCATTCATTGCTTCACCCTA[A/C]AAGTATTTATCTGATACTGGGCTCTGCACTTTGACTATTTTCCATTGCATTCCATCCCTGGCTTGGTCCTCGGAAAAGGAAGTGAATAAAAGTTGGTAAA
2	135226320	SCAFFOLD107820_1332	0.29	0.31	GACCTAAGCAAACAAAAACTTCAGTAAAACCCAGCTGACAAAATCCACTGCTCATGACTACCAAATCAGGCTCACATGTTTAGTTAATGGGTACGTGTAA[A/G]ACACCAGTACTTGAAGCTGAAAACACATAGAAAAAAAAGAACTCAACAAAAATCTGCGATGCTAAATTAATATGGAGGAAACTCTGGTGAAAAGGAGAAT

2	135843820	SCAFFOLD126235_9784	0.29	0.41	TCTGAGGACAGGACTTACTGGAAAGGAGCATGAGGGGGCCGTCTGGGAAAGCGGAAATATTCTGGAAGTGTCCTCTATCTTGATGCCACCGGGGTTACAC[A/G]GGTGTCGACAGTTGTCAAAACTTGTCAAACCGCTTGCTTCTGGACTGTGCAATTTTACTCTATATACCTTACGCCTCAACAAGGGAAAAAAACGGGAAGA
2	136510105	BES2_Contig213_139	0.45	0.44	CACAACTGGACTCTCCTCTCTTCTCAGGGTGCAACATAGAGAATGCCTGCTACCCGCTGAGCGTCTGTGCTGAACGAACTGCCATCCAGAAGGCCATCTC[A/G]GAAGGGTACAAAGAGTTCAGGGCAATCGCTATTGCCAGGTGAGTGAGAAAGGCCAGTCGTAGCAATACTCGTTCATCTGTCCAGCCAGCGTCCACCACAC
2	138317492	SCAFFOLD115441_12359	0.43	0.23	GTCCCAACCTCAGGCTTTTGTTAGGACAAGGAAGGGAAGGCATATAAGGGATTCAGACCAATGTCTGGCACGCGGTAGGTGGGTAATGAATGGTCGGTAT[C/G]GTTATTCATATTACTTTGCGCTGCAAATACTTCTGAAGCCAGCAGAGCCTGATCCTTGCTGGTAAGGGTTTTAAAGGACCGCCCCCAGCCTTGAAATGTA
2	138317817	SCAFFOLD115441_12034	0.42	0.45	CAACAGCAGCTATTCTCTCTCCAGGTCTCCGCTGTCTCACCTTTAAATTGGAGGGCAATATACCCCACAGGGCTCGTGTGAGGGTTAAAAGAGAAACCCA[A/G]CATATTGCCAGGCAGAGACACCAGAGACAGTCATGCCTATAAAGCGTCTGGAACACATGCCTGATGCATGCTGAGTGCTCGGTTACATAGAGGCTGCAGT
2	138454433	SCAFFOLD167367_1606	0.20	0.24	AGTTACTTCTCTGAGAGCCTCAGTTTCCCTGTCTATAAATGGGGCTGATGAAGCTTCTTAGAGCATCAGACAGGACTGAGGATTAGACGTGATGGCACAC[A/G]CCAAAGGCTCAGCACGGGGTCAGCTAGGTGGCCGTCAGAGCCGCTTTGGCATTTGCTGTTGCTATGCTGCTGATTACTTAGGCACCTGAATGCCTGGCCT
3	1071569	SCAFFOLD155050_8253	0.44	0.42	GTTCCCCAACCATGGTTCCAATTTGTGACCCTTCATTGCAAGATGGATTCTTAATGACTGGACCACCAGGGAAGTCCCCTAGATTGAACTTTTTGGTCAA[A/C]CTTTTGAAACTTTCTCTGTAATTTATTCAAACTTTGGCTGTCCTTTTCCCTATACAGAGAATATTTACCTGTGGCTTTTCTTGTCAAGCTTATTAAAGGT
3	1748195	SCAFFOLD265415_14273	0.35	0.30	GTGGATAAGTAGAGCCAGATGACCAGAAATCAAAATAAACCACTGGTGAACGTGCCATCCATGAAAAACTTCCTGGCGTCCTGGCGTCACTCAAAAGCTT[C/G]CCTCAAGCACGGCCCCAAGCAGTTTAAGCAAACGTCTGATTGCAAAGAGTGTTCAGTTAAATCCGTTAAATACCCTGGTCACTGCGCAGCCACGCCAACC
3	2653219	SCAFFOLD280587_5601	0.45	0.36	CCAGGTCGAGAGACTGGATCCTCATCCAAAGCAGGGAGAGCTGATCACAGCTCAGCCCCCACCCAGAGCCAGTCAACCACAGGAATGAGCACCCTGAGAC[A/G]GTTCCCCTGCCAACACAGCCCTGCTGTCTCCCTCAGTCACAGACCCTCAGACCACGGGGGTGCTGGCGGTTGCCTGGGCACTAGTCTCCAGTTGCAGAGC
3	3695334	SCAFFOLD120100_15841	0.40	0.10	ACAAAGTTAAAAACTCAATGGGTAGGTTTTAGACAACAGAATAGATGTGAGACCCTTTGCAAAATTGTAGTTAAAAAGAAGAAAAATAGAATGGGAGCAA[A/G]AGACTCAGAGGAAAGAATAAAAGGTCCTAATATATGTAACTACAGATCCAGAGGGAGAAAGGTGAGGGGCACGTTAGGAAAGATAGTAGCTATGAATTTT
3	3794710	SCAFFOLD125136_12607	0.30	0.46	TAATGTGCCCTGTCACTCCCGCCTAGAAGTTAAAGTGGGAACAGAGGATAGTACACAGGTTTTTCCAAACTTACTTGTTTCCAACACCTCTTTTCCTCCC[A/C]AAACCACTGTTCCAAGGCACATATTTTGGGAAATACTGCTGCAGGATATTAAAGAAAGAGTTCATTTCATAGAGATAGGTAATGAAGGACACTAACTCTT
3	6284252	SCAFFOLD312366_7283	0.23	0.43	AACACATTTGTAGCTGTTTTCATCTGTAAGATTGCTTCCTGACTACAGTTAAATCCTATCCTTCTTACTCTATACTCCTTTTTTTAGGCAGTATTTATCT[C/G]TCTTAGGTTTTCCTGGAAAGCTAACTAATTGTATTTACTGTTAGATTCATTATATTTGCTTATCTGAGACAGAGGGAGATTCAGAAACTACAGCAGTGGA

3	6301347	SCAFFOLD91922_9204	0.09	0.14	TACCTTCAACCTAAGAGCTTGCAGTGGTTCACCAATTTTCTGCAAAGAATTCCACTCTATCGTGAGTCCAGCTGCATGGACTTTTCTATCCTGTAATCAC[A/G]TCTTCACAGGCAGTCATGCCAACAACTGTGTGGAGCAGCTCCTGAGCTATGATTACCCTGCAATGATTATTTGTGATTTTGTCAGTCATTCCCTGAAACT
3	7703976	SCAFFOLD200186_2816	0.20	0.30	TCCTGCCACCAGGAAGCGAACCCCTCGGGCCCTGAAGACCACCCAGGACATGCTGATTTCGTCACAGCCTGTCCTAAGCAGTCTGGAGTATGGGACAGAG[A/C]TGTCATCTGGGCAGCCCCAGGTCTCGTCTTCCGCCCAACCCAGCCCAGCTGATGCTTCCCAGCCAGAGGCCACCACAGAGGTGGTGGACAGGGATGACGC
3	8237422	BES5_Contig212_565	0.38	0.37	CATTAGTTGGTAAAGAGATGTATCCCCCAGTGTCAGGAATGATCTTTTGATCTTGCCTTCCAGACCAGAAACACAAAGGCCACCACATCCTTACGTCTGG[A/G]GGCGAAGCCCTCAGCAATGCTTATGCCAACACATTCTGGGGAGAGGGCCAGCTGTGGGTCTGGTAAATCCATTGTAGACTTTAAATCTCACTTGGATGCA
3	9300478	SCAFFOLD110_7480	0.26	0.24	NNNNNNNNNNNNNNNNNNNNNNGAGCTTCATAGAAAAGAACTAGTCCTGGGAAGAGGTAGAGAAGCTGTCAGGTGTTGTTCCCAACTGTGCTACTCTGCC[A/G]TTCTTTTCAACTTTTCAAAACTTGGGTGTAGACTCAGCTCTCACTGCAATCTCTTCTGTGTGTCTTCTTTTCCCCACAGACATCAGACTGCGGGTTCGGG
3	9439439	SCAFFOLD305059_28432	0.19	0.21	TGTTTCTTTCTCTTTTTTTTATAATAAATTTATTTATATTTGGTTGTGCTGGGTCTTTGTTGCTGCATGCGGATTTCTCTAATTGCTATGGCTTGGGGCC[A/C]TCCTTCATTGTGCAGTGCACAGGTTCTGGTTACAGTGGCTTCTCTTGACGTGCAGCACAGACTCTAGAGCACAGGCTCAGTAGGTGGGGCACACGGGCTC
3	10840880	SCAFFOLD285212_13930	0.46	0.07	CAATGAATGGATCAGACCACAGTGTAGGCTGCTGTGCAAGTCCCCGACTACCCAAGAGAGTCACTAGGGAGCAGCCCTGAGGCTCCTGGTCATCCTCTTC[A/G]TCTCCACATTAAGGGGCAGCTCCAGACTGCCTCTCGGTCCTTATCCCAGCTAAGTTCTCTTACCTGTAAATGATATGACGCCATAGAGCCCAGGTCCCTA
3	12898241	SCAFFOLD166329_7952	0.46	0.46	GTGCTAGGTTGCTGCTACTTAGGATGGTTTTGTCCAAGTCAAGTGGCTATTTTTTATACAGATGAGTGTCCTAGGTCAGTTTCTAAGGAAACACACTCAG[A/G]TGCGTGCAGGAGGCTTGCTGGGGGTGCTCTCAGCAACAGCAGGATCGAGGAGAGGTGAGGCTGAACTGTGCTGCGGATTCAGTAGAGGATTTAATAAAAC
3	13491779	SCAFFOLD155427_2175	0.34	0.28	CTATCACCGGAGACGACAGGGGTAAGAGGAATACAGGGATAATTAAAACAGTCATCTCTCCCCGCGTGGAATTCAGGCTCGCAGGGAAATTTGGCTCACT[C/G]CTTTCCTGGTCTCGGCCACCTCTGCCTCTCTTTTCCCCGTTGGCATATTCCTTTTCAGTTTCTTAGCCAATTCTATTCTCTGTCCCTATGCCCACAGGAA
3	13532206	SCAFFOLD141089_7170	0.32	0.27	TCCCCTTCCCTTGGAATTCAAACAATCACCTCCCCTGTGAAGTCCTCCCTGCTTGTCAGAGCCCTCTGTACCCACTTCCCACCTCTGCTAGTGGGCGAGA[C/G]CTTGCTGCACTACCCACCCCTGTCCTGCCCAGGGAGAACTCCTTGCGTCTGGACTGTGTCACTCAGAGCGTCTGCCCCATCACCCAGCACAGGGCCTGAC
3	13542666	SCAFFOLD135166_5813	0.34	0.28	TTCACATACTTTGGGGAACAGTCCCCGGTGTTCATGGCCTAGAAGAGAAGATCTATCCATGGACTCTTGCCTTGGTCATCAGCACCAGGTCTTCCCATCA[A/G]TATTCTGTAAACTTCCAACAGTAGTTAGATGAATGATTTGGCTTTCCTGGGTTTCTCTGTTCTTGGGGCATTTGCGTTTCATTTGGCAGGCAAACTGAGT
3	15190306	SCAFFOLD155988_4390	0.36	0.37	GCAGGACATGTGGGCAGTGCTGACTTAAGAACCATTATGAGACTATTTCATTATTTTAATGACTGGTTGTTAAACAATGTGTACTGTGGAATACATACCA[A/G]GGAGGGACCGTTCCACCCCCGTGCCTGCCTCTCCTCACTCCCCAGCTCCTACACAGCATGCTGTTCACCCATCGGGCCATGGCGGAGCAGCTGGCTGGAC

3	16138425	BV105522-227-Y	0.19	0.24	CCACTCCCCTCAGACACTTCAATGATGAGTCTCCCCTGGGGCTTCGCCGGATCCTCTCACAGTCCACGGA CTCCCTCAACATGCGGAACCGGACACTGTC[A/G]GTGGAGTCCCTCATCGACGAAGGTGACCGTGCCCG GACCTGGGGGAGACCCAGGCGTGAGGGGCRGRCGGGAGCTGGCCTGGGAAAGGCAGGGTGGAAAG
3	17279419	BES9_Contig536_881	0.06	0.18	AAATCGTCAACGTATGACCTCCAAATCTAGGTGCCAAGTGCCCTGCCACAGTTGTTTACACACCTGAGG AACGTGTTCTGGAAGCTGTACTCTTGGGCTT[C/G]GACCATGTGTTGAGACAAAACAGACGTTGTGACC AACCAAGCTGATGGAGGAAAAGCACAAAATCCAATACCAACCTATAGTTCAGATCCCAAAGAGATG
3	17279806	BES9_Contig536_1268	0.43	0.18	AACAACTCAGTATTGGTCCCAGACTCTCCATTCTGCACTGACCAGAAGAGAACAACAAGAGGGAGCAG CAGGATGTCAATGGAGGAAATATATCCATGGC[A/G]AACTCAGAGACTGATGTGTTTATATTCAGAATC AGATGAGCTTCAATATGAATTTGCATCTTGCCTCTGTACTGCTTGCTATTTTTTAATGATAGGAGAA
3	18081786	SCAFFOLD130114_24383	0.13	0.14	CCCAGCGGCACTGGAGGTTAGGATGGTCCAGGGAGAGCTTGGAGGTCTGCCTTGGAGACAGCGCTCCT TGATACACAAGTGGCTGAAGAAAGGATGAATT[A/G]TCCTCCCTTTGTCTAATGAGCCTTCTAGAATATC CTGTGCTCCCTGCCCACCATTTTTGGCCTCAGTCAGATTTGATTTATTGGGAGAGGGACCTAACAC
3	18082195	SCAFFOLD130114_24792	0.47	0.10	GCATACAGTAAGAATATAATGAGTGCAGACTGAATGGGCAGTCCTGGCACCTGCCCCAGGAGGTCTGG GAGCCAAGAAGGAGGGTGGGGTGGGAACATAG[A/G]GATCCCCTTTTACCTCAGTTGAAATGTGGGAA TTGTCCACCTGCCCATCCATTCCCCTGACAGTGCAGGCAAATGTGAGTGATAGTTTACAACTCTCAGT
3	18218645	SCAFFOLD155677_3961	0.32	0.29	GGCAGTTCAGCCTCTCTGCATTTTCCTATTCCCTGCCATCATGGCCCTTTCCTCTCCTGGGAACCCGGAAC TCTGCTCCAATCCTGGGCTGTCACAAAGC[A/G]TTAAACCCCAGGCAGCACTTAACCCCATTGTCTCTCC CTACTCTTGACCATCCACAGCGCCCAGCAGAGGACAAATCATTAGGGAGGGAGAGAGCCTTGG
3	19454740	BES11_Contig277_971	0.43	0.48	CCAGGCAGGTTCCCCCGCAGAAGTGTCCTGTTCAGATTCCTCCCATCAGACGCTGCTCTGAGAGTTGTG CCCCACGACCCTCCTGGGGTACCTCCTGCCC[A/G]GATCTGAGATCACGTGCAGAGCCGTGTCCACTCCC AAGCTTCCATCCTCCACGGCGTTTGGACCAATGTCCAGAGCCATCACTGCGGCGAAGTCCACCTC
3	19455355	BES11_Contig277_1586	0.32	0.29	TTACATGTGTTCAATGCCAGGTTCCATGCCAGTCTGAGGTTTCCTATGTGCAGTGTGAAGGCCCATGCCC TGTGCAGACCTGCTATGTAGAATGTGCTCC[A/G]GTTTGTTATACAGAAACTTGTTATGTGGAATGCCCA GGCCAGAGCTATGTACCCTGCCCGGCACCTCAGCCTGTCCAGACCTATGTAACAGGTCCCCTGG
3	20291134	SCAFFOLD15001_86738	0.32	0.24	ATTGAGCAGTCCATTGACAACATCCCAGAAGCCTAGGGGAGGGGCTTGCAAAGGCTGTGATGAGAATG CTTGGGAGGTACTTGATACAGTACTTAATTTG[A/G]GGGCTCTGGAGTCAAGTAGAACTGAGCTCAAAT CCTGCCTGTTATTGTCAACTTTATAACTTATTGATAAGAATAGGTACCACCTATTGCCCAATTTCTA
3	21525127	BES5_Contig554_1101	0.16	0.24	GAATCATCCTAGTTCCTCTCAAAAATCCCTTTCCTGTTGGCATCTGTCTCCCCTATTATGGGGTGAAAATA GCTGGTATTTGGTACATTTTCAGTAAATA[A/C]TGTTTCTTAACATGTTTGAATATGGCATTTATGCCGTG AGGTCAGCCATTCATAAATATTCATTAAAGTTAGACTTGTTCCAGAGAACAATCATTGCCAT
3	22224517	SCAFFOLD51350_7097	0.38	0.46	CACAGGGTCGGACACGACTGAAGCAACTTAGCAGCAGCAGCAGCAGGGGAAGGGAAGGGATATGGA GGGAAGCTGGACTTCTCAAGCATACTGTTACTGT[A/G]GAACCATGTATCTTAAATGGTAGCAAAGTAA TTGCAATGGGACTGTAGGAACTCAGTAATCCTTCATTTTCTTTTGTTCTTTATACTGCCAAATATCTTG

3	22958652	SCAFFOLD155264_15493	0.31	0.35	GGGAAAAAAGAGACCTATTTCCGCCTGGTTTCGAACCGGAGACTTTTCACATGTGAGGCAAACGTGATAACCACTACACCACGGAAAGGCGACAGTCGCT[A/G]CCACTGAGCCCTGTATCCAGGAAAACCCAAGGTTCAGCCGCCACTATCCAAGCCACCAACTTTTAACATTCATGCAGAAACCTTGGAGCCCCACGCTCTC
3	22958709	SCAFFOLD155264_15436	0.30	0.27	TGGCGGCTGAACCTTGGGTTTTCCTGGATACAGGGCTCAGTGGCAGCGACTGTCGCCTTTCCGTGGTGTAGTGGTTATCACGTTTGCCTCACATGTGAAA[A/G]GTCTCCGGTTCGAAACCAGGCGGAAATAGGTCTCTTTTTTCCCCCTGTGATTTGCATTCTCATTGGCAAAAGGAAAAGGCGCGAAAGGCACGGGGTGAGG
3	23786086	BES2_Contig311_734	0.15	0.27	AGAAGCACACCATTTCTCAAGTGAACAGACAAATTCCTCCCAAGGGTCCTCATAAAGAGGATAGAGTAATATAATGAACATCTTTTCAGTAGTCACAGCA[A/G]TGAGTTTTGTGGGATTGTTTCTTATTGTTCCCTTGACACAGAATGATAGCATCTCACCAAAGCACATTTCCATGAAGACAATACTATACAGGGATCAATA
3	24394895	BES7_Contig442_725	0.49	0.47	ACGTGGCAGGTAATATTAAATATGGTAGAAGTCAAGAATCATTAACTTGTGACTTGTTGGGTCTATAACTATAAGGCAAAGAAAAAGTCTGGGTCATCCT[A/G]AACATAAACCATGAGGTTCTTCATGGCAACATGATGTGGAGGACAGGGGAAGAGATAAGGATGTAGGTTCAGGGGAGAGCTACTCCATTTATCAACTGTA
3	25585193	SCAFFOLD298038_3437	0.11	0.23	CCCGGAAGCCCCAGTGTCCCTCATTCTTTACTGGGTCCTCTCCAGTTCCTTTCTGCTGTGACCTGAGGCACAGACTAGCTTGAGGATCAGGTTGGCACCC[A/G]AATGCCTTGGCTCTTCCTGGAGCCTCCTGCTCGTCTTCTAGAGAAGATAGATGCCCCTTTTGACTCGGCAGACCTCTGGTGAGCAGCGCTCTGGCCTGCG
3	26589133	BES11_Contig221_1196	0.31	0.44	GAGATTGTAAATGCTAGCTTCCTATTTTTCTCTCTCCCTTGGAGAAATAGTTTTACTTTCCTTTCTCTGCTCTAGGACAGGCCTGTTTTTCATTGCAGCC[A/G]TTGTCTTCAACCCCACTGATAGAGGAGGAAGGTGAAGTGATGAAAGTCAACATCTACCTAGGGACCTCTTGTGGTTTAGTCAATTAATTGTTGGTCCTTC
3	27536162	SCAFFOLD173435_9502	0.19	0.14	GGCCCGAGCTTCCACATCTAGCCCTCACATGCACCAGTGGACACACGTCTCGGGCTGGGAGTGTAGAGGGGTGGCACAGACTGTCTGCAGGCCTCTCTCT[A/G]TTCTAGCTGCTGCAAACCAGTTGCTGTATTCTCCTCTGAGGCTCTGAAGCTCTCCCTCTGTCCTCGCTGACTTCCTCCTAGTGAGGGGACTTCCCAGGGT
3	31885700	SCAFFOLD221183_465	0.48	0.00	TGCTGGTTGATCCAGTGCGCCAGCCCTACCGAGTGAAGGTCATTGACTTTGGTTCTGCTAGTCACGTTTCCAAAGCTGTGTGCTCAACCTACTTACAGTC[A/G]CGTTACTACAGGCAAGTGGCAGATGCTAAACAATATGTCTTAAACTAGAGATCTGTCTTTATATTTAGCATGTACTACAAAGGGCTACATATAACAATGA
3	34095512	BES9_Contig264_1036	0.14	0.20	GCCAAAGGCTAACATGTTCTAGGGTGACCCTAAAATTTGGTTCATCCAGATTTTTCTGGTCCCTCCTCCCTTCTGTGGAGAAATGAAGGAGATGGAGAGA[A/G]GGAGAGCCACCAGCTTTACGCTCAGTCTCTCATCTTTTCTTCCTAGGGCACCCCAGAAGAGGAGAACATGGGCAAGACAACCTCACTCATCGAGAGCCAG
3	34818380	SCAFFOLD10041_32940	0.04	0.02	TGTTCACAGCTTTCAAGAATTAGTGTACTTTTTTAGGTAGTTTATGCCAGTGGTTCCTAATCCTAGTTGTATCTCAGGACTGGAATCCTGGGGAGCTTAT[A/G]AAAATATGAGGCTCCATCTTTAACCTACAAAATCATCATTAAAGATAGGACCTGGGAACCTATATTTTTAAAACTTCTTCAGCTGATTCTGACATCTAAC
3	34818537	SCAFFOLD10041_33097	0.04	0.02	GGTGCTAGAATCTTTTTCAGCATAATGGAAATCTGGAAGACATCTAGGTATTTGGTGTGTGCTTCCTTCAACTCTAGATAACCATCAACATGAGATCAAG[A/G]TATGTTTGTAGTAAACCTCAGTTGGTGCTGATTCTTATGAGCATATTATATACCTGGTTAGATGTCAGAATCAGCTGAAGAAGTTTTAAAAATATAGGTT

3	37210375	BES2_Contig295_840	0.48	0.02	GATACTATGGTAACATTTAATTCTCATCATGACACTATGAAGTAGGTTCTGTTACTATCCCCGTTTTATTGCTGAAGAATTTGAGGTTTATAGCAATGAC[A/G]TAATTGCTCTGGGTCACCCGGCAGATAAGTGTCAGAGTGGAATTCAACCTTGGCATCAGGGCCTCAGCTCTTGACATAACCCTGGGTGGCCTCCATGATG
3	37210499	BES2_Contig295_716	0.32	0.39	GATAAGTGTCAGAGTGGAATTCAACCTTGGCATCAGGGCCTCAGCTCTTGACATAACCCTGGGTGGCCTCCATGATGACCAGAACACATTGGGTCATCCT[A/G]CATAATGGTGTCGTTGGTTAATATCAACAGTAATGTTTTCTTATCTTTTCATTGTGCAGATGGAGCCCACAGTACATATCCACCAAGAACATCTCACAGG
3	38503084	SCAFFOLD41750_8551	0.46	0.08	ACAGACCCTAAATGACCAACTTCCTACTTTATATTCATTTCACTTGAAGATAATTTTATAGAACTTTCAAGGCAAGAAAAAGAGGAAAAAACAACAACAA[A/C]GAAAAGGCTGTGAAAATGGATCCCTTCAATGCAAAGACTGAACAGAGAACATTTTTATAGCTCAATGGCAACTCATTGTTTGAAAGAAGTTATAATGAAA
3	38548916	BES10_Contig649_1524	0.19	0.21	AACTCTCCTGGACCTACCTCTTCTTTGTTTCTGTTCATTTCTTCAACAAATTCATATTGAGGCCTCACTGTGTGAAATCGCTGTGATTCTGCAGAATTCT[A/G]AAAGGACTCAGTTCTGAAAAAATTCTCAAATTAGTAAATCTATCTGTACCAAAGCCTCAAATAGATCTATACTTGGACCCTAAACTAGGAAATGCCACAC
3	38549270	BES10_Contig649_1170	0.11	0.13	GATAACCCAGTTACCCTTATATCTTGGTCTAGGAATTGAGTTATTTCTTAAGTATAGTTCAGTTTCCTCCCGTGGTGACTGGTAACATTATATCACCTAC[A/G]TAATATTCATTTATCTAATGACACTCTAGGATTAAATTTAATTGGGAAAGGATGTAAAATGCAAACAAAATCCAGAATTTGTTTTAAATCAGCCAGTTTC
3	38549365	BES10_Contig649_1075	0.03	0.09	GACTAGAAAAACAAAGATTTCGATTAACCAAGCCACTGAAACCAGAGGGGGTGAAAAAAGGTTAGGTTATAACAACCTGAGGTCAGTCTTCTTGAGAAAC[A/T]GGCTGATTTAAAACAAATTCTGGATTTTGTTTGCATTTTACATCCTTTCCCAATTAAATTTAATCCTAGAGTGTCATTAGATAAATGAATATTACGTAGG
3	39359021	SCAFFOLD181993_13526	0.26	0.16	TTTGGCCAGCAAGGATGCCAAGCAGGATAATGAATGTTGAGGGCAGTAAAGGAGTAATCCTGTGTGTTCATATTTAAAAGAAATACACTCAAACAGCTGC[A/C]AGAGATGGGCTGCAATTCATTTTCTCCTGTGGTTTAATATTCTTTGGAAACTGGAAACACAGTTTCTCTCTGCAGAATGGTGTGCTCAGGGTAATTGTCA
3	39359212	SCAFFOLD181993_13335	0.25	0.13	TTTAAAACAAACCAACAGCCTTTCAGGTTCCCTTGGCTGATATTCTTAAACAATTCACTATACTGATCCTTTGCAAGAAGAGGTCAATTAAACTTTGTGG[A/G]TAGGGAGAAGGATTGTTTTTTGTCTTGCTATCTATTACTATTTCACTCTGACCAAAGGATGTAATTTTTCTCTAAATTTGTCATTTCCATTGACAATTAC
3	39710161	SCAFFOLD185349_3515	0.30	0.24	AGCCTTGAAAACAGAAAAATATACCTCTTGGAGCTGTAGCCACTGGGGAGATGTCTAAACAGATCATTTCAGTGACAGGCACTGACGACTATGATACAGA[A/G]GAGTGTGAGATGTTATAAGGGCACAGATACGGAACATTTTACCCAAATCTAGAGGAGACAACATTTGAGCTCTGCCTTTTAGGACAGGTAAAAATTAGCC
3	40201985	SCAFFOLD120581_8167	0.32	0.47	ATTTTATTATTTTGAATGAATTTTGAAGGTTAAACATTTCAATGAAAATTAAACAACTGGATCTGCACACAGCGAAGCTATTGCCCGAGGTATCTGGTGT[A/G]CTGTTACAAGGAAGTTTTATGATATGTATTGTTTATCTCAACCTACATCTTACCATATCTTAGATGTCTGCTAACTAAGATTATTTAACTTAGTCTACAA
3	41822142	SCAFFOLD170701_6439	0.32	0.44	CTAGACTGCTTGAAATAATCTCATATGTGCTACCCTAATCTTAAGATAAAATGCTGCTGGGAATGTCTAAATTTATTGCTAGTAAAGCTTATTAACTCCA[A/G]AGTCACCATGTGCAGTTCAGTTTGGATAATCATCTCATATTTCACAGTTATACACTTTAGCTTATATTATTGTCCAGGAACAAGAAAATAACAATTTTTA

3	42496623	SCAFFOLD175618_10344	0.50	0.33	TGCTGAATCTTTTTTCAAATTCATAAAGAAAGTCTCTCAGGATATTTCACAGTGTGAGTATACACAGCCTCCTTTCCCTGACAGGTCATTATTGAATATC[A/G]TGCTGGGTTTGAGTACAGGGCTGGTGAGTGGCTTCTCTCCCTGCTTCTTCTGTAAATGTATGAGGGGTTCAAGCTTTGGAATCTTAAATGTCCATGAGAG
3	42496799	SCAFFOLD175618_10520	0.49	0.32	GCCTTACCCAGTAATGAGTCATAAAAAACTGCAGCAGGTGGTCATAGCCACATGATGGTCTAACTCATACACTGTGACTTTTGCCAAGGACTCTGACTGC[A/G]ATCCTGCTGAACTCAACTGCAATTCTTGTGTCAGCTCTGCCTGTAGTGCAAATCAATCACCAGGTACCTTTTGAGCTCTCATGGACATTTAAGATTCCAA
3	43502277	SCAFFOLD112707_2297	0.47	0.34	AAATAATAATGAAAAATGTGGTAAAAAATGATAAGTATGATCATGGAAAAATGCCTGATTTCATTCTGGATGCAGAATTTGTTCAAACTGGAAAACAAGA[A/G]AGGAAACAAGAAATAAACATCTTTGTAAGTCAGGGACAAACTGAAAAATGGCATTCAGCAGAGTGATAGTTTGTATTACAAATTAAATACCCAACTAAAA
3	43712628	BES5_Contig186_572	0.08	0.12	AACAAAAGAAATCAAACAATATTAAGGATAGAGAAAAATAGGAAATCTAAAGTTCAGTGTGTTTAATCTTGACTTTCAAAATGAAACAGCTGTTCCCATG[A/G]AAGTCTTAAAATGAAATATTAAAGATAATAAAATTGCAATGATTTTGCTGTAGTGGTTATGATACACTTTCCTTTAAATACAAATATGCTGCTATTCCAA
3	44433271	SCAFFOLD125836_5930	0.49	0.42	CTTACAGCTGGGAGGATTGAATTTTGATGTTTATGTGTCTTATGCAGTGTCCTCTGGCTAGTGACTAGTGGAACCAGCAGTTGAACTTGACAGGGCTTTT[A/G]GCTGAATCCTCTACCTTCTGACTTACATGACCGTGTGTGGCATAAAGGTAAATGTGGTAGTGGAAGTGGGCAGGATTGACATAGCTCAGTAGAGTGCAAA
3	44433532	SCAFFOLD125836_5669	0.49	0.42	TCCAGAATTTCTCAAGCTTGGCTCTATTGACTGTTTGGGCCAGATAATTCTTTGTTGGCATGTAAGATGTTTAGCAGCACCCCTTGTCTCTGTCTACTAG[A/G]TCCACAGTCCTTTCTCCCTTCTCAGTTGCTATAACCAAAAATATTTCCATACATTTTAAAACATACTCCAGGGATCATATTTAGCTTCATTTGAAAACTA
3	44433662	SCAFFOLD125836_5539	0.48	0.43	AACATCTTACATGCCAACAAAGAATTATCTGGCCCAAACAGTCAATAGAGCCAAGCTTGAGAAATTCTGGATGAGATGCTTTGTAAATTAAAAGCACAAC[A/G]AATATTGATTGTGTATCAGGCCCTGTCTTAGAAACCAGGTAGTGTTCTCAAGTTAAACATAAATGCACAGAGATGAGACGCTAAGGTTCAACATACTTCC
3	44433721	SCAFFOLD125836_5480	0.49	0.42	AGAAATTCTGGATGAGATGCTTTGTAAATTAAAAGCACAACAAATATTGATTGTGTATCAGGCCCTGTCTTAGAAACCAGGTAGTGTTCTCAAGTTAAAC[A/C]TAAATGCACAGAGATGAGACGCTAAGGTTCAACATACTTCCATCAGCCCAAAGTATTCAGTTTATTTCTTTGACTTTCCAATGCTGTGTTCAGTGCCTCC
3	44433777	SCAFFOLD125836_5424	0.50	0.42	TGTCAGTTAAGTTCGCAAGCAAAACATGTATAGCAGATATTGCTACTCAATATATTGGAGGCACTGAACACAGCATTGGAAAGTCAAAGAAATAAACTGA[A/G]TACTTTGGGCTGATGGAAGTATGTTGAACCTTAGCGTCTCATCTCTGTGCATTTATGTTTAACTTGAGAACACTACCTGGTTTCTAAGACAGGGCCTGAT
3	45931307	SCAFFOLD100165_19223	0.40	0.40	TTACTTCCTCCCGTCTAATCTTAGAATTGAGATTGGCAAACTACGGTATCTGGGTTTGCCGAATGCTTTTGTGAATAGAATTTCATTGGAAGGCAACTAC[A/G]CCCACTTGTTCATGTATTACCTATGGCTTTTTCTGGAGTTTGAAGGTAGAGTTGAGTAGTTACCACAGAGACTCTGTGACACAAAAAACCCTAAAGTATT
3	45931389	SCAFFOLD100165_19305	0.40	0.40	AATACATGAACAAGTGGGCGTAGTTGCCTTCCAATGAAATTCTATTCACAAAAGCATTCGGCAAACCCAGATACCGTAGTTTGCCAATCTCAATTCTAAG[A/G]TTAGACGGGAGGAAGTAAGAATAAGAGCAAATGTACTTACATTTATTAATTGGGTTGGCAAGTGGTAAAAATGGTATACTTTTCTAATTACTCCTATTTT

3	46423377	BES11_Contig424_618	0.28	0.33	GCAGGTATCACTCATAGATTACTCTTCATCTAATTTTATTTTCATACAATGTACTATGTGATGCTTTCTATAACATAAAACCTACATTGAAAAGTTAGTC[A/C]AAGTACCTCTGTTCTCCAGTTCTGGTTTCATATTACAGTTTTTACATCTACCCTCAGACAGCCTATCAACTTAACTCTTGAGAATTAAATAATGTGTGTG
3	47786227	BES4_Contig306_857	0.31	0.27	ATTATCTTTGACACTAGCCTAGACAGATGCATAACGGGGGCTGAAATTAAACAATAGTGATGATATTTGGAGCTCATCCAAAATGCTCTCTATTGTTTAC[A/G]TAAGTGGCTCCAACATCATTATTTGATCCATAATTTCTCATTTTTTATTACACATATGTTCTTGCATAGAACAGCACACAACATCAACTCAGTGGGAGGC
3	49462205	BES9_Contig508_1511	0.05	0.08	GTCCATTTCAGAATCCAACAGAAATTGCATCACTAATAAGGCAAATGAGGTTTTTAAACAGTTGCTTCTCATCTCCGCACAAGAGGGCAGCCTTTCACCA[A/G]AACCAAGGAAACAGTTGGGCAGAACTTTGTGTTAATTTGTATAGAGACCCCTCCAATCATAGAACTTCCAAAGAGAAGACCTCTCTTGCTATAAAGAATG
3	50647464	BES3_Contig390_1348	0.42	0.38	ACCACCACCTGAGACTGTATGTGCAGGTAATTTCTAATTTGAATAGATACCAGTGTAAAGAAGCATAATCATATTGTGTTAATGCAAATGCCCATCAAAA[A/G]CACGGCGTGAAGATAAATTAAAATATAATTCTTCAATAATGCATTTGTCCATAGGAAGATTTTGGACAGATTAAAAACAAACACCTTGATATCACTTAGA
3	52135762	SCAFFOLD270227_6558	0.08	0.12	GTGTTTATGGATAAACTTTTTTTTAATGTAATCATTCCTAAGGGTCACAAGCTCACAGAATATTTAGAAGTTTATGAAAACAAGAATGAACAGTTACTAC[A/C]AATTGAGCTACTTACTCTCTAGGTAGGGTGTGAACTCCGGGGAGTTGGTAAACGGAGGCTCCACTGTGGAACTGCTAGTGTCTGCGGGGTAGGAAAGGAC
3	52705546	SCAFFOLD285049_14599	0.50	0.00	CAGGCGAGACAGAGGGCCAGGGGGGCCCTGAGTGCTGGGAATCATTAAGGCAGGGGGTGATATGGAGCTGTTTGAAGAGAAAGATTTCCCTCACCCGACA[C/G]AGGTTTTTCTCCCTTTGACAAATTTAGCTGAAGGAATCTGACAGCAAACCACAAGCTTGCTTTGTCAGGCTGAGCAGGCAAGAATCATGTGAAATATTTT
3	56111549	SCAFFOLD100853_6992	0.47	0.32	AATATCTTTGTAAAACAAAAGATAAAACCTAGAAGCAATTTTTTAAAGAAATTATTTACATAAATAAATTTTGATTACATAAAGGAATTGCTTACATAAA[A/G]ATATAAAATTTCTGCACAGAAAAAAAAAGAGTAAAAAAATTCAATTACTTCAAAAACATATTCAATATAAGTTCAGTTCAGTTCAGTAGCTCAGTCATGT
3	56699326	SCAFFOLD125560_2098	0.30	0.18	GAGCACGAGTGTGTATGTCACTAGAGTGGTTGTAGGACCTTCAAAACCTCAGGTTCTTGGGAGAATGATATATAATTAATTATAAGCAGTCAACTATCAC[A/G]CTTCCTCTCTCTGGTTGCCATGGTGAATGAAAGGTACGGTTGCCTTTCTCCTTAAGGGCCTGATTCGGAAATTGCATGTTCCACTTTCATTCCCACCTTC
3	61155135	BV105482-180-R	0.16	0.18	ACTGCCTAGTTTGTATTTGAGCAGCAGAGAAAGGGGTGTTTCTCAAAGATAGCAAAAAAATTATAATGAAAGCCATGCCAATTCATTCACCTAATTCTGT[A/G]TAGTCTCAGCTTACATACTGTTTACCTAGCCACCTTTTATCTGTTGTTGGTTACTACTAATTTTAGCTTTTGTGTTTAATTACTTGACTGTTTTGGGGGC
3	61444602	SCAFFOLD155534_16941	0.48	0.48	TGTTCTACTTTAGTTTTTCTTTTTTGTTACAGAACATATTTTTTAAAAGTGCTATCATGTGCCTTGAAGGTTGTAGCAGAAGGCGAAATAATTCATAATT[A/C]TTTTAGCTAGGTTGGTGACCACTAGATAGTAGGCTGCTTTAAAGTCTGTGTTTTATTTACTTGTTTGTATATTGACCGACTGACTGACTTCAGTTTCTGG
3	64244295	BES10_Contig692_2496	0.14	0.16	CTTAAATGACCTCCACAAGTTTTAGAAGATACAGGGGTTATTTGAATTATTATGATTCATGAACAATTAGATCACATTTATAATCAGCAATGCAAAATAT[A/C]TTTTAGTGTATAGTCCTATTTTAATGATGCTTCATCAAGTTGAAAATAATTAGTGTTAAATAGTTATTTGTTCCGAATGATTAAAGACACATCAAGTTAA

3	66019235	SCAFFOLD313448_2068	0.48	0.49	CCATATTTTTAGCATTGAGACTGTTTCATTAAAGGAAATAGCAAAGTCTACATTGTTCAGGGAGTTCCACTGACGTTTCTATCAAATAAAATGCAATGTC[A/G]AGAGTTCCAACAATTTTACTGATAAAATAACTAGGATAGAAAGTGAGCAATTAGCATTATCAAAATTCAGAAAAGAGATTCTGATCTGACAGTGTTTTCT
3	66424573	BES3_Contig73_181	0.43	0.43	CGCAAAAAAGAAAGAAAGACATTGAGGTTGTAATTCAAGCTTATTGGAAGTGTTTTCTAGTAGACTGGAAACAATCTTCTTGACTGAATGAAGCTAGGCT[A/G]AAATTACAGCTTTCCCCATAAACACCTCAGCTACTATGTTCAGCTCCTACCTTATAATGACTGGATTAAAGTGTTTTTCTTATGTGATTGTATGTCTTCT
3	67032214	SCAFFOLD372341_190	0.49	0.31	GTCTAAAATTTTCCATTAAAATCTTTCATTAAAATCAATAAATACAAAAAATTGAAATAAAATATTGATATTTATGATTAATATAAAAAGCAGTCTTCTT[A/C]AACTATGGTCATGGGAGAACTTCTCAATCATTCCAGAATGTCAGATATCCATAATCATAAGCCATATATTTCTTTAATATTAAAAATATCTTTTAAAAAT
3	67759111	SCAFFOLD141203_6497	0.49	0.42	TGATATTTGATAAAAGCAAAGTAATTTTGCAAATGTTATAGAGATCTCAAACCACTGACTTTTAGTTAATCACAATGGAAATTATCTTGGGTGGGCCTTA[A/C]CTAATCAGGTGAAACCTTAAGTGGAGCTGGACCTTCCTGAAGCTGGGTTCAAGGCATGAGAGAGTCTCCTTCTGGCTTTGAAGAAGCAGAAAGCCATGCT
3	67814801	BES11_Contig312_1237	0.47	0.28	TCTCTGTAGAACTTCCATCACTACTGTTTCCTCCAGACCAAATTAAAGAAGAGTAAATTCAATTTGAGTGAGAAACGTTGCCCAAATTCTCACTACTGCC[A/G]GCAAAAGTAGCAAGGAACAAACCTTTCCATTTGATTAGTAGTTCTCATGTTTCAACAAAACATTTCATGGAGCATTGCTTTATTGGTACAGGAGGAATGA
3	68058861	SCAFFOLD36250_11008	0.06	0.09	AAAAGCTTTTGAATGAAAACAGATTTTTCTTATGTCTGAGGTTCCTGGAGATTCTAAATTTCTCCTCTAGACCTCACTCGAGCTTTAAGGGTATGTTAAC[A/G]TTTTAGAAGTTTTCTTATTATCCACTTTTTTGGTGGTTGCCATTTGCCCCACGTTCCATCCATCATAGGTAAAAGAGTCCAGGCATCCTGTCTCTTCAGA
3	68644513	BES2_Contig447_1495	0.47	0.05	ATCAACTCAGCTAGGAAAGATTTTAGGGGTGCAAATCTAGTTATGTCCTTTTTGATTCTTTAGTAGTTCTTGACCAATACCATTTTCAAGTTTCTGATAG[A/G]AAGTTTTCATATTTTGCTTACTTCTTCTATTAGTGAGGCAGCACACATATATAATGAAAATGAATTTCTAGCAGAAAGAATGTTTCTTTTTCATTTTTTT
3	69538463	BES4_Contig290_1439	0.22	0.35	GTAATGATCCTTCTCAGTGGAACCCAGCAAATGCTATTGCCATTTCTCATAGGCCAGACTAGGCTTGCAAACATGCTGCCACCACTTAGTGAAAACAAAG[A/G]GGGTAGAACAGAAAGAGGAGATCATGCGTATCTTAAAATAACAACAGCTGTATGACAGTTTCAGGCTGTGTTTGGAAAATTTGCATATATCCCATTAAGT
3	72437474	SCAFFOLD234818_2292	0.11	0.23	TACTTTGACACAGACTTTTTAATCTTCCTTTTTTTCTTCCACAAAATCACCTCAGGTACCACAAATCCACTTTATTGGAAACCATAATCTTGTACTTAAA[A/G]AACAACTCAGTGTTAACCCCAGGCCCTCTGGACACCTTTAATCAGGTTTGGGTGAGTCCTGAAATCCTAGAATGCCTGAGTCAGGGCTTGAGTCAGTAAG
3	72583415	SCAFFOLD110844_5365	0.07	0.15	AGAAGAAACAAGATATTTTGATTTTTTTGTGGGACTCAGATCCCCTGCCTATAAACTCAGGGAGAGGCTGAAAGTATCTTCAAGAGCCTAATATCTTGAC[A/G]GTCTGTATTTTCCCCCAGTTTTTAGGAATAGAGTGTTAGAAGTTTTACTGTTAAGGTGCATAACATTTTATTTCTCATTGTTTTCTCTATAATATAGTGA
3	72583513	SCAFFOLD110844_5267	0.47	0.41	ATAGATTTATGAAGACACTCTACGTGTCAAATTATTCAAAGTTCCCTTAGAGATCCTGAAAGTGATGGAGAAAATGTTATGGGAACACTTCAGGAAACAG[A/G]AGAAACAAGATATTTTGATTTTTTTGTGGGACTCAGATCCCCTGCCTATAAACTCAGGGAGAGGCTGAAAGTATCTTCAAGAGCCTAATATCTTGACGGT

3	72584838	SCAFFOLD110844_4329	0.20	0.22	GATTGCAGAGGATTTCTCAGGCTTATCCTTCTGTTTGAAACTCCCCCTAATTGGCTGCAAAGCTATGAGAGTCCAGAAATAGAGCCCATGGAGTCTGCTG[A/C]AGTGTTTTTACCCAATTTAGTATAGAGGGAGGGCCTTGAATTCACAGTCCTGGACCTGAATCTTGATATAATCTTAGCCCAACTGAATAACCTCTCTGAG
3	72584896	SCAFFOLD110844_4271	0.34	0.36	AAAGCTATGAGAGTCCAGAAATAGAGCCCATGGAGTCTGCTGAAGTGTTTTTACCCAATTTAGTATAGAGGGAGGGCCTTGAATTCACAGTCCTGGACCT[A/G]AATCTTGATATAATCTTAGCCCAACTGAATAACCTCTCTGAGTGTTACTCTAGAAAACTGAATGTAGGTATTTACCCACTGGGGTGGATAAAGGGTTAAG
3	74378174	BES4_Contig160_1015	0.42	0.34	TTTGGAAACACTCTCAGCTATAGACTTTAAGAAAACATGTGACATTTACACCTTGCTCTTCAGTTGGAACCACAGTCTATAAAGTATCCTAGTTCACAGA[A/G]GCTACTGTCATCAACTGCCTGATACCTCAGATGGTACCAGGTTCTTGCTTTTCAGGAATAGGTACTGAAATTTCACATTACTTAAAAGCATCATTTTCCA
3	74519063	BES3_Contig399_3199	0.02	0.05	TGTGAAGGTAATAAAATATGAAATCATCCAACACTCCTGGTTCCAAGTGAATTTTGTTTAGTTCTAAAATGAGCAATAAATAACTTCAGCAACAACCAAA[A/G]GATCCTTGAGAATGGATAGCTGAAAAAGGGAGAGAAGAAGAGAATCCTGAGGAAAAACTGTATCATTTTTACCAAAAGCAAAATTGCTACCTTTCTGGTG
3	74685628	AF440380-143	0.05	0.10	AACAAGTGTTTTTATATAGCAAAATTTAACTTAATAGATTAGAAATTTATTTTCACATCTGTGTGGTTTGTTTACCAGTTTAGTTCAATGAAGACTCTGA[A/G]CAATAGAGAAGATAGAAATAGTTTTACAGTAAGTAGGAGAATGAGGTCRCAACAGAAACAAGTAAGATCATACTAAAAATGCTAATAAGAGTTGAAACCC
3	75168356	BES7_Contig350_1198	0.23	0.24	ATCCATGTTTCGCCAAGCTGTTGTAGCTTTCTCAAAGCTTTCAAAATTATCAAACATCAACATACATCTCCAACACTATTACTTTATCAGGAAAAGACAC[A/T]GCAAACAGCATCTCAACTTTCAAAATTCTTTTTCATGTCATTAAGCAGCTGCAGCCAAGCTGCAATGCTTCCTCTCAAGCTATAACAAAGCAATGTTGGC
3	75607987	SCAFFOLD133004_791	0.38	0.47	TATGTATGCACCCTGGTTTGCCATGGATAGTCCCACTTTATGCCTACCATCCTAGTGAAGGTTAGCTTCAGAAGTGACGTAGTTTGGAAAAAAATATATG[A/G]TCACCCTCGCTACATGGATACGGCCCAATGATAGACAAGAAGGAGGCGAGTAACACTGTATGATCTTCATTGTTTAAGTTAAATACAGATCTTAGTACAA
3	76820624	SCAFFOLD265587_17486	0.06	0.03	TTGAATTAGATAATATTAAATTGAGATTATTTGAAAAATTCATTAATTCATCTGAATATTCTTTGTGTTCTCTGCTGCATTCTTGTATTTTCATATCATC[C/G]CTTGTGCCAACTTTTGCTCTGTTAATAATCCATTGTTACTAACAAACCCTACATATGACCTTTCCTCCTGTTCTCTTCATTTTCTTTAAGCAGATCATTG
3	76997459	SCAFFOLD100560_8711	0.25	0.33	TTGGTGATGGACAGAGAGGCCTGGTGTGCTGTGATTCATGGGGTCGCAAGGAGTCAGACATGACTGAGCAATTGATCTTAACTGATGGTCATATTAAAGT[A/G]AAGTAAAGTAGTCGTTCATTTGTGTCCGACTCTTTGCAACCCATGGACTGTAGCCTACCAGGCTCCTCTGTCCATGGGATTTCCCAGCCAAGAGTACTGG
3	79047206	SCAFFOLD136424_1392	0.38	0.50	GTGGTAAAATTAAGAAACTGTGGACAAGAAAAGAAATACTCAATTTCAGTCGAAATGATCTGAAAACACATTTAACTAGTGTCTAGAAAAGACAAGTAAA[A/G]GTTTCTATATCCAGCCCTATGAGTAATCGACAGGAAGAATTGTGGTCAGGGCACCATTAAAGGCTCATGGAAAACAAACTAAAAGAGAAAGAAACCATCT
3	79051740	SCAFFOLD250354_1799	0.05	0.13	GAAATTTAATACCCATTTTTCCTGTTCTATCTAATTTGACAAAGAACTCCAGACAATGTAGAGATAAATGTTAGCCTCTTACTTCCGGTCTATTCTTCAA[A/G]CGTCCAGACACTTCAGCTTAGTCATTTCCTCTAAAAGAAATTAAATCTTACCATGGGTTTCAAAAATGGTGCTATTGAATATTTAAAAGACATAAAACAT

3	79052212	SCAFFOLD250354_2271	0.06	0.13	TTGCAGCACGCCCTTTCAGCATTGCTTTTTTTTGCTTCAAGCAGAAATCTTAGATTATGTTCCAGTTTTGGGTTATTTTTAGGGAAACCATACATTTTCT[A/G]ACTTTTGAAAAATGCAAGCACTCAGAGTGCTTTGCACACAGTATGCATGGAATATACATCTTTTGAACATTAGGTAAATCAAACTCTCCGGCGTATTTTG
3	79725577	SCAFFOLD146034_1976	0.11	0.16	CCTTTTAAATTAATTCTTCCCCAGATGCCTACTGAATACACACAAAAAAAGGTAGTTACTATAAAGGAGTATAAACAAATTTCAAGTTTCTTAAAACTGG[A/G]AAAACAATAAAGGAAAAAGCAAGTTAAACTCTGAATGAGAAACAGTAAGTTTGCAAAACAAATGCTCTAAAGAACAAAGAACATTTTATGTAGGTCTGAT
3	82029709	SCAFFOLD53628_4616	0.22	0.22	TTGCATGACTTCCCTCCCATTAGACAGGATAGCCACCCTTCCTTTTATCTGAGCTAAAGAATTGCTACCTGAAAGCATTTATCTGATGATAACACTGCAC[A/G]GTAAAGCAAAGATGACTCCAAAGAATAGATCTGTCTGCCACCATTTCAGCTTGTCCCTTTGTTTCAGCTATGTATCACTCCAACTCCTCTTCATAGTTAA
3	82395579	BES4_Contig245_660	0.44	0.42	ACCTCCTCAACCATATCCCAGACTAGACAGCATACGTTGCCTAGGGTTTCTCATGTGGAGAGCCAATTAAAATTTGATTTTGTACTTTTGTGAAGAGGAA[A/G]AAGTACCAAGGTCTTTAGAAGGCATATAAAGGCTTTCTCTGACCTCCAAAATAAGCTTTCATAGCTAAATTTTTGCTTGACCTGATCTGTTTCTCTTCTA
3	83039709	SCAFFOLD240107_8874	0.21	0.15	TGCTTGTGTGTGGGACAGGTCCCTGACTCTAATAGCCTAGAAGGAGGATTCCAAAAGGGCTCTTGCCAGTGCCAATATCCCCCAAATGGCTGCTGCCTGC[A/G]TTCTGTCCTCAGGGTGAATCCCATCTGCCTCTAGCCTCTCCAAGTGGCTAAGATCAGCAAGTGGTTCTATCCAAGAACCTTTCAAAGCACTGGCTCTGTG
3	83367481	BES9_Contig361_813	0.20	0.13	AAATTCTGTGAAGCTTCATTTCTTCTTTCAACTCATAATTACTGAGAATTCATTATGTATTAGAAACTGTCCCAGATCCCAGTAACACAGACATAACACT[A/G]TCTTTTCAAGTTGTTTGCAGCCCAAATCCTTTCCTCTTATCCTCAACCCCTGAAATGTGACTGCAAAGAATATTTACACAAAGACAAAACTCTCCCATAC
3	84018286	BES11_Contig186_601	0.36	0.29	TTTACGTAGGCTTCATTATGTTGACATGATTGACTCAATCAATGGCCACAGATTTAACCTCCAGCTCCTCTCCCCTAGCCTCTCTTCTCTTCACTTGGAA[A/G]GAGCAGATGTATGGAGGCTGAAAGTTCCAATATAAATCTCTCAGTTGGTTCCCCTGGCAACCAGCTCCCAGCCTTAGGGAGTTTCCAAAAGCCATCTCAT
3	84435499	SCAFFOLD148432_3242	0.41	0.37	TGTTCACCATCTGACAAATACATTATGTTCCCCTGAAAAATTTTAAATTCTGAAGGCACCATGGTACCCTGGGGCAACTTGGCTCATATTTTGGGAACTG[A/C]GGCTCTAGCACATAGCAGTTACTCAGGAGAAATCCAAAGTAGATGAATTATCTCTTTCAGGTGAATACTTCTATGATTTGTTAGTTTTTTTACTGCTTTT
3	87136028	SCAFFOLD135798_1334	0.31	0.22	ATTAACTAAGTCATTGACTTCCTCTGAGCATCAATCTGCAAAGTGAACAATAATCCCTGCCTTGCCTACTCTATAGGGAAGATTCCATGAGATTCCCTTA[A/T]TCATAACCAGCACGATTGGCAACTAGTTTGTAGTGGGGACAAGCCTGGACTAGGGCCAGACTCCCTGGCTTCAAATCTAGTCAAATGCTTACCGACTAGC
3	87355270	BES10_Contig789_3212	0.49	0.01	ACAAAGGAATCTATTCTGTACTCACTCCGTATCAGATTATTCATGGAACGCCTTTGACTGTAAACGGGAGGTCTGGGACAAATCCCCGTGTAGGTCTGCT[A/G]GAGAACAAACTTCAAGAAGCTAAATCCTTTAAAAATTGTCAAACTTGGCTCCCTCTGATTACACTGGAGGATATGCAAACGAGGACAGTGTCTTCTTGCC
3	88877916	SCAFFOLD105739_19076	0.15	0.10	ACATCAACATCCGCCCAAAAGGTGAAAACTATAAAAGCAATTCTTTGTGGACCAACATGAGGGAACCAGTTCAGTGCTGGCCTGGCTTAAACTGGGAATA[A/G]ACTTGAGAGAAGCTTGACAGCTGGATCCAGGTAAAAGTGAATAGGTGGTTCAATGAGTAAAGAATCCTCAATTCAGGAGACACAGGAGATGCGGGTTCAA

3	88878405	SCAFFOLD105739_19565	0.16	0.10	CAGGGCCCGGCACAATGTCTGAAACAGAACAAGTCCATAAAGAAGACTAGCTATAAAGGCTTCTGTTGTGTCCTTGGGCAAGGTGAATTTCTTGGGTCTC[A/G]GTTTCTTCATCAGAAACTAATGGTGTTGATTAGTTTGCATCTCTAGCATTCTACTTTATGATTCCATGAAATTTAAAATATAACTTTATGTAAAAAAACA
3	90283493	SCAFFOLD10503_7841	0.40	0.50	GTAATGGCACCATCCGTGAACACCAATAACCCTTTCCTAGAAAAATCAAAGTTGGCTGAACAATCTGAGGGTATATGGCTGAGCTGTTGGAGAAAAAAAG[A/G]GAGAATTATCAACAGAAACCTACAGACAAGAAGTATTTCTATTTACTAAATATTATATTTTATCTCTCACATGTATAATGGCATGCTAAATATATACCTG
3	90511672	BES4_Contig326_910	0.14	0.25	TACAATTTCATGGAGATTTATGCACCTGCAAAGTCGGATATATTAAGTTCCCGCAAAAGCTTGGCTTTTGGGGTGTGTTCTTTCCAAAGACTCTGTGGGG[A/G]TACATTTTCAATGTGGAGAGCAATACGCAGGGACCAAAATCAATGGACTTAAATTATTAAAATTCTAAGGACTTGCTACAGATCGGGTCACGTATTATAA
3	90945586	SCAFFOLD312033_10780	0.39	0.29	TAGCATCTAGGGAAAAAAATTCTTCTTTTCTGATTTTGTAAGGTAGTCGATGCTGCCTCGCAATACACTCCTGGCCTTTCTTGTACTGGTCATTTTCTTT[A/C]TTTCTCCTGTGTAGCTGCTTGAACACCTCCCAGCGGGTGAGGAGTGTTTGTTCAGGTGGCACAGAGTAGAGAGATCTTGACTGGTAGAGAGTAACTTAAT
3	91323695	BES3_Contig74_659	0.27	0.29	AGCTGTTATTAATCTCATTTTACAAATAAGGAAAGTAACATCCAGGGAAATCTGAATCTATCTGATTCTTTATCCTGTAAAACAGCAAAGAGTTCTCAGA[A/C]ATATTGAGAAAAAGAGGGGTACAAAGGTTAGGAGAGGTTTAGGGAACAAATGAATAGGGGAAAAGGAAGGAAACAGGAAGAAGAAAGATGAGGGAGAAGT
3	93241855	SCAFFOLD102649_2408	0.36	0.28	TTACTGATGTGTAAATCTCTCTGGGGAACAGCTCATCTGTGTTTCCACACGTTGTAACTAGGGAGCCTGGGAATAATTGGCTTTTTTTTTTTGGTGAGTG[A/C]GTACTTGCCCATCCCTGAAGGACTCCAAAACTCTGCTGGAGGGACATTTGATCAGGCCCTTTTTAGAGGCTTCTTTGCACTGTCTCGCCTTATTAGATGT
3	93417390	SCAFFOLD322630_602	0.24	0.21	TCTCAGGCTTCTGGCAGGCAGCCTGCTGGGATCGGGTTTAGGTTTAGATTTTTCTTTTTTTTTTTTTTCTTTCATCAGACCTGAAACTGAAGCCACAGAC[A/G]TTTTGAAACCTTCTCTCAGCAGTAGCTGCCATGCTTCGTGTCACTTGCTTACCCCTCCCAGGCCATGTGGATCCATAAATGTGTCCAGAGGTCCGGTGAC
3	93639482	SCAFFOLD236264_6838	0.30	0.28	ACCGTCTGTGACTCATTGTGGTTAACTGGTTTCTAGCAAATATTTGCTGAATTGATGGATAAAATCCAATCATTCAAGACCCAGACTAGATGCTACATCT[A/G]CTAAGGAGTCATGTCTAATGTGTACCAGTCTCCAGAAGTAATCTCTCTGTTCCCTATATGTTCAGAGCATTTCATCATTCCCCTCTGATGATAGTTTTTA
3	94144416	SCAFFOLD35718_5721	0.43	0.43	TTCTTTGAGGACGAATGATACACTATACTGAGCACAGTGCCTGTGACACACAGTAGGGTCTGAACAAGAACAATCACAGAGAAGAGGGAGGATTAAATGA[A/G]GCAACCCATGTGACAATGCTTGGTGCATAATTCAATGAGTACTTACTGCATGAATGAATGAGCTCACAGAATGGTTGTGAAAACAAGACATAAATCCCCA
3	96196218	BES1_Contig417_1051	0.46	0.03	TCAACCTAATCCATGGGTGTAGGTGATGTAAACCTCAGCACAACGTTCCAGGTGGGCATAATTATTCCTGATTTACCTTTAAGAACACAAGGCTCCTGGG[A/G]GTTACGAGTCTTGCTGATGGGCACACAGGCATGGAAGGAAGACATGGGATTCAAATGCAGGCCTGTCATCTCCACAGCCCACGTCTGCCTCTCTGTTCCT
3	96196281	BES1_Contig417_988	0.31	0.40	CAAGATAGCCTGAAGCCAACAGGCAACACTTTTTGACCTTCAGAAGTGGCAGGTTCGTACGGTTCAACCTAATCCATGGGTGTAGGTGATGTAAACCTCA[C/G]CACAACGTTCCAGGTGGGCATAATTATTCCTGATTTACCTTTAAGAACACAAGGCTCCTGGGGGTTACGAGTCTTGCTGATGGGCACACAGGCATGGAAG

3	96633512	SCAFFOLD186806_2279	0.08	0.09	ACAGTGCTTGGGGCCACTGTATTTCATAAATATTTGCTATAGAAATTAAACCCTTATTTTCTGCTTCATGAGGTCTCCTTCTATTGCCTCTTCCATAATG[A/G]ATACAGATGTCAGCACATAGTAAGTAGCTTGATAAGAACTTGATCAACTGAACTGAACTGACCCCTTCAAACTTTGTTTCCTGAGTTCAAAGGGCCTCAC
3	97108295	SCAFFOLD235642_5974	0.21	0.09	AGTCCCTGGGGTTGCAAAGAGTCAGACACAACTTAGCTACTGAACAACAACAATGACAATCCACTGAGACCTCTTCTCTGCTTCTCCAGATCCTGTCCAT[C/G]CTTTAATCCTGGAGAAGTTCAGGATAAGAAAGAATTAGAAAGAAATAGTCATTGAGACCTTTGATAAGTGGTGAGCCACCTGGCCCATATGACAAAGCTT
3	97403450	SCAFFOLD90080_5835	0.49	0.44	GGCAGATGGTGGCCATCTCCACTTCCGGGGGAGAGGGGGAGGTTACCAGTTATAGGGGAAATGACATCAGGTGGGCTCATTTGTTACCAGGAAAACTGGC[A/G]GAGAGGCATGGCCCTCATGGTCCCTTTGATAAGCTCTCATGGTGGAGATTTCTGATGAGATGTTCTGATATTCTGATCAGAACAACTAAAACAGTCCCCA
3	99395953	SCAFFOLD15114_12713	0.33	0.46	TTTCAGCGTTCTCTCTGGAGGTTTAGCTTCCTGGAGGTCAGTGAATAGAGTGGGCAGTGGTTTCAGGAGGCAAACATCCTCAGGCTGCTCTCCCTGGGCT[A/G]TGCTAGAATCTGCCCAGAAACGGGGGTGCATTCAGGTGGTTCGTGTTCAAAAGAAAAAAAATCTCAAGATGGTAATTGACGGCATAATGGAAATTGACAA
3	99467830	SCAFFOLD1629_3371	0.11	0.22	TTCCAGCCATCCCTGTGAAGGGCTGGCAGAAGCTGATGTCTGCAAAATACAGCCTTCGAGATGAAAGTTGCAGGGAAATAAAGCCTTAAGACATTACAGT[A/G]TTTTCAAATATCATTATTACTGCTCTATAAATATCCCACACCCACAGTGTATCAGGTGCTACATGGGAAATATTGGATTCACTTGGAGGTTTTCTTAAAA
3	100811876	SCAFFOLD115003_498	0.31	0.29	GATTGTATTCACATACCTCAAGGCAATAAGGCATCTATACTTTCTTGATGTGTGGACTGGGATTCAAATGTTATTCCTTTATTTTCCACTGGACTCTCTC[A/C]TGTCTCCTTGGCACATGCATAGGCTCTGACCCTGTTGACCAAGGATTAGTTGGCTTGGGCCCACTAAGGTCTCTGCTGCACATGTGCATAGACTAAGGTC
3	101449660	SCAFFOLD163794_561	0.30	0.28	GCAGAGCATATTCTTAAATATGCCACTGTTATATGTGGCAGTTGTTATAGGATTAGATTTTCAACTAGTTGTATAATGTCAGTCAAAGCTAGCTAGAACA[A/G]TGCTCAAGAGAGTTAGATTAAGGACCTATTTCCTTTTACTGCTTATAGAGAAAAACAAATGGCCAGCTGAGTCTTTTTCTTCTCATTTACTATATTGTAA
3	102962479	SCAFFOLD60012_53629	0.48	0.10	CTGCTGGAGTGAGACAAGTTTCTTCCCGTGGTGGTGGTGGCTTGTGGTGGAAAAAAAAAAAATCATGCATGACTGGGAGACTCGCCTGCCTGATTCTTGC[A/G]ATAATATATTGAGAATCTGTTGCTTTACAAATGTCATACCACTGATGTAGCGGTCAGCCCCTCACTCTGAAAGATGAATGGTACTATTGGAAATGCAATA
3	104508445	SCAFFOLD166616_2738	0.48	0.06	GGGGGCAGAGCAGAAGGTGGTATTCATCACAGGACGAGTCCACCCAGGGGAAACACCCTCTTCATTCGTGTGTCAAGGTGAGTTGCAAGACCTCATGAGC[A/G]TACTGTGCCTAACCCCCATGTCCAACTGGGGCACTGATGGCCTCTGGGACCATGTAGCCACATGCAGAGTCCTTATCTTGGGTGACAATTTACTTCACTT
3	104517418	SCAFFOLD240852_12041	0.45	0.41	GGAGGAAGAGAAGGAAAAGTGGCAGAGTTTGGAAATGGAAGCTGGGTGGCCGACCTCACTGCCCTGTGCCGACCTCACGTGGCCATGATGACCTGCATCA[A/G]ACAAACCACAACTAAAGGTTCAATATTGCTATTTCTCCTGCTGTACAGAGCCAGGCACACAGCAGATGCCCAATAAATGCCCATTGGTAGATTAGCCCCA
3	104551588	SCAFFOLD245243_1197	0.38	0.29	AGGTGACAGCTCCATCTCTCCCCTCTGCTTGCTGCCAGAGCCAAAGCTATAATCTAGAGAGAAAGAAAAACCGTGTCTTTTATCATAAGTGAGAGAAGTC[A/G]CCCTTTAGAAAAATAATGATTTCTCTTAAAATTCCTTAAAGTTTCATAAACCACCTCAAACTGTTGTAGAAGCTGTAAAAATTTGATGAACTCAAAGCAC

3	106768855	SCAFFOLD50270_3726	0.31	0.27	CATCTTTGGAGGAAGGACCATTATTCAGTCCTCCTCAAGCAGAGATGGAGGGTGGGCAGGAAGATGTACCCAGGCCCAGAACAAGCCTCTCAGCTGTAGC[A/G]AGCCTCCTTCAGCCTGGAGTGAGAGGTGGTGCCAAGGGGAGTTAGCCAGCCCAGGCCCCATGTCTAGAGAATGGCAAATGCCTCCTGCATCACTACCAAA
3	107915818	SCAFFOLD260312_19049	0.17	0.16	TTTAAATGAGACACAGTTCCCAAATTTCAGTAATTTTTGTAAAATGCTAAAACTTTATGCTGTCAATATGGTGTTCTCCCTCTCCCTGTCCCTCCTCCCT[A/G]CTTAACTTTTTTCATTTTCCCAAAGTACTTATCACTTTTTAATATATGGCACTGTTTACTGATTTATTATTTAAACTTCTTTTTTATTTATGCTGCCTCT
3	109598099	BES4_Contig389_420	0.12	0.25	GTTCACTGCTGACACGTGTTAGGCACTTTTTATCCCCTGATTCAGCCTATCCTCATAATAGTCTTATTATTTCCATTACAGTGATGGGGAAGCTGGAGAC[A/G]CAGATGAATTGCACATTTGCCTAAGGTGACGCACCTCCTAAGAGGCAAAACTCAGGACATCAAGCTTAGAACCTAAGTGTTTAACAACCATGCCAGTGCC
3	110254831	BES7_Contig426_708	0.33	0.37	TCAATGGTACTCTGAGAAACTCTGAGTGAAGCCTGTTGACACAAGTGCATTTGAAAGTTCAAACACCAACAGATTTGTACTCTCTGTTTAGAAAGAAAGG[A/G]GGGAGGAGGAAAAATATCTACCTCCCAGTTTTTATAAAGTTTAGGTTAAATTTATACTTATTTTTTTGCTGTTGCTATTTTTTAATTGCTTTATTAACTT
3	110433324	BES7_Contig393_1350	0.50	0.43	AGGAGGAAGCAGAACAGGGGTCCTGCCGGGAACAGGTCACAGCAGCACCAGCTGCTCTCACCCTCTCGCTGTGTCGAGCATCCAAAGTGAACCAGAGGGC[A/G]ATGGCACAGCGCTGCCCTCTGGTGACGGCCTTTACTCCATGCGGGTTTTCCGTGCCGGAAGAGAATCCCACAGCCCTTCCGCACTGGGGCTGCACCTCTG
3	114586153	BES1_Contig302_467	0.46	0.26	TCCAATAGGCCTCTCTGGTCCTGAGCTTTCCTCCTCTTTGCCTGGTGGCTTGCTTGCCCCTCAACACAGCACACCCAAACTTGATTTGGATCTCATTTCC[A/G]GGACCAGCTTCTAATGACACAGCTGCCCCCAAAGCTTTCAAGTTCACCCAAGCATGATGTGACTCTTCTTTATTCATCCCAACAGCACCGTCTCAGCCTG
3	114586527	BES1_Contig302_841	0.22	0.48	AGTCCACACAGACCGGTCTGAGGACGGCCTCCCAGGCAGACAAGGTCTCGCCAGTTTAAATGTGGCTGTATGTTTTACATTGAATACACCCCGGGTGCTC[A/G]GGGAAGAGCTACTGCTGGGTCAGCAGATAATTCACGGCAGGAACCCAGAGAACATCTGGAGATGTAGTGAAAACAACCAGATGGGCACCTCGTGAGAACG
3	114615134	SCAFFOLD51388_919	0.12	0.26	CCACCCAAGAGACATGGAAAAGAAGGTTCGCGCCTTCCCTGGGAACACAGCCCAGGGAAAGAAGAGGAGATGGCAGGCAGGCTGCTGCCTCCTGTGCATT[A/C]CGATAGGCCCACTGCTTCGCAGAATCTCAATGTTCCAGCCCCGAACAGTTGGTGGAGTGGAATTTCAGGAAAGGAGCGAAGCAGAAGTGCCAGTCACTTA
3	114861523	BES2_Contig402_695	0.06	0.12	TGTATCAGCCGCTAGCTGTGTGGCTTTGGAGGTCAGTGAATTATTCCTCACAGAGCACGCAGCACAGGGTCTGATAAATGCCCATGATTTTGTGTTCCCT[A/G]TGGGTTCAGCACTTAGAACCATCTGTTTATTTGATCAGCGTGAGTTTTTCCAGGGCCTTCGAGGGGCCCACACTTTGTTTAGGAATAGATCTATCACTTA
3	114946414	BES2_Contig293_493	0.46	0.40	TAACCAGAGGAAGGGTAACATATAAACACAAGGCATAGAGCTGCGGTGACATAGTTCCTAAATCCTAAAGCAGCCGTCATGGCCTTCTCTGATCTGATAC[A/G]TGGATTTCTGCTCAACATGTCTCACGAACCTATACACGATCTGATACATAATCTGACTCACACACCTGCCTGTAGTCTGACTTGCGGTCCATCACAGCTC
3	116067951	SCAFFOLD115712_6276	0.29	0.23	AATAACTGGAGTATCAGTGGCCAGTCCGCTCTTCTGCCTGTTCTCCAAAAGGTCATTTGTTTCCATCAGACTTTGGATTTGTGCTGGGCAAGAAGTAACC[A/G]TCTCTGGCAAGACCTGGCATCTGGGCTGCTGTTTGGTTGGATGGAGGCAGCTCCCGTTTAATTTCAAGCAGTGATTGCCAGGCTTCCCTGATTGATAATC

3	116468363	BES2_Contig244_1403	0.10	0.31	TCGGGAAAATTCGAAACATATGCAATTCTTATTTTTGTAGCACCCTTTTCATATCTAAAGTCACTCTCTTAATTTAGGAGTGCAAATGCAACCTTATGTA[A/C]TTTGGCTTGCTCAATAAAATTATCATGTTTGACAAATTCAATCAAAGGCTATAGCTTGCTCAGACAATAATGACAAATTTGTTGAGTCAAGTTTTCAGTG
3	116738083	BES8_Contig446_1023	0.07	0.15	TCCTCACACTCACAGAAATCACGTTTCTGTCCTTCCAAGCACTAATCTCAGAGTAATTAGTGTGCTGACTGCCATCTTCCTCCATGAGACCCCACGTTTT[A/G]GACCTGGATGGGCAGTACTGGTTTGGGCCACCACTGGAGCCCCAGCACCTTGCCCGGGGCACAGATACAGTCTGGCTGGGTAAGGCGCCCGGGGGCTGCT
3	118803962	SCAFFOLD151038_10401	0.33	0.23	AATCAAGCAGGATAATTCAGAGGATGCTGTGTTTGCCTCCTCGAGGCCACAGGAGTGAAGTGAGCCATGGACATGGCTGGATTCCAAATAAGCAGAAAAG[A/G]GGACCTTAAAGCAAAGGTTCTATAGTGTGGCCACTTGCCCTGCCCCTCTTGCTATTTATATCAGAATGGGAAAAATCCTTCAAATGGTTCAACAAGAAGT
3	118803997	SCAFFOLD151038_10366	0.27	0.28	TGCAAATTCCTCTTTGGGGTGGAGCTAAAAACTTGACTTCTTGTTGAACCATTTGAAGGATTTTTCCCATTCTGATATAAATAGCAAGAGGGGCAGGGCA[A/G]GTGGCCACACTATAGAACCTTTGCTTTAAGGTCCCCTTTTCTGCTTATTTGGAATCCAGCCATGTCCATGGCTCACTTCACTCCTGTGGCCTCGAGGAGG
3	118806605	SCAFFOLD151038_7758	0.19	0.20	ATCTTTTTCCTATTATGCTAATAAGAGCAACAGGGCCAGCAGACATGAAATGAAGACAAGATGATTTCCTACGTGATCCCTATATTCACGCTATGTGTGC[A/G]TAGTCAGTGTATGATCCCCCAGAACTCAGCTGTAAGGTAACTCTCTATGAGTGTATCTGTTTAAAGTGTTAACTACAGAGTAAGCTTGATTGTCAGCACA
3	118929241	SCAFFOLD318144_4466	0.41	0.42	TTGCAGAAAGGAGAAAGAGATGGACGTGGAGGGAGAGGTAGGAGCCAGCTTCACCCTCACTGCTGTGAGCTAAGCTCTGCTCTGGCAGGGCCACCCCTGC[A/G]CTCCAGTCTCTTCACATAGGTTGGGAACCTGCTTTGTCCTTGTTTCCTAAGCCCTCTTGGTGACACTTCCCATGTGGATTCTTAGGATCTAGAGGTATCC
3	119178717	SCAFFOLD30021_9042	0.07	0.17	ATTATCAATATATAAATTACAGGTGTACAATATAGGAAAAGCCCTGTGAGCCTCAGTTTTCCTCCCTGTAAAATAGGCAGCTAATCCTTTCTGCAGCCGC[A/G]CTAAAGGCTGCATGAGAAGAAATAGGTGATGGTGCCTGTACAAAGGAGCTATAAACATGACAGCGTGCTTCTCCTTTTCTCTGGACTATTTTGAGAGGTT
3	120051960	SCAFFOLD155668_12939	0.38	0.21	TAGGAATATTCCCATTGGAGACCCAGGCTGGGATGACGCTGGGCTGGCCCAGCCTCCCGCATATGGGATCACTCTATTCTCAGAGGCAGTGGCTCTCACA[A/G]CGAATCTTCCATGCGACTCCTGACTCCCAACAGCCAGCCGTTGGGTGACTATAGGTGCCTACACTGTTCTCAGCAGGAGATGCTTCCCCAGCGAAGGCAC
3	120343156	SCAFFOLD15634_20691	0.10	0.15	CCCTTCTCTCACGGTTGTTGGCCAGCAAGGGCACCAGCGTCAGCGTCTTGGTCAGCGAGCTGTTTGGCGGCACTTTTTCCCTGCAGGAAGAAACACGGCC[A/G]ATTTAGCCAGTTGCTCTTACAGGCTTAAGGCCACCGGGGCCCCCAGGAGACCTTGTCTACGCTCACGCAGTTCCCAGAACCCACGTTCCCCGCGACTTGT
3	121492212	SCAFFOLD125573_4180	0.45	0.46	GAGGGACTGAACAACAACAAAAAATATACGTGTGTATGCGTGTGTATGTTAGATATAGATTCAGATATATGATATGTGTGGAGATTTTCCAAAAATACAC[A/G]CAGGAACACAAGAAACTATTACAGTGGTTATCTCTAACCATGGAGTAGAGCATGTGAGACAGAACAGACTCACATTTCATTGCATTCTTTCTGTGCTATT
3	121492370	SCAFFOLD125573_4022	0.34	0.34	GACAGAACAGACTCACATTTCATTGCATTCTTTCTGTGCTATTTGAATCCTCTTTCCATGTGCATGAAAACACTATTTTCATCATAACAAAGTCCCCAAA[C/G]CCCACTTAGCTAATGGATGCATCCACAAGAAGAAAAACTGCCTGCATACATATGCAGATTTAAAAAGCACTAAATTCTCTGCTGTATTCTAGAAAGAATT

3	122128143	SCAFFOLD160057_31643	0.39	0.28	GCTTTCCTGCTGTTATCTTGGTTTCTTACTGTTATCCAGATAATTCACAGAAACTGAGTCTGCAAATCCAGGTCCCACCCTCCCCTCTTGCTCCAGCCCA[A/G]AAAGTTGTGGTTGTGGGCAGGGGTCTGGGGTGGGAGGCAGGACGTGGTGAATGACACAGGGAGGAAGTGATCACTGGCATGGAGAGCAGAGACATCCATC
3	123464468	SCAFFOLD25591_13966	0.31	0.18	TGTGTCCTTGACTCACACGCTTTGACCATGCTGCCTTTTGACTCCCCATGTGTGTGGGTTGTCCACACGTTAAGCTGTTCTCTGCAGGGCCAACTGGGGT[A/G]TCCTGTGATTCCGTGCAGTTCTGACCTCCCTAGAGAGAGCCTCAGGCCACGCCCCGCCCCATGTTAAGGGCTCAGCCCTGCAAAACTGCTCCCTCCAGCT
4	2075445	SCAFFOLD146556_5946	0.50	0.00	TTACTCTCTTAGCTGCTTTCAGGCATATAAGACAATATTGTTACCTATAGTCACCACCAAGCTGAGTATTAGATCCCTCGTACTTATTCATCTCACTGCT[A/G]GAAGAAGTGAGATGTACTTGAAGAATTTTTCCCACACCTCTGGACTCTGATAACTACAATACTACTAGGTTTCTATGATTTTGACTTTTTTTTTTTTTTT
4	4603423	SCAFFOLD240033_40040	0.01	0.07	ACACAACTTAGTTACTGAACAAAAGCAGCAACAAAAATCCTGTGCCATAAAGTTATGACACAATTTATCACAAGGTAGCACCCTTGCCTCCCAGGAGCTC[A/G]GAAGTCTGCAGATCAGAGATACGTAATGGGAAACCCTAACCTCACACAAGGGTTTCCAGCAGGGGAATCAGCACAACCACGAGAGGTGAAGGTCAGGGCT
4	5657544	BES3_Contig373_1182	0.49	0.33	GCAGCTCCTTAATATCATGGATAGATGCTGTAGCGGTCAATATAGAGGATTATATGAGTGAGACGCTTGGGAGAGAGGAAAAAAGAGAGGTAAGGCAAAG[A/G]TGGTACATTTTTCTAATCTGATTTTACAGTGGCTCAATTACTCTCTTTCACACTTCATTTCCTGTGCGCCTCTAAATGTATACAGCTCATGAATGCGAGC
4	8306588	SCAFFOLD110043_29262	0.22	0.16	AATTAAAGGTGGAGTCTCTTTGTTTTTGATGATGCATGCAGATATATAAAATTTTCATTTCCTTAAGGTTTTAGAATTTATAGAATGCTTTGTTAATTAA[A/C]CTTTTTCACCCAACAGACCAGTTTCAAACCCCTCAGTATCTTCAATAATCTGAGCTCAGACTGCTAATCAGTTTCTCCCTCTTTTCAACTGCTTAATTGG
4	8306720	SCAFFOLD110043_29130	0.22	0.16	TTACTAGACTTTATGCTGAGGGATTCAGTAGTTGGTGAGTGTCTAGACGTGTGGTGAAAAATTAAGAAAACAGTCACAGTGACTCCTAGGTTAAAGAACA[A/G]TTTGATGATCAATACGGTTCTTAATGGGAAACCAATTAAGCAGTTGAAAAGAGGGAGAAACTGATTAGCAGTCTGAGCTCAGATTATTGAAGATACTGAG
4	10541198	SCAFFOLD151622_1051	0.18	0.25	GGGAAGCCTGGCAGGCTGCAGTCCACAGGGTCGCAAAGAGTTGGTCATGACTTAGTGACTGAACAACACAAATACACAAAGGCAAGCAGTTGTGAAGCAC[A/G]ACCTTCTTGTACTAGAGAAACTGCTCTTGGGGGTTTCAGCAGCCCAGCCGTGGCTGATGCTGCAGAAGATGCACTGGGCTCGTCTTTGGATGCTGGGTAG
4	11519848	SCAFFOLD130927_2750	0.11	0.15	TTTTTAATCCTCTGCTTCCCTTGCCTCTTCCATTCTTCACACTTTTCAAATTCTCCTAATTTTCTGCCCATTAGTTCCTTTCCAATTTGCTGTAGCCAAA[A/G]GAGCAAAAATCACTTTGGCCAAGCGGGGAAGAGAAACCAGACAGCTTTGATAAATGAGCCAAGCCTGAGCTGCCTGCAGAATCGAGCAAGGCCAGGAGGG
4	11522963	SCAFFOLD130927_5801	0.11	0.16	TGACTGGAGTGCAGAGTGACCCTGTTATCACCCCTTTCTGTTCCATCTACAACTAACTTTATATCATATGGCTTCATTTGCATTTAAAACACGTGTGTGT[A/G]CTAAAGTCTGAGTCACAGTGGTAGTAGCAATACAGTAAGAAAAGTTTTTCTTTTCCTGTATTTAAAGATTGGGTTTTATGTCACTGGATGAGAAGTCTGA
4	11687104	SCAFFOLD221336_1415	0.07	0.08	TTCATTCAGTTCAGTCTCACAATTTCAGATCCTACAACCATAGTGGCTATGCTGCTTCAATAATCCAGGTTTTCCCCTCTTCACACAAAGCTCAGTCAAC[A/G]CATTAATAAGCTCTGCACTCAGAATTTTACACTAATACAGAGTTCAGAAAACTAAGTTTTAAAGATATCCAAATGTTTTTTTTGAAGTTTTGATATATGC

4	11913823	SCAFFOLD160549_686	0.29	0.15	TTTGTCTGAGCCTTCCAGTCTGACTCACTGCCTGGGGAGGGCAGAGTTCAGCCCGGAGTTGCCAGCAAGCCCCTGGCCTTAAAGGCACAGCATGTGGATT[A/C]TGGCTTGAGAGAGGAGCTGGGCGCTAACAAGTAGCTGGGCTGCCACTCACTAATTTCCAGCTGCCTTGTTTATGAAGCGTTGTTGACAAACAAATTCCAG
4	12016246	SCAFFOLD51473_4481	0.15	0.08	ATTGCAGTAACTGTTTGGGAAATGTGATTATAATTTACCTTTTAGGATAACAAAATTCTCAGCAAGTTTTGGAGAATTCGAGAAATTCGAGCACACGTTT[A/C]TAAACTAAAGGAAAAATCTCTCTGCAGAGTAAACTGATAACTGGAAAAGCATTCCTGGAACCTGATGCAAATAGCTAACGCTGCCCGGCTCCCTCCCTGT
4	13174293	BES10_Contig790_1642	0.49	0.03	AACCACTTTATTAAACAAGTCTTCTAAAGTTACTCTCTGTCACATCACCTTGGAAATTTTCTTCAAGGCACAGTTATATTTGACCATCTTGTTTATTTGT[A/G]TACCAATTTCTCACATGACTCCCTCATTCGACTTAAGCTTTTCTGTCTTGTTCATATATAATGAACAAACAGATGAGCCAGTAGAGACCAGATTGCATTG
4	14013999	SCAFFOLD100382_24646	0.24	0.18	CTCTCAGAGACAGTTTACACCAGAAAGAAGAAACACGGAGTCAGGAAGAAGCAATGAGATACAACAGCAATGAAGAGCAGGGAAATCAGTGAAGTGTAAT[A/G]CAAAGCATAAATTAGAATGAATGTAAAATGTTTGTGAGCACTTAATCACAAATTCTGCAGAATTAATTAAGCCCTAAACAGGTTTTAGATGATGTGCTTT
4	14396692	SCAFFOLD111544_8475	0.15	0.21	TTCTATGTGTTTAAGAAATGAGGCCCATTACAATGTTCTGCTAAGCAGCGTCTACCTAAGAGTCATCTTACTCTAGGTATGCAATGAGGTCCATAAAGAC[A/G]TGGCAAGAATCCATGAAATTAGAGTTTTGTCTTTACAATGAGTTAGGTCAAATTTTGGAGCAAAGCTTCTCTTCTCTCCTTCCCCTAACTCTCTCTCCTT
4	15464440	SCAFFOLD5665_12793	0.15	0.17	TGCCTTGGTTGGCATGGTCTTTCTCCCCTCACCCCCACTGAGTGCCTTGCCCTGTCTCCTTCCTGTTCCCTCTGGAAGTGGCCTCATCAATCATTTTCCT[A/G]TTTTTCTTTACCTTCAATGTCTCCTCTCTACCAACCCCTCCCCTTGTCTACAGACCTGTTGGCTTTCCTCTCAACCTAATGTCCTACCCCTGACCCAGTG
4	16324268	SCAFFOLD216478_17289	0.12	0.22	AAAACTGAACGTAAAGTGCTTCTTTTCTACTTTTCTAAACTACTTTTTCTAGGGACTTTTATTTCTGAGAGCATTAAGCAAGTCTTCAATTTACTGGACT[A/C]ATTTAAAATTCTAACCCCCCCCTTTTTCTCTCCTCACACACATCCGTATTTTTCTCTCTGTTCCCCGCTTTGCTCTCTGAGCTTCGTACTTGTGTAAGA
4	16532789	SCAFFOLD116028_5808	0.28	0.30	GCAGATGGAGGAAAATCCACTGCACCCACTCTCTGGGACACACACCCACAAACACACACACGTCAAACAAAAAATAAAACAAAAAAAATCCCTCCTCCAC[A/G]AGTGAAAAAAAAACCCCAACAATGCTAGACCCTGAGTAGACACTCAGAAATACTTTTTCATGATCCCCAAGCCTGGTCCTCCATTGTAAAGTTAGAATT
4	16532909	SCAFFOLD116028_5928	0.28	0.30	ACAATGCTAGACCCTGAGTAGACACTCAGAAATACTTTTTCATGATCCCCAAGCCTGGTCCTCCATTGTAAAGTTAGAATTTAACAAGCAAGCAATCTTA[C/G]TAGAAAACTTGACACCTGGAGCATGGGTAAATCTACACAGAACACTCCAGACTATAAGGTGATCAAGTAAGAACAGTACTCGGTTTTAAAAGTGCACCAT
4	16533094	SCAFFOLD116028_6113	0.36	0.44	GGCAGGCAAATTGTTTACCAGCTGAGCTTCCTGGGAAGCCCCTCCTATACTTACCCATGTATGTGTAGCTGGCTAGGGATCTGACTGCTTCCTCACAGCT[A/G]TGTCTTTTCTCATAAACTTTCCCTTCTCCCTGTCTTCCTCAGTGTCCTACAGAGAACTGCTGGGTTGCTTCCTTTCATGAGCTAATGGTGCACTTTTAAA
4	16613145	SCAFFOLD110655_10917	0.23	0.12	ACTTAGCAGCAGCAGCAGCATAGTTGAAGGAAAGAAAAATACATCAGAACAAGTACAAAATAGTACATTACAACAATTACAAAGCAGTGTATTAACCAAA[A/G]AGTGAATTCTTGGAAGTTCTGGGTTTTAAGTGAGGCTTGGGGAAGATAGCTGCTGTAAATCGACAGGGAGGAAGTTAAGGAAAAGACTTCCTGTTTGGGT

4	16613381	SCAFFOLD110655_10681	0.25	0.12	ATATTCACACCCTGGCTTAAATCTTTCTTAAGTCATTATGTCCAAGAGTCTAAAATGGCACTCAGTAGATACTTGTTTTAATCCAATTTCCTGTGTCCGC[A/G]TGTTCTTTCCTCTCCTTTTACACTATACCTCATCAGTTTCATCTCTCTGATTCTCAACAAAACCCCTTCTGTGTATTTATCAACCTTCACATGGCAAGTG
4	16615834	SCAFFOLD110655_8344	0.24	0.12	CTTATCCTGTCTGTTCAGGCCTGGGGGCTCTAAAAAGTATACCGCAGCAGGGTTCAGGCACCTGGCCCCCAGGACCGATGTGGAGACACCAGCCATTTTG[A/C]ACTGCAAATTGCACTCATAGAATAAAATGAAGTGAGAGAAATGCTACAAGCCTAACACAGAACATAAGCTCTTGGGGTCAGAGAAAATGAGAATAGAAAA
4	16615945	SCAFFOLD110655_8233	0.23	0.12	GCACTCATAGAATAAAATGAAGTGAGAGAAATGCTACAAGCCTAACACAGAACATAAGCTCTTGGGGTCAGAGAAAATGAGAATAGAAAATGTGGAATGT[C/G]CTGGAAGAGGTGGTTGCTACATTAATGTGAGGTTTGGTCTCTTCCACCACAGAAACATTTTCCCCTGTGGTGACTAAGCACTTTAAAGGATCCATTTATC
4	18625991	SCAFFOLD150972_1596	0.36	0.40	TCCAAAAGATTAATGAAAATTTAACAATGAATATTTCAAAACAGGCCAAAAGAAATTAAGAAAATGGAAAAGCCTATGAAAGAACCAGGAACATTGCCAT[A/C]AGAAAACTGAGGCAAATATGCAAAATATTTGAAAAGAGAGGTTATGAAACTGAGAAAGATATTTTAAAATTAAAAAGTCATTTAAAAAATGAAGTATAAA
4	22890326	BES1_Contig599_2824	0.49	0.37	TTGGTTACTGTATGACTATAACTGTGCCAGCAGAACACAAATAAAACCATATGGGCTAAGGAGACCTTGTAGCTACATTTCTGGTTTTTCCATGGTGAGC[A/G]GCACAGGGGCAAGCTTTGTGTTAGCAAGACAAGGTAGGGAAAATTCAGGAGTGGGGCAGAAGCGCAGGTCAACTCCTTCTTATGAGTCTGCTCTATGAGG
4	23580349	BES9_Contig548_1270	0.20	0.16	CATATAGCAAAAGACATGTGATGAAAATAAGTATGGAAAACATTGATTTTAACATTACAAGTGATAAGATTCAGTGATTTGGTTCCACCAAGCTTTACAT[A/C]TTGAGAACTCTTTAGGTTCGTGTTTCTACATGTCCTCCAAGTCTGGCTTCCCAGCTCTACCTTTACTGCCTTACTCTAGCCACCACTAGGGTAGCATTTA
4	24924028	SCAFFOLD91917_1349	0.14	0.08	AGGCTGTTATGGGATTTGTTAAATAAATCTACATTTGAATTCACTGTCATATTCATAATTCTATAATTTTAAGGAAATATAATTAGAGATGAATCTCTTT[A/C]ATTTAGAGGAGCGGTTTTATATGTTCATGCTTTATGAACATGCTCATTGTTCGCTATAGTGCAGTCCTGAGATGCCGGTAAGGCATCTGTCCTATTCCTG
4	28014312	SCAFFOLD105231_4567	0.05	0.16	CGCACAGCTTGTGGGATCCCTAATTCCCTGACCAGCCGACCAGGGATCAAACCCTGGGTCCTTGGCAGTGAAAGCATGGAATCCTAATCGCTGGACTGCC[A/G]TGAATTCCCTACTTGGCAGTGAAAGCATGGAATCCTAATCGTTGGACTGCCATGAATTCCCTACAAGTAGTATTTCAAGTAATGAACACCCCTGGAAGAT
4	28116131	SCAFFOLD21740_3255	0.23	0.40	ATGGTGGGAACAAAAGATTTTCTCTCACTCTACAGCCTAAGTTATCTTAGCACCTGCTGCTCACCATTCACAGCCTGGCTGTTCATGGATTTTTAACAGG[A/C]TCATCAGAAAATCTCAGGGAGAAAAATCTTGAGGACATTCAACCACATTGAGATGCCCACTGATCATCAAGGATGCTTACAGTAACAGCCTCTTGGACTG
4	28601536	SCAFFOLD76779_1571	0.49	0.05	GGTATTCTTGCCTAGGAAGTCCTGTGGACAGAGGAGACTGGCAGGCTACAGTCCATGGTGTCCCAAAGAGTCAGATACACCAAGCATGTGGGCACGCAGC[A/G]TACTAGCAAACAGTCATGCAATTAACAAATAATGTTCTTGAAAGCCCAGATCAGCAGACTGGCTTTTAACGAAGACCAAGCACAAAAGTCGCATCCAAAA
4	28601726	SCAFFOLD76779_1381	0.02	0.05	TGGGTTCTTAATGGCTTTAGAAATTTCACTGCACATTTTGGATTGGACCAGTTCAAGTCACAGATATTTTCATAACACAGGAGCAAATAAATACACATGC[A/G]CAACAACCGCATCACTTCGGAAAGCAAACCCCTGACAAACTGCTCCTCAGCTACGTTAGGAAAAGGGGCTTCTGTTTGCATTATTGAAGTTTTGGATGCG

4	28848783	SCAFFOLD230196_1836	0.08	0.12	ATTTTTTTGGCTGTAGACAGAGGCCTTGACGCTGCAGCCCTGGGCCAGGCCGACCTTCACTGAAGAAAAGCGGCGTTAGACATTTTCCCACTATTAACCC[A/C]GCCAGCCTTGCTTGTCTCAGAGCCAGATCAGACTTGTGCACATGGCTTTTTGAAAGTTGATTTTCAATTTCTTATTAACCATTCTCCTGAAATGTTGAGA
4	29650677	SCAFFOLD126016_4728	0.17	0.30	CTTTTAAAGGTGTAAAATGTAGTACAAACTTTTTCTCTCATCATTGTTCTCCCCATACTTAGGTCTCTCTGTTATCATACAATGGGGAAGAAAGGGGGAA[A/G]AGGAGGTAGTTTGAAGCAAAGTTGTTGTATTTTCAAATTACTGAAGAAATGTAACCCATTCACATCCAGATGAGGGTAGTTACAAGTCTAGATATTTCCC
4	29650723	SCAFFOLD126016_4774	0.18	0.30	TTCTCCCCATACTTAGGTCTCTCTGTTATCATACAATGGGGAAGAAAGGGGGAAAAGGAGGTAGTTTGAAGCAAAGTTGTTGTATTTTCAAATTACTGAA[A/G]AAATGTAACCCATTCACATCCAGATGAGGGTAGTTACAAGTCTAGATATTTCCCCTAAATAGTGACTGTACTACACACTCACAAGTGTAGCTTTGCAGTG
4	29650804	SCAFFOLD126016_4855	0.18	0.30	CTTATAGATATCCATAACAGAAATGAGTAAGGCATCTTCTTCATTTGAACAAAATTTACTGACTACCTTGCTATGTCTAGACACTGCAAAGCTACACTTG[A/T]GAGTGTGTAGTACAGTCACTATTTAGGGGAAATATCTAGACTTGTAACTACCCTCATCTGGATGTGAATGGGTTACATTTCTTCAGTAATTTGAAAATAC
4	32991114	BES4_Contig444_559	0.47	0.39	CAGCCAATAAATTGCTTCCTCTCTTTTTGCAGAAAGGAAACAAATATATAAATATATATGGGCAGTCTCAGTTTCTTGCCACTCAATCCATCCTATGTAC[A/G]ATCTTGATTTAGTGTTAGTGAAATGTTATGATGTCATTCCCCTGTTTACAACCCTGGGAGTATGTTAGATTCACTTGCAAAGCTTTTAAAATAAACTCTA
4	33148502	BES8_Contig349_708	0.11	0.33	TCCTTGAAAAAAAAAAAACAAAACACCAAACTAAAACTTTGTGACTGCACTCTTGGAACAAAGCACGGGTGGCCATATTTTTGAGTCTCCAATGAATCTT[A/G]AGGTTCATTTTCTCATGTTTCAAGGGTTTCTTCATTTGCTCCGCAAGTATCAACAACACAAATTAAACAACTTGTTACTGGAAAAGCTCGGACTTTGGAG
4	33275393	SCAFFOLD195587_10773	0.49	0.50	TCTCAGTCTTAGCAATTTTCACTGAGAGATTCCTTCTACTGAGTACTGGATTTATTTAAGGGAGAAGCCAGGTTATGGAGGTTCTGAAAAGAAGGCATTC[A/G]TCTGTCATCTGTGCACACGCTCCAGGGCACATACCTTGAACTGAGCCTCCGGCGGCCCGGTAATGATGACCATCCTCACTTTCGCGTCTGGTGCTTCAGC
4	37514064	BES8_Contig497_586	0.20	0.23	ACTGTACCTAATTTATAAATTAAAGCTTATCATATGCATGTATAGGAAAATACATAGGATATATAAGTTTCACTACTATCCACTCCTTCAAGCATCCCCT[A/G]GGGGTCTTGGAGTATATCCCCTGAAAATAAAGGGGAACTACTGCATTTCATTTTTTATAAGAATAGAGGATGTTTTAAAACAAACACTTCATAATAAAT
4	37641044	BES3_Contig263_962	0.28	0.31	TTCTCTAGGGATAGAGACAACAGAATATATCATTTAAGAGTTAATTTCTAATTTGCTACCCCTGGGATACTGAACATTCTTAAATATTTGGAAAGACATC[A/G]TTTCACCCTTAGACTTACTCAAGGTGATTTACTATCTTTCTAAATGCTCTTGTGCAATTAAAATCTACCTTATATACAATATGTATATTGAAGTTGAATA
4	38777810	SCAFFOLD195063_3960	0.49	0.47	TACTTAGAGGGTGTAATCACTGTGCCTGCCCTGCCTTTATTCTGTAAACAAACCCTTTAACTTCCCACCCTTCCCTACAGAACAGTTCTCTGACCATGGT[A/C]CTGAAACCACTCCTGCTTTATGGTAACCTAACAATTTTCATTAAAGATACTGGAAATATGAAAAATCTTGATGTAGGTGTAGGCTTTTATCCCCTACTTC
4	45229417	SCAFFOLD105877_5049	0.28	0.45	GTATTAGTGAAGTGAAAGGGCTACTTTTTCTTTATGAACCAGAAAGGTGACCTGCTGATATAGACGTGGGAATCAGTTAGCAACCTGCTTAATTAAAATG[A/C]TTTTTCTTTAGTTCGTCTGTGATTAGCTGAGTCTAAGTAGTTAACTGGTTTAAAAGAGAAGGGTCTGTTTAATTGTAGAATTATAAGATAAATTGCTTTG

4	47362414	SCAFFOLD322436_6046	0.05	0.08	AGGCCATTGTATTATCACTTGTCTAACATCTCTAATTTACATTTGTGCTCTGACACAGGGAAGAAATGAAATGTAACACTTCTGGATACCAGGACTGTTT[A/G]TAATGACCCAAAACAAATTTAATCATCTGACTTTCTTGCACTTACTTATTTTTTTAAACTCCCACACTTCTCTTAAGCCTCAACTTTTTATGATGTCAAC
4	51406351	BES8_Contig223_1384	0.30	0.24	ATTGTACGCTATCATTATCTATTCTTTCAGGGTTCTGAAAAGGCTTTAGAATCTGCTGATTGTCAGTCACTAGCTACCGGATGCCACAAAGCTCTGCAAT[A/T]CTACTGGACCATGGCAAATGTAAAAAAGATAGTTAACTACTGCAAATAGCACTTCTGTTGCCAGATAAATGGCATTACAAAGCTGGACTTATTGTGCACA
4	52280010	BES2_Contig536_931	0.16	0.26	GTTTTGACTTATATTTGTCCAGAAAGTACTTTGTTTTATTTACTCTTTGTTTGACTCTAATGTAAATGAGTTCAGAGAGAACAGAGGACCTATGAACACT[C/G]TCTGTGCTGGATGCCAGGACTGGGTTGGGAGTCTTATCCCCACAGGTCTCCTGGGCATTTGATCTGAGCTTTCGTTCTTGTTGGGAGGGACTGGCTCTCA
4	53718968	SCAFFOLD140124_7042	0.44	0.37	GTATAAACTTGTCATCTAAGGAAGAGCAGGAGTTGAGGTGCTCAAGATGAACAGGTAGGTGCAGGATGTATAATTTCCCATTTCAAAATGAAAGAAGAGA[A/G]GCTTGGATATAATTTGTTAATTATATCTGGATGGTGGGAGTCATTGAATGGGGAGAGGATTAAAAAAACGGGAGAAATTACAACTACTGAGTGTCTTTAT
4	54568493	SCAFFOLD110072_26327	0.30	0.21	TATATTTGTACTTAGAGCCCTCTTTCTCAACATAAGAGAAGTTTATTTTCTATTTCTAGAAAATATGAACCTTTAGGTCTAAGAGAAACCCCAATATGTT[A/T]TTCAAGTTTATAACTTTGAGCTTTATTCAGCTATTTCCCCATTGAATTAATGAGATTTATTAATGGAATTAGAGGGGGAAGAGGTATATTGACCTAGATA
4	54727829	SCAFFOLD20938_6775	0.33	0.46	TCATCTTGTATATTCAAGTTCCATACATATCTGCAAACAGTTGACCCTTGTCTGCATCTTGGGCCTGGGGAGAGCACCTTGGTGTCATGATCTTCCTTCA[A/G]GAGGATGAACCTCAGACACAGCCGGCACAGGATCAGGGCATTTGGGAGAATATTCCTGGTGTAAATTACCTGGAGTGTAGTTTAGAAGGGAGATCCCTTT
4	54777102	SCAFFOLD110333_731	0.16	0.36	ACCTCATGACACTGTAATACATCTAAAAGCAAGATTTCATGAATAAGACTGCTTAGGTTCCAATTCTTGATAGCTGTGTGAATTTGACCAAACTACTTAA[A/T]TTCTCCATGTCTAAATTTCTCCATAGAGTAATATAAAAGATTTAATATTTTTAGTATTTAATATATTTAGTCCTTAGTGTATTTAGTATTTATATAGAGT
4	56008310	BES8_Contig586_984	0.23	0.27	CTTTTTTTTTCCTAAGTAAGATCTGCCAAATGTTCATGGACAAAAACCTCTCATTGCAAACTGAACTAGTGAAATGACAAGGGTAAGCCACGTTTCTTTC[A/C]ATAGCTTTATGGCATTAATAAAGCTACTGTTTGAATGCATGTTAATATTGTCATGTGTAGGATGAAGTCTCACAAATTCCCCAAAATGTGAATGATCAAT
4	56448725	SCAFFOLD147673_1155	0.37	0.48	AGTCTTATCAAAATCATCAATGCTTGTGTGATTTTTGCCTATGGTCTTAAAAAACTAAACAAGACTGTTGCAAAACCCAAATGTCCTAAAAAGCTACTCA[A/G]TTTTATACGTAAGAGATTATGTGAAAACAAAATGTGAGCTATCTGTAAAATACAGCTAAAAAATTTAATCCAAAAGAATTAAAGCAGGGAGGTATTTTAC
4	57090374	BES8_Contig580_3561	0.12	0.15	GTTGTAATTATTTATCAGTAACTGTGCTCCTGAGTATTGCCTCTTTTGCCATCATCTGTTCTGTCAGCTCAGCTGAACAATGAGACATTCACTTGGCCAT[A/G]ATGAGGGGAAAATGAATGATTCTTATTCACCGCAAAATAACACTGGAAGAATTCTGGCAGTGAGGCAAGATTCTGGGCTCTCCATTTAAACTTTGTATTT
4	59252723	SCAFFOLD225500_2165	0.44	0.29	AGAGTTTTTAAACATTTATTAAGAAACACAGTCTATAGTCAAAATGAGATTATGCTTATTATCTTCAAAGAATCTGAAATAGCCTATTATATATTTATTG[A/G]CTCCTAGGACATGTTCCAGCCAGATAATGATCTTTTGTCAAAATAAGAGTGATCCAGTAATATGGTCCTCTTCACCCAGGCTGACAGCAACTCTCAGAAA

4	59974296	SCAFFOLD300275_14801	0.12	0.10	CTTATTCCCTAGCCAGGAATCATACCCATGCCCCCTGAATTGGAAGTACAGAGTCTTAACCCCTGGATGGTCAAGGAAGTCCAAGTAACTACATTTTTAC[A/G]AAGGCTTATACATCGAAGGCTTATATGTCAAAGTTTGAGAACTGTTGCTGAGCGTAGAGCTTTAATTTGGATTTTTAAAAACAAGAATGATGGAAATAGT
4	60064362	SCAFFOLD145312_4540	0.38	0.29	AAATTCCTAATGATACCAGTGATGCTGGTTTGCAGATCATACATTGCGCAGCAAGACTGTAAATGAAATACTTTCAATATAATAGAAACTTCCCCAGCCA[A/G]GATCCCTGAATGACTGTGTGGAATAGAGCCTTACATTTGGCATGTAGTATGAGTGTGATATAAATGTGGTATGAGCTATGCAGAATATCAAAGACAGTGG
4	60339249	SCAFFOLD100431_15454	0.46	0.41	AACTGAAGTAGGAAGAATATTTTCACACCACATAGGAGGTCTCTATTCATTAACTTTAAGAAGAGTTACTAAGAGTCTTTTTTGCAATCAGTGCACACTC[A/G]TGACTTTCTCTATTCTTTTTTAATCACTGACAGTTTTTTGACTCCTTCATGAAAGAACCTATCCTAAGAGGTTTCCTAGGTCTTTTAGCTAACCAAGATA
4	60740831	SCAFFOLD105325_12511	0.20	0.29	AATACAGCTGTTAAAATTTGTAATTTTGGCTTCTCTTCATCTTTCCTTCTATTCATGAACAATTTGGACTCAAAATCATGAGACAGGGAAGAGAAAGGTA[A/G]GCATTTTAATATCTGTATATTGAAAACTAAAATTTTGTTCCAGTATCTTCAAATCTAGTCTAATACCACAGAGTCTATTTTACTTTATCCCTTTCCAGTT
4	65442851	SCAFFOLD70177_16088	0.32	0.41	TCTATTTAGAGCTTAATCAGCAAAATGAAAATGAAATCATTAAAAGGTCAATAGGGTCAGTGCAGTTTCACACTAATTGCCGAAAACTTTTGTCTCTTTC[A/C]AGGTGGAGAATTTTAGAAAAGAGAGAAAATATAGGAGCCTCTGGATTCCAGGGGTCTTTCTAACTAACATTCCCACTGACTCACATCCTGAAACGAGATC
4	65611733	SCAFFOLD1218_3655	0.16	0.21	TCATATTATAATCTGTCACTTAGACAAACACCCATGCCTTTTGCAAATCAGGGAAAGTAAATTATGTACTTCTTGGGCAACTAGTAATAACTAGGCATGG[A/G]GTTAAAGAATACTGATCATTAAGTGTTGAAATTTAGAGGAAAACAAATTAGAAATTTGAACTGAAGTGTGCAGAATACTACAAATCTTAAAGAAAATAAA
4	65918329	BES11_Contig255_1065	0.38	0.26	AAATAAATACTCAGTAAAAACAGAAGAAATTTCAAATGCAAGTACTGTATAACAATCCATCTAATATCTCTGCTGGATTAGGTTATAAGAGAATTGATGC[A/G]ACTCTGCCAAAGGAATGCAAAATCGATAACAACTGTTGCTATCTCACTGCCAAATTGTGTTCTACCAATGATGTATCAGAAGAAAGACATTACACAGATG
4	67040318	BES2_Contig362_1265	0.50	0.33	TCCAGCAACAAGATGTGTATGTTCAATTCTATCATCTCCTTTCTTAATTTTGTCTAACAAACTGACACTCTCTGTCTGCCACCCTCTTAAGAGTAAAGGA[A/G]GTGTCATTTTCTATTCCATAAGCATGAACTGCTTTGCCTAGAGAAGCAACAAATTAATTAAGTTTAAAGTTGAATGATCCACAATTAATCTAGCTGGGTT
4	67056698	SCAFFOLD29708_3468	0.49	0.38	GGGACAGAGAGGCCTGGGCATGCTGCAGTCCATGGGGATGCAAAGAGTAGGACATGACTGGGCGACTGAAAACAGTGGGCAGGTAGTGGACAAAAGTATC[A/G]TTCATATCCTGGGTGGAATGGAGCAGGATGGCTCAAGATTTCATCATGCTACTCAGGATGGCACACAATTTAAAACTCATGGGTTATTTCTGGAGTTTTC
4	73006642	SCAFFOLD50887_11878	0.26	0.26	CCAGAGAATCCAGTGGACATGCCCTCCTTCATCTCTCACTCACAACCCCCAGGTTATTAATTAGACTTTTGCCATCTCTGCTCCCAAGATTTTGTGAGCA[A/G]TAGGCAAAGTACCTTCACACATAATGAGCCTGCAGTTACTGATCTAAACCTGAGACTCACACCCTCAAGACCGTTTCCTCTCAGGATATTTATTAGCTCA
4	73006712	SCAFFOLD50887_11948	0.41	0.23	TGTTTGGCCACGGCTCTGTTCCACCCACCCACCCACCAGCATACCTCCTTGGTATGCTGCGCCCCATAGGCCAGAGAATCCAGTGGACATGCCCTCCTTC[A/G]TCTCTCACTCACAACCCCCAGGTTATTAATTAGACTTTTGCCATCTCTGCTCCCAAGATTTTGTGAGCAGTAGGCAAAGTACCTTCACACATAATGAGCC

4	73006976	SCAFFOLD50887_12212	0.42	0.23	GGCTCTGCTTTTCTCTCTCACCTGGGTATTCCCGAAAGACCACCCGCGAAGTTCCTGACATGTCACTCTCTCTCATTTCTGGGTCTTGGCACTTGCGGCT[C/G]CTTTCTGTCTAGAAGGCTGTATCCATTCTCGCCTCCCCGTTAGTATGGTATTATTACATTTGGGGGGCTTCCCAGGTGGTGCTAGTGGTAAAGAACCCAC
4	73335945	BES8_Contig557_1050	0.23	0.30	GGCAATGAATGGTGAATGACCCATGCCATTAGTTCATACTTGCAATTTGAGTCTAACAGCAGAGTGGAAATATGGGTTTAGAACCATGTACACTTAGACT[A/G]GAATTCCTATTTCCTCACTTTGTAAAATTTAAGTTATCATGGGAGAGTCAAATTCACCTATATAAGACTTCAGTTTCCTTGTCTGTAAAATGGGAAAATA
4	74071828	NPY_499F3-SNP1	0.24	0.32	GTATATGCTAGCTGCCTATCAAGCACAATTAAATCTAAATTTGGAGGAAAACGTTCCTATTATTGCTCCTTTGCAGGG[A/G]CTCAAGGAATACAAAAGCAAAGCCAACTCTCTCGCCCCCATCATTTGCGAGATTTCCTCAAAGATTTTCTGGTGTTGTGATATCAGACTGTAGGCAGCAA
4	74074200	NPY_201R11-SNP7	0.42	0.28	GGGTATCTAGAAAAATAACAGCACAAGCTAGGGAGGAAACAGCTGGGTCACCAAAGACATTGCCAGATTTTTCAGCCTGCCCAGACTGAAGTGAAAAGAC[A/G]TCTAGAACTATTTCCCCCGTACAGTGTTTTATTTCGGTCTGAACCCCTTACCTGAAAATCCTGCTCCTAATAAGTAGAGATTTAGCATGAAAAC
4	74074573	NPY_109F11-SNP5	0.05	0.10	GTGGGCTGCGCGGGACTCACGACTCCGGGAGCGCCCCACTTGCACACCCGGAGATCATGGGCATCTTGAAGGACAAGGTCTTTTTCTTTTTNTTTTTTGT[A/G]TCCCAGGGCCAGACAACATCAGGCACATACTCAGCTCTCAGTAAATGTTTGCACAGGGAGCAACTACCTCGGCCACTACTGTCACAAAATCCTGATCCCC
4	79316649	SCAFFOLD86356_3447	0.42	0.07	ATGGTCAGGAACTGCTCCTCTTCTCCAGGGGCTCCACCCACACGTGGTCCTGCACTCAGTGGTCTCCCTCTTTTGTCCTGGGACGTCACCCCCACCCTGC[A/G]GCTCCCTGGACTTTGTAGGAGAGAGAGGACAGGGAGAGGTGGTGACTCCTAGAGTTGGGCCACAGCGGGGGGCCAGGAACTTCGGGAGATTAAGTGCTGC
4	79541329	SCAFFOLD278390_1226	0.49	0.02	TGAGCAAAGAAGCCTCAGGACAAAGTCTAATTGCTGATGTGGCCTCCTTAGCCTGCTCTGCTGCAAAGACCCCAGAGCCACCCGTTACCTGGTTGTTGAC[A/G]ACCACGTGGACAGTGCCATGAGTCGTGTAAGATGGCAGGTCACTCAGGTGGAAGGTCTCATAAACGATGCCCTGGCCAGCAAATGCGGCGTCCCCATGCA
4	79916489	SCAFFOLD160136_3183	0.49	0.46	ACAGGCAATGCCAGGACAATGGGGATGGTCCCCAGCGCAGCTGTGCTCTGCCAGTCTGGCCCTCCCAGCTGGACCCAGCAGCATCCAGCCTCAGACTTTG[A/G]ACAGAGGTGTATCCCTGGCTATAACTATGGTTTCTAGCTTCAACACCCAAAGTGTTCTTTGCTGCCTCCCAAGTGTCCTGGCCACCCAACTCCTGGACCT
4	81872380	BES4_Contig204_631	0.49	0.44	GGAGAAAAGATCTACGTATGTATATATTTTCCCAGAGTATTTTTTTTTTAAAGGAAGAAATTTTCACGAATGCAATTACAGTGTTTGGTGCTTTTTTTTT[A/G]AAGTCATCAGCAAATTCCTCAAGAGCACTTCATTCTCTTAAGAGGACTGGATTCCTAAACTATGCTGGTGCAGAAAACAATCTGGCACTGCCCACTTGTC
4	81939355	SCAFFOLD201086_2241	0.44	0.44	TTGATGTGCTCATTTCATTATACATATATATGCTGCAAATCACACAAGCTCCTGAAAAGAAGTAATTTGTAGTTAATGGGAATCAATTCAACCCAAAATT[A/T]TCTCAAATTTGGCGGTGAGGTCTCTCAGCATTCTGGAATCTTTACCTCATTCCATTAGGGCATGCCATAATTTTAGGCAGCACTCAGTCTGTTCTTTGGC
4	82141967	SCAFFOLD136007_3193	0.34	0.36	AGTTTCAGGCTTGACTTAAATATCTCCTGCTATAAAATCTTGGAATGGAAATCTTCCCATAGTGCTTTGTGGTACAGCATTCGTTTATACAGGTGTGTAC[A/T]GGGTTCCTACACTATTTCCCACCTAACTAGATATAGCTGCTGGCTTTGCTAACCTTTCATTACCTACTCTATGATAATATTCTTTTCATCCTCTGTGCTA

4	83416542	SCAFFOLD85346_7303	0.38	0.37	TATTTTTGCTGCTGTCCAACCAATAAATTTTCTGTACCCCTGACACACACCTGAATTTTTAGAGGCAATCTTCTAAACTAAAATTTACACACACAGTGTA[A/C]ATTTTCGGAAGCTTTCCTCCTGAAAAGCCAATTTCTCATACACCTTGTAACAAACACTGACCACATCCTAACCAAAGCTAAGTACTGTGTAATGCTAAGC
4	84186147	SCAFFOLD317185_4049	0.46	0.37	TACTTGTTATACCCCTACTGTGTGTTCAGGAATTCTATTTCTTCATTCAGTCAGAGGAATCAGGCTCTGGCAAATCGAGTCATATAAATTCAAGAGACAT[A/G]AACAAGCTAGACTGATTGCGTCACTTTATTTGTTAATATCTTTACAGAGGACAAAATCCCCCTAGACATTCAAGACACCCTTCCCAGAACAAAAGGTACT
4	86439991	SCAFFOLD101100_3645	0.10	0.16	GGAAGTTTTCTTAGAACTCATGATCACTTATGGTATTAGAACTGGAAATTCTTTGCAGATAATGTTACTGAACTCATCTCCTAGAGCAAGATTTTGGAGG[A/C]CAATATAGAAGCAGAAGAAGATAAATGACTCCCCACCTCCCCTCAATTTTTCCAACTGTTTCTTTGCTCTTTCTTTTTCCATTGTAATTTCATTTAGATA
4	87760448	BES10_Contig535_1147	0.36	0.32	TAAAGGTGATGGGAGGGCACTGGCTCTTCTGGTCTAACATAGCTTTATCCTGTTTACTGAAATAGTTCCAAGCAGTACCATGCCGGGATGATACATTACC[A/G]TCCTTATTGCTGTCAGCACGAGAGCTCTTTTGGTTTGTCTTTTGAGGGCTTAGTAGGAATGTACAATTATTTTTTAACAGTAAGTGAGCAAAAACTCAAA
4	87907647	BES10_Contig752_2751	0.24	0.31	AAAAGATAGCAAGTTCTTATTAGTTTTACTTTTTTCCTTTTCTCCTTTTGTGCAGTAGGTAAAACTACCAAAAGGAAAAAAAGAAGGAGTGTCCATGCCT[C/G]GAATTCATCTCTTTCAGTGCTATCAGTGTAGAGTTTTCACTGAAGCCCAGGGAGGCTGACAAATCACACTCCTGAGCCGCGGTTGTTAGCCCTTTCTCTT
4	90418034	SCAFFOLD220733_6491	0.31	0.35	CTAACACTTGACACAACCTAATACTGAAGGCTTAGACAAGTTTAGTTAAATGAACTTAATTTCTCATTAGTTAGTTAAGGATCATGGTGTGTTACCTCCC[A/G]GTATGAACCCTCATTTCCAGCTATCATTTAGTTGTGCTTTGCGTTATTTTCCATGCTACCATCTGTCACTGGGCTACGATCTCATCAAGTTTCCAAGTCC
4	90418076	SCAFFOLD220733_6449	0.40	0.48	TAGACGCTGTTAGAATGGGGGCCATTAGAAGTCCATCCAGCTGGACTTGGAAACTTGATGAGATCGTAGCCCAGTGACAGATGGTAGCATGGAAAATAAC[A/G]CAAAGCACAACTAAATGATAGCTGGAAATGAGGGTTCATACCGGGAGGTAACACACCATGATCCTTAACTAACTAATGAGAAATTAAGTTCATTTAACTA
4	91217499	SCAFFOLD152066_1487	0.37	0.31	AAGCAGAGAGAGCAAAGACAGAAAAAAACAAAACAAAACAAAACAAAACACAACAGAACAGAATATCTAAGAACTGTGAGAGAGCTAAGGTGTAACATAC[A/C]CATTACAGGAAATCATGGGAGACTGTTAGGGGATTTTGCCCCTCTAATCATGATATGTGACTATGACATCTCTATATTCAAGAAAGTTTCAAAGTGAGTG
4	94612980	SCAFFOLD10817_8267	0.36	0.45	GAAGGAACCAAGTTCCAGTTGACTTGGTAGTATTTTTTCCCCCATAACGGCATAAAAGAATGGCTTATCGTCTAATCTAAGGTGCTACATTAGATTCGCT[A/G]AATATGGTCAATAGCGACAGAAGACTGCATCATTCTTTTGCACTTTCTTTAAATCTTGCTTTGTCATTGAAAGTGAAAGTTGCCCATTAAATCTTGCTTG
4	95382363	BES9_Contig569_554	0.11	0.22	TTGTGCCCAGATGCCTTCGTTCTAATCCCGTATTTGTGGTGTCCCCAGAAAAATAAATCTCAGTGTGAATATTTCACGGCCATCTTCCTAGCCTGTTGGC[A/G]TTTTCCACCAGCCCTATAGTAGTGATGGTGGCCAGCAGCAGTGCCCAGGACTGGGCTGCCCACCACTCTCCCCGCCCTGAGAGGGAGCGTTCTCTATTTG
4	95689996	AY138588-252	0.11	0.08	TGGGTGTTCTCGGAGATCGACGATGTGCCACGTGTGGTTTCTTCTGTTTTCAGGCCCCAGAAGCCCATCCCGGGAAGGAAAATGCGCTGTGGACCCCTGT[A/T]TCGATTCCTGTGGCTTTGGCCCTATCTGTCTTACGTGGAGGCTGTGCCCATCYGCAAGGTCCAGGATGACACCAAAACCCTCATCAAGACAATTGTCACC

4	95691972	BTA132764-140	0.32	0.27	GGCGGCCAGCAGGTGGAGAAGGTCCCGGAGGTTCTCCAGGTCATTGGATATTTGGACCACATTTCTGG AAGGCAGACTGGTGAGGATCTGTTGGTAGATC[A/G]CCAATGTCTGGTCCATCTTGGACAAACTCAGGA GAGGGTGGAGCCCAGGGATGAAGTCCAAACCAGTGACCCTCTGTTTGGAGGAGACGGACTGCTATGC
4	95692129	BTA132764-29	0.19	0.13	CRGGACTGAGGTCCAGCTGCCGCAACATGTCCTGTAGTGACCCCTGCAGCCGGCTCAGGGCCACCACCT CGGTGGAGTAGAGGGARGCTTCCAGGACRAC[A/G]CCCAAGCTCTCCAAGCTCTCCAGGGCCCTGACCT GCGGCAAGGGGCAGCTCTTGGAGGCGGCCAGCAGGTGGAGAAGGTCCCGGAGGTTCTCCAGGTCAT
4	95692144	BTA132764-312	0.19	0.14	CAAGGCTTCAGCACCCRGGACTGAGGTCCAGCTGCCGCAACATGTCCTGTAGTGACCCCTGCAGCCGGC TCAGGGCCACCACCTCGGTGGAGTAGAGGGA[A/G]GCTTCCAGGACRACRCCCAAGCTCTCCAAGCTCT CCAGGGCCCTGACCTGCGGCAAGGGGCAGCTCTTGGAGGCGGCCAGCAGGTGGAGAAGGTCCCGGA
4	96600291	BES6_Contig314_6 77	0.36	0.49	ATTGCCAGTGACTGGCTGCATGTGTATGTGGGTGGACATTGCTAAGGGACAAGCTTGCCAAAGGTGAG AGAGAGACAAACCTCCAGACAGACTCACAAGT[A/G]GGAACTCTGGAGGTTTCCCTAGCACAGCCCAG GTTTCTCAGGTTCTGGCTCACTGGTGTGTACTGTATGTGTCCCAAACACCTGGGTCCTTGTAGGCCTC
4	103000079	BES1_Contig603_4 73	0.18	0.22	CCTGTCACATAATTTTTCATACATTATTTCTTTCAGTGAATTATCCCACGAGCTCAGAATTCAATACATAA CCATTTAGCAACAAATATGAAAAAAAAAA[A/T]AAATGGCTATGTGTCTTGTGGCGATACCTGGGTCTT GCAGGTTTGTGGTACTGACAGGTTTCTTTAATTCAAAGCCCAACAGGTAGAGAGCGAGATTGGG
4	103278068	SCAFFOLD20555_1 4349	0.12	0.25	TTGTGTCTTATATAAATTTCAGGTCTCCGTGATATCAGGGAGTGTATGCCCCGATGGGATGGTGTGTTGT GGAGCTGCGCTCTGCCACCCGCACAGCATT[A/G]TCAGTCTTCTTGGTTTAATTTGCTGCTGTTTATGCTG GTTTAAAATGACCTCCTCCTAGGTCTCTCTGGCTCCAGTAAATTACGCACAGGTTGCCGAGGC
4	104925374	SCAFFOLD200500_ 2098	0.49	0.40	AAATTTCCTTTACTCTTCTTCTTTAGAGCCATTTATTTAGACACAAACATCACAAATAAAGCCGACTTTATT TCATGACCCATCTTCACTGCTGTTCTCA[A/G]TTAGATCCTATTGGCTCGCATCACCCTTCCCATCCCTTTT GCCAGGGATGTGGTCCACATCCTTCAAGACCAAGCACCCTGGGCAGATTTTCCATTGAGC
4	104945421	SCAFFOLD151159_ 1914	0.19	0.28	AAGAGCTGGACACTACTGAGTGACTGATCAACAACAAGAAGCTGGTCTCAATAAAGGATGTTTTGATTT GGTAGAGTGGAAGATAATTTGGGAAATTATA[A/G]TCCTCATCAACTCATTTCTAATAAGTGTATTTATT CATGTGACTCCTTTGGGTCAAGCACTGGGATGAGCTGACCAGTATGCATTTTTGACAATAGAGGG
4	105269938	SCAFFOLD283606_ 592	0.43	0.45	AGAATGCTGCAGGTTTTTTCTTTCTCAGGCTCATTCTTCTTTTGATTAGAGGTCTACCCACAAACAGCCCA GGAACTTTGGCCTTCTGTCCTCAATGAAG[A/G]GAGGGTCCCAAATCTGTGGGTGAGTTCTTTCCTGACT GGACCCTTTGGAGCCCTAACTGTATTCTCAGATTTCTCCTGACAAGAGGTTAGATGCATGATG
4	106698262	SCAFFOLD146712_ 2778	0.43	0.36	CTTTGCCACAAGAAAGTGGCAGAGCTGGAATGTGAACCCAGGTGAAGTGGTTTACAATGCGGACTGAG AGGTGTTTATGGACGGAAAAAGAAGGATTCAC[A/C]CAGCGTACTTGCCATGGGCGCCACCATGCAGC TCAAGGGCATCAACCTTACAACACGCAGCCCCTGGGGACGGCAGAGCTGACCCAGATGGGGTTCAGCC
4	107076639	SCAFFOLD140875_ 7899	0.43	0.28	AAACTGCGATGATGACTGGAATTCTGCAAAGACGTTTGCTCTGAAACAGTCGGAGCTACGCATTTACTC ATAAGCTGGTGGTATCAACTTAAATTTTTTT[A/T]AATTGTAGTCATTCCCGAATCATATGTCTTCTCCAA GGCAGGAAAGGCCCAGAGGGAATAATAACCGCATCTGTCTCCGCCTTCAAGATTCATGCATTAT

4	107080502	SCAFFOLD140875_11762	0.15	0.16	ATAAAGATACCATTTGTTTTAACTGGGCAATTGAATAATGCTGTAAACTCCAGATGAGCAAGGACTTCCTTCTTTGTGGAATTTCCTTCCCAGACTGAC[A/G]ATGGTGCTTTGCCCTTGATGACTACTAAGCTAAGCAGCCATACACTCAGCATCACCTTATAATAAAGATAGCCAATGTAAGAGGTGAGAGAAGCAGAATG
4	107080581	SCAFFOLD140875_11841	0.16	0.16	GAATTTCCTTCCCAGACTGACAATGGTGCTTTGCCCTTGATGACTACTAAGCTAAGCAGCCATACACTCAGCATCACCTTATAATAAAGATAGCCAATGT[A/G]AGAGGTGAGAGAAGCAGAATGGGAAATTTTTCCCCAGGCTTGGCTGCTGGACGATAACCCTTCCCCCAAATCATTCTTTTAAAAATTAATAACATTAATA
4	108150577	SCAFFOLD146059_2695	0.47	0.09	AGTATACGTGCCTCTGGTAACACACACGGTCATGAATTCTTCACCTCCCATGCCTATCCCTCCAGCAGCGCGGTGGACAATGCAGAAATGACCAAACTGC[A/G]TTCTCTGTCCGTCCTTTCCTCTCTCACCGAGGTTTGGCTCTATCACGTGGACTGGAGTACAGATGGTTTAGAATTCTGAGGGCTTGAGGGGTGAGGTGAC
4	108150720	SCAFFOLD146059_2552	0.47	0.10	GATAGGCATGGGAGGTGAAGAATTCATGACCGTGTGTGTTACCAGAGGCACGTATACTTGGCTTTGCAGTGCGTGGTGTGATTGCTGGCAGCAGGAGCAG[A/C]TAATTTACCTTCCTAATTAGGCAGATATTCAAATGAGTTGGCACTCCCTGAACGCATGCCACCAAATTGCAAGGCCCTGTCAAAGAAACCATGTCCATCT
4	109231937	SCAFFOLD75114_7252	0.08	0.13	GCAAGAGACCCAACATGCTTGGGAGAGAGCCACCCTCAGACTCACCGAGAAATAGACATTGCCGTCAGGCGAGACGCCTCCGACAACCAGGTCGCTTCCC[A/G]CTCTGCTGTAGCGATCTGTGACACCCAAACCCACCCAGCCAGCTGTCCGGACCTGGAGCTCGAAAGTGATGATCTCAGCCTCAAAGTCAAAGTCCCAGCG
4	110063081	SCAFFOLD161053_2988	0.30	0.44	AGCACATTCTTCTAAAGAGGGCAGATTAGGAGGCTTTGCCCAAGTGGTGACCAAGAGCATGCGGTCGCTCACAAAACCTCTGGGTGATCCTAGAGCAGGT[A/G]CTCCTGAAGGCCAGGGCAGAACCCACAGCAACAGACTGGGGACACCTTGAATCCTCAGCATGTATATTCCTTAGCACTGCTAAGAACCACACACCTGATG
4	111399074	BES8_Contig470_1089	0.36	0.25	ATTAGCCATTAACAGAAAGAGGGAAGTTGTGGTGTGCGCTGTGTTCTGAGTCAGCTAACAATCATTTTCAGATTCATCATGTCAAGTCTTTTAATCCACA[A/G]TAAAGCAGAAAGGCTCCCATAAATAGGATCAGTGAACACAGGGTGTCATGCATTATTTCCATTTCTACAGAATTTTATGTAACTCTGATTGCAAAGTTTA
4	111817892	BV103782-242-K	0.35	0.48	AACCTCACTAATAATACCTGCCACAACTCTGCTCCCAAATAAGACTGCATTCTGAGGCTCTTGAGCTTAGCACTTCAACATATATTTTGGGGAAATACAG[A/C]TCACCCATAAGAGTGGAGAAGGCTCAGAGGTGGGCAGCTATAGACTGTATCAGACCTAGAGCAGCCGGGGCGAGTACACYGGGAGGCTGAATTCCCCATG
4	113735155	SCAFFOLD105499_10077	0.20	0.29	CTGATGTTCTGTGAGAGATCATAGCATGATAGGTTTCAAAAGTATTGGATCTATAGACCTAAGAAGCCTAACAATCTTTATCAAAGGCCCTGAGAAGTCA[A/T]GAAGAATTGAAACCCGTTTAATCCATTTTTCTCCAGCTCCTTTTTTCAGTTCAGTTCAGTTGCTCAGTCTTGTCCGACTCTTTGCAATCCCATCGACTGC
4	114098111	SCAFFOLD280379_15486	0.07	0.10	TCGATGTAAGGAAGGACACCATCCTAAGTGTTTAAGAGCCAGCGGTTGCTATGCAGCAATAGTAGGTTCAGATGGTGCAATTAGAGCAATTTTGGTTTTA[A/G]AAGTTTGAAAACTAATTAAAAGCCATATTGTGAAGAAACTTGAATGTAAGTATAGATTTTATCCTACATGCATTCAGCAGCCACTTTTCTTTTTTCGACT
4	114244352	SCAFFOLD121635_632	0.40	0.50	GTGAGGATCAGTGAGTACACTGAGTGTGTGTAGTCTGAAGAAGCAGCAGCAGCTAAGGGGAAACGTGATCTCGAATAACACAAAAAATATGCAACCTTCC[A/G]TTTACAATCAAATGCTAGGTGTCATCCTCATGACTGATGGAGTGAGGAGGTAAGGCGTGAACAAAGAAATCTGTACCACGGTGCTGAATATGGTGTTATA

4	114530113	BES3_Contig465_3267	0.39	0.41	GAGCTAAATGTAGTGCTTTGAAATGCAAGCAGAATACTTTTTAAGTAGGCATTTAATTTTAGTGGAATTTGCATTTGGCAGTCCCAATTCATCTGTTTCA[A/G]CATCTGAGTGAAATAGCCCTCTTTGAATTCTAATGGAATGAATACAGTGAAATTAACCTTTTACCACAAAAAACATACTACTATTCTAGGCGAAAGCTTT
4	115013168	SCAFFOLD141328_8675	0.45	0.37	GAGAAAGAATCTGTAATTACAAGGTTTCTAAAGTAAGCACTCTAGGAAAAGCAGGGTCAGGAGTCCAGAGTCAGAAAGAAATCCATCTAAAGTTTAGTCA[A/C]GTTGAGTAGAACATGAAGGCTATTTTGGTCAACATCTACTTAGTGGCTACCCACTCCAATATTCTGGCCTGGAGAATTCCATGGACTATCCATCAGGCCA
4	115385991	BES1_Contig459_655	0.17	0.24	CCGGAACCACTCTAGGTAGTCTCACACTGCATTATAGTGAGATATGTATTGTTAAAAGAGGACAAATTATGTTTTCAAGGATCCGTCAAACTTCTCAAAT[A/G]TAAAGTGTGATGTTTTTCTGTTCACTTTGTAAAATTCCCAGGTAAGGAAACCAGGTGAGAAGGATAGATTTCTGGAAAATTTATGATGAGTTGCAAGCTT
4	115515467	BES3_Contig393_1300	0.29	0.36	CATATTTTATTCAGAGAGGAAAATAATGAGGCATGTAGTAGACAGTGATTTGCATTATAAAATGTGCTTTGTGACAATACAAGGGGTGGCCGCGGGCAGG[A/G]AGCCGTGTGCAGGCGTGTGAGAGCACAACAGCCTCTACGGTGAGTGCTCCCACCTCCTACGGTGTCTGCTTTGAGGAGCTGGATGAGAAAAAAAAAAAAG
4	117126683	BES4_Contig479_1305	0.20	0.15	GGTGGCCAGGAGAGCGGTGGTCTTCTGTGTGTGTGTTTTGAGGGGCACTTAGCTGGTGAGCAGCTAGTACATTGAGAAGAACTTTTGACAGTGAGGAAAT[A/T]TACAGTCTAGTCCCGGTTCACCTTGCTGGGCGACCTTGGGCAAGTAAGTCGACCTCACTGAGTCTTGGTTTCCCCATCTATAAATTGGGGTTCAGTAAGC
4	117126810	BES4_Contig479_1178	0.24	0.28	CTCAGCTCAACAAACACCCCCAAGTTCAGTTCCATCTCCTTCACACTGCTTCTCCTAAGGATCCTCTTTCCCAGAGCACTGTGTCCCTCTTAAATTGTTG[C/G]CCACAACAGCCCCGAGGAAGGGGCAGGCTTACTGAACCCCAATTTATAGATGGGGAAACCAAGACTCAGTGAGGTCGACTTACTTGCCCAAGGTCGCCCA
4	120452315	SCAFFOLD165677_14451	0.20	0.29	GCAAGTATTTATCTAGATTACAAAATAGCCCTTTCTCTAACATACTCCTGCTTTGTTTTCCAAGGAGGTAGGGCCGCTTAATGTGAACAGTTAATGAAAA[A/G]TGGATTCAAGGATCAGAAAGCGTGTGCTGTCTCCATATAAAAATCACACTTTGGCTCTTCCAGGCAGAGAGTTGCCTTGAATTCCTGGAGCGACTACATA
4	121617490	SCAFFOLD160853_1270	0.46	0.40	TTTTAAGATTCCATCTCCTTGGTGTTGATTTTGAGAAGAGCTGTGTGTTGGGGTCCTTGGAGGTTGAGGCAGAGTCCGTGTCACTGCCTGTATTGACCAG[A/G]TGGGCACGCTGCGCTTTGCTCTCGTTAACGCTCGGGGAGGTCCTGTTCTAGTTCTGTGCTTCGTGCTTTTCTGCTCGGTCTGGTAATCGTCATAGTAAAG
4	121617593	SCAFFOLD160853_1167	0.35	0.36	TCATCCTAACAGCCATTAAACAGAAAATAAGTTTTTCTCGTATTGCTTTGGGGCTCGTAAGCACTTTTCCTCTTCATGACTCATGCTGAGCCACATGAGC[A/G]CACTTTACTATGACGATTACCAGACCGAGCAGAAAAGCACGAAGCACAGAACTAGAACAGGACCTCCCCGAGCGTTAACGAGAGCAAAGCGCAGCGTGCC
4	121620283	SCAFFOLD33996_192	0.44	0.46	GTGCCCACCGTTGGAGGAGACATCCCAGAGTTCTCAAACAGCACTTGATAAACGTCTCGTTAATTATGCATTAGTCACATGGCTGTGCCTCCCAGCTAAG[A/G]AGGCTGGCTAATACAGCGTTTTAGTAGGTGACAGCACCTCACGTTCAGCTAAAGCGGGGAGTGTCATGATCAAGGACGTCACAGGGAACGGGGAACAGGT
4	122723671	SCAFFOLD85854_5077	0.13	0.04	ATTGAAGAGCCTAAGTCCTGAGACTTTGTAAAAAGAAATATACATAGAATTGATGTGAAATAGAGATTTGAAATTAAAAATTTGTGACAACAAGTTAGTC[A/G]TATTCTGGTATCTGGGTTTTACAGTCACTTCTACACAAAATACAGACTATACTAATACAGAACGCAGAGCCATAAATACCGCACTGGAAAGCAAGCAAGA

5	1968821	BES2_Contig202_1418	0.41	0.40	AGAAAATATTTAAGTATTGAGTAAAGCTTGTAATTTTTATGTTCATAGATTTGTCAAAAAGATGTTATCTTCCCAGCAGCATTGTGTTATCAAAGATGCA[A/C]ACCTTAAATCAACAGCTGTTACACCTCCAGCAAGTTATGAATTTTTCCTGATTTATAGCAATCATTCTTTTTATTTCTTTTCTCAGTCAAGCAGGGAAAT
5	2784168	SCAFFOLD112034_3244	0.39	0.37	GTCCTGCCTGCAGCCTGTCGTCCGTGATGGGGTGTATGCGGGAACTTTTTGCTTCACATGTGTAAGATTCCCCCATGTATTAAACCATTGATGTCTCTTG[A/C]TGACTGCAGGGCTGTTTCTTTGGTTTTAATGCTGGGCAAGCACAGGGTTTGCATGCCTGCAGGATGCACCCCTGCACTCCCTCTGTTGCTGTCGCTGCTG
5	4206050	SCAFFOLD285252_6607	0.39	0.49	CCCTAAAATCATGGTTCTGAGAAAATATAAAATAATATATTAAACAAATAACTGATCATGACCTTTCAATGCACCTGTAATTTATTCTAACTCCCTACCA[A/G]GACAGTATACGAACAGAGAAAAGATTGATTATACACTAGCTGAGATCAAATGGAAGACAATCTCCGTGTGAAAATAAATTGCAATGTAAACTTGCACGGA
5	4585434	E64H04-38779-1	0.10	0.10	TCTTTACTATAATTTTTGATACTAGTCATTTCTTCTGTATGTCTCTAGAGTACAGTTTTCTTTTATAAATAGAAAGGTTGGTAAATCCTGTCTTTTAGAT[A/G]ATTCAACAATAAAGGTTAAGTGCTTTATTACTGTCAAAATGCATCAGGGATATCAAAGAATATGAGGAACATACTTGGATTCTATTCATCCCAAGATAAT
5	4654466	E64H04-38787-1	0.27	0.15	TATGGACCTATTTCCAGTCAAACTCTGCCACAGAAAATATTAAAAATAAATCCAGAATATATGGACAATATCACTAAAAATTGGACAGAATATTTTTTTC[A/G]GCTATGAATAACTGACCAAAACACTTGCTTTCACTTGCTATTCTCTTCTCATTAAAAAAACCAAACAAAAATCCTATAGGAAAATTATTCATTAATTTTC
5	6591071	E454D12-38941	0.27	0.31	GTTATAATTCTCGGCTGCTGTTCTCTGCTTAGGTTCCTCCTCAGGTGGGCTCTCCCTGAGTATAA[A/G]GTAGAAGAGATGACGGCAGCAGTATTGGGCTTTACTAGAAAGCACCTAACAGAGGCCAGGTGACTTCCTAATACTGAGAATATAAAGATGTTTACGATAT
5	6765455	E454D12-38943-1	0.34	0.44	TCTGCCACTAATTGTCTACTTGACACAGGGAAATGTAGCCTCTTTTTTTCCTAATCTGAAAACTGGAGGAATTGTAGCTTAGATAGTCTTCCCACTCACA[C/G]GATTATATAGAGATAACATATGTGAAAGGGCTTTGGAAAAATAACAAGTAGTCAGAGCCTTAAATTATACATTCCAGCACCTAGAAGAGCACGAGGCATT
5	6765674	E454D12-38943-3	0.31	0.44	ACTTGGCCTATCAACAGTGCTTTCTGTATGTTGACCTAAGGGAAGAGTGATGATTATGGCCAACCATATAGCATTTTAATATTTGAGGAAAAGAACAGTC[A/G]ATACTGCTTCCTTCTCTGCCCGCTACCAGCTCCTTTCCATCTTGATCCGAGGATACTATTTATTAAGAACCAAATATTACTTCACATACATGATCTCATT
5	8176317	BES7_Contig521_4435	0.38	0.46	TGTGTTGCTATTTTAAGATAGAATACATGAGAAATAAGGCAGAGTGAACACAGGAATGAAAGCAAGCCAGTTGCTTTTCCTGAGACTCTCTCCCCTGTCT[A/T]CTTCATGCTTCCCGCTTATGTTTCCACAGAGAAATGGAAGCATTACTATGAATGCTATCAAGAACTATTCTTCTCTTTGAAGGTGGTAATTTTGTTAAGC
5	9087184	SCAFFOLD45033_20771	0.34	0.30	CCCAACTGTAGCTTTTCACAATTGCATCTTCTTTCCAACACTGATTCCAAATGGCTTTTCTCACCAGAAAGCATTAAAAATCTCTCTCCCATGGCTACAA[A/G]TCCCAGAAAGTATCTGATTTCTCAGAGCACAGTCCCTGGAGTCACTTTCTAAATGTTGGTGAGAATACATGAGTTAGTAATATATACCCATTTTGGAAGC
5	9087244	SCAFFOLD45033_20711	0.47	0.47	GAGGGGATGGAATAATAGAGTCCAGATCTATATTGCTCTCTGCAATGATTTATTAGTTTACCCAACTGTAGCTTTTCACAATTGCATCTTCTTTCCAACA[A/C]TGATTCCAAATGGCTTTTCTCACCAGAAAGCATTAAAAATCTCTCTCCCATGGCTACAAGTCCCAGAAAGTATCTGATTTCTCAGAGCACAGTCCCTGGA

5	11110522	PAWR-392	0.50	0.44	AGTCTTAAATACTGGATGGCTGGGGAAGTCACTGCATTGTTTAAGGGGGAAAAAAGTTTAAAAGCTAATATCATTTTCAGCTCCTTCATTTTACCCATTT[A/G]TCAATTAACTGGCAGAGCCAAAAGTAAAATGGAGGATCACTATTTTTCTGTTTTCCACAGAGCAATCTCCTTATCTTAAACTGTGTTTTGAACAAAAGTT
5	12216056	E54B08-34266-5	0.49	0.00	CATAGTATTTATTAAGCCACAGTCTTCCATCTATTTTTTCATTTGTCCTTTACTTCAAAATCCACATCCTCTTACATTTGTCACTTAAGAAATCTGGTAA[A/G]ATTATCCCTGATATTTAAAATTATAAACTTTGATAAAGGTGTATGTTCATTTTATTTCATACTTTGTGCCTTTTGTGTTTTTTTRCAGTGCTAATTTGTT
5	12279101	E54B08-34262	0.48	0.36	AGGCTCCAGTTTAAATCTCAAATTGCTTATCTTTACCGAGTCATTAACATCTTGATTTTCTGTTTCCACAGCCCTGTTTGTTTTGGCTTTGGCCTTAGTC[A/G]AGATTACCATCATTAATATCAAATAAAAACATTTTAATTGCAAACACACCAAGGATTCACTGGGAAGCCTACCATGTCAGTTGTAGCAGTGGAGGTCACA
5	12285672	BOVMYF5A-1948	0.49	0.36	CCAGGTGCTTCTCCACTCCCCAACTTCATCCCCATGAATTGCTAGATATTTGTTGCAAATTTCTACCAGGCTTTCTGTGACCACCTGACCTTTGGGTTTC[A/G]AAGGTGGTGACCTGCAGTTTAAGCGGCCGAATAAGCAAGGATTTTTTTTTTTTTTAATGGTTTTCTCCTTGTGTCCTTAGTATATGCCACGGATAAAAGC
5	12328921	E54B08-34286	0.41	0.48	AATGTCACAGCAGAAGTTAAGCTTGATAATTTAAAAGATAACTGAATTCTACAAAACATTGAGTCTGTATTGAGTGGACATTATGTATAAGCAATGTGTG[A/G]GTGACAAGTAGGATAAAAACAATGAGGCATTGTCTTTTCTCAGTAAGAGGTTATAATTGAGTAGGAGACACAAGACATGTAACACTGATAATTATAATTC
5	12350330	E54B08-34264-2	0.42	0.34	ATAAACATTATCTTCTGTTAGAACTAATAAGAATAGATACAAAACTTAAATTAAAAAAAAAAAACAAATAGTACTAGCGTTCTCTATCCTTGCTTCTAAC[A/G]AACTAAAAGTTGAGCTGAAATTGCCTATTAATGCATAATAAAATTCATGGAAATGGAAAATAAAAAGCCAATTGATTCTAGAACCTGAATAGAGTATAAA
5	12380624	E54B08-34244-2	0.28	0.29	AAAAGAAGCATCTCACCCAGGCACGGGAGGGCCAGAGATGGTTTYTTCTGTGAAGAAAATTCACAGGCAAGTAGCTGTTGGGTAGGTGAATGGAGGCAGT[A/G]AGTAAGGTGAAAGAGCCATGTCAGAGCAAACAATCCTAGAGTCCAGACATGAGGAAATTGCCAGTTCATCTCTTTGGACCCTTTAAGTGCAAGGGGGGGG
5	12398549	E54B08-34274	0.43	0.43	ATCTGCCTCAAATAAACCCTTTTTTCAAGGTTTCAAGGTTTCAGGGTTTGGAACATCTGAATGGACCAAACTAGGATGTTATCAAACTTAACC[A/G]AAGAGGTTGTATGAGTCTATTAATGCCTTTTTGCTAATAAATGTTTGCTAAAATATGCTTTTATGGACAGCAGACTTTGCTTTAAAACTTACATAACTAG
5	12423627	SCAFFOLD145116_14148	0.27	0.34	AGCATTTTGAAGTGTTCTCCCAAAAGTTAAGTGAAGTTCAGTTCATTTTCAGGTCTAGGCTTTTTAATAAGAACTTGGAATTCCTATACAGTGTTAGTGT[A/G]TAACTAAGAATGCAAAATGGTGCACCTGCTATGAAAGACAATATGGAGCTTCCTCAAAAAATTAGAAATAAACTACCATATGACCCAGAAGCCCATCTTC
5	12423781	SCAFFOLD145116_14302	0.24	0.30	AATGAACTGAACTTCACTTAACTTTTGGGAGAACACTTCAAAATGCTCCTCTGAGGATAGGGCAACAGCAAACATTCACTTAAATACCAAAATATATCTA[C/G]AGTTTAGGTTGGAAAAATCTCTGCATTTTTGCAAAATTTCAGATTTTTAAAAAAATAAAAAAGATTTGTTAAATTATACATCAATTATACTTTAATTTTTA
5	12451776	SCAFFOLD260110_17851	0.34	0.40	GTCTAAGCAGTTAAAACCAGAAAGCTTAAGATGCAGTTACATTCTTAACTAGTTCAACTGTTTGTGTGGAAAAGACATGCTTTCTATTCAGAAGGCATAG[A/C]GCAGAATATAGTTATAAAAATATTAGTCCCATACCTCTCGAATAGCAGTACAAAACTCACTCTGAAGCACTTTTTGAGGGATTGTAGCTTGTGCACTGG

5	12455145	SCAFFOLD260110_14482	0.32	0.41	ATAGTCATTTTCAGCTTTCTGAATTTCCAGTTCAGTCACTTGATACCCTTTAGCCTACACATTCAGGCTTTCATCCTTCCTGCGCATCAGACCCAACCCA[A/G]TCTTGACCAAAGCTGATAGTGATCTGGAGGCTTATTCTCATGAGAAGTTAGTTATATGTCCACCTAGACATAGGCCTCGACCCCCTTCTTTTCTAGGCTC
5	14827654	BES11_Contig478_2145	0.42	0.40	ACATGGAGGATGTTTGCAATGATAAGAGGATTCCAGCAAGGACTTGTCATAAGCAATTGCATGCAATTTCGTTGAGTATGAGGTTGTGGGTGTGGATTTT[A/G]TGCTTGACATAATGTCTCTGCTAAATTACTGACAGTGGTTTGTGGCTGTTCTGACTGCTCAGGAATATCAAGCCCTGAAATATATTACACTTCTTTTTGA
5	17037386	E249A20-36347-1	0.33	0.27	TTCTMAAGTGACACTTAATTGCAATAGCAATAAATTCTTAAAAATAACTGTAAATCTAAATTTCAACACGAGGGTTAAGAGTCATTTTTACATAAAAATC[A/G]TAGGTTACCATAAATACACAAGGAGATATCAATTTAGGTTTACAAAAATACAAATGTAAAATAAGCTATTTATTCAAAGTAAACTATCTAAAAAATTTTG
5	17080200	E249A20-36331	0.38	0.26	TAAAGAAAAAGCAACAGAAAACTTGTTTTCAGTCATATTTGCTGTTCTATTACGTTTAAATATTCCTGTTTAATAAGTCCATAATTATTCTGAATGTAGA[A/T]ACAGGGAATACAATCTGCCACTGCACTTATATATGCAGATTTTTTGTCCTAGAAGTGAGTTGGACAAGCTTCAAATATAGTAAAATATTTAAAATTCCAG
5	17129722	E249A20-36341-3	0.11	0.20	GCAATACAAAAATCTGATTTAAGGGAGAAGACCTCACCATTTCATATTTCTGGAACCAGTCGTTTCAAGAACCCCAACCTGTGTATGAAGCCATTGGTAC[A/G]TATATTCTTTACTCCTTTCTAATTTTTCATTTGCAGTGGTATCCTTATACATAAATTCTTCTTCCTTATCTAGGGAAAAATATGAAAATATTATTTGTTT
5	17272101	SCAFFOLD151901_11629	0.18	0.22	GACAGATGAAGCGGGTTATCAGCAACTAGCACAGGGAGGCCAGCTTATAACTTTTCAGTTTACAACAGGATCACTCCAGAATTCCACTATAAATCTGGAC[A/G]CATGTATTTTGCATAACTTGTATTTTTTATATAGCTTCTATTTTTAAGCATATATATGGAGTGGGGAAGTGAGGATTTTAAAAGTACCATTTTGTATTGT
5	21557079	SCAFFOLD155471_12240	0.32	0.37	AGGAGCCTGGCATGTGTGTAGACAACAGAGAATTCCTGTATTATTAACACCTCATTCTCTGACCCACAGATGGGAGGCCAGGTTGACCTAAACCCAAAGA[A/G]AGAAAAAGCTTTCGTGGGTGTCTGGAGGCTTAGAAGAGCTCGGCCTCATAGAGAAAGGCCTGGGAAGGGGCAGAGGTATGCTTGCCCTACGTGCCCCTAG
5	21687412	BES6_Contig159_782	0.39	0.44	TTTTCCCCAAGGAAAAGAACTCATCATAAGGATCCTGCCCAAATATATTCCGGAAGCACTGTAAGTGTTTGCCTCATGCTTCTGTATCAGTTTCAGGACC[A/C]TCGTAAATCAACAGTTGGCGGATGGAGGTGCTCAGACCAGAAATCATGGCTTTGAAAACACAGCATCATTCTCCTCGAGCAGCTGCAGCCTTTTATTAAT
5	24576130	SCAFFOLD185016_10368	0.07	0.11	AAACCTGTTAGCACGTTGCTTCAGCTACATCTTGCCTTCTGGGCTTGGACTTGTCTGACACTTGACCTCTGATGCCTGTGGAGCTCCAGCTGGCACCCAT[C/G]CATGCGTAGAGCTGGAAGATCAGCATCCTATAGGGTAAACCTTGAGTCAGTGCAGGATCGGACACAGTGGGTAAATGCCTGCTCTTCTGCCCCATCCTCC
5	27358607	SCAFFOLD111401_12750	0.24	0.21	CACCCCTGTATTTGAATGTTAGAAAATCAGTCATATCATTCTATCATTGAGCTCTTTATTTTTCCTGTAAAGTTTTTATTTCCCCTAATTTCTAAAGTCA[A/G]CCATGGGCACTATATAAAATCCCAACATTATGGAAATATCTTACATTAAGGAAACAAAAAGATGTTTGTTTGTGTTAGATAGTCCATTTATAAACCTGTG
5	27358666	SCAFFOLD111401_12691	0.16	0.13	AGATATTTCCATAATGTTGGGATTTTATATAGTGCCCATGGTTGACTTTAGAAATTAGGGGAAATAAAAACTTTACAGGAAAAATAAAGAGCTCAATGAT[A/G]GAATGATATGACTGATTTTCTAACATTCAAATACAGGGGTGAGTGCCAAAGTTCAGCAAAGAACCAGAACTTTGATGGGACCCATGAGAAGAAAGGAGGG

5	29754060	BES7_Contig351_844	0.12	0.14	TGCAGCTCATCATGGATGCTTATAACGTGAGGGGCTGGGGGGGAGGGGCACTGGGGAAAGTGGAAAGGGACCCCCAGGTAGGATGGCAGAACCAAGAGGA[A/C]AATCGCCTGTTGGTATCACACCCCACAGCTCCTGCCCACAGTCTGTGTGTGTGGTTGTCATTAACAGCTCACTCAGTCTTGCACTCATTCATAAATCTTA
5	30554866	BES11_Contig416_1082	0.38	0.44	ATCCCTCCCCACTCTGCTAATGTTTTCAGATTTTAGCATCCCTGTTCTGGGAAGGGAGTGCTGGACCACTCACCTTGCTGCGGTACCAGGACTCAGCCTC[C/G]GCCCGGCTGCGACCGGCAGTGTCGTCATACTGAGCCTGGATCTCAGCCACAATGCTGTCCATGTTCAGGTCCCGGCTGTTGTCCATCTTGACGATGACCG
5	30554944	BES11_Contig416_1160	0.28	0.41	ACACTGCCGGTCGCAGCCGGGCGGAGGCTGAGTCCTGGTACCGCAGCAAGGTGAGTGGTCCAGCACTCCCTTCCCAGAACAGGGATGCTAAAATCTGAAA[A/G]CATTAGCAGAGTGGGGAGGGATGCTGAAAAAGCATTGACCTTTGTCTGGGTGGGCAAGGGAAGGGCGGGCAGCTCTCGGGATGGGGTGCAGATGTGCAAG
5	31636085	BV105572-86-R	0.30	0.44	TCCAGCTACCCTGTTGTGCAGAGGGTGATAGCAGAGGCGGGAAGCCAAGTGCAGGGAGGTGAGACGCTAAGAGGCCCTTGTCCTC[A/G]AAGCCCCACTGCTTGGTTCCAGTCCCTGCAGGTTTGCTTGCTTTCAAGTATATCTGTATTTACTCGATTCTCAYGTGATKGCAGCAGAAAATTSATTAAT
5	32232866	SCAFFOLD15052_35561	0.13	0.11	TAAGCTCTTGGTGTGACCAAGGAAAAAAAGCTTTGGTGAGAAAAACTTTGTAACACATATGAATACAAATAACTAATTTTCCATTGTCATCACTTACTAT[A/T]GATATGAGACAAATGAAACTCTCCATGCCTCACTTTCTCATCTGCAAAATGAAAATATTCTGTCTCACAGGGTTTTCTTGATTATTTCTAAAGTATTTAC
5	32829967	SCAFFOLD15258_15271	0.29	0.24	CTCTACTTCTTCTCTAACTCCCCCTGATTTATAATCTCCCCAATTAACTAATTTATCCCCCACTTCTCCCCATACACTCTTACACCCCAGGTCCCCTCTA[A/G]TTTTTCCCCTGACTCTAAACTCGCTTCTCATCACTGAGCTGACCACAGTTTACCATTGGTTACTCCCTGAACCCCTAATCGCTCCCTCAGCCTCCAGTTC
5	32899679	BES10_Contig780_1565	0.15	0.09	GAGAAGGTGAGTTCTTTGCTTGCAGAAATAAGAGGGCAAGAAGGCATCTTGAGTTTCCATCAGAAGACTGTAGGGTTCTCAATTGAGTTAACTGTGGGGG[A/G]AAAAAGTCATTTTGGGCTATGAACTGACCCTGTGGCACTGAAAGGGATCTTGAGTTCCTAAATTCATGCTTCAGTTACCGAGCGGCACAAAGTGACAAGG
5	33154435	E522K15-33401-2	0.37	0.42	AAAATGTGTTAAAAAAAAATTAGGAGAATTTTTTTTTTTTTTTAATTTAAGACTTGGCTGTTATGTTTTAGAACAGGTTAAGGGAGATGCTTAGAATAAA[A/T]CAGATTTAACAGTATGTGAGAAGACATAGGTGTTCTTTGATCAGACTATCTGACTCCTGTCTCCCTCTGCCTGCCCCTGCTGCCCTTCCTTCCTCAAACC
5	33212777	E327D11-34412	0.27	0.20	ATTACTCATCAAAGGAGGAGAAGATGGCAACAGAGAAGTACCTGAGGATAAGTCACGGAGGGGAACTTATGGTGTGGGGGATGTTAAGGGCCAGGTGACA[A/G]CTTAAGGAGTCCCCTCACATCAGACACTGTGGGCGCCCTGCTTCCTCAGCACTCTTCTCAGAAGCCTGAGTGTCTGTGTGCACACACGTGTTTCCCTCAC
5	33224564	E327D11-34436-2	0.14	0.22	CATCATGTTTTCATTCTCTAGGTCCAGAAGCTTCTCTGTGAGCTGCAGGATGAGAAGGGCAGGTCTGGGGGCCAGTCCTAAAGCCTGGCCACTCAGCGGA[A/G]CCTAAGTCCGAGTGACCAACCTGGGCCCGACCCCTCCACCCAGGGCTCCAAAGGCAGCTTGGCCCAGTGGCTTCCCTACGGACTCCTGTGGGCCTGGGGT
5	33228122	E327D11-34444-2	0.42	0.43	CTTCAGCTCTGGGAAGACTTGACAGAGGCAGAGGCGGCAATGATAGCGTTGAGTTGCTGGAAGGCAAGAGGAAGAACTAAGTAACCCCACGCCAGGGCAG[A/G]ATGATGTCAGAGCAGGGCCAGCAGGGAAGGGGGCCAGCAGGTCAGGAGTTGGGCGGGGGCACTGCAGACCACYAGGATTCGGGGGAGAGCCGTACCGGCG

5	33234562	E522K15-33417	0.44	0.43	ACCTGGGGGAAATAAGAACCACTTCTCTTGCTTTCCCCATATATCACCTCCCCTGGAGGAAGACTGGGAGATTCCTTTGGTCTTGGATTTTCCTGTCCCC[A/G]GTTCCCAGACTCGAGGGCTTCTGGAGCCCAGCGCCCCTCTGTGCTGACTTGTATCTGCTCATCTACCCAGGGCTCTGAGTCAGGAGATGAGGAGCTGCCC
5	33834359	BES4_Contig503_1207	0.46	0.46	TACTGGGTTGGATGATGCTGGTTCCTTGTTGGCCTACCCCCAGAGTACCACCCTGAGGGACTCCTAAATCCCTTCTTGCTCATCCCTCTTCTCACCTGGC[A/G]GGGCCTCCGGTCAACGTAGCATCAATGACATGTTCATTGTTTATTCGAAACATGTAGATGCCTCGATTCTAGAAAGGCAGAGGTTGCAGAAGGGGCAAGA
5	37022996	SCAFFOLD191219_4666	0.13	0.15	GCTTCTTGTAGGCAGGACAAGGCTTCCTCCTCTTTGCTTATACCTGGAACAGAGCACTACTCAGCAAACATCAGAGGACGTAGAGCTAGTGGATCGCTGC[A/G]GACTAATCTGGGCTCAACCTTCTTGCTGACAAAGGAGTGAAAAGGAGAAGGGAACTCATAGCCTTGATTCCACAAGACAGAAATAGTTTCAGCAAGTCAG
5	37666660	E228D18-38734	0.47	0.04	TTCATCCTCTTACTTTCCTAAATGTAAAACAGATACAACATGTACTAAACAGTATTGTATGTTGTAACAGACATGGTGAACAGAACTAAGCATATTAAAT[A/G]TTATGTATATATAACAAGTGGTAGGTGCTAATATTATGTTAACATTCTCAATGATACCAAATAATGTTACAGAAACAAAACTACATGCTCTCTCTAAAGT
5	37728419	E228D18-38743	0.38	0.41	AAGGAAGGTCCATGTAAAACAGTTGTAAGAAAACCAGCAATATACAGTTTTGTACAACAATTTTTTTTAAGTCCAAGATACTCTACCTCTTTTTCAAAAA[A/G]TCAAGTGTTATAATAGGAACATCAAGTTAACCTTGACTATCACCGTACTATTGGCCTAAGTAAGCTAAAACAAAATTGTTCAACTAAAATATCTGAAATA
5	43286973	SCAFFOLD307284_3932	0.14	0.16	TGTAATTATTCTATGCTTTGATTTATGTCATCATTGTACAATGCAAATGCAAGTTAAATGTTGAAGTGTTATCTTCACTCTCTTATTACTGTATGTTCTC[A/G]CTGTTAATATAATAAGACCTCCTGCCCTGCTCACATTTTGTGCTCATCCTCATCTCCCTGGCTTCACTATGATAGTTCCTTCTTGATTAAGTTGAACAAA
5	43287445	SCAFFOLD307284_3460	0.10	0.15	GTAAATTTCTCTCACCTGTACCTGTATCATTCCATCGGGAATTTTGGCAAGTATATAACACACATATTGAGACTCACGTGCTTTCTTGGTGAAGGTCTCA[A/G]TCATATCACTGGGAGGTCCACACCCTGCACTATTGCACGCCCTGACTTCCACAAAATACTGGGTGTCAGGCAGAAGGTTCTCCAGCCTGGCCGAGTATTC
5	43713251	SCAFFOLD121897_4511	0.48	0.35	CCTAGGATCAGACTCTGAGATAGATATTAACAAACATATTAGGGAAGCTCTTGGAATCCAAAATTGTGGAAGAGAAGGAAGGAATCAAGGCTGGATAGAG[A/G]GAGAAATCAAGACATTATGTGTCTTCAGTGAAAGCCTTGGTCAACCTTTCAGGGCATTCTTAAGATCCAAATTGTGATAGGAGGGCCAAAGCTTTCTACC
5	43717398	SCAFFOLD121897_356	0.48	0.35	AGAGTCTATGATTCAACAAAATGTTGCTATACATGTAAGTAAAATTAAATTCCACATATGAGAGAAATGTAAATTGTTAACAAAGATTTAGTGTTTCTTT[A/G]AAACAAAATGACAGATCATTAACACAAAGTCTCCTGTGTTTTTAACACAACCAGTAATCTAACTCATATGCATACTGATGATTAATATAATGCATTATAT
5	44751134	SCAFFOLD150985_4812	0.44	0.41	CAATATAAAATTTTTAAGAAGATATTATACTTCTTTTTTTTTGACTAAGTCTTTGAAATTTGGTTTGCATTTTACATTTATAGCACATCTCAGTTAAGAC[A/G]AGACACATTTCAAGTGCCAAAGCCACAATGGCTTGTGGCTACCCTATTAGGTAGCACAACTCTAAATACTAAGACTTTGCCAAGTATATATATTGTACAT
5	46117940	SCAFFOLD125222_757	0.19	0.30	TATATTATGTGACTTTTAATGAAATAAATTTTTTTAAAAAAATGGGTTGCTAGGTCTTATCCGCCTTTTAATGGATAAAACTACAGAGGGCTTGAAATCA[A/C]TGGAGACTGTTTTGGAGCTTGGGTGCCACAATCTTCAAAGCAAAACTGGAGTATCTTTTTATTACGCTATTGCTTATTATGTACTTCTACCTGTAAACTT

5	47589344	E154A23-34328-2	0.03	0.03	AGCAYCAGAGTCTTTTCCAATGAGTCAACTCTTCRCATGAGGTGGCCAAAGTACTGGAGTTTCAGCTTTAGCATCATTCCTTCCAAAGTATTATCACTCA[A/G]GATTAATTATAAACTCATGGCACTTACATTCTGTTTCATGCTTATAGGCTCCTAAGGTAAGCTGACGAGATGTTGTCAATAATTGTTGCATCATTGCTGT
5	47851648	E254B18-36398	0.29	0.28	GCACTTTCTGAAAAACTCGGTCTCTAATCTCCTCCCTCCCCCCAGCCAGCTCACAGGGGCAGTGATTTCCTCAAAGAATTTCAACAAATTAGTTCTCCAC[A/G]ACTTCCCATCCTGAAATGATGTTAGCTGTCTTTAATCAAGCTTGCTATTCCAACTGTTCTTGATTTGATACCTTCAAGTCGTTACAGAGTCCTTCTCTTG
5	47858719	E254B18-36380	0.32	0.25	TTAGTGGAGATTCCAGGGAAGTTTGCTTCAGTTTCTTCTGCATTATTTGATTTCTTGTTGCAAAAAGAAAAAATGTTAGTGCTTTGTGAACCAGGATTTC[A/G]GAGCCCTAGACTTCCTTAACAAGTACAGCACACGTTCCCAAGGGTATATGGTACACACTCCCCTTTTAACAACTTCACGTTTCACTTTAATGCTCAGTGT
5	49190216	E261H16-33705-2	0.17	0.33	TTTTTTTTTTATAGTCACTTTGAGAGACGTTCTATATTTGACAGACTTTTAAGCACTTTTAGGTATAAGATGACTCTTCATTTTCAGGGGATG[A/G]ATTTGGAAAAAAGAAGTATTTTATTCCCCAATTTTCTGAGTTTGAATTTTTACATTTAAATTTTAAACAAATTTAGTTGTGTTCTTAATTTACAAAGAG
5	49230778	E261H16-33697	0.13	0.15	GAAAGAAGGAAGGAGGGAAGGAACAGAGGGAGGGAGGCAGAAAAGAAGAATTTGGCTAATGTTTGACTCTTGTACTCTAAAATCTAATTGGGATTGAAAA[A/G]TTAAAATGGGGCTTACCCAAAGAAGGTATTAATGCCCAGTTTTAAGGCAACAGTTTTCTCCTGAATAAAACTTCTTGATAATAGAATAGAATGAACTGTC
5	49276740	E261H16-33727-2	0.37	0.44	AAATTTGTATGTTTTACCCTACTGACTAGCTTACCCTTATAAAAATTCTCTGCACTGTAGGCTGGAATTTGGATTACTGTGTACTTTCATGGACCACAAG[A/G]AAAAAGATGTCCTGGTATTTTAAAACTGAAAATCTGCTACCAACAGCCCAGAGTTTAAGTTTCATGACCCTGATGCCCAAGGATAGAAAGCTCATTACCT
5	49283596	E261H16-33737	0.35	0.34	AGTAAGACTAATCCTGGTTTATTCTTTCATTGACTATAAAGTCCTAAACTTGTCTTTAACAATACCAATAATCTCATCATGTGGTTTTTGGAAGAATTAA[A/G]TAAGATACTATGTCAAAGTGCCTAATACGTAGTAGTTCTGTAAAATTGTTCATATCTTTCTTTACTGTTTTCCATACTTAGAACACTAATTTTCTTTCAA
5	49295542	E261H16-33709	0.36	0.37	CCTTGTTGGGTTTTTGGGGCCATGTTTTATCATGGTCCTCCCCTTGCTTTTCTCTTTTATACTTTATTAGTGTTTTTAAAAATTATAAAAATAGGGGCTG[A/G]AAGAAATTGCTGCTGCAGGAGACCTGGATTTAGAAACACCAGCTTTAAGTACATATTGGATATTTTATCCCATAAAACAAAAACCTTGGTCTGAGTTCAT
5	49352070	E261H16-33719-3	0.42	0.41	ATATCGCCTTTAAAAGATAGCTCCAGCTATAATTATAACAGCTAAATTTGGATATATGATAAAATATTTAAAATGCCCAAGCTATTTTAATTAACCATAA[A/G]TAAATTTTTAAGAAATTCCTAAAACAAGACAAAAAATTTTTTAAAATATAAACTAGATTATAACTCCTACAAAAAAAAATCTCAGCAAGTGACTGAGTCCA
5	50700153	SCAFFOLD270045_9984	0.39	0.43	ATGTGATAAAGGAAATCATGTCTTTACCTTGCGTTTATCAATTAATGTCATACTTTAATTATCATGAAAATATACAATTATAAACCCTGATTTATGCTCA[A/G]TAGGCATCATGGCATAGTTTTACCATAAGCAATTAAGAGTAGATCCTATTTGATGAGATTTTTTTACTCTCCTGTTACTCAACAGTTCTTTTCTGGAATA
5	52172681	E254B18-36368-2	0.25	0.44	TGTACTGTTTTCTGGACAGTAACTCTTCTCTAGGAAAACTGCCACCTCCAGGGTTTAATATCCAGACCAACTCTCAGGATTATAGATAGGAAGTTAGAAA[A/G]CACACAGGTCAATGCTCCAAAATTTTAGCCACATTTTCCCTAAGTGCTTCAAAGGTTTGCAACTGTCCRGGCAATCCTGAGCACCACCCTAGAACTCTTT

5	53242603	E254B18-36388-2	0.50	0.34	CACTAAAAATTTTTGAAGACTGATCACCGAAATGAAGATTCATGTTTATGCCAATAGAGATATAATATAATTTATGTGCCTGTCTGTTTCCTCTACAAAC[A/G]TTTGTGGTGCCTGTTTTTGCACTCATCACACACTGAGTGATTCCAATGTGTTACCTGACGTCATGGAGCTAATGGTGAGGGTAATGGTGGCCATGAGGCG
5	53243102	E254B18-36388-1	0.26	0.27	TCTGGAGAGGGCGAGAACAGGGGAAATCCTGTGGTCAGGAATCAGCCATGCCCTGTTCCAGCTGACAGTTTGCAGAAGATGAGGGACATGAATCTTAAAA[A/G]GGGCCCCAGGAGAGAAAACACAGGTCTAGGACTAAATCTCCTTTAGCTTCTCAGAGCTGACAGGAAGGATGCCTTATTTTCCCAGCAATAATAGATTTCC
5	55226469	BES4_Contig227_995	0.37	0.26	AAGCTTATTAAAATGAACATGAGATAGTGAAGAGTTTTTTTTTATGATATGATGTATAAAAATGTAATTCCCAATTTTTCTTTCCATTTTGGAGGCATG[A/G]TTGTGAACTTAAAGTTTATAATTCTGTAAATACATACTTATTTTCAATATACCTTTTAGGCAGTATATATGATTTGTTAAAAGCACCAAGAACTCCTAAA
5	55272386	AF440372-229	0.36	0.50	GAAAAGTAGTTTATACATATGATGGAGTATTATTAGCCTTAAAAAAAATCTGACACGTGTGACAACATGGATGAATCTTGAGGACATTATATTAAGTGAA[A/G]TAAGCCAATCGTAAAAGAAAAATGCTATATGGTTTCACTTATATGAGGTATCCAGAACAGTCAAACTCATAGAAACAGAAAGTATAATGGCAGTTGCCAG
5	55429339	SCAFFOLD65181_13625	0.40	0.48	CTTGTTATTTTTCATCAATACATGTTTCAAATAGATTTAAGTAGTGAAAATGAGTATGTAAATAAGTATTCAAAGAAATAACGAGGGCTAGATTATTTAC[A/C]TTTTAGTAAGCTAAAAAAACATGGATGTAGCAAAACTGTGCTGAAATAAAGGTATTAGAAAAATCTTGAGACTTTCTGGACTCAAATTAGTAACAACACA
5	55881323	SCAFFOLD310378_3418	0.38	0.41	ATTTCTCTGTTGCTGGAAAAAAAAGAAAACTATTGGTTAGGTTTTGGAAAGTTTACATAGTTGGGAAGATTGTGGGTGGAGGAGGGCTTTGGAACTACAA[A/G]GTTGCAGCTAGCAGCTACTAGCAAATTCTGTTTTGAAAATGGCTGATAGTGTCCTCAAGTTTGATTCAGTTTAAAAAAGTCAAGTTCTCAGTGATACTGG
5	59034363	SCAFFOLD65330_13078	0.39	0.26	TTTTGAAGCCTATCATACAGGTATGTAGTTTTCATTTCTTGAGTTCTGTTTCCTGGAGAGACCTCTAGAAGCATTAACTCTGTCTCCCGATATTGTCCCC[A/G]TTTTTAAAAGGTTGTGGTCCTGACCTGAGATCAGCTTTAATGGACCACTATGAGCCCAGTTACATAAAGAAGAAGGAATGCTACCCATGTCCTCATCCTT
5	61286503	BES2_Contig371_797	0.37	0.34	TTGTTTTTCTAAAAACAGATGCAAGTCCAGCATGAGGATCAGTAGGAACTTTTGAAGATTTATTTAAGGTGGTGAAAAAATGATCTTCAACCATCTTTAA[A/C]TTAGTAAGCGAAAAAATGATAATTTAATTAAGAATTGCATTGTTGTATTTGTAATATAGATTTCAATGGAATATACCTGATATTTGTTGATGGTGTTCTT
5	61286610	BES2_Contig371_690	0.31	0.46	TCTTCAGTAAATGATTGATTCCTTTGCTAACATTGATATCCAGTCGTGGAAAACAATACTGGAGCATGATCTCCCACTCAAGCCAGAGTCCACATTTGTC[A/G]TTTTTGTTGTTTTTCTAAAAACAGATGCAAGTCCAGCATGAGGATCAGTAGGAACTTTTGAAGATTTATTTAAGGTGGTGAAAAAATGATCTTCAACCAT
5	61644104	BES11_Contig132_176	0.18	0.19	ATGGCAGCCATGTGCAGAGGACTCTTCCCTTCTTTGCTCTGTGGAAACACAGCATTTTTTCCCCTAGTGCGAAGCATGGAGCATGCCCTAGTCCCCTTTC[A/G]AAAGGACACATCTTTGAAGATAGGCCGACCATAGGGACATCTTCTCAACCCACTTTCTCCTGGGAAGGTCTGGTCAATTCAGGACACAAGCAGATGACTC
5	61798501	BES11_Contig325_836	0.23	0.21	TCCCACCCTCCCTAGACACAATAGGGGCTGTGTGCTGCCCCAAAGAGATATTTATTCCAGGACCTAGAGAGGCAGGATGGGGGTAGAGAAGTGCCCAGTT[A/G]GAGTGGGGGCGGGGAAGAGGAGGGTAACCAAGTTGTGGTCCATTTCTAACTTCTTCTTAGGGAGAGGAAAACAACCCCACATCCTCCTTGATTAACTCCT

5	64474109	SCAFFOLD10056_29579	0.26	0.21	ACTGATGTGTTGTGAGTTTTATAGTTGGCTTTCCTGAAAGTTTAAAAAATGGAACTTGCACATGATGCTTTGAATATAATCTCAAATGGAGGCTGGAATA[A/G]AGAGTTGCTAAAGCGAGTTAATGGGGAAACTGACTGGAAAGCAGCCATTGGAGCTGTTCTTAAATAAGTCTTCTTTATTCAGTGATGGTGTGATCACTGA
5	64565964	SCAFFOLD5029_246	0.26	0.29	TTGTGAAAGTTTGGAGACTGCTGTTGACAGGAAAAAAAGAGAAAAAGGTGTGTATGAAAACCTGCTGAAACAGAAGCACTGCCACCACGTGTGGAAAAAA[A/G]TGACCACATACAGAAAGTTGTACTCTTTAACCTTGAACACTTAGAGAAACTATGTGAAGATGCAACCTGAAAGGAGAATATGTTTGCATGAAGTCTCAGC
5	64705739	E314A10-38917-3	0.46	0.49	GTTCCTGCARAATTCAAAATGGCCCCTCCCATGTTCTACCTTAAACTTTCTGTACTTAYAAATGCTATTTACTCACAGAAATATTGCCTACTAAACTTGA[A/G]GAATAAGGGCCTTAAATCCAAAGTTAATACAAAATTATTATGAAAATAGTCCTAACTCTGATGAAGTCTAAGCACTTTTTCATGAATGCAGTTAGTTTCT
5	64705851	E314A10-38917-2	0.46	0.48	TTAAATCCAAAGTTAATACAAAATTATTATGAAAATAGTCCTAACTCTGATGAAGTCTAAGCACTTTTTCATGAATGCAGTTAGTTTCTCTAACATAACT[A/G]TAGAAGATTCCTTCATCATAAAGGAATAATATAGCATTTTCCCACTTTCTTCCTTCCTTCAGGAATGTTTGAATGAAGGCAAGTTTTATTACAAACAGAA
5	64728115	E314A10-38927-2	0.23	0.24	ATGTTCCAAGGTCAAATCATTCAAAAGATGCTACGCTATCTCTATGTAGGGAAGTAACAAAGGAAACTGACAAAATAAAGATTGAAAAGTTCTGGAACAA[A/G]GAAATTTGTTTTCCAAACTAATTGRATTCTCTTTTTCCCATCTAAGTCCTGTKAATATTTGGGGAACTAATATTATACAAATGGCTTTTGTATTAACAAT
5	64808701	E314A10-38929-2	0.43	0.50	CTGTTACTGACCAGAGTTCTTGGCCTCCTTAGCCGATGGAAATTGACCAGAGGCCAGACAAGAAATTCAGGCAGGGTTTTACTAGGGCTCCTGTTGCAGC[A/T]GGGGCTGCGRGGAGSGGGAGGGCGAGAACAATCAGCAGGGTCCCTTGTTCACTGGCTCCCTGAGGGGTGGGGGGAATTTGTTCCTTATATGGAGTGAGGG
5	65557646	SCAFFOLD130216_24898	0.44	0.48	CAGAAAGACATACTAATCATAAATGTGTATATACCAAATAACAGAGATTCAGAATATACTTTGGATACCCAAAGCTTGAAAAACATTCTGCTTGGTGGAC[A/G]CATTGATATACTAAGCAGATCTCAGGCCCTGAGTCCATAGGAAAGGTCACAGAAATGCCTTATCTTAGACCCATCCAGACCTGGCCCGTCTTGCATTTGG
5	66501886	E258B16-36408-2	0.10	0.05	GTCTTTATCTGAACTGTTTGGAAAAAAGAAACTAACGATGCTGTTTCTCCATGCAATAYTGGTAGAGGAATGTCCTAAGAGAATATTACTATGTATACAT[A/G]TAGGAACCAAGGATGAGAATATTATGAAATCCATGACATTATCCAGATTTTGACAGGTGGAGTGGAATTTGTTTCTCTTCAACAAACTTGTATTAAGATA
5	66501993	E258B16-36408-3	0.22	0.18	ACCTAAGTTCCTGATCCTAGCTTTTGCAAATAAGACCCACAGAAGCCCTAGTAATARCTGTGGAAAAGATAATGTCTAACTAGTGCGAATGGTGCAACCA[A/G]TCAGAATATCTTAATACAAGTTTGTTGAAGAGAAACAAATTCCACTCCACCTGTCAAAATCTGGATAATGTCATGGATTTCATAATATTCTCATCCTTGG
5	66531514	E258B16-36414	0.35	0.31	TTTTATCAAAGTCTGAGCTGGTTTGAATAGTCATTCCCAAAGTGTCCAAATGACAATTTTGTGGTAGCTCAGAGAAGCACAAAAGACCAGTTATCTAATT[A/G]TTTTACTTATATTCAAAGAAATGCAACAAATGCTACTCATTTCTTGGTTGTTTTGTAATTGTATGAAATTCAGAAATAAAAATGTGAAGGGGCCAGATTG
5	66550371	E258B16-36426-4	0.10	0.05	TAAGTACAGTTAAACATATTAATTACAGACACTCTAGAGACTTAGAAAAATGGCTACAATAATCGCATGTCGTTAATTCTAAGGAAACACAGCTTCCTAG[A/G]CAACACCTGCTAGGTACATGTAACTGCCAGGTTAATATGGACTTTTACAAATTAATAGTTTCCTTGCTCTTAAGCGACAGTGAACTCCTGAAATCTCTAC

5	66582373	E258B16-36422-2	0.44	0.13	CTGACCTATGCAAAACATAGTGAGTGATGCAGGCAGATGTGACATTTAAAGGGACGATGGAGAGGCCCAAAGGCAACAGAGATGGAATTCAGCTTCATTT[A/C]GAAAAGGAAATCGGTTTCAGTTGCAGAGCCCTCATCTCTTCATCAGAGGACCTTCTAGGAGGGCACCCACATCTCACTCCTCTTCTTCCCAAGACAATCA
5	66652398	E258B16-36410-2	0.33	0.22	CTGCACATACAATGCTGTGTGTAAATAATYGATATACTTTTGGACAACTTCCCACTTTATACATTCCTCTTTAAAAAGTAAAAATAAAGCCAAAACAAAA[A/G]AGCAACCCATGGAGATCAACTAAATGTGAGAAATGCACTTTTCCTAAAACAAACAAACAATTCACCTCCCAGCTTGGGCTGGGTGGTATGCGTGCTGGAA
5	66652469	E258B16-36410-1	0.37	0.34	TCACATTTAGTTGATCTCCATGGGTTGCTYTTTTGTTTTGGCTTTATTTTTACTTTTTAAAGAGGAATGTATAAAGTGGGAAGTTGTCCAAAAGTATATC[A/G]ATTATTTACACACAGCATTGTATGTGCAGGCACTGTATTTGTGCAGACACCGTCCCCAGCTGCTGCTAAAAGGCAAGAAGGCATTTTGCTAAAGGTTTTA
5	66668222	E258B16-36432-4	0.44	0.14	TCTCTTATGGGTAACAACAGCCCAAGTATATTAATTGTAACTTGAGAAATCCACGTATCTAACACATAGGCACATTTTCCTCCAGGCAGGTTGAAACAGY[A/G]TGAGCATAGACATAAAATAATCATCTGACTCACTGCCAACCTTTGCCCTCATTTTCATTCCTGGTTTTATGCAGGAGTAACTAAGCAGGTTAATGTCACA
5	66843802	SCAFFOLD140266_19958	0.32	0.34	CATATTCAGTTCGACCAAGTTTCTGTCCTGATTGAGTGAAATGAATCTAGTATTAAATGTATTTTATGCAGCTATGATTATACATGGCCCCTTGGCTTTT[A/G]CATACATTATGTTTAATCCTCACAAACACCCCTGTTGAAACAGTATCATTCCAATCACACCAGGAAACTGAGACTTTAGGAGCATAAGAAATCTATACAT
5	67053078	E193A21-38797	0.23	0.14	CGCCCCTTGAAAAATTGGAGAAATTTATGGAAAAAAAATCAGGCTTAGGAGACTACAAAGTAGGGAAAGGAGTAAGATTTGAGGGCACGGGTATTACTGA[A/G]AAGGAGACTAAGCACCACACGGAGAGACTGTTTGAAAGTATCCATGGTCTGGAGTAGGGGACATCCTTCCCATATTTATCTTCCAAATAAACATATTTTT
5	67061837	E193A21-38799	0.48	0.47	AGGATTCTGCTAAGAATGTTCCCCCTAAAGGCTTTAATTTGGAAGAAACAGGGAGGAGAGAGAGAGAAGTCTGTGTTCAACCATGGTTAAAGAGGCCACCTT[A/G]GGAGCTGGGGAATGTTCCCTTTGGTTTATTGTCTTTTCAGCAAGGCTGGTGGTGTTTTTGTTCCCAACTGATTACTCTATCAAACCCCTAATGTTTTCAA
5	67072382	E193A21-38803-1	0.28	0.41	AGTCCTGAGTCCTGCAGTGGTTCAACTGGACCAGAGGGAAAAGGGAGATGGAGGAGTGTGTCAACACAGACAAGAGCTTCTCCAGTCAGGGGGTGCTGGA[A/G]CAGAGCCCCAGGGGGTGGGGAGGCAGGTCAGAGGAACAGATGGCAGCCATTAGAACAGGCTGCTTTTAAAGAGCAAAAGAAACAAGAAAGGCTATCAATC
5	67072485	E193A21-38803-2	0.28	0.42	ATGCAAGTCTATATCCCTTAAAATAGGGAGGAGATGGTTTTCTTTTGTTAAGATATTATTTTCCCCTAATTATTTCCTAGCTTATCCTTCCTTTTTCTTA[A/G]AAGATTGATAGCCTTTCTTGTTTCTTTTGCTCTTTAAAAGCAGCCTGTTCTAATGGCTGCCATCTGTTCCTCTGACCTGCCTCCCCACCCCCTGGGGCTC
5	67075483	E193A21-38821-4	0.29	0.42	TTTTTTCCCTCCATCTAACCTTTATCAGCAAACTAACAGAGGAGTATGAACCCCCAGCAATCTTGGCCTGGAATTTTAGCTATGGGTTTGCCACTTTCCT[A/G]GAAGTTTCCTTAACACCCTACCCAAAGCATTTTCTTAATGGGGCAGTCCCCCAGCCTTTTTAATTTTGGCTCATTATTTTTCATTCTACTGAGCTAAGTG
5	67075796	E193A21-38821-2	0.29	0.42	CACATTTTTATACCTAAAGGCTGATTGGTTTATTTTCTGCTGTATATATCAATGATCATAAAGCAAGAAGACCCCTGGGACTCTATTCTGTTTAGGCAAA[A/G]GCAGGTGTGCTGGGGAGCTTTATATTTGCACAYAAACACAGAAAGCAGGGCTTATTTTTGCAATTACTTGTGAAGGCTGCCCATGTTTTCCAGCATGTAA

5	67075920	E193A21-38821-1	0.25	0.21	CAGGGGTCTTCTTGCTTTATGATCATTGATATATACAGCAGAAAATAAACCAATCAGCCTTTAGGTATAAAAATGTGTTTTCGCTCATATGCAGAAGCTC[A/G]TCGTTAATCAGCATTGACTTTACCCGTGGAGAGCTGGGTTGTATAAACTAAGTGTGACAGTATCTCCTTGTAACCCCATGAGGTGAATTAAGGTATTCAC
5	67335731	SCAFFOLD170731_20633	0.40	0.44	TTTAACTTTTAAACAAGCCCCAGTTCTGAGCCTTAACCTGGATGATGACCTACAGATTCTCAACCTGAGTTAGAGGTGGATGAAAAGGAAGCAGTTTTCT[A/G]GGGCGTGTTTTCATGTCAATAGATTCATTTACTCAAATGTGTGATAAACACCAACTGCTCACACTTTATGAGAGATACATAAAAACGTGGGGACAGAGGA
5	67572773	APAF-474	0.48	0.34	GTGAGTTGCCTGTTTCTCAGTGATTTTCATGAACCTGAAGAAACATTTGACTTCTTCAGAACTTTCTCTTTTTTATCTTGCTGCATATTTGTACTTCTTA[A/T]CAAATGGAGATATTATTCAGGGATTTAAATACATGATTTTTTTTTGTGTTACTCTCTTTATCTGCAATCCTGGCTCATTGTACATTGTGGCACATTAAAT
5	67700904	SCAFFOLD122517_7471	0.46	0.40	CTGCTCGGTTACATCAGTTGTGAGATCCCAGGTCCTGTCCACATTCCAGCACCACCAACCTATCAGGAAGTTCTGTTCAAGATACCTCCCCAGCCAGTCC[A/C]TCAGTATGAATTCATTTATAAGCATATTTTTCAACTGCCAATTAGGATGATTAAAGGCCAGTAACATGAGCTATTAACTGATTATAGAACATTTTTATTA
5	67701052	SCAFFOLD122517_7619	0.25	0.26	GGTGCTGGAATGTGGACAGGACCTGGGATCTCACAACTGATGTAACCGAGCAGGACTCACTGCTGATGTAACCGAGTGAGGCCTTTTCAGGACAGACCCC[A/G]CTCCCACATGCTCTCAGAAGTACCCAGATAATAGTAGCTCATGCCTGAGTTTTTCAGATGCTAAAAAACCACCACCATGCATGCATGCTTAGTTGCTTCA
5	70277640	SCAFFOLD250217_1856	0.50	0.01	CCCCTTTTCTACTTCATGTCCTAGGTCACTGATATATGCTGGCCAACTTAGAAGACATTCTATGCAGAGGCTGCATTTTAGCTTAGGACCTATATGTCAG[A/G]TGCTGTGGACACAGAAAGCTGAAGACTCATAACCTATAAAGCAGATCCTAGGGGGAAAATCTGAGGAAGTGATAAAAAGTTTTAAGGAAATCCTTGGAGT
5	70277717	SCAFFOLD250217_1933	0.00	0.01	CACTGGACCACCAGGGAAGTTTCCCCCAATAACTTTTTAAAAGGCATATTGGTGCATCTAAAATAACACAGATATTTCCCCTTTTCTACTTCATGTCCTA[A/G]GTCACTGATATATGCTGGCCAACTTAGAAGACATTCTATGCAGAGGCTGCATTTTAGCTTAGGACCTATATGTCAGATGCTGTGGACACAGAAAGCTGAA
5	72588293	SCAFFOLD210395_13865	0.27	0.28	TTGACCAAAATTCGGTGGAAATGTGTTTTCTTGTGATTCGTTGGGTCCTGGAAGCTATATGGACTGCTCTCTCTGACGGGAGTGATGACACCTATTCACA[A/G]ATCAGTTACAGAGTCACTCTGCTGTACAGCAGAAATTAACAATTATTTATGAAGTAAATCAACTATACTTCAATTTTTTTAACAAAATATAAATCAGTTA
5	73062135	SCAFFOLD255941_5771	0.22	0.28	AAGAAGATCATGAATATTTTAAAGACTGTTCAGTAGCTCACAGGGATCAGGGAGGTCAGAGAAGCGGAGAGCTGTCTCTTTAACTAGAACCAAGTCCTTA[A/G]GGGCCGCTCCAGTAAAGTCATCGCTATCACTGTCTGTAGTGGGTTGTAGCAGGGCTGGTGGCTGTCATACTTTGTGTGTTATACGTAACCGCACTTGGTA
5	74052334	SCAFFOLD100565_4294	0.44	0.44	GCCACTCCATCCTCAACTGGCCCTCACCCAGTGCTTCCCGTCTAGAAGAGAACTGTCCTCGTAGTCAGACATTCAGTACCCACCAGTGTGGTCATCTTAG[A/G]GGCATTTGCACTGTTTAAGGCAGAGCCTTCTGCAGGCTGGGGGAGGGGACTGTCTGTGCTGAGACCCCAGGGTACTTTTCAGGGAGAAGGAAATGCTCTG
5	75593741	SCAFFOLD312109_31344	0.31	0.46	TTGTCCCCTGATCCTGTTGAAAGGAGCCAAGCTCCAGGTGGAACTGCAGCTCGGGCTTGCTGATATAGCACGAGTGGAGTTTTCCATAGGAAATTAACCA[A/G]GTAACACTCACATTCTTTCCCTTCTCTCCTTTCCACTCTTCTCTTCCCTCTTTAATGAAACAGCTTAAGCTCAGCACTTGAACTCTCTCACAGGGGTGAA

5	76274368	E213B06-36295-2	0.32	0.50	TCATCTATTTTGTTATTTAAATTGAGGTCTATTAGGGACATAATTTAGGTGAGAAAGGGAACTTAAGTACTGACCGCTCCCACCCTTCCCTGCCGATACC[A/G]TAACTTTTTATATTCTATGGCATGATATTTTCAACAAATATTAAGATTTTCCATTTTCAGCAAAGGCTGATGTGAAATTCCTATCAGCTTGAGAGTGCTA
5	76274550	E213B06-36295-1	0.28	0.39	CTAGTGCACTGAAGGGAACTTTTACCAGTTCTACCTGCATGAGTAAGGTAGCAGCTGTGAACACAATGCCAAAGGAACTCTAGTCCTTAACCACACTGCA[A/G]TTCCGCAATTGTGCCAATCTGGAGTAACAACACATCCAGACAAGGGATAAATGAGTGACATGGACTCTCAGGACTGCAATCTAGCACTCTCAAGCTGATA
5	76327020	E213B06-36281	0.15	0.27	GGAGTGCTGGATAGGAAATTGGGCTGCACACCTCCAACTGGATACGGAGGAGCCCATGAACG[A/G]GACAGCATGGAGTAAGCTCTGACACTGAGGGGAGGCGGCAAGAAGAGCAGGGTCACGGCAGACATATCATGGTGGGGCAGGGCTCTCCAGCTATGAGGAG
5	76353955	E213B06-36277	0.09	0.13	CATCGGAAAAATACTGCAAGGGGGAACGGCGGGAGAAAAACCNCCCTAGGGATGGTTCTAGGCACTCAGATTTGTTAGCATTCTGCCCTTTCCTGATGCG[A/G]TAGATAAGAAATCGGGGGCAAGCCATCTGTCAAGGTCAGGCAGTTAGGAAACGATGGAGCTCCTCCACTGCCACCCAGGTCTTCTCCCCAAGCTTCCCCT
5	76609610	E189E10-33745	0.23	0.26	GGAACAGCGAGCAGAGGTTACAAGTCCTCCTACACTGGGGAGTTTCCACCTCCTTCTCTGGCCAGCTATAGGGTCTCTGGGGAGCAAAGTGACTAAGATG[C/G]GGAGAATTTCTGGAGTCCTCCCTGTCCCTGGCCTTAGGCCTCACCACTGGGAAAAGCAGAGAAAAGTGGTGTCTCAGCCTGGAGATTCTGCACAAGGACC
5	76653711	E189E10-33481	0.23	0.20	AAAGCTCCTCTTTCCCCCTTAAAAATTTAGCTGTAAGGAGAGGAATCTAGTATCCACAGTTTTCTGTCTTGTTTCCTTCCCAACTTTTAAGACCCTTCCC[A/G]TCCTAACTTTGACTGTAGCCCCATCCAGAGAGAGCTTTCCTTCTCTCAGTGTTTACTTAGAATTTTACCCCTTGTTCCATGAACTGTAGTTGTACTATTT
5	76686530	E189E10-33483	0.15	0.15	AGTCAGGCTGCCTGGACCGGCATGCTTTGTCCATCACATCCTATTCATTCTCTACAAGCCTCAGTTTCTACAAAATGGGGCTAATGATAGCACTTACTTT[A/T]CCGAGTCTTAATGAAACTCAAATGGAATAATCCAAATATTGAGCATAAATGTGCATAGCAAATGCTTGGTGGATGATGGCATTTATTATAATTAATGACT
5	78451865	SCAFFOLD121791_4528	0.16	0.10	CAGGTTGATGAGCCCAAAAGCTGTGCCTCCTGACTCCAAGTCTCATGTGTTTGCCTACAATGCACTGGAAGCTCTGTAGATGGTTTGAGATGCCTCTGGT[A/G]AAGATAAACCATCAGAAACAAATATCGATCACATTATAAACCTAGGTGTGTCCCACGGTGTTTCATCATGTTTCCCAAAATAAATCAATTTATTTCATTT
5	78452321	SCAFFOLD121791_4072	0.22	0.08	CACATGAGAGCCAGGGAGCTGTCTTTATTGAGAGCTTGCACACCCAGGTGGGGGTGACATTAGAGAGCCAAGAGGCACAGGCACCAAGGCCAAGTGAGAC[A/G]TACTGTTCGTGCCCATAATCTGAATCTTAAAGACAGTAGGCACAAGTCACATGGCAAAGGGTTATGCTTTTTGAGTACTTAGCCTTACAGTGACCAGTAT
5	79053570	SCAFFOLD343248_236	0.40	0.47	AAGGGGTCCCCTTTGCAGATGAGAAAGCCAGGTCCCAGAGAGGGGTGAGTGAAACCAGAGATCTCACGTCCTTCGCACAGTGCCTGGCATATAGTGAAAC[A/G]TTTTACCTTCAGAGCACTGCAGTGTGGTCGTCCTCACTTACAGAGGAGACACCAGGGCTTAGAATCAAGGTCATTTGCCCACGATCTCACAGCCGTTGGT
5	79730962	SCAFFOLD246651_2804	0.36	0.24	ATGTGGCTGGCTGATCCTGGAGCAGATATGGAGCATAATGAGAAACCGAGTTCATCGCAGGAGCAACGTGTGGTGTTTAAGCCTCATTAGGACTATAATA[C/G]CAGTCGTCCAGACGCGAACCCCACCGTTGACTTTGACCTCACTTCCCTGCTTCCCGCTGCTCGTGGCATTATTGAACCTGGGTTTTTCCTGTAGGCCAGA

5	80043821	BES6_Contig369_1157	0.27	0.38	CTGGAGAGCTAGGACGATGCCGGAGCCGATGACCTCCACAGGTCCGCCCACTCAAGGATTCGCTGGCTTTCTCTGGTCTATTTTTGCCTGACACCTCTCC[A/C]AGGGGCCTGTGGGTCTGAGCTAATTTCCCTGGCTGGCCCCGATCCAGGCAGCTCAGGTGGATAGTGCCAAGCTAATGAGGCCAAGGTCATGGGCCAGTTA
5	80761583	SCAFFOLD76928_9451	0.30	0.39	CAGATCTTACTGCAGACTGTAGTAAAACACTATTTATAGGTTCAAACAAAAGAGCTAAAATATCTCAGTAAGATCAAAATGATTTATTGGATTTATTAGC[A/G]CAGTGATCATTTAAATACATTTTCCTACATTTTGCAGTGCTAAATTCATCTATAACAGAAGGATAATTTAATTCCAAAGGCAAATTAACCGCTCTTTGCC
5	80761718	SCAFFOLD76928_9316	0.31	0.39	CAGTGCTAAATTCATCTATAACAGAAGGATAATTTAATTCCAAAGGCAAATTAACCGCTCTTTGCCTTCTCCCAAAGTCACCCACTTTTGATATTTTTTT[C/G]CATAACTTGATTCAATTTTTTCTGCTCTTGCCACATTTTCTCCAGCTCCTACACACTTTTTTGGATGGTTATAACGTTATTATCTTATTATTAGTTGCTA
5	82134398	SCAFFOLD135541_8418	0.49	0.07	TAGAAGGAAGACCATGTAAGGACCCAGGGAGAAGGTAGCTATCAAGAAGCCAATGGGGAGAGGACTCGGAAGAAATCTACCCTCCTGACAACTTATCTAA[A/G]ACTTCTAGTTTTCAAAACTCTGAGGAGTAAGTTTCTGTTGTTTAAGTGACCCATTGTAGTACTTGGTTATGGAAGCCCTAGCAAACTAAAGAACAAATCT
5	84279556	BES7_Contig480_1179	0.36	0.40	AAAGAGCAATACCACCATTGTTTCTCTTTCAAATGGGACTGTTTTTTGTTTTTTGTTTTTTTTTTTAAAGAAAACAATACCTGTATCCTCTGTACTGCTG[A/G]GGCCAAAGTATGAGTCTAGGTGTTATTAGTGAGGCAAAAATGAACAAAGGAAGTGCTACAAAACCCTAACCACTCTTTTCTAAACAAAGTAGTTTAAAAT
5	85279271	SCAFFOLD144700_3610	0.47	0.04	TTCAGACACATTGTACACAATCTGTCTTCAGATTTTTTTAAAGATTTTTTTTCCAACTTCTTCTTGTGACACTTCTACTTTCTTTCACTTTGTTTCATTC[A/G]TTCTCATATTAATTTTCCCTCTTTAAAAATGAGCTAGGACATATGCTCATGGGAGGTAGAAATATTCTGTCTTTAATTGTACACCTGATGATGACTACAT
5	85279449	SCAFFOLD144700_3432	0.05	0.06	ACTGAAATTAAAGAAAGAACAAGGTGTGAAATTAAATAATGAGTCAACATGGAAAATGCAGCCAATGATGATTGTGGAGTCATATTTGATTAACAGATAT[A/G]ATTCAATTTAAAGTCTCCAGATAAACAGCTTAAGCCTAATTAATAGGTGAATGTTCTTAACTCAGAGCACAATGTGCTTCAGACACATTGTACACAATCT
5	85279642	SCAFFOLD144700_3239	0.02	0.04	ATTTCAGTTTTGACAGGTATCATCTCAGTGAAGTCAGGAGCTACTCATCTGAATCAAGTCAACAAGGAAAGGTGTTCAAACTGCAGGAAAGGGATGAACA[A/G]TGTTGATCTGAAGAGAAGATTTCTATTTATTAGCAATATCTAATTTAATCATAGATTTTTTTCCTGTCACCTGCAAACCTCACTTCACTAACTCAGTTCA
5	85482238	SCAFFOLD130352_25743	0.49	0.37	TCTGTGATTCTCTTTTTACTTTCCCTGTGCTAATACCACATGATTTTTAATATTATAGCCTTATAATAACTATGGATTTGGTACAATAACAAATATGTTT[A/C]TTTTTTTATGGGTTCTTAGCTATTCTTAGACATTAAATATTCCAAATTTATAAAGTTTTGATTAAAATCATATTGAATTTATAGGTCACTTAAAGAGGCT
5	85482365	SCAFFOLD130352_25616	0.49	0.37	GTAATTGACTTTTTTAGTACACACAGAGTAGCCTTGGAATACAGTTCCTCTTCCACTATGTCAAAAATCACTGATCATCATGTGGCTTAGGAAAAATCCA[C/G]CCTTTCCTCACTGAAGTTTCCATGTATCTGTGATTCTCTTTTTACTTTCCCTGTGCTAATACCACATGATTTTTAATATTATAGCCTTATAATAACTATG
5	88228164	SCAFFOLD116588_4628	0.48	0.09	CAAGGCTAAGAGTCAGACATGACTTAGTGATTGAACAACAAAGAGCAGAGGAACATCTTAGAGCTCTGTCTACCACAGCCCCCCACCTTTTAACTTGATA[A/C]ATGTATCCCCAAATACAGACTACATCTCCCAGGCTCCCTTACAGCTATGAACAGCCATGTCTCCATGCTGGCCAAAGGAATGTAGAGAAGCTTCCCAGAA

5	88749463	SCAFFOLD10352_4140	0.22	0.30	GTCAATTAAATGAGACGATTCCAAGCTAGTGAAATCTGACCTTCAACAGATGAGTTTCCTTCTCCTCATGAACCCTTCCCAGAACTTCTCCTACCCACTG[A/G]TGGAAATGATTCTTCCCTTCTATTTCCTCACTGTAGTTTTTGGCGTGCCTACGAGAACCCTGTGCCCACTTGCCATGGCTTATTTAGGTATGTATCTTAG
5	89249253	SCAFFOLD59715_196	0.35	0.20	TGATTTACTTTGTGGTATAGCAGAAACCAACAAAAACATTGTAAAGCAAGCAAACTCCAGTAAAAAAAAAAAAAAACAAAACTTAAGAAAACAATGCCAA[A/T]AGATTGCTGGATGCACCGCTGCCACACACTTTCAATTTATAAAAAGAATGCAGTATCTGTAAAGCACAATAAAGCAAAGCACCATGACACAGGTATGCCT
5	92854996	SCAFFOLD106566_3136	0.25	0.35	ATGGGCTCCTCACTAATGCATTTCATGGAAGGAGATTATTTTTTTCCCTCACTTATTTACATGAATCTCAATCTTGTTGTTTTCCTTTTTCTGTTAAACA[A/G]TTTCTATGCAGTTTCTTTGTTTCTTCTTTCTCCAGTCATTGCAGTATGATTTTTGAAGTGATCCTGTTTTTCCTTAAATCCAGTATGACCATGCTGGGCA
5	92855031	SCAFFOLD106566_3171	0.25	0.35	TGCTGCTCTCAGACTGCTTTCTGGGTTAGCCTTGATGCCCAGCATGGTCATACTGGATTTAAGGAAAAACAGGATCACTTCAAAAATCATACTGCAATGA[A/C]TGGAGAAAGAAGAAACAAAGAAACTGCATAGAAACTGTTTAACAGAAAAAGGAAAACAACAAGATTGAGATTCATGTAAATAAGTGAGGGAAAAAAATAA
5	93343336	SCAFFOLD130785_8516	0.27	0.44	AATAAATAACAACAACAAGAGAGTAAATCCTAAGAGTTCTCATCACAGGGAAAATATTTTTTCTATTTCTTTCTTTCTTTTTTTACTTATATGACATGAT[A/G]GATGTTCACTAATTGCCTGGAGAAATGTCAATAACCTCAGATATGCAGATGACACCACCCTTATGGCAGAAAGTGAAAAACTAAAGAGCCTCTTGATTAA
5	93798102	SCAFFOLD171924_3268	0.23	0.34	ATTGTTAACAGTCCATAAAAAATCTTCTATTAACTCCACTTTCCAGATGGAGAAACTGAGGCAAAGAAAAGTTAATTTGCCCAGAAGTCACACAAAATAC[A/G]GGGCGAAACCAGAATTTGAACTCAGATAACATGCTCAGTGCCTTGCTCTTCCCTTCTCCTCCACATCCTACTGTACACATTTGGCAATAAACATCATGCA
5	94444028	SCAFFOLD60743_10641	0.26	0.34	GTTTGGTCTTCTTGCAGGCCAAGGGACTCCAGAAAATCAATGCTTTGGCTCTGTAGAAACCTGGTGAGGCCGGGACAGTTTCTCCTCATGAGGCCTGCCA[A/G]GCAAGGCCTTGAATTACCTATGGTTGGGGCATAAAAGATCAGACAATTGTTTGGCCTGGACTCAGATTCCTCAGTGTATGTAGTTTACAAAAGAAATGCA
5	96419167	SCAFFOLD154895_214	0.16	0.12	AGACAGCCACTGCTACATCCCTTGCCATGCAGGTCCCTTCTTAGAGACTCACAGAGGACTGTATGTGAGGACTCAAAGACCTTTCTTTGAGTCTATCCTG[A/G]CACCCTCAGATTGAGTTAGTACTTGGATCACAGCTCTGTGTGTTAGAGTGAGATGTTATGCCCCTCAGGATATTCTCCTCCTTCATCTTGCTAACACTTA
5	98531522	SCAFFOLD246578_8287	0.38	0.42	ATTAACAAGAAGTCTGTACTTCCTGCTTCAGAATACAATTTGGGGACCAGAGAAACAAGATGTTTTTATAAATTTATAATTCTCTGCCTCCTGTTGTTCA[A/G]GAGAAACCCTGTTGGTTGGCCTCCAGAGTTTGATGAGGCCTCTTTAAAGACTATTTTTATGCTCCTTTCCTCTTCTGTTTCATTAATTGCTACTGCTGAT
5	98531638	SCAFFOLD246578_8403	0.37	0.41	AGAGAATTATAAATTTATAAAAACATCTTGTTTCTCTGGTCCCCAAATTGTATTCTGAAGCAGGAAGTACAGACTTCTTGTTAATAATTCCCAATCTTGT[C/G]CAAATTAATGCACGTTAAAATAAATCATAAAGTTGAGTTAGAGATCTCATTAATTGAAGCCCCAGCACATATTTTAGAGAATCTTAATTATTGGGCTAAT
5	98531793	SCAFFOLD246578_8558	0.37	0.41	CTTTAAGTCCTTAATATATTCCCGATGATAACTAAAAATGCATTCTTCACCCAGAGTTCTGGAAGAAGCACACATCTCTACACTACTACCTCAAAGAAGC[A/G]AATTCTAATCTAGAACTGTTTTTCTGCAAAAAAGTAAAAAGTGGAGTTTGAGTAATTAGCCCAATAATTAAGATTCTCTAAAATATGTGCTGGGGCTTCA

5	104421187	SCAFFOLD140224_4081	0.38	0.39	GTCAAGGGTATCAAGAAGGTCCTGAACACGTGGCCCAACATGGTATATATTTCATTGAAGTGAATCATTTGAATTAATGGGCTCTGAAGAAAAGATAGGG[A/G]GAGATTAGGCTTAAAAAGCATAAGTTTGAATTCTGACTTTACTGCTAGGATGAGCTGTGTGACCCTGGACACATTTTATGATCTCTGACCCCTGATTCTG
5	104805575	BV105396-56-Y	0.48	0.43	TTTTTTACAAGCTTATACACTGCAATTATAAATGTCTTAAACAGACTCTTTGTATGTTTTAAATCTTGTTTTTCCTTCTGTAGATGTTAACTGGTAACCA[A/G]TGTACAAAAGGGTTGGATCTCACCTTCAGGATATCTGAAATTTAC
5	105498357	SCAFFOLD115022_16294	0.20	0.31	GTGTTCAGCTGAGTCACATTATATTCGAGGACTGCCCTGCAGCAGTCCCAGACCCAGCTGGATGTGGAATATGGACCTTTCGGGATGAGATCGACTATAG[A/C]ATCATCACTAAGGGGCAGAGGGGTTTTTAATCAAAAACAGAGGACGTGGGGCTTCCCTGGTGGTCCAGTGATTAAGATTCCGCCTTGCAGTGCAAGGGAC
5	105498792	SCAFFOLD115022_15859	0.18	0.32	CTACTGCGTTGACAGGTGGATTCTTTACCACTGAGCCACCTGGGAAGCCCACAGAGGCATGAAAAGCCTTCTTAAACAACAAGCCTGAAATAAAAGGTCC[A/G]AGAGTCCCTTGGAGGAACTGACCTCTCTCTCTGCCTTCTCCTACCTCAAAGGAAACGGGGAATGATGGAGAGGTGGGGTGGCTACTCAACATCTGGGTCC
5	112776759	SCAFFOLD49992_3357	0.22	0.25	AGTGACTTTCACTTTCACTTTGCTGTTCTTAGAGGTATAAAAATCAGGGTAGATGAGGTCGAATTTATATGCAGTACAATCACCCTTCGGAGGGTTTAGC[A/G]TTCTAGGAATTTTGACAACCCGATGGATGGTGTACCCACCACCACAATCCTGACGTAGGACATTTTCATTACATCCCCCCAAATTGCTTGTGCCTCTTGT
5	114285326	SCAFFOLD220063_9756	0.25	0.18	TGTTGGATTTTTCTCTTTCTTTTTCTTTCTTTTTTTCTTTAAACAGCATGCATTATTTGCATAATTTTAACAAGAAGGACACAGGTGGTAAGCGGCATTC[C/G]GATGCTCTGGAGGTGAGCAGAGTGAGGCCTGGAGAAAGGGAGACTGCTGAGGCTTGAGGCCCCCGGGGCCCTGCCCTAGAGTCTGTGCTTGTGTGCATGG
5	114771507	BES10_Contig488_621	0.12	0.22	CTGGCTTCTTGCTGTGATCCCCATACCTGTAGAATATTCCTCTTTTATCTTAAGAATCCGTTATGTGTGACCACAGCCAGTTAGTTTCATGTAACTTCCA[A/G]AACTGAAAAATCCACACATCCAAATTTCTGTTAGCTTGATTTGTCTCTCTCACTTGAGAAAAAAATAAAATATTGTCTCTGCTGCCCGTTTGCCAGTTAG
5	115695379	SCAFFOLD245168_20619	0.24	0.40	GGATGTGCTAGGGTCACTGGGAGAAGGAGAAGGTGGGCAGAGAGTATGCGTGGGAGGTCCCGGGAGAAGTCAGGTACTGTGCAACCCTGCCCAGGGTGCC[A/G]CTGGGTTGGAGCCTGGGTCCTGGAGTCACTGGCTTGAGTTTGGACCCTGGTTCTGCCCTTTGGGGGCTTCCCTTGTAACTCAGCTGGTAAAGAATCAGCC
5	116781074	AC137534-31795-1	0.45	0.45	AAATTGTAGCATCCTGGGGACCTTTACAGTTTTCACGCTGTTGCCATCATGTACGCATTCAGTAGACATTCATGGAGCGCCTACTGAGTACCAGGCAGTG[A/T]GGAGAGTGTTGGGTGCATGGGGTGAACAAAAATGTGACCCCAGCCTTGAAGGAACACTGCTGGGGCGACTGCAGTCCAGGGCCCTGACAAGTTGCTTTTG
5	116851974	AC137534-32977-2	0.37	0.35	TTATTTTTGGCGGTCTGGTGACATCCACTGCCCTTGGCTGAAATCCACTCCAGGCAGGCTGCGTGCATCTTCTTGGGTGAGTCTTAGGAATCCCATGGTC[A/G]CCGCTCCTGGGCTGTGGTTTCAGTGGGGGTGAGGTTCCCAGCCCCCGAGCCTCCTGGTGCCAGCATCATTTCCACACCTGGTGACTCACCCTGTGGGTCC
5	118137032	SCAFFOLD135556_32619	0.32	0.37	ACAGATGCTGCAGACAGTGCAGGCGCCAGGGGCTGACTCAGGTCTGAAGGCCATCCTGCCAGGAAACAGGCTGCCTGCTGAGGAGTGGGCTCCCCGTGAC[A/G]GGTTACAGCTGTACAACGAACGGATGCTCCCTCCTCCCGACCAAGCCTTCCCCCAGCCCGTTCTCCCCAACCCGGTCTCCCTGTCCTTGAGTCTGTGGAC

5	118175927	SCAFFOLD116319_3651	0.25	0.34	CCACCCCAGGGCATCTCTGGCTCGAGGGGGTGCTGTTGTGTCCTTGTTCCTCAGGTCATGGCTCAGAGCCCTAGTTAGGTCCCTAGCCCCTCATCCAGCA[C/G]CTGGCTTGGAGGCAGCTCCCCCTGCCAGCTTGTCAAACAAATAACTTGAGGGACAAGATCAGAAAAAAAGAAAAAAACTAGGAGCTGACAAGAAGCAACA
5	118682287	SCAFFOLD15017_7532	0.36	0.31	TTCAACAAAAGGCACAAATTAAGGACTATCCACCTGCACGCTTGATGTATAACTCATTTTTATTTGCTCCTGAATTACCAGAGTTTGGTGTGCCATAAGG[A/C]TGGGCTCTGCAGGAACTTAGATCCAAAATGGCAGCTGAGCTTCTGAAACTCACCAGAGCCATAACGCTTAACATACGTGAAACCAGGTCTTATTTATCAC
5	118699873	BES7_Contig519_1395	0.32	0.40	AGCGTGATACATTAACTGGAAAGAGAAATTACTGAGTAGTTAAAAACTTTATCTCAAATGATTAATTGAAAAGCCAAATTGATTAATAAAGAGACATTTT[A/T]AAAAGGATCTTAACTGTATTGAACTTAAGTAGTAATTCTTAAAGTGTAATCAAATAGAGGACTTATCAAAGCTATAATGAAGAAAATAGAAATCATTTGT
5	119739697	E494C23-36531-2	0.28	0.44	ATTCAGAACACRTCACTGATGACTAGTTCCCTGCCTGGTCAAACTATTTTAAAATAATTATTAAATAGGGAGAATAATCCTGTCATTTAACAATGAACAT[A/G]TTCCTAGATGCCAAAAACTGTCTCTGAGTGGTTTCCAAACTTCAAGGCCYGCGAACAAAATTCTTTGTGGTAAACACACCAGGCTGTTGCTAGGAGGCAA
5	119739786	E494C23-36531-1	0.41	0.26	GCAGCCACAGCCTTCGCCCTTCGTTCACAGACATGCCATTGTGCACACATCAAATGCAACCCAGTATAGTGTCACAGAAAGCAAGCCAGATTCAGAACAC[A/G]TCACTGATGACTAGTTCCCTGCCTGGTCAAACTATTTTAAAATAATTATTAAATAGGGAGAATAATCCTGTCATTTAACAATGAACATRTTCCTAGATGC
5	119753900	E494C23-36521-2	0.48	0.44	CCTATTATGCAAAAGCCTTCCCCATCAGCCTTGCTTGCTCTCACCCCACTAAGACGGGTGAGGCTGCCAGCTCTCAGAACGGAAACTGACGCCAGCCTCA[C/G]GAGACCCTGTTCCTAGACCCTGTTTCCCTGTGGGGCCCACCCCTCCCTTCCCGTCAGGGCCCCTGGGGAGGTGCAGCTGCCTTCTCGTTACAGCCTCCAC
5	119759740	E494C23-36525-4	0.30	0.49	AGACTGTGAACATGGTCCACATCCAGAAAGGAGTGGTCGGCGCAGACCGGAGGACTGCAGAAAGCATCACAAAGAAGCTGGTTGTGACAGCGACCTCAGC[A/G]GTCAGCTGCATTCGGTTAGGCAGGTGGGGGTGTGGTGGGCAAGAAGTGTATTTTTAGGCTGGACTAACACTGAGAACATAAAACTGGAAATGCAGCATCT
5	119760092	E494C23-36525-2	0.28	0.26	TYTGCAACAGTTGATTTTGTTAGGGATTCCCTCCTTGTTGGCCTCTTCTCCCAGCCAGCTGGCTCATTTCTGGGGAAGGAACCAGGTCTTCTAACTTCCC[A/G]CATTCCCAAGAGGCGAGACCCCTCAAACCTTGCTCAGTCTGCTGAGCARATGCCTCGCCCGAGGCAGAGGCCCTGGGATTTCTGTAAAATTCCAGGGAAC
5	119771496	BV105351-426-M	0.47	0.44	GGGCCTGAGCTGGCTTTATSTTCATCTCTGGGAAGGCATTTCCTTCCCTCTCAGGGGTTACCTGGAGAATTCAGGGGCCCTTCTAGCTAGAACAAAGGCT[A/C]ACGGAAGGAGGTACAGGTGGGGCCAGGTGTAGGAGGGCAACCCTCCCCTCCCCTCCCCTCTCAGGACGGCTCTGGGAGAAACGCATGTGCTTGGCTGGCC
5	119787096	E494C23-36489-3	0.20	0.10	GGAATAAAGAGCTAAAGTCACATTTCCTCCCAACTTCAAGAAGCTCAAATCCTAGGAAAAGCAGACAGACCTAWAAATAATATAGATGCAGAGAAGGCCA[A/C]ATTTGGTCACTGCAGCCGGACTTACAGAGATATCCCTATATGTGTAAAATCAGCAAATCGAAGGACCATGCATTTCTGAAG
5	119866976	E494C23-36507-3	0.47	0.42	TAAAGCCAGCATCTGATAAATCAGTGTCTTATAAGTGCGTTAGTCCTTGTGGCCTATAATTCTAGGGACGCTGACAAAATTAAGTTTCTGATTCTGCAAA[C/G]GTGAGGAATAAACCAACCCTGGGAACCCCCCTCCATATCCTGCTTGAGACTCTACCTGGTCTTCCTCCTTAGTAACTCTCTCCTGGAATGCAGAACTTAT

5	119869693	E494C23-36481-2	0.47	0.42	CGCCCCAGGTRTCTTCCTTGACTTGATGCACCTAAAGAAACGTGGAGGCTTCGACAYATCCTTGTTCTACAGAGATGTTATCATAGCAGAGAATGAGGAC[A/G]TAGGGATTCACTTTGAGGAGTCTAGCAAGCTCGAAGACCTGTTGAGGAAGGTTCGYGCCAAGGAGACCAGGAAGCGTGTGCTCTCCAGGTGGGTATTAAG
5	120785991	SCAFFOLD291363_7096	0.26	0.30	ACTTTTTGATCACTTCGAGAGCTATTGGAAATGTAGGTATATGGATGTGGACGGTGAGACCACGGGGCTCGGCGGGGGCGGAGGGTGCTGGGTGATGCTG[A/G]GGGCCAAGCAGAGCCGACTTGAAGGCCTTAGATGGATGCCAGGACTTGGGCTGAAACCTGCCCACCCCCACTTGCACACGGGCAGTTTGAGGGTCTAGGA
5	124744457	SCAFFOLD100797_15399	0.37	0.38	TTCCAGGGGGAGAGGGCGGCCCTTGGGCAGGGAGGCACACACAGCTGTGGGGAACCGGCACGTCTGGGTTGTGAATGCAGACAGGCAAGCCTGGGGGAGG[A/G]GCAGAGAGTCTGGACAGGTGCCCAAGAGGCCTGCAGCCTGCGTGCAGGGGCCTGAGTCTTATCACACAAGTGGTTCGGTGTGGACAATGCCTCAGGAGCC
5	125064493	SCAFFOLD195099_30552	0.07	0.09	GCTGACAGGGCTCCTGCTGCAGGTGGGATTTTCCCAGCATGCACTGGTGGAGGGAAGCCGTTCCCAGGATTTATACAGCCCTTCCTCCACGGAGCTCCAA[A/G]GCGCACACATTCCATCATTTTAGCCTTTTATATACTCACTACAGTGGTTTGGGAAACATTTCAAAATTAGATAGGCATTAGCTTAGCTTATAAAATTAAA
5	125364393	SCAFFOLD90967_7491	0.26	0.34	TGGCCCACATGCTCCTGCCAAACCGGAAACCAGCTCTTGAGTAACACTGGGTGCTGAGTGTGGAGCTGAGTGACTGAGCAGAAATCGGCATCCGGCCTCC[A/G]GTGACCTCGGATCCTGGTGTAGCCCCGCCCTAGGCAGGCTGCCTGGTATTCTTCGAGGTCTCCCTCGGGCCCCCTTACTGATTCAGGATGCCAATGAGGA
5	125364418	SCAFFOLD90967_7516	0.49	0.40	GGTGGAGCCTCAAGGGCTTTCAGATTCCTCATTGGCATCCTGAATCAGTAAGGGGGCCCGAGGGAGACCTCGAAGAATACCAGGCAGCCTGCCTAGGGCG[A/G]GGCTACACCAGGATCCGAGGTCACCGGAGGCCGGATGCCGATTTCTGCTCAGTCACTCAGCTCCACACTCAGCACCCAGTGTTACTCAAGAGCTGGTTTC
6	7015203	SCAFFOLD10121_13387	0.34	0.42	TGTCAAAATCCTCTCTAGGCTCCTAAATGAAATGTTTAAATGCGCACTGAAGTGGCTTAAGCACCCACATATAGTAGAAAGTATATGCTGTGTGGCTCAA[A/G]TCATACTGACATTAGTCATGTCTGTGGTGGGCATTTATAAAAATGATTAGGTTTGGGGGCATTTGTAGTCCAGTAGAGGTGAAAGTTGTATACAATGTGA
6	7015368	SCAFFOLD10121_13552	0.31	0.26	GAATACTGGAGTGGGTTGTTATTTCCTTCTCCAGGGGATCTTCCCCACCCAGGGACTGAACCCCATGTCTCCTGTGTTACAAATGGATACATTACCACCC[A/G]GCCACCTAGGAAGCAAGCCCAAAGTTGTGAATACTTATGTTAAAACTTGTTCTTTTTACTCAAGTCACATTGTATACAACTTTCACCTCTACTGGACTAC
6	7348764	SCAFFOLD103365_533	0.05	0.09	TTCCAACCTTTCTATTAAAGCAAAACAGAACAGGTGTTTCTGTATTTGTGATGCTTATCACTTTTACCTAAAATATCATTTAAATACATGTGCTAAAGTC[A/G]TTTCCTTTAAAAGCACAAGCAGACAGAAATTTTTATCATAGTTTGTTAATCAACATTATTTTGGAAGTTTCAATGAGTGCAATAAAAATATTAAATAATT
6	13856009	SCAFFOLD106320_3036	0.27	0.41	AGTGAGACTCAAGGCTCTGTGATTTTAAAAAAAGCCTTTAGTGATTCTGTTCAGTGATTCTGCAATTGGTCCAGGGACACCAGTCTATGGGCTCCATGTC[A/G]TTGGAACATCACTAACCCTTTCGTACTTGGGTCCTTCATATGTTCCTAACTGTTTATAGCTCAACTGTCAGTTAAAGACTGTTAAGTGTTCTAATATGCC
6	13856295	SCAFFOLD106320_2750	0.26	0.41	AGAATTCTTATTCTTAAAGCAAGATCACCTTACTTCAGTGATCGCTGACCACCAGTATGTCAGCACCACCTAGGAACTGAGATGCACAGTACTGGGCCCC[A/G]CCCTAGGTCTTCTGAATTAGAATTTGGGAAATGGGCCCAATAATATGTTTTAGCAAGCCTTCCTACTACAGGCTATTCTCGAACCATTATGGAGCCATGG

6	15458394	SCAFFOLD61725_891	0.19	0.31	CATGGGTCCAATTTACATGGGTTAGGCAGTACAAGTGCCTCTTCAGTTGCAATATTCTTCATAAGCTGCAATCCATTAATTATGGTTTATTTAGTTCACA[A/G]TGACTTAGTAACAGCAGCAGCACGTGGTCTCCTGACCACATGGCTTGATGGCCATTATGGCCTCCCAACAGGGCTCCCAGCAGGGCTTCTGCAGTGCTCC
6	15458483	SCAFFOLD61725_980	0.20	0.32	TTTAGTTCACAGTGACTTAGTAACAGCAGCAGCACGTGGTCTCCTGACCACATGGCTTGATGGCCATTATGGCCTCCCAACAGGGCTCCCAGCAGGGCTT[C/G]TGCAGTGCTCCCAGCCTGTGTAGAATGCAGCAGCACAGCCCCTTAGTGGTGCGAGTAACCGAAATTGGATTTGATTGTCTCTCCTCTGTAGTTTGGTTTG
6	15458602	SCAFFOLD61725_1099	0.20	0.31	CCAAAAACAACAGCTTTCACCAGGCCCCTGCTCCTCAGCTGTGCCACTCCAAAACAATGTTTGCTCCTCATTAAAAATCTGCAAAGCCAACTGAGAGTCA[A/G]TCCACCACAAAAGTAGCCCAAACCAAACTACAGAGGAGAGACAATCAAATCCAATTTCGGTTACTCGCACCACTAAGGGGCTGTGCTGCTGCATTCTACA
6	18183979	SCAFFOLD45958_4009	0.45	0.31	TTAGTGGGAACATATTCCCACCAGATCCACACTAAAACAATGTTCGACTTGGAAAACATGATACAAAAATAAAGCAGATATCAGATCGAGTGCTGAAAGC[A/G]CTCAGAGACAATAGTTTACCTCCCACGAGGATTCCTTTCAGCTGGTTAAGTGCTAAAAGTCGACTCCTTGTCGGTACTGTACCTTATTTCCTATCCCCGA
6	18204967	SCAFFOLD80477_8767	0.40	0.47	TATCTCTTAGTACATAGTTGGAGAAGGCAATGGCACCCCACTCCAATACTCTTGCCTGGAAAATCCCATGGATATGGGATATCCCAAAAACCCAACAGGA[A/G]GGTGCTGCAGTCCCTGCCCCAGGGACACTTTCTGACCCCTCTCCAGGGGCTGCTGACAACACCAAGCACAGCTCAGACCATCTTGCTTCTGGCCTGAGCC
6	18544027	SCAFFOLD42119_3327	0.33	0.19	GGCCTGGAGATAGTTCCTGGAGGCTTTCCTTCCATGCCTGCCCTGATTCTGAGTCTAAAACGTATGCTCCCTTCAGCACCCATAAAGCATGCCTTCCTGT[A/C]TGCCTGGCAGCTGGGTGCAACCTGAGAAGTCCATGCCAGTTCCCTGGAGGCTTGCGCACACCCTAATCATCCTCTGGCAGGTTGGAGAATGCCTGGAAAA
6	21322548	SCAFFOLD135633_16260	0.35	0.28	AAAATAAGAGATCAAAAACATTGTTATTAATTTAAGGAACAGGTAACTCTTTAAGTATTTTCCATGCAGTATTTTAAACATACCAATACTAAAAAAAAAA[A/T]TTTTTTCCTGAAATTCATGTGTAACTGGGCATCCTACATTTTTATTTGCTAAATTTGGCAAGGCTAGCTGCACTGCATGGATCATGCCAAATAATATTGA
6	21620639	SCAFFOLD11046_21118	0.23	0.24	CTTTCATTTTTATCATTTTTTCCCAATTTTTAATAAAGCTGATTATTATTTCTGATGTTTGGTCACTTACAATGTGAGCTTCTTTCCAGTAGGAATTATG[A/G]AAATGCTACCAATTTACTTAACATTACCTGTTTAAACAGGGAACATGTGACAGTGACTTCCTGGACAAATTGATGAATTTGTGCCATTTGTTCATCCTCC
6	22363193	SCAFFOLD295396_5116	0.16	0.10	TATACACTAAACTTGTAAAGAGAGACTGTAATAAGCTTATCAATCAACATTTTTATTACGATGTACAATACATGCTTGTTGATGGTTTAGCAAAAAGAAA[A/G]GGAGGATTTTTCTTAGACACATACAGTACTATAAAACAACTTTAATCCTTTGTCTTTTATCCATTAATTATGTTTGCCTTCTACCAACTGGATGTGGGAT
6	23332868	SCAFFOLD289496_5970	0.38	0.46	GTTCATCACATATGGTGCTGCAGTGAATGCGGTTGTATTTCTGCCCGTATATGTGGATATACTGGTAGGATAAATTCCTACAAGTGAAACTGCTGTAGCA[A/G]ATGTCTCTGTTGATAGATTTTAAAATACTATTGTCAAACTGTCTCCCTGAAAGGTTATAACATTTAAATTTTCATGAATAGCATCAATTATTTTCAAACT
6	23333022	SCAFFOLD289496_5816	0.38	0.45	GCCACTTATTTTCCACATTTCGTACCATCTGGATATTTTTCTAAACCAGTATCATGTAAAGAGTGTCCTATTCTTCTGCTTAGTTTTCCACTATACAGAC[A/G]AACCCCACTTATCCAGTGCCTTACTGACAGACACTTGGATTGTTTCTGATTTTGTTCATCACATATGGTGCTGCAGTGAATGCGGTTGTATTTCTGCCCG

6	23882877	SCAFFOLD195156_10467	0.17	0.19	AATTTAGAGACAAAGAAAAACCTTTATGTTAGGCAAAGATTTCAACTTTTCTCATTAAAACTGAAATTGAGTAATCAAAGCATTTAGTTTCAGTAAATCC[A/C]AGGAGGGCACCGCCCTGGGTAGTTAAATCCTGATAATAAAGGACTGACTTTCCTGAGGATAGGTCAAAAATGCTTTGATTACACAAGTAGGGGGTGGGAG
6	24997938	SCAFFOLD125519_9311	0.30	0.18	TTGTCTAGGAACTTTGGAACTTTAGTAGTTAGAATCCAAGTTTGAGGAACTATCCCTCCCTGCTTTTCCTCAGTTGACATTAGAGGTCATCTTCTTTCTC[A/C]AAGTTTTAACGTGATTTTATCTTCAGGCTGCACTTTTCTTTGTTTTACTCTTTTGAAGAACTTCCTCACCCTTGCTTTTCCCCCAGATATTAAGGACCTT
6	25649145	SCAFFOLD135049_16999	0.50	0.02	GCAGCAGTTCCATCCAAGCTCACACAAGATGAAGAAGTCGTTGGCAATAACCTGAAGGCTAATACAGAGCAAGTGTCACCATGTCCCTGGATCAGCTCCA[A/G]AAAAGTATGCACAGTGGGAATAACTGCTGCACTCTTTTGGAGGGAGCAAAGGTACTAATACTGAAGTGTGGAGACAGAAATTGAATTGGCACTCAGAAGA
6	26486541	SCAFFOLD136974_34486	0.34	0.45	ATGAAAAATCCTCTGGGGAGCATTTTCAATACAGGCATCAGAGTTCTTTTTATACTCTGCAAGTGTTGTCCGTAGCCAGGGTTGAGAGTCACTGCAATTG[A/T]CCATTTAGAGAAGGAGAATTATATGATTATTGGCTTTGTGTAGAAATGTGTGCGATAGAAGTGGTAGGCCATCATACCAATTGGGAGGCTGGTGTAAGTC
6	27574152	SCAFFOLD15216_16358	0.01	0.02	GAATGCATGTTAAACTCCATTTGACAAAATTCTATGCAATGATGTTAGTTTAGAAAGAAAGGCACTTTCAGGCTTTGCTAGTGATGATTTGTTGCAAATT[A/T]TCTAGAAAGTAATATGGCTAGTTATCAAGAGCATTAATAATGTTCAAATACTTTATCCTAAGATTTCCACCTGTAAAATATGATGAAAGTTTACACACAA
6	28147518	SCAFFOLD110150_24658	0.32	0.43	GCTTGGAAAATCACAAGTGTGTTTGGTGAAATACACTTTCATAGTTGCCAAATAAAGGATTTGTTGATAACATATCTACCTGGGTAAGGGAAAATTCTAT[C/G]GAAGCCCAGCATATAAGATAACAAGGATTTATGAATTGCCTACTATGAACACGGAGAAGGCAATGGCACCCCACTCCAGTACTCTTGCCTGGAAAATCCC
6	29139241	SCAFFOLD166116_3594	0.23	0.40	TTATAGATTGTTGGGACTGCTATTCATGTAACTTATTCTCCTGGATATACCCTTTGACGTGTTTTCATAAATATTTTCTTTGTCTCTCCAAATGAATTTC[A/G]AACTCCTGAAGCTTAGAGGGCAGATAAAGTGCTTTATTAGCAATATACCCCAGGTCTCACATTCACTTGCTGCTTAGTAAATATTTCCTGAATAAATGAA
6	30816951	SCAFFOLD80071_25898	0.34	0.29	GAGACCTGGATGCCTCTTCCATGTCCTTAGGAGGGTTGCTTAACAAGGATCTAGTATGAATTCTGATTGTGACAACCAGACTACAACAGGAGACGCTCTA[C/G]GGCCCTTTCTTGTTGAACATGGAATCCCCGGGATCCAGAACACAGAAGGCCAGGAAGTCAGTAAATGTTAACAATTTAATGGCTCCTCAAGCTTTGCAAG
6	31521513	SCAFFOLD125267_26732	0.48	0.41	CTTATGTTTCACTACTGCCACCTGGGAAGCATAGAAATTTTTATTGATAAAAGAGGTTATTAAATTGACTCATCCGATACTTATATTAACCGCATATACT[A/G]TATCCTCCTTTCTACCTCATTGACTTGCTTTTGGGTCTTTCATACTTGTATTCAAAACAGACTAGTTTCTTGTTTCTGTACAGTGAGATGCGCTGAGAAA
6	31579805	SCAFFOLD282047_3224	0.28	0.30	TCCTTTCATTCAAAATGCACACTGATAACTAAGTTGACAGTTTCTATTTATAGCAGTAATTCCTTAGTCATCATACAGCAAATACTATAACCAAGTAAAG[A/G]TGCTTTCCATTCCAAGTGGAAAATGTGTACTTTTTTTTTGGGGGGGGGGGGGGGGCGCGGGTTAAAACATTTCCAAAATCTTGGCTGGAAATAAGAAGAA
6	31895195	SCAFFOLD196877_11917	0.30	0.34	CTCTATTACCATCTGAACCACCAGGAAGCCCCTCTCTATTACTACGAGTACTAAATATAGACAGACAGAAATAATAACCCAAAGTCCAACAGACTTAGTA[A/G]AGAAAGAAGGAAAAGAGAGAAGCAATGGAATTGCTAGAAGCCCTCTGAGTACCTGGGGCCTCCTCTGCTGAGGCTTGGCTCAAGGGGCACTGAGAAATTC

6	31937563	SCAFFOLD141562_8222	0.14	0.26	AAAGTGACTTCTTTTTTCTTGAAATTCAATGTGCCTATTAAATTATTCCAATGTGTCATTCATTAGTAGTAGTTACTGGATGTCTGACAATTTTTTTGTT[A/G]TTTGAAGGATGAGGAAAAAGGAGGAGACAGTAAGAGAGTATATCAATATTTTTCAATACTCAATCCTTCTAGATCTCTCACTTCCAATCATAACAAGCTA
6	34391663	SCAFFOLD50174_9116	0.37	0.24	GTGGTCCTACTGACAAGAGCTCAAAGGTCCCTAAAAGAAAGATAAGTCAGAGTGAGGGGATGGAGGAGGGCCTTGGGAAAGTTGGGTTGCAGCCCAGGTC[A/G]TGGATAATTGTAGAAGCCCTTCTAGACTTGTTAGTAGCATCATTTTGCAGAGAAATAGCTGAGCATATGAATATAAAAATAAAACTGAAGCTTTAGAGAA
6	34391775	SCAFFOLD50174_9004	0.36	0.24	CATTATGTTAGTATTGAAAATGAGTCCCATATGGTCCAGTGTATTCTGTCTACTGTTGTCTCCTTTGTTAGCTTAGATTCCTGTACAGAAAGAGATGGCA[A/G]TTCACAGTTTATTCTCTAAAGCTTCAGTTTTATTTTTATATTCATATGCTCAGCTATTTCTCTGCAAAATGATGCTACTAACAAGTCTAGAAGGGCTTCT
6	40981871	SCAFFOLD175661_7643	0.11	0.20	GTTTTTGCATTTGTTCTCCCTTGATAGTGTTTAAGTTTAAAGAGAAGAGCAATTAGTAAACTTGTTCAATCAGCATGTCATATGTCAATGTAAGATACAC[A/G]CTTACACGTAGTTGGCTTTTTGTACCAGTTTCCTCACTGTGAATATTGAGAGTCTAGAAAAGTCTATTCTACAATATCTTTGATAGTATTTATAAATTCT
6	40989163	SCAFFOLD175661_15307	0.43	0.35	CTCTTCTTCATCACAAGCATAGGAACAAGGGACAGACACACGATTCACACATGCACAAGTGCCATGGTGCACAGGATGTCCACGATGTCATTACCTGAGC[A/G]TGCCTTTCTTAGGGCAGCTGAAAACTTCGTCAAGAAGAAGCCATAAGTCAACTCGGAAACTACACTGACATTGGGCTCGTTAAGTTCAATCACAGTTCTC
6	42810750	SCAFFOLD186587_2456	0.36	0.28	TACAAGCTAAAAGGTTTCCAAAAACCTGCACGGTAATACATGTGTGAGGAGTCATCTCTAACAGATAAAACAGTGAAACTCATCAAAAAGCAACTTCCGT[A/G]TATTCAAAAATATTATTGGCAACAATAATACTCCCTCAAACCTACATTACTGTATTTTTCATTCAGTTATGTTATTTCACATCTTTGTGACTCACTGATC
6	43422441	BES8_Contig582_862	0.13	0.18	CTTTTTCATCCTTGGAGAAAGTTTCTACACTATTAATGGGAATCTAAATTGATCTTCTAACCTTCCTAGATGTCTAGGAACAAGCTTTTTGTAGAAGGAT[A/G]GTGATTATAGAACCTTTCTCAATTTTGCTAGTCAGACACCTTGAAGAAATCAATTAGTAGGCAAATTATTAACTAATTGAGCATTTAGAGTGTTCTTACT
6	44507206	BES10_Contig290_963	0.47	0.41	GGTGGGGGCTGTGAGCTGAACTTATTCTGAATTATAGTTCCCTACAGTCAGTCATTCTGGGGTCTCTGACTGCAAACTAGGCTTGAATGCAGTTGCTGTG[A/G]AATGCACTAATTAATTACATTTATTTTACATAATTGGCATTAGGGCTTTCTGAACAAAAATGCAAATTTATGGAAATTGGTTGAAATACTTATGCAAAAT
6	46068197	SCAFFOLD284472_542	0.45	0.43	GAGACTATCTATAGTAGTAATCTTCAGAGTATAAAAATGCTCATTTGAGAACTCTATCATTGTAAGAGAATAAAAGTTTGCTTTTTAACAACTTCATTCA[A/G]TAGGAAAGTCTTTTTCTTGAAAAACGTGGATGTCCTCCATAAACCATAATTGCATATATGTAGCATAAATATTGTTTTTTTATTGGATTTGTTGCAGAAC
6	46289361	BES10_Contig387_829	0.39	0.42	CTTTTCTTTTGGAGATATAGAGATGGGTTGGAATAGATTTTCAATTAAATTTCTTTTTCTTGTTTCAGTGGACATTATAAGCTTATCTATCAGACTAGAC[A/C]CCTCTGCCATGTATAGTTGGCTTTAAAAAGTAAGATTTTCAAGTTTTTTATTTATACAACATTAGAAACTTATAGTAAACAGTCACATAAAACAAGCTAT
6	46639539	BES10_Contig763_1215	0.23	0.29	TTTTTACCCGCTGAACCATCTGGGAAGTCTAGGGCAGGCTAGTGGACCCACTTAGGAGGACCTACTCTGTAATATGCTTTGAGGAAATGATGAGCTGTTC[A/C]TAGCTTAGGCAGAATTTGTGGACCCAAAAACTTCAATGCATTTGGAAATCATTCTCTCACTTAGCAATCCCCAGTTTGCCTCCTAATGACTGGTATGCCA

6	46683965	SCAFFOLD35310_9826	0.46	0.49	TTCTCTAGGGAACCCAATGTGCTTTGCAAACGTATTAAATAATTAACTCTCTCAAATCTGAGTGAAGTGGAAAGGCAGTGAAAATATTCCAGGAAGAGCC[A/G]AGCAGGTTGGTGAGTGATGACCAGATGTGTTGCTTTGAAAAGGCAAATCCACATGGCACCTGGCAGAGACCACAGGACACGAGGCTTCTTCTGGGGGCTG
6	47210709	BES7_Contig380_533	0.31	0.30	AGGGAGCACACAACCCGGAATGCAACCAAATCTTAAATGGAGAGGAAATGTTTTCTGAGTGGGTGACATTTGCGAAGACACTCAGTGTTGGGGAGAAGAC[A/C]AAAAAAAGGGATTTTTTGCATTTTTATAATCTTACAACTATCCATTTCTAAAGTATTGTCTTCCAGCTATTTTAGTTGGTCATCTAGTAAGAGCAGTCTG
6	47454399	AJ496635-013.SP6-114	0.18	0.17	TGAATCCTGACCTCCAATACCTGTAAATGTGACCTCATTTAAAATAGGAYCCTTGCAGGTGCAATGAAATYAAGATGATGTCTTACTGGAGTAGGGTGAG[A/C]TCAAAATCCAATATGACTGGTGTCCTTAAAAGAAGAGGAGAAGAGAGACTCACAAGGAGAATGTAGTATGACAGRAAGAGATTGGAGTGTTGCATGTATA
6	47454474	AJ496635-013.SP6-189	0.26	0.29	TGATGTCTTACTGGAGTAGGGTGAGMTCAAAATCCAATATGACTGGTGTCCTTAAAAGAAGAGGAGAAGAGAGACTCACAAGGAGAATGTAGTATGACAG[A/G]AAGAGATTGGAGTGTTGCATGTATAAGCCAGGGAACACCAGCAATTGTCAGCAACACCAGAAGCTAAGAGAAAGACATGGAACAGATTCTYTCCTGGACC
6	48041333	SCAFFOLD120627_3195	0.22	0.33	TTCAGTCTATTCAACTAATATGCATAATAAAGTGGCAATGACGGGGCCGAAACCCCCACCCAAACTCTCTGAAAGAGACACAATAAACTAACTGCCTTCC[A/G]TTGCTTTAAGAAGTAAAAACTGATTGCCTTGTTTGATCTGGTTATAGCAAGCATTTCAAGTCATAGCAATGATTATTTATTAAATATTAATGACTATTTT
6	48085009	BES8_Contig430_959	0.30	0.28	CTTAAAAGAGGCGAGGGATGTGCTGACATAGACATTAAGTAGGATTGAGCCGCCCAGCAGCTGCATCCTGATGAGCTCGTTCAGGATTTCCCCAGACTCC[A/G]CACTCCTGGAGCAGAAGAGAGAGAAGAGAAGGGAAAGTGAGAGGAAGGTCTGGGTGTCTGAGTCTGTGTTGGTGCTGCCCAGAGTGGCCCGTACCCTGCA
6	48085270	BES8_Contig430_698	0.31	0.28	TTAGCCAATTGGCGAAAGAACAAAGAATCAATTTAGTTTCAAATAGTTCCTTTAATGGTGGTGCTTTGCATTCACAGTAACTTGCCAAAGCGCTGTGTGC[A/G]TTTGTCCAGGATCCTCAGGGAGCCCAGAGTGGGCTTGTTTTTCTGCTGTGAGGCAGGGAGGGGTTGCAGTGGACCCCTGCTTTCTGTTCGATGAGAAGCT
6	48676372	SCAFFOLD15443_3220	0.46	0.37	TCTGAAAATTTATTCATCAACTAAAGTTTCTCTACAGAGAAAAGGCAAACAGAAGACATGGGGAGAGCCCTGTTCTGGGAAGGTCCTGTAGGGTCCTGAT[A/C]AGTTGCAATTAGATGATTAAAAGTGTGGTTGAAAACTTTCTTACCTGTGGGTTTTGGCCTTTGACTCTGCAAAGTATAGTGGTTCTTCTCTTCTCATTAA
6	51685676	SCAFFOLD322251_8603	0.18	0.28	CCCATAGGATAAAGGTGAAAATCTGTATTATGGTCTATAAAACTGTGGGTAGCCTGATCTCCAGCTATTTTCCTAGCATTGAGTTAGGCCTCTTCTTTAC[A/G]AAATTGCTTTGCATCATCTACAGTGGCTGTTCAAGGATTAAGCAATACCAGCTTTCTTCTCTCCTCAGATCATACGTGCTAGTCTCTGTTCCTACAAATC
6	52326162	BES7_Contig220_370	0.14	0.12	CAGAAGCATGGAAATAAATGCAAGTAATTTGAGTGCTCTGCACTGGCATTTAAATACAAGAGAAAAAAATGTTAATAGGATAAGTACCAAAAAGGATTAA[C/G]TGATGCTAAATAGCAAAGAGGATTAGTTATTATCCTGCCTTTACAGGCACCCAAATTTCATGTGGCCAAGTAGATAGGAAATAGAAAACCAATGCTACAA
6	52326214	BES7_Contig220_318	0.16	0.19	TAAAACTGATTTTAATATAAGCTTATTGCAAAGAGAAGAAAAAGGTCCCATTTTGTAGCATTGGTTTTCTATTTCCTATCTACTTGGCCACATGAAATTT[A/G]GGTGCCTGTAAAGGCAGGATAATAACTAATCCTCTTTGCTATTTAGCATCACTTAATCCTTTTTGGTACTTATCCTATTAACATTTTTTTCTCTTGTATT

6	52912651	SCAFFOLD166430_3864	0.43	0.43	AGAGAGTTTTGAGATACAAGAATATAGCCTAATGGATGAACGGGTAAGGGCATTTGAAGCAGACGTTCAATCTTTGATACAGCCAGCAGGAGCCTCGAGT[A/G]GCTAAGTTTTTGTTGTGCAGTTTTTGGAAGAACAGGAATGAACTGTATATTCATAAAAGTCAGTTGTTTTATGAGCAGTCCAAACAAGGTGAAACTTGAA
6	53887888	SCAFFOLD215078_3400	0.33	0.25	ACAAATTAGTTTAAATTTAAATAAAATATACCCTTTCATATTCCAAGAAAGTAAAATTTTCAAAACAGATTATCATCAAGTCTGTTCAATTACAAAAAGT[A/G]TTTTTTATTCTATGTACAATTTTGATGGAATAATGTCATTTAATGAATTCATTCTCAAATGCCTATCTCTGCCAAGGAACAATCTAAAAACAAAGTATAA
6	57337063	BES10_Contig751_552	0.09	0.22	TTCCCTGCAAACGCAACTTTGAGAAATGGCCCAGAGAGCCAACGTTTTGCGTTCTTGGAATACCCAAAGAGAATGTGCAGGGACATTCAGGCCTATGGTA[A/G]ATTGCTTCTCTCAGCATAGGCTGCAAAGCCATTGAAAACCTGGCTTTCTTGGGCATGCCACTGTCTCGGATTACCAAATAAAGCAGGTAAGGAGGAAGAT
6	57577953	SCAFFOLD140203_3711	0.30	0.24	ACTAATATGTGATTTAAGTAGAGACAACAGAAAATTCCTTTACCTTCATATACTTCCATAAAAGTAAAGTATATTTCTTTATTACCTTAAATGAATGTAA[A/G]TGTTGGATGCTTCTAAAATCTATTCTTTCTTGTGATGTCTATATTTTAAACTCTTGAGATAAAGTGAGCAAATTGGGAAAATTAAATCCCCTAAACACTT
6	57762925	SCAFFOLD260436_4579	0.09	0.20	GACTGTTTTCAAAGAACATACTGCTGTTTTTCAGAACGATGTATTGCTATTCTAGTACGGTTGTTTGTAATGTGATTCATTGCCTAATTGCAGATGAGGA[A/G]AAAATTGAAAAGACATCTCACTTGGAAGAGATACTTAATATTTTCCTTTCTGGGCTATTAAGTGTATATGAGGATATATGCTGTTTGTATGTGATTTTTA
6	57763160	SCAFFOLD260436_4814	0.40	0.40	TGCTGGCCATTACAGGTACTGTAACTGAACCATTTCAAAACTTGCTACACAGTGTGAGCAAGAATATTATCTAAGAAACCAGACTACTGGACTTCTATCA[A/G]TTAACCCTATTAGTCAACGCTCTGATGATGAAGATCTTAATCTCACATTGCATCACTTTTTAGTCTCCAGGGGCTAAAGAATAAAAAGATACAGATTCAG
6	58924721	SCAFFOLD317735_3027	0.36	0.30	TCGCCTTTTTATGTTAGTGGAGGTTAGTAACAAAGCAGATAAACATGGTTCAAAGAGAAAGATGAGCCAATAATAAGTAGGGTCCAAGAAGCAGAGAGTA[A/G]AAGTGCAAAACCTCAAAAATGATGAAATATTTTACTTCTATTTTCCAGTGTTTCTTTTTATCCATGCTTAAAGAAATGACATTTTCCAGAGCTTAAAACT
6	58924847	SCAFFOLD317735_2901	0.15	0.11	TATTATTGGCTCATCTTTCTCTTTGAACCATGTTTATCTGCTTTGTTACTAACCTCCACTAACATAAAAAGGCGAAGGTTGCAAAGCACCTGCCTTCACA[A/C]TGGCGTCTCTAGAAACGTTGTGCATCTGAAAATTGCAGCTGGAACTTCCCTGGCAGTCCAGTGGTTAGAACATCGTGCTTTCACTGCCGAGGGTATGAGT
6	59612217	SCAFFOLD110023_9176	0.02	0.03	TGTTCCAGTTGTGTGCGATATTTTAACATGGAGTAGTGGTAGAAAGAAAAATGATTTGGAGCCACACGAGGCTGAGGTAGAGTCCAGTGTGTTGGACTTC[C/G]GGAGTCTTGTGAGCTCTCTGGGTCTCAGTTTTCTTAAACTGTAAAATGGGGCTAACCAAGCCCTCTTCGTCAGGTGGTTCTAGTATAAAATGAAATGATA
6	59787612	SCAFFOLD130962_2571	0.31	0.39	CCCAGAGGAGGGCTATGTTGATGTCTGCCGCTTCCACCCAACCTCTTCACCAAGTTCCTTTCTTTATCAGAGCAAATAAAGAGCCATCTGAACAGTGATC[A/G]ATGGTCTTTCTCATTTCATTCTATCAGATACTTTTATTTAACCTACCGTGGATTTCCATGAACCAGTCCTAAAACAAAGAGGTATTTTGAAAGTATCTGT
6	62962733	SCAFFOLD93662_2444	0.29	0.27	GTGCAGGGGCTTTGGTTGCCAGCCTGTGTGGGCAGAGCTGGCCTCAGTAATGTGTTTGCTGCTTCTTAGGGATCGACTCTAGGGTTTAGCCATGGCGGTG[A/G]TGGTACCATGGGGTACAATGGCCATGTGGTAGCTGACATGATTCTATAGCAGTCCTGTATATAAACTGGTGGGAAACTTGGCCTTGTGGTCTTATATTTT

6	62962989	SCAFFOLD93662_2188	0.43	0.45	GGGATATAGAGGTAGACTAGCCTTTCTGGACTCTCTCCAGCCCTTGAAGACACATATATATCTCATGTGGAATTTCAAGAGCTATTTAGAAGCAACTTTT[C/G]TGGGGCTGGTGTGAGCATATTGTTGGCTTTGTTGGCATCATCCAGGGCAAAGAGATGAATGGTCTGGCAGAAACATGTCTGCTGAGTGAATGAATGACGG
6	68780721	SCAFFOLD26747_1262	0.25	0.30	TCACTTTGTTGGACGCCTTTTATCAGAGCCTGGTCTGCTTCTTCGTGCCTTACTATGTGAGTCCTTGTTCACTCAAAGGATTTGTTGTTAATGAATAAAT[A/G]GTGCCTCTTTGTTCTCTGTCACTGGTGTCAGCATTTAGAGTAGGTATACGAGGGTAGATTGCTCATGGAATTAAGTTATTAACTAAATATAATGCTTCTA
6	70665464	SCAFFOLD15013_21643	0.40	0.40	GATAATATATAGTCTCTTGATAACAAGAAGGGGATGGAAAAATGGAGCTGTTCCAAGTGTAGGTAACAATCTCAATTTAGACCAAAGAGGAAGGAAAGCC[A/G]TAGTAAAAGAAGCAATGGAACAAAGCAATGTTTGTCACCTGGCTCCTGGTTGTCTGCCCCACTGGATGCAAAGCCTCTTCAGAGCCTGGCCTCTGTTCAC
6	75645612	SCAFFOLD28042_518	0.47	0.47	GGGAGATAGAAGCCTACCCAGTACTCAGAGAAGCTGAGAAAAAAAAAAAAAAAAGGATGAAACACTGACAGCGGCAGATTTTTATTTGAAGTCATAAAATC[A/G]TAAGAATAAATAGGATGTCAGCAGAATCTATGATTTCTTTTTTTGGCCATAGACTTGTACTAATAAATAATGTCACAATAGTCTTTAGACATGAGAAAAA
6	78854889	SCAFFOLD113475_827	0.02	0.09	GTGAAGGAAGTGATATGTTGCACTTTTTCCTAGAAAAACTCAGGTTGGAATATGGTAGACATTAAAATACTGAGATCACATTTTATCAGCAATACCAGAT[A/C]CCTAAAAAATAGTTTCAAACAGTTAATGTACATGGTAATATTGGTAAATTGTTTCATTATACATAAATAATTGTACCTAAGATACGTGTCAAATATTTAA
6	78855277	SCAFFOLD113475_439	0.48	0.09	CATAATAACCTGTGTAGGAATTAAAATAGCGATTTACCCTTTGCATATTAAGACATAGAAATGTAATGACCAACTGTCAGATCAATCAGCACAGATTTAA[A/G]TATTCAATATTGAAATTCCAAACAGTTCTGGTTAAACTCTCAAATTCATACTTCAAAAGACTATTTAAAATAACACAATCAGCATAATATGGGGCAACAT
6	78855304	SCAFFOLD113475_412	0.48	0.09	TTTAACTGATTGATAAATCTGGTTATAATGTTGCCCCATATTATGCTGATTGTGTTATTTTAAATAGTCTTTTGAAGTATGAATTTGAGAGTTTAACCAG[A/G]ACTGTTTGGAATTTCAATATTGAATATTTAAATCTGTGCTGATTGATCTGACAGTTGGTCATTACATTTCTATGTCTTAATATGCAAAGGGTAAATCGCT
6	80373771	SCAFFOLD125132_7482	0.49	0.48	TTAAAATGCAGATTTCCTATCTCTCTCAAATCACAGCCCTAAGATGTTGATTCAGCGTCAAGTGAGACCTGGGGACAGTATTTTTAAAAAGTGATTCTGG[A/G]TTTGAAAAAATATATCACTGCTTTAGATTCCCTAAGGTATAAATAAAATTTGTATCTTAGCAGGAATGTTATTTTAATGCATTAAGAATCTGTGGAGCGA
6	84387497	SCAFFOLD130868_1137	0.05	0.10	TGTGTGCATATTAAGGGGCTTGTACTTTACAGGTGAGAGTAGCACTCAACTATTGTTGGTTGTTGACATCTTCCAGTAAGGTTGCAGCACCCTTATCTAG[A/G]CTGGCATGGAAATAGAGATGAGAGGTTTTAAAAATATATAATAGAAATGAATCAGTAGGCCTTTGTGATTAATTTGCCTTTTCTTCTCCAGGAGATCTTC
6	85658108	SCAFFOLD210144_9408	0.28	0.07	TACTGAGTGGTTATAAAATCTTCCTTTTTTGACTTGAAAAACAAATATGCCTGTATTGTCAGGCTGTGTGTACATTGATGCAGGCTTGCATAATACAGCT[C/G]CTAAGCTAAAATAACAAAGCAAAAACAAAACCCCAATTATTTTTTTCCATAGAGAGAAAAGACTAATTTTCATTTGTGTGATTTTGAAGGACTTAGTTAA
6	87989652	SCAFFOLD151156_3843	0.16	0.22	ATGGACATGAACTAAGGTAAGTCAGGGTATACATAAGGTATACATATACATTTTTTTTTCCTTATTTTTTTCATAATCCTTAATAGAAAATTATGTCCAA[A/G]TTATTTTTGAAAAAAATTTGAATCAATGGGTGAAATATACTGAGAATATTGCCTTATAATTGTTCTATAAAATTTATCTGTCATATATATACCAAGGAGT

6	89030769	BES3_Contig104_546	0.19	0.23	AATTTAATGTTACATGTCAACTTCTTGTCTTATAGAGCCTGGGTATATGTGAGTGTGTCAAAGCCTTTCCTGTTTTTCTGAAGGTGTTAAGTTTTGACCT[A/G]TCCTGCAAAGGACCATACCTATGCCACAGCGACAGTGTGAGCACACTTAATAGAAAAGCATCTAATCTTAAGCATGTGTGTGTGTTTGATGTGGGAGAAG
6	89037068	BES1_Contig554_1218	0.19	0.23	TAAAGGGTAAGCTTGTGACTGCTAATTTGCAAGGTACTGTATGAATATTTTAAAAGCTTTCAATTTTAAAATCATTTTAGAGTTACAGAAGTTTAGTGCT[A/G]AGTTCCCATATACTCAGCTCCCTGTAATGTTGACATTTTACATAATCCCAAGATGCACGTCAGAGGGCAAACTGAAGCAAACTGAGAATGCAGTTCTTCT
6	89274692	SCAFFOLD154819_5764	0.41	0.27	ACTCATTTATCAGACAGGATTTGAAGGTGTGGGTGGGTTTAGGACATTTGATTAACTGCAATATTTCCTGTGATTACATCATATATCAAATTACCAGACC[A/G]CAGACTACATGTCCTCTGGGAAAGCAAAGTGTTAAAGTGCTACAATCATATACTAGAGAAATTACAGCAGTACACATAAACATATGGGATTCAGCTACCT
6	89727906	SCAFFOLD300083_15251	0.06	0.04	TCTGTTCAACTTTTCAAATAATTATTAACTGCCTGTTATTTTCCTCCTTTGGAAGATGTGGGAAATACAAAGGTGAGTAAGTTCAAACTCCTGACCTCTG[A/G]TTGCCTTTCATTACTCTAGGCAAGAGGATAAAATAGATGTCTAAATTATGTAAAATAAGAAATTGTAAGAATTATCTATTCAAGTCTTAATGAAGGGCTA
6	91747096	SCAFFOLD125417_673	0.18	0.20	GTAAACGTTTTACTAAAATGCAGTTTTCTTGAACAGAGAAATGATAGCACATTTTGGTCAAAGGAGCCTTTTTGAAACTGCAAAAATATACAAGTATCGC[A/G]TCTTGTTTTTTAAGCATTCTTCTGATACAATATTTATGGCCATCCTTTCTGAAAGTGAGAAATTATAATAGAAAGCTCCAATGGAGAATTAAGGAGTCAG
6	91793236	AF061521-1028	0.43	0.44	CCTTTTTCCATTGCTTCTAAGAATTCCTCAGTAAAGATGCCAATGAAACTTCAAAACAAATCTACTTCAGTGCCTCATGTACTGTGTGGGTCTGGTGTAG[A/G]GTTGTCAGATAAAATACAGGATGTCCAGTTATATTTGAATATTAAGTAAAACAATGAATATTTTTTCAGTGTYGCATGTACATTGTTCCATGTAGGACAT
6	91793309	AF061521-1101	0.42	0.44	GAAAACAAAAAAGCAGGATTTCCAGATCGATTGGAATTTACAGTATACAATGAATAATTTCTAAAATATAAGCATGTCCTACATGGAACAATGTACATGC[A/G]ACACTGAAAAAATATTCATTGTTTTACTTAATATTCAAATATAACTGGACATCCTGTATTTTATCTGACAACYCTACACCAGACCCACACAGTACATGAG
6	91793435	AF061521-1227	0.42	0.44	ATGCTTATATTTTAGAAATTATTCATTGTATACTGTAAATTCCAATCGATCTGGAAATCCTGCTTTTTTGTTTTCGGYTTTTGTTAATCTTGCAACCCTC[A/G]CCTGCTGGCCAGGATTCCCGAGTGCCTGTTGAACTGCGCCTTGGTTTCTTTATCCCTAAACTGGAGAAAAAAAATCTCAGCCTCATC
6	92061352	AF061522-613	0.02	0.06	CACAACTGAGCGATTTCAAAAGATTAAGTAAATTTAACCAGAAATGGTAAAATGCTTCCAAACCATTAAAGAACAGATGTTGTTTACCTCYWTTCCTCAA[A/T]GGTTTCTACCTGAAAAATGGAGACCCAGGTCTCCATGGGCAACAGGGACCTCARAAACTTAGCAGTGAAGAGCAGGACTTTKCAGTGAAAGCTCCCTGTC
6	92061493	AF061522-754	0.25	0.08	AACAGGGACCTCARAAACTTAGCAGTGAAGAGCAGGACTTTKCAGTGAAAGCTCCCTGTCTAGGATGCCTGTGAAAGTTTTTTAATCCCTTTGAGCAGAA[A/T]CCTTTTCTGAGTGCGGAGCTGGGCCAGGGTCTGCATGCAGAAGTCCCTCCAGCCTTGGGGCCTGTACAAGAAGAGTAGCTTTCCTCAGCACTTATAACCC
6	94082193	BES10_Contig207_566	0.25	0.19	ATAGTGGCATTGAGAAAAAGATGTAGTAGAGGTATGTAAAAACACTGTTTTCCTTTATCACTATTCTTCAAAACAAGTTTTGAAAAATCTGTGCACATCA[A/C]AGGTAATTTAAAGGCCCTTTCCCTTACCATTAGAAATCTAGGTGTAAAGGTATGAAGAACAGTGAAACAAGAGTCAAAGACTCGGATCTGGCTCTATACT

6	94723804	SCAFFOLD306733_6306	0.30	0.44	CCCGCTTAGAATTCCTCATATATGGTGTAGTCCAAATGGTGACCTGGACCTTTTCAAGGACTTAGCAAGAACATTCTACATTTTCTCACCCAACGGTTGC[A/C]AATGTTTAAGAACGGTAATATTTTAAAAATTATCCAATTGAACATTTTTTAAATAAAGACCTTTTTTTCCTGATAACTGACAAACAAGATACAGAACTAT
6	95643454	SCAFFOLD206090_3335	0.17	0.17	TTTTGAAATGTATATCCCTGTTGTAAGTGAGATCATGGAGAATTTGACTTGAAACTTCTAGAACCATCTGGCTACTAAAGAGCTGCCCATTCTCCAATCC[A/G]ATCTAGGGACAGGCAAGTAGAAAGTTTCTTTGGTCAGTATACACTAAATCCCAAACTCTGGACAATAAAAGGAACAAAATCAGGTTTTAGAACTGTTGTG
6	96296531	SCAFFOLD145019_22761	0.31	0.35	TAAATGTTAGTGAGTAGGTGAATTCACAAATGCAGAATCCACAAATAAAAAGAATTGACTGTGTACCACAACTTGAAGGAGACACATCCAACCTCGTTAT[A/C]CTTAGGCACCTCTAAGTCACTCCCTGGTACACTGTGAGGTCTCCTGGTTTCTAAGGTCCAGCAGCACTGACCCTGTTTTATGATATATTATTGCCCAGCA
6	96636289	SCAFFOLD75609_20579	0.46	0.07	CCTCCAGCTCCCGGGTGGGCTCCCAGCCGGGACAGCAGGACTATGTGGAGTATGCTGGCCAGGTAGGTGGGCATGGGGGCCTCTGACTCCCGAACTGCCG[A/G]TGGGCTGCAGCCAGGCTGGGAGGTGGCTCTCGTCAACTGTGGTAGTTCCCCCTTGTTCACCCCACCTCCCTGCCCATCGCACCCTCCCTCGGGTCACTGC
6	96723956	SCAFFOLD220122_21854	0.22	0.29	TGTTCAGTAATTCTCTAGAATACCTCACACAATTCACTGCAAGTGTATTTCGGACTGCAGTTTTACTGTAAGACCTACACATAGAGCAAAGTCTGGGAGG[A/G]GCCTGGATTCAAAGCTTCCTCACCTCTCCCCATGGAGTCTGGGTGCCTCATCTTCCCTGCACATCTGGGGGTTCTCCAGCCGGGAAGCACCTCTGAGCCC
6	96737291	SCAFFOLD220122_34849	0.43	0.47	TGCTCCACAAGAGAAGCCACTGTAATGAGAAGCCCTCCCGCTGCAACCAGAGTGGCCCCAGCTCGCCACCACGAGAGAAAGCCAGTGCACGGCAGCGAAG[A/G]TCCAGCACAGCGTAGATAAAAACAAATAAATAAAAACGTAAATACCTCTTCAAGGTCATGCCTATCCCCACTGACACTCTACTGGTGTTGTGAAAGCACA
6	96803613	SCAFFOLD100769_598	0.01	0.03	CTACATTAATACTATGTGACTTTTGACACATCCCACAACTGCAGGAGACAGCAGAGTGTATCGACTGTAAGATAGAGAGAATGAGCAACTTTGAGTTCAT[A/T]GACAAGAGTATAGAACTGGGCTTCAGCATGGCTGAATAATAATCTTGAATCTTGTTTTGACCTCAGACTTTTCCATGTGATCCTGAGTAATTCACCTTAC
6	98767642	SCAFFOLD106936_12205	0.26	0.16	TGAACAATAAAAACAGTTCTGTCTGTCTGTAGTACAGAGTAATAGAAGGACAAAAAGAAAATAAAGGTAGATGAGCTGATCTGGGAGAGTCTTAAATGCC[A/G]GACTATGGAAATTGAAGGCAACGCAGAAGAAATGTGTAATCACTCTTCATTCCCTTGGGAATTTGAATGTTAGGCAGTCTGCTTCAGCTGTCAAAAAAAA
6	101204760	SCAFFOLD266863_5165	0.26	0.13	TATGATGTGGTGCTAAGTTCCGAAGCAGTACACCGTGGGAGGACTTCAAAATTATGCCAGGTCTGCAGGAGGAGACCACTGGCCTCCTGACATGCAGTTT[C/G]TCTTCTCTGGGAAGAATGATTAATAGTCATTAGCTTGCTCTGAAAGAAGGCAGTTAGGCACCTGCTGCCTGAGGGAAGGACAGAGCATTTTCTGTGGCTG
6	101738362	SCAFFOLD140036_39477	0.49	0.03	GTGTGTGTGTATATGATGGAAGTTCCAAGTTCTGTGGAGATAGCCTTTGGCTGAAAACCATCAATCAAAAAATAAGAGTTTTCTTATCTTATCTAGGGTA[A/C]AGCCATGACAAAACTCCACCCTAGCGCTTTTCAAAATAAGCTTGAACTCAGCACGTTTGAGAACAGTTTCCTGTTTGTCACTATTTTCTTAGGTCATTTT
6	103627410	BES7_Contig354_1224	0.34	0.43	AATTTCCCAACATTAAAGTAATAGAAGATGAAGAAAATGAACAGAGACTATGATTGAAATTAAATACCCAATTACCCTTCTTTCTTCTTCACAGGCACGC[A/G]TATCATTCTGTTTCTACTCCCCCCGTCTACCCTCCTAAAAACGTTGCTGACCTGAAACCACACGTGGCAACTTCATCTCTCTCAAGTTCTGATGCCGTCA

6	105866912	SCAFFOLD55166_20081	0.12	0.15	GATTACATGATATACTTTATGCTCATCACAGATGTGGCTGGAGAATTGGTATTGAGCTGGTAGCATCAGCAGAACAGAAAGTGAGATGTATTTTATATAT[A/G]TCAATAAAGGAATGACCTGTTTTTATCCTACAGACAGTGCAAATTGGAAGTCAAATACCCTTTGTATTCCAAAATAGGACCTCAAACATCTTGTAATTTT
6	107235779	BES11_Contig481_1108	0.20	0.27	GTCTGTCAGGTAGGCTGTGTGCAGACACCACGTGAGGTCCCTGCATCTCAGTGGTGAGCGAGGTGGGCACAGTGCCTGCCCTTGAGGAGCGTCCTGGCAC[A/G]TCAGCAACATGCCTTCATCAGAGATGCTCGGAGAGCAGGGGCTGAGATGGGAGGTCCCCACAAATTACGGAGCTGCTAGAGGGTTTGCGGTCTTATCGCC
6	108219700	SCAFFOLD111628_3947	0.39	0.40	GGACCTTCTGATGGGCTCTAATGGACGGTGGTCCTATCCAAGGTCACACCATGGTCACTGGGGGACTCTGGTTGGAGCTTGACATTTACCCACCTCCTCC[A/G]TAAGCTCTCTTTCTGCTCCATTAGGCATGTAGCTTCCTGTGGCTGTTGCAGCAAATTCTCACAAAATTGGTGGCTTCAAAACATGTAAATCTGTGACCTC
6	108554388	SCAFFOLD40472_6872	0.16	0.25	GGTTCTTTGCCACTGAGCCACCAGGGAAACCCCTACATGAAGTTTTAAGGGAGGCAAAACTCTATTTAGTGAAAAGAGTTCAAATAGTGGTTATCTTGTC[A/G]ATGGTGCTGAGTCGAAGCAGAAGGCTACCAGGATATGGCAAGTTTCCAGTCTTTTTGTAGGTGGTGGTCACCTGGATATATACACTGGCAAAAACTCATC
6	111541598	SCAFFOLD145412_11483	0.33	0.43	TATCTCTTCTTGCTATTCTTTGGAAGTCTGCATTCAGATGTTACTGAATATATATAAGGCAAGTGAATTACTGTATGCAGTAAATATCAAAAAGCAAAGC[A/G]AAACTAACTTATCTAAGGCTTCCCTGGTGGCCCAGTAGTAAAGAATTTACTTGCCAATGTAGGAGAAATGAGTTTGATCCATTATCTGGGAAGATCCCAC
6	113412937	SCAFFOLD226951_9486	0.21	0.28	GAACTGCAGCTCCTGCTATGTCGGGGGCTCCCTGGATACCCTCTTCCTCCTCCCTTTCCAGATTCAGATATTTGAGCTGAAAGAGAGAGACCATGAAGGC[A/G]TTCTAACCCAACTCCTTCTCACTGATAAAAAACCAAGGCTCTAGGAATTTAAGTAATTGGCCCAGATCACATAGCTTATTCACGCCAGGTCTGAGATGGA
6	113779252	SCAFFOLD280465_4204	0.35	0.35	ACTCTCTCGTCCTTAAGATTTAAGGCCTGTAGAGTTCCATGGTATTAACTCACAGAGCTAACCAATTTTTATCGAGCACCTACTCTGTGTCATCAGTGTC[A/G]TAGGCACATAGAAAGAAATCAGTGAGAACAGAGTTTGTTTGTTTGTTTGTTTAAATCTCTGCCTCTGTGAAGCTTACATCCTTGTTTACTCTAACAGAAT
6	114089011	SCAFFOLD16736_4538	0.18	0.17	TCCAAGACATCTTCAATATATTTTATTCATCCATTCTCTCATTACTAGTGCGGAAATGAATTACAAAGGAAAAATTGTTAGACTAGTGTAGAGCCTCGGA[A/T]GCGTCCGGTTTCACACCATTATTTTAGAACTGGATTCTTAATGGTGAGAGAGGAATCCCGTCTGTAAATACCCTGTAGAAACTGAGTCCTTGAGATCACT
6	116092430	SCAFFOLD115102_212	0.36	0.27	TCGGTGGTCATTCATGGGACCGACATTCATGTGGCCACAAGGAGAGCTGGGGATTGAATGTGATTTTGTGTCAGAGACCAGGGCTCAAACCACAGCCCCT[A/C]CAGCTCCCAGGTACAAGACACGGGTTAAATTATTTAGCCTGTCTGAGACTCATTGTCTTCATTAAATTTCAGCATGCTTCTCACTGGGACAAAGGGAGAG
6	117063764	SCAFFOLD142559_1766	0.11	0.13	GTTACAACAGAAAATGTTCATCCGCTCACATTCCTCTGTCTGGGAGAAACGCAAGGCTACTCCCTACAGTTTCCTATGTTCTCCTTGTAACTAATCCCAT[A/T]CTCTCTCACCCAAGACAGCGAATTCCAAGTGTCAGTGAGAGGGGTGTTTCCCACAAATAAAGTTGAACCCACTCTAATTCATTATTTTAAAAGAGATTCA
6	117063835	SCAFFOLD142559_1695	0.09	0.10	CAGAACCACCAACTACATGAATTCTAGAAAGCAGTGCTTCTTGCTTTTCTTCTGGTCAGTTATAATAGAGCGTTACAACAGAAAATGTTCATCCGCTCAC[A/G]TTCCTCTGTCTGGGAGAAACGCAAGGCTACTCCCTACAGTTTCCTATGTTCTCCTTGTAACTAATCCCATACTCTCTCACCCAAGACAGCGAATTCCAAG

6	117300223	SCAFFOLD310333_4540	0.36	0.48	CTGCAGACTAAAGCATCAAGGAGCCGGAATAAAGTACAGGGGAGTGTGCAATGGTTGCATAAGGGGGAGGCTTAACCAGAGGAAGTGAAGACCCTCCGCC[A/G]TCCTCCCACCTTGCAGTGGGTCCTGATGGAGGGTCTGGCAGATCAGCGCAGATGTGGGGGTGGGCATCTGGTAGTTGAGGGAGCTTTGAATAAAAGGCTC
6	120079664	SCAFFOLD230303_18208	0.50	0.50	CCCCCTTCAGGGCAAGGACCTCAGAGAAACATGCCGCCAGCCTTGGGGTCCCGGTCCCCTGATGGTATGTATATAATAACAGCCCTTGTCCACAGGACCG[A/G]CACAGTGGCTGGGCTCCAGGCGGTCAGATACTGTGGTTGGCAGCACCCTGAGTGTGGGCTGTCACTGGTGGCTTGCTTCTGATTAAAGTTTACGTATTAA
6	122523572	SCAFFOLD134696_1091	0.36	0.33	AAGGGAAGCAGAGAGGAAGCAATAAAGGAACAAGGGGATTAATTAGTGGTTCCAGAGGGACCTTGGGCTTCTCGTTACCACGGAGACAGCCAGGCTGCAG[A/G]AGGCAGGTAGGACCAAGGTTAAGGAGGGTGGGGCTGAGCAAGAAGTCCACGCACCCCCCACCCCCCAATTCCCACCTGTTCCTGCTAATACCAGTGAGAA
7	2190301	SCAFFOLD140093_5035	0.34	0.43	AAAGGATAAGGCTCAATTCTAATTTTTTCTCCTTTCTTCACTGGCAACATATTAAACTGTTATTCTTGGTGTACAGCTTTCAGGACAATGTGAAAACCTC[A/C]CATTTAATTCTTTGTCCTTTCCTCCATTTTATGGGAGGGGTTTCAAATTTATCTCCCAAGTTAAAAAAATAAAGGACTATTCTTCCACTTTATCATGTAG
7	4367747	SCAFFOLD86698_2043	0.05	0.16	TTCTTGAGGCTCACTGACACAAGAGGGCCTTTCGCTCCACCAACCATGGCAACCAGGGGCTGGGACTCTCCCCCGGCTCTGCAGAAGACAGGAAAGGCGC[A/G]GTGAGCTCAGGGCGTGCGGAGGGCCAGCCTGGTGTCTGCGCGCTCCCCAGCCATCCTGTGGGCTCTGTGCACTTGTGTCTGGTCTCGTCACGTGAGCTGG
7	4808855	SCAFFOLD120796_3553	0.11	0.13	TTGTCCACAACTTGTGAATAGCATGCCTTTGGAGAGGTTTCCTGACAGGGTCTCAGTTTTTCCTTCTGTGAACTGGGAGGAGTTAGCCCATTCTGGCTCA[A/C]TCTGGCTCAAAATGACCCCTCTCCCTCTGAACCGCATGCTGTGTGACCCTGGGAAAAGCAAACCCATCTCTGGGTGTTTTTCAGCCTGAAAAGGAGAGGC
7	4820181	SCAFFOLD246126_13408	0.12	0.13	GTTTATGACTGTTCAGAACAAAAAGAAGAGGCAGGGGTCGGGGAGGACCCCCTGCCTGTTGTCTCGTGGTCCTCCTCTTTTTTTGTTAGCCAGCAGTTCT[A/G]TGGTTGGAAATGTTATTCTGTGCTTCTGGTTTTGTGAAGCCGCAGCCAACTGGGTTCCTGGAAGATTCTGTGTTTAATGCATCTGTAAAGCCCTCACTCT
7	7078535	SCAFFOLD60158_10651	0.49	0.48	CTCTCCCAGCCCTGTTGGCTGCTGTTTGGGCCCGAGGCAGTTCTCCAGCCGGGAGAGGAGCCCCCTCCTGCCTGACGCTTGGGTTGGGGGCTGCTGGTGG[C/G]GGGGGGTGGCTGCTCTTCAGTAGGAAAGGAAGCGCAGGTGTGGTCTGTTGTTTTCAGTTACTGTCACGGTCCATGTTTCAGAAGTGTGGGTTCCTCATAC
7	7214961	SCAFFOLD130445_7735	0.32	0.35	GGCAGCATTTAGCCTGGCACAGACTATCGAAGGCAAGTGTGCGGTTACGGTCGTGTTCAAAAATACGGCTTCGACGCAGCAAGCTTGGGGCTGGGCTGCA[A/C]GCCCTGTAATTCTCTTTCCAAATAAAAGGCAGAACTATTCCTGGCTCAGCTTGGGGACTTCTGCGGCTGCTGGGCTCCAAGGAGACTGGCCTGGAATGTC
7	7215131	SCAFFOLD130445_7565	0.40	0.46	TTCGATAGTCTGTGCCAGGCTAAATGCTGCCAGCTGCCTAGACGCTCGGCTTGAGGCTCTAAACACAGGTTCTTTTTTATTCCTGTTTTAAGGTCTATCC[A/G]ACTATTTCTGTGCAAAAACAGATTTAAAATCTCGATGGCTCAGAAGCCGCTGGCAGCTGCCAAGGCCGCCCTGTTCTCTCAACTCCCCTCAAGGGGAATC
7	8443679	SCAFFOLD109850_10815	0.50	0.35	ACAGGCTCACAAAGAGTTGGACGTACTGAGCACACATGCACAGGGACGGTGTATTCAGTAAATAACTTCAATTTAATGAGATTTCAATGTGCCTTTATAA[A/C]TAACAACTATTTTGAGTGATTTTTTTTAAAGGCATTTTCAAGTTTAAACTGCAGTGAACCACTAATCTGCTTTCTGTTTCTATAGATTTGCCTGTGCCT

7	9943356	SCAFFOLD241555_8746	0.14	0.13	CACGACTCCCGATCCACGGAGACACAGACCTGCCCCACTTGCTGCTGACCTGACAGCAGATTTACACGCTTGCCCTCCCAGAGCTGGCAACCCGATGGGG[A/G]AAAAGATTCCAGCACGCTGAGAGTTTAGTTTCATGGGGAGACAGAGCCTTGCTCAGGTAGCTCAAGATCAGGCCCTCTCCAGGGAGCTCCCTCCCATTCA
7	10764886	SCAFFOLD358501_284	0.21	0.19	CTGCCCGTCCCCACCTGAGACCCCATCAATGGCAGGGCTCCATGGGGTCCAAGTCCCACCTCCTCACCCCCATCCCTGCGGACCCAACCCTGGCCCAGAA[C/G]AAGGAAGGACTCACTCTGTGATACTCTCACCAGCTCCGTCACATTCCAGACCCCGGAAGTCACAAAACAGTCCTGGAAACTTAAATGCCCTGAGGAGGAA
7	12292285	BES10_Contig724_1957	0.00	0.03	AGTACGTTCAGCAATATTTCTCAGTGACAAATTCTGTTTAGAACTGCTTCCTTCTTGGTTCATGTGTTATTCACCTCTGTTGATACACCCATACTTTATA[A/C]AACTTCACTGAAAAGTGAAAGGGAGAGAGGACATTAGAGGTAATACCTCCAGGATCTCAGTAAAATGCAACCTTCTTTTTTACATATGTTAGTGTATATG
7	13611810	SCAFFOLD295072_19940	0.22	0.30	CTGTTAAAATGTGCTATGAAGTTTTAGATTGCTCTGATAAAAGGCTAGCGGTGTAGCTTTGACCACCTTTACTCGAGCTCACATCTCCCATTGGGTTTCC[A/G]TTCTGGGAGGTTGAAAAAACAGTTGCACAAAAAGTGAGTAGTACTTTTCCGAGCGGATTGCACCGCCCTAATCCTTGGATGTTCTTATCAGTGTTAAAGG
7	13612147	SCAFFOLD295072_19603	0.20	0.20	GAGATGCGCAAGGGGCCCTGGGCGGACTCGGAATCGTGAAAGCATGAGCACTGTCCGAGGTTCCAGGATTTGGTCTGAAAATTACTCCACTGTTAGAAAA[A/C]ACTTTATCCAAAACTCTGCCCCAGCTCTCTGCATCTCGATCACGGCCACAGGTAACAGCAGCCAGTGGAAGAAGACAAACGAAGAGGCCCTGGTTCTACT
7	13944196	SCAFFOLD11216_1018	0.07	0.11	CCTTTCCGAATTAACTTTTGCCTATGGTATGGGGTCAAGGTCCAGATCCATGCTTTTGCACGTGGCCTCTGGTCATCCCAGCATGGTTGTTGGAAAGACT[A/G]TTCTTTCCCCTTGGATAACATCCTTTTTTTTCAGTAGTTCATAGTCGGTAAAATTTGTGTTAATAAAAAAGAGATTTACCCAAAACAGCACTCCTCGGTC
7	14679285	SCAFFOLD128762_1361	0.29	0.15	GCCTTCAAAGTTGGCCTATCCTGAGCCAAGGGATCTCCTAAACCTCCCACCACTCCAATACTGACTCATTTCAGACCCATGGAAGAGCTTGACTTCCTCT[A/C]TCCCCCAGCCTTGACCCTGAGCCACTCTGGCCATCCCCTAAGACAAATTCGTCTTCAAAGAACCAGGACGCAGTGGAAGAAATTCCTCCAGCAGCTGCCG
7	14850002	BES8_Contig520_815	0.10	0.20	AGGCTGGTTTGGTTCGGGTTTGGGATTGGCTGGTGGAAGGGAAGCGGACGGAAGGAACCGATGCTTTCCACCTCCGCTAACACTGCCCTTCCTTCAGGGA[A/C]GCTTCGGAGGAGCATTCGCTGCTGGTGAATTGACTCGGACCCGGACTGCTACTGGACATTAGCCCCCTCACCACCACCACCCCCGGAGTGCGGAGACCCA
7	15314370	SCAFFOLD108415_899	0.06	0.06	TTCCTAGATCAGGGGTCGAACCTGTGTCCCCTGCAGTGGCAGGCAGATTCTTATCTACTGCCCTACGAGGGAAGTCCAGGTAGGGAGAAGTCTAGAAGGA[A/G]TGGCTGCCTGATGCACCAGCCTTCCATGGGGCTTAGCCATGGAGGTATTGGTCGTGCGTGTCCCCAATGTGGGACCGTCCCTCCCCACCTTCCACATGTA
7	15429321	SCAFFOLD160001_65120	0.05	0.09	AGATTTCCAAAATCCTTCTGTGGGGCCCCCAGGTGGTCACTGACCCAGTGTACTCTGAAAATGGGGGGTGTTCGCCAACAAGGCATTTTCTTTGTGAGGC[A/G]GGAGAACCATGTGTCTATGTACACCTCCAAAACAGGACAAGATGAGTTAGGAAGGTTTTATTAGGTAAGAAGCCTGGAGGAATGGGTAGACTAGGGGCTG
7	15613496	SCAFFOLD181588_9953	0.11	0.18	CAGGATGACTTTCTTCCCCCTCACTTGCTTCCTCCTACCTTGGCATCCTCCTGGCCCATCCCCCTGAGATTCTCTCAGTCACCATGTCTAGTGCCCTGCT[A/C]TGACCTTGGTCCTCAGCTCCCCCAGCCTTCCCACCACCTCTGCTGTGGTCTCCATCCTTAGAGAGCCAGTTCTGGCACATTCCAAGCTCCCACTTTCCCT

7	15692315	SCAFFOLD41765_2717	0.29	0.36	TTATGGTCAGAATTCCTAGGAGTGTTGTTATTTGACTGTTTATGCCTCCATGCTTTTTCAAACATGGCCAAGAGCACAGAATAAAAGACTAACTTCAAAC[A/C]ATAACATTAGAGGAGAAAAACTGCATTCCCAACTCAAGAAACCACTAGAGTTCATCTCAAAACCCAGTGCTGAGTTTCCTGGTGGCCAAAGCAAGGAGGC
7	17671182	SCAFFOLD285555_7465	0.40	0.40	TTATCCAGCCCTGCAGCTCCCACCCACCTCCTCTCTACCTACCATCTCCCTCACCATGGAAATGAGGAGTTAGAGAGGACATCCCTGTCCCCTCTGCTGC[A/C]CCCAGACCCTTGAGGGGCTGGCCTGGTCTCCCCTCCTCCCTGCACCTGATGCCCTGGCCAGACTGGACACCATTGCACCTCCAGCTTGCTCCCACTGGCC
7	18953162	BES9_Contig395_449	0.48	0.42	TGTCGTCCCAGGGTGGGGACTGTGACGGCTTCTCGACTTTCGACGTCCCCATCTTCACTGAAGAGTTCTTGGACCAAAACAAAGGTGAGCGCTGACCAGC[A/G]GTTCTTGAAGCAAGCGTTGAACCAAGAGGGCATCGAGGCGGGAGGCAGTGGGTGCCTTCCTCTCTTGCCCTCGCAGCCCAGACTGCTGAGATGTGGGGCT
7	19328611	1179-290-R	0.17	0.26	GGGGGGTGTGAGTACCAGCTCTTGGACCTCACCAAGTAGTGAGTGGGGCTAGGGCAGTGCCTGGGGCCAGCGGAGGGGAGTGGACAAGGTGTTGGGTGAC[A/G]CCATCTGCACGCTGCTCTTCAGCTGGGACGTGGACAAGTYACCCAGGGAGGCGGGCATCTGCGGGGSTTTCTGTTGTCTCTGCCCCTTTTCTGTGTAGCT
7	19778747	SCAFFOLD10954_11613	0.42	0.39	GTAAAAGTTGCGTACTAGGCTAACTTTGTTGGAATTACGAAAAAATGGGACTTATGAATGTGCTTTTGGAAGGGAACTCACTCATAAGTGGGGACTTACT[A/G]TATTTCCCCATACCATCACAAATTCTAACATTTGATTTTGGGATGATTTTAGATGGAACGAAAAGTGACAAAAACAGCATGGAGAACTTGCACCTGCCCT
7	20475876	AF440369-283	0.23	0.19	AAGAGCCACTCTCTGCCCCCTCATAATCTGGTCTCCCCCACCACCCAGACCTGTCTCCGCCGGGCCGCTCAGTTCCCCTCCTCCTACAGACTCACTGAAC[A/G]TGCTGCCTGACTCCCTGGTGTTTCCCCCCACCCCCCACAGTACTGTGCAGCCCTGGACTCCCTGATCAGCATCTCCAACTGCAGTGTCATCCAAAGGACC
7	20792064	SCAFFOLD110047_28436	0.42	0.13	TGATCAGTTTAATCTATGGATACAGTCCAAAAAAAACAATTACATGGGAGCAAAGCCTTCACCCTGTCAAGCTGTCCTTTGACTGAGTTCTAGTGGAGAC[A/G]TGTGTGGGAGATGGGTGGGGCCATACCTGCATTTTAGAAGTTAGGATTTGCATTGCATCCACTTGGACTGGTCTTCAAACTAACTAGAGGCCAACCAGTC
7	21228652	BES9_Contig501_1378	0.45	0.09	TTTGCTATTATCAAATGTGTCCTGGATTTAAGTGTATCCAGGATTTAATTCATTTTTATATTTTAAAAGAACACACGATTGTATTCAAATTTTCCATCAC[A/G]ATGGCCCTTAAACAGTATTTCCATGTCTTTTACCCATACACAGTGAGTACGCTCTTAAAATGGTATATTCTGCAGTCTAAATTATGCAGATTTTGCTTTC
7	23447386	SCAFFOLD125009_37271	0.26	0.22	CCAGCTCTTGAAGTCATCTGATTTTTATCATGTGGGAAAACACCAATACCATTGCCATCGTTTTCCCATTTGGATCTAAGTGCACAGTGTATAATTGAAG[A/G]AGACAGAAACAGTATTAATCTGTTAAATCACTCGGGATGCCAGTCTCCTAGATACTATATATAAGAAATCATTTAGGGATTTCTTTAGGTGGATTATATG
7	23594896	SCAFFOLD166571_1352	0.18	0.16	TTTAATGCTGGGTGTGTTGTTAAATGAGACAATCTTTAAAGTTATCTCCAAAGTCCAAAATATAGATTTCAGGACCTCAGCGCAACAGAATAAATGCCCA[A/C]TTCTCTTCAAAATGTAACTGAGGTTACATATGATTTGGGGTAGAAGCAATGCTTCCCACCTGCACATATCCTCAGATTATCGGCAAGTTGAATTAAAAAA
7	24604791	SCAFFOLD30306_6015	0.09	0.05	CTAGTTTTGAACTGTCTTAGCAGAACTCAAGTGGGACCCTGTAACTTATGAATGTTTTGAAGATAAAAACATCCCCTCATTTACAGACCTTCATGGTTCA[A/G]TGATCTCCATAGTCAGGAACTTCGTTCAGCTTTCATATTCAGAATGTGTAAATGCAGGATGTTACCCCATTATTTCATTGCTTCCCCCAACGTTAGTATT

7	34247503	SCAFFOLD220833_3676	0.06	0.13	TCATCATGTTTTTTATACACTGACTACTTAAAATACTCACTAATGGGAAGCTCTTAAAGAATTCCATAAAACTAGTAAAATTATCTACTGTTTACAGTAA[A/G]TGTTCACACCCGAAGACTGGTGGGATATAGATGGGAAAATATTCATCCTTTTAGTCCCCTATCTGGTTAAGAGTGAAATCACAGTCTTACTAAACATTCC
7	34989404	SCAFFOLD175177_23964	0.21	0.33	GTAATTATTCAGAAAGTGTCTAATGACAGAATCATAGGTTTGCATAATCTAGAGATAAATAAAAGGTTCCACAGATAAACCTATTCACCTCCCATCAGGA[A/C]AATATTCAAATTTGTTCTGCCAATTTAAAATGTATACAGGCATCCAGGTTTGCTTCATAAACTCTTCCATCCAATTCAACCCATGAAGCTTCCAGAAGAA
7	35864297	SCAFFOLD65183_10518	0.23	0.38	TCCTCTTTGATTATTTACAGTTAGTCCCCCATAGTGCTGAAAGCTGTACAGGAAGAAGAAAATATTGATGTTCCTGGAAAGTTTTTCATGGCTCAGTTCA[A/G]GATCTTGCAAAACAAAATCTGGGGGCTAGATACACTTTAATGGGAAAGCTTAGCATTCAACTATGAGAGATGGTCGAGACTGATTGTCTGGAATGTTAAT
7	35965286	SCAFFOLD131133_3718	0.47	0.36	AAAAACAGAAGAGCTGATTTGGAATAATATCCAGGGATGGAATCATGACTCTTTAATTACTGCCAAAAATATTCTTGCCTGTAGCTATGATAATGATGTA[A/G]TTTTTGAGTATTAATAAGCTGATGAGATGGTATGATGCTTCAAGATGTAGCTGTTGTTTTTTTGGGGGTGTCATTACATGAAGACACAGGAGGACCATAT
7	35965364	SCAFFOLD131133_3796	0.44	0.30	TGATGGGCTGCCGTCTACAGGGTCACACAGAGTCGAACATGACTGAAGTGACTTAGCAGCAAGGGAGTCCCTGAGAAGAAAAACAGAAGAGCTGATTTGG[A/G]ATAATATCCAGGGATGGAATCATGACTCTTTAATTACTGCCAAAAATATTCTTGCCTGTAGCTATGATAATGATGTAGTTTTTGAGTATTAATAAGCTGA
7	37439984	SCAFFOLD150535_3379	0.21	0.41	CCCAGGATCTCAGGACTGAGACCTTCAAGTACAGGCTCGAGGGAAGCTTTCAGAATGAAAAGTTCTGCTGAAGAGTCAGCATCACAGGGAGGGGCCTGCC[A/G]AGCCTCACAGGCTTGTACCCACCAGCGTCCAAGTTCTCATGATTGACAGCCACCAGGACAGCCTGTGGTGGAGGTTCCTCATGTGCTGCTCACAAGAAGT
7	37440256	SCAFFOLD150535_3651	0.42	0.48	CAAGTAGCCAGGGAAACTGACCCATCAGCACATAAGGCTTTGGTGTGTCTAGGCTCAGAAGGGGAGGTAGACTCTGGGGCAGGGCTTCCCATCTGAACCC[A/G]GGAGGGCTCCAGGCATGCTTGGTAAATGCAGGTGGAATGAATGAAACTCTGTGCACGTGTCTAGGTCAAGAGGGCACTGCAAAGCCAGTCCCACGTGGCC
7	37499993	BES2_Contig338_770	0.31	0.25	GCTTGCTAGAGATGGAAATTCCTACCCTGGAGCCAGGCGGGGGCACTTATGAGAAGGGACTCTCAATTTTAAATGAGCTGGTGATTGGGTGGGATGGAAG[A/G]GGCTTTATTTCGTAGCGACTGAGAAAAAGCTAGGCTTCTCTCAAGTTGGCTTCAGCAAACAAGGGAATATGTTGGCTCATGTGGCAGTCTATCTTCAGGA
7	37617743	SCAFFOLD2403_705	0.39	0.46	TAGTGAGGAGGAGAGGCTGTTGTCAGCTTCACTGGGCCTGTTCTCGGAGCACAGGTTCCCAAACAATGGCTTTCTGGCCCCATCCCCAGGGCTGGAGGAG[A/G]GAAATGAAGGGGCCCACAGAAGGAGCACTTCTTGGGTCTGCCTCTCCTGTCAGCCTTCCACATGGGCCCCGAGCGTCACCCACAGCACTGGTCATGTCAC
7	38095665	SCAFFOLD147057_270	0.43	0.42	GCACAAACCAGCTAGTAAGATATCTGGGTCCCAGGTGGCCCAGCCTTGGGCGGGGAGAGCTGTGAAGGATAAGAGCTCCCAGCAGCAGGAGGGAGCTCTT[A/C]AAGAGTTGTACTGTGTGCTTAGTCGTGTCCAACTCTGGGACCCCATGGACTGTAGCCCGCCAGGCTCCTCTGTCCATGAGGATTCTCCAAGCAAGAATAC
7	39757182	SCAFFOLD136640_2607	0.19	0.26	GGGGTCTGGTTCCTGGAATCTGCTCTGCCCTGGACTACTTACCATGGCCTACATCCTGGCATCTGGTACCCATCCTGGGAGTCAGGGACCTGCTTTTTAG[A/C]ACAAGTTCATGACTGTGGCCTGCCTCAGTTGACTTTCAGGTACTGCTTCCCATGTACCCAGTGAGGGGCCTGTCACCCTTCCCTCCCTGAAAATCCTACC

7	46591403	SCAFFOLD36422_8695	0.09	0.08	TGCAGTGCATTCATTGAGTTTGTAAGACTGTGTGGGAGAAAGCTAACCCACCTCTCTATCATGGAGGAAGTTTTGATACCTGATGATGTTTATAGCCTAG[A/G]TGAAATTCACACTGAAGTCTCCAAATACCTGGGAAGAATATGGTTTCCTGATGTCATGCCTCTCTGGTAATTGACTACTGAACCAAAGATTCTTAACTTT
7	47784391	BES4_Contig295_1011	0.39	0.35	GGGTGTCTCAGGCTATCTTTGGATGTACTTTTGTTTTAAATAGAATGTTCTCAAGCTAATGTCCTGTCTTTAATGTTCCTCACAGAGGGGATGGGCAGTC[A/G]GCAAGGCCTTCCACGCACAGAAGGACAGGAGAGATGAAAGTTAATTGGTTTGGGGACCTTGGTACCTTTCCAAGGCACACACTTAAGTGATGTTTTTCAC
7	47784483	BES4_Contig295_919	0.39	0.35	CTTTATGGATGTCAGGCACCAGGCTAGGACCCATCTGGGGCACTGACCATGAGAGTTCCGGGGCCAGAAAATGGACTTGAGGTGATTTAGATGGGTGTCT[C/G]AGGCTATCTTTGGATGTACTTTTGTTTTAAATAGAATGTTCTCAAGCTAATGTCCTGTCTTTAATGTTCCTCACAGAGGGGATGGGCAGTCGGCAAGGCC
7	47784916	BES4_Contig295_486	0.21	0.15	ATTTCCATCAGGGCTCAGGGTGTGCTGAGTGGCACATGGGTGTGAGTTGTCATTTTAGCCCGTCCACCCTTAAACAAGAGGCAGCAGGCAAGCTTAAGCC[A/C]CCTGAGACTCATGGCATGTGTGTGTCGCCTGCCTGTTCTTGGCCACTTTCCAGGGTCTGGAGAGAGCTGTGCCCTGTGTCCAGGGGAGCAAGGTCAGTGT
7	47899280	SCAFFOLD29492_996	0.17	0.09	GAGATATAAAATAACTCCTGATTATATGGTACCTAAGTCTCAGTGTGCTCTTCCCGAGATGCTACCTGTGTTGAAAACATGAGCAGGGGCTTGAGAGGGA[C/G]CCAAGGCGAACCTCAGAGGCTCAGTTGTTAACTTGGATGAGGTACCTCCTAGGGCATATTTGGATAACAGTTTCTAAAAAGTCACCCTATGAAAGATGAT
7	47899801	SCAFFOLD29492_1517	0.42	0.50	GATCCTGTCCAAATCTGCTATTATAATTTTATACAGGAATTCAACAGGAAAATGCCATAATTTAATGCACTGATGGCGCTGCTGATTCAATTTTCTGCTC[A/G]CATAAATATCTGCTAAATCCCTTACTATGAATGAACACGGCTGGCCTCAGGTACAAGGCCTCCAGTTCTAATCACTGCAGCCACCAGAACTAATGATTCG
7	48199692	SCAFFOLD290169_15087	0.42	0.40	GCAAATTAACCAAGCTTAACATTTTGATAAAGGCCTCTTATTTTCAAATGAACCAAGGAATCCAATACTCCATTGCAAGTTTAACACAAAAGAAAATTTT[A/T]AAAGCCTGAGTTTTTAAAGTAGGTGACAGCAAGAGACATTAACCACCTGTAAGATGGAATACAGAAGCAAAGTGCATAAATCTATTAGTTCAAAACAATA
7	48199731	SCAFFOLD290169_15048	0.16	0.27	TAAATTATTGAAACACCTTAATCCATAAGCTCACATAAATATTGTTTTGAACTAATAGATTTATGCACTTTGCTTCTGTATTCCATCTTACAGGTGGTTA[A/T]TGTCTCTTGCTGTCACCTACTTTAAAAACTCAGGCTTTAAAAATTTTCTTTTGTGTTAAACTTGCAATGGAGTATTGGATTCCTTGGTTCATTTGAAAAT
7	52738784	BES11_Contig367_1030	0.29	0.28	AGCCTCCTTGAAGAAGCTGAGCGGCCAGGTTGTGTCAAGGGACTAATTTTTGCTCTCCTTGTCCCCTTAACACTACCTGCAGGTACTGGCCTGAATGAAA[A/C]CAGCAGGGGCCTGGGAGGTAGCTCTGGGTACCACTGGGACAAGCACGACTCAGGTCCAAAGAAAATGATAGTATGAGATCATTCATTATAATATTTAGCT
7	52832106	SCAFFOLD10028_2365	0.17	0.19	ATAAGGTTGAAAAAAGTCAACTGAAAACACAAATAAATGAGGTTGCTGATGAAGATAAGGGATGACACCATGGTATCACCACTGACAGTAAGCTCTATGT[A/G]GGTAAAAATAGCATCTATCTCTTTCATCACTGTAAACCCAACACCTGATAAATAAGAAGTCTATAATAATGTTTTGTGGAATTAATAAATAATTAAAACA
7	53252292	SCAFFOLD116197_8648	0.42	0.45	CCACCTCACAGCGTTTGTGTTGTAGCCTCTGCAGGGTGAACATTATTCCAGCTCAACGCTCTAGAGGAAAGCGATCCCCGCCCCCTTCCCTTACCCCACA[A/G]CCACGTGGGACTGAACACCTCGTCCTAGCCTGGAGAGCAGATGCGGGGATTGTTCTCAGCCACAGAAGGGCAGGCAGCCTTTGGTTGCTTCTGTAAGTAA

7	55240708	SCAFFOLD265658_20099	0.40	0.41	AACTAACATGCTGTGCTTCATGATGGGGAAACATCAATCACTGCCTTTCAGCCACTGCTTAAGGTAGAATAACCTTTGTCCAAACTAAAACACTGTGCTT[A/G]GTGTGGGTTTTCTATTATTAGCCTAAAATAGCTTTGGGAATTCTCAAGATAGTTCCTCGGAATTCCCACTGTAGTCAGGCACAAGGGCTACGACATTCAT
7	55241030	SCAFFOLD265658_20421	0.47	0.49	TGCACCAATGTGATGTATGTGTTATTATGAAATGAGATGAACCATAACTAGTGCATCTGGACCACAGGATAATAAGGAGGAGAAAAAAATCTTGCCTCTT[C/G]ACAACTAGGGGAGAAAACTGTCACTTATAAATGAAGATTTTTTTTTAGAATGGATCATTGGTAAGGCATTCAAGAGTCCATACCATGGAGTTGATTTTTA
7	59683262	ADRB2-AF331034-176	0.20	0.17	CAAGTAGAACTTGGAGGTCCTGCGTTGTCCTAGACCGCTCCGCCCATCCTGCTCCACTTGACTGACGTTTTGGGCATGGAAGCGGCCCTCAGATTTGTC[A/G]ATCTTCTGGAGCTGCCTTTTGGCCACCTGGAACACCCTGGAGTAGACGAAGACCATGACCACCAGGGGAAGGTAGAAGGACACAATGGAGGAGGCAATGG
7	59683706	AF331034-624	0.23	0.21	CATTTGTACTACAATTCCTCCCTTGTGAATCAATGCTATCACTGGGCACAGTACCTTGCTGGTTCACAAAGTTTTCGGTGCCTGGGGGGTCTTCACACAG[A/C]AGTTCACTGTCTTTCTCCTCCCCCAGGTGATATCCACTCTGTTCCCCTGTGTAGTCAGCCCTGTCATTGCTGTTGCTGGAGCAGCCATTCCCATAGGCCT
7	59967966	SCAFFOLD100365_20196	0.33	0.33	GCTTAAATCCCCTCAAATCACACATCTGTTCCCTGCCAGGACCCTAAGATAGACTCCTCTTCTTTTTCACCCTATAACTCAATCAGGTACAAGGCCATGC[A/G]GACTTCATTCTCTAAAGGATCTTTCCCCTTATTTCTATTGTTGTAATTACTCAGTCACCCAGTCATTTCTGATTCTTTGCGATCCCATGGACTGCAGCAT
7	61434241	SCAFFOLD115418_11162	0.14	0.21	GGCAAGAAGAGAGAGAGAGGGAGATGGAGGAACCCCCTTCTTCTACCACCTTTTAAGAGTTGCCGTCTGTCCGGAAAGCCTGTTCATAGACTGGAGTGAG[A/G]ACTTGTTGATCTGTAGAGATTTTGCCCACCAGACAGTTGCACTCATTGGGCAAACTCCAGGAGATGGTGAGGAATAGGGAAGCCTGGAGTGCTGCAGTCC
7	61764506	BES8_Contig486_506	0.13	0.10	GCCTGGCATATAGGAAGCCCACAGTAAATGAGGCTTTTATTATTATTAATCAGGCAACATCAATAAGTACACAGCAGCTGGTAGGTGACCTACCTTCTGA[A/C]TCCTAGAGCAGGTTTCCATCCAACTAGGAAGGCAGGGAGAAGGCACAGAACGGTGATCTGCTCTTTCCTGTTTGTTGCAGAGTGGAGCAGCATGGAAGCC
7	61910225	BES9_Contig342_525	0.23	0.30	ATCTGATTATTATTCCAACTAGAGGGCTTTGTAGACCCTAGGGGCTCACCAACTCATTTTACAGATAGGCCCCAGAAGGGACAAGCTTAGGGTCTCACAA[A/T]CTGCTAGAACCAAAGCTGGTGCTGGGATTCAGTTTTCTAACTCCCAACCTGGTGCTCTTTTCTCAATGCCCGGCTATTCTCCTTCCCCCGAGAAGTCCTG
7	62595707	SCAFFOLD321745_32776	0.46	0.03	ATTCAGGAGCCAAAACCTACAGTTTGCACTGTTTTTTCAGGTCAGATCTGAAGCGCATTCCTTTTGACGCCATGCCACCGTGAGCTTCCAAAACAAGATC[A/G]GCTCTCAGGCAAGCCTCTGAATGGGTCACAAAATTCAAAGCAGAACCGAATGAAGCCAAGTTGCCAGGCATGATATAGAAAGCTCTGTTGTGCATCTATA
7	63945744	BES4_Contig405_551	0.45	0.11	TACAAGGTTATGGTAGAGATAAGTGCACACAGTAGAGGTAATACTATAGAAGAATTACAGAAGCTCTGTGAGAATGCAGAAGATAGGCAAATCTACATGT[A/G]TGGTCTGAGAAGGCTTCACAGAGGAAGTTTTAAGTCCAATGTTGCTGGTCTCCAAAGGTGGAAGGTGTAGTCTTCTTCCCTCGTTTTTTAATTTTGTTGT
7	63959387	SCAFFOLD121921_5377	0.01	0.05	TAGAGCATGTAGGTCACCACCTGCTTCTGAAAATAAAGATTTTATAAAGAAAAGTCATGTTCATTTGTTTATATATTGCCCACAGCTGGTTTGCATAGGA[A/G]AGATTGGAATAGCTACAATGGGAAGCATATGGTTACATAGACTTAAATATTTACTATGTGACTCTTTACCAAAAAAATGTAGATGCCTACTTTCTCAGTT

7	65978304	SCAFFOLD12882_751	0.08	0.12	TTAGCAGCCAAGTCTGCCTTGATCCATAATCCCAGTACTTACTGCAATGCCTGGTGCATAGTAGATACGTATTTGTCGATGAGTGAATAAATAAAGGAAT[A/G]CATGCTCTTTTAAATCACCCAAAAAACAGTAAGCATAAACCCTGCCTTTAACCCAAAAAGCCTCCACCCTTTACTTTCTTAGACACCCGGACAGCCTTTG
7	67347672	BES1_Contig630_964	0.29	0.26	ATTGGGTCACAGGAGTCTGGCTTTTGAGCCTGCACTCTTAATCAGTACACTATGCTGCCCATTTTCCAAGTGCTATTTTTCATGAAGACAAATTGAATTT[C/G]TTCAGTGCAATGTCTAAAACATGATTTATTTACCCTAAAGTAAGATAAAGATCCACTGAGGGTCAGAGGAAGGCATTCTAATACTTGTAAATTGGACACA
7	75196686	SCAFFOLD300067_13432	0.45	0.04	TATATTTCCAGGGCTGAAAAATTCCAGGGCATTAAAAAAGAACCCTGCAACATATACAAGAGATGTTTAATAGACTTCATTTAAACTCTAGGAGACCAAA[A/G]TGGAAGTTTTCTAGCAAAGATCGGGTAAAAAGTTGACTGGAGGAAGACCAGGCAAACGAGTTTAAGTCCAAAGTGTCCTTGCTCGGCTTCTACTTTTGGT
7	75421193	BES8_Contig294_1573	0.44	0.43	AATTTGTGCTTGTGAAAAAGTTCTTGGCATGTCTTCTATAGGGAAGACATTTATTCTCTGCAAAACCTAAAGATAGGAGGAAAAGGGATAGTTCCATTTT[C/G]CCAAGTAGAAAGGGAAGACTCTGTTAACACTGGGCAGAGCAGAAAACTGTTTAGTTACATGATTGCTTGCTATAAAGCCCTTTGAAGCAGTCTGAAACT
7	76415984	SCAFFOLD296555_4978	0.37	0.29	AGGTTTCTTATAAAGTAGAGTTAAGGAAATTTTTGTAGCTGAAATGAGGGGGAATGTAACAGAAATAAAGAAAGACGATGGTGAAGTGCAGCTCTTTGAC[A/G]TGATTTGAGGCTGTGCATGTCACTGAATATTTTGGCCAAGTAATCTATGTTTTTGAAAGTTAACTCTGTACTGGTGGATCTCCAACTTCAACATGCACCA
7	76674263	SCAFFOLD135413_2432	0.05	0.02	AAAATCTCTAGATAATTAGTCAAATTATGTATCATAATAATCCTTTAAATTGTCTATATTATATGTTTCCTCACTTTTCTTTTGGAACCAAGTATTTTCA[A/G]GTACAGGAAAATTTTCAACATTTCCAAGAATAACCCAAGCAGTTGAAAAACATTATGACTCTGCCTGTGTGTATTTTAAAATGGTTTAAATAAAGGAGAG
7	79003131	SCAFFOLD228120_3336	0.04	0.14	AACACAATCATACTTTAAAGCATCTCACATTTCATAATGTAACTGAGATGTTCATTATAAATCCATTCATCCATTTGATGAATGAGAAGACTCAACCATA[A/G]CAAATTTCATCCAGTAACTCATTTAACTAGCATTTATTGAACACTTACTATATGCCAGGGTCTCTGCCAGATGCTTCAGATATGAAAGTGAGCCTCTGCT
7	80131729	SCAFFOLD145666_13245	0.21	0.40	TAGAATCAAACTGCTTTGACCATCTAAAAAGGGATGCACTTTTGTTTTAGTTTGACAGAACATAGTTTTCTTCCACAGTCAACCCAACTGTGAGGAAAAT[A/T]TCAGAAATTAAACTAGACTAAGTTAAACTAAATTAATGGAATGCCTCTACTGAGTTTTTTAAGTGAATTGATGAAAGAAGTTTGTTCCTTAACATAAAAT
7	81049743	BES10_Contig573_944	0.43	0.45	TGATGGAGAGGGAAGAGATGAATCATTTAAAAACTGAATACAACGCTCCTTTAGAGATGGACAGGAGACCGTTTCCCTCAGCCTGCTGCTCACGGCATGC[A/G]TTCGCTATATTCTGCGTTATTTATTTCCACTCACTAGGAGAAAGACCTATGGAGGCTTTGTGGCTCATTTATAAGGAGATTTTGGAGGCAACAGCTTGTG
7	83855165	BES9_Contig513_1348	0.42	0.24	TCTCTGGCTGTTCAGGGCAGCTGGGCACTCTTGTTCTCACTTGGTAAGCTTTGAGGGGCTGCCAGGAAAGGGGACCTGAGTGACAATTGTTTTGCTCCTC[A/G]TACGATCCCCTAAAGCTCTATAAATCTGTGTGGCTTGCTCTTTCAAAGAGGATTCAGCTGCTAACAATTCCCTCATTTCTCCTCAGAATGAAAATCTTGG
7	87785272	SCAFFOLD186218_5128	0.38	0.26	ATAGTTTGTGATCTGAAAGACCAAATGCCCAGCGGAAAATCAGAGGTTTTATTATTATAAAGTGGATGGTAGATGACCATCAGCTTTTTAAATCTCACTC[A/C]CTCAATTGGACTATGAGCTTCCTAATGACTAAAACTATGCCTCAGTTCAAATTCCAACATTTAATAAAATGCTTGCATATATAGTTGACTAAAAATACCT

7	89784938	SCAFFOLD300151_9022	0.39	0.37	AGACATCTCAGTGCGATGCTCACCCTCCAGCTGTGCCTGGGTACACCATTTGTTTTATGGACTAAAATTCAGAGCTAGTGGTACCCTCACTCCTGGGCTC[A/G]AAGAGTCATAATAATCCCCTTCCTTCTGGGTTCCCTTGCTTGAGCCAGATTACTTTTCATACTGTTACTTCATATACTTTGCAACGCGATCACATCGCAT
7	90138601	SCAFFOLD246793_2602	0.37	0.41	GATAAGAAGGAAAATCTTTGTCACTTTTAATTAGAAGTCTTCTTAAATATTTTGTTCATGTACAGTCTGATTTTGCAGGCTTAGTTAATAAATCCTGCTC[A/G]TGTGTTGAATTTGCTAATTTTTTTCCCAAAAAAGTGAATAAACTAATTTGTAATATGATAGGTCAGGAAACTATCTTTAGTGATATGGCAAAATATGACT
7	91480741	BES3_Contig337_769	0.20	0.29	TTTCTTATAGACTAATTCTTAAGCTCTCTTTGAGCTAGACCAAGGGATTAAGCAAGTTTATTTATAATACATTTAAATGTAAGAGTTCTGAAGACTGAAT[A/T]TGCATACTTATATCCAGCTATTAATTTGAAGAGTAAAGTATAAGAACTAGTAGTAGAGATAATAAATTTAGATAGTGATGAATTGTAAGAGTTACTTTTG
7	91481018	BES3_Contig337_1046	0.20	0.29	GAACGTTCTTCAAATTCCTGTAGTTCGGCTGGCTGGAAGCTTTGGGGCAGTAAAGGTTTATTGGAAAGCAACAGTGGACAGTGCTGGCCTGGAAGACTTT[A/G]AGCCGTCTCATGGGATTCTTGAATTTGCAGATAGACAGGTATGTTAGTCACAGACATATTAGCAGTTTTTGGTTGTACTTCAGACATTTTATAGGCATCT
7	92504782	SCAFFOLD137569_1142	0.32	0.43	CCTGTAGAGTTCTCAGCAACTATATCTGAGTGACTCTGAATACTCTTAAAGGCTTTTCTTGATTTTTCTAGAGTGAGATTCATGCCACTGTTTTCTATTT[A/C]TTCCTGTCTTTCTTGTTGCTTCACCTCTGGGATTGAATCTAGTATAGAATTTTTATATAGCATTAGTATTTAAATTCTTGAATGTTCTTAGTTCACAAGA
7	92504862	SCAFFOLD137569_1222	0.31	0.43	AGCAACAAGAAAGACAGGAAGAAATAGAAAACAGTGGCATGAATCTCACTCTAGAAAAATCAAGAAAAGCCTTTAAGAGTATTCAGAGTCACTCAGATAT[A/T]GTTGCTGAGAACTCTACAGGTTCAAACCTTTACTCTGTTGATAACCAGTTAACTTAGGTTACTTAAACTCTGCCTTACTTTCTTCATCTATTAAATATGG
7	92980094	SCAFFOLD235985_4468	0.48	0.49	GGATTGAGTTTCCAGAAATAGAATTAACTGCTGGAAAACAGACACACATACCACTGCACAATCACATAAACCACACGCTCAGGAGTGAACCGTGCCAAAG[A/G]AGGAGGAAGACAGATTTAAGCAAGAATAACAAGAATAAATGAACATTGCATGTGGTATTGAGATCTAAAAGGTCTGAACAGTTCTACATGCAGCATCTGC
7	92996601	SCAFFOLD100525_17827	0.47	0.49	AGGGAAACTCGGGAGAGGCTAATTGTTGAATCTGATTTTAAATGACGATGGAAAGATGCTACATATTGCCCTAGAAGAGAAAGGTTGGGTGAGTAGGAAT[A/G]TTTATAACTTGGCGACAGGGGAGATACAAGCACAATGTTGTTAAGTGTAGCTTTAGAACATGTTGGAATTGGCTGTGGTCTGGGGGGGACTATGGAGAGC
7	94411399	BES10_Contig490_1065	0.44	0.50	TAGTCAAAACCTAGAATATGGCCCAGCATATTCTGACCTGCTGGGGGATGAAGTGGGGTGAAGGATGAAGCTCCCTCTGTGAGCCCAGTCACTCAAAAAG[A/G]AAAAAAAAATATTCTAATGGGCACATAGCATTTGAAAGTTGAGTAGCCAAATCTGTGTTCTATAAGGTCTCCAAGTTCTTGGTTAAAATATATTTGGAAT
7	94444968	SCAFFOLD140453_1962	0.13	0.20	TGTTAGGGAATATATTCTAAAGGGTATGACATTTATAACCCGCCCAGATAATTGAGAAAAAATAATGTGTATACACAAATACGAGTGTGTTCACAGAAGT[A/G]CACAGAGTAAAAGAGTAAAGGCTAAAACAAGCATAACGGTTATTCTAAAGTTTGAGTACATAGTATTCTTTTTAAACATAATACGCACTGTACCGTCCCT
7	96198246	BES9_Contig287_1008	0.07	0.16	ATAAACATATTTGCAGTTACTAGTTGAGGGTGTCCTTTGTATAAGTGAGTCTTCTGTGTTCAACTATTTGTTACATTTGTCACTTAAGTAAACTACAGAT[A/G]ATAGTTTTTTTGTTTCTTTGTTTCAGCTTTTAACTGCTTTTGAGAATGCGTTGGGCTTATCAGATAAGATTCCTAGCGAAGATCACCAAGTGCTTTATAA

7	96973132	BES11_Contig421_1185	0.33	0.38	TGTTACATTGTAAATGCTGTTATTTTGGTTTCCAAAAATGTCATGACAGGAAATGATTCCATGGCATAGTTGCCAAGATGGCTGTTGAGCAGAATCAAGA[A/G]CAGGGGGGTGGGGGCAGTTAAACTTAGACAACAAGAAACTGGAAAAGGTGAAAATATTACTTACTTTCTGCAATAAAATAATTCTTATAATCCAAAATAA
7	97150321	BTCN16547-5	0.34	0.47	AACATTTGACTTGTGCAAGCAGGGAAGACGTGCTTTGTGCAATCAATTCAGGAAGTTGGATTTAGTGCTTGAGCTCCTAGCTTTCGAATATCCAGTTTAG[A/G]GAGAAAAAGAAAAAGTACCCCAAAGGATATTAGAAGCAGAAGAATCAATGACAAAACACCCACTTCAGACACAGACACAGGCAAAATCTAAGGGGGAAGG
7	97730611	SCAFFOLD5869_6784	0.21	0.25	CACCAGCCCTGCCACCAGCTGTCCAGTGTCACAGACCCATTTCTCCATGGTCCCACAATCTTTACTCTTCCTCTCCTCACCCATCTATACTCTTGACTAA[A/C]TGGTTTTGCTGCTGAGCTTGGTCAGAGGACCACAATAATAGTGTCCCCTCCTCAGCCCCTACCATAACCTCTCCTCTGCTAGGGGAATGTACCCATAGAA
7	97763034	BES4_Contig422_901	0.39	0.45	ACTCAGAATAACTGGCTTCTCTGTTAGGTTCTAGGAAGGGACGTCAAAAAAAAAAAAAAAAAATAGATATGGCACCTGTCCTCAAAATTTCCAAATACAC[A/G]TGGAGGAACAATTCAAAACCACAAATTAAGCAATTAGAGAATGACAAATACATGAAAAGTTTGATATTATTTGGTACTAGATATTAATATTAGCTGTTTA
7	101479993	SCAFFOLD131848_6299	0.38	0.35	AGTCTTTCTCCAAAGCCCATGGTTTCAACCATAAAAATCTAGGCTGTAAAATATAAGAACTTTCCTTTCCAAGGGCATTTCCTAAAAGCCTCTCATTTGG[A/G]GAATTTCTTCTTTATCAAAGTCCAGGCTACTGACATATATGTACACCCTGGTACAAAGTATCTGGTATTTTAGCCACTGCTGTTTCTTTTTCATGAAATT
7	102131335	SCAFFOLD255186_3538	0.06	0.15	TTGTTATGGTAATGAATGCATTTAGTCCTCAAATTGAAAAAAAAATGTATACTGCTTCTTTTTATAATAAGATTTATTTTTATTATGTAGTCCTTTGTTA[A/G]TCTTAACTGGGAGATGGTTTTCTTCAAATATTATTGGATCCACATTTAGAAGGGCCTGCTTTACAAAGTACGGGCTGATAATGTTTTCCAATCAACAAAG
7	102131407	SCAFFOLD255186_3466	0.43	0.45	TTTATTTTTATTATGTAGTCCTTTGTTAATCTTAACTGGGAGATGGTTTTCTTCAAATATTATTGGATCCACATTTAGAAGGGCCTGCTTTACAAAGTAC[C/G]GGCTGATAATGTTTTCCAATCAACAAAGCCCTTGAAACTGTTCAGCTTGTTAGACATCTGCTGTTGAAGAGTGTATCCAGCTCTGAAAACAAGATGCAAG
7	102131470	SCAFFOLD255186_3403	0.15	0.25	TGGATCCACATTTAGAAGGGCCTGCTTTACAAAGTACGGGCTGATAATGTTTTCCAATCAACAAAGCCCTTGAAACTGTTCAGCTTGTTAGACATCTGCT[A/G]TTGAAGAGTGTATCCAGCTCTGAAAACAAGATGCAAGGAAATGCAGTCCTCATTTTCTCCAGCAGTTAGTTGCTCAAAGGACTATAGCCATTTCAAGAGG
7	105595593	SCAFFOLD15514_11206	0.39	0.46	CTTTCTCATTGTGACGTCTCCTCTCATTGCAGACTGCAGGCCCTAGGACACCTGGGCTCAGGAGTCGTGGTTCATGGGCTGAAGTAGTTGTAGCCCACAG[A/G]CTTAGTTGCCCCGTGACATGTTTAATCTTCCAGACGGGGGATTGAACCTGTGTCTCCTATATTTACAGGCAGACTATTAACCAGTGGACCATCAGGGAAG
7	105808578	BES8_Contig198_1077	0.18	0.19	AGCAAGCTGTTTTGTTTTGACTCTCTCATAAACCCAGGTCTAACACACATTTACTTGGCCATTCTTGGAAAAAAGAAATCAAAAGAGCCAGTCCTGGAGC[A/G]TCTGAGTACTGGTAAGTTCAGAGAGCTTTCAGCGTCCAGGGGAGGCAACAGAGAACTCCCATCTGAATGACTGCTTCTTAATGGAGGGATGAAAGGATAT
7	106483103	SCAFFOLD55366_2475	0.08	0.08	GCACAGTGTGTGTTAACAGTACTTTTGTAACCATCCTGGGATTTTTTTAAATGACTTGTTATTTCAGCAGTCACACACACACACAAAAGTGACTGCTGAA[A/G]TAGGCAAGAATTAGAGCAAGGTGATAGAATCTAGAGCGTCTTATAAAAGCCTGACCTTACCGCTATAATTTATTTAACTCCCACACAAGCTTTTCTTCTC

7	106483290	SCAFFOLD55366_2662	0.08	0.08	AACACACACTGTGCATCCACATATAGCCCATAGCACTGAGGACATTGGAATAAACTTGGAACTCTGCAATTGAAAAGTTATTTTTAAATCTAAACCAGGC[A/G]ATAATTTTAAAAAAGGGTGGGAGGGGGTAAGTGTAACTGAAGCAACTTAGATTTGTTTTCTGTTTCCATATTGTTTGCCTTGCTCCTTTCCTTTCTGACA
7	107345908	SCAFFOLD255050_10929	0.29	0.33	CACATTGTAGCTCATCAGGCAAAGCACACAAGCAGACACGGCTATTGCCTCCAGCACTCAGCAAGCTGGACACGTCATACGCACGGCCCGGTGACAGACT[A/G]CTGTGGTCTATTCTCCTCACTGTCTACTCTGGAACCCCTGATTCACCACAGCATTGGTATACATTTGTTCACAGGCTTGCTGACTCTAGATTAAGTCAGC
7	108057541	SCAFFOLD150127_23694	0.23	0.31	TAACAGCACGTGAAATGTTATGCTTCCAGTTTTGAAGTTGAGAAAATTAAGGCTCAGGGAAATTAAATGTGACTGCCTGAGATCATAGAGCAAGTGAATT[A/G]TGGTCCCTGGGTCCAGATGTTCTGCCTGACTTTAGAAAGTGTGGTCTCAACACTCTGACCTTCGGCCTCTCTGGCTTGCCTCACTCAAGCTGGTTACAAA
7	108101556	SCAFFOLD200327_5844	0.19	0.24	GGAATCCAGATTCATTTATAAAGTATCTTGTGAAACATCTTGATGTGGAAGCATTTCTGTGTCGTTAGATCTTTTAGAGTTGGATAGAGAGTGACACCCT[A/G]TAAAAACTGGGAAGCGCTTTAAAGTTTGTCGTGCCTTGAGCTGAGGTTTTCAAGACTGTGTCACTATGAGTATGCAGAGGGGAGTTTAAAATGTTCAAGC
7	108763313	SCAFFOLD280597_793	0.43	0.21	GAAAAGGGGCGTTCTGCTGTCTCTTTCTCTCTTAAAGTGATGTCTGCACAATTCCTCCCTATAGAGTCTGCTTATATGCAGCAGAATCGATATTGCCACA[A/G]AGGAAATTGGGAAATGTGGCTTTCCATCCAACTGGGTTTTCCTGAAGAGGTCTTCCCTGGTGACTCGGATGGTAAAGAATCTGCCTGCCATGCAGGAGAC
7	110569115	SCAFFOLD260285_24265	0.47	0.50	TAATTAGGTTGCCTCCACATAGATTAATGATAATTAGGATACATTTTCTTCTACCCCATAGTACGTGGCACAGTGCTCTGCAAGGGTATACCTTTTGTTA[A/C]ATATGCACACAGTTACTTGCTATTTAGACCTGCTAAGCCCAGTACCTGAGCTCACTTTACTTGGCAGCTGATGGTTGCTCTTCAAAGTAGATGCGAAGGA
7	110964595	SCAFFOLD105909_15047	0.02	0.08	CAGATTGACCAAAGCGCAGCCTAATAAAGGAGTTCTCCAGAGTTATAGCTTCTTGAGCACTTTTTATATTATTCATGTTAAAAATTCCTTCATTTGCTAT[A/G]TTACCATTATATAGGTCATATTTGGCTAAATGTGGATTTGAACTTGATGTTTCCAAAATCTTATACACTTTCAGTCCCAGTGGTGGCATCTCTGCTAGAA
7	111352039	SCAFFOLD317872_1752	0.46	0.45	AAATATTCTTTTTCAATCTCAAGCAAAAAAAGACAGCTAATATTAGTTAAAGTCATATTGAAAAATATGACAATCCAGGAAGCTAGTCAGATTTTCAGGC[A/G]TTCCTGAGGACTATCAAATTGGAAGACATTAATGCAGAGCCATGCTGTAGTTTCAAGTACCCCCACATTCAAACTACTTGTGTTCTCTGTGTCTCATAGG
8	394920	SCAFFOLD116505_320	0.21	0.25	TACCTTTAGACTGAATCTGCATAGAGCTCCTCTGCCAAACACCACCAGCGACGAGCTATGGACGGCCAAACCCAAACAGCCCTGTTTAGTTCTTCAGTTA[C/G]TGACCTGTTGTCAATATCTGAGATTTTCATGGCATTTCTTCTTTGAAAGGGTCTCTTCTCCCTTGACTTCAGCTACACTATTCCCTTCCAAATCTGCTCT
8	1869794	BES11_Contig407_907	0.12	0.19	AGAAATCTTCCTTGGGGAGAAATCTTCCTTGGGTGTGGCAGAATGGGTGTGCCCCATTCCTGTCAGTCTGGTTCCATGATGTACTACAGGTACACCTGTG[A/C]GTTTTGACAGCGAGGTTTTAAATTTAGGAAATCCACAGTCATCACTTTGACACTTTGAGGAGTGGCTGCTTGTCACAATGAGTTAAAACATGCTTATGAG
8	3820328	SCAFFOLD217834_1689	0.37	0.37	GACTAGTAGATAACCCAGTTTTAAAGAAGATAGAAAATCAGCAATAGGATCTCATGATAAAATATTCTATTAATTTATGCAGGCTTTCACCTTGAAATGT[A/G]ATCTGTGAAAGATCACCTTACTTGTGTTTTCATGACTTCATCTAATCCAAAAAGTAAATATGATGAGTTTACTTGCTAAGCTTTTGCACATGAGATTATA

8	4168839	SCAFFOLD195145_10221	0.50	0.44	AGTTTGAAAAATCACACAAGCTGTTGGAATATTTGCATAGTGATTCTCTGCTGAATAGCAGCTTGGGCTTTCCATTTCCATCTGCTTCAACTTACTAAGT[A/G]CAAGAGGTATTATTATTACGATCATCATTATTATTATCATTGAATTATCTGAATTCCAAATGAGGAAACAGACAAACCACATAGCATCTTTAGTGGCATA
8	4723566	SCAFFOLD280322_7667	0.39	0.26	CTCAGTTTCTCTGTGCACCATTGCCAAATTGAATCTCAGAGACAGAATTTTGGATGAAGTAGAAAAAAATAGCTTTATTGCTTTGCCATGCCTAGGCGGA[A/C]ACAGTGGGTTCCTGCCTTGAAAAACTGTGTGTCCCCACCCTGGAGGATTTGATGAGGAGTTTTATAAAAATGCTTCACAAGGGTGGACTTGATGACAAGG
8	5170144	SCAFFOLD140642_7473	0.14	0.07	ACGTGTCTCACTGTGATGTTGATTTAAACAGATGGGGTCTTACACTTTGCTAAGTGAACAAAACGTGTGGCTATTGATGTGGTTTGTGTGCAGCCAAAAG[C/G]CTGGGGGTGGGGCCACTTACTTCCCCTTGAACAGACCACCAGGATTATTCTTAAAGGCCTCACTCCCTACTTCTCTCCTCTGGACACACACTGCCCTAAT
8	8125073	SCAFFOLD116640_2128	0.19	0.17	CCTGGCTCAGATGAACCTCACATATGGAACTAGGGCTTTATTCACAGTTCTTAGTCAATACAGCTCACTTCTTACTCAACGTTATAGTAAATAAATATCC[A/G]AATCTTCACTCCCTTCGTTGTTTAGTCGCTCAGTCGTGTCCAACTCTTTTGTGACCCCATGGACTGCAGCCCGCCAGGCTCCTCTGTCCATGGGATTTCC
8	8127600	SCAFFOLD116640_4655	0.48	0.48	TGTACACCAGAAACTAACACAATATTGTAAATCAACTATCCTTCAATTAAAAGGAAAGGGCCTGCCTCAAAGTCAACCTGGACTGGAGTTGATCAAAGAG[A/C]GTCTGAGAACCACCTGCATCAGAACGCTCTCTCAGCGGGTAAATTATGCAGATCCACAGAGGGGGCCAGGACAAGACCTAGATCCTCTGATTCCAAGAGC
8	8529543	SCAFFOLD160202_23463	0.23	0.30	TGGGATCAGGTGGAGACCACTGGGTGGAGGCAGCAGACATGAGAAGATGCCCCCCTCCCCGGTCAGGCCATTGCACTGACCCGTGGACAGAACTATTCCC[A/G]TGGAAGCACAGGCTGGGGGAGGGGCTTGGGCTCCTGTTCAGCTTTGCCGCTAACTCCATCCTTTATTCAGCAGCTTTCCGTGGATGACCTGCCTGCTACC
8	8658053	BES7_Contig155_926	0.10	0.16	TTAAAAAGTAAATTTCTTCTTACAGTTTTATTCATTGATATCAGTTACTTGGTGTCTTATCAACAAAAATATAAGGTGCTTGTTTTCTCTCACAGAAACC[A/G]GAGGGTTAAAAGCTGATATGAATTCTTGTCACAAATACAAGGAAATGTAGAGCACTATTCATTTTCTTAAAGTTCTATTTTCAAAAACTAAGGTTTAAAT
8	11229947	SCAFFOLD280171_4902	0.48	0.42	AGGTTCTTGGCAACCTTAGAAAAGTTACAAAGATATATTTTCTCAAGACTTTGGTGAGGAGATGTCCTAAAGCCTACATTTCCCGCAAGAATAAAGACTT[C/G]GGGTAGAAATAAGATGGCACAGAGGATAGCGAGGTTTTTCCAAAAAGGATTTCATAGCAGGTCTTAGGGAAGGAAGAAGGATAGGACAGCCAGATGCCAA
8	13562780	SCAFFOLD313402_3070	0.48	0.49	ATACCAAATGAAGCAGAAAAAAATTTGAGGAGATGACTGGGAATTTTGAATATAACTGGAAGAAAACGAATTATAGATCTAGTAAATTCAGAGAGCCCCA[A/G]AGAAGAATTAAGATAAAAATTAAACACATAATGAATAATGATAGCTAAAGTACTGAAAACCAAACACACACACACAAAATCTTAAACTCAGCCACAGCAA
8	16496200	SCAFFOLD146494_5715	0.22	0.12	ATATTCCAACATTTTTGTTTTTAAGGAATCAAACGAAATTTGAAGAGCATTTTACCTGAAGTTAATATTCATGTATCTGGTACAGAGAATTTTGTTTTCC[A/G]CATGGAAAAATTCACGCCTCAGTTTGTATATTCTTTAAGTGTTAGCCTTATGAGAGAGGTGGTCATTGAAATCCATCCCTGAAGGTCTTATAAACAAACT
8	17008968	SCAFFOLD155453_19758	0.21	0.24	TAAAAACAAATTTCTCCCATTAAAGAGATGGAGAAACTGATTTGCAAAATAAGCCGCTGGCCTGGACTACACGTGTGGCCACATGTCTGTAAGTGCACAC[A/G]TGTGTGCCTGCACATATGAACATTTGAGTGGATACATACGCTTGCCACCCACAAGAGAAACTGCCTTCATTCTGTTAGAGCTGCGAGTTACCCAGTCTGT

8	18201094	BES9_Contig548_2778	0.46	0.50	GGATTGATGAAGTGCTGAGGAGTAATTAATTGATCGCTAAAGCATCATGGTATTTTCCAGTTCCACAGTGAATTCTCTTAGTTTTAGAAACACTTTTAAC[A/C]GTAAACTTCATCGTTAAGGCTGTTTGCTTGTTGTTATTTCTGTTATTTTTCCCCAAAGATGTCACATGAAGGCATAAAGTAGTCAAAGACTCAATTTTTA
8	18747802	SCAFFOLD311898_4113	0.20	0.27	ACACAGAAATATGTTAAGGTCTATTTTTAAAAGTTTACCTGTCCTGAAATAAACGTGTTTCATGTATTTGTTTAAACTCTGTGTTCGTCTTTTATTTTCA[A/G]TATTTTCCCACTGTCCATGATAATATTTTATACAATGGATTATTTAAAACCTCTGTGAAATTTGCCTGGAGGCAAGCTTGCAGTTCAAGAATAATTAAAG
8	19404854	SCAFFOLD1524_5146	0.46	0.47	CTACTCTTGAACTTCACATGTTATTAAGTGAAAATAAATTAAGATTTAGTGTCTAGCCAAGAACAGGGGCTCAATGATTGTGCATTGAATGAATAAGTAA[A/G]TAGGAGTCAAGGCTAATATAGGAGCTGATATAAGAGCTGAATGTACTCCTTTTCTCATAGCTGATACCTTATCAATAACATATAGCCTTCTTGGGATGTC
8	21462326	SCAFFOLD262688_3069	0.39	0.36	CTGGAGCAGGGACAGAAATAGCCAATTTGGGAGGAGGGTTATGGCCAGAGTATAGAACAAATGACAATTTTCTTCCTTGCGTTTCCTGGCAGACTGCCTT[C/G]CTGGAACACATACATATAGATAGTTGTCACACAGCTTAATTATATGGAGCACAACTGTAGATGACACAGAATATATATTTGCTTCTCTTTGGAAATACAC
8	22707523	BES9_Contig507_1365	0.24	0.31	TATTCCCAGAAAAATGAAAGCATTAGTTTAAAGTCACATGTTTAATTAGGAAGAGAGCTGGCACTGGGGCCTAGGCCTCCTGGCTATAGTTTTTCTTTAT[A/T]ATTGTTTTCTTGTTTGTCATCATATCTTGATGAAAGTGGGAGGAGGAGGAATCCCTCTTCCTAGTAGTAGAAGCAAGAGCCAATAGAAACTTTTAAAAAT
8	23974535	SCAFFOLD125542_14933	0.26	0.25	ATACTATTACTCTGAGAATTTCCATCAGATTGGCTCATCAGTTACTAAGGAAACCAGCAGCATGGGGCCCTTCCTTTAAACAACTCAAGTTACCGCCATC[A/G]CCAGGGCTAATATTTACCTTTTAGGAACTAGCAGATGGGGAGCCCTTTTGGCCTAAGTATCAGAACAGCCACTAGGTGAGGTAAGGGCAAGCACATTCCT
8	25568605	SCAFFOLD131125_6609	0.50	0.39	TGTTAAAGTTTCTAACAGGCCACCAGGTTTGGAGGTTACAATCCTAGCAACAATCTTCTGGTTGTTCAGCTATCAGGATTCTGTTTCCCAGGGATCCCCA[A/G]TGCTCATCACGGACTTGTTCTGGGTACAGATTTCTGGAATGCTTGTCTACCCCAGCCTCTGGGGGCTGCAGCTAAAAAGCTATGGGTTTAACAAGTTCTC
8	27104962	BES6_Contig355_1291	0.16	0.25	GTGCATGCATGTGTATGTATGTATTCAGACATGTGTCTGAATATAGGAGGGGATGGGGAAGGAGGCAAAAATAATTTAAAAAATGATCATAAGGGAAAAT[A/G]GAATGAATAGTTGGTTTGGGGGTAAAATGATTCATTCTCCATTGCCTTCACTATTCTGACTGCTCATGTAATTTCTATGTAGGACAGTGACCCAATTCCA
8	27314753	SCAFFOLD157160_911	0.17	0.20	TGCACCCCAAAGCTGCATTTTGAAAACAATTTTAAACTGAAGAGGGGGTAATGAAATCTCACCCTGAAAAACAGTGCCATAAATTGAGTCCTCGATGTGA[A/G]AGTGTTTCTTTGGCATAGGTAATGTATTTTTGTAACCTTAGTTTTACAATTCAACACTTCTGCTCTATGCTAATCTTTCATTATAAAAACATTCTGAAGG
8	27394429	SCAFFOLD144544_2876	0.13	0.15	ATATATTTTTAAAATTGTTTGTTCCATAGTCTCAGATCTCATACTCAGTGATCTCTCCCTACAAAATGTCAAGCACTGCACGTTACAAATGAAAGGAATG[A/C]AACAAAATTTAAAATTTGATTTGACTTATCTCTAGCTTCCTGAAGCACTAGAAGTCTCTGCTAACATCTCTACAAGACTTAATTCAGGACTTCTCATACT
8	29788196	SCAFFOLD195714_964	0.40	0.33	CTGAATATCATTATTTAATCGAAGATTAGTCCACGCCTGTGATATAGGCTGTAACTATTATCTTCTTTGATTCTTTACATAAGCACACATTTTCTTCCAG[A/G]ATTAAACCTCACATTCATAGAGGTAAATACTACTCCATCTTTGACCAAATGGCCAGGAGCATGATGAAGTAACCCATTGGGATTGTGACCCTCTGTATCT

8	29979793	SCAFFOLD300129_32456	0.47	0.31	AGGAAAGGTGAAGCAGGGTGTGAGCTGCCACTGGGGGCCCTGACCAGGCTCCCTGGTGAGGGGGCGTGTAAGCCGGGCCCTGAGGACAGGTATTCTTGGC[A/G]GCTGCAGCATGAGCACAAGGGCAAAGGGGACAAGAAATGACGGTGGAGATTGTCAAGGGCAAGATCGCACAGGGCCCTGTGGGTCTCTGGAAGGTTTTAG
8	30264345	BES10_Contig593_1041	0.02	0.07	GGAGATGCAAATAATTTTTCCTCATTTTTCTTCTGTCATGTAACACTATGTTTCACAAGTAATTGTATTTTTTTCTTCAGAGCCTAGAACTAGCCCCTTA[A/G]ATAATCAGAAAAGTATTTTTTACCAAGGAAAAAAATATATGTTTCATAGCTCAGTACAGTATTAACATTCTTTCTGGGCAGCAGACGACAATAAGACACT
8	30490120	SCAFFOLD45297_18349	0.49	0.49	AAGAGTACATACTCTATGATTCCTTTCCTATAAAGTACAAAAAGGAGGCAAAATAAATTAATGTCCTGCTATTAAAAATCAGGATAGCAGTCATCTATGT[A/G]GAAGACTTTGATAATTTTCCATTCCATGACCCAGGTGCTGGTTATAAGTCATACACATAGCTAGGCACATTTCTTTACATATATTATACTCTGTGGTTCC
8	32029890	SCAFFOLD135622_1280	0.20	0.26	TTTTTCACCATTCTGTCCTACTGTCTCCATTTTTTAAGGGCTTACAGCATAGCTGGTGACATGCAGTTCACAAATTAGAGAATATTAATATCAAATAATT[A/C]CGTGAAGTAGAAATGTTAGTCGCTCAGGTGCATCCAACTCTTTGCGACCCCGTGGACTGTAGCCTGCCAGGCTCTCCTGTCTATGGAATTCTCCAGGTGA
8	33016310	SCAFFOLD103011_2035	0.14	0.12	ATCTCAGTTCTAAGTTTTACGAATCAAAATTTCTTAAGAGATTTGTTTGGCTCCCATGAAACAGAATATCAGAGTGAAACTAGCAGCTGAGTTCAACTCT[A/G]TTCTCATTCCTTTTACCTATTATCTCCAGTAATTAAAGAAAAATACAGAATAGTGCAGAAAATAATTTACAGGTAATTTTGTACCCACTACACTAAGGTA
8	36274938	SCAFFOLD180501_2533	0.34	0.39	GTAAATAGAGTAATAGAAAAAGGACAGTATCTATTATGTAATCAAATACGAATAGATTGTTGGAAAGGTTTATATCCAGTATCTCAAAATGCTTGAACAA[A/G]CAATACATTCAATTACATATGCTCTTGGAATTATAAGCCACATTCTCACACATATATAGCTATTACTACTTGTGATCCATATTTCATTACTTTAACCTTA
8	39498104	SCAFFOLD130478_1823	0.47	0.49	TATTTGCCTTATTGGTCAGAAAAGTCACATATCCAACCTCTAGACCACCAATTATACAAGGAAATATGATGCTAATACTTGATTAAACTCTTCATGATTC[A/G]GTTCCTGAAAGAAGGTATATGTCAACAAAGGAAACAGAGACAGAAATGATTGCTGCACAGCCAAGTAATAGTGTCTCCAATATATGGCAGCTTGAGAAGT
8	39820920	SCAFFOLD175694_9694	0.10	0.18	TAATAATAAAACCGATTTACCAGCAGCGCCAGTTAATTATGCACCATTGCTATGGTTATTCTGTGAGACAATAAAAGGTAATGGGGCCGTTTCTGAAGTT[A/G]GTGGGAAGGAACCCAGCACAATCACCGAGGTGGACTGCTGACTCTCTTGAAAAATTTCTCCCCAAGTCAGCATGTGTCTGGGTGAGCAGAAGTCATTAAA
8	39821138	SCAFFOLD175694_9476	0.15	0.22	AACGTGCTTCTGTTGTAACAAACCAGCTTTTCCTTCAGGTTTGTGTATAACCCTCGGCCCTTGGCTAGCCTATGAATGCTATTTTTTTTTTCCACTTGGT[A/G]AAACAAATCTAATTGGAAAACTTGTTGATGGAAAATACTTTATAATTAGAGGGGTTTTGTTGTTGTTGTTGTTTTAAGGAAGGGCAGGTTGCAGCCCTCA
8	40453705	SCAFFOLD40157_4581	0.17	0.23	ACCCAGCCTTACCTGAACTTTATGTTCCTATATTCAACTGCTTGCTTGACTAGTATTACAAAATTAGTGACTCCAAGTGGAGATCTTGATGTCCACAAAC[A/G]TGTCCACTGAAATCCTACTCCTCACTCAATCATCAGTAAATAATATCATTGTCTCATCCACCTGTTGAGGAAAAAGCTGAATTTCATTTCAATTCTCTCT
8	40456636	SCAFFOLD40157_1649	0.17	0.22	GTCAGCAAAATCTAAACCATATGCAAACAGTAATTAAAAGTCAGTTTCAAAACTCACTGAACACTACTAAAAATGGGGTAATAGCTATTCTAATCACTGA[A/G]TTACTGTTTCCAACAGTAGGAAAAGTCCAGTGATTAATATTTGAAGTTAAAATTTAACTTTAATATTAGGGGAAAAAAAAAATCAAGACTTAGATCATTT

8	42424418	SCAFFOLD226597_2045	0.26	0.26	TCTGACCTTGACGTCACCATACCAATGTTCAAGGTCCCTGCCGAGGGCACAGGGCAGTAGTGGATGCTGAGTGGGGCAGGGGAGCCAGTGGCCATCCTGG[A/C]AAGCTGTGGAACTACAGATGTTTGAACTAAGACTTCAAGTCCGCTTCCCTCACAAGTTTCAGTGTCCCACCAGATTTCACTCACAAAATATAATTTCAAA
8	45269167	SCAFFOLD66519_10623	0.21	0.48	TGTTGTTCATTTATAAATATTTCTGTTTAGGCTTTGATCAGCATTTTGCAATGAATATTGTGGTCATTTTCCAGATGGTGATTCTCAGCCTGAAGTGATT[A/G]TTTGGAGGTACTTGTGGTTGTCACAGTTGGTTGGAGGTGCTACTAACATCTATTGGGAACAGGCTAGGGATGTGGCTATATATTCTACAATGCATGGGAG
8	45269457	SCAFFOLD66519_10333	0.13	0.30	GTCAGAAAACCCGGGCATGAAAGAGACTTTAAATGAACAGATGAATAGAGCAATCAAAGGGCCATCCAATCAGATCTAAGCAGATTAGAACTTCATGCAG[A/C]CTCAAACACAAAAATAGACTGAGTGCTCTGTGATTTATTAAGGTCACTCATTCAAGATGCTCATGTATCCCTGACCCTCAACTGCTGCTCTTGGGGCTGA
8	45507466	SCAFFOLD140665_2003	0.17	0.28	CAGTAAGCTAGGTGTAAAAATACCCTGAAAAGGGAGATGGTTCTAATATTGTAATAATGCTTGCATTCCCCCCTGTACAGATATTCTAAGTAACATGTGT[A/G]CAGATGGTGATTTATAGCATCCAGATATGCTCTGTAAAGTCAGCCACATGCCAGCCTTGGCTTCCCTGTCAATCTCCACCACATCCCCAGACACACATGG
8	45656881	SCAFFOLD280513_16380	0.21	0.37	ATAACTGTTGAAGATTCTCATTAAGTGCAGAGTGTTTTCTAGTCATGAAGTCCTGGTGTCAGAGACAGAATCTTGCACTGCAGTTGCCCCTCTGCCCCCA[A/C]CCCCAGTCATCTTTGTTTTATCTTTACAGTTACTAATTGTTTTCCTCCTATTAGGAATTTATTCTTCATATTAAGTATAAGGGCTTCCCCGATGGCTCAG
8	45951327	SCAFFOLD300011_37178	0.19	0.22	TGCAGTTGTCCAGATTGGGGAGATGGTATGATTTTTTCATAAAACAAGTACAGGAGCTATAAATACTTCTGAGTTCACAGACCAGCAATGGGCAGCAAGG[A/G]TCTGGAATCACACTTGCAGCAACAAGGTCTGTATGTATAAAATGAGAATGTTGGTGTAACTCAATGATTTCCAAAGGTTGCGTCCAGACCACGACCCTCT
8	47837322	SCAFFOLD145007_39103	0.37	0.25	AACAAAAGGTGCAGGAGAGAGCAGACCCATTTCCTTCTGTGGATCTTTATTTACCCAGAAGTGTACAAATCAAAAGTTGCTTAACAGGAAGGAAACTTAC[A/C]TTCTGCATCAACTCTAGCAGCATCTTCTATCTACATGAGGCTTTTCTGGTCATACAATAACCACCATAGAAGCCATTCATTTTGTGGTCCCCTCTTCCTC
8	47837607	SCAFFOLD145007_39388	0.30	0.48	CCCTCTCTTCTGTAGGATCACACAGGCAGCCACTGAACTGAAATAGGAAATAAACAGCATCAGAGCTAACAGACCAACCTGGGAGATGAACTTTCCAGGC[A/G]AGGATACCACAGTGAAGAAACACGTGGACACTTGGCTGAGGGGGGGACTGACCGGGGTTTGTAAATGCTAGAAAGATGTTCATGGGGTTATGCTAATTAA
8	49116743	SCAFFOLD195121_5031	0.22	0.16	TATTCTCTGAGATGCCTAAAACGCTCCAGGCTGCCTTTACTCTCATCTAAGTGTCTACCAAAATGTATTTTGACCCTGTATTTTTGAGCATATAAATAAA[C/G]AGAAGTAGGATAATTGTGTCACATAGGTTATAAATTATAGAAGGCAAATATCATGTACCTTTAGAGTTTCTTGAGGTTAATGATCGATATTTGTAGAACC
8	49116897	SCAFFOLD195121_4877	0.42	0.39	ACGGCAAAATAGATTGTCCTTTATTTTTGTTTTACCTGCCTTGCCAGGTAAGTGTCTTGAATTTTGCCTGATAAAGTCTCAATATATCCAGAGACCAAGG[A/G]CATTGTTGTAACTGTTGAAAAAGCCACTGTCCCTAGAGTTTACCCCAAGATGCTATTCTCTGAGATGCCTAAAACGCTCCAGGCTGCCTTTACTCTCATC
8	51275639	SCAFFOLD90375_6220	0.45	0.49	TTCGCCACATGATTTCCTCCTTTTCATTTTCAGTTCAACAGCCAGGTCCATACTACAGCTCTTCCATAAGCTCTTTCCTGGTTTTCCTTTCTAACACTTA[A/C]CTCCAGCCACATGTCAGGGTGATTTAACCTCTGCCCTTTACGTAGTAATTACATCACATACTAAAGTTAACCCATGGTCCATTTTCACCTCTGCACTGGG

8	51279036	SCAFFOLD90375_3106	0.44	0.49	TGAGAATGTGTCAAAGGCAGCATAGCGTGTCCTAACTCTCTTGTGGAATCAGGGTTGTATCACGTGTTAAGGGTAAATTATCTATTTTGTAAGTTAGGCC[A/C]AAGTGTGCTCCTTCAACTAACTGTGAAAGCATTCTTGAACACCCATGAAACCTTATTATTCTAATTACCAACAAAAGGGGTTGATAAATGAATGATGAAG
8	51653238	SCAFFOLD76252_6147	0.40	0.38	ACCCACAGGCATCCACTTTGTTTCCCTTCTTTAGGAGGGTGGGGAGCCTCAAAAGGACACTCATGTTTCTAACTACTACATCATTATTCAGTTTCCTGTA[A/T]GATTCTTGAAAAACCAGCTCTTCTGGAGTTAGCACTCATGGTATGTTCATACTCTTCAAAGTTTTTTTTTTCCTTTTTGAAACTTTTGTTCTTCTCAGTA
8	52301015	SCAFFOLD180353_11672	0.14	0.27	AAAAGTTCTTTTCAGGACTATCCTGTTTTTAGTCCTCAGGCCAGAGAGACTGGGCTTTTCTTAGGACTGTTTTATTTTGTTCTAGTCTGTGCTCACTGAC[A/G]TTTCTGAATTATGGGCTGCTTTAGTATCCAGACCAGGACATGTATGTCCTTCCTTAGGCACTCTTCACTATGGCATCCAGTCCATTTTCTCCTTTCCACC
8	54618622	AJ496785-128.SP6-131	0.49	0.42	AGTAGCATGATGATGGAGCTCTAATACTGGCTGAAAGGTGAGAGAGTGCTGATCAGTCCAGGGATGGATATTACTGAATAGCAAGGTCTTCCAAGGGTAC[A/G]GAAATGAAAGGAATGGGCACACTATCCATCTTATGGAAAATGTAAAAATGTTGATTCTTCTTGACTGATCCATCACATCTTGAATTACTGTTTTTTGTAG
8	57153862	SCAFFOLD133314_2838	0.21	0.23	CTGGGGTAAGATGGGTGAGAGGAGATATTGTCTTCAGGCCGTGGCTTCATCTTGCTTTAGGAGAGTCTGATGTAGCTGATGGCAGGTGTGCTGTTCTCCC[A/G]TCAGGGCTTTTCAGGATTTATAGATGCCTCAGGGCCCTGAGCTGTGTGCATGCTTGTCTGTCTATCACAAAACATGAAGCTTCTTCTGGGGTTGCCTTTG
8	57662749	SCAFFOLD115201_15533	0.17	0.26	TCTGGCCAAAGGATCTTAAGAAGCTGGGAGCTCTAAGAAAACTTCTGCTTTTCTTGATAAAATTAATGATCATTCTCCTTCTTGATTTGAATAAGGATGG[A/G]AACTTCAGTACCCATACTGTGGTTATGAGGTCAAACTATACTGTAGCAGCAATAAATGATACACCAAGTAGCCCATATCTCAAGAACTATTATGTAAATA
8	58282117	SCAFFOLD191230_2910	0.21	0.24	GCTACATACCACAGTAATTGTGGATGGACAGGGGTCAGCAGGAAACAATGAAACTGTCAACACAAACGAACGGCAACTGGGCATGGGTAGCAAATAGGAC[A/G]TGGGTGTTTTATGAGGAAAGCCCAGGAGGTTTGAGTCACAGGGAAGAAAGATGGGTTTTTCCATCCAAATTGACCACATATTATAAGTTAATTAAGCTTC
8	58282177	SCAFFOLD191230_2970	0.19	0.20	TAATATATATGCATATATCTGTTTCCCTCACAATTTCTCCACTATCTGGAACAATTTCTAGAAGCTTAATTAACTTATAATATGTGGTCAATTTGGATGG[A/C]AAAACCCATCTTTCTTCCCTGTGACTCAAACCTCCTGGGCTTTCCTCATAAAACACCCATGTCCTATTTGCTACCCATGCCCAGTTGCCGTTCGTTTGTG
8	58554851	SCAFFOLD95308_978	0.44	0.08	TTTCCAGTACAGGTTCAGGCCAATTATGCCTTTGACATCTGCAACTGGCATTTTAAATGATCAGGAAAAATAGCTGCTAGTGATGTTTTTTTCAAAGTCC[A/G]CTTGGCTGCACACTATGTCATGATCTCCCTAGAAAAGTGTATCAGGAGGAATTAGATTAATTTTTAAAGGCTCTTTACCCTTGAGTTTACTGTAAGAATC
8	60221694	SCAFFOLD130365_14655	0.46	0.44	GAATTAAATGAGGTAATGTGTCTGCCAGCTCCTGGCATGCAGAAGGCACCCCCTCCCCTCTTGATTCTTCCTCTGAGATTTGAAGGAACAAAAAAGAGGA[A/G]CTAAGAGATATTTTGAGGTGGAGGTGAGGGTTGATAAAGCAGCCAGGAAAGTCATCCCCTTGACCATCACCCTCCAGCGAAGTAGAAGAGATTATTTGCC
8	62184906	SCAFFOLD95963_8952	0.01	0.05	CTGGTTGTCATCTTCAGGCATGCTAGGGAAATGTGTTTATTTGGTCCACACCTAAGGACATTCAGACATAAATAGCCAAAGATGACAAATTAGGAAGGGC[A/G]TTCTAGATGAAAACCAACACCCTAGAGCATTTCTTATGACAGGTGAGACAAAAGCTGTTGACTGGTTTCTACCCAGAATGCTCTTCCTTCTTGGCTTTGG

8	62198542	SCAFFOLD290559_11486	0.13	0.05	GCTTGAAAGGCAAATTGCCAGCTCTAGTCTATCAGGCTGGGGACATGGTTTAAAGCTAATACTGGGCACCAAGACCAAATCAGGGCACCCAGACCTTGCA[A/C]CTTACGGAGAAAAGAGGGGTGGCTTTGGAAATGTAGATTTTCTTCTTTCGGAAGGAGAGAAGTAAGAATTTGAGTCATTCCTGGAATTGGGGGCTTTGAT
8	63495631	SCAFFOLD210634_2319	0.34	0.16	TGCCCTCCCCATTTAAACATTCTCCTCATACTCTGCCTTATACAGTTAATAACGAGCAGGAATCATAAAGGTGTGTGTCAGGGGCCTGCTATAAGACTCA[A/G]TTAGGTAAGTGTTATCCTAGCAGAATAAACAAAGTGCTGCAGGAGAACCAAAGAGGGAGAGATTTAACCTTCTTAAAGAATCTGGAAAAGTTTCATATAG
8	63500379	SCAFFOLD77761_2493	0.12	0.09	AAAAAAATTTAGCAGCTTGTGACAAGAATGACTATTTCAGCACATAAAGTCTGAGATAATTTCACCAGCTCCCATTCTTCCGACAGTCCATCCAACCTGG[A/C]TTCTTCTCTTTCATCATTTACTTTTTCTTCCTGTCTCTTTCCGTCTTTCTCTCAATCTCCCTTTCAAGGCATTTTTATATTCTCATGGTTCTTCCTCACA
8	65569795	SCAFFOLD15926_5127	0.12	0.13	GATTTGAGTTCTAGATTTGCGCAAGTCTTTCTCTGCCCTTGGCCTCAGTTTCCTCCTGAAGGGAGTGCGTGTGCACTGGCTGATCTCTGAGCATCCTTTC[A/C]AACCATGAGTGTCTGAATCTCTGCGTTACTGAGTTAAAGTGAAACCCGTGTGATTCAAATCCACCCTGATTAGGAGCCTGCTTTTGTAGTGAGCCCACAC
8	67316102	SCAFFOLD171260_4931	0.43	0.47	CATGTAGCCACACAGGTTTCCCCTGAACCTCATGTTTTCTTCATTATTAGTCTGGGTCTTTTTAAAATATAAATGTTCACAATTGTTTCCTGTATTTTAA[A/G]TTTATAGAATCTAAAACAGGGGACTGTGTTTATCTGATGATCTCTCTGGTCCCTTCTCTCCAAATAGTGAATCCTAAGTGGCCTTTGAACTTGGCCAGGC
8	69997377	SCAFFOLD150048_24355	0.47	0.41	ACACTTAAGGACTTGCTTAGGTACCTTACATATATGAGTATTGATAGATACTAAATGCTTTTTGTTTGAATCAGGATAAATAGAACTGAATTTGTTTTAG[A/G]TTGTCTAATTTCTCTACCATTACAGGGGGGCTAAACACATTCAAATCACTTGTTTTACAACTTGGACATACATGCATAGGCAGATCTATCTATGTATTTC
8	70299523	SCAFFOLD125856_2734	0.47	0.48	TACTCTATTTAAAAAAATGAACAACAAGCAAATGACCTTACCACCATTCTTTTGCCTTCTGAAATTGCTGTTATTAAAACTGAGGCTCACAATAAAAAGA[C/G]TGCACCTCACTTATCCAGGGAGATGCCTGATAAACAAGGGCAGCAGCAGAAGCTTGAAAGTTGTGGCACATGTGAATGAAATCCACTATCTTTTGGCAAA
8	70303317	SCAFFOLD125856_6108	0.48	0.48	GCTCTGCATAAAAGATTGGGCTGTGTCACACAGTGAAAGAAATGCTTTTCAGTAAAAGGTCACCATTTGTTTGATATGGAAACCTTCTCTGAATTTTCAT[A/C]GATACCCCAACTATCTAATTTTCACTCCTGGGGGGTTTGTTGTTCTATGAGGGTGAGATTTAAAGTAGAAAGTATGGCTCTGGTATCTATTACCAGAATT
8	71382489	SCAFFOLD120335_19914	0.22	0.29	TGAACCGTTTATCTCAACTTGCCCCTGGCAGCAAGCCACTCTTCTGATGGTATTCCCAACTAGGATTACCAAGCTCTCAGTGCTGATTTTGATGGGCCAT[A/C]AGACTTGATACACTGTTGTCTTCATAACTGTGACCTTTGACGTACTACAGACCTATTTATCCAATTTTTATAACGTTGCTAGCCCTGCCAACGGACATGC
8	71408727	SCAFFOLD100926_5864	0.16	0.17	TGCCATGACCTGTCTGGGAGAAGATTTTGTGTGAGACCTAGAACTTCTGAACCAGAAGCTCTGTGGGCAAGTGGTCCTAGGAAGCCAAAGGTGTAACTAA[C/G]CTCTGGCCATTCTTATCCATCAACCTGATGCTGCTCCTTAGACTGGAGTTTAGAAGGCATGTGGCAGAAGAAGGGAAGGAAAAATGAAGAAAAGGAAGGG
8	71438556	SCAFFOLD152030_4000	0.06	0.24	CACATGCTCATGACTAAACTATTCACTGTCAAAGGGAATGAGGATGATCCAATCAGCTTGCATGATGCCCAAATGGTCAGGTGATCCCCAGTGCTCCCCA[C/G]CTTCTTGTATTCTTACCCCTGTATAATCCCCTCCCCTTGAGTGTGGGTGGGATATGTGACCTTCTTCTACCAAAAGAATATGGCCAAAGTATGTGATGTC

8	71438630	SCAFFOLD152030_4074	0.07	0.24	GGTCAGGTGATCCCCAGTGCTCCCCAGCTTCTTGTATTCTTACCCCTGTATAATCCCCTCCCCTTGAGTGTGGGTGGGATATGTGACCTTCTTCTACCAA[A/C]AGAATATGGCCAAAGTATGTGATGTCACTTTTATATGGAAAAGGTTATATGTTGTCATTGTTATAATTATATTAGATCACATTGTATTAATATAAGATGC
8	71524097	SCAFFOLD235063_48565	0.23	0.37	CCACCCTTGTGACCTCATTTAACCTTAATGACCTCCTTAAAGACCCATCTCCAATATGAACGGAAGGGGGCACAGACATTTGGTCCATTACAGGTTGGTT[A/G]GCCAGGCACTGCAGCCCCAGGTGGCTTCCCAGATGGCTCAGTGGGTAAAGAATTGGCCTGCAGTGCAGGAGACCCAGGAGACAAGGTTTGATCCCTGGGT
8	81562137	SCAFFOLD148863_1059	0.06	0.11	GATTCTGGCAAAAAAAGGAAGGGTGGAATAGATGAAATATGAAAGGCAAAATGTTGATAACTATTTCATCTAGGTCCTAGATATGTGATGGAGAAAAGTT[A/G]GTCATTTTACTCTGTTCTTTATTTGCACATGCTTGACATTTTTCCACAATAAAAGTTATATTCATAGAAATAAGCTCAATTTTCTCATTTAAATATTTGA
8	81577887	SCAFFOLD140407_3027	0.30	0.41	TTTAATTCCCATACTACTGTAATGATGTAATTAAATTTTTAAAAATATGTACACTGGTGGAAAGTGAGTGCAAAGGAAAGAGTTCTCATGTAAACAGAAT[A/G]GAATCCTTTGAAAAAAATTAAGTGAACTGGGTTTCTTGTAATTGCTGATTTTAAAGATGAGAAAAAGAACACATAAAAATCTAGAAATACACTGCATTGT
8	81724490	BES3_Contig328_1070	0.12	0.19	GGATTTCTTACTTATATACCCCTTAGGAATGCTGCCATTTTGAGAGATGTCCCTTGGCAACATATTTCTCTGTTTAAATGGACTTCAGGCAAAAATGTAT[A/G]TGATAGATAAGGAAGATGAAAATGCTTTTGCTCATCAGCATTTTTCTTTAGGCTCAGCAAAGAGACTGTTGGACGGTTCAGATAAGTTGAGAATGAGCTT
8	82397504	BES3_Contig415_1312	0.32	0.39	TCATGCCTTCTAAGCAATTCCTGTTCATTGTCTCTGTACCCATAGAAAATACTGAACAAATTAGAGGCCCTAGGAGATCACACCTGATTCCAATCCTAGT[A/G]ATGAGATACATTATCTGCACTCATTTGGTAGCTACAAACTCTACTTTTCAATTGCCGGCACACAGTCTGAATGCAAGGAGCAGAATGCAATCAAGCACAA
8	82397771	BES3_Contig415_1045	0.31	0.39	GTTAGCATTTATTTTGGTAGCAAAGTTGGTTATAGGGAAAATTGTAAAATAGGAGAAGCATACCAGGGTCAGTCTTCTAGTTCTAAACCATCCACCTATT[A/G]TGTGTTCATCCCATCCATACAAAGAAGCCTGTTTCCTTTCCCTTGTCGAGCCTGATAGCAGATCTTTCCTAAAGGATAGTCTTTCTCAGTGTCTGTCCAG
8	84685819	SCAFFOLD246795_1485	0.30	0.37	CCAGACAAAACAGAAAGACATTGTATCCAAGAATGAGAAGCCTTTAGGAAAGAATAATAGTGACTAGGAAATTCTGTTATCACTCAAGGAGGGGGAAAAT[A/G]TCTTAGGCAAGCCCGAGGATTCTAACAGTTAACTTTAGAATTTGTCCATATAGTTAAACCTCATTCCAAATGCCACTTTTCTAAAAAGCCAGCTTAGTGG
8	86673875	SCAFFOLD125499_6290	0.02	0.17	GCTATGTTCTGTGCTACAGTTTGTTCCTTTGTGTGATCCAGGGATGAGGTGGGAGAGAAATGAGCTGATTATCGAGGGCACAGATGAATCCACCAGTGCA[A/G]AGAAGACGAGACTCCTGGATCAACTGCACTGTGGGGATAAGCGGGTGGGACTGTGGCCCGGCAGGCCCTCTGGAAACTTAATACTGCCTGGGACGGAAGG
8	87226343	SCAFFOLD65204_40210	0.43	0.36	AGTATAGCTCTGAGAAGGAACAAGAAGCCCTGATATTCACCCCAGGAGTCCCCAGGAGTCTGCAGCCTTCTGAAGTCATTCAAACCCCCAGCGGGCACAA[A/G]GGGCACTGAGGCTCAACCAGTGTGCACCACAACCATGCTGTTAAATGCAGAAATACTGTCGTATGGTAGGCAAACGTCTGCTGTGGTGGCAGGTGTAGGG
8	87226378	SCAFFOLD65204_40175	0.45	0.34	TTCACCCCAGGAGTCCCCAGGAGTCTGCAGCCTTCTGAAGTCATTCAAACCCCCAGCGGGCACAAGGGGCACTGAGGCTCAACCAGTGTGCACCACAACC[A/G]TGCTGTTAAATGCAGAAATACTGTCGTATGGTAGGCAAACGTCTGCTGTGGTGGCAGGTGTAGGGGGCTGTAAACACCACCCCAGCCTAGCAACAACAGG

8	90157111	SCAFFOLD135002_21267	0.27	0.24	CATTTTAAACTATGAACAAATTTAGTCAGTTTGTAGGCATATCTTTAAGATTCCTTTAATTCAACTTTCTGCTCAAGTCATACACCAATCTTGGTGAGAA[A/T]GAGCCAGTTTCTCTTATTATTAATAACTCGCACCTTCCCATTTTGCACAAGACTAAATAAATCTTGGCCTCAATTCTCCTGGGACTGTTACTGCTGCTCA
8	91886012	SCAFFOLD155145_9683	0.28	0.36	ATGTCTTGCGTGCATTATCTCCTCTGCAGCTGAGGCTTATGTACAAGATAGGCTGCCAAAGGCTGCCTCTCTTTCAGGCCAAAATCCTCAAGGCAATTGC[A/C]AAGCCTTGATATCTGCTAGAAATATGGAGCAGATACTTGAGGAAAATAGAATAGATATTTAAAGTCAAATTGATGCCTCCAATTTCTCAGCACTTGTTCA
8	91886103	SCAFFOLD155145_9774	0.10	0.18	GCAGAAGAAATCGCAAATGCTGTTGATATTGTAGAATTCCTAGGCCCCTCTCTGCTTCTCAGCTGGTCTCTCTCTCCCTCACCCATACAGAATGTCTTGC[A/G]TGCATTATCTCCTCTGCAGCTGAGGCTTATGTACAAGATAGGCTGCCAAAGGCTGCCTCTCTTTCAGGCCAAAATCCTCAAGGCAATTGCCAAGCCTTGA
8	91886326	SCAFFOLD155145_9997	0.12	0.22	ATCCTAGAGATGCTGATGCTATGGTGGTCTCAGGGGGTCAGGAGCTCTAAGAATGGGGTTCCCAGGCTCAGCAAAGAGCCCTCTTTATAATAGCTATATT[A/G]CTTCTCATCCTGCTCAGAGTGCCAATTGTCTTGCTGTTCACACTGTCTGCACTGCTAACTGAACTCAAGTTAGACAAGACATGAGTTAAGTGGCTGAAAG
8	92660358	SCAFFOLD151532_1045	0.39	0.38	CAGAGCACATTGCTTTTTATGATATGATAAGGCATCATGTGGTTCTCAGATTCCATGGAATTCTTGTTCTAAGTACCCATCCCTTTCCATCATCGCCCTC[A/G]TTCTTGGATTGGTCCAATGCACAGATTCGGGGAAGAAATCTTGTTGTCAGTGTCCTTGCAGTTTCCGTCTCTTAGCAACAGCCCATGCTCCTGTTCCTCT
8	93816004	BES11_Contig368_702	0.47	0.40	CAACTATCTTAACAGAGTTTGCCTCAGTAAGCTTCATCACCCCCACCTCAGTCAGCCAGGACCCCTCCACACTGGGCATGCCACAGATGCACCATGTCAC[A/G]TCAACTCCATGACAGTCTCAACCGCGAGAGCCTCCAAAATAATGACCCACTAAGAAATTAAAAATGCTATCAATAAAACCACAGCATGTGACCATTTCAA
8	95943521	BES7_Contig509_1173	0.41	0.26	GGAAGTTGACAAGTTGACGAACACCAGACTTAATTTTGCCCATCAATCTAAATTAATATACTGCACAATTACTTATACTTAACTCTAGGCACTGTTGAGA[A/G]GAATTAAATAGGTCTCTGCCCTTATAAATTCATAGTTGAACTCAGCTGGCACAAAGAGAAAACAAAAGCTACCTTCAGATTTTACATGCCCTAAGAATTT
8	97335528	BES11_Contig229_940	0.14	0.25	TGTAGCAATTTAATTCGCTCATATGGGAGGAATAACAACATAAATCAACAAAGCAAATGTGTACTAAAGCAAGTGCAAACAGCAATTATGAATGATGAAA[A/G]TAATGACATTGGATTTAAAGCTACTTATCTTTATAATGACCTTTCTTATAGGAATTCTAACACCTCCCATTGAGCTTGAATGGTAGCAAAGTCATCCTTG
8	100628165	SCAFFOLD130145_6305	0.36	0.22	GAAATGGGTGGATGGCAGAGGCTATGTGTGTTCAAATGATGCCAGGAGCTCTCCGTATAGAAAAAGTACAGAGCAGGACTATGGATCGTGTGGCTAATTG[A/T]GTGTGCAACTCCTAGGTTAACTGGCTGTAGGTGGCACCAGCAGCATGACCAATGAATCTGTGGCATTTCACCCATTATGAATGTAATCTTGGCCTTGGTA
8	100628707	SCAFFOLD130145_6847	0.47	0.37	ACAAAATGTATAAATGATATGTAGAGTTTCTCCTTTAACATATGAACTCGGGGGAAGATGCTGAATTAAAGGGAAGATGCCTTGGTGCAAAATAAAGTGA[A/G]TTAGGCTGCCAGCAGAAGTGCTAACAGGGAGACATTTTATAATTTATCACGTTTTAAGTGGTTGTCTCTGTCTCACCAATAAACAGCAGAGTTATTCATT
8	101581819	SCAFFOLD190179_31876	0.49	0.02	GATGGTGATGGTGATTGAATGCTAAGTGCCAGGCTTTGTGCTATGTGGTTTACAGATTCAATTTCATTAAATCAACACAGTAGTCCTGAGAGATTTAAAA[A/T]TTTTCAATCTCCGTTTTGCAAAGGAGGAAACTGAGGCTTGGGAAGGTTCAATGATAGTCTACAGGACACATGACTTGAGAGAGTGGAACAGAATTTTGGG

8	102868910	BES1_Contig640_2297	0.31	0.23	AGACCAGCCATGCCCAGCAGCCTCCCGAGACACTACCCAAACTTTCTTAATATTGGGTTCATCACAAAGACATTAAGTTAATCAACTCTTTTTTTCCAAA[C/G]TGGAGCCACTGTGATCAAGATTGAAAATGTGCCCCCATTGTAAGTGGCTACTGTTTCCAATTACATATGTTTCTCGCAGCCACCATGCAATCTGTTCACT
8	103345433	SCAFFOLD111258_4504	0.26	0.44	AATGTTTCCAAACTGATTTCATAACCAGATGATGCCTAAGATTTAAACTTGGCAACCACAGGTCTATTCTTCAGATTGTGAAACCAATATTCAAATAGCT[A/G]ATGAAGTGGATTCCCCATCGAATGACACAGGTTGCATTGAAGATGCCGACCACAGATTTGTCATGGACACTTCATGATGTCTCACTCAGCAGTAGGGGTA
8	103345581	SCAFFOLD111258_4652	0.11	0.21	GACCACAGATTTGTCATGGACACTTCATGATGTCTCACTCAGCAGTAGGGGTACAAAACCACTGAGAGGATTCTGCTTATGGAGCACAAATTTCAGTGCC[A/G]GGCACACAGTAGGCAATCAATAGCTATTTGTTAAACGAATGAATCAATAGCTATTTGTTAAGTGAATGAAGGAATTTATAACATACTCTCTTTCCAAAGT
8	104327227	SCAFFOLD290984_3799	0.33	0.33	CAAATCCTATTGGGATTTTTTTAGTCTCTATGAGGACACAAGGTTTTTCTACTGCTCCATATTTCATGCTATTTGATTGTCCCTTCATCAAAGGTGACAC[A/G]CAGCCAGGTAATAAACCTTCCAAAGAGTGGCTGCATTAATACTCTGGGTGTGTTCCTACTGAGCCATGGCAGCCACCTGGACCTCCACCAAACACCTCCA
8	104683168	SCAFFOLD120208_7951	0.31	0.29	TTCCAGGCAGGAAGAATAGCAAGAGCCAAGATACTTAGGTGTGCAATGGCAAACTATTGATACAGAACTGTAAACACTCTGGTTTTCCTGGTGTGTAAAG[A/G]GTAACAAGAAATAAGACTAGAGAAATGGGTGGAGGATGACGTCACAGATCATACAGGCCTTGAATGCCCAGGATTGGGTTTTATTCTTAGGGCAATGGGC
8	104885923	SCAFFOLD155358_14286	0.03	0.04	CAGTGGTCATATACTCAGCGTCTTCAAGGGCCAGGCAGGGACAATAGATGAGATAGTAGAAGTGAGGAGAATGGTGGTCCCTGGAGAATCACCCTCCCAA[A/G]TGGTCTTTGTTCATCTTCATAAAAACGTTAAAATGTATGACATCAGATTTCAAATACATTATTTTAATGCATTTGAATGTGGTTATTTAAACAATGTATT
8	104888840	SCAFFOLD155358_17055	0.04	0.04	GATATCATTTGAAAACCTGTGGAAAAGATTTGTAATGCTGGTTCCAACTGACAAAAAATGTCAATCTTCTGTGCAAAAGACAAAGGCATCAAAAAAAATG[A/G]GGTGGGAGAGATATATAGACACAGTATTATAAATCTTTTGGGCTTCCCCAATGATCCAGCTGGTAAAGAACCCACTTGCCAATACAGGAGATACAAGAGA
8	107820671	BES8_Contig471_1044	0.09	0.14	TCCAGATTCTTTGTCACTGTCTCTCATGTGAGTGTCACCTTCAAACAGCACCTGATGGCTTACTCACCTCCAGCCGTATTGATCACCAAACCAGAAAACA[A/G]TAGTTCCACGTTCTTAAAGCCAAAGTAGAAGGTCATAGGCTGCCAGGAAACCAAAACATGTCAGTTAGAAACAATCGAAGTTTCTACATGAGAATCTCAG
8	107821115	BES8_Contig471_600	0.09	0.14	CTTGGACGGAAGACTATGGTCAAGTTTGAATGGCATAAGAAGCATTTTATATATACATACCACAAAATAACCCTAGCTTCAAAACTATTAATCGAGTACA[A/G]TCCTTCCATTTTACAGAAAAATTAAGCTTCAAATAGTGAAATGATTAGCCCTAAATAAATTAGTATGTCAGAACTAGGACGGGGACCTAAGGCTTCTGAC
8	107840541	SCAFFOLD221622_3349	0.32	0.38	TACCTTTCGTGCTCAAATTTTCTGTAATGATTACCTGCCTCAAATATCACTAGTTTATGTCCAAAAACTACAAATAATAAGCTTGCACCTATCGAAGTCC[A/G]TATTCTCTGCCTGCCTGACTTGGGATTATCATCCTGAGAACCTTTTTCTTAAACCTTTTATACAGTTTTTCTTCAGTGAATTGTTAGTAATTTTACTAGG
8	107890604	SCAFFOLD241182_8269	0.40	0.48	TCAGATATTATTTCTGTTTCGGTGACTGAACACTCAGAATTCTTCAGGAACCTTCTTTAATAATCATTGTTCAGGAAGTCAGTGATAATCAGGACTTGTT[A/C]GATACTGTGAGGATACCAAAGATGTTCACTGCATCACAATACTTGCAGGTAGAAATGCTGCTATTTTAAAGAAAACACCCTTTTCCAGGTTATTTTTAAC

8	107893513	SCAFFOLD241182_10732	0.26	0.24	GTGACTTTGTGTTTTTCCATGTCCTCCTGAGGGGAGGGTGATGTGGAGCTGATGCTCTTGGCTTAGGTTCCCTTGGAAGATATGTCAGTGTTAGAAGTCA[A/G]TTTAAGTCAAAATCAGGTGCAGAGATTAGAAATACAGACTCAAATGTGGTTCTCAATTCAAATTAAATCCAAGTACAATTTTTAGTGAGCGTTGGGGACT
8	108801251	BES2_Contig467_1067	0.19	0.19	GAGGATGGGGTCCTTCTTTAAAGGAGTGGACGAGACTGCTACCTTCCGACCTGAGCTACGAGCCCAGAGCTTATGCTTTCAGGAGGCGCCGGGCTGCAGA[A/G]GAGGGACTAAGGCCGGTCATCCACTCATCAGCCTCCAGTGCCAAGGGCCCTGGACCTCAACACTGAAGGTTATGTTTCTCCAACTTCCTTTTCAGTGATC
8	109814329	SCAFFOLD165033_11046	0.25	0.32	CAGGGCGACTACCTAGGAAGCACCTCAGTTTTAGCAAAAGCATACATAGTTTATTCTGAATCTTTAAAGCTACTGAGCTGTTCACAAGTAGTCTAACTTC[A/G]GGCATGTGCCACCGTGCATGTTAAAGCTTTTGTCCGTGAGGACATAGTTACGGGAAGAATGATACAGCATCATAATGCTCTGTTTCTGAAAGTAGCAGCT
8	110894109	SCAFFOLD95889_1935	0.22	0.38	AGTGTCGGATGTGGCGGTCGGCTTTTGTGCGGCTGACAACTCGTCCCTTTAGAGGCCGGGCCGGGCTCATTTCACGGGGGCCCTCCACAGGCCGGCTCCC[A/G]GCTCCGTAATTACGCCATTCACCAGTCACCCAGGCCCGCCTCTGAACTCCTGACCCGGCACTGAAAGACTTGGGATCTCATTACTAATCTCTTAAGCCAC
8	112436215	AY297040-1656	0.25	0.28	CACAAACACAAGCAAATGCATTCTGAGTAAGATTTAGCCAAGTGAGGCTCCTTGGCAAATTCTGTAGTTCTTGCTCCTTAGAGGCCATGATACGGTTGAA[A/G]CTGCAGTCTAGGATCCGGAGCGAGTGGAGTGGTTCATAAAGAAATGTATCCAGTGACAAGAGTTTGTTGTGACTCATATTCAGCACCTGAAGGCTAGAGA
8	112436215	SCAFFOLD317637_26927	0.25	0.29	CACAAACACAAGCAAATGCATTCTGAGTAAGATTTAGCCAAGTGAGGCTCCTTGGCAAATTCTGTAGTTCTTGCTCCTTAGAGGCCATGATACGGTTGAA[A/G]CTGCAGTCTAGGATCCGGAGCGAGTGGAGTGGTTCATAAAGAAATGTATCCAGTGACAAGAGTTTGTTGTGACTCATATTCAGCACCTGAAGGCTAGAGA
8	113240717	BES10_Contig543_926	0.38	0.41	CATTCATTAGCTAGCAGCCAACCCATGCATCTTCATAACGAAGTAGTGTCAGCACTGCTTGGCTGGGAGGGTCTCATTTAGGGGCTGCACAGGTCCCTCC[A/T]GCCATGGTCACTGCAGAGAGTTAGATAGAGGGCCTTAGGGAGCCCCAGAGGTAGTGTGCCATTCCATCATCTTTGTTCCAGGAAAGAGCAATGGAGCTTC
8	114251249	SCAFFOLD245097_12780	0.32	0.46	GCAGTTCTGAAATCAGTCTGAAACACCTAGTTAGGGAGCTTCAGAGAAGGACCTTCCAAGAAGGACCAAGGTACCGACTAATTCCTTTTAATTTTTCTGT[A/C]TTAATCAAACTCCCAAGATGGGCTGTGGTCCAGATGACTTAGGAAAAGGGGACCTAAACTATGGGACATTTTCCTATCATCCTCTCTTGTACCAAGCACA
8	115053578	BES9_Contig546_2551	0.48	0.49	GTCCCCGTCCAGCTTGTGTGTTTCATCTTTATGATCAATTATTTACAATATCTGAACACTGCCGAGGGGTCGTCTGGGAGCTTTTCTTTCTAGATAGCGG[A/G]AAAAAAAATGCAATAACTCAAATCATATGAAGCCACCAGCTCTGGAATGATCATTCCCTGAATCATTTATTTGACAAACATTTACTGAGTGTTTGCTAGA
8	115054054	BES9_Contig546_2075	0.21	0.21	GGGACAATTCTTTAGAATTAAGGATCCTTTCCAATCTCATTTCCCTTCATTGCCGCAGCCATGTTTTTGGCCTCACCGGCCTGAAGGATGGGGAGATGGG[A/G]CCTGCCAAGACTAGCCAGGGAAGCCCTGGGGAAAGTGAACAGCTTCAAGGCTAGAGCAGGTAGCAGAAAAAGCCCTGGGCTTAGACAACAATACTTACTG
8	115096125	SCAFFOLD60719_8437	0.15	0.18	CTTATGACATAAAATGAGTTGAGATGGGTTTATATTCTCTACAAGTGTTTGTGTAAACTGGCATCACCCCTTAAGTATTGAGTGGAGCTCGTTAGTGAAG[C/G]CCCTTGGGTGGCTGGAGTTTTCTCCATGGGAAGGTTTCCGTTATGAATCCAATCTCTTTGATGGGTGTGAGACAGTGGTAACATGCTTTGTCCTCTTCTG

8	115245018	SCAFFOLD94178_2657	0.17	0.19	TTTTTTTAGGGAGCGGGCCCATCAGCTTTCATCGGGTTGTTCTAGAGGGGTCCACAGCCCCAAATGCTAAGGAGACGTCGCTCTAAGATACGAGCGCCAC[A/G]GACTCAAGTCCCTCTTCTCCTCCAGCGACTCCCTCCAGCTCCGTCCATCGAACTGCACGAAGAAATCAACATCTATTGGAAGGAAAATAAACCATCATGT
8	115371950	BES4_Contig450_2173	0.46	0.06	GAAACCAGAAAAAGTCACCCAACACAGGTCGCAGAGTTCAGTTTCTCTCCCATCTTACTCCTACACTGAACAGGCCTACAGCTCCGGTCCCTACTAGCCT[A/G]TAAATGCTTTAGGAAGGGGCTGGGTACATGACTAGAGCTGTAATAAGCCCTTGTTGATTGATTGACTACATACCGAAAGCAGGCCAACAATTTGCAAAGA
8	115426852	SCAFFOLD5002_20230	0.29	0.31	AGAGAAAGCCCGGGTGCCCAACAAAGACCCAGCACAACCCAAAGTAAAATTTCTGTACCTTATTCTACCCAATTAAGGGAGTCCTTACACTGTTAGTTTT[C/G]AACTAACAGTTGGGCTATATGAGGTATTTAGCAATGTCAGAAATGATAACAAAAAATAACCAATTTTCATTACTACTCCCCCAGACTGATCAATCATGTC
8	115907050	SCAFFOLD20777_7940	0.16	0.10	CAAAGATAAAATTTGGTAATCAAAAAGTGTAGCTCCTTCTTTGGTAATACTAGGTATTATTCTGTACTTTAGAAATGCCAAAACATGACAATTATCCTAC[A/G]TGAAGGGCTAGACTACAAGTGAGTAACCAAAGTGCTGGCTCTTCCAAAGGCTTCTTGCAGTAACACGGGCAGTGTGGACCCACCTCATTACCAAAGACAA
8	115941562	SCAFFOLD80021_27341	0.33	0.40	TGATTGACGTGTTCTTATACAGAGGTTCATTTAGATTCACAGTATTTAGTTTTTGGCAAGAAGAAGGTAGTGTCCATAGGCACTTCCTTTTGCGTCACCA[A/G]GACACACATCATCATGCCTGTTTGTGTCTTTTGTGACTTTGGGTTAATCAGTAGATTCAGATATTGTCAGCCATAGTGGAAAAGGTTTTTAAAAAATTGG
8	116028292	SCAFFOLD45904_7761	0.47	0.43	CATCCTACCAGCCAAGTATCCCTTCTCTCAAGACACGAATTCTTGGAAGAGTCATTTGGGTCAGACTGTGGTGGAGCTAGGGCCTCTTTAGGGGGTTGTG[A/G]ATGGGACAGACAGTCCTGCTGGGACCTCATGGGGATTGAGGAGAGAGGCAGACACTCCAAAGAGACAGGGAAGGGACTGCTACAAAAGATGCGAGAGGAA
9	2289236	SCAFFOLD50340_7682	0.25	0.38	GTTCTCTGTGATCTATTGTCTTCCTGCTGTCTACTCTGGCTTTCTTCCTATAAGGAAATTGAGAATTGTCTCCAGCTCCCTACAGCTTGAAAGTCAAAGT[A/G]ATAGCCAATTATGTGACTTTAGCCTTATGGGAGCATATGGATTTTGTGGAGAATTTTTTTTTAATTAAAATCTTTGAGCTGTTAACTCATAAAAGAAAAT
9	8499320	SCAFFOLD149286_883	0.42	0.40	CTGATCAACCTCCATCAGGTCGGGGATAGTGACCACAGAGAAACAGGGTGCATGTAAACGCCAAAGGCAGTTCTCTGATTAGTGTCAGAAAAACAATACA[A/G]ACCCCGGGGTCTTGGAATCAGTGGTTGCCCTATAGCCACATCCCCTAGAGATGTGCTATTTTTCTGTTTTCCGTGCTGATACAAAGCATGGCTAGAGGAG
9	8619026	SCAFFOLD105304_2010	0.47	0.37	ACAAAAAGAATGTTTGCTTATTTTCAGAAGCTCAGTAAAACTGCCTTGTTGATGAACGTAGGCAGGAGGAAATCACATATTTTGATGGCATTTCCATCCT[A/G]AGTCCTATCCCACCTTCCCATCCTGAACAAGGTCACATTATAAAAGTGCACGTAAGGTCAGCTATGCAGATAACCCTTTTGCTTAGAAGGTTTTTGTCTT
9	9886211	SCAFFOLD200128_32745	0.48	0.43	TACTTATTTTTCCTGAGCTTGAAACTCAGATTGAACCTGCTTGAACCAGCTTGAACCTGAACTCCATTTTGTAATGATGCTATCTGTAAATGCTCTGCAT[A/G]TGCAACCTTTCCTCTATTAAAGAATAACTAAAACCAGGGGTGAATACTCTAGTGACAAGGGAGCCTACTCAGAAGGCAGTCCTGCGTGGGGAGTCAGCGC
9	13861925	BES2_Contig266_1111	0.49	0.44	CAGGGTAGGAGGTCTTTAGTTTGCTCTTTGAGGTATGAATAATTTCTCTGCCATCTATTTCACTCCACTCAAGTTACCATGTGGAATGCCATTTGGGATA[C/G]CTTGGAAATGGTTCTATGTTGTATCATAAAAAAGACTCTGACAAGTTTTGTGTATTTTGTGATGGCACAGAAAATAAGGAGAGGATTCAGGGTTTGACCT

9	14831302	SCAFFOLD175002_56192	0.26	0.37	AGGCAAGGCCTCTAATCTAAATTCAGGGAAGATCCCTCTGATATCCTGGGGGCTGTACTACAAAATTTGCCTAAATCCTTAAATATAGGAGCTTAGTGCA[A/T]GGGTTTATTTAAAACTCACAGTATAAATAGTTCTTCCCTTGAACAGCTCAGCAGCAAGGTTTTCATTACCCATTCCAGATAAAATACAGAGACTTCTTTT
9	15046353	SCAFFOLD140160_3611	0.27	0.19	AAAGGCAGGCTATTGATCACAATTACAACCCACTTCAGTTGTGCACTTAAGTCATGTTACTTGGAGTGATGCAACAATGGATGATCCATTATTGTGAACT[A/G]CCAATTATGGTTGCCTACATCACCTAAGAAACAGAAAGTAGCTGTACAGTATCCATTTGACACTAACAACAGTGTTACTTTTAGCCCCCTTTTGGGGGCT
9	15359625	SCAFFOLD155268_21318	0.25	0.39	TGTGGTGTAGGGGGCTGGGTGCTCCTAGATCCTCCCCAAGTTTTAGAATGAATTGTATGCAATATAAGAGCAGAACCCATGGACCTATTGAGCCTGGACC[A/G]AGACAGGCCTGGTGTATACATTCTTAGCATATAGAAATGGCATGCTGGGGAAGGCCAGTAGACATTCTGTGCATAAGCCTTGTGGAAGGATGAAATAGAA
9	19430616	SCAFFOLD1075_11860	0.50	0.44	ATCTGGGAGTGGTTTCCCTTGTGCAAGTTCTCTTAATTTTCTCCACCGTAATGCTGGCCTCGCCTCCGCTCAGCTTGTGAAAGAGGAATCGTCATTGGGA[A/G]GTGGTGACGCATGTGCCTGGGCAGAAGGGAAATGAACAGCGATGAGTCATTTGCTCACAGGCTACTTCAACAGACACTGAACATCTCCTAGGAACTTGGA
9	19432239	SCAFFOLD1075_13483	0.47	0.36	TAGACTTTTTGATGATGGCCATTCTGACTAGTGAGGTGATACCTTATGGTAATTTTGGCTTGCATTCCTCTAATAATTAGTGACATCAATAATTTTTCTT[C/G]TGCTCTGTATTTTCACTTTGCCCTGGACCCCACAAATTCTGTAGCCTGTCATGTTGATGAAGGAAGTTTACTTATAAACTAAACTATTGCAAGCATCCCT
9	22784033	SCAFFOLD265193_16592	0.31	0.39	CAATTTAGACCAAGAGGATCAGGGCTGCCTACCGGGGACAGAGGAGCAATGAGCTAGGAGTATCCTGAGGGTTTCATAGAGCAAGGCTGGACTATCACTG[A/C]TGGACTTGAAATCTAAGAACTTGTAAGAGAGAGAAATTTACTTCTGAGTTTTCTGCCACTCACAACTAAACCCAATCCAAATGAAAGCACCCACTCTACC
9	23740648	SCAFFOLD72836_6481	0.47	0.05	GAGATGTGATCGGCTGGGCGTGCAAATACTGAAGAGAAGTCATGGGCTGATGGGGATTGATGTGCTCCAGATCAGACACCACAAAATCAAAACCTGTTTC[A/G]TTCTCTTCAGGGATAGTCATTACCCTGTGTTCGAGAACAATGAGCCTCTGAAGCACTTTGAGAAGTTGTGACTGGAGTGTGCTGCCACTGTCAAATTCAT
9	24389745	BES9_Contig132_328	0.42	0.42	AATTTTTAGTTTGTGAGTATTTTCCAAAATGAAACACTGTTATGTAAGCAATTTTCACACAGAGGCACAAAGGAATACTTCCAATGGGAAAAACAATTTT[A/G]CTGTTTCTTTCTCCTAACATTTTTATCCATTAATATGTTTACATGGTATATACATATTTAACACATGTCTAACAGCATTTTTCTGAAAAACAGGTAAACA
9	25735288	SCAFFOLD110752_5035	0.17	0.16	TATGGGTTTTCTATCTTGTTTTATATCAAGATAGATAAAGACATTAGATGGTTCTTCTGTTTTTTAATCTAATCCTGAATCTGGAGAATAGAGAATAGAG[A/G]ATGAAACAGGATTTAAAAATAAGACCAATTAAGGAAAAATTTACTCTCCAGAGGCAAGGAATGTTTTAGGGAAATAAAACAGATGAAATGTCAGTAAATA
9	27368574	SCAFFOLD32492_3890	0.18	0.27	TCCTGCAGAGCCTGTGAGTGCCGTGTCACCTCCTGCAGGTTCAGAGATGATCATGCTTGAGTCGGAGAACAGGTGGTCGTCGAGCACCACCATGGGCAGT[A/G]AGCTGAAGGTCACGAATAGCTCCTGGCCCTCATTGTCCAGGCTCAGGCTAACGAGGGCCGTGAGCGTCAGCCAGTGGTTGTAGCTGGCCTCCTTGAGGCA
9	32043039	BES4_Contig184_623	0.49	0.49	ATGAAGAAAGGCATCTTCAACCTCAAAAGGGCACTGTTTGGTCTGGTCTTCTTTCAAAGAGTAGAGAGTCCAAGATGAAAGCAAACCTGGCGCTTTTACC[A/G]CAGTCAACTGGGGGAAGATGTCATTTAATCTACTGGATAATAGTGGGAATAAGTGCCAGAGTCATTTCCTAGAATTAATGAGAAAATGGCACGCAGGCAC

9	35925301	SCAFFOLD105738_2098	0.26	0.17	CAGTTAGAACGTAGCATCACTATCATATTTATTTCCTCAGATAAAAATTACAATTGATAAAGAAGAAATTTTCAGCAATGGAAAGTTCATAAAAGATTT[A/C]GAAGAGTACCCAAACTTTAATATTTTATCTCTGAGAGCACATCTTTCTGTCTGTCTGTATGACCAGCTGTCTTTTCACAGTTTCAAATTTCCTGACCCTA
9	35925360	SCAFFOLD105738_2039	0.47	0.32	GATAGTTATGCTTGAACTATGCTTCTCGTTCACTGTATTGATGAGGTGAACCAGAAGCACAGTTAGAACGTAGCATCACTATCATATTTATTTCCTCAG[A/G]TAAAAATTACAATTGATAAAGAAGAAATTTTCAGCAATGGAAAGTTCATAAAAGATTTCGAAGAGTACCCAAACTTTAATATTTTATCTCTGAGAGCACA
9	35925590	SCAFFOLD105738_1809	0.42	0.28	AAGTGAATTTCCTCACTTGTAATTTCTGAGTGTTGGATTAGTACATCAGAGTTGTTTTCAACTCTGTTGTCCTGAGTCTTAAGGCAGTAGTCCATAAATT[C/G]CTAGTCCGTTGTCTTCTGCGTGTACTCTTGGGGATTGATGTCTGTCTCCAAGTTGCTTGGTCTCTATTTGGGCCCTTGGCCTCTTTCCCTTTCTGTACAG
9	36408876	SCAFFOLD140032_5765	0.13	0.17	TAGAGCCAGTCTGATAATTACAGAGAGATTAGTGTTATCTTATGATGACTTTGTGAAACAGTGTCAGCCATCCCTGGATAGTTGAGATGCTATACTGTTA[A/T]TAGATACCTTCAAATTTCTGGAGAATTGAAATTGTCTCACATAAATCCAAACTATCATTATCCTTGTCTGAATAAGCCAAACATTACCAAAAATGCATTG
9	36732573	SCAFFOLD111038_5817	0.49	0.50	TTAGCCCCAAGAAAAAAGAAGATATAACAGATAAAGAGGAAGATGATAGATGCTAATTTTGTCAATTTTCCCAGCAGATAGTATCACATTTTCACATGCA[A/G]GCGGGGTGAAGAAGAGCCTGGAAGATCAGAGAATAGTGACTAGCCCAGTATGAGCCAATAGTGCATAGATGTAATGGTGAATAGTAGTGAATCAGACAAA
9	37509758	SCAFFOLD135239_5109	0.23	0.35	AACATTGCCACATCAGGGGCAAACACAACCTTCTAGGTAAGAAGTGGGCAGTAGAAAGGGAAAGAATGTGCCTTTCAAGGTGTGAGTCAGAGGCAAAGAA[A/G]TAAACGAGGGCATAAATGAGCAAGGCAAGCACAGGCAGAAGTTTGGTCTTGGCAAGAAGCACAGAAATTCAAAATAGCAGAGAGACTCAATCTATTCATC
9	37510220	SCAFFOLD135239_4647	0.21	0.35	AAGCCACCTCCACTTCATTCAGGTTGCCTCTTCCTTTTCCCCTGAACACCCAATGCTCATTTCTACCTCTACTGCTTGCCTGAACCATTCTGTCCTCTCC[A/G]TGGACAACCGGCACCTTTTTCTCCCTGCATGTCCTTGAAGCCATTCTGTCTAGAGTAGCCCATGTCGTTACTCTCCTTTGTGCCTCCTCAGCACTTCAAT
9	41937039	BES5_Contig171_564	0.50	0.00	TTGTCCAGTTGTACTTTAAATTATTAACACAAACTGCAAAACTTCTGGTTTTCCGGTTTCCTTAGTTTCCTGTCTGATTTATGAGAAATGTTCTATTGGT[A/G]CATATTGTGATTTACTTTGCCAGTATTTTTACACATGCTGTATTTTCTTAATGTAAGAAGAAACAATTTTAGTAAAATAATTTCTATTCTGATTGCCTAA
9	42911335	SCAFFOLD135437_5344	0.32	0.22	TTCATTGTCAACCTTAGATCTCAGTTTTATTTATTAAAGAAAGCAATGCCATTTTCCTTCATCAGTTACATCAGTGACCTTTTTCATTGTTGGGGAATGG[A/G]AAAATGAAAGCCCAGTATTTGCATTCATTTGTTCAGTTGACGAGGATTCGATCCCTGGATGGGGAAGATCCCCTGGAGAAGGAAATGGCAACCCACTCCG
9	44568721	SCAFFOLD225040_14973	0.22	0.24	TATATCATGAATATTGTAATATATGTAAATTATAGCATTAAATGCAAACTACTTTGGCATTGCAGTTTTTTTGCAACTAAAAATAAGTTGTCTTCTGTCT[A/C]AATCAGATTTCTCAGTATCTCTAACATTTGAAACTAAATTTTCATGTACCATAAAATTACTGTTACAATTAGACTTTGTTAAAGGAAATACTGCCCAGTA
9	45051969	SCAFFOLD94465_1515	0.45	0.48	TCAGATCAGGCAATTTCCTTCCACTGGGGGTAACATCCTAGGGAGGGACTCGGCTGTAAGCCATCAGCTTCTGTTTCAACAGCTGGGGCTTTGGGGCAGT[A/G]AGAGCCTCTGTGCTGAAGGGACAGCTGGGCAGGAGACCACAGCACCCCTTACCTGTGGCTGTGTTGTCTGAGCTTGGGAAATCTGGGGGCCCAGAGATAA

9	45052194	SCAFFOLD94465_1290	0.45	0.48	TTATGGCAGGGCAGCCTTAGGAAACAGATACAGTTGGGAAGACAAAGAGAGAGAGGTAGGACTGGGCAGTGAACTTGAACTGTGATGCGGCTGCCAAAGC[A/G]GCCTCAGCCCCTGGGAAGCTTCGGAGTTGGAAAGACCCTACAGAAATGTCTCAGATGGAGGCAGAGGGCCTTTGTGCCTCCGGCATCCATTCATCATTGG
9	47307855	SCAFFOLD116602_3801	0.38	0.45	ATTGTGGTTAGTTTCTCCCTGGTTAGTTTCTTCTGACAAAGTTAATCAACTATATGTGTACATATATAGAGAGAGCTCCCTCTGAGAGCTCCCTTCCACC[A/G]CGCTCCCTATTCTTTTGTTTTTAAGTGAATAATAAGGCTCTGTCTCTACTTTAGAAACTTCATGTGTGTGTGTGTGTGTATGTGTGTGTGTGTGTGTATG
9	47704352	BES9_Contig552_847	0.21	0.14	CTTGCGAGTTGCCTTCTTTGTCCATATTTTTTGAAATATGTTAAGGATGCTGGGCCTTAGCCACTAAGGTTCCAAATCTCTTCAGTTATCCAAGAATGCA[A/G]TTCAGACTAATGAGTGGCAGTGAGGGGTGTGTGTGTGTGTGTGTGTGTCTGTGTGTGTGTGCGCACACGCGCGCACGCTGCCACTGCCCTGGGGCTGTTT
9	47705809	BES2_Contig457_911	0.21	0.14	GGAACAAATCATTCACTCACCTCCGTATGGAACACATGATGCATTTTGATCCAATCTATTTTACATTTGCAGTGAGGACATGTGTGAGTTTGAAAAGACT[A/G]GGCCAACTGCTAAACTGTTGCTGGGTGGGCTTCGCAGGCCTCTCCTGAAACATACTGTACTGCATATGGCTTAGAACGAATAACCAAATGTCTTAAACTT
9	48193040	BES9_Contig458_891	0.07	0.16	ATGTCAACTTTCTTGGAAACACTCCAAGCCAATACAATATGATACTTTTATTTTCATGGTTAAATCCTGACAACTGGACAACATGGAAAAAATATGTATT[A/G]TGCCAAATAGGGCTATAAAATAAAAATATTGAAAGTCTGTGATGATTTGGATCATATTTAAGTACCCTGGAATATCTTCAAATTAATGTTTCTTAATCTA
9	51438990	BES3_Contig258_1395	0.04	0.02	TTTAAAGATTTTTCAGAAGTTTAAATTTTGCTTGAGTTTAGATCAATTAAAAAACAAATAAAATGGTAAAAGTTTATCTGTAGTAAAATGAAAATTCATA[A/G]TGCTTTTTGTAACATTAAGCAAAATATTTTAATTGCATGTAACAGTTACATTAAAACATTTTTGCTTCCTTCTTACCTATTTCATCTTTCCTCATATCTT
9	51593826	AF440366-255	0.08	0.18	TAGGCRATACAAGATTCTCTTCAAGTCTGGAGGAACTGCTCAATGCAGGGGGCTTAGAGGGCCCTACCTGGGTCCTTTTCTCATCTCTGCCCTGCAAGCT[A/C]CCCATGTGTTCTTTACCCCAAGGTGTCGGCCATCACCCATCCCATGGCTATCAATCTTTCTTCCTGGCTTTGAG
9	51639576	AF440371-949	0.31	0.42	AAAATCCCATTCAAAGCAGAACAGAGAACTGTTCAACATCTTTCCAACAGGACAGAAGTCAACCTGGGAACAGCTGTTCTCTCTGCGGCTCAGGTAGTGA[A/G]ATGCACAGGACCTGTCCTGGGTACTGTCCCTGTGAGTATTCAAGCTGGACTCATTAGCTGCAGTTACTTTGCTGCCAGTCATGATTCCATACATACACAC
9	51731023	SCAFFOLD225626_1260	0.15	0.14	ATTGATAGCCCAAGCTTTCAATTCAAACATTTCAATACATGGACTAAGGGAGATTATATATGTGTCTTGTGTTTCATGGACTTAGGGTTGTGATAATTTA[C/G]GTCTAGATTGCTGATAATCTTGGGGCATTGTACAATATATAGTCTGACAGTGGCCATAAAATGGACATTTGTTACTGCATTTGCCTTCATGTTGCTTAAT
9	52314339	SCAFFOLD290694_4742	0.28	0.34	TAGTTAATATTTTTCCAGGCACCTTTTTTTTCCATTTCAGGCACCCTTATAAAAATAAGACTCTAGTTCTAAGAGCAGGCATTTGGACCTTTCTGGCAGG[A/G]ACTCCTGCCCTGGGCAAGCACACTTGATGATCACACGGTACATGGTCCCTTCTCTCATGACCATCTGCTAGACTGGCTCTCTGCCCACTCACAAGCTCAC
9	53334789	BES7_Contig455_1223	0.22	0.39	TGCTTGATTTTTGTTGACAGTGATCATGTATTCATGGATCCTTTTAGTAATTTAGAATTTAGTTAAATTTGCACTTAATTGATTCTACTAACTAAAACTT[C/G]TCCTCACATTTGGGATTAAACTTGAGAGAGGCAGGGCTAGCTCTGTGGCCTAGATAAATGCTGACCTCGCAGAGAACAAAAGTATCCTATAAATGTCCCC

9	53619231	BES4_Contig462_956	0.33	0.47	CTAAATAAAAGCCCCTACCCCCCTAGACTTACACTAAGGGGTATACTGTGACAATGTGACACATAGGCAAGGGCTCTGCCACGGGATTAAGGTTCATGCC[A/G]TAGAATTAAAGGCTTTAAAATAGCACAATTAATATCCAGGGGAATAATAAAGAGAAACACCAAGTAATATATAATCACAGTATCATGCTGGATAGAAAAT
9	53619344	BES4_Contig462_843	0.32	0.47	AATCCCGTGGCAGAGCCCTTGCCTATGTGTCACATTGTCACAGTATACCCCTTAGTGTAAGTCTAGGGGGGTAGGGGCTTTTATTTAGGAAAATAACTAC[A/G]TGCTGAATTCATCATAAGTTGGAAAAACAGATCCTCTTTCATTCATTGAACAGACCACACATGTTTCCATAAGATGTAGTGCCATGTGGCCAAGGCTCTG
9	54574545	SCAFFOLD292194_2697	0.45	0.42	ACTTCAATTAAAAAAAAAGATTTCTCATGAAGACTTGCCCAAGAAAACTAGTCGATTTCTCTCCTGTTACTTAGCATCGAGAATATGAAAAAGAGAAGGG[A/G]ATATGTTAAAGCCCATGTATAGAACTGTTCTTAGACTATACTGTACTTCAGGTGAATTTGAAATAGTGCACTCAAAATTTCCCAAGTGGGAGTATTAAAA
9	55659013	SCAFFOLD255517_8183	0.36	0.40	CTTATATCTTTTCATCTTCCTGAGCTCATCATGACCCTGCTTCTTTCCTATGCCACTGTAGGAGAAAAATTAGGATAATTCTCGTCAGTTCTATTTGATC[A/G]AGATAGCCTTTTGTTTTACTTTGGTTCAAAAAAATCACAGGAAAAAGACATCCTGACGCAAGGTGATAGGGTAGCTCGAAGAACCACTTGTGGACAAAGC
9	56206600	SCAFFOLD140116_30449	0.47	0.39	TTGGATTTCTCCAGGATCATAGTACCAAGGAGCTGAGAGTTTTGATGAGATAATGTCTAAGAAAGGAAGGAATAAGTAGAAATCAGAAGGTTTACAGCTC[A/G]AGCAAATTCAATAGCACATATAGTGGAAGTGAGAAAGTGAGAAAGGTGGGAGTCTAAGAAGTTATAGGATATTTGAGTGTTAAGATTTCCAAAGTAAAGC
9	56206860	SCAFFOLD140116_30189	0.45	0.39	AGTAGTGTGGTGTCAATACAGATCCATTGTTCCTAACTCAACTGGGTCCTCAATAGCCTGAAAAACAAAAAAGCCCCTAGTCACTCATCCATTCCTCAGA[A/G]TGGCTCCTTTATAATCCTCAAAACTTTTCTAAAGCCTCTATGATTTAAACTTCTTTTCATTCTTCAGCCAGTTCAGTTCAGTCACTCAGTCATGTCCGAC
9	57990257	BES8_Contig519_816	0.21	0.16	TATTGTTAAAGCTGAGCTAAGAATAGATAGATGACAGCTGATATCCAATACCTTCCTGAAAAGCACTCAATATTGAAAAGGGGAATCCTTATAGGACCCA[A/G]TAGTCCCATAACTATGCAATACATAGCCCATTACTTAGTTAATCAGAGATATCTCTAAGATACTGGATTTCTGATTGCAGATTTAGCACATTAACTCTGA
9	60969293	SCAFFOLD175228_4641	0.46	0.48	GTTACCCCTGCTGCTCACCATCAAAAAACTGTGTATGCATTTGGTTGTCCCCAAGAAGAGCAGTGTGCAGCAGGAGAGTGCTGGTATCAGGGAGAGAAGT[A/G]GCAAAGTGCAAAATTGTGTCCATCGACTGTGCAGCCTGGCCTGCAGTGCTACAGTGGTGGAGGCTGGGGCAGTAGCTGCCAGGCAGAAAGTTTACCCTCA
9	61552088	SCAFFOLD75088_30189	0.17	0.17	CATCACTGACCTGACTATAAAAAAAAAAATCAAAGTTTCAGTAAAGAAATTTAAAGACTGATATTAAAAATCAGTATTGATTTGTTATGGCCCATTTCTC[A/G]GCATGCAGGCTGTCTGATTTTACCCAGAGGGTGTTACAGACCTGGGTTTCTATTTAGAATATACTGAAGTTTACACCTACCAGACACACTTATGTCTTTA
9	62043459	BES7_Contig493_1415	0.37	0.50	AGACCCAACACAGACAAATAAATAAATATTTTTTTATAAAGAACTTGGACAAATAAATTGTATCTGTTAAAATTTCTGAGGCTAAGATGTAATTTCATAC[A/C]AGAAATGAGGCATATGCATAGCTTCCATTTTTTATTCTAGCCATCAAATGTTTTCCCCATGACTGGGAGAGATGTCCCATTTCTCTGTACTGAATAGGTC
9	62043638	BES7_Contig493_1236	0.25	0.40	ATTTCTCTGTACTGAATAGGTCAATAGCACAAATAAATTTGTTCTGTAGAATTTCTGATGCATGGTGTGACAGTCTTATGTAGTTGTTTGCTAATTGCCT[A/T]GTTCACAAGCCTGAGTGTGGTAAAATATTTTATTCAGCCATAAAAATAATGACATTAAAAATTAAATTAATAATGATTAAAATTAAGTATTAACCATTCA

9	62946258	SCAFFOLD321728_27398	0.26	0.20	AGTCCTGTGGTAACAGGACTGGGCCATGCAACCAGCAGCCCAGGGCCCTCGTCCTTCCAGCGCCTTCACTGTTTTCCTAGAGCTGTTGCTTCAGTTCCTC[A/G]TGCCCCTCCTTTCCAGCAACAGAAATGACTGAATCCTGAAAAACACTGCTGTGGAGTTATTTTTGGCTCTCTTCCCGCCAGCCCTGGTGTCTGCCGCTCA
9	64770216	BES4_Contig366_1332	0.33	0.44	AGACTAGGGTAAAAAGTGTGGAATATGCCCTCATTTTTACTTGCTACCCATTTCTGACATGAGGATCCATCCAATGATGTATATTTGTGTGTACGCATGC[A/G]TGGAGATGACAAAACCCACAGGCAAAATGAAGGTGACAGTCACTGCTCAGTGAAATGACCTCAACCCTCCTCTTTTGCTCAGCATCCTGCTCCTCATTCT
9	66156866	SCAFFOLD165270_13385	0.20	0.20	TAGAACCCAGCTGCTTCTATATGTCTAGTTCTCCTCTGCAGCATTAATAGGTTCACGGGAAGCATAAGAAACGTGTGCTCCACTCACCTCTATGACACGT[A/G]TGTCTTCAGCAGAGGGTGCCGACGGCCAAGGCAGCACGGAGACCGTGGAAATGACCGCTTTATCCTCAGAGCAGAGATTTCCAGGCATCCCAAGGTGTGC
9	66690353	BES3_Contig286_1171	0.26	0.24	TTTTTCTGACCAGATGTCTATTTTCAGTGACTGTGTGTAGCCACTAATAACTCTTCCCTCGGGTGACTGAGGAAGCACGGTGGCCTGGTTAATTTGTTTT[A/G]CCTCCCCGTTTTCCCACAAGTAGACCAGGAATTGTTGCCAATTAGTCAACGAGGATTTGCTAAGTGCCCACAGAGTAAAAGTGGTATGCTACGTTGGGCA
9	67217752	SCAFFOLD308591_803	0.45	0.47	TGTTCTCATTGTATACACACACACACACACACCCTACCCATTTATTTTCCTAGAATCTTCTAGAGGAATCAAATCCATAATGATAATGATGATATTGACA[A/G]TGATTGCAGGGGCCTCTTGAGGACTCTGATCAAGAGGGAATCATTGCAAGGCTGCTGCAGAAACAGGCTTAATTGCTGTAGTGTTTAATGCAATCAATTA
9	70979263	SCAFFOLD110029_13368	0.43	0.48	GAAATGTAAACTTCACTAGCTCTCAAATTAGCATAAATCAGTCTCTATTCTTATCACTTATTTTATAATTGTGTTGATTGACCAAAGCATAATGGATGAA[A/G]GGAAAGAAAAGCTGCATTGAACAGAACTTTCATCTCTTATGCCCCAAATTTATTCAAGCAACAAGTAATTTGGGGTGTCAAAGTGCCCTCTTTTCCCTGG
9	70979419	SCAFFOLD110029_13524	0.43	0.47	TTCACAGGAAAATGGTCCTTGAAATAGATTCCCTATAATCCCATCATATCTGGGATGGTGATAACAATTTGATGTTTCTGGTCACTAACAGGCTATGACA[A/G]GAGTGAGTGGCTCTCTGGGAATTGTTTAAGGGTGAATGTTTGCACCCCAGGGTCTCCAGGGAAAAGAGGGCACTTTGACACCCCAAATTACTTGTTGCTT
9	71183777	SCAFFOLD63387_599	0.07	0.08	AAATAAAAATTCCCTCACTTAGCTCAGAATTTCTTAGATTCTCTCGTGTCTGTGATGCAAAGGATTGGCTACATTATTTTTTCAGGCCCCTCCCAGACTC[A/G]TGATTCAGGAGGACTGTATATCCCCAAACGTATTGTTGTAGGACTTATTTCAGAGCCACTTCTGTTTTCTTCTTGCTTCTCTTAGAATTCAGTACCTTTT
9	73154749	BES9_Contig154_466	0.10	0.19	TCCTCTCAGCTTTTAAATCATATGATATCAATCTTTGCTCATTTTCCTCTGGGTAATTTGTTATTATGCTTATAGTAACCAACTCCATGAGACATTAACT[A/G]GCAATCCACGTTACCAGTGAGTATACATTACAGCACTAAAATAAAAATAAACTCATTGAAGAAATAGCTAGAGACCTCAACCATGCTTTTCTGTTGGTGA
9	73154819	BES9_Contig154_536	0.36	0.34	TATAGTAACCAACTCCATGAGACATTAACTAGCAATCCACGTTACCAGTGAGTATACATTACAGCACTAAAATAAAAATAAACTCATTGAAGAAATAGCT[A/G]GAGACCTCAACCATGCTTTTCTGTTGGTGAAATGGCATGACAGTTTGGGGATTTGTAACTGAGAATAAATTTAGTTATGTCACTACCTTGCTGCTGCTAA
9	73154971	BES9_Contig154_688	0.45	0.50	CAAAAACAGGAATTCCCAGGATCGAATTTAAGTCTCCCTTCAGATGGTTTAAGAGTGAGAGAGGCAGCCCAGTTCTTCACCCAGCTTTGTAATGATCCCT[A/C]TTTTCCCAGCCTGGTCTGTGCTGCCCTCCTGATATACATCTCAGGCACTAATTAGCAGCAGCAAGGTAGTGACATAACTAAATTTATTCTCAGTTACAAA

9	75357799	BES1_Contig431_794	0.26	0.43	TAAGTGACTTGGTTAATTTAAGCACACAATGTAAATTAATATCAGTACTAATTAATTAAATAAAGAAACTTTTGGCAAAGAACAGTTTTCTGCTAAGACA[A/G]CTGAGATTGTTGAACTGCATATGCAGAAATGCATACTTCCTTTCTTAGTTTGGTTTATTTTTCTATGACTTCATTGCAATTCAAAGAATAACAAAGAACA
9	76064305	SCAFFOLD152924_4453	0.45	0.31	AGAAATTAGTAGGAAGGGAGAAAGAAGCTAATAATGAGACCGAAGGTAAAAAAAAAACCTATGGAAGATGTGTCCGAAATATGAACTCTTGGGGAAAATG[A/C]AATTTATCAATTTGTTGCATTTTTAAAAATGTTCTGGATTTGGAAGTCAACAGAGGAGATCCAGGCACTGAGTTATATGATCAAATGCCTTAGATCTCCT
9	77028408	SCAFFOLD323674_1825	0.41	0.39	AAAATCTCCACTGTTGGCCCCACCAGAGGAGAGTTGCAACAAAAGAGGCAGGTAGCAAGTGGAGAAGGAAGTTGGTACAGGAAAGAACACACAGAACCAT[C/G]GAGCTCCTGACTTTGTCTCATTAATGCCTGGACACATTTTGTCTGCTGAGTGGAAGAACCAATGCTTTTTCCAAATATAAACAGTGCATTTGATAGGATA
9	77028478	SCAFFOLD323674_1755	0.40	0.39	CTTTCTAATCTTTGCCCACAGAACACCTGAGAGTCATGTGCAAACATTCGACAGACGGTTCTTGGCTGTGTATCCTATCAAATGCACTGTTTATATTTGG[A/G]AAAAGCATTGGTTCTTCCACTCAGCAGACAAAATGTGTCCAGGCATTAATGAGACAAAGTCAGGAGCTCCATGGTTCTGTGTGTTCTTTCCTGTACCAAC
9	77225083	BES7_Contig402_572	0.26	0.34	TAATATCAGTTCCTTTCTTGATTGAAACTGTGAATAAAAATCAGCTCATCCAACACACATAAGACAAAAATACCAGGTTCTAAATCTTGTGCTGGCTAGG[A/G]TGAGTCAATGCCCAATATGTGACACAGAGGGGCCAGTGACAGGCAAAGGGGGATTAGCCCTTTTCTACCCTTTCAGCTCTCCCCCATTTTCCTGCTGGGC
9	78440704	SCAFFOLD150039_18054	0.28	0.15	CTTGTCTTGACCTTGGTCTCTCAGAGACCATGGGAGGGGATGAAAGAGATGTGAAAAGGCAGAAACACCAGGAAGAATGATGGACTGACGGGGCACTCTG[A/G]GAAGAGACCACTCAACTAAGGGGTTTTTAATTATCTCTTTTTCCCTGTGAAGATTTGACTGACTTCACCGTGACCTGAAGTACTATTCTTTGGAGATTAT
9	82711720	SCAFFOLD240057_5347	0.22	0.28	CATCTTTAAAATGCTTCTCAGGTCTTTGGCAGTGAGTTCCGTGCTAGAACTGCCCTCCGTAACTGACGTGCACACAACTTTCTCTAATTTGAATGCAAAC[A/C]GGGCCGAGACATCCAGGACAACAGTAAATAAGCTCCTCTGAGCTGTGGGGGCACGTCCAGTGTGTTTATTACACGGCACCTATTGGTTTTGTCTTTCTCA
9	85062574	SCAFFOLD130401_10348	0.49	0.42	AAAGAAAATTGAGAATTATGTATATATATATAAGAGAGAAGAGAGAAACACAGAGAAAGAGAAAGTGAGACAGTGAGAGTAAGAAAGAATGTATTAGGGC[A/G]TATGAGAGAAACTGCAGGAACACATGTAGATTATCAGGATATAGGCTGTCTTTCTATTTTATTTTCATAATCATACATTCAAAGTTCTAAGTCTATCAGT
9	86008057	SCAFFOLD190221_16495	0.11	0.15	GGTGTAATCCTACAAGAGTCATGGACGAATTTCCTAATGGGGTGTGCAGAAGGGTGTGGAAATCTGTGGCTTGGGAACAGCTTGTGAGCTGCAGAAAGAA[A/G]GGCAACATTCCTTCTGGCTCTCAACATTCCTTCCTCGACCACCTGTCACTTTCATGTCGAGGGGATTGTCTCATGCCCAGAATACAGAGTACCTTTATGA
9	86636705	SCAFFOLD150952_3905	0.22	0.24	GTCTATGTTTTACTTCTCCAGTTCTACCTTTATTGGTACTTAGCAAAGTCCTTTACATATGTTATCATTTTTCAATCCTTAAAACTGTATGAATTTAGGT[A/C]TATTATCACCATTTTACCATTGAGAAGGTGAAAGCACAGATAGGTGAAGTCTCCATTGGGGAGAAATGCCTTCCTTTCTCTTTACTTAATGCTCCTTTGT
9	88354914	SCAFFOLD141002_4349	0.15	0.17	ATTGACATCACCTTCCTTTAAGTCACTGATAAAATGAGTTGTCCTGTCCCTGAGCTCTCTCCAGACCCAGCTTAGCTCAGTCAGTCATGATTCTAAGCGA[C/G]TGCGGGCAGGGACCAGACCTCAGTCATCTTATTCCCCTCAGTCCCCAAAGGCCACACACTATACCTAATAAAACTGCTGTAGATCCAATTGAATCCAAGG

9	88395434	SCAFFOLD210030_5194	0.27	0.28	TTGGCTTCATGATTTATATTATATGTCTTTTATGAACATCTTCCACTTTGAAATAAGAGGTACTAGAGTTTCTGCCAATGAATATGTCCAAGATGTCATT[A/G]TTTTTCTTATTAAAAAATGCCAGCTATCTGTAACATATAATGACCATAATGAATTTCCACTGTTGGGTAAAAGTAACTGTGTGATTATGTGAGTGATAAT
9	89329673	SCAFFOLD130158_5039	0.18	0.18	CTATCATTAGGATAATTTAAGTGCACTTTCCAGTGTTTTTCAACATTCACTTTTTCCCTACCTATCCGGCTTCACTTTTCTTGGTAATTTCTTTTTCTTT[A/G]TAATTCTTCTGTCTTTTGCTATTTTATTGTCCCCAATATTCTTCATAATTTATTTCATTTGAAATTTGTATCTATTGTTCAAATTGTGCTTTTTAATACT
9	91137263	SCAFFOLD129955_485	0.36	0.32	ACTCAGTTTTAGCATCTCCCCTCTTTTTAGAAGTAAAAGGAGTCCACATTAGCAAAACCAAAGATTTCCAGGAAGTACTGGTTGCTGTAATGGTGGGGAA[C/G]GTGTAGATGAATAAATCACTGTTTGTTAGGAACTATAAAATATTTTATTGGAAGTCTGCCTTCTATATTCAGATTTCCTTGAAGCTGAAAGCCTATTGAG
9	91242005	SCAFFOLD175636_20769	0.45	0.44	TCTAAAGAAGCCAGGGAGAATCTAAGGTGACACATAATAGGGAGAAGAAAGATGGAGGGTGTTGGGAAGGAGGGTGGTTGGAAGAAAACGTCCCAAATAG[A/T]GAGGGAAGCACCTGGGCAGGAAGAAGATGCTGCTTTGTTCAGGAAGTCTCTGGAATGCTTTGAGTCACTTCCCCTCCCCACCTAGAAAAGTGCAGAATCT
9	93443309	BES9_Contig542_948	0.06	0.12	GGCCGCATCCACACCAGCACCATGACTGTAGCCATCCTACCCCAGCCCACTGAGGTGAGGCACCATAGCCCCGCTCCCGGGAGTCCTAGCCCAGCACAGC[A/G]CCTTGCCCCCAAGTAACACTCACCTCACTGAACTGAGTATCTCCTTTTCTTATTTGACTGTCATCTTGTCTTTGAATTCATTTTTCAAATATATTATCGC
9	94364802	SCAFFOLD111929_14239	0.38	0.48	TTATGTTATGAAAACATATCTACAATTTATTTTTGTTCTTAATACATTTTTATAGTGATCAAGCATATTTCTGCTTAGAATTTATGCCATTGCTTAACAT[A/G]AGAATTTTTTTTGGTCAGCTTTAAAATATACATCTAGATATCAGAACCTTAACTGATAAAATATAACCTACAGTTATATCATGGAAGTAGTCATCAAGTT
9	96468568	SCAFFOLD65016_14724	0.26	0.50	TCATAAAATACAGACTTGGTGGCTTAAATGACAGAAATTTATTTTCTCGAAGTCTGGAGGCGAGAAGCTGAAGACCAAGGTGTGGCAGGGTTGGTTCCTC[A/C]AAGAGGCCTGTCTCCTTGGTGGCTGGCTTCTTGCTGTGTCTGTCATGATCTTTCCTCTATGCGTGTCTTGTCTGGTGTTTCTTCTTGCATGTGTGTGTCT
9	97505265	SCAFFOLD145441_3461	0.31	0.47	TGCAGATTCAGCTGTTATTACCAAATAGCTGCGTGGTTCACCAAAACTTTTGCATTTGCAATCCTATGAAAATCTGGTCTGATTAACACTGGCAAAGGCC[A/G]TACACAGTCTTGGCAGGAAAACAATGTGATCTGACTTGCATTTTCAAAAGCTAATTCCACCATAATATGCAGCATGAAGAAAGGACTGAGGGAAAGTAAG
9	97507613	SCAFFOLD145441_1127	0.35	0.50	CTTAACCTCTCTCTTCCCATGAGAACATAAGCCCCAGGAAGGCAGGGATCATATTATGTGACCCCTGCTGTACCCTCAGGGCCCAGTTGGATGTTTGCAC[A/G]TAATAGGTGCAACAACTATGTACTGAATGAACGAATTTTAAATTCCTCTTGTACACAGAGTGCTGAGCACCACATGGTAGGTGCTGAGCAGATAACTGGC
9	99397888	SCAFFOLD247622_5113	0.50	0.34	ACATGTCACGCAGCTACCAGGACCCAAACGCCCTGCGGTGGGCCCAAAAGTGTGATCTTCCCTTTGGGTCTAGGCGGCATCTCGGTATTCACTTCACCTC[A/G]TTGTTCTGCAGCTAAGAGCTGGTGCTTGATTCATTAACTGATGAAGCACTCCCATGCTTTTCCCTTTTAGCCTCTAGGGTCTAACACGCTTCTTCTGCAT
9	100858720	SCAFFOLD59205_535	0.10	0.08	CAGTGCTATCAAAATTTTCAAGTGGCTGCTGATCAATAAAGTTGAGAGCCAGTCAATACCTGTAAGTGATGACCCTCGAAATAAGGGTTTGATCCTCCAA[A/G]TTGAAATGTCTCTATATGAATGCCCAAAGTTCTTTGCACATGTTTTCTATCACGACATCATCTATTATAAAAGCATTTTATTATGCTTATTATTATTTAT

9	100948908	SCAFFOLD141809_449	0.39	0.28	TCGACTGACTGTGAGTATGTAAAGAACACGCTGGGCTCAGAAGGCAAGACCTATGGTCGAACTGCGGGACACTGGGCCTCCGTAAAAACCTGGGGGATCG[A/C]GGCTAACCAGCTCACCAGCACATACCCCAAAGGGTCCAGGAAAGGAGTCCTAAGGGAGACCCACTCAGTGTCAGAACCATCCTCCTGGACTCTCACCGTA
9	100948911	SCAFFOLD141809_446	0.37	0.28	GGTGAGAGTCCAGGAGGATGGTTCTGACACTGAGTGGGTCTCCCTTAGGACTCCTTTCCTGGACCCTTTGGGGTATGTGCTGGTGAGCTGGTTAGCCTCG[A/C]TCCCCCAGGTTTTTACGGAGGCCCAGTGTCCCGCAGTTCGACCATAGGTCTTGCCTTCTGAGCCCAGCGTGTTCTTTACATACTCACAGTCAGTCGAGTG
9	103545105	BES9_Contig562_937	0.18	0.30	TGAAGATGTCAGGCTGTGTTCCATGACTGTCAACAGCTGCATATTTATCACTGCGCCATAGGCCCTCTCAGACTGAAAGATTTCACTGTTTATTCACAGC[A/G]AAGACACTTAATTAATGACTGGGCCTAATCGGGCTGTCTTATTATTTACAGGGATACCAGTGTGGCTTAGCTCAAGCGATGTGCAAACAAACCAGCCTTG
9	106897965	SCAFFOLD105074_11875	0.17	0.14	GAAAAAGATCTGAGGCATTTACTGGAAAATCGATTCCTGACCATGCACAACAGGGCGTGAACTCTGGCTGCAGAGAACTCTGAAGAGGACACATGCTCGC[A/G]ACCATTAAGTGCATACGTGGGAACCCAGTTTTGGTTTAGGAGACAAGCTTAAATATTTCAATGCATGCATGGTGACAAATTCCAGTTTTATGGGGAGGAA
10	3099875	SCAFFOLD120193_885	0.35	0.43	ATATTTCTCCTTCACTGTCTGACTGACTTCACTCAGTGTGACACTTTCTAGGTCCATCCATGTTGCTGCAAATTGCTCATCATGGATCCTGTGATCTATC[A/G]GTTCCATTCCTAGGTATATCCTATAGGCAAAGTCACAGGTGAAGGTGTTCTTGGCCAGTACCAGGAGTAGGGGGAAGTAAGGGAAGATGCCTAGTGCTCA
10	3099948	SCAFFOLD120193_812	0.36	0.44	TGCTCATCATGGATCCTGTGATCTATCGGTTCCATTCCTAGGTATATCCTATAGGCAAAGTCACAGGTGAAGGTGTTCTTGGCCAGTACCAGGAGTAGGG[A/G]GAAGTAAGGGAAGATGCCTAGTGCTCAAAATTTGAGGAGGTGCTCACTCCCAGGGTCATAAAAGTGCCAGAGTGATCTTGATCAGTGAGTGCTTCCTTAA
10	3100159	SCAFFOLD120193_601	0.36	0.43	CCAAACACCTTTCTTGCCTCACCTACTGTCATCTCTGGTCTTGGCATCAGTGTTCATAAGAGTCAAAACACCCAAATATCCATCAACTGTAGAATAGGTT[A/G]AGTAAACTCTTATTTGTAAAATTCACCATGCAGCAGTGAAAAGAATGAACTGGTAATATGCAGCAACACATATTAATTTCACAAACAATATGGAGCCTAA
10	3269636	SCAFFOLD285310_5186	0.40	0.48	TTCTCCACACGTGTGGAAAGAAGATCCCAGCGTCTTTCCTGAGTGAATCTCAGAGCCAAGAGTGGATATTTGTTTCGTGCTTTAAGCATCATTTTCCCTT[A/T]GGCAGTGGCCAGTGACACAAGCTTGTCTGCTGAAAGAGGCAGTCTTCCATGTCGTATGCGGCTACGCCGTGTTGGAGGCCTCTTTTTGGTACGTCCTTTG
10	4359586	SCAFFOLD45894_1504	0.35	0.26	CAGCTGGGTGGTAAAGGAGTGGTAAGCTACTACTTACTAACCTACTACTTATTTACTTACTAAGTAGTAAGTAGTAAACTACAGCTCCCAGCTTCTGGCT[C/G]CCATTCTAGCTCCACATGGCCACAGTCAAGACCTATTAATGCAGCATCACACGTAAAAATTAATCTCACTGCCCTAAAGTTGACTTATCTAGCTTCCCTT
10	5630805	SCAFFOLD669_15136	0.45	0.29	TATTTTGTCGTTTTCCCATTCCTGAACAAAGCACAGAGGCCTCGGGATGTCTAGTACAAAGGGAGTGTGTTACCATCTCTTATTTTCAGGTAAGAAAAGA[A/G]ACTCACTGCAGCAGTGCAGAGTGACCAGAATGACATGCTGCCCTGGGATTTAAATGCAAGAGCGAGACTCCAAAGTCCCCTAGACCTGATCCTGACCAGG
10	7858886	SCAFFOLD311748_23298	0.16	0.13	AAAGTTCAATAAGAGAAAACTCTCGCTGTCAGCAGTTCTTTGCTGGTCATCACTGTTGTTCGTTCCAATTCATGATTATTCAAGACAGGAATTTCAGTGC[A/C]GTAGGGGAGCGTCAGCTGCAGCCCTGGTGAGCTGGGGCCTGGGCAGAGCCAGCCCTGGGCAGTGGTCAGCTCACACCCCACCCCGCCAAGCAGACAGAGT

10	8143120	SCAFFOLD26390_1527	0.46	0.50	CTTTCTGTCTGCTCTATTAAAAAAAAAAAAAAACCACTTGAAGATACTCTTCTCAAACTTCCCAAACAGCAAAACAAATACCAAACAATGAGCCCAAGAAC[A/G]CTGAGAGAAAATTCACGCCACATGCTGGATCCCCGGCCTCAATGGTTACCTTTACATAAGCTCCCAAAGTCAGGGCCGAGTTGGTGCCATTGTTTTCATG
10	8432880	SCAFFOLD313308_2790	0.38	0.32	TGTTAAGCAGTTGTTTTACTGTTTAAGTATTTATCTTCTTAAGTCCCCAAATAAAGAGATTATGATTTTCTTGCATGTAATAAAGAAATCTGTTTTACAC[A/G]GTGATAGCATGTTCAGATACTGAAATAGAATTCAGCGAACAATGGCCTCTATTCCAGACCATTTATAATTCTGCAACAGTGCTGGGTGCACATTTACTAA
10	9401741	AJ496782-118.T7-311	0.43	0.36	AACACCATGATCTACCAATCACAGCTATTGAGCCCTCCCTATTGGCCAGAGCACCCTGAYTGCTGCCGTGRTCTTGATGCCCACTATGGCAATTCTGCCT[C/G]CTTTGCTCAGATGGTTATCTGCCACCATCTCATCTGTCATTTCAGGATTCTGTCATTTGCATTGACATGAGGGAGCTCATTTCTATGACGGCATCTGCTA
10	12484781	SCAFFOLD100416_40455	0.34	0.40	CTCTTCTGCTTCCCCCAGCTTGTCTGCTTCATTTTTCTGTCTCTGCATGTTGTCTTTTACCACTGCGCATGGCTGAGCGAGTCCTCTAACTCTTAAATTC[A/G]TATATTCACTCATGGTTCTGTGATCCCATTCTAAACCCTGGGAAAAAGTCCAGTTTGAGGCAGATGTCTGTGTCTGGTCCAATCAGCTGTGGCTGATGAA
10	12645043	SCAFFOLD230250_13663	0.07	0.20	CTCATGTTCACACTAGGTAACACACAGGTCTTGGTTTCTTTATTAATTACAGGCAGCTGTTGAAATCAGTCTCCCCAACTTTTCACCATCAAGAAGCCTT[A/G]TTGGTGTCCCCTGTCTGCAAAGCTCTCTCAATTTGAGAGACACTGCTGTCCCCACGCGCTGGTGTCCAAGGCCAGTCACGTACTGAACAGGTGCAATTTG
10	12645355	SCAFFOLD230250_13351	0.03	0.05	AGGGTCCTTAGAGATGATCCAGCCACTGGTTCCCAACTAGACACGTACAGAAGCTGCAGCAGGTTGCCAGGAGCGATTCTGAACATTTTACAAAGCCTAC[A/C]GGCAATTTTCCATTACCTAGGATGAAGCTCACTAAAACACCAAGGTTTTTCTTCTGCTGTTAATTAGAATTAAATTAACTCTGTGATCTTCCAGGTGAGC
10	14420657	SCAFFOLD300198_20107	0.08	0.06	TACTGAAGTGGTCTGAATGATGTGTTAAATGATGAAAGCAAGGTCCGTGGCTTAGAAAAATATCTCTAGATAGGTGAAGAGCACATTCAGAGAGTCACAC[A/G]AGGTCATTATCATCAAAGTAGCACTGTCTTCTGAAGATTTGATGGGTTGTATTGTTCTGACAGAGTTAGAGTTTTTCTTGTGAGCTTCAAAACCACTTTA
10	14478637	SCAFFOLD80554_8307	0.23	0.36	GAAAACAAGGCTATTACCCACTATCATTTCTGTTTCGGCTTGACATCTGTGGAAATTTCAACACAGGTTATCCCTCCCTCTGTCTCCATAAAACTTTAAT[A/T]TAACAGCTGATCAATTTTTTTGGACAGAAGGCAGGCAAGGCCCTATCTGCTCAGAATGGGCCCCACCCCCAGGTTGGTCCCAGACCACACAGCAAGATAA
10	14514569	BES8_Contig490_1171	0.45	0.50	TGATTAGGACCCTGATTCCATGAGAGCCTTTCCCCTTCCTTACTCACGCCCATCCATAAACCCTACCTTCCTGTCCAAAAAAAATCTTGAAGGTGTCAGC[A/G]GAATTAAAACAATAAGCTGTATACTTTCCAAATCACAGAAGATAAAAATAAATAAATCATACCTATCACAGAAGGCAAGAAAAAAGAGAAAGCTTAGATC
10	14514967	BES8_Contig490_773	0.45	0.49	ACAGCAGTCTCCCCCGCGGTGCGTGCAGCCTGCTGCCTGTAGGCATTGTCTTAAGGAACCCTGGAGACAGCCCTTTCCAGTGGACATTCATGACTGGATG[A/C]TGTCCCCAGTCTAGGCTCCAGGCTTGTATCCAAGGTGCCTCTGGAAAGCCCAAGGGCACAGTGGTCAGTGTCCACATAGGCCAAGTCATAACGCCCATCC
10	14515215	BES8_Contig490_525	0.13	0.17	AATTTTTGAATAAGGAAGACTTACATAGTTTTGAAAAAGATAAGATAACTAAGCCTTTATTGGAAAGGTGTGGAAGTAAGGAACTGGAAAACTTCTTACA[A/T]TGCAAGAGAGACAAAAATTGTAGATTTTTCTTGAATGTGAGAGTTGGAGAAACATGGGTTCCATAAAATGTTTTACCGTCTTGTTTGCAGCCGATTAAGC

10	15035089	SCAFFOLD90340_12768	0.04	0.13	AGACATCTGGCTAGCTGCTCGCAGCCACAGGGAGATGAGACTAGTATTCGATAAGTGAGGGGAGCCCTGGGCCTAAATGACTCCAACACAAGGTAGAAAG[A/G]GACTCGGGCCGTCAGAGAGAGAGGGCTTGGGCAGGGTAAGGCCATTTTAGGCTGGGAGAAGGATCCAGAAAGCCTTCATGGAGAAAGTGGTCTTACAAAG
10	15316650	SCAFFOLD105073_11046	0.34	0.40	TACTTAGCTGTGCCAGGCCCGGCTTCCAGCTTCAGCCTGGATGTGGAGGCGGCAGGTGGGCAGCCCTGGTGGAGTGTGGCAGGAAAGGGGTGGTGTACCT[A/G]GGGAGGCCCGAGCGGCTAATCAGGCCTCTGGGCACCGTGCCTGCTCCCTGGAATATTCATAAGGCCCCTCCAGCTGTGCCAGGCAATCAGGGTGCTGATT
10	15794481	SCAFFOLD231091_2678	0.22	0.28	TTTCTTATTTCCTTTTCCTTTTTCCTGTTGAATTTTGAGAGCTCTTTATGCATACTAGTTACTAGCCTTTTGTCATATATGCGGACTTTGGTAGAGCACA[A/G]GTTTTTCACTTTGATGAAGGTCAATTAATCCTTTTATAGATGATGCCTTTGGTGTCAAGATTAAGAACTCTGGGTTTAGCCTTATGTCTGGAAAATTTTC
10	16698523	SCAFFOLD95025_37039	0.21	0.33	ACGTGTCATGTAGTTTATTGGGACTTGTTTGCTCGAGGCTGTGCAAACACAAGAACAAAGGCGGCTCTGAGTGTCACCCCCAGCCTTTGCCATCACTTCT[C/G]TCACTGGGAGCTCAGCGGTCTTCAGCACGCCATCCCCTTCCAGCCTGTGGTGCTTGAGACGGGCCATCAGGACTGGACAGGAGATGAGGCATTCTCTGAA
10	16698621	SCAFFOLD95025_36941	0.35	0.36	GAGGGAAATTAAACAATGCAACCTCCTCCTGTACTGCAACCTTGGCTGTAATTCCTTTATTGTTCTAGAGACACTTTCTCTTTGCATTCTCAATATCCAC[A/G]TGTCATGTAGTTTATTGGGACTTGTTTGCTCGAGGCTGTGCAAACACAAGAACAAAGGCGGCTCTGAGTGTCACCCCCAGCCTTTGCCATCACTTCTCTC
10	16835735	BES7_Contig491_1253	0.17	0.34	TCGCCCCATAAGATTAACTCGCAGTCACCTCCGTGAAAGCTGATCTGAGTGGAAAATCAAGTTAGAAAATTCCGCCTTCCCTTTCGCTCACACCTCAGCA[A/G]ATTAAGGGAAGGCCTGATGCTGAGTTATGGGGATTTATGGGAATGTGCAGTTGCTGGCTGTTAAGCGCTCACTCTTCGGGATGTTGGCACCCTCGCTTCC
10	17286691	SCAFFOLD61134_10794	0.03	0.03	CAGGCCGGGAGGTGTGTGTGCAATTCAGGCTGACTTGTGAAATGCAGGCATGATGTGGGTCACAGGCACTGGGGAGGGCTAGAAGACAGCCTGTCACTGG[A/C]TCGAGGTGTCACCGTGGACCAGTCACTTCTCTGTCATCTCACAGCTGCTTCCTGAGAGGTTTGGAGTAGTCAAATGGCAAAATCTGTCGGCCCACACACA
10	17403592	SCAFFOLD55009_46185	0.30	0.26	GACTAGCCCCCTCAGAATCTAGCCCTCAGGGTATGAGAAGCCCCAGACTCACAGAGAGGCCACATGGGAGTGTTCTGATGATCAGCTCAGCTAAGCCCAG[A/G]CTTTGAGATATCCCAGCCCAGGTACTGGACAGGTGGGTGAAGAAGCTTCCAGCTGAATGCAGCCCCCAGAAATTCAAGACTTGAATCTTCCCAGCCAAAG
10	18654619	SCAFFOLD125215_9058	0.20	0.32	ATGGGCTTCGGGAACTGACCTGTGGTCTCCACTGTGCCTCAGTGGATGGAGGGCCATGGAGCATGGAGCAGTTTAGTGTTACCGACCACGAGTGAGCAAT[A/G]CATTGAATGATGATACCATACCATTACTTTGCCTGGGAAACAGTGGGTGCCCAGTGACTGTTTTCTGAATTTTGATATGAGTAACAGCTGTGAACTTGAG
10	18654669	SCAFFOLD125215_9108	0.19	0.33	GGGCCATGGAGCATGGAGCAGTTTAGTGTTACCGACCACGAGTGAGCAATGCATTGAATGATGATACCATACCATTACTTTGCCTGGGAAACAGTGGGTG[C/G]CCAGTGACTGTTTTCTGAATTTTGATATGAGTAACAGCTGTGAACTTGAGTATCCTAAAAAGTAAAACTGAACTACAGTCAAATGATGTAGACATTTGAA
10	20035877	SCAFFOLD1191_3568	0.46	0.06	AGAAGACAGTAAATTACGCTCAGTAGGCACCTTGAACTTCTGTGAGGAAGGGAGACATCCCACACCTGTAGTCCGAACTGAAGGGAGGCAGAGAACAGCA[A/G]TGTCAGAACTTGCTGCCCCTCATGTGGGGGTTTTAATGCTGAGACCCAGTGAGCTTCCTCAAACTCAGATCTGGCCCCTCTAGCTTGACTCTGGAGTGAG

10	20238385	SCAFFOLD140040_19121	0.32	0.31	TTGAATTTCTGTATTAGGACATTATGAACTTGAAAAAGAATTTCTGATCCAGATTTGATTATAAGTGGACATTTGTTTTTTTAAATGATATATCTGTGCC[A/G]TAAAAGTTTTTCAAAACATGTGTTTATGAGTTAAAATCATCCATGTAATATTAACAGCTAGCCACTTATCTAACTTGAAATTCCCAGGAAAGGAGTTCTG
10	21153203	BES9_Contig497_1108	0.27	0.28	GGTAGGTGCTTCCCCGGCCACCCACTTAAGGGCCTGGGCTCCCCTCAGTCACTCAGCCAAACATTAACAAGCGCCCTCTCCTTTTCCTTCTGATCTCCTC[A/G]TCTCTTTGTTCTCCCATCTGTCCTATTTGTCCACTGCCCTCTGAAGGAGTTTCATGCAGGTGAATCTGGGCCCAGGAACGTCAACTTTGGCCTTGTTTCT
10	21208472	SCAFFOLD317768_9669	0.07	0.08	TTCTTCCAGGCTCTCAGCCCTGGTAATTGGACATGTCTGAACAACACTCTCCAAGGGAAGTAAAATGGCAAAAGGATCAGCTTCCAGCCCCAGAGAGGCT[A/G]GTTATGTTTCTCAAGAATGACCTACCTAGACCTTAGAGAAGGCTTCATTCCCCTTCCTTGCATCAAGACAGTGTTTAACAAATCCAAAATTTAATTATCA
10	21208728	SCAFFOLD317768_9925	0.20	0.23	TGTAAATTCTCATGCTGCCAGGTTCGTTGCAGGTAGTGGCTCTAGAATGGAGATGTTTCACGGGAAGGGTACTCACTGTCCCTGACTCTACTGGGTCAGT[A/G]CCGGCTCCACTTGCCCTGGGGTAGCACCCAGATGCCAGCCTTATGCAGAAAATCCCAGAATCTCAAAGTCATTGGGATCTCTCTTTATTGAGGTATTTAA
10	21431396	BV105349-369-R	0.38	0.22	TGCACCCTCAGGTGGAYGGTCTCGGGCAGAGGCTGGCTGCTCTACCAGGCTGCCCTTACTCGTGGGGCCTCACCTTGAGAGGCCGGTGGACATCACAGCT[A/G]CACAGGGAGTGCAGGGACAGACGGAACGGCTCCCAGCTGGGGTTCAGATTGTTCTTCACCACCTTGGAGTGGCACAGAGAGCTGGTCACCCCCGGTCTAG
10	22615008	AJ245969-1143-Y	0.36	0.47	CATGTGTGAGGCCCAGTGCTAGGTGCTAGGATACAGCTGAAAGCCGCTGGGACACTGTCCCTGTCCCCRTGGCAYTTACAGTCTAGAAGGAGGCACACAG[A/G]AACCAATCCATAGATAATACAATGTCAGGTGATGAAGTGCAGGAACAGATCAGGCTGGATAATGGGRTGGAGAGCCAGGCACTGTCTGTGTTTGGTGGCT
10	22615336	AJ245969-815-K	0.02	0.04	CCAAATCACACAGTGTCAAGGATGGCCATGGATGCCTAGATCATGCCTTCTCTCTCTTGAAATACTAGATACAGTTCCACCTCATACCTCATTCCTGCTT[A/C]GGAAAGATAAAATGAGACTGAGGTTCTTCCAAAGTGACTTTGGGACATTACAGGAATTGGGAACKGTGGCAATGGTTGTTGAGTGCATTAAGGTTGTAAG
10	22615445	AJ245969-706-R	0.29	0.39	AGAGAACCAGAATGGCAGAAGTCTCCCCATCTCAGTGGTCAGCCCCATCAGAGAAGTCATCTGAGAAACACTCTGAGAGGCTAATATCTTCTCTTCCAGC[A/G]GTCCTCCTCTTACAACCTTAATGCACTCAACAACCATTGCCACMGTTCCCAATTCCTGTAATGTCCCAAAGTCACTTTGGAAGAACCTCAGTCTCATTTT
10	22615883	AJ245969-268-R	0.44	0.38	GTAGGAAATGGTTTCGGAGGTTTCCTACCATGCCGAAGAATGTATGACTTTAAGACTTTTCTTGAGTTTACCTAAATATGTGTTTAGTGTTGGACTGSGT[A/G]TCAACCAAGTGAGGGATCCTGGGAGAATTCCTGTGCTTGTAAATGAAGCTCACTTTGGTTTTTGTGGTGAAATAGATCACTGTGGGGTTTTCAAGTGGCC
10	25928920	SCAFFOLD300004_26688	0.25	0.21	ACAGAAAATTTAATTATTTTTATGTCAATATTTCTTGACATTATCATAGTTTCTCTTTCTGCTAAAAATTATCTGAAAGAAATTATCAAGGTAAAAGAAA[A/T]ATGTCCCAAACCATAAAGATGGCATAGTGTTAGTTGTGAAAATTTAATTTTGCTCTAATAACCACATGATATGATTGCTTTATCATTAAATTCTCTGTAG
10	26661442	SCAFFOLD270489_15691	0.45	0.40	AAGGTGGAAGTAGGAAAGACACAGGGAAAAAAATAAAAAAGAAAAGAGCACTTTTGCCTACCAGATCTATAGCAGAATCAGGGCTTTATGGTATACACAA[A/G]TGTCGAGCAGGCTCTGACAGAAAATGTACAGGAGTTAGTTTTAAATATAAATAAATGACCTAGGTGAATCATTAGCTGTGTAGCAGCTGTAGATAAAATG

10	26865889	SCAFFOLD10195_1494	0.50	0.43	GCTGTGTCCAGTCTGTTTTTAAATCAAGTCCCCTTAAATTTATAGCTGCACATATTTAATTTTATTTCAAAACATCCTTTGGTTCTTTCTTAGATTGCTT[C/G]TCTGTGAAAATTTTTTCAGTTTTTCAACTTTTTCATGTCGCTTTAGATTATACGAGTAATACTCTTTGCTAACAATTGAGCCATCCATAGTCTACTTCTA
10	33312366	SCAFFOLD10023_31672	0.26	0.31	AATACCAGAGTGGGTTGCCATTTCCTTCTCCATTTGATATCTCTTACAAAGCGCAACAACAATGACTCAAAAAAAAATCTAAAATGAGAGTTCTCAAAGG[A/G]TCTCTATTCTACTGGTTCAATGCAAAAGTTCAAATATGCCAGTGGGCTTATTTGTGGACTATGCCGGATGATTACAGAGCCTTTGTCGATGAGAAATGAC
10	35862278	BES6_Contig306_1185	0.41	0.48	TGGCCATATTGGGATTTGGGGCATTTCCCTTATTCTTTTCTGGGGCCAGTGCCATCTTTTCCTTTTATTCCTGTCCTTGAGGCTAGGATTTGGGGCTGGA[A/G]CTGATTGGTGGTCAAACCTTCCAAACTTGGACCTTTTTAGATAGCTGGGGATCCTCCACTTCCAGGGATCCCCAGTTACCCATCCCTTGATTATATGAAA
10	35928135	SCAFFOLD100339_6666	0.16	0.16	CCATTCCCAGCCTACTCACTAAAAACTGGTCTTTTGCAAATTCTCTCAGTCTAATTTCTTACCTGTTAAGTAGAGATAATGTCTGTCTTAATGGATTGCT[A/G]TAAAAGTGAACTATGGCCAGCACCAAAGTCTGGCTGCTGCTGCTGCTAAATCGCTTCAAGAGTGGGATTTCTGGATCATGTGGTAGCTCTATTTTCAGTT
10	35928330	SCAFFOLD100339_6471	0.17	0.15	GAGAGACTTTACTTTGACTTTTCACTTTCATGCATTGGAGAAGGAAATGGCAATCCCAGGGACAGGGAAGCCTGGTGGGCTTAGTTGATGGCATATGGGT[A/G]TGGGGGGAAAGAATCTACATACATAAAGGTTTTGAGCGAGGGTGACAGAATAAGAATTGGTTATAAAATTAGGATATAAAAACCAGCTTGCAGCCCATTC
10	36477788	BES11_Contig382_1421	0.43	0.36	ATTTACTAAAGGTAGTAGATGATAATACAAGTTCAAAGCGGTCTCATTTTATTTCACATTTCCAGAAACACAACTCCAGCACTGGAAGTTGCCATACACT[A/G]TATTAAACAAAAACCACTACAACCCTGTTCTGTTAATCCCCTTGCCCATGTAAAGTATTAGCCTGGAGCTATCACAAAGAATTCTAAAATTAAACCCAAG
10	37391101	SCAFFOLD10146_9294	0.20	0.27	TTTCTAGCACCTTACCTTTCTCGCCACCACTGTTCTTTCGAGCTGCATGGTCTTTCATGGATCTTCGTCTCCTACACCACTGAGTCTGAGCTGCCACACA[A/G]TTTTGTCATTTCCTTTATCATGTCCCTCAGGCCACCTCTCTGCTTCTTGCTTTGCTCACTTTTTTTGGTTGTTGTTGTTGTTGTAATTTTTTGGCTGTGT
10	37498857	SCAFFOLD113682_4331	0.06	0.03	TGGGGCTTTTCAGGTCATAAGGTCTCTGTCATTGTTATTCTGCTACTGTAGCAAAAGAGCATGGATGTGGCTGTGTGCCAGCAAAGCTTTATTTATAGAA[A/G]CATATGGAGGACTAGGTTTGACCCCCAGACAGTACTTTGTTGGCCCTTGCTTTACATTTTTCAAAGCCAGAATATTTGTATTCTGTGATACCAACCCAGT
10	37498925	SCAFFOLD113682_4263	0.07	0.03	CTGTCTGGGGGTCAAACCTAGTCCTCCATATGCTTCTATAAATAAAGCTTTGCTGGCACACAGCCACATCCATGCTCTTTTGCTACAGTAGCAGAATAAC[A/C]ATGACAGAGACCTTATGACCTGAAAAGCCCCAAATATTTACTATTTATGTAAGTAGATAAGATAGGGGCTCCCAGAGAAAGAGAATCAGTCATGGTTTTC
10	37498972	SCAFFOLD113682_4216	0.07	0.03	CTTTGCTGGCACACAGCCACATCCATGCTCTTTTGCTACAGTAGCAGAATAACAATGACAGAGACCTTATGACCTGAAAAGCCCCAAATATTTACTATTT[A/C]TGTAAGTAGATAAGATAGGGGCTCCCAGAGAAAGAGAATCAGTCATGGTTTTCTTGACATAAAAGAAGCCATTTTTCTTCCCCTAAGCCATTGTGTAATG
10	37501544	SCAFFOLD113682_1644	0.40	0.36	TAAATATTGTTTTCAATTATTTGATTCTCAAACATTTCTTGAATATAAACATTTCTTAGTTTTGCAAAACACATATTATCAAAGCCAACTCATTTTCTCA[A/G]CCCTTTGATTTCTGATTATAAGCCCCAACTAAGGAAACAGGGTCTTACTAAAGCTGATGGTCCTTTTCTTTTTCAGAGGAATGGCTGCTTGTTGGAACAA

10	38264639	BES10_Contig603_291	0.41	0.44	TCACTTTATAGTTATTCTTTAAGCTGTTAATATGCTTTATACAATCTTTTGTATAAAAGAGATGTACTTAAAAAAAAAAAATTGTAAGGGGCTGTCTCTT[A/G]AGGTAGCAGATGCTCCACCACATATGTTTAATCAGAGCTAAAGGTTCACATATCAGATGTGTTATAGCAGGAACTCAAGTACTCATCAGGAATTTGGACT
10	38265345	BES10_Contig603_997	0.19	0.21	TACTGCGATTTATGGGGTCGCAAAGAGTCGGACACAACTGAGTAATTGAACTGAACTGAACTGAGGTACTGCTCTACAAGCTTGGGAGATATTATACAAA[A/G]TAAATTAAAATCCTTTCCCTTATGTTGCTTACATTCTAAGGAATTTTTTCTTTAACCAAGCCTCATTTACCAGTGAACAATCAGTGCCTGTAAAATACAT
10	38481675	SCAFFOLD106386_649	0.48	0.49	CCATCTACCTTTAATATATCCAAACCCCTTCAAAGACTTCCAGAACTTTGACTCTTCAGAATGGAGACAACCACAAATGCCCAAGCACATGAAAGAGCCT[A/G]GCATCAGGACTTCAAAGAAATCTGTCCACATGCCTTTTAGAGTTTTATTTACAATAAGAGAATTGACTCGGGTAATTAAGTTGCATATTTGGGCAAAAGG
10	38481745	SCAFFOLD106386_719	0.49	0.48	TGTGGACAGATTTCTTTGAAGTCCTGATGCTAGGCTCTTTCATGTGCTTGGGCATTTGTGGTTGTCTCCATTCTGAAGAGTCAAAGTTCTGGAAGTCTTT[C/G]AAGGGGTTTGGATATATTAAAGGTAGATGGATGTGTCTCACATGGAATTGAATGCACCCCCCACTCCCTGTCAAGTTTAGAGTTCGATCTTATTACTTTA
10	38481810	SCAFFOLD106386_784	0.39	0.35	GGATATTTTTCTAAATTGACCCAATGAGGACATAGGAACTTTTGAGTTTCATATTGAGAGAGAGTTAAAGTAATAAGATCGAACTCTAAACTTGACAGGG[A/G]GTGGGGGGTGCATTCAATTCCATGTGAGACACATCCATCTACCTTTAATATATCCAAACCCCTTCAAAGACTTCCAGAACTTTGACTCTTCAGAATGGAG
10	38481901	SCAFFOLD106386_875	0.46	0.41	ACCCCCCACTCCCTGTCAAGTTTAGAGTTCGATCTTATTACTTTAACTCTCTCTCAATATGAAACTCAAAAGTTCCTATGTCCTCATTGGGTCAATTTAG[A/G]AAAATATCCTAGTGAGCTGAATAAATACTGATGAATTGTGTTAAGCAATTCTTTGTAAAAAGGTTACATTTCATCAACTTGCTTAAAAGTAGTAAAAAAT
10	38725566	SCAFFOLD106181_13619	0.37	0.46	GAAAGGCAAAACGGCATCAAACTTTTAAAGAGGTAGAGGGAGATAAGATAAAGACAGGAGACTGAGAGAGAATGAAACAGAGAATAGCATGTAGGGAAGA[A/G]AAAGTAAATAAGAGGAGAAACCCAGGGAAAAGGAGAGAAGCAAAGGGAAACAGACATTAAAATAAAGCTGAAATGCTATGAATAAAAATTACCCAAAGAT
10	41994949	BES11_Contig306_895	0.18	0.27	CATTTTCCTTCCCACATACTTGTATGTAAAGGTCTTACCATTTACCACTCAACAGTATTCATTGGCAGTGAGTAGGCAGCTGTTCAACACATTTATGGGC[A/G]TCATAAATCCAGCTGTTTTCATTTTCCATTACCCAGGTTCCTATAGTAACCAGTGACATTCAGGGCTGACTTAACGATTCACATGGCACTAAAACTGCTT
10	43736140	BES11_Contig425_1305	0.21	0.34	ATGTATATATAAAATTATGACTGGTTCATGTTGCTGTATGGCAAAAACCAACACAAAATTGTAAAGCAATTTTCCACCAATCAAAAAAAACAAACACATA[A/G]GGGATAGAGCTAGTTTTTCATAGGTTTGGTTTAGAGGTTATCATCTCCCCATTTCCACTGATTATATGGCTTGTATTAAATACCCAGAGTCTAGAAAAAA
10	44093123	SCAFFOLD135062_12754	0.44	0.47	AGATTTAACGTAGATAACTAACTTGTAATAGAAGCAGCCTATTATTAGAAATACTAGACCCTTTTCAAGTTTCTTTTGTTTCTTAGGTGCTTTTTATAAG[A/G]TTTAACTTCATATTCAGTAAGCTAAGGGAAAAAATACTATGGTATATGTATTCTTAATTTCATACAGATGCTCTAATTCCAATTCCCTAATATTTCACTT
10	44093322	SCAFFOLD135062_12953	0.01	0.03	CTATTTTAATCAGGAATTGTGTTACAGAGATACTTTGATTGTAATTCAATTTATTGTTGTGGACTTTGGCTTTTGTGTGTGGTACTAGTCTTTTTCCACA[A/G]TGCCGATTACTTTTAGAAGTTTTATTTTCATAAAATAAGTCAAGAAAACAGAAAATGAAGTACAGAAACTGTATTATGTTTAACAGGTATACATTTTATT

10	44361985	BES7_Contig511_2306	0.21	0.32	TAAATGCACATTTTCAAATTGTCCCCCAGAAAGTCTTCCCTGAACTTAATATTTTAAAACACAATTTCAAATAATTAAGTAACCTTTTAGCAGGGAGTCT[A/C]ATTAAGCAACCAGCTTCATTTTTCTGCTTGTGGTAGGAATTTAAGTCTGATTTTTAATCCTCAGTGACTTCACTCCTTTCAAGACATGGCAGAAAAAGAC
10	44711019	SCAFFOLD240152_6640	0.26	0.35	AGTCACCTATTCTCACAAGCTTTCCTGGTGCTTCTGCCCATTTAAATTCCTAATCTTCATGTCAGCGTCAATGCACTTGTTATACTGATAAGTCATCATG[C/G]GGAAGAACTGGCAGTCTGGCAGCAGGGGAAGCCAATACACTAGACAGTGAGACTAATTCTCTCCAAAGACAAATGCAGTTTGGTGGCAGACAGTGGAAAA
10	46415087	SCAFFOLD240566_6864	0.44	0.49	CTCTCTAATGACCGTGAACCACCCTGCCTGGAAATGCGTGTGCAGGCAGCACCCTGTCCTCCTCGACTTTTCTGATGGTCTGTGGGGGTGCTTATCCCAC[A/C]CTGGTCCTCCCATGGCCTGGCGCATGCGGTGAATGCAGAGGCGGCTGGAGTGCCTTCCGCCTGCACCTTGGAGCAGACATCAAATTAACTCTGGCCCTTC
10	47963182	SCAFFOLD295006_45298	0.48	0.42	AGGGCCAGGCATGTGTCAGAACTCCATCTAACATCTGTCTTACCATTTAGTCAGTGCTTATCTTTTCCAATAAGCCCATAAGGGCAGAGGCCATATGTAC[A/T]CTGCATGCTGTTTTAACTGTAGTCCCTGATATAGAACCTGGAATCACAGGTGCTCAATAAATACAGGGTGAATTAATCTAGACCAACCCCCTTACTTAAG
10	48243964	SCAFFOLD21136_2257	0.32	0.25	CTATTCAAAAGGGAAGTTGATTTGCAGTCTTTTCATACCCATCCAAGCCCCCTCTTCCTTTCAGTTGCCTAGTACCACCTGGTCCTCTCTCTACCTTTCA[A/C]ATCTCAAAACCTGCTCTTGCTTATATCTCTTTTGAAGGGGATCTGATTGCTCAATGTAGGGTTGTAAAAATGTCTGATGGAAGCTGGACATCTAGGGATT
10	48742585	SCAFFOLD295243_5984	0.33	0.26	TAATTTATAATACAGGGTTTTGCCTGGAGCAACATGGAAGCCCCGAAGCAGTGCTGTGAATTATTACAACTAAATCGGGCACTATCTGCCAAAATATACA[A/C]GATTGCTTGTATTTCTATTGCACGAAGGCTATAAACATGATTTCTGAGAGCTCTGTGCTGCAAGGACATCTAAATAAAATATCAGTTTCATGACCAGTGT
10	48742686	SCAFFOLD295243_6085	0.33	0.26	AGAGAGCATAAAACCTTTAGGCCACAATGTGTAGTTAATATTCAGAATTAAGTCAGTTGATTATGCATCAACTGGTAGGTCAAATTCAGCTTTGTGTGCT[A/G]TAATTTATAATACAGGGTTTTGCCTGGAGCAACATGGAAGCCCCGAAGCAGTGCTGTGAATTATTACAACTAAATCGGGCACTATCTGCCAAAATATACA
10	49803491	SCAFFOLD10405_7089	0.10	0.11	TGTTGGTTCCCAAGGAGATTTGCACAGTTAGTGTGAGGCATTTTGGGAGTAGAGTGTCAACCATGGCCATTTAGGAAGTCAGGAAAAGCAGACAAAGAAA[A/G]TAAGGTTGCCAAGGCCTCCCACATAAATAAAGAAGAGGTGAGGAGGGTAAGACAGGCTCTGAGACTATAAAGATGAGAATGGAGGGAGAGGGCTGGAGCC
10	49890208	SCAFFOLD95345_1310	0.38	0.45	AGATTGCTTATTAAAAATGATTTTATAATGAAGTTGCCTCAAATAGCAGCTCTTTTCCTGGTTGTCCTTGAAGCTGTGGTGAAGAATCTCAGAGACAACT[A/G]TCTAGGGTTTTCCTCCTTGTTAAAAACTTCTCTCTCACCACTGTTACTCTTGATTTTTAAGGAGACAAAGTCTAACTGACACAAGTCCCTCTCCAGTTGA
10	49892800	SCAFFOLD95345_3902	0.09	0.18	AACCAGATCATCAAATCTATAGAGTAAAAAGAAAAGAGACATTTTCAGTTACCATTGGCATCTATTGAAATGCAAAGGAGAAAGCTACCTACTAGAAAAG[A/G]GAATTGGCTGATCTTCCCAAGAATGGAGACTATGTTCTATCAAGGGAAATATCCTGGATCTGTCTGGTCTCTAGAGGAAAAAGCGATCTAGATTTGTTAA
10	51138334	SCAFFOLD125539_11512	0.25	0.44	TCACCATCTTCAGGGGGGTTATGTCACTCATTTGAAATACAGTGGGGTTTATTTATTTCTATTTGTCTTTAGTTTTATTCCCTCTACCCTCAGCTGCTTA[A/G]TTTTATTTTTTGAACACATACAACATTATCATGCTTCCAATGGGTAAAACTGTTCAAAAAGATATACTCAAGAGAAGTATTCCTTTAACCCATATCCTTT

10	51138432	SCAFFOLD125539_11414	0.11	0.25	AGAAACAATTCAGGGTTGGTTTGCAGTTGTTTTGTCTTTAGCCTAAAGGTGCATAATCAAACTGCTGCATTCAGAGTTACTTGGATCAGTTCTCCCCCTC[A/G]CCATCTTCAGGGGGGTTATGTCACTCATTTGAAATACAGTGGGGTTTATTTATTTCTATTTGTCTTTAGTTTTATTCCCTCTACCCTCAGCTGCTTAATT
10	51737658	SCAFFOLD67171_639	0.04	0.14	GGATGGGGGCAGCGGTGCAGAGTGGCCCCCATTCTCATGACCACAAAGGTGAGGGGTCCGGTGAAACTGTTAGGTTCTCCACCAAAGATACACTGTGCAT[A/G]CTCTGCTCCCACACCCGGGCTTTACTCCCAGAATTCTCCAATGAAACCACCCAGGAAATCACCTATACCAGAGGCAGGACAAGCCAGGCCTGCCCAGCAG
10	52599553	SCAFFOLD235193_12499	0.41	0.47	TGCTAAACCAAAAGTTTGAATTTGCAAATTTGTCTAATTCCAGTCCAAAGAAACTTGATGTTTATAAGCAGTCTGGGGTAGCCTGATCTCTGGTGACTAC[A/G]ACTTCGGGGAGGTTTTCTGGTAGAAGGTATCCAGAAGAAACCCAGCAACAGTTTTCTAGCCTCTCTGGAGGTCTGAGGGCCCTCTATCAAGAAGTATCAA
10	56497454	SCAFFOLD11903_9629	0.29	0.41	TAGTTGTATGATGGGTAATGACATTATATTTAGGAAGGTGCCATTATTCCATAGGTACCCTGTATTCTTTCAAATGAGTATGTTCACTGCCTTTACTTAA[A/G]GTACTGATAAGTAGTGTTTTTGTACGATTTCAGTGCCTGCATTTGTATTTTAGTATGCAAATGCTCTGGTGTCAGGAGAAGATCAAATGACCTGGGAGCA
10	56831533	SCAFFOLD99458_2134	0.40	0.48	TGATCAGAAGACATGGGGCATTAGATATGTTTAGAAATTATAAGTAGTCTCAGTGGCAAGAGCATGGAAGGTGTTGGAGAGTAGAGACAGAAGTTATAGT[A/G]GGGGTCAGGTTGCCGTTGGTCAGGCAAGTCACCTTAATGGGCTTAAATTTCTCTATGCTACTGAATGTGTGACAAGCAATTGATGGTTTTAAAGTAGTGA
10	57102100	SCAFFOLD155532_4758	0.20	0.07	TGCCCTACAGCTACATCTCTCAGATGCAACTAATGGGCCACAACGGTCAAAACTACATAAGCCACTGTGAATAACAGAGCCAATAATTCTTTGTAGTGGC[A/G]AAAAGATATGCCATGACACTACACTGTGATAAAGTAAAACAAGGAAGAAAGGACAAGAGGAAATACAGAGATACAATAAAGAATAAAATGTTGAAGAAGA
10	57303361	SCAFFOLD92148_4975	0.37	0.38	TTCTCTTCATGACGATGCAGTCACCATTAGGTAGCAGTGCTGTCTTCTCTCCATTACTGACCTGGCAAGATTGGACCTTAGATCTCTTGCTGTAAACAAT[A/G]TGGGAGGTGGTTCTCCTTGATGGAATTGTTGGCTTATGAATTTGAATCAATTATTTCAACTTTTGTCTTCAGGGTCCTGGTAAAGAGGGACTTTTTAAAG
10	57303509	SCAFFOLD92148_5123	0.02	0.11	TATGCCTCATGAGGAAGGGCAGCCAAGGGAGGGCATTTTTCAGGCCCCTTCCCGGTTCAGAGCATCCCAAAGAGGTGCCAATCGTCATGATGGCTTCTGT[A/G]CCCATGGCCATCTGCTTACTGGGCTTTGGGCAGATGCCAGCGTATTTTTCTCTTCATGACGATGCAGTCACCATTAGGTAGCAGTGCTGTCTTCTCTCCA
10	59150258	SCAFFOLD10521_8032	0.19	0.22	TCCTGATTCTCAATCCAGGAATAGGAAAATCTAGATTTGTTTTTAGAAACAAATCAGTGTGCTACTGAAAACCATGTGCTGGCTAGTGATGTATAGGAAT[A/G]GTAAAACGGCCTCAAAATCACGTCTTACAAATCTCTTTGTTTAACATCTGGAAACTCTTCCCATTCAGAAAACCTGTCTGATGAATTTTATTTCATTCTC
10	59669114	SCAFFOLD206177_14785	0.03	0.07	CAAGAAAAACATAGTTCTAATCAATGGTTTCCATTTTGAACATGCAAATAATTCACTTCACAAGTCCATGGATAAAATATTGCAGGAGAAACATTTTGTA[A/G]TGTGTTGGTTTATCAGTCAGGTTAATAGCAGTTCACTTTCAAACACTCAATACTTCAACCCTTTATGTAACAAAACTTCTAAAATTCATTTAGCTCTTTT
10	60001420	SCAFFOLD195517_10504	0.44	0.45	TACGTCACCACATTAAACATCACCCCCACCCTCTGAGATAAGTACTGCTCTGCCTGTTTCACCGAGGATGAGACAGAGGCTCGGAGAGGCTGTCACTCGC[A/G]CATGGATTGTAGCCATCAGATCCCGGGCTAGTTGACTCCAAGACCTATGCTTTTTGTCTTCTGTTCTCTTCTGAACCTGGAGGGAGTGCTTACTGGTGGG

10	61645811	SCAFFOLD5514_22949	0.30	0.39	GTAAGGGAATCCTCCTCACCATCATGAAAATTTACAGTCCCAGGTAATGAAGAAAGCAAGAGAGACAAGTTCCTCTGAAACGAAAATATTTCTCTGACAG[A/G]TTTGCAGAGCTCATGAATTAGGTCCTCAGAAAGCACCAAAGGGCCAACATCTCCTGAGGTATCCTTTTTAGTAAAAAACTTTAGTAAAGTTCTCACTTGC
10	61648017	SCAFFOLD388314_456	0.25	0.26	ATTGTTTAAATGCTTTTCTCAGGGAGAGTATCACAGTTCCTGTTGTTCAATTAAAATAATGCAACTGGATGGATGTACTGCAATTATTGCTCTTCCTAAC[A/G]TTTGTGATTTGCAGAGCACAACCTGACCTTCATATAATTACCACACCAAATTACTGCCATTAAGATACCTCTAGTGGTTTTTCTAAATAGCAGCATTTTA
10	63707096	SCAFFOLD5120_2929	0.02	0.05	TTCAACCCCAGGCCAAGGATATCCAGGATTAAACTTTCCAAGATCTTTTCTTTCTCTCCATAGTAATACTGTATATCAGCTGTTGTTTCTATCATATAAG[A/G]GGTCAGGGAAAAGATAGCATCTCATTGCACTGCTAGTCTTGTTTTCATGCCTCTGAAATGTCTTCATTTCAGAGTCCCCAAAATGATTTCTGACAAGATC
10	63707270	SCAFFOLD5120_3103	0.48	0.05	CCTGGATATCCTTGGCCTGGGGTTGAATATCAAGGTGGAGCACAATATGAGTTGTGCATATTAATTCCTGTGCACTGCAACCACTCAGCCCGCGATACCC[A/C]CCCTGGTAATTACACCTCCCCAAGGCAGTGCCCCCAAAGCACTGTTACATTAGGCAATTGTGGCAAACTGGTAACCTGATAGCAGCACATTATGTAGTTA
10	65664708	SCAFFOLD306009_7185	0.13	0.19	GGAAACAAATATTTGCCACAGCTTGGAATTCCATGCCATGTTGTCTGTACATAATAGGTGCTTAAGTTCTTCCCAAAATATTGATGCTTTTCTAACTTAC[A/G]AGGACTCCTGAAACTGGCCTCTCAAACGAGATCACCTTTCTTGCTCTCAGAAATGCATGCTGAGGTTCACAGGTACTCCTATTTCCTCTGAAATTTATGT
10	65963858	SCAFFOLD125279_19173	0.29	0.25	TGATCAAGTTATCATTTGTCATGATCAAATTGAATTATGAATATTTCAAGTCTTTTTCTCATAACAACTTCAGAAATGTTCATTTTAGTATTTTTGTGTT[A/G]GGTTATTTTTCACTGGACTTCTGTGGTGGCTCAGAGGTAAAGAACCTGCCTTTCAATGCAGGAGGTGCAAGTTTAATCCCTGAGTTGGGAAGATCCCCTG
10	67485298	SCAFFOLD145288_4171	0.38	0.32	TATATCATATAACATATGTTCTTTGACTTCACTAAATTAAAACTCAGTGAGAGAAAGATAACTAGAAAATCTCCAAATGTTCAGAAATTAAGTAGCACAC[A/G]TCTGTATAACCAGTGAGTTAAACAAGAAATCTTGGGTAAAATAGGAAAATGTTTTTATCAGAATAACAATGAAAACATGACACATGCAAAGTTGTGAAAT
10	70341719	BES8_Contig406_894	0.40	0.45	CATCTGGCCTGTGTTTAAATGATCACATTAGGAAAGCAGCCTGTGGGATTTCAGCTTAATTACCTATAACATACACTATTCATGCAAAGAGGAATGGGGG[A/G]AAGTGAGGGAAACTAATATTAAAAATGAATGCAATGTAATGTATTGCTCCCCATCCCCCAACCTTTTGGTACAAAGACAAGTAAAGCAAAGAGGTGCAAA
10	71398100	SCAFFOLD272440_2754	0.18	0.28	CCAGCTTGACAGACAAGGGCAGTACCAAGGGTGCTCATGCACCAGTCTGTGTGACAAGTTTGGAAACAGAGCCAGAGCCCAAGGCGAGGCCATATCCACC[A/G]TGGAAGTCTTCTCAGAGCACATCTTCTCTTCTGCTGGCATGAAGAAAAGCTTTAAATTTTTTTTTAAATCATATAATGAAAAACTACATCACATTATTTC
10	71398236	SCAFFOLD272440_2618	0.19	0.29	TTCCAAACTTGTCACACAGACTGGTGCATGAGCACCCTTGGTACTGCCCTTGTCTGTCAAGCTGGGCCACAGGTAACTTTCCAACATTTTAGCTGGAACC[A/G]CACCTGCAGCTCTGTCAGAACTCCTCTGTGTAAGTTTCAGAGGCACAGTGATTTTGGTTTGTTAGAGGTAGTGCCGTCATGAGGTCGTCTGGTATTTTAG
10	72073023	SCAFFOLD136763_992	0.23	0.26	GATAGAATTACAAACAGGGCAATGGGAATTTTATAGAAAAAAACTAAGAATCTTATCTCATGCTGGACATGTTCATGAAAGTGTCATTTATTCATTTAAT[A/G]CATATTAAAATCAGCCACATGGCAGATCCTGTGCCAAACACAGAGGACACAATAGTGAGCAAGACAGAAATAGTCTCTACCTGTAATGGTGCCTTCCCAT

10	76600139	SCAFFOLD16316_2366	0.16	0.19	GCTAGCAGAGTTTGAATATTTTAATTCTCACTGAGAAAAAAAAATAAGCAGTTTCCTTTACTATTTACATTTTAAAGCAACCACCAGATTTAATATGATT[A/G]ATGTATATTACATAAGAGTGATAATGGTCTCATCATGGGAGTTTTATAATTTAGCAACTTGATTTTCCATAACTTAGTCTTCCCTCTCATCTATGACCCA
10	77594015	SCAFFOLD210139_7598	0.22	0.21	AAAACTGTCTTTTGGAGTTAATGGAGAAGGAAACAGCAACAGGATTTGGTTATTATTTGGAAGCAGGCAGAAAAGCTGAGGAATGAGCCATAAATGAACT[A/G]ATTCAATAGATTATCATGTAAAACTGGGACCAATAAAATGGCTAGTGTGAGCAATGTGTTCGAAACAGAGATCAGGGAAAGAGTCTAGGATTACAGGTAA
10	78927778	SCAFFOLD107047_3609	0.36	0.44	GTTTTTTATCTTGACTGGATCAATATCAATACCCTGGTTGTGAAACTGTACTGTAATTGTGCAAGATGTTACCACTGAGAAAACTGGGTAAAGGGTTCAC[A/G]GGATCCCTCTGTACTATTTCTTAGAAATTCATGTAAACCTGCCACAAAATTTTAAAATTTAATTTTAAAAAGGAACAAAAGAAAAGATATAAGCAACACA
10	79625237	SCAFFOLD105461_1724	0.29	0.40	TTTTGTTATTAGTGGCTATTAATATAAAGATATTACCAAGTCCTACTACAGGGCAATCAAACATGTGTGGAAAGAGGCAAAAATAAAAATAAAGCCATAT[A/G]GAGTTACATCTGACCCTTGAACAATGTGTAGGGGTTAATCCAAGTGTAATTTATAGTCAGCCCTTGGTAACTGCAGTTCCTCCACATTCACAAATTCAAT
10	79933253	SCAFFOLD311992_936	0.48	0.40	TGGTGCTGTACAGCAGCACAGCATAGCCTACAACCCATTTATTTGCTGAGCATGGCTTGAAAACCCACCCAGTTTGAATGTCCAGGTAACCACCTCACCT[A/G]ATCATAAAGCATAACCTATGGCCCCACCCAACCAGTGAGTCCAAAAAGCAACCCCTCAACCACTGAGCATAGCCTCCAGCTCCACCCAACTGTGGGGTAC
10	82562803	SCAFFOLD150365_14014	0.36	0.44	CACCCATGAAGTCATCTGAAATCCTGTCTTGTGTTTCCTTCTTGTCCTTTGCCCTTTATTTCACTGGAGGAAGGCTTTAGGGAAATAGTAAGAATGCTGC[A/G]GTAACAGAGGGAATTAATTTGACCCTGCTCTGAAGAAACAGTGTGTTGATTCCCACTAGCAATAGTTTCTCCACCCTTCAGAACAGGGCAAGGGATGTGG
10	83499488	SCAFFOLD155430_14295	0.31	0.34	AGGCATCTCTGCATCTTGCAGGTGAAAAAACAAAGACACCACGACTCGGCATAGATGATTTGTATGTAATGAGTAAGCACTGGCTGAATGGGTGAATCTT[A/C]TGTTAGAAAAGCGCATCACCATGAAGCCCAGGTAACAGGTCACCTTGCACGTGCTTCTGTCACTTACAGACCCCTACAGCTGGGACTGGCTGTGGTTATG
10	87622925	SCAFFOLD5614_3703	0.26	0.22	ATGGTTTTTGGGGTGGCCTTGAGCATCTGTCTCCTTTACTCAGGATGCATGGTGAACAACCTTTATTTGGAGATTTCATTTCTTGCGAAGAAACCCTTCT[C/G]AGAGTCCCATGCAGAGAGGTGCTGGGGCTGATGCTCGGCTCCTGGGTGGGGGCCATTGGCACTAGTAAGGAACCCAAGCATGGACTGACTTGACCCAGAT
10	88263593	BV104999-587-Y	0.45	0.48	GGCCGCTGCGTGCGCGTGCGCGAGGGCTACACCTGTGACTGCTTCGAGGGCTTCCAGCTGGACAC[A/G]GCCCACATGGCCTGTGTGGGTAGGTCTGGAATCAAGASTGCTAATGCCYGGGGGYAGGACGGCCCTGCTGGATCCATGTGGAATAGTCAGACTGGCCCAC
10	90353061	SCAFFOLD205616_3089	0.28	0.36	CCCAGAGGAAGGGGCAGCTAAGCAGGAGTGAGGAGTGAAAGTGCTTGGCAGAAAGAACAGCAAAACAGGCCCACAGGAGTTGAGTGCCCTCTGCTCAGGA[A/G]GTGAGTAGGTAAGGGGCTGAAACAGAGCGTCAGACCGGATGTCAAAGGGCTTCACAAGCCCTTTGATGGGAGGGCTTCAAGGAAGTGAAGGAAGTGAAAG
10	91618684	SCAFFOLD72801_339	0.44	0.29	CGGGGCTTATTCTTCCTGGCTCAGGGTGAGCTCTTGCCCAGGCGTCTCAGTTCCTCGTGGAATCGCCTCCCATCTTCCTAGGCTGTCAGCTGGCAGGCCT[A/G]TGCAGACTTGGCTTGTGTCCTTGTGGGGAGGGGCTCACGCTTGCCGCGAGGGACTGGATTCTGCTTGTGTGGTAGAGGGCCCTGGGCTCTGATTCCAGCC

10	92552333	SCAFFOLD155540_1585	0.38	0.43	TTTGTGTGTCTGTGTGTGTGTTGTTTGTTTCATTTTAGTTAAAAGGAGAAATGTTATTTTCTATATTGATGAGCTTTGGTTTGAGCAAGGAAGAGATGAA[C/G]GTTCCTAGGAGCTGTGGGTCTTCAATAATGGATACAGTCGACAGGAGTACTTTCTCTGCCTGAGTAACAGAGAACCCATGATGACTGAGTAGTTCAGAAA
10	92605461	SCAFFOLD27815_931	0.28	0.29	TATGCATATTTCTTTTCCAGACCCATGTCTACATATGTTCTGGAATAAAATATGTCTCCATGTCATACATTAGGTGTTAAGCTGTTTAGGCAAGACTTGT[A/G]CTCCTACATAGAATTATGGTAACATGAATTGCTTATCTTCAAAGGAATAGGTTAAGCATATTTGTTGTCTTTGCCAAGCCTTTTCTCACTTTGTAGACTT
10	96040233	SCAFFOLD260711_6055	0.07	0.12	AAACTTGTAGGGACTATAAAATCCAGTGATGCCCAATCCGAGTATAGAAACCTCCATCATGTTATATCAGAAACCCACTGAAGGAACAAGCTGTTCAAAA[A/G]GAGCCCAGTGTGTTAAATTCCAAATCCTTGGGCATAGTTTGAAAGCTGGAACCAAGTTTAAACGCAGAGTTTTGTGTATTATCTTGAAATTTGTAGACCC
10	96074573	SCAFFOLD50205_19130	0.07	0.02	AAGAGTCACCTGGGAAACTTGTCAAGAAAAATGTAATCCTTGGAACCTGATTCAGTAAGAGTTGATGTGACCCAGAAAATATGAATTTTTAAAAAGTGCA[A/G]CAGGTGCTACTGCATATACGATATCCTCAAAGAGATTTTGAAAACTCTTGGCAGACAAGGAAAGCAAGGTCTAGGGACTCATTGACTTGCCCAAGTTTAA
10	99805528	BES4_Contig352_1404	0.37	0.19	CTTGGGACAGATATGACCCATGGCATTATAGTGTCTAACACTTAAAATATATATAATAACTGATTATTTTTCATCTCAGAGAATCCCATAGCTTGCTGTA[A/G]GGCTGTCATCTTATTCTGATATGCAAGAATCTCTAGAAGAAGTATGAACTCTAGACAAGAAAATGAAAGGTAGTTTCAAAGCAGAGGACTTTTTAAAAGA
10	99923199	SCAFFOLD126125_3685	0.49	0.44	GAATCTGGGAACATCTATCCTCCGGTGGATTTCCTCTATTTATAATAACTCGGGTTCGGCAAGATTCAAGTGTTCTTCTCTGAATGGCTTTTAAAGGCTC[A/G]GTCTCTCTTAGTTCGAAATGGAGATGGGGAAGGAAGGAGTCGGTTGGGGACGTCTGAGTGTGCGCTCTGAGTGGCCCTTTGGGGGCCGACGCTGACAGCG
10	99937682	SCAFFOLD15241_2094	0.31	0.24	GTTAAATGGAGCGAAATTTCCTAGGTGGCAGAATATATAGAAAAAAATACTTTCAGAAATTCACAAGTGACTTTAGCCAGACAACTATAAAACCACTGAG[A/C]CTCTGGCTTAGGAAAAGTTCTAAAGATGATCAGTTAGTGATCTCCAGTACCGGGAGCATCCACATTCCCAGGCTTCACCACAGACAGAGGATATCGGCAT
10	100255297	SCAFFOLD245184_4736	0.13	0.23	AGACTAAAGAGTCACAATTGATTTGCAGGACATGCAGAACGAGCAAGAAATCAGTGTTGGTTGTGGAAAGTCACTGGGTTTAAGGATTCTTTGTTACTAT[A/G]GCATAGCTGTACATCCTGCCTGAAAAAGACGGTAAGGCATGCTCTATTACCTTTACAGGGTTGGGTTGAAAAATCAAATACAGACATGTAATTGGAAGCT
10	102561237	SCAFFOLD152310_1829	0.19	0.15	TTCTGGGAAGATTGCTGGTAACAGATATTAACGTAACACAGTCTCTGCTTCTCTCCCCCCTGCCTACTTTTTCATGTCTTCTTTCTCCCTGACCTACTTC[A/G]CCTTGGTAGCCAGGACAATAAACAAAGGCTGTATTATGGATTTGATAAAGCCTAGTAACACAATGAATCATTATGTACGTTTATAACCATTCTATCTTTA
10	103244624	SCAFFOLD201023_3793	0.29	0.34	GATCATCTGGTTCTGGCTTAATATCAATCACATCTTCAGAGGGTGCTAGCTCCCTCAAAGGTTTCACTGTGGACATTAGTCGGGTTGCAGCTCCATCATC[A/G]TAAGTCTGTCTGTTAAAAAATGAAAACAAAACCAAAAATGGTGTGTTACTAGTTTAAGGCAGCATCTTCCACTATTCATATTCAAAGACTAGTCAGATAC
10	103936503	BES3_Contig376_957	0.48	0.47	TCCTGTTTGATTTTGGTTTTCAAAAGCAGAACAATTTTTACATCATTATATAAAGGCCTTCCACTATTTAAAATGTCGGTGCTAAATTAATTTTATACCC[A/G]CAAGGCACACTGCATCTGACCACACTGGATTCTATCCACTTAATAACATGAGAGAAGATCTTGGACAAGAATTTAGTGAAAGGAACAGTAAAAACAAAAA

10	104007631	SCAFFOLD248438_6644	0.18	0.32	CGTTTGTGGTGCCAGCGTTGGGTCATTTCTTCTGGTCTGAGCCCACGGGTCTGTCTGTCATGTATGGAGAGCTGATTGCTTTTTCTACCCTGGGGTTTTT[C/G]CTCGGAGAGAGTCCTAGGGAAACACCATCTACAGCCTTCTTGCATGGAGGAGAACAGCTCAGGGCTGAGCGCCAGAAGCCTGCAAGAGAAGCCGGACGAT
11	593611	SCAFFOLD10217_12574	0.44	0.36	GCTTTGTCATGGGCGTGTGTTCGGGATCTTACTCTGTAAAATCTCCTTTCACACCCGGTTCCTTTGTCCTCCAGCACGGCAGCAACCTGATAGGGATGTC[A/G]GACCTCAACGTGCCCGAGCAGCTCTATCAGTACATGGTTGGGGGCCACAGGCGCTTTCAAGCTGGGATGCACAGAGAGAAACATAAGTCACCGAGTTACC
11	1801183	SCAFFOLD135323_1575	0.34	0.41	ACACTTGCTGCCTTTTGACCACCACATTGTGACACAGCTTCCAAATGGTCTACTGGGGTCCCTCTGGCCACCCCTCCAATCCATCTCCAGTGGGTAGCCA[A/G]AAATCACCCTCAGATGTACATCTAAGTCATGTCACTCTCTTCCAACGGCTTTCCTGTTTTTAGGACAAAGACAAAAGCTTGCGGCTTACAAGCTCTTACA
11	2603118	SCAFFOLD175947_14270	0.49	0.38	TCAAAATTAGCAGGGCCTGTTTCTTTCCAGGCAGGAAGTCAATGCCTCCTTATAGAGATCACAGCCTCACGGACAGACAACAGACTTATAGGGAGTGAAA[A/G]CCACCCAGGACCTCCGGGATCCTTTGAAAAGGGGCATTGATCAAACCACCTAAGTCCCCAAAGGCTCTCTTTTATGTGATTCAAATATCACTTATCAGTG
11	2676384	4013-295-Y	0.32	0.20	TTCCTGTGCTGTCAGCAGACATGACTTATTGTGTGAAAAGAGGAAGACCCAGGTGCTGGTGATGACAGCAACAGGTCAGTGGTCTCTCAACAACATCAGC[A/G]TGAAGGCACTGGTTCCACTCTGTACCGAGGACCACACTSAGAGCATCTGGAGAAACACTTCACTTCTTCAACTGCCTCCTTTAATAGGAATTTGGCTTTA
11	2970042	SCAFFOLD140359_23711	0.17	0.19	AGCACTGAGCACCCTGCACTCATGTTTGTGGCCTCTTAATACCAGTTCCCACTAACAAGAATAAAAGCTTCCTGGAGAACGTGTCTGATGCCAGGTCTAA[A/G]GTAGGAAATACAAGCTAAAAACTTCCCAGAAAAGCAAAGAAGATATCAAAAACTTATAGGTTCTTGTAAAAGGACTCAGGAGTCAACTTGAATAGGCTCC
11	2971978	SCAFFOLD140359_21775	0.17	0.18	TACAAGTAAGAAGAAAACCAGTCTTGGGTGGAATATAAGTACCCAGTGCTGATCTCTGGGAGGGTTCCAGTCAGGCCCAGAGTGTCAGAGGGGCCATTTC[A/G]GACCCACCCCAGATGGGAGGAGGATTCCTGGAGGCAGGTACTGAGGTCAGTCACTTCTGGGCCTTGTCCCTCGAGTGACCCTCTGAGCGTGGGCCCTCGG
11	2972387	SCAFFOLD140359_21366	0.17	0.19	CCCTGGGGAAGCCCCTGTGTGTATGCATGTGTGTGTGTATATACACATACATACAAACACATATCACCAACTGCCACGTAAACTTCCTGAAAAATCATTT[A/C]TTATGCTTCTTTGTATGTGTCCACCATAGCTCCACCTCTTCCCCCACAAGGCAAGTACTAGGTTGGCCAAAAAGTTTTAATTTTCCATTAACAGCTTATG
11	2973125	SCAFFOLD140359_20701	0.15	0.16	TTGGTCTCCTGTCTTCTGTGCCCTGTCTTGTTTTTATAATAACCTGCTGGACCCTCTCAGGCCCAGAAGAGGCGGTATTCTCCGGGAGTCCAGGTACTCA[A/G]TTCTTGACTCACTGTGGCCATCCCCGATTCCCTTGTGTGCCCATCCGACATTTGCGACTGTTCTCAGTCTGTGTGGCCATTGTCTCCAGAGTCACCAGTC
11	2973217	SCAFFOLD140359_20609	0.14	0.20	GTCAAGAATTGAGTACCTGGACTCCCGGAGAATACCGCCTCTTCTGGGCCTGAGAGGGTCCAGCAGGTTATTATAAAAACAAGACAGGGCACAGAAGACA[A/G]GAGACCAAGTCAGAAAGAAACATCTGTGCAAGAGCTATGACCCAGCTGCAGTGGAAGAGCACCAATCTCAATCCCCAAACCAATCTCAATCCCCAAATAG
11	2973443	SCAFFOLD140359_20383	0.16	0.19	CCACAAGTCCTCAGACCCCTCACAATTATAGGCAAAAATAATTGTAGGCAAAAAACTACACGAGCAGGTGCTTGTACATTTTCTAAGGAGAAGGTCTGCT[A/G]CTATCATCAGATACTCAAGGGGGTCCATTAGGAAGACCTCTCTAACCCACACAGGGTCCTAAAACTTTCAGAACTTTATAAAAACACCCAGCATCACACT

11	3219718	SCAFFOLD31034_2838	0.03	0.07	ATGTCTTTTGCTTTTTTTTTTTGGCCGCGCCCTGCCAGGCTTGCAGCATCTTAGTCTCCCCGCCAGGGCTAGAACCCAGGTGCCTGGCAGTGGAAGGGCC[A/G]GGGAAATTCCCCTCTCAGTGTTTTACACACAGGACTGGGGAGAACAGTTTGGTTTTCCTGCGTGATGGAGGGGGAAACTGAGGTGCTGACCAGTTTTGAG
11	3828302	SCAFFOLD288551_777	0.20	0.27	CCCACCCACACTCACCAGGGCACGGGTAAGCCAGGACCTGCACCTTCCTCTCACCCGCCATGGGGAGAGGCAGCGGCTGATTGGTCCCAGGTGCAACTGA[A/G]TCTAACCTGAGCTTTCTAGGAAGATTGCATCTTGGAAGGCCTTGAATCCTTACCAATGAAAGGAAAGTAATTCCTTCAGATTTATGACAGGATTGAATGC
11	4684784	SCAFFOLD276577_2702	0.08	0.03	GTTTCTGTTCCTTTTATCAGTTTCTGAACCAGTGGAATTAAGTGCTGCTGCCGAAGTTGTGGAGCAAGGACTACCCGAAAAAGAAGAGACACCGCCTCCT[A/G]TTGAACCAGGCCAGTAGTGCTCATGTGTCCTAAGTTGTCTTGGTTGTCACTAGTTTGGAGAAACCAGATGTCTGGCAGAATGGCCTTCTTACTTTGTAGT
11	4911850	SCAFFOLD273657_2601	0.41	0.45	ATGAAGAGGAGAATGAACAGGTAAGATTTTCTTTCATGTTGCAGTTCTTTGAATGTGACATGGTAACACTGACTGAGTAGACGGTCCTGGGTGTTGAGTC[A/G]TATGGATGCTAGATTGCAATACCCTTGGCTGCTGGGTGTCCTCTGCTGATGACAGGATTCTGTGCCGTTCAGCCAGTCTGTTCAAATATAAATATCTTGG
11	5205297	SCAFFOLD291452_7375	0.28	0.29	TAGTGATTTCTAACCTCCCAGCCTGTACCGAGGTGCAAGATCCCCAAGGACAGGGACTGTTTTGACAGTCAGTGTTACTCCTGGGATCATCCACATGGAC[A/G]TTCCTTAGTGAAGATTTCCTGAATTGCATCAGATAGCAAACCTTCCAATACCTGGAGGATAGTTACACAGTTCCACCAGTATTCCAGGCTCTCCATCTTC
11	6474700	SCAFFOLD212128_13243	0.41	0.35	AAGAGATGGTGCAGCCGTGGTCACCTTGTGATGAGACACGCACTGTGTCCCAGCCGTGGTGCTGGATGCAGGCATTTCCACAGGCGAGCTACATGCCCAC[A/G]TGCAAAAATGAGTTTAAGGAAGGCTTGGGAAATCCGAGTGAGATCAGTGGATTTGTATCAGTGTCAAAACCAGATTGTGATATTGTACTAGAGTTTGGCA
11	8202166	SCAFFOLD221057_5880	0.18	0.33	AAACAGCTTTGAGAAGTAAAACGCTGTTCTCTTGGCCTGCTGAGTAAATATGAATTTCACAGTTAAATACATAAAATAATGATTACATCCAATGGTCACA[A/G]GGGACATGTTTTTAGGAGGATACTAATTAAACTACATCTTCAGACAACATGTTTCTACACTTACTACCAATGAACCATATCTGATTGGTCATTAAGTAAT
11	10515118	SCAFFOLD200640_6150	0.37	0.23	TCCTGAGGTAGCTTCAAGGTGGGGAGTGGGCTTAGGTCTGGGAAATCAAAGTGCGATGTCACCCTTGCAGGGTCCCGTGCTCTTCTGTGCAGCTGTGGCA[C/G]GGAACCATAGCAGCAGGCTACGGGAACGGGCAAGTGCGTCTATATGAAGCCAGTACAGGAACTGTACACGTCCAGATCGATGCCCACGCCCGGGCCATCT
11	13169935	SCAFFOLD84361_971	0.47	0.09	AGTCAACGTGGGTGGTGCTCAGTGTCAACTATGTGTACAAAGGCAATTTCTCCCAAGCAGACACTTGGGAGTCATTCAGCCAAAAATGGTTGTCTCAGAC[A/G]CAAAGCACGTCAGAGCTAGGAAAGACCTCTGAGAACTTTGAGGTTCAGCCCCCATGTTTATAGATCAATTCCCGTGAGATCCTCCTCCAGAAGGATTTTC
11	15996682	SCAFFOLD161755_10008	0.25	0.19	ACTCTTGGCCAGTAAAGCAGAGTTACCTTATCTAAACAGCAATTTAGATGAATGGGGATGTTATACAAACAGAAGTGGGGAAAAAAAGAATACTTTCCAC[A/G]AGACTCACCTAGGGTTTTAACCTAGGAGACTTATCATAATTTGGATAAATGGTAACAAATAGCCCAAACTAGCCAAACTTCTTATGTCACAATGTTTGAA
11	16684267	SCAFFOLD140043_24985	0.32	0.42	CAGGTGGGTATGGCTGCCTGGCGTGGGACGCTTCTCCCCATGTCCTCCTTCAATACATCCTACAAGTTGTGGATTACAAATTGTAGGTTTTGTGAAATCT[A/G]TATTTCTTTAAACTGCACTCTACCGCTTCAGCAATCTGATGTCATTTATATTTCAATGAGTGTCAAGTGGCGGCTGTACTGTGTAACCTGTAAGCACATA

11	19241127	SCAFFOLD131620_2609	0.08	0.12	TAGAGCAAACAACTGAACAAATTTTGGGGATCAGTAATCCAAAATAAAAGATTAGTATATATTACCCAAGTCACAAATTCTTTATAGATTTCCTATCCTG[A/G]AAAGGAACTGATCTCAAAATATAGAAAATTCAGACCAGAGTGGGGAAGGAAGAGGAAACATCTGAATAAAATGAGGAGATGTGACATATTCTAAGGATTT
11	19734848	BES11_Contig184_366	0.12	0.19	TTTAAAGCTTAAATCACCAATTGGTATATTTTTATCAAGTCCCCATTTAAGGATGATGTAAGGTGACACATACCTCTTTTCTTCAAAAATATTTTTTACA[A/G]TTTTAGAATTGCAAGTTGTCAACATTTGTGTGTAAATATTTCTAAAAAAATTTCATAGGGAAATTTGAAATAAATCTACACATATAATATTTAGAACGTT
11	19872606	SCAFFOLD136019_1945	0.17	0.11	TGAGAAATATGGTTATTGAATGTTTTACTACGTCAGGGGTTAAGGATGTTCAGAGTTGGATAAGACCTGGCTCTTGTGCATGAGGACTGTATGTCTAATG[C/G]GGAAGTTTGACTTATATGAAGCCATTACTAGAAAGGATAAGAGGTGTGCACAGGTATTTATAGGAACATGGAGGATAGAGCAGCTCAGAGCTGGAGAAGT
11	22774051	SCAFFOLD317126_4807	0.44	0.45	AAGGAAATGCTCAGTAGATAACAATTCTCTTTCCCATTTCTTTACTAGTCTCCTGAAATAATATTTTCTGATGGCATACATCTTTAAAGAAGTCCTCAGT[A/G]TGACCAAGTACCAATTGGGGAGTTAAGAATAAGTCTACAGGAGAAAGATAAACCCACTGTTTATTGCTTATTAAATGCAAAACATTCCCAATTATTATGG
11	25397320	SCAFFOLD1150_5094	0.20	0.24	GGCCATAAAGATTTGGAGAAATGGAGCCACAGAAAGTAGGAAAGCTTGATCAGGAATTAAAAACTCCTAATCCCAGAGAAGTCAGGTTTCTCTTTATACC[A/G]AGTAAATCCCTTAGTTTTCCCTTCCCCATTTTCTCAATAACACTGCAAAGAAGCTTTAAGGAAATTGCTAATATTGTGCATTTGCCTGGGGTAAATTAGG
11	25489880	SCAFFOLD285043_26411	0.05	0.15	CCATCCCCTGCATGGAACATCTTTTCCCCTTTTCCCGGATTTTTGGGTAGCTGAATCATTTCCATCATCCCAGTGATTCCTTTAGGATCATTTCCTCAGT[A/G]AGAATAAGATAGTCTAAGATAGTCCCTCCCTCGACCAACCCTGTTACCTTCTATCCCATGAACCTGCATATTTTTCTTATACAATAATAAATATATCAGA
11	25819737	SCAFFOLD302888_550	0.33	0.32	GGAGACCCTTGTGCCCTTTGACCCTGGGCTATAGAGGCATCTATCACGTGAGGGAGTGTGTGTGTGCGCGTGTGTTGTGTGTGTGTCAGAGGTGGGCTGG[A/G]GAGGGGTCACAGAAGATGGATGGAGACATCCCGCCCGGGCAGTCCCAGAGACCAGAGCAAGCTCTGAGAAGTGCCCAGTGGGACAAACTGTCCTGGGCCT
11	25839729	SCAFFOLD296989_1191	0.38	0.46	TTCAGAACCTCCCTCTGGAGTTGCTCATTGCTGTGGCCCACCTGCTGGTCAGGACCCCCTCTTCCCAAATGCTAGGAGGTCCTCCGCTCATCTCCCCCGC[A/G]GCCGACTGCTGCCGAGCTGCCACCTGTTGTCTGTCCCCACACCCTTGCGCACTGCGTTCTGCCTGTCACCCTGGACGGCTGCCCGGCAGCTGCAGCTGAG
11	27663187	SCAFFOLD65017_16054	0.03	0.02	ACAAATTTATTGCTGCTTTATATTTATTACTTCCATAAACTGCTTTTAGACAGATCAGACCCTGAATTTGCAAACCCCGCTGTGTTATAGAATGACAAAC[A/G]AGGCTGCCTCCTTGCACGGCTCCAGTGGACACCATTCACATGGAGGCGACACCATCCCCATAGCATTAGACACAAGTGGTAGGTGTCCCGTGGAATTAAA
11	29427682	SCAFFOLD322209_10265	0.13	0.23	TCTGTCATTCCAGAAACTCTCGTCTCTGGCTGAGCAGGTCCCCAAGCACACGCTGTGCCTCGCCAGCTCCATGCATTGCTCATCGGTAGTTCTGTTGTCC[A/G]GAGGCCTTCCCCTCTGCTAACAGAAAGCCTGTTCTGCTCACATTCTGCTGACCTTCCTGGAGAGAGGTGACTGCTTTCCTTCACTCCAGTGGCATGTGTA
11	31532398	SCAFFOLD291653_1401	0.49	0.01	TCTTCAGAGCCCTTTCTCCCATCGCCTCCTCCTCTTGCCACTTACTCAAAAAGGACTCTTTTCAGACTTCTGTCTGAAAGTGTCCTTTCAAGAGGAGACT[A/C]GTGATGGTTCATATTTATACAGCTGCTTTTTTATAAGCAAGATGATAATGATGACTCTCAAGGTCCAATTAAATTCCCACCCCTACCCCAAGTATTCTCT

11	32616069	SCAFFOLD142015_4176	0.44	0.49	GACGTGGCGCAGGTCAGCCCAGGGCAGAAGCCATCACTTCCCATGAGCTGTGAGAGAGCAGCCTCCATTCCAGCATGAGGGACTCACTGGCACTTTGCCA[C/G]CTGCTGTCTGCCGGGACTGCATTGTGAAAGCCAGAGATGTTCACTGCTTATCCCCTAATCTTCATTTTCCTTTGACCTTTCACTTTACTTTTCTCCAATC
11	34760383	BES7_Contig224_786	0.41	0.39	AACTCTGTAAGTGCTGGTTACACCACATTTAATGTGATAATCTATTTAGCCATCATGTTCCTTCTTGAAGAGGAAAATTTTAAAGTAGTATTTTTTTTTT[A/T]AAGTGAGCTTGCAAATATGCCAGTTTAAAAGTGAATTTTATTAGTAAACTATATATGAAAGCTTCTCAAAGGTTCCCTAAAGCTATGACAGGATCATAAA
11	35797425	SCAFFOLD150288_2548	0.32	0.31	GTAAAGTCAAAGCCAGAGTAAAGGAGAAGAGATCTTCATCAATTTCAGATAAAATAGTTTACTACAGTTAGTTTAAAAAGGGATATGTGACCATGTGCAT[A/G]TATTTCTGGAATGCTAGAGTACTCTGAGACCCTATCAAGGAGCCACTGCAAGTGAGAGGCTCTGAAAATTGTTTCCTTAGCTTTCTGGTACCCCTCTCTG
11	38800904	SCAFFOLD11677_746	0.18	0.22	GTTCTGCTTTGAGTCAAAGGCCTGGAAGAAAAGATCTCATTTCTTTCCCGAGCATCACACGAAAATGCTGTCATGGCAAGGAGTAATCAGAGACACGGGC[A/G]GTTAAAACAGGTCGTGCAACCTTGAAGCTCACTGAAGTAACAGCTAAAAATAACCTCAGACACTAAGAAAATTCAACATAGAGAGAAATACCACCAGAAG
11	39528188	BES8_Contig394_1331	0.34	0.39	TCACTGCTCAAAAGGCAGTTGTACTGAGTAAAATTCCTTTTCCTACCTCTAAAGGGGGTGGAGGGTGATAAAATTATTTACTGAAGTTTATCAAGCTTTG[A/G]CCTCCTCATTTCATACAGCTAAATGCATGTCATAATTCAAATGACCTTGTGGTTAGTTTTTTATTTCACTTACATTAATCCATAGACAATAATCAATACA
11	41385170	SCAFFOLD100651_19325	0.41	0.49	AATCATAAGCTCTGGATTTGAATCCCAGATCTTATATTACTAAGGAAAATAGATGGGCAATGTAATCCTTCTTGATATCCAGATCTCTGTGTTTAATAAC[A/G]AAAATACAGATGCCATATCATTGTGAAGATGAAAAATGAGACAATATACATAAAACTCCCAGATGATAATGGAAATAAAGACCAAAGTACTATTTGAGTT
11	41385364	SCAFFOLD100651_19519	0.37	0.34	TATGATTTGTAATCTAGATTCTTTCATTTTTTGTTTTGTTCTGTTTTTGTTCTTGCCCAGTATAGGGTAGATGGTATATATTCAAATTCAGCAAAATAAA[A/G]TGACAATCCTATATATTAACCCTCTGAATATCTACTCTTGCTGTGATCAATGGTATTTTTAAATAAAATAGTAAGTTCAATCTGGGCTGAATATCAACCT
11	43134930	SCAFFOLD1401_3966	0.15	0.14	AGTATTCATTTACATCTGAGGTTCAAACTTAACACCAAATTTAGAATTTGATATATTCTGGGCTTAGTTGGACATTTGGATTGCCTTCTTTTATTCAGTC[A/G]TCTCAAGTTGTTTTGACCATTTTGAAAGATAAAAAGAATATGGAAGGTGTTTATTATGTGTTAGACTGTTATGGATCCCTTTCCTCCAACTTTGATCCTG
11	44726733	SCAFFOLD170036_23917	0.07	0.09	CAAGCTTAGGGAGTGAATGAGTCACTGCGATGTCCTCAGGAATCAGGCTTTGCAGGAGTCTGAAAGTGAGACCTGAACTGGCCTCTGGAAGTTCCAGAGC[A/G]TCTCTGTACTGTATCTCAGTCAGACAGGAGCTACCGGAGAGACGCCTGCCAAGGGAGGGAGGCTTCCTGGAGAAAGAAGTCCTGACTTGCTTTGAAGGAT
11	45173928	SCAFFOLD11407_4812	0.17	0.25	TACAGGGAAGAAAAAAAGTAAATGTAGTATGAAAGAGCACATAGTGGGGAAAAGAGCTTATAAAGAGGAGGGTAATCGAAGAATGCTTCATGAGGAAGGT[A/G]GGACTTGAAGTGAACTTTGAAAAATAGATGGAATTAAGATAGCAGAAAGGACAGCAAGCATTTGAGGCAAAAGTGAAAGTGTTAGTCACTCAATCATGTC
11	46636162	SCAFFOLD155239_14305	0.27	0.30	CAGCACCAACTTTTCAGCAGAGCCTAGCACATGGGAGGTGCTCAGTGACTCTTCTTTGGACAATGGATAGATGAATGGATAGAAGGATGGAAGCATGCAC[A/G]CATAGATGGATACATGCAAGGATGGATGGATGGAGGAATGAATGGATGGAGGGATGAATGGATGGATGATGGATGGAGGGATGAATGGATGGAGGGATGA

11	46636484	SCAFFOLD155239_14627	0.39	0.35	CCAAGAAACAATTCACAAACACGCTCTCCAAGGGAGTGGGTTTAGACCCGAAGGAGCCAGGTAGGCTTGGAGACTTGGCAGTGGAATTCTGGGCAGAAAG[A/G]GCTGCAGGTCCTAAGACTAGGGGCTGGAGCAGCTCTTGGAACTATAGGAGCCTGAGTCTCTGAGTTTGATGATCAGGGGCAAGGTCAGAAACGTAGGTGA
11	46646925	SCAFFOLD155239_24191	0.40	0.45	GGAGTTTCACTCCAGAGTGAGGCCACTGTAAAAACTGCCGCGTGTTAACCTGTGATGCCATGTGTGCACGTCTGGCTCTGTCCCCCGTCTATGTAGACAG[A/C]GTGGACACACAGGAGTGCGCTGGTCCACAGCTCGCCCACATTGGCTGGCTGAGTGCCCGACTCAGGGCAAGAGGGAGCCTGTTCCACCAGAGCATGTCCT
11	49194342	SCAFFOLD87273_8202	0.27	0.30	CTTACTGAGCGCCTACTAGGTGTCCAGTGTTGCACGGATGCTGAAGAGACAGCAATTAACAAAAAGGGCTGTACTTGAACTGGTGGCAGCTCAGACCCAC[A/G]GTGCTTTAGGATAAAGGACAGATGACCCTGTCTTCATGCTACTCAAGAAGGGCCAACCTGCAGGGGTTAGAAGTGCAAATAGATGAACATGAGAGCCAAA
11	51228013	BES1_Contig588_854	0.49	0.50	GTCTCATTTATCTGCACTAAAAATTCTTTTAAGTAAACATCCCTCAGGTAACAGGAACACAAGTCTGTATCAAATGTGGAGAACTTTTCTAGGGTAGTTA[A/G]TCTAAGCCATGGAGCACTAGAATTTTAGATTCTTCCTAATAATTTAAGCCTCTACTGAAGGCAATTTTTTATGATATCAACACATTCTAGGAATTAAGAT
11	51228123	BES1_Contig588_744	0.45	0.42	GGTAAAGCCTATTGCTAGTGAAAAACATTGAGGGTGTGGGTGTGTATATAAACTTGATATATTCAAATCTCAGAAGGCGAAGGTATGAAGAAAGACTCAA[A/G]TGGGGAAACATCTTAATTCCTAGAATGTGTTGATATCATAAAAAATTGCCTTCAGTAGAGGCTTAAATTATTAGGAAGAATCTAAAATTCTAGTGCTCCA
11	51624643	SCAFFOLD11005_14622	0.19	0.15	TAACATCACAGAATGAGTCTCTGGGATATAAGAAGAAATGTCAGTATTGTTACAATTTTATTAATATGTTTAGTGACCAAAAGTTTTAACTAATATTTTA[A/C]TACATGGATATGTTCTTGCAGTATTGAAAAATATTTTTTAGGAAAGGATGAAATGGTATCATCAGGGGAGAACCAGAAATGCTTAAAGTAGTTAGTACAT
11	52234314	SCAFFOLD322419_6669	0.34	0.23	TTTATTCTGCTTAAGGGGAGCCTAAGGTGCCTCTAGCCTATGATAATAGCCTCAGGGACTGCTTCACTGCTTTCTGTTGCAATACAAGCATTATTATTCG[A/C]TTATCTCACATTTAAGTGTTAGTCGCTCAGTTGTGTCCGACTCTTTGCAACTCCATGGACTGTAGCCCACCAGGCTCCTTTGGTCAGGGAATTCTCCAGG
11	52236190	SCAFFOLD322419_4793	0.23	0.31	TATTTCTAAACTGGTTTACAGAACCTTTCTTTCACAAGCATTTTATAGTTGACTCAGGGTCTCTCTGCTTAAATGTATGTCCGACCTATTAGTATAGACA[A/G]TCTCAGGACGGGCCCCACCTGGCAGGAAGGCTAAAGGAAAATTTTATGCAGGCAGAAAAGAAGAGTAAAAGTGTTCCTAAAAATCAGATCCTATTAAAAG
11	53414022	SCAFFOLD185915_444	0.50	0.42	GCTTGCCATCTTTATTCTGGTCATCTAGAACGGTTCTTTCCTTCCTGTAGTAAATATTGCTTATTGATTACTCAGAAGTTATGTCTACTTGTGCCCTCTG[A/G]CTTTGCTCTACCTAAAAGGCACTAATATTTGTGCTAATAACACAAGCTCTTTTGTTCTACATATCCCAGTGACAAGAAGGGCAGTAAACCAAAGAACAAA
11	54363220	SCAFFOLD116204_366	0.10	0.06	ATCTTATTATCAAGTGACATATAACATTCATATAAGATCACTAAACTTGCTTGTGGTAGAATCTGGATGTTCTCAACATCTTGGAAAATATTCACTAAGT[A/G]CATTTTTATTTTTCTCCACTGTCACTCCTACCAGCATATCCAGAATAAATGGAATAATAAAATTTAGTGTAGTTTATGTATCAAATGCTGCTGCTGCTGC
11	56051149	SCAFFOLD250122_6450	0.20	0.30	CTAAATTGAAGGATATTTGTAATAAAAAGATATAAGCCTCCAACTCCCATTTTTGCCAAACATTAATAAAAATCAAATTAGAAGCAAGTGCCTTGAAAGG[A/C]CACTGGCAAAAGATTTTAGTATTGTAGCAATGTGAATCTGATAAGAAAAGGAAATGCTCTTCCTTCTTTTTTTATTTTAGGAATAATATAAATACAACTT

11	56425473	SCAFFOLD102006_4351	0.08	0.12	TTCTTTATACCCTAGGATATCATCTAATGATACAAACCTCCTGGCCCATTTTTAGACCCTCCCTGCTTTTCCATGTCTCCTTTAATTTGTGGGGATCCTT[A/G]ACCAGCCAAAAAATAACACTGTTTTAAAAATGACAGCATGAATAGGAGACATATTTACATGCACTCAGAAAACTTCACATGCACACAACTATTTATATAG
11	56425699	SCAFFOLD102006_4125	0.50	0.37	GATATTCTTGAACCTCTGTATAAGGGACACGTTAAACTGATAGACTCTTAGAAGGGAGACATTTTATAGGCCAAATACATTGCTAGTAGCATCATGGAAC[A/C]AAGACTAGACCAAAGTGATTCTCTCGAGTCATTAAAAAAATCCTCCCCTGGGAGACATAAGGGTAGAACTGAGGGTTGACCTTAGGTCGCCAGTCTCTAG
11	56517942	BES8_Contig333_1177	0.39	0.35	TTGCAGGTATCTTGCTAACTCATTTCCCACCCCACAAATGGACCTGGATGTGAGAATTTCCAGGTGCTTGCTTTCTTTAGCTATTTGTGCTATTAAAGTT[A/G]GTTTAAAAGTTAACTGTAAGTGCGATGCCTTTTTTGAGAATGCTGCAGCTGTTTGAGCCAGTTATAAAAGATCTGTTAAGCAGTCTCAAGGGATATATGG
11	57775635	SCAFFOLD206529_9408	0.18	0.29	ACTTTGTAACTTACACTTATTTGTGTAATTCTTAAGGAAATACAGAATTATTATTAAGTTAACTACATTTTTGAGAACTAGATTTACTGATTCAGCATTA[A/C]TGCAAATATCTTACAACCCTATGAATCATTACTTGCCACTTGCTACAAATTTTACATCCACACTCAACATTCTACAATGAAAAAATTTCCAGTTTTCAAC
11	59067281	SCAFFOLD1537_522	0.39	0.47	AATCACTTGAAAAACTAGAAAAGATATAAAAAGTAAATGAGAATTGTGGAGCTGAGTCACACTGAGAATAGGATTATGGATAGACGAATCATAATTCCTG[A/C]TACTGCAGTATCTGAAAATGGCCTATTTTCTTTCCATCCCATGTTTAATTGAGTTACTCTATGAGGTTCCTAAATACCTGTCAACAAATTGGTCTCCCAT
11	60025313	SCAFFOLD102021_8291	0.13	0.06	AAAAATAAAAAATAAAACTGTCAGTGAGAGGGATCCAAAAAAATCAATTTTCCAGTCTTTCACTGGTTGTAAACCCTTGTGCATAGTCCCAGACTCCTTC[A/G]CTGGTTGCCTTGGGCCTTGTTTAAAACACAAGGATGTGCTTTTACTGTATTAACCAAGTCACTGTGTTCCAAGGGCAATCCATCTGGGCCCTATGGTGGT
11	64050042	BES11_Contig422_2192	0.19	0.09	CTTTTCTTTTGAAAAATCCTCTAAAAACACAAACACAGTCTCTTGTGTCTTATGACTTTCAGACTTTCTTCTCCTTCTCCTTTGTAAGTCATCCTAGCAA[A/G]ACTAGTTAACAAAATACCTGAATTTTAAAAGGCATTTTAACTACATATTTGTAAAAATTGTTTCCATAGAGAATGAGGCAAGAGTTTATTTTCTACAATC
11	64393474	SCAFFOLD250698_10358	0.45	0.47	ATAATTTGAAAATAATTACGTACTCTTAAATGAGACGCCACTAAAGAACAGCAGCACAACATTAAAGTAGCATGTGACACTCTCTAATTTTACCTGCTGT[A/C]ATTTTAATTTGGGGTTCAGAGTCAGGGTGGCAATGCCTCTTCAAAACATTCTGACCTTGGTAAAAAAAAAAAAAATCCAGGCAGAATATCTAAGAGAAAA
11	65710009	SCAFFOLD140292_18342	0.03	0.01	CTGAGCTGCCAAAATGACTCAGGTCAGAATGAACCTCCTTCAACCGCTAAATTGTTTAAATGTTGTTGGTTTGGGGGAGCAGAGTTGTGTTCATGGCAGA[A/G]CAAAAGGTGGTGGACTTATTTTCCAAGCTCAAGTGGCCTCATCCAGAACAGAGGGCATTAGACCCAGTGATCACGGCAGGGATCTGTGCACTTTCACGGT
11	67617120	SCAFFOLD30888_4001	0.36	0.26	GGGGAGATTTCTTTCTGGTGAAAGTTTTCTGACTGTTGGCAGTGTGTGGACAATTAGCAATATGTATATAAAACTAAATAAGTGAAAATGAACAGATATC[A/T]GGGACGCCAGGTGGCCTGGGCAGGAATTTCCAATTATGGGAAAGTAATAAGGCTGGTAAATCCTATCTTCCTCAAAATAACTATGCTGCTTAGTTGCGTC
11	67801515	SCAFFOLD265094_43968	0.45	0.20	AGGGGAGAAGAATATTATTTAGCCTGATAGCTAGATAGTAGAATATGTTAAGTATCATATACATTGTGCAAATAATAGGTCCTTAAAAGCACAGAGGACA[A/G]AGATATGGAGATCTTAGCAAAGGCTCTGAGAGACCATGGGACTTTAGATTTGAAGGAAAGTTGAGAGATATCAATAGGGAAAGAACACAGCAGATAGGAG

11	68274305	SCAFFOLD70144_44104	0.39	0.48	CGTTGCAAAACAGCTCTCTACTTGCTGCTCTGGGCTTCAAAGGGATATTATGTTTTAATGCACTGCTGTATAATTCATCAGCCCTCACCCCTCCCAGTC[A/G]AAATTACTGATGGAAATAAGCAAACACCCCAAATTTGCTATTTCCCTTTCCCAATGCCAACAGTGAGGAAATGTTCCCATGTAAATCAAAGCTGGGTGTG
11	70562037	SCAFFOLD296111_2548	0.10	0.17	GTCGTGGATCGTCTCTCTAGAGTCCAACAAGGCACAGAGCAACACAAGATGCCGGAAGGCTACTGGACTGAGGACCACCGCCACTGAAGAGGGATGTGGC[A/G]GCCTCCTGCACTGTGGGAAGACAGGAGGTACGTCTGTCCTACAGGGACATCTGTCCAGGTTCAGCACATCGAGGTGTTGGGGGGATCACGCGACCTTAGA
11	70865905	SCAFFOLD21235_6639	0.14	0.07	CCTTAGATGTCAAGAAAGGCTTCTGGGGGAAGTACCTTCCAAGCCAGGATTAGAAGAGGAGAAAATAACCAGGCCTGGGAAAAGAGGAGAATGCATTCCA[C/G]ACGCATCGGACGGGGGGCAGCACCTTCAGAGGTCCAGAGGGAGAAAGAGCAGGTCAGGCAGAACGTGGCTGAAAATTGGAGGATGTGACGGGAGGAGTCA
11	71024436	SCAFFOLD265651_7554	0.11	0.14	GTCCGTGGGATTTTCCAGGCAAGAGTACTGGAGTGGGGTGCCATTGCCTTCTCCAACCCTAAGTATAAACTGGCAGAAAATCTGTACTGTGCTCCTTACG[A/G]AGCTGCTAACTGCCCACGGACACTCTATGGAAGATAATCAAAGTAATTTCATTATTATGGATGCTGGCACCCTCACAGAGTAACTAAATGAGGATGTTAA
11	73608651	SCAFFOLD155529_13403	0.33	0.20	TTTTTAATTTTTTTTAAAAAAAAACACTTTAACTTTACACAAGCCCTTTTGTGATAAGTACAGGTATCATGAGAGCCAGACCTGAGAATTGCTGTTGCCT[A/G]TAAGCTGAATGTAGATTTTCAATGTCTGCATGCAAATTCAACCCAAACTTTACTCAGCTTGCTAAAATTTGGGGTGCTTCGTGCTCCAGCCAGTGGTACT
11	73608904	SCAFFOLD155529_13150	0.33	0.22	CCAAACATTGGGAAGTCACTATTCCCTGTCCTCAAGATATACAGGTCATCACTGATGTTTAACAACAGCTGGCCAATAGTCGCATGCGATTCTGGTTAAT[A/G]TTGGAAGTTCGTCCGTTCTTGAACATTATCTCTAGCATATTCGACATGAGCCATCCACAGACAAGCCACTATGCAGTGACTCTCTACCTATTCCCCACTA
11	76264190	POMC_121F2-SNP1	0.18	0.21	CGCTGCGCCCTCACTGGCCCTTCTTGTGGGCGTTCTTGATGATGGCGTTTTTGAACAGCGTGACAAGGGGCGTTTGGCTCTTCTCGGAGGTCATGAACCC[A/G]CCGTAGCGCTTGTCCTTGGGCGGGCTGCCCCAGCGGAAGTGTTCCATCTTATAGGGCCCCGAGTCCTTCTTCTCGGCCGCCTCAGCCTCCGCCTCCGCCA
11	77932725	SCAFFOLD301078_9080	0.50	0.00	GCCGCAGGTGGCAGGGTGACCCGCTGGGTGTGGGATATGACATCTTCCAAGGGCACTGTATGAAGTCTGAAGTCTGATGGCCAAGCTAAACACACCACCC[A/G]CCTCTACCCATCACCCCCAGCTGACAAGTACAGACCTGGAGCTGAATACACCAAGGTTAATGTCGTTTCCCTAGGACTTCGGCCGGGCGTGTGCTAGGCC
11	78704512	SCAFFOLD80476_18947	0.18	0.27	TTATGTTAGATCTACTGAACTATCAAGTATAAATAAAATATAAACCTTTTTATGAGAAATGGACATTACCGGTTAATTTAGTAAGGAGGTCTGCAGTCAC[A/G]CTGACTTTCTCACAATACATTTCTTTCTGGGTTAAGTAGATACTTTTTATCAAAAAGTTGGTTTTTTTATTATAAGTTTATTTAATTGGAGGCTAATTAC
11	78803054	SCAFFOLD110052_16832	0.50	0.07	ATGTAGGGCCAAGGTCTTTTCCCTCTGATAAGCATTCCACACAGTTTCTTTTTTTCTTTTTCCACAAAAGCCACATTTATGTTGCATGTTCTACAATTCT[A/G]TCTTTTGATATCTTTAAGTGAAAGTGTGAATGCAAAGTCTCTGGAGAGTCAATCTGAGCCAAGAATTCTAGAATTTTAGTTTCCCTCATCTTTTCTTTTT
11	79221114	SCAFFOLD165099_24110	0.18	0.17	GAAAGCAAAACTCATCACTTTCCAGGTTAGAACAGCTGAGACCCATCAACTCGCAGAAAACAATTTGTTGAGAGTAGAAGCTGTCACCGGCTATTACCAC[A/G]TGGAACGTGAAGAGCCGCTGCAGACCTCGGCTCCCAGGCTCAGGCAGGACCGCTCCCATTTCACTTGACAGCTGTGGACATTGGAATAATACAAAACATA

11	79221464	SCAFFOLD165099_24460	0.49	0.03	AGAGCATACTGTCTGGATCTTCTAAGGCTTGTGCTGATGTATGGGGCGCAGAGATCAACTCATGGGCAGAGGAGCACTGCAGATAGTCTCACATGTACTC[A/G]GCTGTCTGAGACTGATTTTGCGTCTTGACTCAAGAGCATTTGTTCTCAGGCTGATATCTTTGCCTCATGAGTGGGGTTAGCTCACACCAGTATCCTTGGG
11	79221476	SCAFFOLD165099_24472	0.02	0.03	CTGCTGGCATTACCCAAGGATACTGGTGTGAGCTAACCCCACTCATGAGGCAAAGATATCAGCCTGAGAACAAATGCTCTTGAGTCAAGACGCAAAATCA[A/G]TCTCAGACAGCTGAGTACATGTGAGACTATCTGCAGTGCTCCTCTGCCCATGAGTTGATCTCTGCGCCCCATACATCAGCACAAGCCTTAGAAGATCCAG
11	79257273	SCAFFOLD47483_528	0.17	0.19	CTTTCACCCCAGTGGCCACCTTGCTGTTTTTCAACAACACCAGGGTTACTCCAGTTTCGTGATATCGTCACTTGTCTTCTGCCTGAAATGCTTTGCCCTC[A/G]GGTTTCTTTTGGTTCACTCTCTACCTCTGTATTCATATCTCTTCCCAAATGTTACCTCTGGGTCTTTCTCAGCTTCCTCAATTTTAAAACAATCTTTCAT
11	79443335	BES11_Contig369_1053	0.34	0.41	TTCACCCCTCTTGGCTAGAAAATCATGGTGTTGTGACCAGAAAATTGAAATGGAATGAGATCCAAGATGGCAGACACAGCCTTCTTTTCCATTAACCTTC[A/G]GGCTGGAGTGCTCTGCAGAAGACAGGGTGACCAAGATGAATTACCTCTTGGATTTCAGTGCATTGTTTCACTAATGTAAGAAGGCTATCTGAGAAGTTTG
11	79480432	SCAFFOLD70339_4331	0.14	0.09	GATTGGCAGGCATGTACAAGCTGAGAAGTAGCATTTCCTCTTTCCCAGACTTGTTCTGCCCTTTATTTGAAGGCGGCAGCAGTTCAAACTAAAATTCTTG[A/C]AAGTAATAGATCTTAATGTGGATTCAATTCCATATCGGTGGCAGATCCGTACATTATAGATAACAGGTAAGTTATAAAGGACTTCTTGGCTGTCTACAGT
11	80473823	BES10_Contig646_1317	0.20	0.29	GAAAAGTCTATCCAGGAGTCATCCAGTGTCAAAAACTGTGAAGGGATGTATGGCTTTGAGAATGAAGTGTTCCTGAGTCTTCCATGTATCCTGAACGCTC[A/G]GGAGTTAACCAGTGTTATCAACCAGAAGCTGAAGGATGAAGAGGTTGCCTGACTCAGGAAAAGTGCAGATACCCTCTCGGGCATCCAGAAGGACCTAAAG
11	81776062	SCAFFOLD160408_1267	0.26	0.47	CCCAAGGCAAAGTAAAATGTCCGGTAGTGGTTACCCCATTCCTCAACAAAAAACAGCTTTCTAAAATGTGATGCATATAAAATATTACATTTCTTAAACT[A/G]TGTTAGCTTTGGGCGGGTTGCACGTTTTCTATTGAAGTTAATACCACATATTGAGGTGTAGGAACTTAAGACAGGCATCTAACACAGTGGGCCATTTAGG
11	85523417	SCAFFOLD115412_13015	0.45	0.46	GGCATACCTTGACTCTTGGTACAGGCACATAATAGGATCGCAGTACATGCCATGTGTAGTAATGTGATACATGGCCCCAGAACAGTGGAGATACTTAGTA[C/G]TCCTGATGCCTGTAGGCAAAGAAAAGCACTTGATTCTCCATCAGTAAATAGAACAAATATTTTCAAACAAAATACTAGTGACAAGAGAACTATTTCTATG
11	86608060	BES11_Contig379_951	0.26	0.19	TAATTTGTTTAATGCCTTCCTTGTGTTGAAGGAAAAAATAAAACCTAACAAATTTCAGAGGAAAGAGAATTCTTTAAAATGCCTACACTTTGGAGGTATT[A/C]GTTTATTTATTCATTAATTCAGCAATTTGTTTTGTGCCTGTATTGTCACTGTGCAAAGCACTGCAGACACAGGTAGACAAGACAGGCTTGATTCCTGTGC
11	87730076	SCAFFOLD120028_3470	0.41	0.48	GCACACGCTCATTTCACCAGAGAAAGCAAACATCTGAAAGATTTTTAATGACTTTATCCTTTGAATCTCTGGCCACAGAAAGGAAATGGAAAATAAATAC[A/G]TCCTGCTTATTACTTCAGTGTATGGTTAACTCTGAACTGCATTTAATTTGATTGTTAGATGTACCTCTATGTCACTCTGAGTCAGCAAGCACACTTCCAA
11	89970612	SCAFFOLD168801_508	0.16	0.10	TTTGGAAGCAGGGCTGTCTTAATGATATTAAGCCTTCCAATCCATAAACATGGGATGTCTTTCCACTTAATTATGCCTTTAATTTGCTTCAAAATATTTA[C/G]TCATTTCCAGAGTTTAAGTCTTGTATTTCTTTGGTGACATTTATTCCTAAGCATTCTATTCCTGATGCTACTGTAAAAGACATGATTTTCTTAATTTAAC

11	90032978	SCAFFOLD265623_27416	0.45	0.43	CTCCAAATACAGCCACATCCTGAGGTCCAGGAGTTAGGAGGTCAGTGTGTGAATTTGTGGGCAGCAGTTCAGTCCGTGACAGCAGCTGGCCCTCTCTGCC[A/G]GCCTTTCTGGACACGTGGACGCACGCCACCAACAGAACAGCCTCGGGTCTCAGCGGAAGGCGTGAAGAGCGACGGTGGGTGGGTGCGGTGAGACCCTCCA
11	90508793	SCAFFOLD125360_791	0.15	0.12	GGAAAGATCCCCTGGAGGAGGAAATGGCATCCCACTCCTATACTCTTGCCTGGAGAATCCCATGGAAACAGGAGCTTAGAGGGCTACTGTCCGTGGGATC[A/G]CAAAGAATCAGGCACCACTTAACGACTATACAAAAACAACATGTCATATGAAACTCTTGTTAAATAAATCTGTATGCTTTTCTCCTGTTGATCTGTCCTA
11	95021154	SCAFFOLD110374_786	0.48	0.46	ACGCTCACATATACAGAGGCAGACCCTTTCTTCCCACTCTGTTTCACCGTAAATATTACTTTGAGTATCTTGTTCTAAAAACAATTGATTTTTGAGAGGA[A/G]ATAACTGAGAGGAAAAGAGAAGGAAAGGTATAATACTGTAAAAGTTTACTAGACAAATTTTATAAAATCAAATATTGCCCGTTTTTTTTTTTTTTGATAC
11	95382206	BES3_Contig455_1132	0.23	0.28	TAAGGAAAGGAAATTGATCCATTTGAGGATCGCCAAGGTAGACTGTGTCCTACTAAGAGTTCTCCTGCTGCAAAAGCAACTCTGTGTTGCTTCAGACCCA[A/G]CAGAGATAGGAAGACACAGAAATCAAGAGGACAGCCCCTCACAATCATTATCTTGTTGCCAAACACTTCCCTTTGCGTACTTCTCTCCAGTAAGTCAATT
11	95382283	BES3_Contig455_1055	0.22	0.29	AACTCTGTGTTGCTTCAGACCCAGCAGAGATAGGAAGACACAGAAATCAAGAGGACAGCCCCTCACAATCATTATCTTGTTGCCAAACACTTCCCTTTGC[A/G]TACTTCTCTCCAGTAAGTCAATTGGCACATATAGAATGTTTCTGGCTTTTTCTTTGTCTGTGACAATTTTTTTTTTTTTTTGGTTCAACTGATGGTGTAT
11	96403685	SCAFFOLD316249_3210	0.46	0.38	CCTGAGCAACATAAGCCAGGTGTGTGTAATACCTGGACATGAGTTAAGGAGGGTGTGCGCATCAGCCACGTGTGAACGACCTCATGTGTGAGGTGGATAC[A/G]GAACATGTAGTATGGATGTCCTGAGTCTGTGTGAATGACATTTGTGCTCTTTGGACTATGTGCAGCTGCTCCAGCACAGAGGTGTGTGGGAGACTCATAC
11	100288504	SCAFFOLD10013_26872	0.15	0.27	GTCTTTGCCTCTGCTGTAGACAAGAGTAAGTCATTCCCACTATAGTAAGAACAGGCAGTATTGGCAGCGGTGGGGAGAGAGAGAGGCATGTGGAGGGAAG[A/G]GAATAGCAGGAAAGGCAGGGAACTGTGAGGTGTATGCTAGTCTATGGGGATCTGGGACAACTTCCACAGGAAGTCACTTAAAACTAAGACCTGGGAGCTG
11	100609317	SCAFFOLD145079_36012	0.49	0.48	GGTTTAAACTCATTATGTAAACAAGATTACTAGAGTAGAAAACAGTTAAGAAAATAACCATACTTCAGGTATCTTAGAAACAGCCACTTACATACAAATA[A/G]TAGAAACCAACAATTTAATAATTGTTACATCTATTATGTATATTCAGTTACTATGTGTATTCAGGTTTTTTGAATTACACCCTATACTCTTTTCAAAGAT
11	100609582	SCAFFOLD145079_36277	0.48	0.48	AACCTTTGCAAAGTAACCTGTAGGGGTGTGTCCATTTTTAAGAAAAAGTGACTTAAGAGTTCTGAACATTGAAATCTCTTTCCTTACAAATAACACTGGA[A/C]TTGTCTTCCTCTGGAAAAGAATTTTTGCCCCAAGATGAAACAAAAATATACTTTATTTAGCAATCTTCTCAAAATGATGTAAGAAAAGGATTCTAGTTTT
11	100609735	SCAFFOLD145079_36430	0.47	0.49	CTTTTTCTTAAAAATGGACACACCCCTACAGGTTACTTTGCAAAGGTTTGGGGTTGTCGGCTGTGAGGGATAGAAACCTCAGACCAGGAACAGCTCCTCC[A/G]AAGCCTGACTGTGGCCGTGACCGAGAGGTGGCAAAGTCAGTGCAGCTGATTAGAATCAACCAGCTAATAAGACACGACTGGTTGAGAATAGTTCCTGCAG
11	100657545	SCAFFOLD105425_19654	0.47	0.50	TTTTTTGAGTACTCTGGAGGTAAAGAGAATACAGAATTCAGTTAGTACAGGTTACTGAAAGTGCTCTGGGCTCTGAATTACATTTCTTTATATTTGGTCT[A/C]ATTAGTCATTCCGTACTGTAGGCAGCCAAATATATTTACCTTCCTATTTAACTTCTTTCAAGACTAGACTAGATACAAAGTAGAACTTAACTGAAAATAT

11	101206485	SCAFFOLD133207_4290	0.24	0.24	TGGGCTCTTCTGTGGACTAATCTGCATGCCCTCTGGCAGATTCTAGATTCCAGGAGCTGCTGGTCATGGTCCTGCCTCGAGGAGGACCCCTAGGCTTGTG[A/G]AGGGATGTTGAAGGCTGACTTGCCCCTGGAGACTCATTAGGGGTCAAAGTGTCGTTGGAAGTTGGGACAGTTGGTGGGATCTGGGAAAAGAAAACACCTG
11	101206654	SCAFFOLD133207_4459	0.24	0.24	CATGCTATTAACCAGTAGGCCACACGGTCTTCCCAGAGTTAGGTGCCTGAGCCCTGACTCTTGCGCTTCCTTTCTGGAAGGCTCTAGGCAAACAGGGATC[A/G]TATCTGAGCTGCACCCCGCTCTGGCGCTCTGGCCGTCACTAGGGCCACGTCTCTCAAGGCTGCCCCTTCAGGTGTTTTCTTTTCCCAGATCCCACCAACT
11	101475357	BES11_Contig391_1117	0.29	0.30	ATTCAATAAGATATGTGTCTTTTGAGTGTGTAACTGTGCATTCAAGATGCAAATGCAGTAACAATTTTGCCCCTGGTTTTAATTGTTTTTCCTCAAAGAC[A/G]CATGTGATTTTTCTTATCTGATTTAGGCATTTCTCAAGGGGAGGGGGTAGGAAAGTATTAGCTACTGCCACATCAAAAAAGCTAAATATAATTGGAAGTT
11	101649690	SCAFFOLD290146_30534	0.09	0.19	TGGTGCTGAGCCCCAAGGGGAGGCTCCTCCCAACCCTGGGCATAGCCCCACAGGAACTGCATGGCGGCCATGGCCTCCTGGTGCTTGTGCTGAGACAGCA[A/G]GAAGCGAGGGGTCTCAGGCATGAAACACATGAGCAGCAGCATGAAGGAGGGGGCCACACAGCCCAGCACGGCCAGCCAGCGCCACTCGAGCACCCAGCCT
11	108690051	SCAFFOLD288881_1674	0.11	0.17	GCAGGAGGTCGAGGGGGTGGACGTGGGGCTCGTGGGCTGGTGGGGCCCCTGTGGTCTGCAGCTGGTCGCTGCGCTCAGTTCTCACCCCCTGAGCCGCTTG[A/G]CTGACTTCTCACTTCCCTAGGTTGCCCCCGCCCCCTCCTCCCAGAAATAGCACTGGAGTGGGTGGGGGTGGGGCGGGTCGGCAGGTGGGCGGGCAGCTTG
12	1827430	SCAFFOLD125291_16671	0.46	0.43	TGAAGAAAAAGAAAACACATTGAAAAATATTTCTCTTTTTCTGACTATGTTTTATATGCCCCAGCTCAGTATATATTAATTTTGAAAGCCAGGAAAAAAA[A/G]TGGCAGAGATTTTAAATAAATAAATTTGGCTGAAACATTACAGAGCAAAGCAAAAGCATAATGTACCTGGGATAACAGTTATAGCCATTCCTCAAATGAG
12	1842680	SCAFFOLD140123_40267	0.37	0.40	ACAGATGCTTTATATATGTTACTTAGATCATTCAAACATGTAGATAATGTATGTGAATTTATATTTACCATGAGAGCCATTAAAATGTCTACAGAGACAG[A/T]CGAGTCTTGTAGAATGCCTTATTCTGCCGTTCTCCTTTCAAATGAATCTAAAGAACTTTTGCAGGCTGCCACTTTGACTGTTCTGCTCACTCCCCAATAC
12	5564734	BES9_Contig515_961	0.14	0.33	GAAGTGAACTAAAAACAAGTAAGCACCAAAGGAAATAGATAAAAACAAATAACTTATCTTTTAATTACAAAAAATCAATTTAGCAACCACTGGGAAGGGG[A/G]AAAAAAAGGGATACATGTCTTATTGGTAGATTTTAGACTACTTAGCAAGGTTATTTATCATCAGTGTTAGCAGAATTAGTGCCCTTTGGTAGATATCTTG
12	8844711	SCAFFOLD30413_12165	0.05	0.07	ATAGAACATTCCCCCCATCTAGCAGATGGCTAATGGGGAAGCCGGTATTTTCTGTGGAAACCAAAGTTTCTTTTAACATTAGACGTTTTCAGCAGAGAGA[A/G]CAAATAATCATTTGTTTTTCTATTCTATCACATCCATAAAGAGAATGTATTTCAAACTATTAATTTTAAAAGTGCATAAGAAAGTGAGGTTTTCCTTAGG
12	9475889	BES11_Contig381_736	0.16	0.32	CCAAAGTGTGTGCGCCGAACAGCTGACCCGGTCTTGTTCCAAATCCAAGGGCCTGTAAGACAAATGGGATCGGAATGGAAGCCAAATAAAAATGGAAAAA[A/G]TGCTACTCCGGTTACATAAAGAAGTCGGTGTTTAATTAGACAGGGGCTATGAATCGCAGCCTTCAAAATAAAATTGAGGGGTGAAAAAAAACAATAAAAC
12	9476130	BES11_Contig381_495	0.43	0.12	TAAGTGGTTTCCCCTCTGTATTACTTATTTATTAAAGCCAGCGAGGCATAACTAAGGGAAGAGAAAGGAACCAAGGAAATAGAGAATACAATGGCCCCTC[A/G]ATTTTAATGGGAAAGATGGCTGGAAGTGATTCCTAAATTATTAAATAAATCGATTTGATTCTTGCAGCCAGCCTCCGAGGTGTGTTTGTCAGTTTCATTA

12	10343016	SCAFFOLD322140_14795	0.40	0.18	AAGTTAGTCATTTGAAGAAAATGCTGTGGGTTCTGGAGTGTACTGGTTGGAGACAGATTCAGTGTACTTCTGAAAAGCTCGTTGACTCTGCCTGCGTCAG[A/G]CACTGCCTACAGTGCCTCGTGCTGCCATGACGACCCACACTTTCAGATGTATTTAAGCACAGTGGTGGCCCACAGTGAGTCCGTGAAGAAAGCGGCCACT
12	10435763	SCAFFOLD215881_4945	0.24	0.30	ACTGCTTGAATTGACCTTACCTCTTGAGGGGGCACGTTAAGTTATCAATTATTTGCAGATGCCACGGTCATAGAAGCTTCTGGCCCAGAGACTCAAATGT[A/T]GTTTGCTAAAACAGCTAAAATAATGTGGGGACTCAGGAAAAGACAAGTCTATCATTTGACATAGTGAAAAAGTAAAAATGTGCCTTTTCTGTCTGTCAGC
12	10435798	SCAFFOLD215881_4910	0.25	0.30	AACATGATTGGAGAAGGTGACAGACACATGCAGGTACTGCTTGAATTGACCTTACCTCTTGAGGGGGCACGTTAAGTTATCAATTATTTGCAGATGCCAC[A/G]GTCATAGAAGCTTCTGGCCCAGAGACTCAAATGTAGTTTGCTAAAACAGCTAAAATAATGTGGGGACTCAGGAAAAGACAAGTCTATCATTTGACATAGT
12	10435828	SCAFFOLD215881_4880	0.25	0.30	CACCTGCAGCGGTCACTCATGGAATGGAGAAACATGATTGGAGAAGGTGACAGACACATGCAGGTACTGCTTGAATTGACCTTACCTCTTGAGGGGGCAC[A/G]TTAAGTTATCAATTATTTGCAGATGCCACGGTCATAGAAGCTTCTGGCCCAGAGACTCAAATGTAGTTTGCTAAAACAGCTAAAATAATGTGGGGACTCA
12	10436000	SCAFFOLD215881_4708	0.28	0.37	TTCAATCGGTTGAACGCCGTGATTCTTTTTCAGGTCAGACTCTCTCTCACCATATACCACATGGCTGTCTACTATCTCGGACACAGACGCACTGCTGGCC[A/G]AGTGGGGCAGAGGTGGCGTTGTCACTGTTGATATGGGAGGCCGTGTCCGGCTTTGGGAAACCGGCCTTGAGCACCTGCAGCGGTCACTCATGGAATGGAG
12	10436129	SCAFFOLD215881_4579	0.25	0.30	GTAGACAGCCATGTGGTATATGGTGAGAGAGAGTCTGACCTGAAAAAGAATCACGGCGTTCAACCGATTGAAGCCCAGGGAATGATGCTAGTTCAGCTCA[A/G]CTTACGTTCAACTTGCTGTAAAGAATTCTCATAGTTAAGCAACAAGAACCTGCTGTACAGCATAAGGAACTACACTCAATGTCTTATAATAACCTATAAT
12	10651936	SCAFFOLD130755_5479	0.34	0.42	GCTTGGACCTTCAGGGTATCTTTAAGTATTTCATTCGTTTCTGTCCATATCTACTGGACTTCGAGCTTCTTGAACCAACAAGTGGATTTTATTCAACTCC[A/G]TCTTCCTGGTGCCTAGCACAGTCTAGAAGCCATTAGGCTTGTTGAGGGAAGGAAGAGAAGGAAAGAGGGAGATAGAAACGGAGGGGAAAGGAAAGAATTA
12	12551009	BES10_Contig531_767	0.25	0.28	CAACAATATAGTTAGTAATCTTTTAAGCTGTTAACACACACGCATTAAACAACGACCCTGTTCTACCACAGCAAATCATACAGTGAGTACCCCTTGTTAC[A/G]GCTAAATGAAAACATTCTGACTACTGAGACTGTATAAGTTGAAAAATGTCAAAATGATAACACTAGTTGATATTGACAAATGCAGTTTTGATTTTCAATG
12	12551341	BES10_Contig531_1099	0.28	0.32	GTTTAAGCCAACGTGGGGGATATGTTATTTCTAATATCCTTGTAACATCCCACAGAGTATATAATAATAAACATGAGAAACAGCATATCAAATTTCTTCT[A/C]ATTTCTATCTGTCTCCCAGTGAAATCAAAGGATTACAATCTGTACATAATCTACTTCCAGAGTTCTTATTGCAATAGAGTAAGACTATTACCCATTTAAA
12	14405209	SCAFFOLD191599_16534	0.36	0.29	GGCAGCCACAGTGCTCCTGGCACTGCTGTCTTCATTTTTGAGCCTGCACTGTGTCCCCAGTTGTATGCTTGTTTGCACCCAGGGTCTAGCCATTCGCACA[A/G]TAAACCGTTCTTTGGAATTTTACAAAGAAATCATTCATGGCACCCAAACTGAAAACCGAAGCCCATCCCGTCATTTTCCATTTATGAATCCTGTTTAACT
12	14405373	SCAFFOLD191599_16698	0.39	0.39	CATCCCGTCATTTTCCATTTATGAATCCTGTTTAACTAGAAAGTCGTTTATGAACAGTGTCCCATGTTTTCTATGCAGTCTGAAGTAGCAGATGGCAGGC[A/G]TGCATATTACCTGGTTGTCTGAAGCCTACTTCAGTAACTCGCTGACTCATTTACACAGGTCCCTCGTTGGAGCCAGCTACAGAAGGCGTGGGAAGCAGGC

12	14648490	SCAFFOLD376915_542	0.17	0.14	GCACACAGGGACCCTATCCAGGCCTGGAGGTTGTGCACAGAGTGGAAATTGTCTCTAGTTTGCCAGGTGGAATTCCTAGCATAAGTGCTGCGTGGTTGAA[C/G]GCTAGCGTGAGACCAGCTTAGTAATAACGGGTGACTCTGGAGAGAGAGGCAGGGCTAGGTCATTCCTTTATTCACTCACTCAGGAAATATCCATTGTGTT
12	18071553	SCAFFOLD121345_5143	0.22	0.14	AAATGGTTGGATGGTAGAATCCACACGACTCGAGACCAATGCTAGAGGCAATGACTTGGGTTTTATGGGATGCTGGTATCATTATCTGAGTTAGGATTAT[A/G]GGGAAAAGAGCAGGTTTGGGAGAAGATAATCACTTGGGATTTTATGTATACATGATTTTCTTCCTGAGTGAACAGAGTACAGGAAATATACCTGAACCCT
12	21872295	SCAFFOLD140305_7297	0.22	0.24	TGGTTGGCCTCCTGCTGGAAGCTGACAGCTCATGCTAAGCCCCCCTCAAGAGGGCCCTCAGTGTCAGCTCTTCCCAGGCACGCAGGGTACGACCTGTTCT[A/G]AGCCCGCTGTCATACATAGCTGCTCGTCACCAGGTAGCAGCTTCTCCCTAAATCTTTTAGTCATCACACCCCTTCCTGCAGACCCGCTGCCTAAAGGAAG
12	25363434	SCAFFOLD140006_43910	0.20	0.23	AAATGACATGGTCTTACTGGACTAGCAAAGATTAATAATAAATAATAATAATATGCAGGGTTGGCAAGCTTAGGAGGAAACCAGTCATCTCATATGACTC[A/G]GTTCAAGATTAACCTGATGGAACTCTTCCGAAAAGTAGTTTGGTAATACGTAACTACAGCCTTAAATAATATGAACTCTGTGGTCAAGTAATTCTACTTT
12	26952728	SCAFFOLD100457_11884	0.12	0.28	ATAGTATACAACAGTCTTTTGTGCTGGATTTGATGTTAAGCTTTGGGGCCTCAGTCTTGCTATTACTGATATCAGCAGATTTTTCTTTTAACTGCTATCC[A/G]TGTGGCATTACATTGTGGATATTCCACAACTTTTATATTCATTTACCAGTGGGTTCCAGTTTGGAGCTTTATGAATAAATCTGATATTAATGTTTACATA
12	27020219	SCAFFOLD57013_2704	0.19	0.13	GAAATTCTAAGATAAAGAATCCAGTGTGAACAGTTTGTTTGGGAGGTACTACAAAGAAACACAGACAAATAGTGGGTAATAGGTGAGACTGCAAAGAAAA[A/C]GCGGCAGTAAGGGTGAGCTCCTCTGTGGAGCAACTGGGGCCCCACATACTTGGAAAACCTTGGAAGAACACAGCCTTCGGAGTTATCCCACCTGAGAGTC
12	27735665	SCAFFOLD309947_1540	0.48	0.45	CTTCCCACAGTCAGCATCCTCTCATTAAAGTCTTGATAACACCAAATTGCTTTTTAAATGCCTCGTACTCTATAAAGCTGATGAGCAGTGAAATAAATCT[A/G]TTCAGTCAACGGAGATTGGGTTTATTCTCAGGGGTGGCAGGAGGTGGGTACGTCAAGGAGAAGGGACTGAACTGAATCCTTTTCACAAGAAATTATTTCA
12	29556714	SCAFFOLD220223_3154	0.15	0.09	TAGGGAAACAAGGGGAATCTTATCGCCTATAGGAGAAGAGACAGCAAAGACCTCCGTCTCTCGCAGTAGACCCACCCAGTGGCCACACCTCCCGTGCAGC[A/G]GGCGCTCAGAAAGAACACCCCAATGACAATTAATCAGAGGGTGTCTCCCACGCCTCTGATGAACTTGTCTCCCTTTGCTTTCAAAGCTATTCCAAGACTA
12	35004860	SCAFFOLD151911_7094	0.23	0.41	TTGATGTATACCAATCCACAACGATCTTAAAGTGCTTTAAGACAGCGTTGATACCAGGTTTTGTGAGGATGCCTAAAGCTTTCTCCAAGTTTACATGTAC[A/G]CTCTCCACAAGGGGAAGTGACGAGCCAACCAGGATGGCATGGGCTGCATCGCACATGTCAGGGGGTGCACAGAGCTCACAGAGGTCGCCCTTCCAGACTA
12	38978670	SCAFFOLD160629_768	0.04	0.05	GGAAAATGAACAGAGGAAACCTTTACCAAAACCAGTGTCAAATTTGGTAAATGCCCTGCTGAAGATATGGAAGAGGAACAAGCTTTTAAAAGATTTAGAA[A/T]TACTGATGAGATGGTTGAATTATGTATTCTGCTTCAAAGCAAGAATGCTGGGGCAGTGATAGGAAAAGGAGGCAAGAATATTAAGGCTCTCCATACAGAC
12	42522026	SCAFFOLD317155_7805	0.14	0.07	CCACGTCTCTGTCATAGTCAATCATTACCTCCCAGAACACTCTCTGTCCCTTAATTTATTTTAGACCCTGCAGTTAGGAACTGCTCAGGCAGGATGGCTT[A/G]AACTACACTCCCTATTTACAGTTTTTATTTGACAACCACAAAAGCTGTTTTGTTGCAATTAAAGCAATATTAAACTGAAACACAGTACCTGATTTGTGTT

12	45365350	SCAFFOLD130137_7387	0.34	0.29	GACTGTGGGCTAAGTCACAAAATCTCTGTGGGCCATCTCATCACATCCTTTACATAATGATGATGATAGTAACTACTTTACAGTATTGGCACAAGCATCA[A/C]AGACTTATAAATACTTGCAAAGCATTTGTTCTTGTAAAACATTTGTAAAGCATTTTGAAGAATGCTTGTCACATAACTGTATGCGTGTGTAAAAATCATG
12	49174587	SCAFFOLD20370_15205	0.06	0.07	TCTGTCAGTGTGCTCTATCTCTGCCTTCTCCCTCAGAAAATGGGCTGTTTATTCAGTGCACTCTGTGCTTAAATGATTGTGCAAAGCTTCAGGGTATACC[A/G]TACTCCATGTGGGACCTTCCTTAGTTCTGAGAAAGTTTGCTTTTTCTGTATTGTCTGTTTTTCTTCCTGGTAGGCTACAGTCCATGGGGTCCCAAAGAGT
12	49365732	BES9_Contig262_245	0.09	0.06	ATTTTTCCCTTCATGCCCTGGCTTCTAGGAACCTCAGATTCTAATGTAATGTATATTTATGGCTATTATATCAACCTTTAGAGTTAGAAATGCTTTCTCC[A/G]TTTTCTTTGTTCTGCTTTTCTATTTCTGTTTCATCAGCCTCAGGTGATTATTATACATATAATTTTTCATAATCTGCTTCAAATGTTCTGAAAGAGGATC
12	49559660	SCAFFOLD179577_214	0.34	0.28	AGTGTTAGCACTACCCTTCCTGCCTGTGTTTATATAGCGCCTTCTAATATGACTGTCTGATGATGCTGCATGAGCTTTGTCTGAAAAGATGCCCAGTTAC[A/G]TGGGAAGATTCTTTTTTCCCCTACTCTCTTTCCTTGATGACTGTCGTTTGATGGATGCTGTGTGAGGGAGAATGCAGTGCTTGGTTGATGTGTGGGGGGC
12	50533572	SCAFFOLD100976_23622	0.45	0.48	CTACCAGGCTCCTCCATCCATGGGATTTTCCAGGCAAGAGTACTGGAGTGGGGTGCCATTGCCTTCTCCGACTAGTTTCATGGGAGACAGTTTTTCATGG[A/G]TGGGGGCTGGAATTGGTTTTGGGATAATTCTTTAAGGACCTTGTAACTGGGAGATATATGTATAATTATGACTGATTTTCACTGTTGCATGGCAGAAACC
12	51648410	SCAFFOLD241049_3342	0.15	0.21	ATTTATGGTACAGGGTTGGGCTTTTTCAACATTGTGAAGTAGATAAAGTTCTATTTGGGCTTTTCAGCCTCTCTCTTTTATTTCCTTTCTCAGCTGCCCA[A/G]CTAAGCCCACTATCAGAAAAGGATAAGATATAGACTCTGTTCTATCCTCGTTATCATTTAGAAAAAAAGAAGTGATGTCAATACTCCTTACACAGTAAAC
12	54576942	SCAFFOLD10665_6174	0.25	0.28	ACAGCCAGCACAAACATCTCCTTTCTTCTTTCTCCCACCATGTGTATACATAATGGAGTTTCTTATTAAATATACATGCAACCCTTCGTTGCATATAAAT[A/G]TACCTCATCAAAGAATAAGGGAGACCCTGCCACTCTGCTATTGATTGGTCATGAAGCCAGGGCAATATCTGAATGGTGTTCTTGGGCTTTCTCTTAAAAA
12	55402725	SCAFFOLD245021_7420	0.31	0.43	GCCCCGCCATTTTTTTCTGGTTATCGGACATTACAGGCTTCTGCTATGTTTTCTCAGTTGAGCTACTCTTTGATTTGTGAGCAAAGGTTTAAAAGGCTAA[A/G]AGGGGGGAAATTGGTTCAATATTTCAGGCACAATGAAACATTTCTTTTGTCACTTTATTTCCCTGCTGTATTAATTTGTGGTTACTCACCTTCTCCTCAG
12	55526591	BES10_Contig764_3023	0.04	0.04	CAGTATGGTTGGTTTCTGTTATAAGCTTGAGAGCTCTCTTTCTGGCCATCTTCTTACCTTGTCCTCACATGGTGGAGAGAGAGAGGCGGGAGAGAAGGTC[A/C]GGGGATTTGGGGAAGGGAACAAGCTCTCTGGTGTCTCTTTTTATAAGGACACTAATACCATTATGAGGGCCTCCCCTATGACCTCAACCAACCCTAATTA
12	55764125	SCAFFOLD116899_5317	0.49	0.42	ATGGGAGTTGTTGAGAGAGGTAAAAATCCAAGTCTAACGGACCACGGTGCTATGCTGAGAAGTTAAAAACAATACAAAAAGTACCATGACTAACACAGTC[A/G]GTTGAGTTAACAGTCTTGTGATGGAAAACAGTAGATCATCAATTTTATTACTGTAAGTTTCTAGTTAAATGAGCTTTTCTCAATAGCATGACTGAACAAT
12	55777787	SCAFFOLD115574_15191	0.49	0.43	GCAGATTCTTCACCACTTGCACTGCCTGGGAAGCCCAGTAGAGTCAGTATCACTCCTTTATCTGAGCCTGGTTAGATTTCTGCTCCGGTGTTTCAGCTCT[A/G]ATAAATTGATTATCTATATTTGCCTGCCTGTCTCTCCAGCTTGGGGATCAGTGGTCTATCCTTCAACTTCACTTCTCTGATAGTTCTAAGAAGAGTTTTT

12	55813428	SCAFFOLD105428_14408	0.50	0.44	GATGAGCTGAATTTTCCAAAATGGGTACTTCTTCATACCTATTCTTTCTTAGACACCATCAATATATCTTTACACTATTAAATTCATCCATTCATTTATT[C/G]CTCAAATACATGTTGAGAACCTCCTATGTACTAGGTGCTGGATTTAGCTCTGGAAATACAGCTGTAAACCAAATAAATAAGGTCTAACAAAAATAAAAAT
12	57946285	BES4_Contig323_1373	0.33	0.37	CAATACATTCTTAATTATTTTTACCAAGGGTCAAAACTGTGAAGAAAAATGTGTATTGTAATGGTTGTGATACAACAAAGTAATTACATCATAGCTGTCA[A/G]TAAGGAGTTTGCAAATTAGCCTAGTAGGATTGCAAAACTATATAAAGACAAGTTTAAAGTGACAGAAAGATATATAATATATAGAAAAGATTAGTCGACC
12	61483846	SCAFFOLD129549_530	0.13	0.22	ATCCTGTTTTGGAGTAAATGTTAACACTTGCCATATCTCTGTTGCATATAGGTGAGTACAAATTGAAAATGAATGATAGGTTGCAAAATACCTATTAAAT[A/C]ACATCCTGGTGGATGACTGTTTATTAGGTTTCAGCATTCCTGAATCAGGTCCCATATTAAGTATTCAGAGACCTCTAAGCCCTAAAAAAGATTAATTAAT
12	61843920	SCAFFOLD275295_17649	0.14	0.22	ATATTCATCCTAAAGATAGTTGTAAGAATTGCTTTTTGCCTTTAGCATATCAAAATATTTCACATTCACACCTGATAGGTTCCCATACAATTTCATTACA[A/G]TATTTCATTATTTATGATGTTGTACATCTAAGATACAAAACTGTGGTTTCTCCTCTGTTTTGTCCTCCAAATCTTTTGGAACAATGTGCATGCAGGAATT
12	64122765	SCAFFOLD105804_4090	0.22	0.21	TACTTCATTTACAAACAAACACACAGAAAGTTTCTCAAAAATACTTGTTGAAAGTTGAGTTGGATACATAAGTAAAATCTAATTGTCTTTCAGATTTACT[A/C]ATGTGATCACTTCAGTGGTGGTGCTGGTGGTTTAGTAGCTCAGTTGTGTACAACTCTTTGAGACCCATGCAGCCTGCCAATCTCCTCTGGGATTTCCTCT
12	64306747	SCAFFOLD322357_3917	0.50	0.37	CAGAATATATAGCATTTTTAAAGTGAGGGAAATTGTCCTGTAAAATGATGTAACATAATCAGTTTGCTCGGGGCATACAGAAAGACTTTCCTGTGACCAA[A/C]ATTATAAATGTCTGAGCACTATTAAGTCTGAAAAGCAGACACAAACCAGAATGGGTGGGAAGAGAGACTGCATTTGGCACACCTTCAAAAACATCATTTC
12	67173304	SCAFFOLD159366_840	0.17	0.19	GTACACGTCTGTAAACAGAACGTACTGTGCTTTCTGAGGTTAACACCATTTGTTTAGTCTTGTTAAATAGCTGGTAAAATAATCCAGGTTGTAGAGAAAC[A/G]CAAACTGTAGTGACTGCTGCACATGGCAGATTAGGGACCCCAGAATGTCACAAAGGTTTCTGGGAGGAGAGATGTTCAGAGTCAGGTAGGACCCCTCCAC
12	67898103	SCAFFOLD185013_5107	0.36	0.25	TGAATAATTCACCAATTTTGTTTTCTAGTATGTGCCTTAAAGATACCTCACTAGTAAAACTGGTATCTGGCACAACAGAATGACATAGGCAGACTGTAAT[A/G]TTGTAGTGACAGTAATGAGGTTGACTGTTACACTTATTTAAACTCTGAGTATTAAATGCTGCATCCTAATTATTTATATAGAACATTGGCCCAATAACAA
12	70709740	SCAFFOLD105163_14904	0.16	0.17	ACCTTCTTTTATCTCTTTCTTCCTTAACATTCTTTCTGAACTCAGTTTATCTTTATTTCACTGAACTGACCTGACAAAACCATATACTTTGGACGCTGGG[A/G]AAAGAATAGTTACAAAAGATGGTGATATTTCTTATGTGAACTGTGCTCGCTAGGATTTTCTTGACATTATTTAAATATGAAGGTAATAAATAGCAAAAGC
12	72490094	BES7_Contig406_1227	0.33	0.37	TCTTTATTTCAATATTCACTTCAGTTTTTAAACACTTTGTTTTACTGAGGTAACATTGACTTATAGCATTATGTAAGTTTCATGTTACAACGTTCCGTTT[A/C]TACTACTGTATACCCTACGGCATGGTGGTGGTGGTTCAGTTCTTAAGTCATGTCCGACTCTTGCGACCCCATGGACTGTAGCCTGCCAGGCTCCTCTGTC
12	72490543	BES7_Contig406_778	0.02	0.01	TGCAAGCCTTGGCTTTCTGCTCTCAGCAATTCTCTTCTAGCTGTGCGGCCTCTGGAATTCACAGAGCCCACTTCACTCGGCGATGACTCACACTCACCAT[A/G]TAACAAATTGGTTCCATTTCTACACCATTTGGGGGCCTTAGACAGAAAGACCATAACCCCGTTCCACCACAAAAGACGGCTTCTCAGTAAAGAGTTTTCA

12	73401897	SCAFFOLD131110_4022	0.38	0.44	GCCAACGTTAAAGTCAGTGAATTATTTTGGTTAAACACCGATCAGCCTGGAACCTAGAGAAAGGTGTAAGGCACAGAAAAATGGGGGCCAGGGAAAGGGC[A/G]GAAGACCAGAGGTCGTGGATCTGGCCCCAAGTGTCCCCCCGGCCACTCCTCCCCCATGACACCTGCCACTGTTCAAAGTTTTGTGAATAAAGTCCAAAAC
12	74417588	SCAFFOLD135238_4049	0.07	0.08	CCTGGGGGAAGTCTGTTATCCCTCTTCCGGATGGCTTTCACTGGTGTGAGAGTAGGCTCTGGCTCCTCAGGCCCTGGAGAAACCCCTTCATCAGTAGACT[C/G]AGCCAGTTAGCTTATACTGCGGTCCTTCATATTGCTTGATCTGTTGAGTCACTGGTGACCAGAGGCACAGGGAGCAGAGGACACAGAAGGCCAACCCCAC
12	74417829	SCAFFOLD135238_3808	0.48	0.43	TCTTGACCTTCAGTGGGCATCCCTAACCTTCTAAAGCTGACCTTCAAAGTTCCGTTTCCCCCACAGCCCTTCCCAGGCCAAACCACCCCACTTGATGGCT[A/C]TTTCTGTAGCTTCTAATCCCTTAAGCACCACTTACAGATGGACTCGCTTGCACTTTTAATGGTAGGTCATTTGCACGAGTTAACCTGACCCCAACCCAGC
12	74684907	SCAFFOLD125326_1056	0.19	0.33	AGGATATCAATTAATTCAAGGGGACATGAAACAGATCACCAGTACCTCAGAGAAAGAGACAAATTAAAGTATGGAATTTTAATATCCTTGTGACTACAAG[A/G]ACTGAATAAAGAATACAGTAGCAATTTACCAGGTAGAGTTAACGGTTTCAATAAAAGCTACTAAAATCACATTTAGTTCCCCAGCAATAGGCCCATCTCT
12	74685056	SCAFFOLD125326_1205	0.04	0.06	CTCTGAGGTACTGGTGATCTGTTTCATGTCCCCTTGAATTAATTGATATCCTGTGATTTTTGTCTATGTTATCCCCTCCACTTAGCTCAAAGTCTGACAG[A/C]GAGTTGGTGTTCATTGAAAATGCACTGTGCTGTGGGTCATTGAGAATACAGAGATTTGTAAGACATTTGAGAGGTGAATTTACTGTTTAGGTGGCAGAGT
12	74685135	SCAFFOLD125326_1284	0.20	0.34	ATAATTAAAAGAACTCCTATGTGATATTAACAGTATTCTTTGGAAAAGCAAATTCTATGGTGTTGGAGCTGGATCTAGTACTCTGCCACCTAAACAGTAA[A/C]TTCACCTCTCAAATGTCTTACAAATCTCTGTATTCTCAATGACCCACAGCACAGTGCATTTTCAATGAACACCAACTCGCTGTCAGACTTTGAGCTAAGT
12	74942658	SCAFFOLD267044_4226	0.25	0.27	GTTGAATTCTCGTTTTAAAAAAAATAGTGCTGAGCCTGATACAGATGTTCCCACATTGCCCAGTCCTTTGATTAACCTTTTATGAAGTATCTGCCTACCG[A/G]AATCTCGATTAGGATGTTCCCACGTGTCTTATTAAAAGGCTGCCAGGCTTTATTGAACTTTGTGTTTATACTTTGTGTTCTGTGGACAGTTTTGGTGCCG
12	76877117	SCAFFOLD150166_15644	0.24	0.33	CAAAAGGCAAGCAGAGAATTACATTCATTTCAAAGATGAGCATTTTTTATGTTTGCACTGAATTCTGTGATTCCCTTTCTCTAATAAGAGGACTCAATGG[A/C]ATGTCCATTTGACTGAGTGAACTGAGGTGACAGTTCTCTGATCTTTGAGAATCAGCTTTGACAGTGAGAAGGAAAAGCTGCCAGGTTGCATGGCTTGTAT
12	77285854	SCAFFOLD161061_1683	0.28	0.20	TTAACTCCTCTTTTGTTTAATGTGCTTATTGGTATTTTCTTGGATGGAGCTGCCGCTGCCGCAATGACACATTTCTGCTGACAGCCCTGAACTTTCAGAC[A/G]GAATCTCTGTGAATAAAGGTGAGAAGGAGGCTTTAATTAATGTCCTAAGCCTGGGAATGAAAACAAAGAACAGGAAGGTTGGGAGATGGATCTGGCTGTC
12	78069273	SCAFFOLD136726_3272	0.38	0.26	AAAGAAAGTTTGGTTTATTAAGAATATTTTAAGAGTAGAATTATTATTAACAGATAGTAATTAATAAAGCTGGACATCCCATAGCTCATTACTATGAGTT[C/G]CATACAGTGGATTTTGATTACGCTTAAAAGGATCTTTTCATATGTTCCATCTCTGTGGTCTGGTCAGATCACAGTAATTTTCTTTATTGGAACAATCATA
12	78512046	SCAFFOLD152119_510	0.16	0.17	GAGCTGCACATGACTGAAGCAACTTAGCATGCATACATGCATGGACATGTAAAACTCTTCCAAGTGCATTCAGTTCCAATTCACTTCTAATGTTGTTGCC[A/G]GGACTTGTATATATGTTTCTATAACTCACACTTATTATCCATTGCCCTGCATGAAAATCTCTTATCATGCCCTAAATATCGACAGTGGCCTTTTCAATGG

12	78783490	SCAFFOLD300961_10786	0.21	0.11	AATCTAATGATTATTCATTTTTGACATGATATTTTTTTCCCCACTTCATGCATTCACATTTACTATAGGTGGGCGATGATAGATCACCCAGCTGGGATCT[A/G]GGGGTTTGGAATGCTTCTACTCTCGAAAGACTCTTCACAGTCCTAAAGAAATGGTCATTTCATCATTGAATCAGATGCTTGCATGTAATTGCATTTGGAT
12	79208194	BES8_Contig521_1197	0.24	0.33	CAGGAACCAAAGCCTGGCTCAGCTCTTGAGATGAGGCCAGCATCCAGGCTGTTTTTCTGCTGCATGCTGATTTTCTGAGACGTGGGAGTTCTGGAAGATA[A/T]GAACAGAATAGCCTATTAGCTCCAACATATGGCCCTTGTCTGATGGACAAAAGGAGTGGGAGGCTTCTTGCGCAGTTCCATTTTCTCGGATGTCCTTACA
12	79208449	BES8_Contig521_943	0.43	0.37	GAAAGCGTTGGCTGCTTGCAGCTTCCCTGAAGCCCGCCTCAAAGGTTAAGGAGTGAAATATGGGTTCTAAATAAAGACTTATCAGGTTACTATACATCAG[A/G]GCACTGATAAGACTGCCTTAGGAAATCGCCAAATGGAACAAGGACCTATTCAGAAAATGTTGGTTTGTAGATAGACCATCACAATGGAACCCATCTAAGT
12	79422522	SCAFFOLD161107_7544	0.24	0.17	ATCAGAATTTTAAAGCAGAAGCATGAAATGCAAGGGATTGCAAGTCTCACGGAAGAAGCAATAACACACTCCATGTTGAATGGCTGATATTTGCTTTCTC[A/G]GGCGCTTTTGATCTCAGAACACCTAGTGGGGCTGGGGTGGATGAGTTTTGCTGACTTGTTGCATCAGCTCTAAAGATGAAAGAAAACAGTTTCTGCATTC
12	79422812	SCAFFOLD161107_7254	0.44	0.37	CACACTGGATTTTGGTTGTCTTAAAGACAGAAGCCCCTCAGGCATAAATGAAAAGTAATAATAATTAACCACATGTTCTTCGTTATGCTAAAGGACTGAC[A/G]TGTGTCTCCCTACAGCTAACAGATATTTTAGCCACATATAAGAGAATCCTGTGGACAGAGGAGCCTGGTGGTCTACAGGCCACGGGGTTGCAGAGAGTCA
12	79592809	BES10_Contig668_769	0.15	0.21	AAAAATCATACAGCGAACCAAAGTGGCACACCTGCCTTCATCCTGTGGACAAAAAACGTGTGTGACTCACCGATAATCCACCCCAGCAGTTACAAGGGAG[A/G]TTAATTAAGTGGGAATGCATGGTTCTTGACTACAAACTTGTTATTTTTCAGCGTGAGTCATGCCATACAAATTAATGTTGTCTCCCAGACGTGCAGTTCT
12	80076729	SCAFFOLD269299_1521	0.39	0.37	CTGAACGGAAATTTTAAAAGGCTTCTTCTGGTTTACTCTTTTCTTCTAGTCTCTGAGAACGTCATTACAGATTCCCAAAGCACAGTGTGGCACCAAAAAC[A/C]AAAGCATTTGGCGTCTCGCGTGCCAGACTTTATACCTTAAAATCTGATATTCCAGAGTTTTTCATTTCTTCGAAACTTCTCAGCACAACAAAAACAAAAC
12	81260515	SCAFFOLD130379_11660	0.46	0.36	TAAGAGGATTTAAAAAAAAAGGCACAGGCAAGGAGAAATATTAACAAAACACATATTGGATAAAGAACTTATATCCAAAATATAAAAGAAGTCTAAGAAA[A/G]CAAACAGCTTTTAAAATGGGGGGAAATCTCTGAACAGTGTCCCCAGCAAAGAATCTTCTTTATTTATCTCCTAATATTCAGAGGGCAGATAAGCACACAC
12	84529022	SCAFFOLD170188_12460	0.46	0.10	TCCACTCTGGATTGGATCTGGTCCACTTTGCCTTCTGTCACTCGGGGAAATATGGAAGAATCTGCTCCTTTGCAAAACAGGTAAATTTCTCCTAGAAAAC[A/G]AGGTGAGAAGACTTTCATCGACTCACGCTATGGGGAAGATACCTTTCAAACGATGCTTTCATTAAAAACAAAACCAGGGGCAGTAGTGCCGAGGCTGTTG
12	85109167	SCAFFOLD115880_2637	0.26	0.13	AGGTCCGGCATCCCTCTGGGCCTCGCCGGCCCTCTTGCCTCTGTGGCTGCAGCTCCACCCCCTGTGTCTGGGGACAGATCATAAGCCTGAGTCTGGGGTG[A/G]CCACAGTGGGTCTGACCACCATGCTCATGAGAACCATAGGGCTTCTCTAGATTTGACACCGTTTAACTTTGCTTTCCTGTGGGTCAGGACTTCCCTTGAA
13	1712822	SCAFFOLD93087_505	0.16	0.34	AAAACTGCAAAGATCTGTTCAGTTGAATAAAACTCATTGAGCCAATGTCTGTTGAGCAGAGATTCAGTCAGAGTGAACAGGTTCCAGCAGTCATTTTGCT[A/G]TCTGACTTCTTTATGGTTCCTTTATTTTAATACAGAGATAAAGATTGAATATTTATCTAGAGACTACACAGACCTACATATAAATGTTACAAGTTAATCT

13	2087525	SCAFFOLD170799_16937	0.33	0.49	TGAGCTATTTGCGTTGAAAAGCAGCCTATTTTCGTCAGGCCTCTCCAAAGCAAACAAGTTAGTGCTGGCTTTGGGGACTGTGCATCCACGGGCTTCAACC[A/G]CAGCGGTCCAAAATTAAAGGTCTGTCAGAGCAGGTGGGTGGAAGGCAAAACCCAAAGGAATATGAACACTGTATGCCTCAGAACCTTTCAAGAGGATCAC
13	2088156	SCAFFOLD170799_16306	0.18	0.16	AAAGGACTATACAATGTAGTTTATAATATTTAAAAGGTGCAACATTTATGCCAGAAGAAGTGATGGTCATCAATCAGGCTGAGGTAACCAATAAAGAGTA[A/G]GAAGTGTTTCAACAGGGCTCCAAGTACTTTTTCATAAAGATATTTACAATTAGAAGATAGATTTCTCCTCTGAATCTTAACACTTCCAACTAGAAAATAA
13	3111153	SCAFFOLD200024_14429	0.44	0.41	CACCCAATGAACTGGAGCTGGTGCAGAGACACCCCAGCTTCCCTGTCGCTCAGTGGGGCACACTTCCTGCAAGACTGAGACACCAATAATAGGCTCACGC[A/G]CACATACTTACTGCCTTTCCCACCCCATTTCAGTACCCCCTCCCTTGCTGCACTTCCTCCGTCACCTCCCAAATAAACTACTTGTAGCCACATCCTTGCC
13	3676718	SCAFFOLD206273_10196	0.19	0.19	AGTGAGCTGTCACATTCAGATTCTCATTTGAAAAGAAAGTGAACGGTCACAAGACGGCTGGTGCTGTGGCTCTTACATGAGGTGCTCCGTGCGAAGCGCT[A/G]ATCAGCGCACTGCAGGGTGCTGACTGAAGGGGCTCATACATACATACACGCTAGTTACCATCACCGTTATCCCTGCCACCAAGGCACTAGGGTTTCTGCT
13	4862125	SCAFFOLD140462_11319	0.44	0.49	ATGTAGTAGGTTTGTGCAAATAACAGAAGCTTTTCCTGGGCCCAGTTCAGGTCATGTGGTGTCAAGACCAGAATTCCTCTTTTCTAGATACCAAAACCAT[A/T]CTCTTCTTGGAAACTTCCCACCCTCAACTGAAAAAAATGAAGAGATGATGGGCCTCCAAAGCACCCAGTTGACTGCTGGTTTTCTGTGGGTTCCTTAGT
13	5256365	SCAFFOLD103707_6915	0.24	0.31	AATAAAACCAGTGCATGAATGAGGCATATTTTGAGGTATGCAAAGAAAGAAATAGAAATATCTGGTCCTACAAAATAATTTAGTGTTAAAGTTATATGCA[A/C]TTTGGGACTATTTATTGCATTCCCTTGTAGTTCAGTCGGTAAAGAGACCCAGGTTTGATTCCTGGGTCAGAAGGATCCCCTGGAGAAGGAAATGGCAACC
13	5480551	SCAFFOLD255028_58009	0.22	0.22	ATGTTCACTCTGGCTGCTGAGGGTCCACTGGAGTGACCATCCAGGTTTAGGAACCAGCAACTTCAGCTGTGAAAGGGAAGAAGAGTCCCCGAGCCTGGTG[A/G]TAGACCCATCCTTATGTGTTTGAAGCCCCCTTTTCTGTTTGGGGCAGAAGGCAGGGCTTTTGGTGATGCGTCACCACCGCTGGTATCACAAGGCGGTTTC
13	7786379	SCAFFOLD125068_2484	0.42	0.42	ACAGTCTTAAGTAGTGTGTCCTAAGTCCAAATAACTGAGCCAGCCCAAAACTCAAATCCTTAAGGGGTCAGACAGATGAGAGGAATACAGAAGTATTAGC[A/G]TGTAAGGCTTAAGGAAGGGCAGGTTCTGCTGCGAAAGGAAATGCACTGTGTTCTTCCCACCACCCACCCCCGCCCCAGGGAATCCATAGTGTGCTGTCCA
13	8740419	SCAFFOLD84048_438	0.24	0.23	AGAATCAGTTGATCCCTAAGTGGATTTTCAGTGCTGCTGTCATCTTATGTAAGGCACACACAGTTACAACCTTGGGAATGAATGGCAAGGTAGGTGGGTG[A/G]CAACTGCCCTGCCTCCAGTGTGTGGTGTGATGCCCGGGGCCTGAGCTCAGGCATGCTAAGTGCTCAGAGCTGCAGGCGGAGGGGAGAGGTGATGGAGACC
13	9623598	SCAFFOLD285455_2244	0.49	0.40	ATACTATTGAATTCTGACTCATTGGACATGCTAAAGAAGTTAAACTCTTTCTTACAAAAGATTCATGCAGAGCCATTTGTAGAGTAAATACTCTTAAACC[A/G]TTTGTATGTAGACAAACATCTAACTGTATTTTGTGACACAAAGTAATAAGAAGTAAACATTCATTTAAAATACTATTAACTTGATTATGGTAATAGATTC
13	11064271	BES8_Contig508_777	0.13	0.30	GTCCCACCGGACCTCTGTACTCTGTGTCCCTCTCTCCCACACCTGGTCCAAAGCTAGCCCAGCCCAGAGACAAACTGTAAAGCAGGTAAAGGAATCTTCA[A/G]CTTCCTCTACCTGCCCGCCCAAATGTCCACCTCACTCCTTTCCTTCTTCCTGTAGGCCCCAGCCCAGCCCTATGCACTCCTAAAGCTTAGGCTTTAAGCG

13	14206536	SCAFFOLD135036_36271	0.11	0.06	GAACTCTACTATAACATAACATTTTTCATAACTATTGATATATATTGAATATAAAGGTTGTAACTATCACTCTCATGAGGCTCCTTGTAAATTTCAAATA[A/T]GCATTTGTAATGAATTTGATCTTCACATACTCTTAATATAAATCTGAAGGTGTGGACAAAGAGTGTCTCCCTGTTACTCAATTAGATGACTCAACAATAA
13	14809237	SCAFFOLD70189_17695	0.02	0.05	TTAGTTGATCAGGTGGAAAATTTTTTCATGCTATGACATGTTTGGAATGTTCTAATATGAAATACTAATTTAGGAATTTTTCTGATTATTCCCAATAGAT[C/G]TGACAGGTTAACTCTGTTAGACACTAAACACTCAAAATAACTGGGCTCCAGGCACACGGCACAGTGAAGCTAACATATAACCATAGCCCCTGAATGCACA
13	17514586	SCAFFOLD65435_9187	0.43	0.47	AGTAACTCTGGGGTCAGGAGCACGAGAGCGCCCACAGCTTTAGAATTATGTTTTATAAAATGATGGGGAAGTCAGTCTCATTGCCTTTGGTAAAATCAAC[A/G]GATTGATTATCTTCTTGGCAATGAGCCAGCTAATTATCATCTACAGCAAATGTGGGGCAATTCGTTATTGCTATTTTTGTATATGGTTAAAGAAAAGACA
13	17514773	SCAFFOLD65435_9374	0.22	0.29	AAGAGTTGGACAACAGAATGAGCAACTAACACTTTTCACTTTCAAGATAGTTTCATGTTTATGTCCCAGTTCAGCTTCTGGTCTTGGGTAAATGAAGAGC[A/G]TAGAAGATTTAAACTTTATCCATTTTGATGTATTAGTAACTGGTCCATATAAATCCTGGCACCAATAACCAATCTAACTATTAAGCAGTAACTCTGGGGT
13	18933945	SCAFFOLD145172_21009	0.38	0.39	ACCAAAAACATACCAGGAAAGGAAAAGGCTGGAGAAAGTCCCTGTCCTGATTCCTGTGTTAAAGCTAGTATTTTATAAGCATTTCTTATCTTGTTTGAAA[A/G]AAATAGAGAACTTAAATATTCTGGGATAAAAAAAATAGCTTATGTTATTTAAGTATACGTGCATATGTGCATTTAAATAAATTGGAAAAACAGGAGAAG
13	18972619	SCAFFOLD5373_38904	0.10	0.06	GTGGGTTCAACTGTGGGCAAATTATTCAACATTTTTTGCTGAAGGATCCAGACTTCCTTTTCAGGAGTGTAGGCAGAGCCTGTCTTGCACTCATTTCAAG[A/G]TATATAAAAATGACTAATTCCCAGGGAGAGTTCCTAAGCAAGAAAAAAATACCAGGATGATTAATCCCCTCCTTTCTACATCTATGGCTGTTTCAGTCTT
13	18973819	SCAFFOLD5373_40104	0.36	0.28	CAGGACAGACGGAGGCAGGGAGCGTGGCCAAGCAGGCTGGCTTCTGACACTCTGTCCAAGAGTGCTTGACCACATTCATTGCCGAAGCGCCTCTCAGGAC[A/C]ACACTTAACTTCTGAGGGGCAACAAGGGAGGGAGGGAGAACAGATTGAAGTATTTGGTGGCAGCACAAATGACACGTGTCGTTCTCGGGATAGTTGCCAT
13	19145474	BES7_Contig502_1131	0.24	0.27	ATGGTTCATAGGGTCGCATGTTTGGAGCTGAGCATCACAGGAGTTAGCCTACAGAACAGAAGCTTTCAGGATTAGTCTAAAATAGAGGCAGTATTTTACC[A/G]GCAGCCTCTTAAAAGCACATGACTCATTCAGAAATACAACATTTGGTCGATGACTATGTCAGACTTTCAGGTAAATTTAATCAATAGACAACCACTATTT
13	20842651	SCAFFOLD150225_6727	0.43	0.50	ATTATTATGCACTCATAGAATGGAATGCCGTGTAGCCTTTAAACAGAATGAACAGCTCCATAAAAATCAAGGCGCAGAACAATGTCACACCCATATATGT[A/G]TGCATGTATATGATTGCAGACAGGGATATTTAAACATGCATAAACTATCTCTGAAACAGTTTGAAAGAGATATGATTTTAAGTTCACAAAGAAATTGCTG
13	21918984	BES1_Contig330_454	0.39	0.36	ATAGAGTAATAGGGGAAAAACATGTTTGACAAAGACATTTAATTGTATGATTTTCACTTTGAACTTCTGAAAAACTGTGTATTCCTATTTAAAAATGCCT[A/G]CCACGTTATATGACCTCATGTTAATTTTATTTCTGTGCCTTGATGTAATGAGATAAATGTCAAATCCAAGTGTATGTATTATGCTGAAGAATCTGGTCTT
13	21955039	SCAFFOLD100910_4645	0.39	0.49	ATGAGCAGAGGCTGAAGAATTCTGAAAGCCAAAAGGCCCTCTCTTTTGTGAGGCTGGCCCCAACTGAAGGCAGACAAAAAAGGCAAAGATGAAATAAAAC[A/G]GACTTTGTCTTACTAACCTGGCACACATGCCTGCTGATCGGTGGCTTAGCTGGCAAATTGCAAGGACCTCGATGGGAGGTTGAAAACTCAACAAGTCACT

13	22615125	SCAFFOLD120395_9107	0.31	0.36	GAACTGCATGGAGTGTGTTGAGGAGAGGTTGTGGAAATGCTGTGATTATAACCACTGCCTTCAAAGTATTTTGCTTTGAAGGGGAGCAGAGAAACAGGAG[A/G]GGAATTGAAAAGTATTCTGGGGTCATTTGAGGTGTTTTGATTTTATTTTTCATAGGACAGATCACAGCATGTTTGGATGCTGGCAGGAAAAGTCTAGAAG
13	24831806	SCAFFOLD140080_30362	0.40	0.47	GCTTCCACCACCTTCTATTCCTATCCAGGCTCAGTTCCTGACGCTAGGACTGAGGACTTCTCCTCCTTATAAGCATCAATCAGAAAATGCTGGGTTGACA[A/T]GCACTACTATCTATAAAATAGATGGCTGGTGGAAAGCTGCTGTATAGCACAGGGAGCTCAGCTGGGTGCTTTGTGATGACTTAGTTGGGTGTGGTGGAGT
13	24856643	SCAFFOLD140080_5028	0.41	0.48	GATTTCTAAGGAAACCAACCAATCCCATGAGGACTGAGAATGCTGGAAGAAGAGCAGCCCTGCTGGCCTCAGAGCTGAGGAGCCTGAACTCGGCTAGGGC[A/G]GAATCAGGACCAGCAGACGTCTCAACATAAGGCCCAGTTCTGTCCATTCTGCCATGTGGCGCTAACGCCACCTGCGGCTTTCCCCCAGTTAGGGGTTTCA
13	25009393	SCAFFOLD150469_7997	0.47	0.44	TGGCACAGAACGGACATTCGATAAATCATTGCTCAATAAGTGAACAAACTTCTTATTTAAATCTTACATCAAGCTTTCGCTTACAACTAGTTTGAAAGGG[A/G]AAAAAAAAATTCTTTTCAATTTAACTCACTTAAAATGCAAACACTTAATTCCAAACAAGTTGGTAATCAGGCTAGGTCCTTATGTAGCCAACTCAACATT
13	25009472	SCAFFOLD150469_8076	0.47	0.44	ATACAGCAAAACTTATTAAACCTTCAAATCTCTAGCCAAGAAAAAAGTAAATGGTTTTCTTTAAACGCCCACTGCTGTGAATGTTGAGTTGGCTACATAA[A/G]GACCTAGCCTGATTACCAACTTGTTTGGAATTAAGTGTTTGCATTTTAAGTGAGTTAAATTGAAAAGAATTTTTTTTTCCCCTTTCAAACTAGTTGTAAG
13	25009905	SCAFFOLD150469_8509	0.47	0.44	CAGAGTACAACTCTTAGCAGTTAAAATAGTGTTTGCTATAAGACTGGCCCTTCTTTTAGTGTACTCCATCTTCATATATTTTCAGGCATAGTATATTGAA[A/G]AATTGGTCAATTTGTGGCAAAGTCTTTCTGAATTTATAGTGGTAGTCATTGCCAAGTTACTGTTTCCTTAATAGAAATTAAAACCAAATTATTTGAACCG
13	25319293	SCAFFOLD120821_3986	0.14	0.10	TCCAGCTCGGGGCTTCAGACCAGAGCCGGCTGCAAAGGATGAAGTGAGATGGCAGAGACGAGGGCAGTGAATACATTTAAAGGGACAGCCGGGCCCTGGA[A/G]GAAGGGACAAAGAACCAGGACCCTCTAAGGGGGAAGGCTGAGATTAGGGTTTGAATTAACAAAAGATTCATAATAGAAGGCTCGTGAATCTGACAAGAGC
13	25319337	SCAFFOLD120821_4030	0.35	0.30	ATTCAAACCCTAATCTCAGCCTTCCCCCTTAGAGGGTCCTGGTTCTTTGTCCCTTCTTCCAGGGCCCGGCTGTCCCTTTAAATGTATTCACTGCCCTCGT[A/C]TCTGCCATCTCACTTCATCCTTTGCAGCCGGCTCTGGTCTGAAGCCCCGAGCTGGACTCAGGCCTCTCCGAAAGACTTATTTTGTTCATCTTCAAGTTCG
13	26448943	SCAFFOLD56245_9129	0.17	0.20	AGAAAGGGAAGGATGGAAAAGGAGGAAAGTCATTACGGGTATGAATACAGAATCACTGCCAACAGGATTTTTAAATTCACCTCCATTTCCTGGCTTTAAT[A/G]GTCTCAAGTTAAGGATTAACTTTATCCATGTTCTTCAAGTTATTGTTTTCCCCCAAACCTGCTTTCTTGGCTCTGTGGAGTCCAAGAAAGAATCAAAGGG
13	26448988	SCAFFOLD56245_9084	0.18	0.20	TGGGGGAAAACAATAACTTGAAGAACATGGATAAAGTTAATCCTTAACTTGAGACTATTAAAGCCAGGAAATGGAGGTGAATTTAAAAATCCTGTTGGCA[A/G]TGATTCTGTATTCATACCCGTAATGACTTTCCTCCTTTTCCATCCTTCCCTTTCTTCCCACAAAGATGGTGCTGGGGGGTGGGGGGAGAGCTGAGAATCA
13	26449087	SCAFFOLD56245_8985	0.18	0.21	AGTGATTCTGTATTCATACCCGTAATGACTTTCCTCCTTTTCCATCCTTCCCTTTCTTCCCACAAAGATGGTGCTGGGGGGTGGGGGGAGAGCTGAGAAT[C/G]AGGTTTAATATCGTGAGCTGAATGCTTATCCTTAAAGTAAGTAACTTGGCCACTGGGTTTCCTTAGGTGTTCAATTCAAATCACTTTTTCTTGAAAAGTT

13	26449195	SCAFFOLD56245_8877	0.25	0.25	ATATCGTGAGCTGAATGCTTATCCTTAAAGTAAGTAACTTGGCCACTGGGTTTCCTTAGGTGTTCAATTCAAATCACTTTTTCTTGAAAAGTTCGGCTTT[A/C]TTTTGGTTAATTCCCCCCTGCCCCCGTTGCTCCTTTATTCTTCCAGGTATAAACTGTGCGAAGGCTTCCACATGGAGAAAACCCTGAGCTCTACGTAACC
13	26449315	SCAFFOLD56245_8757	0.19	0.26	CACGTATGAGTCCATGAAATTGATAATTGTCTTTTTCCCTGAAAATGATCATGATTGCTTATGAGTCATTTTGTAATGAATGCTGAATCATTGTTCCCTC[A/G]CCTGGGAGTGGTGATCAGTGGTTACGTAGAGCTCAGGGTTTTCTCCATGTGGAAGCCTTCGCACAGTTTATACCTGGAAGAATAAAGGAGCAACGGGGGC
13	28618368	SCAFFOLD50203_5035	0.21	0.15	AACAACTGGGGAAATAACAAAAATCAGTTCACATTTAGATTATCATTTCACACTATCAGGTTGGCAATCCTAAATTTTAATGAAGATGTAGACAGAGCAG[A/G]TAATGCTTGTCCATAGCTGGTTAGAGGTTAAATTCGTGCAACTTTGTTGAAAATATTTTACATTATACATGTTGCATTTACCCCAAGCCCCGACAATTCC
13	30552086	SCAFFOLD110358_14004	0.28	0.24	GATTGGACAAATTAAATAGTTTTCTTAAGGTTGTATAGTTACATTGGATCATAATTGTGATTTGAATCCAAAGTAAATGGTTTTAAGTTTCTGGAGACAG[A/G]GGTTTTAACTTATGGTTTTTAATTATTGGGGACTTGGTATCATCAACTATAAAACATTGGGATTGGATAGATCAGTGAGTAAACAGCAATCTGTAAACTT
13	30554457	SCAFFOLD110358_16377	0.29	0.26	GGGGCCTTAACTAAACAATGTCTCCCCCTCCTCTGACTCTCAACACCTTTGAAAGAGACAATATGAAGGTCCACTATCCAAGGGGAAAGTAAGTGTTCGT[A/G]GCACATGTTCAGGCAACATGAAAAATAAAATACAAATAAAAACTTTTAAACTAACTGTGCATTTGGAATCTGTTTCATTCAGTCATGTCCTTGTGTTCCC
13	30574710	SCAFFOLD206_10369	0.29	0.18	CAAACCTTGCCATTTCCCCAAGAGTCAGGCTTTGCATCACTCAGAAGTCAGACCCAGTTTTTACTTAAATGATTTGGATGTGGTTGATGCTAGCAGCTGG[A/G]AGATGAAGGTTTTATATACTACGCTGTGGAAAGCATCACTTGTAACTCTTCAGCCTGGTTATTCTCTATTTAAGAAAAAAAAAGTGAGCAGCTTTGATGG
13	31355972	SCAFFOLD161794_8551	0.11	0.12	CTGCTGTCATTCGAGGCCTACATAATCCAAGCCAGAGAATGCATCAGTAATTATTACATGCAGTTTTTTAAGTCTAGAGAACAAAGTTTCTCACTCAATA[A/G]CTATGGACTGCATACACTTTTGCTTTGTGGTCATCTCAGCTGAATTCTCCCTGCTCACTTGTCATTTCTTTCTTTGCAAATTTCCTGGATATGCCTCCTA
13	32879631	SCAFFOLD130253_23585	0.48	0.42	CGTGTGGAAAACGTACTTAGATGTGTGGCAGGTTCTTCTGAACCTGTGGTTGTCTATCCTGGCTGCCGATTTAAAACCAGCAGAGGTTTTAAAATGTAGC[A/G]AACTTAGGAGATGGCCAAAAAAATTTTTTTAATTAAAAAAAAGTCATGGACTTGTATCCCACCTGTGATTAAGTGAAGCCCAATGTCTAGGATGGGACTT
13	32983769	SCAFFOLD210023_26639	0.50	0.48	CACAAACTTTTTGGGTTAGAACAGACCTCTTAAAAAAAAAAAAAAAAAGAACAGACCTCTTAGCATTTTAAGGATGAAGATGACGAAACAAGAGAGGCCC[A/G]GAGGGGTTGAGTAACATATCCAGTTACTAAATAAACTCAGAATCAGAAGTATTATGTTCAAGTTCTAGCATTAGCACGCACCATATAACCTCGAACAACA
13	32984104	SCAFFOLD210023_26974	0.50	0.48	CTCATCAAATAACAGATTTTTCACATGTTGTACTGAGGCTAGATGCGTATTTACTCTGACCGTGGTGAATGACGGAGGGTGAGACAAGTTATGTAACAGT[A/G]TTTCAAACCTCCTTTCTGCTTCTTGTTTACCTAAAGCGGACAGAATCTGAAAAGAAGAAAAGAACAAGTTACTGTTTCAATTTCATAATACACATTAAAA
13	34231448	SCAFFOLD188701_463	0.48	0.05	GATGCCTGCTTGTGAGACAAGCTGGGACCTGGGACCCTTTGCTACAGGGCTCACACCTGGACAAAAGTCTCCTCAAGCCACAAAATACAAAAAACTATAA[A/G]GGACTAAAAATAACTGTGCGCATGCATAGCTAGGGCAAATTATGGACAAGAAGATACACAAAGACCAAAAAGAACCCAACTTCCACTTCTGAGGAGGGAG

13	35683787	SCAFFOLD269966_1382	0.17	0.11	CTATAAATTTTCTTATGTCTTCTTGGTTATTAATTAATGCGCTAATGATGCCAAGAAATTTCTTAGCTGGAACTTGGGATTTAAAGTAGCTCTTCTGAAA[A/G]AAAAAAAAAAAATCCTTGGCCAGATTTCCTTTATAGACCTAAAACTTTAATACTTACTATATAATGCATGATTTGTCCTCATTTTTGGAAAGCTAATGAA
13	36879180	SCAFFOLD70508_2761	0.46	0.09	GAGTAATGTGAAACAAATCACCCAGGTGCTAGTAGCACTTGGAAAGCACAGGGAGCTGCCTGGGGATCGGTGTCTCATCAGCAAAGAGCGATGCTGGGGT[A/C]GAATGTGTGGCTCTGGAAGTCAAACCAGTGTGGCATACGGTGAGTGCAGAGGAAGCAAGGCACCCTCGAAGCTCTGACCAAATCAAGACCCTTTCCCTTT
13	37833209	SCAFFOLD1473_926	0.35	0.39	CCTGGGCCTTCCATAGCCTGATTCATGAGATATTTACAGCTGGCTTTTTGCTAATTTTAAAATGTTGCCATGACTTTCGGATGGAGTAGCTAAGCTGTAC[A/G]TTCCCAGGGAGACCAGTCATCATAGCCCCTCAGTGGGGCCAGGGCTAGGACTGGGGATGTGGTGGGGATGTTGTGGGGCAGCACTCCTTGGCTCTGCCTA
13	38040023	SCAFFOLD265686_4100	0.09	0.17	AGTCCTGAAGGCAGTGGCCGGGCCAGTTGGCTCCAGAGCTGAGTCAAGAAAGAACATTGACTAGGACTTGGTGGAAGACAGAGCCTCCAGGGGAAGAGGC[A/C]CCTGATTTAATAGGAAGAGCCCTCCATGCTGGCCGCCAACTCAATTTCACACTTATTTATTGAACGCCTCCCCATGCCAGACACTGGGCCAGGCCCAAAT
13	39533528	SCAFFOLD276847_9095	0.15	0.25	GATCAAGCCTGCGTCTACCTGCCTTTCCTGCACTGAGGCGGGTTCTTTACCGCTGAGCCACCAGGGAAGCCCCATTTTATTGCTTGCATTGAGGCATCTC[A/G]TGGAAGTGGGAAAACAATCTCCCCTGTGTTCGCTGACTCGGACATTCATCCTGAAATTGTGAGTTAGGGTCGGCCTCTGATGAGGGCAGTGGGGAGGAGC
13	41346482	SCAFFOLD245014_15993	0.15	0.18	CTTGAAGGGTGAGTTGTGGCCCCAGGTGTGGGGGATGGGTTCAGGGTGAGAACAGCCTCCAACAGCAGACGCATCAAAGAGCAAGGAAGCACACGTGTTT[A/G]CTGGCCACTGCCAGTCCCTCCTTCTGTGGGGTTCCTCGAGGAACACCAACTCTCTGGCATCTCCAGCTTCCCTCAAACTCTGGGCAAGTTCTCAGTCAGA
13	43109828	SCAFFOLD128306_4159	0.10	0.26	TAAAACAAAGGGGACAATGCTCCATGGTTGAGAAGCAAGAAACAAAACCAAAAAAACAAACAAAACTGGCAGCAGGGGCCTGGCCAAGCCTATGACATAT[A/G]CATCAGACTGAAAACTATCAATGATACTGAGGAATCATATTTACTCGGCAAGTTGTTGAAAAACTACAAGTGTTACAAATGTTGATGAATGAAACAGAAG
13	43109972	SCAFFOLD128306_4303	0.12	0.26	CTCGGCAAGTTGTTGAAAAACTACAAGTGTTACAAATGTTGATGAATGAAACAGAAGAGCCACAAAACTGTACACTCAACATGCTTTGAAAGGTGTGCAT[A/G]TATATTCAAGAACAGGAGAGGATGCAGAAAGACATACACCTGGATTCTTAACACAGTTAGTAATGGACGTGATGAACAAGTTCTCTCTTGTGCACCTTGT
13	43470740	SCAFFOLD313617_4437	0.22	0.23	TCTAATAGACTTCTATGTCCAACTGTATGCATGCATCTTCACTTGGGTGCCTAATGGCATCTCAAAGATACATGTCTGTAATTGAACTCCTAATCTTCCC[A/G]CAAAAGTGTGCTCCCCCACATTGTCTCCCTCTGAGAAAATGGCATTTCCATGTCCTGTCCCTTAGCCACAAACACCTGGAGTCAACCCTGCTTCCCCTTT
13	44301095	SCAFFOLD40198_4164	0.10	0.18	TTTCCACAGCTGAAAATAATTAAATCCTCTCATAAAGCTGAAGTGCATGAATTCCTTGGTAGGACAAATAGTTATTCTGCAGATGACAACGTGGAGAGTC[A/G]TCGTCTTAATTCCAGGTGTTTCTGAGCCTTACTTTCATCAGGTTAAACTGCTAGTAATTTAGAGTCAAATTTGCATTGATAAAACGAGTGGTCCCTCCTT
13	44751599	SCAFFOLD45360_28738	0.20	0.21	CCAGCCCCAGAGGTGTGCTGAGGGACAGGAGGGCCCTAGTCGACGGGCTGTGTTGTATATGAATCTGAGTTTCCCCCTTCAGCCTCCTTCACAAGGACAG[A/G]GCACTTAACTCGGGGACCACGTCTGTGCCACATCCTCACCCCTCTGCCTTCGTAACCTGCAAGAGCAGAGTGGCCTGGAAGACCCAAGTTCAATCTCCTG

13	45766259	SCAFFOLD150507_14530	0.22	0.38	TGATATTTCAAGCCTTGTAGGTCACATGGCGGGTGAGGGTGTCAGCAGAGGCTGACAGACCGCCCTGAAACACACCAGCAGCCGCAGAGACAAAAGCTGT[A/C]TGTGTGGAGAAACCATCGTCAGAATACAGGGAAGTTCTTGGCAACAGCCAAGTACAAACATGGTGGGACCCAAGGGAGCAATCTTTACACCTGCCCAGCG
13	46918409	SCAFFOLD240740_7358	0.41	0.35	TGTGGGGTCGGAACGTGCAAACAGTGTCTGTAGAGCGTTCCTCTCAGCAGCATGTGTATCGGAAAGGCCAGGGCTGCCTACCTTGTCTCTGAAACTCTGT[A/G]CAGTTCTCATCGGCCATCCCAGCCCTGATTCCAGTCAGCGCAGTGGCCAGAGTCAGCTGCTGCTAGTGGTGGTGCCTTCCGTGTGTGAGCAGGGTGATCT
13	47210789	Prnp02	0.22	0.31	CAAGACAGACTGAAAAGTGACAGCCAGTGAAGTAGGAGGATATCAAGAGTGTGAGAGAGGAGGTGCCTTTCAAGAAGGCAGGAGTCAGCTATAGAGAGAC[A/C]AAATAGAATGGAGGTGGGAAAGAACTCACTGGATTTGGTGATGTGGAGGTCGTTACTGGGGCCTTGATGGGATTAGTTTCAAGAGAGTGATAAGAACAGA
13	47211732	Prnp03	0.50	0.00	CTGATGGGGCTTGGTGGTAGAGGATGTATTCGGAAATGGTGAGAAATGGAGGAAGACCAAGTTTTAGACGCAACCATTTTTAGAGAAGTTTCTTCCATTT[A/G]GGGTATGTTGAATTTTAATGTAATCTCCAGATTGGGAGGGTTGGTGCTGAAGATGTGCATTTAGAAATTGTCAGTAAGAATCTGCCTGCATTATAGTAGA
13	47211800	Prnp04	0.50	0.00	AGACAACAAAGGGAATACAGGAAGCCACATAGAAAGTGACCAGGTCATGTAGGGCCTAACAGCCAATGACCAGGTCTTTAGATTTTAATTAAATGTGATA[A/G]GAAGCCCTTGAAGATTTAAGCAGGGTGGTGACCTGACCTGATTTCAAAGACCATTCTGATTGTTGTGTGGGGCCTGAGGGGCACTGATGGGGCTTGGTGG
13	47212041	Prnp05	0.30	0.45	GACCTGGTCATTGGCTGTTAGGCCCTACATGACCTGGTCACTTTCTATGTGGCTTCCTGTATTCCCTTTGTTGTCTAATGTCAGAAACTATAACTATCTA[A/G]TTCACACTAGGTTCTCTATAAATTATTTGCTGAACAAAATATTTCTTCTTTTGAAAATAAGAGAAACATAGAGTTTACTTCATTAGCTTCTCCACATTTG
13	47213900	Prnp06	0.50	0.00	AGAAACTATAACTATCTAATTCACACTAGGTTCTCTATAAATTATTTGCTGAACAAAATATTTCTTCTTTTGAAAATAAGAGAAACATAGAGTTTACTTC[A/G]TTAGCTTCTCCACATTTGCTGAGGAGGATCTATGTGATGTTGACAGGTAACTTCAATTGAGCCAGGACACAGGAGATGCGAAGGGAGACTTTCAAAGAAT
13	47213958	Prnp07	0.22	0.32	GAGGCCTAGAAAGTATATTAGCCATCACCTGCCGTCATTTACTCAGTTCCTGCTCATTATCTACAAAATAAATGATTTAGATTATGATATTCATAAGTGA[A/G]GGAAATGCACTCCATTTAACAAGTCCATTCTGCACATTACCTGAGGTTTCTTGTATAAAACATGGTGTGTACTTGATCATTATTACTGCCAAAATTTTAT
13	47214453	Prnp08	0.05	0.09	CGCTGGGAGTTTAAAGGACTACGCGGCTATTTACCTGCTTCTGCGAGAGAGAAGACGCTCTCAGCTCTGCGGCTGTCAGCGACTGGCAACTAGGGCGGGT[A/G]AGTCGCCGGGCGGAGGAGGAGGGGCAGGGGCGGGGAGGCGACGGCGCGCGCGCGCACGCTCAGGCGCACCCGCTGCCGGCGCCCGGGGTTTACTGGCAGA
13	47217116	Prnp11	0.00	0.02	GAGACACAAATCCAACTTGAGCTGAATCACAGCAGATGTAGGTACCCTGCAGAATCTCTTTGGTCTTGTGATGGTTGAAAGTGCCCAACTGTTTCACAGA[A/T]GATAAGGGACTGAAAGGCTGGGATCACAAATCCTTGCTCTGGAGGCCACTGAAATCTATATATGTAACCCACACCTATTATATCACTCTTTBTTGTAAAA
13	47218934	Prnp12	0.39	0.48	TCCATAGCAGTCTAAAATGCAGCAATCACTTACATTCTTATCTTGTTAATCTCATTCACGAGAATGCTTTTCCTCTTTCTGTTAAGATAGGGTGTGGAAT[A/G]TGTATTACTTCTAGTTCTATAGAAATTTTTTCAACATCTTAAACTTAAAAAGTCAGGAAAGCACTGATTCCTGAGTTTAAGTGAGAACTTTGATTTTAAT

13	47220088	Prnp14	0.27	0.47	CTAGACACTGATATTTGTTTTAAGACATTAGTCTGCCATTTTCTCTGGCAGCCAGCTTTCTAAGTAGTCATATTCCTTGCTTCATAAAAAGAAAAAAAAA[A/G]AAAGAAAGAAAAATCTGTGTGGGAGGGATTACTTTTCTATTCAATTATAAAAACTGATATTGAATTCATTCAATTAAATAAATATTTAAATTAAATAAAT
13	47230627	Prnp20	0.50	0.01	TGCTCATGGCACTTCCCAGCATGTAGCCACCAAGGCCCCCTACCACTGCTCCAGCTGCAGCAGCTCCTGCCACATGCTTCATGTTGGTTTTTGGCTTACT[A/G]GGTTTGTTCCATTGACCGTGGGTACCACCTTGACCCCAGCCTCCACCACCATGTGGCTGTCCCCAGCCACCACCATGGGGCTGACCCCAGCCACCTCCAT
13	47230864	Prnp21	0.18	0.08	TGCACATTTGCTCCACCACTCGCTCCATCATCTTGATGTCAGTTTCGGTGAAGTTCTCCCCCTTGGTGGTGGTGGTGACTGTGTGTTCCTTGACTGTGAT[A/G]TTGACACAGTCATGCACAAAGTTGTTCTGGTTACTATACTGATCCACTGGCCTGTAGTACACTTGGTTGGGGTAACGGTGCATGTTTTCACGATAGTAAC
13	47231587	Prnp22	0.39	0.42	GGAAGGCTGCCACCAAGGGCCCCTCTCCCGTATGTTGGAATTTTTTAGTCTCAGAAGCCTTTCGTATTATGTTCATTCCTTGTGAAAATGTCCTCTGCCC[A/G]TAAGTCAAAGGTTGCATGAATATCCATTCTTGATGCAATAGCTGGCTGTGTTACCTGCAAATGTAACCTGCAAAATGCAGAATATCCTTTATAGTTCTCT
13	48371647	SCAFFOLD60595_7917	0.02	0.04	CCTGAAAACAAAGAGACTTTAAAATCACTTAAAAGCCATAATAATGTAATTAAATCACTGGATCTTCCATGGATCTATGTGGACCTCATAGCACCTTTGA[A/G]GCCCTCACTACCCTTTTCAGTGGTGAAAGTATTACAGACACAGGCTGGAAACTAGGCTTCCCCATTCACAAGGCACAAGATCACACTCAAGAGTTTTAAA
13	53707148	SCAFFOLD225588_7603	0.21	0.22	TTGCTTCCTCTGCTTCTGTGTTATTTGAATCAACTCATTTGGCCTCAAGCACTTAATCATTTCATTCCTCTTTCTAGTTTTATTTTGAGGCATTAAGAGG[A/G]AAGAATCGCCATGGGAGCTAAAGAGGGCCTTTTCCTCTTTCTAGCTGTTGCTTCTGGATTCCCTTACCCAAGGCCCAATGCATATGTTTGGCCAGATGGC
13	55631070	SCAFFOLD121913_8423	0.45	0.50	GTGAGGCCCAGCCCCACGGTCTCCATGCTCCTCCCGCAGCCCGCAGCTCAGGACTCTGCTTCGTCCACCCTCCATCAAGGGTGACCCAGGCTGAGAGGAC[A/G]AGTGTTACTGCCACCAGGAGCTGCCCGGGGCTCTGGACAGCAGAGGGTCAGGATTTAGGGCAAGGGCCCTGGGATTGGTGAGTGACGCCTGCCAGGGCAT
13	56485244	SCAFFOLD185077_2460	0.10	0.26	GGGTGGATTTTAAAGGCAGATCAAATGGGAAAGAACCATTTCTCTGAAATAAAATGTTCTCCCATTGTGATGTGTAATATTTCCCCAAGAAATAATCTCC[A/C]TAATTTTTCCAAATGAGATTTTGGAAATAAATCTCAGTTAGGTGACCAGGCAGTTCTAGGTAAGTAAGATATTTCAAGAAGATGAGTTTCTAATATCCGA
13	56485316	SCAFFOLD185077_2388	0.39	0.26	GTCTCTCTGTGCATGGCTGGAGGAAACCGAATTGATGAGAGAATTGTCAGAAATGATTCCGCATGTGTTGTGGGGTGGATTTTAAAGGCAGATCAAATGG[A/G]AAAGAACCATTTCTCTGAAATAAAATGTTCTCCCATTGTGATGTGTAATATTTCCCCAAGAAATAATCTCCATAATTTTTCCAAATGAGATTTTGGAAAT
13	56901562	SCAFFOLD135401_8690	0.29	0.42	ACTTCCCCCCACCTCTCACTCCAGGCAGCAGAACCAGAGGCAGGGAGAGAGGATGGGGAGTCAACCAACTGGAGTTGACTGACTTGAGATTGAAAAGAAG[A/C]CTGTTTTATTTCCATTCTTGATCTCATGGAATGTATCTACTTCTATCTTAATTAAAAAATAAGAAGAGACAATAAAATGCGTTATTACAGATTGGATGGG
13	59892951	SCAFFOLD5083_24631	0.25	0.30	TTGTCTTACCCAGCTCCGTTCTCCCTAATTTATTCTCTGGTCTTGGGTGAAGCTCTTGCTGAGATTTAATAAAACTCATATTGCTCTGAACCCTTGTGTC[A/G]GCTGCGCTGGTCCCTCTAAGCCAGGAGGGGGCAGCGTTTTCCTGTGGAGCAAAGGGCTCCGAAAGCAGGGGATTGGGGTGGGGGCAGGGGGCAGACTATC

13	61543380	G73153-333-Y	0.24	0.11	GTTAGCTGCTTCTCTAGGGGCTCTAGCTACTGAAGCTAAGGTGCAAATGTAAACCTTTCTTTACTCAGGAGGGCTTCTACTTCTCACTGATG[A/G]CGATGACTGGGGAGGGGAAAGGTCACAAAGACCTGGACCACTTGAACCCAGATGTGGTACCCGGGCATCAGAGTGCACCCTGGATTGTGTTCCTTTGTCC
13	63922955	SCAFFOLD106976_19933	0.39	0.38	AGTTTCATACATGCTGCACAGAGTGCCTATGTGCCCACTCATCGAGTGTTTAATGGGGTTCCTTGGGTAGAAACATCACACATATGCTGCTGTTATCTTC[A/T]CAGATGGGATACAGGGGAAAAAGGAAGCCTACACATGGATTCCTCCAGATCCTGACTGTGTCTCACCTTTCTGATCCAGCTGCGTATTCTTATTATATTT
13	64247824	SCAFFOLD220481_8088	0.19	0.30	AATTTCTGGTCCAGTGTTTTTCCAGTCCTAGTGGTGTTTGGGAATCTTACAGTGTTTTTTCAACTTTCCTTTTATTCTATGATTTTAAACAGCTTGCTAC[A/G]TTTTTGAATATAACTTTTTAGTCACGGGATTCAAGTTTGCCCCATGGGACAACAGCTCCAAGTTTATAGTGGAATTCAGTAGATAATTTGGTAAATTCAT
13	65451082	SCAFFOLD300112_30811	0.04	0.07	GTTCCAGCAGCCTAAGTGGTATGACTGAGCTGAATGGTCTGCAGGGCAGGCAGACAAGGCAAGCGTTGGTTCTAGGGATGTGTTGACCTAGCCGAAGGCC[A/G]GAATCTTTTCCCTTTTTAACAGTCTTTTGAGTATCTGCCAGATTACACTTTTTATAAACAAGCGGTCTAAGAAGACATTGATAAACACTTCAGAATTGTG
13	65960924	SCAFFOLD151177_7050	0.40	0.35	TTCAGCAACAGCCAACAGCAAGGGACTGAAACAGGCTGGGCTCAGACTCATCGTTCACACTCCCCATCACCAGCGGGGCAACATCAACTCTAGGCAGGTA[A/C]AACAGAAGCCTTCAGTCTGAAAAATAAGGCTTCAGATTTAAAAAGTGCTATGTCCTTTTTGAAGAATCTTTCAGTCTAATCCTTGCTACAAACCTGGAGG
13	67271559	SCAFFOLD125540_25494	0.50	0.38	ACACACTATGAGAAGGAACAACAACAAAAAAATTTCCCTTCGATGGAGTGGTCCTATTTCCAAATGACATAACAACAGGGTACAAACAGACCAAAACATA[C/G]TCTGTATTCATCCAAACGAACTTCAGGAAAGACTACACACTCACCTTAAGACTGCTGTTTCACATATCCAGGAAACTCAACGGAATAAAGACCATAGCT
13	69483711	BES10_Contig781_1552	0.04	0.05	GTTACACAACTGGTAAAGTACAAATATAGGGTTTGAACTCCAAACACTAGGTTCTTTATTATATATTCTTAATACAAAGTATTGAATTATCCCTTAATCA[A/G]TCTACGCCAAGAATCATCCCACATACAAACACACAAACTAAATTGCTAAACCCACAGACTAAGAGCAAGTAATGAAATGTTTATATAAGTCAAAAGAAAT
13	69484258	BES10_Contig781_1005	0.25	0.25	TAATGTTTCCTCACTGAAGGCTATTAAGGCTGAGTATTTTTAATATTTATTCCTAAGTACCATCATCTATCGAGTGATAATCTTTGTTCAAACTTCTCTT[A/G]CCTATAGTGTCTCAAACTGTGAGATCAGCTTCTCATAAGGCGGTAATTGTACCTGGTGCTTAAAATGTGAGTCATTCTATACAAAGCCCATTTCAAGTTC
13	70760034	BES10_Contig446_1064	0.47	0.45	GACTCTCGTCATGGGTAAGGGAAAGGACATCTTGAGTTTTAGAGATGAAGCTTTCACAGGTCATGTCAGATAAGCTGCCCCTGGGCGGACCAAGGTGATC[A/G]TCTCCAGCTTCGATTTTCATGAGGTTCTCGGGTTCGTTTTCAAGGGACTCGGAAGTCCGGCTCATGTGCGTATAGGCCAGAGACTCATACAGTTTGGAGT
13	70760179	BES10_Contig446_919	0.48	0.44	GGACTCGGAAGTCCGGCTCATGTGCGTATAGGCCAGAGACTCATACAGTTTGGAGTTGGTCTGGTAGAGGCAACAGAGACTCTCTGGGGCCTGGGGGTCG[A/G]GTCTTTTGTGCTGGGCGTAGTTCCTGTAGGCATCCAGGAAGGACAGCTGGGGGTCGGTCATGACGTAGTAGTCGGTGCGGTTCAGGCCGTGCTTGGCGGT
13	71776447	SCAFFOLD31321_2444	0.40	0.30	TATTTTTTATGTTAACAGATTTTTCCAAATAATAAAACAAAAGCAAAACACTCTATTTTCTTAATTTTAATTTTCAAGTTTAAATTTTGATCAGACTCCT[A/G]TATGATTTGCTGGTGCCCCTTTAATCAGAAATTCACACTCTGTAGCCAGCCTGCTTGTGCTGAATCAGGTATCCCCATATAATTACCCAGGAAAAAAACA

13	71833824	BES7_Contig496_2395	0.32	0.30	TGTTCAGAACGTGATGGTGTTGAAGATGGGGCCGTGTGACCTGACCTTTGTCTATACTAGCAGGAGAAGAGATAGAACTTTCTAGAGCATTTGATGTGTT[C/G]CAAGTGGGGAAGAATGGTGATTCCTACATTTATGGCTTAAAGAATTCAGAAGAAACAAAAGCTTTTTTCCTGGTTACCAGCTTGACTCAATTTATACCCA
13	71834078	BES7_Contig496_2141	0.30	0.31	GAATTATGCAAATTCTTGCTTTGGGCTGGGTTTTCCATTTCCTGCCTATAGGGCCTCCTGCCCATCAAGGACCAGATCCCATTCTCCCTACTGACCTTCT[A/G]TGGACCAGAGCCATCTGCACGCCCGGGGTGAGTGAGCCCTGAGAGGCCACTGTGGCCAAGCCACAGTGTGTCTGAACACTCACATGAAGCCAGCACCTCC
13	71834154	BES7_Contig496_2065	0.31	0.30	TCCCATTCTCCCTACTGACCTTCTGTGGACCAGAGCCATCTGCACGCCCGGGGTGAGTGAGCCCTGAGAGGCCACTGTGGCCAAGCCACAGTGTGTCTGA[A/G]CACTCACATGAAGCCAGCACCTCCTGGCACCTCTGAGAGCTTGCTCTGAGCTCATCCATACAGCAGCCCCTGTCTCCATCAACACGTGTCTGCCCCAGTC
13	72073425	SCAFFOLD230463_13155	0.19	0.15	TTCTCATATGCTTCAGTAAATGCTGTGCAAATAGAAAGGTGATCAGGAGGGAGGCAGAGGGTGATAGTGTCAGAAAGCAGTTTATGTTCACCTTAAACAT[A/G]CCTAAGAGCTCACTTCAATCTTAGTAGCATTCAGTTTTTCTCAAAATATTTGATTTCTGTCTGGATCAGACAATACACAAGCACAAACTTGCTCTCCAGC
13	72075811	SCAFFOLD230463_15541	0.01	0.03	CGACTGATCTGATCTGATCTGATCTGATGATTAGGGGACTTTCAGAAGTGAAGTCTCAACTTATAGTGTAGGTGTCTGAGCCATGCCGAAAGCAGCCATG[A/G]TTTCTCCTTATTAGGAGCTATGGGGGAAGAACTGGCTTGGCAAAGGGACACGGATGTGGTTCTTGATTCTTGTTACCTGTCGTCTGGGCAAATGAGAGGG
13	72485675	SCAFFOLD300567_13883	0.30	0.48	TCAAGGAGCTTATAGTCTAGAGAGCTAGATCAGATGTCCAGACTGGCCAACATAGACCACACAAGTAGGTGTGAGATAGCCTGGGGATAAACCAGTGGCA[A/G]TTCTTGGTGACTGTTTCAGTGGGGCAGCAAGTCTTCGAGGTGAGCTGAGGCTTCCTTGTGGGAAAGAAGCAGTTTTCAAGAGGAACTGGAAAGAAGAGAT
13	72725948	SCAFFOLD110184_5068	0.05	0.21	GTCCTCAAAAGAAGCGATCAGGGCACAGAGGGAAGATCACGTGAGGACACAGGGAGAAGACGGGTATTTACAAGCCAAAGACAGAAGCATCCGGAGATGG[A/C]AATGCTGCTAATGCCTTGATCTTGAACTTCCAGCCTCCAGAATTGGGAGAAAATATGTTTCTGTGGTTTAAGCCTCCCAGGCTGTCACAGCCCTGGCTAA
13	72728214	SCAFFOLD110184_2802	0.21	0.48	AGTATCTGTGACCTTTTGTGTCTGGCTCCTTTCCCTTTAGCATCATGTTTTTGAGGTCCATCCATGTTGGAGCAGGGATCGGTACTTCATTCCTTTATAT[A/G]GCTGAATAATACCCCACTGTGCTTAAATTTTGAATAAGATGCTTATGCTTCTCATTTGCACTTTGCACTGGGCTCCGTAATCATGAAGCTGGTTTGAGCT
13	74157196	SCAFFOLD292027_918	0.30	0.47	TGATGATGTAGACCAAAAGAATTTAAACGTCCACTCTATCATTTCTTGCTTTAGCTTTTATTTTTTTTCAAGCTAAACAGAGTTAAACTATGGTACCTGA[A/G]CCCCAGGAAAGGATCTAAAATAATCTACCAGCTCATTTGTAACCTATTGGCACATTCTTTAATTTTTTAAAACACAATTATATCAAATATTACACTTTTA
13	75020098	SCAFFOLD321788_13725	0.13	0.07	GACTTCGTCATGATCGACTTTAAACCAGCTTTTTCTAAAGACGATATCCTTCCGATGGACTTGGGGACCTTTATCGTGAATTTCAGAACCCGCCTCAGCT[C/G]AGCAGCCTCTCCCTCAACATCGGGGCCCAGTCCATGGCCGAGGACCTGGTACGGAAGCCCCTTGGCCGCCACCTCGCCCTCTGGCCTCCTGGCCTCCTTC
13	75409753	SCAFFOLD221402_10840	0.39	0.26	AGCAAAACAATGGTTCAACTGGGGACTTTGCAGCTGTGCCAAGGTCTTTGTTGACCGCGGGCTGTGCAGACAGGAGTTTCTTCTGAGATTTGCCGGTGGC[A/G]TCTCCTCTAGGACCAGTGGTTTTGGGGTCAGTGTGTATATGCTGTGTGTTGAGTTCACTGATGAACAGTCTATCCAGTTTCTCATTGTACCGCAGGAGCA

13	75796260	BES10_Contig722_843	0.31	0.40	TTGCAACCAGAGGTGCAGAGGAGGAAACCGGAGGCTTAATCAGCGTCCCAGAGGAAGGAAACAGGTG<br>AACTGAGACATGCAGTTTGGTTCATTGGTGGAC[A/G]TTGAACTTGATGTATTACACGGAGGGAGTCAA<br>GCTTATACAAACTTAAAGGACTTGAGCTTGTCTGAATCTTTACCCACATCTACCCCCATTTTTCAAGT
13	75892681	SCAFFOLD115125_34418	0.29	0.19	CTAAGGCACATTACACGTGCTTTAGTTCCGCAGTCTCGCCTCGCAGGTGAGAGGAAAACCGAGACTTGG<br>GTGAAAGGGACACGTCATCACCCAGACACTC[A/G]GCGACGGAGCTGTGACCAGGTCCCGAGTCCTCT<br>GGCTCTGAACCCAGGTGCTTTCTTCAGCAGGGACCGGCATCCCAGCCCAGCACTCTTCCTCTTGCAC
13	75892752	SCAFFOLD115125_34489	0.05	0.09	GTGAAGATTCTTACGCATAAAACTTTGAAATGGGCAGTCTTCATTTGGAATTCTAAGCTCTAGTTTAGAA<br>TCTAAGGCACATTACACGTGCTTTAGTTCC[A/G]CAGTCTCGCCTCGCAGGTGAGAGGAAAACCGAGAC<br>TTGGGTGAAAGGGACACGTCATCACCCAGACACTCAGCGACGGAGCTGTGACCAGGTCCCGAGTC
13	76383483	SCAFFOLD285651_2244	0.16	0.23	GGCGGGTAAGCCGTGTAGGGGGTAGCTTGCTGGATCCCTGCACTGTACTGTGTCTGGCTGTAGGCTGC<br>CATGGCCGAGGGAGGCTGACCAGGTGGGACAC[A/G]CGGGCAAGGTCTGCGTGGAAGAAAGAAGATT<br>AAATCTCCAAGCCCAACCTAGCACATCCCTCCCTCCTGGTATCACCACTACCACTATTTCCCTTAGGGT
13	76383888	SCAFFOLD285651_1839	0.43	0.41	TAGAAGTAAAGATAACTCAGAGGAAAACATCGGAAAATGGAGAGACATTTTCCTCCTGATATCTTCTGA<br>GCACCTGGATCCAGCCATACCTGATATCAGC[A/G]ATGTCCCCAAGCTTACCAGTATGTGAATCATTAAA<br>TTTCCATTTTCTCTAAGCCAGCTTGGGTTAAGTTTCTTACCCTTGCAATCAAAAGAGGCCTTGAC
13	77510210	SCAFFOLD281997_6609	0.40	0.41	ATGTATCTAAAGAGCCCTGGCTGCCAAGTGGGATGCACAGAGTGAGGGAGTAGGATGGATGCCCAAA<br>GCCCAGTTAGGAGACTTGGGCAGTGGTCCAGAT[A/G]AGAGATGGCACGGCCCAGACACAGATTTAAG<br>TCAGACCCTGGTTTACAGACTCCAGCTTTGTTCTCACAGTTCCAACCCCCTTTCCTCTACTTTTCCAAA
13	77867617	BES11_Contig356_648	0.12	0.11	GGGTAGGTACTTGGAGGCCTCACATTCCTCCAGGACTTCCTTGAAATCTGGGTCCAGTGAAAGGCAGTG<br>CAGGGGAATTTACTTTTCCATTTGGACTTAT[A/T]GGCATTTTTTTCAATTTTGGAATGTCTGGCCTAGGT<br>TAGGATAAAGTGGGTACAGGAGATGTGCCAGAATTCCCAGACCCCTAAGTAACAACCCCATGCC
13	78054017	SCAFFOLD130234_3209	0.37	0.40	TAAGTCTTTTCATGTGGACACTTGACAGCTTTAGAAGCATTTCCGCCCCATCCCAGGTCTGCAGGGCAGC<br>CAGTGTTATTTTGATTGTCCCCATGAAATG[A/G]GGCTCATTGAGGCGGCATGTCTTGCCCTACGCCACT<br>CAGCTGGTGGAACTGAGATCCAACGGAGGTTTGCCGTGTTTAATCCCAGTCTCCTTGCCAACAC
13	80921358	BES11_Contig445_620	0.42	0.09	AGTAAGCAAAGATAGATAAGCTAAGATTTGAGGAAATACAGTTATCTCTTGTTTCCCGCCCCCACCCCCC<br>CAAGGGGTGTCTGAGGTATTCTAACATAAA[A/T]AATACGTGTGCTTAATTAGAAAAAACAACAAGTCA<br>TTTCTACCCAAATTTCAGTTTTAAATTCTTATTTACAAACCTTAATTTTATCTTATTTCTTCCTG
13	80921358	BES6_Contig364_1079	0.07	0.09	AGTAAGCAAAGATAGATAAGCTAAGATTTGAGGAAATACAGTTATCTCTTGTTTCCCGCCCCCACCCCCC<br>CAAGGGGTGTCTGAGGTATTCTAACATAAA[A/T]AATACGTGTGCTTAATTAGAAAAAACAACAAGTCA<br>TTTCTACCCAAATTTCAGTTTTAAATTCTTATTTACAAACCTTAATTTTATCTTATTTCTTCCTG
13	82125719	SCAFFOLD140574_17328	0.25	0.26	CGCTAAAATAAAAATCATCATTTGTCTTTGTCATTAATCTCTTCTCAAATTCTCTTATATCTAAAAAGTGAT<br>TGACCTTCTCTGTGAGAGAAGGAGGGAG[A/G]AAAAAGTTTTATACATAGCTGAAAATCTGAAAATTGT<br>GATCACGGTAACTTAAAAAAAAGAAGTAGAAAAAAAAATCCCTTTTAGAGACTTAATCTGGAG

13	82646889	SCAFFOLD300165_20480	0.46	0.42	NNNNNNNNNNNNNNNNNNNNNNNNNNNNNTTTCATCAGTACAGACCATGAGAGCCCAGTCCATGATGGACTCCCTGAGACGCTGCTGCTGGCCGTGCCAT[A/G]TGGTTCAAGAGCTCAAGCTCTGTATAATGTGGACCGGGATCCAAACCCCAGCAGCCTTGCTGACCAGCTGCGTCCTTAGGTACCTTACCTGACTTCTCTG
13	83662423	SCAFFOLD45971_15872	0.36	0.33	TCATTATCAAATCTGGGCACTGCCAGAGATATCTAAATTTAGCAGGACAAACAACAGATGGAGAGAAACATCACTCCCCCTCCCCCCACGCCAGGCTCTC[A/G]GGGTGTGAGCCATGATCCCTTAAGGGGGAAATTAAAGGTCATAAACAAATTGGTAGCGGAAAACAAGCTTGTGCTAAAGATTCAGAGGAAGGAAGAAAGG
14	4037388	LOC51059	0.38	0.40	GCGTGAAGCAGGCGCAGGCTCCACTGTGACTCTGTCCGTTCCCTCCCAGGTTACGGGAAACCAGGAGCCCAGGGCAGGCACCACCATCCTGCTGTCCCAC[A/G]TGACCTCTGCAGAGAGCTCCTCCTGGACAGCCTGAAAGCTGTCGAGGTCGTCAACCTGTCTGTGTCCTGCGCTGCCACCTGTCTCCCTTTCTCGTCTGTG
14	4145186	SCAFFOLD15359_15042	0.47	0.31	TCCAGAAAGGGATGGTTTTTCAACCGGAGATGACTGATGGGAGGGAGATGAGAGGGCAAGATGAAAGCCACATTCCTAGGCGGCTTCACTAGAGCCAAGC[A/G]AAGCTAGCGTTTGGATGGATAAATGACAAGGGGACAGGCAAGGTCATTCTAGTCAGAGACTCTGCATAATCCTTAGAGCCATGAGGGCACTTAGCATGTA
14	4225524	SCAFFOLD4277_4521	0.42	0.40	AAAACACTCCCAGAAAGGTTCTATTCAACTTCCCCTTGATTAAAAGGGGGAAATGGGAAAAAACAAAAAAACAGAAACTTCCCTGGTTGAGAGATCAGAA[A/T]GGGAAGCTGTCATGCTCTGTGAAGATTACAGAGCATAATAGGAAAAGGAAAGAGTCCTCTTTCCTGGCAAGAAAGCAGCCAATTTAAAGCTGTGGACTCT
14	4922533	SCAFFOLD160323_18366	0.11	0.28	TTCTTTTTCCTGCCTTATGCAGACATTTTTAAGGTGGACATAATTTTTGCATCCATCCCAGTGCTGCATGTTTTATTTTAACAAATATATGAGCCTCAGC[A/G]TCAGAACATTTTGTATGTAATCATTAAAACCCCAAATTGCTGTCTGGAAAGGAGGATCATTATTTTTCCATTTGCTCAGATCCTAGAACACAAAATGATC
14	5063805	SCAFFOLD76695_621	0.34	0.24	TAGCAAATTATGGTCCATAGGCCAAATCCTGCCTGCCGTATGTTTTTGTAAATAAAGTTTTATAGGGACTTAGCCATCCCCATCCATTTACATATTGTCT[A/G]TGATTGCTTTCAATTTGCTACAATGGCAGACTGAGTAGTTGCAACAGATACCATATGGTCTACAATCCTAAAATATTATTCCCTGGTCCTTTGCACAGAA
14	5527174	CC775532	0.19	0.08	CTTTTTTTTCCTTCTTACCAGGTCGTTGAGCCGAGAGTCCAGCTAAAGCCCTTGCTTGTTTGGAATCAGCATTTCCTGCCATTC[A/G]TCTTGCATCTTTCATTTCTTTCTTCCCACTCCTCAGCCCAGAATTGCCTTCCATCCCTTTTATTCTATAGGAAGCATCCCTCTGCCGTGATAACAGTTTT
14	6134333	SCAFFOLD91064_10332	0.15	0.17	CTTGAATTTCAGAGCAAAGATCTACCAAAATCCATGGCAAAGGGGCTAAAAATAGACATGGTGTTTATTTGTCTGCAGCCTCTTTGGGTGTGTTATTAGC[A/G]CTGGCACAGAATTCGAGCATCACCTGTAGTGCCAGGCTGGAAAGAAGCTGGGGCTGACATGACCACCGAGACAAGGCTTGATGACTGTGTGAGAAATCAT
14	7620648	SCAFFOLD240007_5847	0.04	0.06	TCTACTGTGGTCTTCAAAGAAATCCTTGTCTTCTGTAGAAAGAAAGTTGAAATTTCTGAAAGCCGAATCCTAAGGCTGCTGAATTATAATAAGTGGAATT[A/C]ACAGCCCTGCAAGGCATGGGGAATTGGGTGAATGGCGCCGTCAGTATCAGAATGAGGACATAGGGGAAGATCCCAGTGAATCCCAGGATTAGGGGGGATT
14	8103466	SCAFFOLD90092_6051	0.13	0.15	CTTAGCGACTGAACAGCAAGAACAACAATAACTTTGGGATGCTCAGGTTTCCCTTAAGGAGGAAGAGTAGGAAACTGGTGCAGTCAGATTCCAAAACACC[A/G]AGTAACTTACTACTGGGATCCCACTCAAGTCTCTGTGAACACCTCATTTAATTTTCAGTGTGGGCTGAATATGTACTTAAAAAAAAACTATCTACCTACT

14	8561778	BES10_Contig690_1296	0.14	0.23	TCACACAGCATCCCCATCACTACCTGCCAACAGCAGGGTAAGGCTCTGCCGTCACAGGGGTCAGTGTGACGGAGCTGCAGGCCAGCTGCAAGGTGGCATG[A/G]GGTATAGATCACAGTGGAACTAAGCTGTTTTTAAGTTCTAAACTAGACATTCTCCTCTGAATACGGTATATATAATTTTAACAATGGGATTACATCATAT
14	9254038	SCAFFOLD105570_18245	0.33	0.41	GGGGTAGGTGCACCAATGTCTCACCGAGACTACGGTGTAATCAATGGGAATAAAGAGAGAGACTTGGGGAAATCAAATGAATTAAGTAGGGAAAAGACAT[A/G]GTGATTCTACTCAAAGAAGTGTCCAGCGGAAGTTTCCAGTGAAACCTCAGTGAGAGCTCCTGGTGCCCATGATGGACTCCAGGAAGACCAAGAGTAGTGT
14	9254403	SCAFFOLD105570_17880	0.43	0.47	CAAACCAGCATTAGGCTTCAGGCACAGCTTGATCCAGGGGCTATTTGGTACACTTGGCTGATTTCCTTTCACTGTTTAGGACTTCGGTTATCAAAATCCA[A/G]TCAAAATTCCACTTTAATTTTGATCACTTTTTTTTCAGAACCCTCTTCAGAATTTTCATAATGGTAACAAATGTATTAACAATAGTAATCAAGTTAGTA
14	10022794	BES1_Contig609_897	0.50	0.00	AGACAGAGAACCCTCAAGATTTGGGTAACAGAAGTATTCGTTCCGTTGTTCTAGGGCACTTGGGGGCTGTAGAAGCTGTCTTACACCAAGAGGTGCTTGC[A/G]AAGAAGCCATACCCAGGATAACAGGTCAAGGCGAGTTAACCAAGTGTGCGGGTTAACGATGGCCGCAGAACCCAGTCCCTTGTGAGCTGTACTGAGAAGT
14	10106755	SCAFFOLD230838_1182	0.26	0.35	CGAGCAGTGGCCTTTAAAATTCAAACTGTCACCTTGTCTACAAAGCCTGGCTCAGACTGTCCAGGCAGTTCAGTTCAGCATGTCCTGAGTGTGCGAGCCC[A/G]GTCCTTATATGGCTAGATGCACAGAGAAGAGGAACTTCCCCTCAACTGGCTGTAGACCCTCCCAAGATAGCATCTTCAAATCACCTCTGGCTTGAAACTT
14	10306983	SCAFFOLD135027_2960	0.35	0.38	TGACCCGCCAAGAGCCCCAATACATGAGGCTGGGAGGTTCTCAAAACAAACAAAAATGAGCTTAGAGCTCAACTCCTAGGACTTAAATGGACTTAAATGA[A/G]CACCGAGATTAAAACGTTTCTTGGGCTCAGACTGCAGCCTAGAAGACAGATGTGAGCCTGACTGCTGTGAGCAGCAAGGGAGGAGGGTGGGAGAGAAGCA
14	10307270	SCAFFOLD135027_3247	0.35	0.37	AGAAGTATGATTAATTAGCCTCCTCAGACTTCATTGGTTCACAGAGAATATCTTTGCAACAAAGGATGCTTGGCCCAGCAAAGCCTATCGTGGAAGACAA[A/C]GGGGTCCTCAAGAGCCCACTGGGAGAAGTGATTTGAAGTGAGGGATCTCTCCTTACCCCCTCCTGTGACCCTCACATATGAGACCACCTGTCTGCTCACA
14	10335527	SCAFFOLD135027_30800	0.36	0.43	ATTGTGAAGCGGGTTCCTTACCACTAGTGCCAACTGGGAAGCCCCAACAGTGTGCTGTTAGTAAATTCCCTTGGCTGTTGTCATGCAACGAGAAATGACC[A/G]TTGATCACTGAACAGGATTAAATTATGTTTAATGCCCCCAATTTACAAAGCCAAGAGAGGCGAGACACTTTTCCTTTCAAGATAGCTAAGATATTTAATT
14	10347750	SCAFFOLD135027_42531	0.44	0.44	GCGATATGCATGATGTCATCAAGGGACCTCTGTAACTGCTAGCCGAGGGACCTGGGCTAGCCTTCATCTCACATCTGTGATGTTCGTTTCCTCACGTGTG[A/G]GATACTGAGACACGCACGCACACAAGGTATTGAGAGGATGTAGGACGTCAGCAGGTAAGAAAGGACTTCAAAAATATACCACTGTGTCATCCAGTATGGT
14	11136304	CC474638-363	0.44	0.46	ATGTAGCTCACTCTCAGCAATACATGAACTCTGAAAACATCCACCTTATAAATTCTGATCCCACGTAGACCTCTCGAAGTCCTCGCTTGCCATTATGCTA[A/G]TTCTCTTTCTGCACTTGCTCACCAAAACGTAGTACTTTAAAAAATTTTGACAACTCATAATTTTGTAGAAGGAAGGAGGGACATCTTTGCTCCATGGCTG
14	11955276	BES9_Contig292_918	0.46	0.50	GAGGAGTCCAAAAACGGACGGTCAGAGGGAAGAAGGCAACCCCGTGCACAGGCGCGAGGAGGGCCTGGGAGGAGACAGAATAACATGTCTGGGAGCCAAA[A/C]AGGTTAGGGAGGTGGGGGAGAAGCAAACAGCAAGGACACCTAACTGACCGTTTCCTCCGGCCAGATTCAGCAGCTGAGGCAAAAGAGCATGGACAGCTGC

14	13423703	SCAFFOLD100068_6509	0.17	0.17	TGCCGTCTATGGGGTTGCACAGAGTTGGACACGACTGAAGCGACTTAGCAGCAGCAGCAGCCTCTAAATATGCACAGACTTGTGAATACTTTGAACT[A/G]GGGAAATCTTAAAGAACACCTAGGTCATCATTGTTTTTGCAGAGGAAGTCACTGAGCCCCATAGGAGTGAAGAGATGTGTCAAGGTCATATGACCAGTCT
14	13465428	SCAFFOLD231170_6186	0.49	0.49	AGGATTCAAACCCAAGCCATTCTGTTTTAAAGGCCTTGCTGTGAACTATTATGTTGGAAAATTGGCATTTCAGAGAATGGATGGGCTCCAAGGCCGCATG[A/G]TTCTTGATGGAGAGGCACTGCTGTCTGTTTCTTAGTTCTGGCAGTGGCTGGTAGAGAGAACACACTCTGTGTTGGTTGAAAAGAAATGGTGTCACTTGTC
14	13999817	SCAFFOLD170354_19509	0.32	0.31	TATTGCTCCGACATGTTACCATTTAATTCTGCCAACAGATCAGTGAGGTTGGAGGTGCTGTACATTAGAGAGGAAGATGAAGGCCAAGAAAGCCGCTGAT[C/G]CAGTGGTGGTCATCCTTACTGTCCCACCACCACCACCACCACCACCATTTTTGCTTGTGGCAAGGTTTGGACAAGCATAATCACGGCCCCTGTCTGT
14	14989208	BZ889135	0.15	0.22	TTATAGATAGACACAATGTGAGTGTGCTTACTGGTTTCAACCATCATGCCCTTAAATGGCCTAACACTGGAAAACGAACAAATTCCAGGGCC[A/G]CCCATCAGCCAAAAGAAGAGAGGGCTTAAACAGGCTTTCCAGACAAAATTCCTCAGTAAAGCACTGCTTGTGGCCTTTTCTGTAAAAAGTCATCTCAGTC
14	15671699	SCAFFOLD250075_12375	0.15	0.16	AAAAATCTTCCGGGTTTTTCTCCCAGATCTGGAACTCAAAGGTTCATTTGAAGAATAGTTGATTTGCCTTCTCTCTGAAAGTTTCATCAACATAGAATAA[A/T]AATAACAGCAACAATAATATTCACGTACTGAACATCGAAAATGTGCCAGGCACTGTGTTCAGTGTCTGCAAGGACTGTCTCATTTAATTCTCCCAACAAC
14	16766493	SCAFFOLD30676_22025	0.46	0.46	TGTCCCATTTCACCAGAGAGTCATTGATCCCTGGAACTGCTGACAAATCACAACTAGAAAAAAATTGCTCCTGACAATGAGAACTGGAATTCGATTGCTC[A/G]TTTCCTGGCCCAGGCAGAAACTACCTTGATTGCTTTCCAAGGAGCCTACCCTCCAAACTGTTTTGGGGGAAAACAAGGCAAACAAAATTTAATAACATTT
14	16927841	SCAFFOLD100871_5973	0.18	0.31	CCTCGATTAACCAAGGGAAAATGCTGATGGCAACTCCCTCCTTCCAGTGCCCTCTAATTATAGATAGTTCTGGTCTGACACTGAAACCGCAGTATTTGTG[A/G]ATTCTGCTTTCAACTAGAATAGTCCTATACTTGTATATATCCTCAAACTTTCTGCTAATTTACTGAACCCTGGGAATATTCTCATTGACAATGGTAGCAA
14	17289087	SCAFFOLD134924_5249	0.28	0.47	ACAATAGCAAAATTGAATATTCCTGACAGAGATCATGTAGCCAGCACAATCTCAACAATTTACCATCTTCTCTGTATGGAAACGTTCAATGATCTCTACT[A/G]TAGGGCACTGGATTTCTTCTGGCTAGGTGAATGAGAACCAAAATCTGCAAACAAAATCAGGAGACATTTTTGGTTTACAAGGAAGAGTATTTCTATCAGA
14	19174409	SCAFFOLD155146_6019	0.50	0.49	CCCATCATCTCTCTTATTTTAACAACTGCTCCCTCGTCATTATCCAAGTGCCATTTCCTGAGAGAAGCTATTCTGACTAAATGAGGTCTGATTATTTGTT[A/G]TAAGATCCTTTAGTAATCATGTACCACTACTTAATTTTTCTTCTTTTTCCTCTGTACACCATAGCTCATGGGACCTTGGTTTCCCAACCAGGGGTCAAAC
14	20438682	SCAFFOLD155270_13353	0.36	0.28	TCCAACACTCAAGGTTTCTGTTTATATCTCACAGAGAACTGGACTGGGAGAGCACAGGGGGTGGTCGTAACTTCTAGCCCCTGGGACAGTCGGGGAAGAA[A/G]GGGTGGATTTGGACCCCAGGACCAACGTGGGATATCTCTTTCTTGGCAACACTTCTGTCTTTTTCCTACAGACCATTTGCATCAAGGGAACTTGGCCCCT
14	20438954	SCAFFOLD155270_13625	0.14	0.28	ACCACTCCCCACATGGCGATCCATTTGTGATAGGGATTAGAATCAGCATAGGTGTTTGACCCAATTTCAGTCAATAAACTCCAAGCAAGATTTCACATTA[A/G]AAGTTCCCTCTGCTTTGAGGAATAATCTCCCATAAAAGGCTGTCTCTATTCCTCTGCATGTATCCAGATGCATGCATCAATCCAGGACTTGTCACAGCCT

14	20439123	SCAFFOLD155270_13794	0.15	0.28	CAAGCAATAGAAATCCAAAATGACTTAGGACATAAAAATGATATATTGGCTTGTGAACCTGATTAACTCTTAAGTGTAGGACTAAGAAGAGGCTGGATTT[A/T]CAATGTCCCCGCCCCATTTCTCATGCCATGCCCTCTCTCTGCCCGGCTCCGAAAGTAGTTTTAGTCTCAGGCTGTGACAAGTCCTGGATTGATGCATGCA
14	20439160	SCAFFOLD155270_13831	0.14	0.28	TAAAACTACTTTCGGAGCCGGGCAGAGAGAGGGCATGGCATGAGAAATGGGGCGGGGACATTGAAAATCCAGCCTCTTCTTAGTCCTACACTTAAGAGTT[A/C]ATCAGGTTCACAAGCCAATATATCATTTTTATGTCCTAAGTCATTTTGGATTTCTATTGCTTGTAGCTTAACACAACCATCTGATAAATTGACTGGTGCA
14	21007796	SCAFFOLD15358_17120	0.07	0.16	CCAGTAGACGGACTGTATACTCTGTTCTTTTTCTCTATTTCTTTATGTTTGCACCAGAAATACCTGTCTCAGGTACTAAAGGTTTATGATAAACTCTGAC[A/G]CCCAGGTCTGTAAGTCTTTCAACTTCCTCACTGTTCTTCTTCAAGACCATTTTTGCTGTTCTTGGTTCTTTTCATTTCCACCCAAATTTTAGAGTTAACT
14	21553720	SCAFFOLD145694_4460	0.47	0.46	ACTGCCAGACAAAGTACTGCAAAATGGGGAGCAGGAAACAACAGGAAATTTAGACTCCGTTGGGAGACTAGAAGTGTGAAGTTGGGATGTTGGCAGGACC[A/G]GGCTCCCTCTGAAATTCTGGGAGGCTCCCTCCTTGTCTCTTAGTTTTTGGAGGTGGCTGGCCATTCTAGGTATTCCTTCCTATGAAGACTCCAGTCTCTG
14	22563482	SCAFFOLD220684_5173	0.35	0.34	AGTAAAGGACGCGAAGGAGAAAGAAAGACTAAGGGTCAGAGAAGGAGGTACAGAGCTCACGGAAAAGTTATCAGAGTGACCCACATATAGGACATGGTGG[A/G]GGGGTGGGGGCTGGAGATGACGCTGCTGAAGAGTCTAGGACCAGAATGAAATGTGTGGAGAGAAAGAAACAACAATTTTAAACCCAACCATTGAATCCCC
14	25882113	SCAFFOLD25283_5604	0.48	0.39	AGGCTGGGGGCCTGCTTTGTGATTTTCCCCCTTGGGCTTGACACATGTTACACACAGCTTCTGCTCCCCCTGCCAGTACCTCCAATACCTGCTGCAGGGA[C/G]CCTGATGACACCCAGTCACATTCAGAGCTGGTAGGTAGCCTTTTGGTTCACTTAGCATTGGGGCTGGAGCAGTATTTCCAACCTTAGAATCTGCTGCTAC
14	25882171	SCAFFOLD25283_5546	0.50	0.40	TTCTGCTCCCCCTGCCAGTACCTCCAATACCTGCTGCAGGGACCCTGATGACACCCAGTCACATTCAGAGCTGGTAGGTAGCCTTTTGGTTCACTTAGCA[A/T]TGGGGCTGGAGCAGTATTTCCAACCTTAGAATCTGCTGCTACTACAACCCACAGAGGCCAGACAGGCATTGTGTCTGCTTTGTGAAAGGAAAGGCAAGAT
14	28084604	BES3_Contig403_1443	0.17	0.22	ACGGCTTGTTTCTTGATATCTCAAAAGTTATTGAAGATACAACAAGGAAATTAATTTTGTCTATCTGGGAAAGAAGGATATGTAGCCTTGTTATCAGTAC[A/G]TATTTATAAGAGCCTAAGCTACATCAGACCACATCATGTATTTTGACATTACTGAATGAATACATGAAAGTATCTAGTAATAACAATCTATTTACATCTC
14	28248005	SCAFFOLD174207_1629	0.31	0.38	CATGTCACATACCTTAAATAATTTCTGGTTCTCGTGTGAGCAGATTATGGGTTCAATTGTACAATCTTTCTCCTGCAGTTGTGAATAATTCTTGGCAGCT[A/G]GGAAAAGGACATCTAACAAAAATAAAATGAAGACAGAGACCTTGAAAAAAATCACATTTAGCCTAATAAACATGAAATATTGAAAACAGGGGGAGATATA
14	29770338	SCAFFOLD65161_8134	0.44	0.49	AACATCTTTGCTTTATTCGAACCACAGCCTTCCCTCCCATAAGCCCACTCAACTTCCTCCATTGCTAGACTCCTGGGAAATCATGTCACACTTTAAATCC[A/G]GCAAAAGACAGTTTTATTTAAAGAAATCTTATACTTCCTTAACATGTTTTCTGAATCTAAAAGTAATACATTTCATACTCAAAAACTTTGCAAAAGTATA
14	30728923	BES7_Contig58_410	0.03	0.09	ACTGGCCCACTCGCATTCTTCTCTTCTTTCTGAGGATGGTTGGTGGAGTGCTAAACTTAGGCACACTTGGGGAGGTCAAAGGAACAGCCTCGCCGTCTGC[A/C]CCATTACAACCGTTTCTTTTCCCTTCGAGTACAAGACTGACGTTAAACTGACATTCCATGGCACCTTCGTTGTGCTGAATTCGCATTAGTTTAACTGGGG

14	32977353	SCAFFOLD110929_231	0.19	0.30	GAAAAGGTCTCTATTTTGACAGTATTAATATTGACATTCTGGTTATGAAGCTCTACTATACTTACGCAAGACCACGACTTTTCAGGGAAACTGGGAAAAG[A/G]GCATACTAGCTCTTTCTGAATTATTTCTTACAACTGCATGAGAATCTACAACAATATCCATAAAAATTTCAATTAAAAATATACAATGAGTTTTAAACAC
14	32977505	SCAFFOLD110929_383	0.20	0.28	AAAAATTCCAGTTGCAAAAGATTTCATACTGTATGATTCCATTTATATAGCTTCCTTGAAATGGCAAAATTAGAGAAATAGAGAACAGATTAGAATGAAG[A/G]AAGTGTGTTGTGTGGCTATACAAGGGTGACAGGAATGGTCCTTGTGGTGATGAAAAGGTCTCTATTTTGACAGTATTAATATTGACATTCTGGTTATGAA
14	34168225	SCAFFOLD110089_13496	0.18	0.28	TAAGATCAAATCCACATTTTCACTGTTGCAAAATAGTCTTCTGAGAATTCAGTGCATTGAGCAAAACACCTGTGTAGCAGGACTTCAGTGCTAAACATGT[A/G]AGAGATAATGCTTTGAATCTAGAGACTTAAGGTTTGAATTTGCATCTTTTCCCTGTTAACATGTAATAATTTAAACAGAGAAAACTTGCTCTCATTGAGC
14	34836082	SCAFFOLD270113_28063	0.03	0.04	TGGAGAAGACATCAGTCTTTTGTAAGTCTGAGATTTTTCAATTTAACATGAAATCTTTGGCTTCTTGGGACTCTTCTCCAGGGGACTGTAAAGGGCACTG[A/G]TGTCACTATGTAATTTCTTCAACAAGAGCTTGTCTTGGAGCATCGCAGTGGCTGGGAATGAAGAAACTTCTGTTTCAGTTCCAACCTCACCAGCAGCTTC
14	35705091	BES9_Contig495_375	0.07	0.08	TTATGAATGCTCATTTAGTTTATAAAAGATTATAATCTGATTACTGTTTTCCTGTCAAAGCTCATGTAACACCAGGTAACATGTAAAAGCTTTACTCCTG[A/C]AGTTTCTCAGCATTCAAAAGTGTTTCACTGTTACCTGTACCTTTCAGTGATATAGCAATTGTTTTATAAACTCCGGGATTAAAACGGTGCAAGGTAGGCC
14	38035979	SCAFFOLD388152_519	0.48	0.48	TAGGTGTTTCCCAATGAGTCGGCACTTCGTTGATTTTTTTTTACATAAATAAAAAATGACACTTCTCTGGCCTTCTCTTTGCACTTTTAGTCAGTCATTG[A/G]CTGGATCTGCATGCATGCTTCCAAAATAATTGCAAATACTGCAGACTTAATAACAAGTCAACTATCTACTGTGCTATTTGTGAAAAAAAAATTTTAATAA
14	42317180	BES7_Contig479_2175	0.25	0.36	TCAACAGTATACATTTGGTATTTTGACATCCATTAGTAACAGCAGTTGAAAAATCAGTTTCTATAAAACTGCAGAGCTAAACTGCCATTTAAACAAAAAG[A/G]AGTGAGTGAAACTGCTTTTAGGGTAATACTGGTGACTACTGGTGACTCCAAAGGAAAAAAAAAAAAACAAAACTGAGCTCATTAATGTTCAAATGAGGAT
14	43507910	SCAFFOLD20653_10639	0.48	0.47	CTACGGTATTCATAGATGACATGTCGATTCATTTCAGAGTAGGTAAAGAGGAAGAGAGAGAACCCCACGAAACATGCGGATATAGTGATGTTTAACTAAA[A/G]TGAACTCAGTTCACAAACTAGCATACAACCTTGAAATTCATCTCTTTTTTTAGATCTCTGAAGCATCCTATGAATTAAGTTTGCTTCTTGGGCACAACTG
14	43984153	SCAFFOLD51887_11536	0.31	0.43	GACAAATGTCGATGCTTCCCACCTTGTGTTCCCACTGCCCGATACATACAACCCCACTGGTACATTTTTCTTCACATGCTGCAATCATCCCTCTGTCTGT[C/G]TGTCCATCAGGCAGTCGGTCCTGAAGATGGGATGTAGCTTTACATTCCCATTTCACCCGTGTCGAGCACGTTGCCTGGTGAGCACAGGTGCGAAGATTCA
14	45060918	SCAFFOLD153924_776	0.24	0.13	TTTTTCTTTTAATGTGATTCAAATATATTTTTCTAGCAAAAATATAATTACTGAACAGTGACCCCATAGTTGATGATGAAAAGCATATTGATGTTGGGGG[A/C]AAAAATAACCACCTTGTGTACTCTCTCCACGGTCAGTTGAAATCATGCACAATTCTGTTTGAGCTAATAGCTCCGAAATATGACCCATTATATCTTTTGA
14	47656527	SCAFFOLD280227_18587	0.48	0.42	CTCTCAAATATACTTCTGCTTTTTTAGCTTGGTGACTGAGACACCCATTACAATTATAATGAAAAATTATATGAAAGCACATTGAAAAATAAAAAGCACT[A/G]GAAAGTACACAGTATTAAAATTGATGGCATTGTTATTATTGGGAATGTTTTTAGAAAGAAGCATAAATTAGAAAGAATGTATAGGGAGGCATTAGTAAGC

14	49407927	SCAFFOLD106433_368	0.46	0.49	AGGGAGGCCTGGCATGCTGCAGTCCTTGGCGTCTCAAAGAGTTGGACAGGACTAAGCAACTGAACTGAACTGAACTGACTGAAGCAGTTCATCATAATAG[A/G]GTTTGACCTTTTAGAAATAGGATGATCTGTACACCATAACAAATGTCAAACCCTGATTCGTTACAAAAGGTTTGATAATTGAAACATGGCAATATTAAGT
14	49652936	SCAFFOLD115821_7580	0.28	0.15	ACAGTTTTAAATACTGTGTGATGTTGGACATGTCCTTGAATTCTGTGAAACTCAGATGCTTTATTAAAAGATAATGCAATTGTTTCCAAGGGGCTTTTGT[A/G]GAAAGTAAATAGATATTATAATCTACATAAAGGGGCTTCTCTGGGGCCCAGTGGTAAAGAATCCATCTGCCAGTGCAAGAGAAACAGGTTCAAAGCCTGG
14	49653110	SCAFFOLD115821_7754	0.28	0.15	CCAACATCACACAGTATTTAAAACTGTAAGTTTTGTCATTCCCAGAGTCAATCTGATACTACCAGCAGTGTAAGAATTTTGCTTTAAGATTGGTACCTTT[A/G]AAAAATGTTAAAAGTCTTTATTGTCTTTAGAGCAAGTCTCCGTACGTATTCTTTCTTTAGTAGTTGTACTGGCAGTAACCAGACTGTCTCCATATTGAGT
14	49653353	SCAFFOLD115821_7997	0.30	0.16	TGATTATAACATTTATATGGAGCCTACATACCAGTACTCTTCTAAACACTTTTACATACTTTATCTTATTAAACCTTCCCAACAATCCAACAATATACGA[A/G]TAAGTATCCTCCTCATTTTACAGCTGAGGAAATGGAAGCAGTAACAGTTTAACTTTCTCAAGATCACATAGTCAGAGATGAAACTGGAGTTTGAATACTG
14	49770819	CC517527-184	0.47	0.35	ACTATATCTATATTTTAGGAAAGGCATTCATCCTTTTTTAAGTGGACTGAATTTAATAATGGTAAGAGAGAATTACAAATCAGTTTCCGTTATAGGTTG[C/G]GGAACAAAGTTGTTGAGCAAAAGACACTAAGTACGCTGTCTTCACTTCATTTCTCAAGCTTAGTTACCAAAGGCAAAGTCTTTATAATGATGTTGCACAT
14	50576162	BES2_Contig249_835	0.45	0.45	AACAAAAATATAGATATATGACAGATAAGATATATAGATATTTAAATTAAGCACTTATTATAAATCAGACCATGTTCTAGGCACTTCAAATATGTAAGTG[A/G]ATAAAGCAAGGGTCCTATATTTTTACGGCTTAATTCTAACATGTTAAAAATCAAAGGAATTCAATAGTTCCCTTATCTTTAAAAGCTTCCTGAGTTAGCA
14	50633049	BES7_Contig67_926	0.26	0.23	ATTTTTAAAGTATCACACAAAAATTGGTGCACTATGTACAAGAGCTGAATGAATTTAGCAGACTTCCCTGGGTTAAACAGTTTTGATGGAATTGGAAAAT[A/G]ACAATATAACTTTTCTTTTACATAAGAAGAGAAGTAATTTAAATAAATTTTGATGAATCTAAGGATTATTGAGATCTTTTCTGTATAAACAACATCAAGG
14	51592022	CC493754-405	0.26	0.25	CTTCCAATGAACATTCAGGGTTGATTTCCTTTGAGGAATTGTTAGGGGAAGCACACTGATTGAAACCACTCACCCTGGTCAGATACTATAGCAACCATTC[A/G]CATGAATTTTTTTGACAGGAGGTCCTGGTAAGGAACAGGAACTAATAAGCCTCCACCAAATGGAAGAGTTCGGGAAAGGTCGAAAGGATACACCACATGT
14	51592039	CC493754-388	0.25	0.25	TCGACCTTTCCCGAACTCTTCCATTTGGTGGAGGCTTATTAGTTCCTGTTCCTTACCAGGACCTCCTGTCAAAAAAATTCATGTGAATGGTTGCTATAGT[A/G]TCTGACCAGGGTGAGTGGTTTCAATCAGTGTGCTTCCCCTAACAATTCCTCAAAGGAAATCAACCCTGAATGTTCATTGGAAGGACTGATGCTAAACCTA
14	56976615	SCAFFOLD115297_3807	0.44	0.41	AAGTATATTTTATGGTAGTTTTACCTTTTACAATATAATTGATATGTCAAAAATGGGTTAGCATTGATTTGATGCATAGCTTTTAATATGTCTTTGCAGT[A/G]TCAATAAATCTGTAATATATGCACTATCATTGTTGTCATTTGGCCAGTGAAATGAGGTCTCTGGACTCCTGAGAGATTTGAAATGGCTAGATTATGTAGA
14	58934123	SCAFFOLD135991_1371	0.44	0.09	AAGGTTCCTCCATCCATGGGATAGCTCTGGCTTATTAGTTACATTCTATCTATCTATCTATCTATCATCATCATCTGTATTCCTCTCTCCCTCCTTTTTC[A/G]TTCTCTCTATTGTGTTTTTTTTTTTCTATTGTGTGTTTTTGTCTACTTGTTTTAGCAGTTTCTGAGATGACTGCTTACGTTTTCAGTTATAATTGCTGAT

14	59954628	BES9_Contig344_1026	0.13	0.15	GCCTGGGTTAGACGGTAAGAAGTGCTGCGGCCACAGGCAGGTCACCGAGTGGCAGGACTTGTTTCTTATCACATAAGAGAGAGAAGAAAGCTTTTATTCA[A/G]TATTTTTTGAAGGTTTCAGGTGAGGTCACCCCAACCACCGGTAGCACAGATTTTGAACTTGTGTGCACAATTTGTTGTTTTACTTTCACCATTTATACTT
14	60576871	SCAFFOLD60293_15497	0.26	0.25	AATCCTTTTGAATTTCAGACAAGAGTTGCAGTAACTGAGTCCGGTTTATTCAGGAAGAGTGGAGCCAGAATGGGTCTGCTGTGAATAA[A/G]ATGACAGGCCTGCTTTCTTCACAGGCGACTTCTTCCCTATGACCGAATTCTTTGATGTTAATGTAATTCTCATTAATTGCTAACGCCAAAGCAACAGAGC
14	61785726	SCAFFOLD40049_15114	0.27	0.22	GTCAGAGAAAAGAGGAATCTAATTTGAGCGGATGTGGGTCTTATTTACTTTAGGAAAGGATGACTTTGCAATCTATTACTACTGGTGCTCACTCAGCCCC[A/G]CACACAGAGGTGCCCTGTGGTTTGGTTTGGACTTTTTGTTTGTTTGCTTATTTTTGCAAGGGCCTAGGGAAAAGAGTAAGGTAGAAAGAAGGAAAATGGG
14	62182796	BES7_Contig136_464	0.36	0.43	CATTTTTGAATTAAAATAACAGGTGTTCTGTATGTGCCAAATATTTTACTAGAAGTTAGCTGTAAATACCTGCTGCTCCAGACTCTTGACTGTAACTAAT[A/G]GATGAAGAACGTATAGTTCTCAGACGTAAACTCTGAGCCCTCTCCTGTCGAGCAGCATCACAGGTCTGATTGGGGTGCCAAATCTGCTTACAGTGGTAGC
14	62424259	BES3_Contig324_806	0.08	0.10	TAATAACTGAGCTAATGAAATACTTATCTGAACAAAATAAAGGCTGTTGTGATGACTAAGAAACAAACAAAACAAAAAGCCTTGAAATAAGTTTGTCACT[A/G]TTCATTGCAATTGTAATTGCTAGAGTTATTAAAAATAAAAATTAAGGGACTGATAGAAATTAGCTCTAAAGTTAATATATATTACATTGAAATAAAGTTA
14	62424685	BES3_Contig324_378	0.08	0.10	CATACTTTAAAAGGGTGAAGGGTTTCTTGGTAGAAAAGCTGATGATAAGCTTGTAGGTCAAACCAAAAGTACACACAGTTCTTCCACAACTGCAGATGGA[A/G]AAGGTACATAAATTATTACCATCATTATTAAAACTCTACAAAGCCCTAATTTCAAATACAAGTAATTGTTTAGCTCTTTGCTGTTACTATAAATCAGGTT
14	65438173	SCAFFOLD230109_5390	0.03	0.02	GGGCCTTGTTCCCAGGGAGTAGAATGTGCAACACAGCAACACTATGGGAGCGGATGACTGCAGACGGTCATTTCCAAGCGTGGATATTCTCTGATTTTAT[A/G]TTATCGGAGTGGAATGAGGTGTTGAATAGACAGTGACTCAGAAGCATATTGTTAAGCACAGAGGCACAGTAGCTATGATGCTAGCATAGGAAAGTGGAAG
14	66958822	SCAFFOLD81045_4911	0.35	0.35	AAATGGAATAAAACAGGTACAGAAATTAAACTTGTTAAATTTTTTGTAATCGTTGTAATAGGAAGTGACATCTCATAAACTTGTCTAGGAAGAAAATCCT[A/G]ACCTCCTGTAATAAAGTCAATATGTGCAGAGAAATGGCATGAGGGTCAAATTAAGCGTTGATCTGCCTTCCCTGGAAGAAATACCAGGCCACCTTTGGTC
14	67867946	BES7_Contig473_1475	0.30	0.42	TGAAATTAGAACTGACATTAAAACTTAAGTGTACAACCCATTTTAAGTTCCTTGGTATTTCAGAATTGATATAAATCGGTGAACTAAATAGTATACTTAC[A/C]TTTTCAGTGTGTGAATCTTTGTGGTTCTTTGCAGAAGGCAAGCTCCAGTTGCAGCTGTGTTTGCAGGAAGTCCACAATTAATGGGTGTGATTAAATTATG
14	67868280	BES7_Contig473_1141	0.32	0.44	GGATAAACACATCTCCTACTGCCATCTAGGTCAGGTGGCGATAATGCAGGGTAATGGGACAGTGACAGTTCAATGGTGATGCTGTGAATTCTCAAATTCA[A/T]CTTTGGCAAATACTAACCACCATAACCCAGCTCCTCCCAGATCCACCTATCCTCCTGCTCCTTCCCTGCCTTTACCCGTGTGTCTGTCTCCAGTTAACAC
14	68250814	BES2_Contig190_681	0.12	0.07	AATTTGCCCAAAGTTGCACAGCTATAAATGAATGTCAGACTGGGAAACCAAGCTCAAGCAGTCTAGCCCCATCTTCTACTTTCTTTATCATGATACCATC[A/G]TGTTTATTCCTCAAAGAATCCAAAATCTGGGAAACATTACACAGGAATTTGATAATTCAGAATCCTACCGAGACTCAACTAACCCAGGTTGATTAGTATG

14	69282937	SCAFFOLD195060_35182	0.32	0.32	CAGAGACACGAGCATGAGTTTCCTGGCCGCTGCTGTGAGAAGAGCTCTATCTGCCTGCCTCTGCCTGTGTGCCTGCCCTAACAGACTCAGCCCCTGTCCC[A/G]TGTCCTCTGCTGGGTGCTTCTGGGACATGCTGCACAGTGATGGCTCGCTTTCCTAATTCCTAGGACTGGAAGGGTTTTACTGCTATGGGACATCCATCCT
14	70303682	SCAFFOLD211738_6006	0.13	0.14	TACATGTTAATTCATTAACTGATAAATTGAATAGCAAGGTATCTGGAATTCAGGGTCAAGGATGAAAAACGTGGAGCCAAGAATGATGGTCAGGGGAGGA[C/G]AGCGGCAAAGAACCGCTTTGCAATTTCATCTGGGGAGGAGGAGAGGTCTACTTTTAGTGAGGTCATGACTTCTGAGCCAGGTTAAGTACTTGTGGAAATT
14	70719065	BES2_Contig375_891	0.26	0.28	ACATAAGTTCTGTCTATAAATTATTAAGTGCTGTAGACAGTAACACTAAAGCTTGTCCAAATGGAAAGATGCAGTGAAGTTCAGCCATTTCTATGACTAC[A/C]AACTAGGGATCCCTCCTTTGTTATGGCCACTGCACACGTGTCAGGAGGATGCTGTCAGGGATTTCATATCACCCTGAACTGCAACCTTTGCATGGTACTA
14	70735425	SCAFFOLD261445_870	0.06	0.21	GTGATAAATGCCAGGCTCGGTTCACTCAGTCAGCTTGTCTCCCTAATGATATGCTGAAGTATTAAAACTAAATTTCTTCTTTGTCAACAGTCCATCATGT[A/G]TATCTAAGATGAGTGGCACTTTTCTATTCTAATTTGGTTCTGAGAAATTTTTTTAGTAAGTTGGTACATGAGGTTGGTTTGTGACATTAGCTAGAACTAC
14	71310511	SCAFFOLD60962_16617	0.37	0.45	TTAAGTCTGAGCACCACTGAGCAACTTATTAGGGAACAATCTTTTCACAGTAGGCTAGGTGGGTAGCTGAGTATGTGAATAATAAGATCAAATCAATAAG[A/C]TTATTTTGCCTCGTAACCTCCAGAGTACTTGCACAATAAGTGAATACTAAATATGTACTTAACTGTATAGTTTTTGGAAAACAGCTTTAGACTCGCCTTT
14	72543576	SCAFFOLD140142_17934	0.41	0.36	GTCTAGATGACTCATTCTTGTCAGTAGAACAGAGTTATTACGACACAAGTCAGAGGTGACGGGTGGGACCCAGCGCCTCAGCAGGGGAGTTATTGCATAC[A/G]TGGTATTTTATCAGCAAATGCTTCAGAATTTATTATTCACACTGCCTGTATTTCGTCACCAGTGATTTACTCCTACTTCTTACTTCCTGGCCTTTAGTTT
14	73373525	BES5_Contig538_1040	0.35	0.19	AAGAGATTTTTAAAAAATTCCTCTGGACATTTATATCTATGATTGCAATAATTACCAAAAAAAAAAAAACCTTACAGAAAAGTTAAGGCAATGTTCAAGG[A/C]AAAATTAATAGAGCTTTTTTCCTTTTAACACCTTTCATCAGACCGTTGCCTTCTTTTCCGCCATAGCAGCCATCGTCAGTATCAAATCCTAATGCGCTGC
14	74116485	SCAFFOLD155037_19335	0.07	0.05	GGAAAACATTAACTAAATTAAATGAGAGTGTGGAAAATTAAATTTAAAGAGTGAAACAAACTAGATAAGAATAAAGGTTATCATATAGTCCAGTCATTTT[A/T]AAACATGGCTGCTCATTAGAATCAACCTGGGGTGTTGGAGAAAAAACAATACTTGAGCATTTGCATTTTTTCAAAAAATTCCAGGATAATTCTTATATGC
14	75887560	SCAFFOLD20441_13371	0.28	0.30	TTTCGGCTTGAGCCCTGATGGTCCCCTGGTCTTAGTGCAGACCTCCTTCAACTCACATGGATGGTGATGCTCTTGGGAAAAAGGGGCATTAACCTTACTC[C/G]GGGCTGCCCCTCCTACTACAGAACTCTCTGTCTTCCCCCGCAACACTCCTCACATGTCTGTCTCCAAGAGGCTTCTGCAGCACCGGGCTGTTCGACCTTC
14	76520018	SCAFFOLD15134_29247	0.31	0.37	TCTGGAAAATGGGAAATAAGCCTGCCCTTCAGGACGCTTCAGTGCCTAAGGGTAATGAATGTCCCATAGTCAGCACTTTGTAAGCACCCACTAACTTGCA[A/G]CCAAAATGAACATTTATCTGTTGGAATTCATAGTTTATAGAATATTAGAGATACTTTTTAGGCACAAGCTATGAACTTGTTGGCTCCTGACATATTCTTT
14	79992192	SCAFFOLD105715_30138	0.19	0.23	ATAACCTACCAGGATCTCCTGTCCATGGGATTCTCCAGGCAAGAATACTGGAGTGGGTTGCCATTTCCTTCTCCAGAGTGATTGGCTGTACTCCAACACA[A/C]AAAAAATTAAGAAGTTTTTATTAATGAAATTTTCAAACATCCACAAAAGTAGAGTGAATGATATAATGAGCTCCTGGCTGCTCTTCCCTCATTTCAGTAA

14	80458251	BES8_Contig464_1373	0.43	0.35	ACTCCCTTGGAAACTCTGACTGCTCTATATAGAGATATTCAATCCTACTAAAACCATGGGAAACAAATCAATAATACCCTATTTTATCCTTTATGTCACA[A/G]CACTGCAGCATTCACATTTACAGCATTACAGAATTACACTGTAATTGAGGTGTAATTACTGACTCTTTGGAAATCGTTTGGAAACAATCTGTGGCATCCT
14	80575914	SCAFFOLD228734_390	0.31	0.36	ACTGCTTTTCCAGGATGGGGCTCAACATGCTGCATGTTTAACGTGCTTGATTTCCTCAAGCACCGCACCACAGTCCGTATGGAAACCTGGAGAAAGGCCC[A/G]GGAACACACAGGGCCAGAGGCATCCCTTGCTTCGAGGCGGTTCTCCCCAGGGACTCAGCCTTAAACTGGGGGAGAAATCCTGACAAACTTTCAGCCTATT
15	486218	SCAFFOLD180081_24350	0.44	0.34	TCTATGGCATCTTTTTCTTCCTTGTCCACAAATTCTTGTACTGCTTCACCCATTCCCCTTTCTGTTAGAAGTGAGAGCTGTACGTTCTGTAAGAGACAGA[A/G]AACAGTATTTTCTGATGCAGAAAACAGTATTTTCCCTACAAAACAATTCTATACAAGTAAGGAAAATTACAGAATCAAGGAAAAAGACAAAAAAGTTATC
15	7312211	SCAFFOLD16253_557	0.39	0.40	ATCACTCGCCTATACACAAATCAAGCACAGGTGCTGTTTTCTTGAAGAACCCATCAGGAGTCACTTTTAACTCTTCCTTTCTGAAATATTATGGCCATAT[A/C]ACTAACTTGTATCCTGACTTGCTTGAAATAGTACTATATACACCATTTATAGCATTTTGCCCAGTAGGATTATTAATACTCTCTCATTTCACTTTCAAAA
15	12274036	BES7_Contig298_1427	0.33	0.25	GGGACCAGAATAGTAGAGCAGTGATTGCAGTTAGGCTCTGAGAAGGGGTGCATTTTGATCTGGGATTTGAGGACAGTGAAAGAAATACACTGGTTGAAAA[A/G]AAAGACCCTCTTTGAGATTGAAATGGCATATGGGGAGAGATGACAAATGCAGAGAACCAGTCATGTATAAGGAAGTATTTTATCCTCTAGAAATCCAACT
15	12274433	BES7_Contig298_1030	0.33	0.25	AATATTTTGGAGGATTAGGAGCTTTCTGAGTTAAGACTGTGCTTTTAATATTTTAAGCAGACTCAAGCAAGACAGTAAACAGTTTTCTTAGCAGAATGGT[A/G]CAAAATGAGCTCTTTTACTTGTCTGATTTATCCCATGGCCATTCATAATGGGGTGGACGGGTTGTTGTTGTTTTTTTTCCATCAGGGAGCAGTCAGTTAG
15	13018807	SCAFFOLD305429_3244	0.43	0.50	TTCTTACTCCTGCTTTTCTGTCCTCTCTTGTACATTCACTTGAATTCTCTCTTCTTTTCACATTATCTCTAGACTTCAGTTTGCCAAATGTTTTAGATTC[A/G]ACAGACCTGCCTTTTTCTAGTCTCTCCCATGAAGCAAGGACTCCATTACCTGGTGGCCATCTCCCACGTGAGACCAATCTGTTATGCTGCAAAGGGATCA
15	13078403	SCAFFOLD132195_1835	0.37	0.25	AAACGTTAATTTTACTTTCAACAAACCTGCAAGCAATTCAATGGAGAAAGGAATGTCATCCTAACAAATACTGCTGGGAAAACTGGATACACATATGGGG[A/G]AAAATAAGTAAATAGTCACCTGTCTCTACCTTACACCAGACATAAAAATTAATTCTAAATGAACTACAGGTCTAAACATAAGAGCTAAATCTATAAACTT
15	13078597	SCAFFOLD132195_1641	0.39	0.28	ACATATTTTCTCAAATCCAACATTTTTTGTTCAGCATAAATATTTTGGGTTCTTATGTGTTTTGTGTAAGTAAGTACTTTGCCCTTTGTATTGCTGACTA[A/G]TACTTCATTGTAAGACTATAACATGCTTTATTTATTCATTCTCCCATTGATGAGCATTTAAAATGTTTCTAATATGAGACTCTTTTCATTCAGAAGTTTA
15	13179906	SCAFFOLD120672_10956	0.38	0.26	GACCAGGAGGCTCAAACAACAGAAATTCATTTTCTCACAGTTCTGGTGAATGTTAGTCCAAGATCAGAGTGGTCCAGTTGAGACTATCTTCCTGGTTTGC[A/G]TTTGTTTGCATTTTTGCAGAATGTCTAGCGGCAGAGAGAAAGAGAGAGAGAGAGAAAGAAACTAAGGAAGGAGAGAGAGAGGGTGTGTGTATGCTCTCCA
15	13179956	SCAFFOLD120672_10906	0.39	0.26	TGTTAGTCCAAGATCAGAGTGGTCCAGTTGAGACTATCTTCCTGGTTTGCGTTTGTTTGCATTTTTGCAGAATGTCTAGCGGCAGAGAGAAAGAGAGAGA[A/G]AGAGAAAGAAACTAAGGAAGGAGAGAGAGAGGGTGTGTGTATGCTCTCCACTGGGATTAATGTTAATTAATTGAGGATTAATTTTAATTATATGAGAGGT

15	13492936	BES8_Contig457_1759	0.15	0.08	CCAGCTGTATTTTATCTCCATAGCAGTTGTCACCATCTGATATATTATATGGTGCTCTTTTTCATTTTTCTTTTTTCTCCAATAGAATACAAATTCCATG[A/G]GACTAGGGCTTTTTGTCTATTTTGTTCATTTGTGCTTCATCAGTGCCTGAAGGAGTACATGGAAACTACATGGCTTATAGTAGGCACTCAAATATTTATT
15	13671287	SCAFFOLD52197_3147	0.40	0.49	CTGCAGGGGATCTTCCCAACCCAGGGATCGAATCCTGGTCTCCCGCATTGTAGACAGACGCTTTACCGTCTGAGCCACCAAGCCTATACATCTTCCTTTT[A/G]ATACTGATCACTAGTATCCCCCTAGATTTGAAATGTAGGATATTCATTTCTTTAGGATTTCATTTACTTTAAAGTATTTTATTTCTTTATGATTACAAAT
15	16316618	SCAFFOLD115666_13006	0.15	0.21	CTTCACATAATTCCATTTTACATACTGTCTGTGCCTCTTTCATTGCACTCATTCTGAATAGTATTACAGTAAATTCTGTACAGATTTCTTCCTGTGTCTA[A/C]TAAATTTTTAGGTTTTTGTAGTCTGGGGCTGTTTGTCTTCTTTATCTGTGAAGATGATTTGAATAAAGAAGTCATTCAGGGAGAGGAGGGAGGCTCAAGA
15	19129877	BES9_Contig405_919	0.30	0.41	TCCTTTTCATTAAATAGCAGTAGGCAATTTGTCTGTAATGCATTTAAGAGATACTTGTCTTCTTGCTTTTGGCAGCATATAAAATCATCTTGAGAAAGAC[A/G]ATGGATGGGAAAGAAGACATCTTTTCATATTTGCAGCATATTTTAGATTTTAGAAACAGTTGCTCTTTAAAAAGGCTCAAAACTGATTTTCAGCTTTTTA
15	19443927	SCAFFOLD135585_12264	0.26	0.28	CTGACTTCCATGGTCCTAATTCAAGCTAACCCTTTATAGTTGTGTCGAGGAAGAGGAGCAGAGGGTTACATCATTTTCCTTCCTTTTCTTTTAAATTTTA[A/G]AAGACATCTTGAGGCTTTTAGCTTTCCTTTTATTTTGACATTTAGGAACCAAAGTGCATAAATAATAGGAGCTGGGACTCTGGAATCCAATCTCCTTCAG
15	19444308	SCAFFOLD135585_12645	0.28	0.35	ATACAGATGTACATCTCAGCAGTTCTATTTTATTAAACTCACAACATTTCCACCCAATTTAAACTCACTCTGATAGCACACGTTGTGATGGATTTGGGTG[A/G]TGGTTTCCTTTCCAGTTGGCTCTGAAAATGTCCTATTAAGCCAGACCACAATCAGATGCCAAGTTCATAATTAGCTTTCTCCTTCCACTTGGGTCTCTTT
15	20459550	SCAFFOLD183699_249	0.37	0.49	GGTGTCTTTTGAGCCAAACAACCAATATTTAAATTTTTAAAATTTTATTTGGCCGCATCAGGTCTTAGTTGCAGCACTCAGGATCTTCACTATGTCAC[A/G]CAGGATCCTTCTTTGTAGTGCTCAGACTCTCTAGTTGTGGCACACAGGCTCTGGAGTGAGAGCTCAGTAGCTGCAGCGTGAGGGCTTAGTTGCTCTGAGT
15	20459950	SCAFFOLD183699_649	0.37	0.49	ATCCATACAATTCCTGAGTCGTTTTCAGCATCCAAAGACCGAACAGGTCAGCCTCTGCTATAGACTGACCTACCAGACCTGTGACAAGGCCCTGACCCAG[A/C]AACAGGTAGCATCAATGCAGTCCCAGCTTAGGAAAGAGATTCAACAACGACTCCATGTGACACCTCGGTAGTTTAGTAAACTCATCTCTCCATAGTGATC
15	20509763	SCAFFOLD295094_16436	0.45	0.37	GCAGTGTAGAAACATCCAAGGTTTTGGCTCCCTGGCTCCCTGCTAGCAAGCAAGAAAGGCAAACACGAAAAGCAGAGGACTCTTGTGATGCACTGGAGAC[A/G]GAGCTCAGGAGGAGACCTGAATGGCTCAGGAAGGAAATCAGCTCTGCCGTGAAACCAGATACCTGTTCAGAACTCTGAGATCCTCCAGCTGCAGCAGAAT
15	21761607	SCAFFOLD60682_8173	0.23	0.26	CCAACCCGGCTTGTTTATTGACTTCTGAGTGGCAAGCAGTGGCCCCCACCTTCCAGTAACATCTTCACCAGTCACATTTCCATGCCTTGGTCCTGATACT[C/G]CTGTTGTTTCAGACATACTCTGTTTGATATCTCTGAGCAGCCATAAGAGAGAAAAAATTGCCAAGAGTTATAATCTGCTTTTGTTCAAGTTCTGACTCAC
15	22044756	SCAFFOLD160134_19720	0.22	0.23	CAAAAGGCAGGAGGAGGAAGAAGCTTGGTTTTAAAGCAACTTAAACCATCTCATGGCACTTCTCCAATCTCTTTTCATATATCCTGCAGTAATGGGCTTC[A/G]TTTTAGTTAGCCACAGCTTTGTCACCTATTCAGGAGTAATGGCCGTAGCCTTAAAGAACATTTATAATGGATTGCTGATTGACCAGAATGTCAGAACATG

15	22587880	SCAFFOLD153002_10495	0.18	0.36	ATGTATAGAGATCCCATGTACCCTTCACCCAGTTTTCCCCAATGTTAGCATCTTGTGTAACCATAGTATAGGATCAAAACCAGGAAATGGACTGTGGTAC[A/G]ATTCACAGAGCTTATTCCGATTTCTCCAGTTTTCCTTGCACTCATTTGTGTGTGTGAATAGTTCTGTGCAATTTTATGACCTGTGTAGATTCATGTACCT
15	22598835	SCAFFOLD301689_4716	0.13	0.23	GGAAAGAAGTATATTTCTGCTGCTCAGCATCTAGAAGAGGTATTAAACACTTTCTCTAAAGCATGAACAAGTAATGCTACTCTTGTTTTAAGTAAATTCT[A/G]TGTCACATGTATTCCTTACTGTTATACAAATATGTTCGAATTGCCTAGAGTACAGATGAAATGTTTAACAGTGATTCCATTTCACTGCCAGGCCCAGAAA
15	22599044	SCAFFOLD301689_4925	0.40	0.49	CACAATTATATTCCTCAATAGCAGTAGAAAACTGAAAAGATAAGTATAGTTATTAGGTATTTTATCTGATACATCTTATTATTTCAAGTCTGTCCGCTTG[A/C]TTGTTTTTAAAATATGAAAAAAATAGCACATAGTCAAGGGATACTCATTAAACGATAAGACCAACTGGGAAAAAAAAAAAAAAAAAAACAACTTTCTCAC
15	22865920	SCAFFOLD55477_14453	0.04	0.05	GCTTGCCTGTGTGCACAGGCATTGCAGCCAAAAAAAAAGAGAGGAAGAACCGGTTAAATGATGTGAAAGTTCTGAGGACACTGCTGTCCACATCGCAGGC[A/G]TTGTCACTCAAGCTGCCTTCTCCTTGGCTGGCACAGCCTCAGAGAACGGGCACATCATGACGCGTGAGGAGGTTTCTTTTGCAAACAGTGTAAGTAAAAA
15	22919210	SCAFFOLD50003_7361	0.27	0.37	GTCTGAACATCCCAGGGCCATTTTAATATTAAAGCTGCGGCTTCCCTTGGAGGAACCTAAAATTGCCTTTTCTCTTGTCTCTTGTTTTATCTCCAGTGGA[A/G]CTGAAATATGTTGCCAGGCTTTGGATGGTGGAAAGCTAATGTCCGGTTTTTCATTAATTTGGAGAGTTTGGATTGTCTATGATTTTTTGACTTGTGGTGA
15	23647376	SCAFFOLD120241_12664	0.40	0.36	GAGGAGGTGGGGAAGGACAGCAGCAAGAGCACCCAGCCTTCCAGCTATCTTAAGGGAGGAAGGCTGTATTATAAAGTGCAGTGTTTCTGAGAGTTCGCTT[A/G]TCGTTATAACTGCCTCTTTTTTTTTTTTTTTAAGGTAATTATACTTTTTATTCTGACTGCTTTTAGGATCTTCTCACCATCTTTGGTGTTCTAAAATTCC
15	23647575	SCAFFOLD120241_12465	0.43	0.42	GAGATTAAGAAGATTGACTAAGATCTGAGACTCATTTAATTCAAGTTCTAGAAAGAGAGAAGGTATAAAATTAGGTAAAGATAATGTTTGAAGAGATAAC[A/G]GCTGAGAATTTTCCAGAACTGTTTCAAGACAGATTCAGGAATTCTTATGAATCCAAGCAATGTAAACAAAAAGGAACATGCTGTTACATACATCATAGGG
15	23647814	SCAFFOLD120241_12226	0.41	0.42	GGAAAAAAGGTAAACTGTAAAATATCCTCAGGAAACAAAAAATGATAAAAGGCAACAAAGAATATATTTGAAATAGAAACAAATAGAATTTCCAGAAATG[A/G]AAAGTACAATGATAGAGACTCAACTGATGAGTTTTGTGGCAGACATGCTAATAAAAAATAATTTGTGAGGCAGAAAATAATTTGGACAGGTATTAATGAG
15	23648038	SCAFFOLD120241_12002	0.38	0.28	AAAAACATACATTCTCTTTGCAGGAAGCTGTCCTAGGCTTAAAAAAATGTCCCCAGAAATAATTTTCCAATAGCAATGAATATCACACCATAGAAAATAT[A/C]AAGGTACACAGAGCAAAGCATCATGTGGGAGAATTGGGATAGAAGAGGGTAAAAACAAACGCACAGAAATTTTAGACTGAATTAACAGAAATACTTGAAA
15	23870249	SCAFFOLD130505_17204	0.10	0.09	ACAAAGCAGAAACTTGACAAATAATGTTTGACTTCAGGGAAAGGCATACCCTGTTTTATGTCAGACCACCAGATCTGCAGTTGAACTGGATCGGGTCTTC[A/G]TTGTGGTTTTGGTTTGATTCAATTCATCACTGACTTCAAATATTTTGAGGATGAAATTAGGACATATTCTTCAATAGTCCCTGAGCTCTAAGAACTAATA
15	25767576	SCAFFOLD126178_891	0.34	0.42	ATGGTGGGGAGACATATATCTCAATTCATTCTCACTCAGGGACCCAGGCTGACAGCATCTCCAGCATCTCAAATGTATCAGTCACAACAGCAGAAAGGAA[A/T]CACAGTGAAGTACATATCATCTCTTAAGGCCTCTGTCCAAAGACAATCTTCTACTCACATTTTGTTTTCAAAAGCAATCACACTGCAATGCCTAACTTC

15	27033891	SCAFFOLD125204_26750	0.39	0.39	GAACCATGAGTCACCCTAGGATTTCTTGGAACCTTTTCCCTTCAAAACATTTGTATGTTTTATTTGCACTGGTTTTGATGTCGTTTCATAACATTTTATC[A/G]TTTTCAATAGGGGCAATTCATTATGTGCATAATTAGAAGCCATTTGATTTTAATTTCCTTTAGCGTTTTTATAGAAATAAGCCAAAAAAAAAAAAAAATT
15	27484163	BES11_Contig371_409	0.22	0.18	TGCATTTATCTGTTGACATGTTCTATTTGCTATACCTTTAAAAAAAATAGGTTAAATAGAGAGGATTATTAAAGTCAAAGAGGAAACTGAGCTACTAGTT[A/G]TAACAACAAAGTAGCACTTAAACATGACAAAAATTTCCCATAAATCTCTAGGCTGGTTGACTAGTTCTCATTCAGCATGAGCATAAAGTGGTGCTTGTGC
15	27484342	BES11_Contig371_588	0.22	0.18	GTTGTTTCTTTTCCCATTCCTTGACTATGGTACATTTTAAAAGGCTTTGGGTAAGACTTGCCAACTTTTGTTCCAAGATTCAAGGCATTTTTGGCAAAAT[C/G]GTTACAAACCGACCAACTGGACAAGCTGGCCTGATTTGGGGGTGGCTAAAACCAAGAAACTTAAAAGATGCTCAGGAATGCATTTATCTGTTGACATGTT
15	27557203	SCAFFOLD116323_2427	0.22	0.18	ACCAGGAGAAGGTTGGAGGCACTGCCAGGCATTTGCTTCTAAAAAGCAAGATATTTGAAAGAAGAATTCTGTTGTGTGAATTTTATTGTAGTTGTTTTGG[A/G]TGATGTAAATCCTCTTGTAAGCTGAGTCTGAAACTTCTTAGAGGCCAAACTTGCTTTAACTTTTTAGATGTTGAATATAACTATTCTGATGTGTTTTAGT
15	28170828	BES8_Contig293_787	0.32	0.31	CTGGCTCTGTTTCTGCAGTTTTTGGACCAGGTAGTCCCGGATGACAGACAGGGTGGCCGTGGAGTTATGGGCCAGGGTCTGCACCACTGAGGTGGGAGGA[A/G]GCAAAACCATGAAGCATGCAGCTTTCTGGACAGAGCTGAAGAACCCCTGCCCCAGAGCCCGACCGCCAGCAGCCTGCACCCACCTAGCCACCCAGCCCAC
15	28495153	SCAFFOLD289711_762	0.46	0.10	GGGGACGGGTGAGATAGCTGGTGGGGGGGCTGGGCTTGAAACCAAACTTCTCCTTCTCCAGGAAGGAGGCAGCCAGGTTGGTTCCGTAGGAGGAGGGAGT[A/G]TAGGTGCCATAGCTTGATTTGGTGAAAGGCGCATCTGTAAAACGGGCTGATTCTGTGTAGCGCTTCAGAGTGGAGGAGAGCTGGGACATCCTTCAGGGCG
15	30111170	BES6_Contig352_1429	0.09	0.20	GGACTTGTTTGTTGGCTGAACAAGTTCATCAGTTGAATAAAAAACTACTCATTATTGAGTTTCCAGAAGTTCTGTCTGCCAATTCTCCCTTGCCTAGGGC[A/G]TTTTTAGTCTCTGTCTGACAGGTCCATAGTGAGCTTCCCTTTTTATACTAATTTACATATTTAAAGGGGTCTAACCTCAGATGAGGGCTTCCCTGGTGGT
15	31343064	SCAFFOLD176337_1310	0.35	0.28	AGTGGCACAATTAAGACCCAACCAAGCCAAATAAATAAATATTAAAAAAAAAAATCTGCTCCTAAGTTGTCCATATCAAACGTGACCTAGTTCATCTTTC[A/G]GAGATCATCATGGTCTAATTCAAAGAGCTAAATCTTGAGTCAGAAAGTTTATATTCACATCTCAAATCTGCTGCTTGTTAGCTGCTTGACCTTGTTCTTA
15	31343587	SCAFFOLD176337_1833	0.35	0.28	ACTTGGCTAACCTGAGCACATTCCCAGCCCAGGCCGCAGTCAGAGATGACGCTGAGCAGTCCTCCTTTACTTTGGGGGTTGCTATGACAGCCAGGAATTC[A/G]CAAACCAAACCAGACCTAAAGAAGAATTCTGTCCTTGGCCCCAAAAGTTAGTGGTTCCACGCGGTTCTGCTTGTGGTTCCTGCTGTCCCCTTCGACACCT
15	32194742	SCAFFOLD92619_934	0.41	0.43	CACTGGTGTGTCCTGTGCTTCCTGCTGATTGGATCTCAAGGGCTTATTTTCTCCCTAAGGCCCAAGTTATTTCCAGGACACAGCATTGTCTCGTGATGCA[A/C]TAAGTGGCACCTTAGTGGCCTGAAAGTGTGAATACACTATCTTAGAAAAAAAAAAATTTTCCCAAGAACTAGGCATCATGGAGACTCTGTTTCAACTGTGA
15	33209840	SCAFFOLD55249_11340	0.05	0.06	TTTGTTTAAAGATGCAGGAGAGCTGTATGTTTGGGGAAGCAACAAACACGGGCAACTGGCTTCCCAGGCTGCTTTCCTCCTTCTGCCCCAGAGAATAGAA[A/G]CACGTTGTTTCCAGAATGAAAAGATCGCTGCTGTCTGGAGTGGGTGGACACACCTGGTTGCTCAGACAGGTGAGAGAGTGGCAGGGAATTTGTGGTTGGA

15	35546333	SCAFFOLD150141_8843	0.16	0.14	GAAGGAGAGTTTCCCAAAGTGTGTTTCTGTAAGTCCTACCCCTAAGGATATTAATAAGACATTTAGTGTGTGTGTGTTGTGGGGAGTGGGGAAGGCAGCA[A/G]GGAGGAGGCTCTCATTGCCAAATAAGATTGATAAATGTGGAGTTAAACTGAATTTAAAGTAAAAGGATTTCTCACCTGCAGGACTTTTCAGAGCCTTTGG
15	35546715	SCAFFOLD150141_9225	0.18	0.19	CACATCATAGGGAAGGGTTAACCCTTGTGCCTGCTGTTACTAGTAACAGGATTAAGTGCTGGGTTGAGGGGAGACAGAGATGTGGATGACGAGAGACACC[A/G]CAGGACTGGCCTGGGAGTGGCAGGTGAGGCCACCTGGAGAAGTCCACATCCTCTTGTATGATCCCATCGGTTCTTGTGGGGAATTTGCCTTGGAGGCATT
15	35546817	SCAFFOLD150141_9327	0.17	0.19	AGGACTGGCCTGGGAGTGGCAGGTGAGGCCACCTGGAGAAGTCCACATCCTCTTGTATGATCCCATCGGTTCTTGTGGGGAATTTGCCTTGGAGGCATTC[A/G]AAATTCTAAGGAAGCAGCATGGATGGGTGTGGGTGGCAGCGTCTCAGCGGCACCAATGTTTACTTTGAAGAGAAAATGTCTGCTTTGTAGGCAAATGTTT
15	35549373	SCAFFOLD150141_11761	0.22	0.27	GTAGGATCTAGTTCCCTGACCAGGGATAGAATCTGGGTCCCCTGCATTGGGAGCATTAAGTCTTAACCAGTGGACCACAAGGGCTAGGCTGTCACGTTTA[C/G]AGTGCTTTTCAATTGGAATGGCTTTCTGCCCCTCTTAGGTAGGACAGGAAAACCAGAGACGGCTGGAGCTGGCCTCTTTCTCCTAGGTGGGATCAAATTCT
15	35600664	SCAFFOLD155196_10890	0.10	0.10	GGTTGACTTGGAACACCACTGCTGAGGCCACTGTCTCAGGACACTTGGGTGCTGCTGGACACTTCTATCACACTGCAGGCCCTGGACCCCCACAGCTGCA[A/G]CTGGAAATGATCCTTTTTTCCTCCTTATCTCATAATTTTCAGAGAAGCTTATAGGATGCTAACAAACTTCCAAAATAGGATCATCTGGTTAGCAATATCT
15	35603443	SCAFFOLD155196_8133	0.09	0.09	CAATACTAGATTGCTACATATTTGTGATGTCCTCCCAAACTTTCGGGACTTTTCACTTGGGGCATCAGAGCAGGAGTGTAACAGAGAGATGAAAACCCTC[A/G]GGAGTCTTCTCTTACTACACCCAAATCTACTAAGCACCCACCGTGTGTCAGATCTTTAGACACGGTGTCTGCGTCCCTAAACATCAGGTCTTCTACAGTG
15	35603588	SCAFFOLD155196_7988	0.09	0.08	AATTGCAAACATTTTGGTGAGGAGGAGGATTAGGACAGGATAAGGATCTCTAGGACTTGACCTGGTTTGTTCTGCAGCCTTTTTGCTCATTGAGCCCTTT[A/G]CTGGATTCGATAATACTGAGGAATCAGTTCCTTCAACAGCACCACACTGTAGAAGACCTGATGTTTAGGGACGCAGACACCGTGTCTAAAGATCTGACAC
15	35713980	SCAFFOLD185124_14291	0.50	0.34	GCCTCTCCCCCAAATAGAGTGTAAAACCACAACCACAACCCACAAGACCTTGAGACGTGCAAATCCAGCCATTACCCAATGGCAAGTATGGGAGTGTGCC[A/G]GGAGGCGGTCTCTTCCAGATCCTTAAGTCCAGATTCACTTTGGATCACATTGCACTGACTCACGGAGGACACAGTGGGAGGACTGACCTTCCAGTGTCAC
15	35714095	SCAFFOLD185124_14406	0.49	0.33	CCAGATCCTTAAGTCCAGATTCACTTTGGATCACATTGCACTGACTCACGGAGGACACAGTGGGAGGACTGACCTTCCAGTGTCACCAACCTCAACAGTT[A/G]CAGGACCCTGAGGATGACTTTGTACCTGAATGAGTCTGAAGTCTCAAAGGATCAAGTCACTTGGGGATTCGGTAAGGCAGAGGAGCTGTGGGATTGTCTG
15	35714113	SCAFFOLD185124_14424	0.50	0.34	TGGCTATGACATCACCATCAGACAATCCCACAGCTCCTCTGCCTTACCGAATCCCCAAGTGACTTGATCCTTTGAGACTTCAGACTCATTCAGGTACAAA[A/G]TCATCCTCAGGGTCCTGTAACTGTTGAGGTTGGTGACACTGGAAGGTCAGTCCTCCCACTGTGTCCTCCGTGAGTCAGTGCAATGTGATCCAAAGTGAAT
15	36330325	BES7_Contig444_1293	0.38	0.48	TAATTTGTCTCTGGGGTTTTTAAAAAGAAGTATGTATTTATATTCAGATTGATGGTCCAGCATCTTTAACCCTGACTAAATAGGTTTGGGACAATCTACT[A/G]AAATTCCTGTGGTGGACATATTTCCTGAAAGCCTCTTTGAGATCACCACTCCTAGAGGCTTTACATGGTAAATCATTGGCTCTATGGAAATTTCATCAAA

15	36755138	SCAFFOLD145449_8789	0.49	0.00	AAAGAGAAGAAAGAGATATTATGTATGCATGTATCTGTTTATTGTCTATGTTTGGGTACTTATAAAGAGCATTCCCCTTCTTTAAAGAAAGCAAAGCATT[A/T]ACAATTAAATGATAAAACTACATTTCTACATGGAGCTATCATCAAGACATTCTACGAATTCCGGCAGAATATAGACTATATTTGTTATCATCAATGACCG
15	36854388	SCAFFOLD15945_5566	0.22	0.23	AATTAGAGATTTGAATTTGAAATACTTTTTATGTTAAATGTTCTGCCAACTTTTTGTTTAACCATGGACTCTTCATAACATCAAAGAGTTGCATGTTTTA[C/G]AGCTTAGAGAAAAATGGACACAGTGAATCCTAGCAACTGAAGGCAAATAGACAGATACGTCAGTGCCACGCTTATGTCAGCAGTTTGTCCTATTTGCTTG
15	39760998	SCAFFOLD225150_2977	0.10	0.07	ATGAATCATCTGATTGATTGCTTCTTAACTGTCAAATTAAATATATTATCAAACTTCCTTTTAAATAGTCAAAAGTCTTTGCAGTAAAGAAATTTTTTTT[A/C]ATCAGTTCGGAAACTTTTCGGAGTTTATAAAATCAATTAAACTTTTAAAAATTTACTTAGAAGTTTTAAGAGCACGCTGTGCTTCCAAAAGCATCAGCTA
15	39761126	SCAFFOLD225150_2849	0.14	0.08	TTTGACTATTTAAAAGGAAGTTTGATAATATATTTAATTTGACAGTTAAGAAGCAATCAATCAGATGATTCATCACACACAACATGGAACTATAATACAT[A/G]TTTAAAGATGGATGGACCTTTGGACATCAGATCAAGCTCTTTACTTTATAGATGTACAAGAAATTCAGAAACCTTAAGTGACATGCCTAAGGTCCCATAC
15	39988349	SCAFFOLD155357_6188	0.20	0.22	GAAGACCAAGTCTACTTCCTTACAGAACAGAAGGCTCCCATCCTCAGGGTGGGCAGGGGCCTAGCAAGGGGACCCACTGAGGGGCTGGAGCCATGCCCTC[A/G]CTCAGTGAAATCCCAGAGTCCAGATACTGAGAGATCTTTTATACCCTGTGCTTCTCTGAGTGAGTTCCCTAGCAGCTGCAACTTTTTCTAAATCTCTAAA
15	40570729	SCAFFOLD250148_11147	0.34	0.29	AGGGAGATAAGGAAAGGGAGGGCTCAGGAGATGGAGTGCCTTTCCCAAGGTCGAGCAGCTAATAAGTGGTAGAACTGGGACTTGCACCCAGGTTTGTCTG[A/G]CTCCAAAGCCGTCGCTGTTTTCACTGCACTAGCAGAGGCAATGGCACTCCACTCCAGTACTCTTGCCTGGGCGTAGGAGCCTGGAAGGCTGCAGTCCACG
15	40571083	SCAFFOLD250148_11501	0.35	0.33	ATAACAAATAGCTCCCTTCTGAGGATGGATGCAAATTAGTAGGAGCAAAAGAAATGTTTAAAAGGAAATTCTAATACAGCATGATAAGTACAACAAAAGA[A/T]TCATGTACAATGCGCAGAGGAGACTGTGGCAGAGGCAGAGGTGGTTTCCAGAGTAGACTTCCCAGAGACGGTGATGTCTACAAAGTGTTTTGAACATTAA
15	42438315	SCAFFOLD110137_21046	0.34	0.35	AAAACCAGATAGATGCAACTTCATACCTAAAAATAAACCAAGTTTTAAACTCTAACCAGTTCACAAGATTCTGTCAACAAAATCTTCTCTTTTCAGGGGG[A/G]AAAAAAGGGGGAAGGGGGAGATGTTCATTTCATACAATGCAAAGAGATGACATAATCAGGATAAAGATGTACTTTTTTCACTTTAACTCAGATGGGTTAT
15	42438361	SCAFFOLD110137_21000	0.33	0.35	CAAGCATGACAGCATGTATTAGGAGATGATGCTTGAAACGTATCTAAAAACCAGATAGATGCAACTTCATACCTAAAAATAAACCAAGTTTTAAACTCTA[A/T]CCAGTTCACAAGATTCTGTCAACAAAATCTTCTCTTTTCAGGGGGAAAAAAAGGGGGAAGGGGGAGATGTTCATTTCATACAATGCAAAGAGATGACATA
15	42438540	SCAFFOLD110137_20821	0.08	0.18	ACCAGAGGAACACAAGGAACACAAAACAACTCAGTCTCAGTCTTCATCAGAAAAGGCTTCAGAGATGAGCTTATGCCTGAACTCATTAGGCAGACAAGAG[A/G]AAGAAGGGTATTTAATGGGCACAAAAAAAAAGTGCATAAAGGAGGTTAAAGAAAGCTTGGAGAATTTAGGTAGGTGGTCAAGCATGACAGCATGTATTAG
15	43366680	SCAFFOLD220358_6653	0.42	0.47	AGTTCAAGGGAGGCAAAGGTGTCAACAGTGGACAGGAATGGGGTCCACGTGGTGTGTGAAAAATAATAAAATGAAAATAATCAGGGTACCGTCCCATAAG[A/G]CAGTTGGGAGGCGAGCACTGCTAAGTATTCAATCATAGATATCTCCCTTCAGAATACCCTTAGCCCCCAGGAGAGTCCAGCCCTGGCTCCCTGCTGGCCC

15	43389650	SCAFFOLD290930_14926	0.35	0.46	AAGCTGAATGCCTCCCCCTCCTCAACTCTAAGGGCCACCTCCAGTTCTTAGCCAGACTTCTGGGTCCCTTCTACAACTTGTGCACCTGGTGGGGATGCCT[A/G]TGACATCTGTCAGGCTGTCCACTGGGACTCTATTCCAGCCCAGAGGAAGGGAAATGGGCAGATGGCATGATCCCAGCCAGTGTCTGCAGAGGGGGATACA
15	43389969	SCAFFOLD290930_15245	0.34	0.46	CTCACGTGGAAGGCCCAGCAATATTTTCAGGCCGCATCGGGGAGCCTGTTGGACCTCCCAGCCCCCTTCAGCATCATTAATGAAGCTGGGAGCAGACAGA[A/G]GCTCCAGGAATATTAGTCAGGGGCCCCCAGTGGGTGAGTCTAAGGAAGGCTTGTGCCCAGGACCTGGCCCTGGCCACCAGGGGCTGCAGAGTTTTATTGG
15	44054675	SCAFFOLD136998_2632	0.46	0.33	CCTCATCTACAGTCTGAGGAACAGAGACGTAAAGGAGGCCCTGAGAAAGGTAATTCTAAGAATATATTCATAGGATCCATTCCCAACATTTCAGATGCTC[A/G]TTCATCAGCATTACATTTGTGTGTGTCTGTGTGTTATTCGCTCTGTTGTATTCGACTCTGTGCAACCCCATTAACTATAGCCTGCCAAGTTCCTCTGTTC
15	44054706	SCAFFOLD136998_2601	0.45	0.33	GGGGTTGCACAGAGTCGAATACAACAGAGCGAATAACACACAGACACACACAAATGTAATGCTGATGAACGAGCATCTGAAATGTTGGGAATGGATCCTA[A/T]GAATATATTCTTAGAATTACCTTTCTCAGGGCCTCCTTTACGTCTCTGTTCCTCAGACTGTAGATGAGGGGGTTCAACATGGGGATCAACACTGTGTAGA
15	45562206	SCAFFOLD280628_12261	0.35	0.45	TCAGGAAGGAGGTCATTGTCAGACACAGATGTTTGCTCCTTGAAGGCTTGAGTAGTGGGAGAGAGCTGGGAAGCTTTCTGAGCAGGGGTGGGGCCTGGAC[A/G]GAGGTGATTTGAAGAGCTGGGGTGAGGACTTAGAGCTCACTGGGTCTTTTCTGTACCTAGGATCTGACCAACAGCCACTTCTTCTTGTTTGATTTTGAAA
15	45564389	SCAFFOLD308266_439	0.36	0.45	CGATGACTTGAATTACCTCCCCTGGCTGCTGAGTTTATGTCCCGCTTCCAAATCATCATCTAGGTACCAAAATCCCAACATTTTGAATGGCGTCACCAGC[A/G]CAACCAGTCGCTTAAGCCAGAAACCCAAGATCTGCTTCAGAATTCCTCTCACCAAGCCTCAATGCCCCACCAGTCCTCCTAGCTGAAGTTCTTCCACCTG
15	45768306	SCAFFOLD125693_15530	0.10	0.16	TTTATATTTGAAGATGATCTCTGGAAAGATAACAAAGAAATTGGTAATAATGTTTGCCTCTGGGAAAGAGAACAGAATAGAGGGAAGATTTATTTTTTTT[A/T]AATTCTGTATCCTTTTGTCCCTTTTGAATTTTTAACTGTGTGAATGTGTTACCTTTTAAAAAATTAATAAAAATTATATTTAATTAATAAAAAATACAGG
15	51227965	BV105337-114-S	0.27	0.41	CACTATAGGCATAAGCAGCTATCCTGGAGACCTGGGACAGGGGGACGGGGAGAGACAAACAGARACAGAGGAGTGAGAAGGGAGATCCGGAGTGGGAGAG[C/G]GGGATTGGATGGTCAGGGAGGAGGGAATGATGRGGGACGGGGAGCCAAAGAGAAAGGCAYGGATGGGAAAGGCGGGCGGAAAGAACAAAGAATAGAAAAA
15	51746916	SCAFFOLD100290_3465	0.24	0.32	CCACGCAATACTGCTGTCTTTGCCATGTTTCATCAAGTTGAGGCCCCGGAGGCTCCTATTGGGATGTCATCCCTGATCCCTAGGAAAAGTACTTTAAGCT[A/G]TCATTAACTCTACACAGTTCAGATCCCCTTCCCCAGGAGACAAATGGGGCCACTTATGAGAAGGGACAGCAGAGTCAGAATCAGGAGTGGAGGAAGGAGA
15	52369503	SCAFFOLD316738_25851	0.46	0.43	CAGCATGACGCCTCCCAGGCCCAGTGACTGCAGGGAGAGGATGGCAAGGAGGGGAGCAAAACTGACCGCCCTTAGCCCCCAGCACATACGTTCATCCTGA[A/G]ACAGAGGCTGGCAAAAGCCTGTGGACCCAGGGTACCCTGGTGACAGCCTCAGCCTCCAGGCTGACCTGTATCGGCGCTCAGCACAGGACTGCCAAGCACT
15	52710098	SCAFFOLD126586_1320	0.29	0.44	GGGGCAGGTAGATATTTGTTCACCTAGGACCTGGAGCAGAAATGGAGGTTGACAGCAAATGAGAGCAAGAGATCTCTTTGGGGTAATTAAAAATGGCTTA[A/C]ACTGGATTTTGGTGATAGTTGCACAACTCAGTAAATTTACTAAACAGCATTGAATAGTGTACTTAAAATGGGTGAATTTTATCATATATGTTATACCTCA

15	52710350	SCAFFOLD126586_1068	0.29	0.43	AAATATAGAAATTTTTAATGCACTAAAAGGAATTAAGTCATTTAATGCAATATCCTCATTTATACACATGAACTGAAATCTCGTGACAAGCTCTGAGCAA[A/C]GTGTTACTAAGTCAGAGATAACAGGACATAATTTCTGTCCCTGAGGTGTTTGTATGACGGAGAAAAACACAAAAACTAACAATAGCAAAATTGTACTGTT
15	52967013	UCP2_119F1-SNP1	0.13	0.13	AGCCTGCATTGGCCCCTGCCTTTCTCCTTGGATCTGCAAGGCCAAGGCAGGTGGTTGCCCTCCCCATCTCCTGCCATCCCTGTGCTCTAAAATTTATGGT[C/G]CCCAAAACCCCACTGGCCCTTGAAGAGTCTGTGTCCTGGGGAGGGAGTGGACTAGCTACGCTCAGAAATGCAGGCTTCATTCACTCACCTGTAGCCGGAC
15	53109621	AF127030-1099	0.14	0.24	CCGTGAGTGCTGAGGGGTGGGTCAAGGCAGGATCTAGGCTGTGGGTCAGGAGATCAGGGTGGTGCAGACTCAGAGGCAGAGCTGAGGCATGGGAAGCTGA[A/G]GCATGAGGAGACTGAGCCTCATGTCGAGGAGGATTTGGAACCCTGGCCTGGGAGAGGTGGGCATGGGGCAGGGGCAGCCCCCAGAGACCGGCATCAGGCC
15	53110041	AF127030-679	0.47	0.09	CCCCGCAGAGTATGAGGACTTGGGTGGGGGGTGGGCAGGGCTGTGTGCAGGGGTCCCTGCCTGGGGCTGGACCTCGGAACAAGCATGGTCACAGTGTCAC[A/G]TCTCAGTAAAAATGACAGTTCTGTGTAGCAGTTGCCAGCTCACAAACTGTTGTACACAGTGTGGTAAATCGGAGGGTGATGGCTTTTCTCATTACAAGTT
15	53297053	SCAFFOLD91016_8628	0.27	0.34	CTTTGGAATCATTACCATCAGTATCCTTAGGTATTTATTAAGTTAGCCCAGCGTTGACATTGTCTACTTGACTAATTTGGTATGCTTCTTTGTAAAGTCA[A/G]ATTTCCCTACTGTAGCTAAGAAAACATATGGTCAACATTTCAAGTGCTTATAGCTGTGTTTACCTATCTTCTAGGTTTATTGGGAAAATGGTGCTCAGAT
15	53989613	SCAFFOLD399_5007	0.05	0.08	CCTCCTCCCCACCCTAGGTCTTCTACACCAACTGCAGCTGCGTGGCGGGGGGCGGCCCCGTGCCAGCCGGCTCCTGCGACTCGGCCTGCGGCCACCTGGT[A/G]CTTCCCTTCATGATCCTGGTCAGCCTGGGTGCTGCGCTGGCGAGTGTCACCCACACACCCTCCTTCATGCTCATCCTAAGGTGAAAGCAGGGGAGTGCCT
15	54738581	SCAFFOLD230633_1881	0.18	0.23	CACCTTGTTCAACCCTGCACACATGATCGCTGTGGATGGGTAAGTTAAGGCATGACTTCAGATTTCCCATCCATTCCCACCCTCCCAAGATAACACCTGC[A/G]AGTTTTTACCTGAATGGGAAAGTAGTCTCGAAAGTAGCGCCACACAGCCCAGTTTCGGACCCACTGTGACCTCCTGCCACCTTCCAGGGAACAGAACACA
15	54738901	SCAFFOLD230633_1561	0.17	0.22	TCTGAAACAGCTTTTAGATCCAAATGCTAGAAGAAGTCTTGTTTAGCCAAGGAACAAAGAAGCTTCTTTCTTTCTGAAAATGGGGATCAATAACTAGCAT[C/G]CTTCCCTTTTTGCCTTGGCTGCTAGGAGGCTCAGAGGTGATCATGAAGTTCAATCACTGCAACTCTGTTGACACTTATGGATGAATTACTAGTATGTTTT
15	54912667	BES7_Contig323_581	0.33	0.46	TGAAAGTTGATTTCTAACATTTAAATACTAACAAAAGTCTCAGTGAAACATTAAGGGGGGAAAAAAAGAAAAAACTACCTGAAGGTTTTGGAGGAGTGGA[A/G]AAAGAGAGAAATTAATACAAGAAGAATATGATGATCACACTTAGAAATCAAGGAATTTAAAGATGTTAATATATAAAAACCTACCAGCCCATTGCTTGTA
15	54913026	BES7_Contig323_940	0.49	0.30	AGCTGCCAGAAGAAAAAGAAATAGACTAATCTGCTTTAAAAATTTTTTTAATTATTTGGATTGAATTTAGTAAATTAGTTTATGGACAGTGTGTTTATGG[A/C]CAGTGTTCTTTTTCGGGAAAGATTCTGTTAAAGAGATATTTCAAAGAATAGAAATTCCAACCCAAATAGACAGATTTAAGATACTAGAATAATTTTGGGG
15	57793631	SCAFFOLD165004_21987	0.49	0.30	GAGTCTTTTCTCTTTGGCTTTCATCCTTCTTGCCCTCCTGGACTTCTTAGCACTCATTACCACCTAAAAGTATGTTGCTTTCTTGTTCCTATGTATATGT[A/G]TGTGTATGACACAGGATTCTAGCTCAAAAAAGAAAACATCTAAACCAGAGAAGGCAATGGCACTCCACTCCAGTACTCTTGCCTGGAAAATCCCATGGAC

15	58196359	SCAFFOLD251470_3902	0.21	0.24	AATACTCATTGTTAAGAGGTCACCAGACTATTGAAAAACATTGCTACAATAACATGAAAGACCAATGCAGAGAAAGCCTGAATTCTATCTGTATATCTAG[A/C]ATTTATTAGGCATCCATCATTTCTAAGTTTTGTAAAGAAACTTTAAAGACGCACCCTAGGCTCAAGACTCGTGCCATCTAAGAGTTTAGATGTGCAAACA
15	58214266	SCAFFOLD70668_8488	0.26	0.40	CCACTATTGCTATAGCTTTGGGAAAAAAAAATATTGACTGTCAACTGCAGTGAGCTTTCCCAGCACTTTTTTCGTGCTCGATTTTTAAATTAACATTACC[A/G]TCTCCCACCACTGAACACTGCTCCACCTGGGAGCAGCCATTGCTCTGGTGTTTTTTAGGAAACGACAGTCTAGATGGTTTGAGTGATGTTTTCAGGGCCC
15	58864058	SCAFFOLD266079_8266	0.40	0.40	AACCTGAAGATGCTTTCTTGCTAGTCTTGAAAATGAAGGAAGGCTGTCTTAGCCAAGGAATGTAGAAAGCCTCTAGAAACTGAAAAAGCCACGCCCCCCC[A/C]AAAAAAAATCTTTTAGAGCCTCCAAAAGGAATGCAGTCCTCTCAGCTATTTGATTTTAGCCCTGTGAGACATATTTTGGATTTGACTTCTAGAACTCTAA
15	61633521	SCAFFOLD112140_2309	0.38	0.48	GGGATTGAACTGCATCTCTTTTGTCTCCTGCATTGGCAGGCAGGTTCTTTACTGTGAGTACCACCTGGGAAGCCCACAAAACAGAGTACAAATCTCAAAC[A/G]TTTCCGGGTGCCATTGTGCCTTTGATAGAGCAAAATCAAGTCTCCAAAATATAAGAAAGGCCTGGAAGGTATTAAGGATCAAAATTAGCATCTTTTTTGT
15	62525217	SCAFFOLD154897_7901	0.19	0.17	CCCCAGGCTTTTGGGCAGGCCCTGAAAGCCAGGGAGACATGCCTTCCGCCAGAGGCCTCGATGGCCAGGCAGCTGCCGGGAGGTGGCGCTGTGTCACTTT[C/G]CTACTCGGGAGCTTTTGTTTTCTGCGGGCCTCACGCTGGAGGTGGGGAGTCTGGTTTGAGCAGGGCCTGACGCTAATTTCCCTGATTTGAAATCTCGCTC
15	63067412	SCAFFOLD122789_23101	0.16	0.21	AATAGGTTTAGAGGAGTGAAAGACGCAGATGTTGGTTAAGAATTCCTAAAAGGGAGAGTAACATATGCAAAAGAAGGAATAATTAATTAAACGAAAAAAA[A/T]TTTTCTGAGTTGATAATTTAGCATGAGTGGGAGTAAAGGACACATCAGATGAAACAAGGAAGCCATATGTTGGTAACTGTTGAAACCAAATGATGACTAC
15	63498829	SCAFFOLD125682_3725	0.47	0.05	TCTTATGGACCTAGAAACACTTAAATTGATTATTCTGCCAGTCAAATATCCTGATTTACCAAGAAAACTTACAGAATTATTCCTTTTTACATGAGTAGTT[A/G]TTTAATAGCATTGAGAAAGTAAGCCTTTGGCGATTAGCCAAAGAAGACTGCATCTATGTATAGGCTTGCAGTTCAGAATGAGGCTGGGATCATTTGTATC
15	63498921	SCAFFOLD125682_3633	0.15	0.16	GAGTAGTTATTTAATAGCATTGAGAAAGTAAGCCTTTGGCGATTAGCCAAAGAAGACTGCATCTATGTATAGGCTTGCAGTTCAGAATGAGGCTGGGATC[A/G]TTTGTATCGAGGGTGCAATTAGCCTGGCCTGAGCTGCATTTTGAAGGATGACTTTATGACAGTCTGTCATGACAACAAGGGAATATTTTATGTACAGAAT
15	65080431	SCAFFOLD225528_7850	0.18	0.17	CACTTTCACTTTCATCACTGACAAAATGATATCATACGGTGGATCAAGTCCCAGCAGTATTCTGCATAATCGTCAGATCGTTTCAGTCTCTTTGGACTAC[A/G]CTCAATAGAGAGGATGGACATAGACATAGCCCTGCGTCTATAAATTTGTGCTGTCCCTGCCATTAGGGTCTGTGCTTGCCCTTCAGCTCTTTTTGCCTTC
15	65207827	SCAFFOLD21190_13543	0.23	0.27	AGGAAGTCATTTATTAAGGGTCTGCTAATATCTCCCAGGGCTACCCTAAGAAGTCAGGTTCCCTTATGGCACAGGAATAATGTGGTTGAATGTCAATACC[A/C]CTCTACCCTCTTCTCTTCTCTTCTCCTCAAAGTCATGTCAGTCATCATATGAGGCAGGAAGGCTCACAAACATTAAATATTCCCTAGCAATTAGATTTAA
15	65208287	SCAFFOLD21190_14003	0.37	0.34	ATTGCTTAAGACCTACCTTTACAGAATCAGCAATCTCCTGTAGACCCTTAGCAGCAGCATCTTTTATGACTGGTGTAATTAGACCTCTATCTGTTGCCAC[A/T]GCCACTGAAATGTCAATAGAGGGCAGTTGCTTTGCACCCTCTCCGTCCCAGCTTGCATTAACATTAGGCATTTGCTAGAAAGTACAAGTAGAAAAAAACA

15	66176134	BES3_Contig318_1105	0.48	0.42	TCAAGAGGAATCCAATTCCAAGATGTGAATTCCTGCATCTCAAGGTTCCAGACACAAGAGCACTCATGAAATAAGGTCAGAGGCTACTACAAGCTCCAAC[A/T]CACATATACACACTATTCAATTTATGATTGCTCCATAGGAAATCGGACTCCTACATCTGCAGCTATAAAAGAGCTGATGTAAGAGAGACAATGGTCAAAA
15	67558380	SCAFFOLD110429_9247	0.22	0.23	AGCAAAAACTTTTGTTTGGACAGAAAGCTTCCTAAGAAACTTTAAAATAAGTTAAAAAGGGCACTATAGATAGTGATAAAAGGAAGATAATTAAAAAAAC[A/G]TCAGCTCCTTGAACTTCCTCCCACCCCAGTCTTCCTCCTGTGACAACCGAGGGGATTTTCCCATTTATGAAAACTTAAAAATTCAGTTGAATATATAGTC
15	70390929	SCAFFOLD185092_13896	0.35	0.48	TCTGTGAGTTCTTCGGACCTCACCAACCCTTTGAGAAGGCAGCCACCGAGTGCATGGGTGAAGTTGTTGAATAGAAGGCGTTCACTCCCACTGCTAAGGC[A/G]AAAAAAAGAAAGACTCTATTCTAGTCTGCTTGGGAAGATAAAAGTAAAAAAGCTTCTTTCTTTGAAAGGGGGGGAAAGACTCATTTACTTTTATTGCTGT
15	70632622	BES8_Contig340_1050	0.48	0.45	TGAAGCTAGAGCAGGAATTAAATTAATTTGTGCCTTCACCTTAGCCCAGCCTTTCTCTTTGTATAATGTATCTGTGACATTTGTCTTCATTCCGAGAGAA[A/G]ATGAGGAAAACTCATCAGAACAAATGACCAGTTCTCATTAAACTGATGATTTTGAGGGGGATTTCCTTTGATTCCATGTAATGATACATTACCTTCCCAA
15	70632844	BES8_Contig340_828	0.48	0.45	GCTCTTCAGCTGTTGTAAGTATAGAATATATGATGCAATTACACTGAGTTTATGTTTCTTGAGTTAATAAATTGGTCTCAATTGCTAATGGATTCTTTAT[A/G]CTGATTGAATTTGCATGTTGAATTGGTTTAAATTGCCTGACCATATTTCAAGGTTAAATACAAAAGTATGTGGAGCTATTAATTTTGTGTTGCTGTTGTT
15	71952277	BES2_Contig423_1259	0.33	0.39	GCAATTGGATTTTTATAGCTGGTATTCAAAGTTAGCAGATAAGCAGTTGGAAAGAGAAAGTGCTGTTGAAAAATGGAAAACAAATCTAGAGAAATAAGAC[A/G]GTCTGGACTTGGTTTTGTCCCTCACTGGTTGTACAGACTTGAAAACATCTTGAACTTCTCTGATTGTCTATGTCCTCATCTCTTAAAGGAAAAGAATGGA
15	72074093	SCAFFOLD145103_39605	0.44	0.50	TGCCATGGGAGACCGAGAGGAAACATAAGGCAAAATCCCTGACTTACAGGAATTTAACAGATGTGTTAATAAGGCAATATGCACAGGAGCCCAGCAGAGA[A/C]CCTCTGATGAAGAGATAAATGTGTTAACACATTCTGTATAATTAACACCATGGGCTGTCAACAAAGTGAGCAGTGTTGAGTAGAGTTACCAGGAACGGTT
15	73400986	SCAFFOLD15042_9733	0.32	0.31	AGACTGGTAACTCCCCCGGGTGATAGCTCTGTCCCCTTCCCTCCCACACAAGCCCAAAGGCCCTGCCTCCTGGTCCAGCTCTGGGAGTCCGCTCAGGGTT[C/G]CTGTGATGAGTGATACAGTCCCCCCAGCTCCTTCCTTCCTTGGGGTCCTTGCCATCAAGCCCACAGTTTCCTTTCCACATTAAAGGTTTGATTCCCAATC
15	74261174	SCAFFOLD131120_4581	0.17	0.15	GCTCACAGGAAACAGGGATCAGCCTGGGGCAGAAACTGGAGGTCAGTGTGGCCAACAGGCACTGAAGGAGGGAGACTGGCTGAAATTAGAGCTCTCAGGC[A/C]GGGGCAAAGGCAAGTAACTGCAAAAAGTACATTTTCCTGGCAAATGTATTTTCTGATAAGGTAGTGAGTATGCAGCAAGGCTGAGGGAAAACCAAGAAGT
15	74368508	SCAFFOLD186847_18207	0.35	0.34	TCTGATTTAAGATGTCTCAAATCATCTCATCCTGTCCACCAAATTGCAGTTCTCCTGGAAAGGAAGCAAGCCTTGCCAATAATCACAGCTCAAGTGTCTC[C/G]GCCTGAGGATAAGTGAACTTCACAGGCTTAATTATTTCTGCAGGACCTCTTTAACACCTTGCAGCATTTAGAAGGCATAATTTACAGCCCCCCCAGGACT
15	74993659	SCAFFOLD138415_11603	0.06	0.14	AATTTGCTCACCCTGTGTTCATAACCCCGGGTGTGGTGTGCTGGGGCTGTGCCAGGCACTTCAGGGCATCTTCCCAACCACAGATGCAAGCCTGTGTCAG[A/G]AGCCCGACTAGCGTCTGTGGACACTGAGGCATGGGAGGTTTCATAAGCTGCCCATGATCACACGAAAAGTCCAAGATGGGGCTGAGCCAGGATCAGGCTT

15	75731335	SCAFFOLD141205_5870	0.40	0.31	TGCCCACTGCCCATCCACCTCTCCCAACCTGCTGGGTTCCTCCAGGCTGGGCCCCACCCCTCCACCCAGTGGGGCACTCAGCCACCTTCCCCTGGGACTC[A/C]AGCCGCTGCCCGCTGCCTAGAATGCCTTGTTGTCTGATTTCGCTTAGATACGGTCCCGATCACCCTTGGGGGTCCTTGCTGTAGCGCCCGATCCACGCCA
15	77146056	SCAFFOLD1004_27707	0.29	0.23	TGGGGATTCTGGGATCCTACAAACCTTGTGGCCAAAAAACAACAAAAGAGTACATATTTTAAAACATTTAAACATGATATAGCATCTTTAAAATTTTTTT[A/T]AATTATTTATTGACCATGTTGGGTCTCCGTTGCTGCGTGGGCTTTTCTCTAGTTGTGAGTGGGGGCCACTCTCCAGTTGAGGTGCGTGGGCTTCTCAATA
15	77154724	SCAFFOLD1004_19758	0.12	0.23	CGCCTTGAGGAACAGCCCAGGGTTAGATGACTGATCAGATCAGCAATAGGCTGATACCCCTTTAATTCACATTCCCAGAGTTGGATTCCCACTACACTGT[A/C]AGCTCCAAGGGGCAGGGACCAAGTATTTATAAGTACATGCATGCATGTGTGCATGCTAAGTAGCTTTTCTGTGACCCTGTGGATTGTAGCCTGCCAGGCT
15	77489176	SCAFFOLD80621_7495	0.38	0.44	ACCTCTAGGAGGCAGCCACCCAGCCTCAGTCATCCAGCCCAGGAAAACAGAAGACACGGAGGAGGAACTGTCATTCGTGCACGTCCCAGATCTCAGAGAG[A/C]AGCGGGGGAAGCTTCTTATCCTGCAGGCGCAGGGCAAACACTTGCTCTGAGTGGACGCTGCTCAGTGTCCGGAGGCTCACCAGTTTCATCAGCATCCTTG
15	78342210	SCAFFOLD171891_21805	0.47	0.47	TTCCTCAAGGTCTGAAAAGGATGCTTCTAATTATTGAAAAGACATCTTTGCTTTCTGTGATTTCAGGTGTCAGCTCCTGAGCTTTAGAACATGGCTGCTA[A/C]AAGCAATGTGACTGAAATCATTTTCTTGGGATTCTCCCAGAACCAGGGTGCCCAGAAGGTCATTTCTGTGCTGTTTCTCCTCTTGTACGTGGCCATCCTG
15	80755952	SCAFFOLD150011_3588	0.30	0.26	TCTGACTTAATCAGGTGAAAGCCCTTAAAGGAGAGATTCTCCTGCAGACCAGGCAGCTCGGAGCTGCGGGGTAGTGAGGGAGCCTATGAGGCAGCCACGC[A/G]ACAAGGACTGTGCTCACCCTCTAGAGCTGAGCATGGCCCCAGCAGATGGTCAGCACAAAACACAAAGACCTCAGTCTCACAGCCGCCAAGAAACCAATTT
15	80779274	SCAFFOLD150011_25775	0.38	0.38	CATAAATCTCCAGACTGCCTCACTCACCAGGCAAAAGAAAAGTTTTCTTGCAGAAGCAAGAAGATTGCAGCCCTGCAGCTTACAGACTGAGCCCAGAGAT[A/G]CAGGCAGATCACCCCTTGGGTGGCAGGTGTGGAAGCATAGGGTGCCTTCTACAGAGGGCCACTTCTCCAAGGTCAAGAAATGTAACCAACCTACCATGGA
15	83883810	SCAFFOLD250489_8604	0.08	0.07	GGGAGGGAAGGGGAGCTATTCTCCAGGGAGAGCTGACGAAGGCTCTTGGAGGAGGCCAGACAGAGGAAGGCAGGGAGATCTCAGGGACATGGATGGGGGC[A/G]ACCAGGGAGAGGAGAAGCCGGCCGGTCCCTCCGAGATGCGGGGCTCACCCTGCGAGCTTCACTCTGATACTTGACCGCCTTTTTGGTGTCTGCCACGGCC
16	486637	AJ505159-BULGE105-192	0.31	0.35	GGAAAGAATATAGGTGAGTTGAGTCAACTTTGACCTTTCTCCCTTCCGGCCCCAATGGAACTCTTGATTTATTTCTAGAGACAGATAACATTTCTTTGCT[A/G]ACAGCCCTTTGGAAGCAGTTGAGAGCAGGTAATACTATAGCCCTGTTGTTGCCAGGTTATCTGCTTCTAAAGTGCCATCTTGTCCGGTGTGTGTGGTTTC
16	1351048	BES4_Contig271_698	0.45	0.49	CTAGAGTCATCCATTCTGTTCTATTTCTCCTTTAGACCCCTTCCCCAGTTCCGCAACTTCTCTGACCCCTTATGCACACACACACAAGCTGTGTGCACAC[A/G]TGCATGTCCATGTGTTTGCACAAACACACACAACTTTGTAAATGTCATTTGCAGCTCTGTTCCAGTCCCCATTCTACACGCCATCTGCCAGAAGCTATTT
16	2143654	BES10_Contig687_1379	0.09	0.17	TTTGTTCGCACAACAATTAAACTGGTTAAACACGGCCTGACCCTAGGATTTCTGGCTGGGAGCCTGGGTTTGGTCTTCTTATCTAACAGGAACCTACAGT[A/G]CAAGAGTCCTTCTGGGCTGCCTTTAGGCCTAGTAGGGAAATATATTTCTTCCATATCTTAAAGGTCCAGCAACTTGTGTGCAAAACTCATCTGGCCAAAG

16	2635002	SCAFFOLD265480_4249	0.35	0.42	CGTCTTCTCCTTGGACTGCTGAGTTGGGTGAGTCTTGAAAGCTCCCTTTCTCTTCCAGACTGGAGCAGACACCAATGCCGAACATGTTTGGAAGTGGGCC[A/G]TTGATGACGGCAGTGGAGCAACTGGCAAATGAGGTGTGAGGAGTCTCACTGACTCTGGTCAGCTGTGGAAGGGGAAGAAAGTGTGGTGGAGAAGCCGCCT
16	3345341	SCAFFOLD150038_28634	0.19	0.13	TCTCAATAATTGATAAAAGAAGCAGACAGAGCACATGGTGTGCCTCAACAAATGTTAGCTAACGTGGGTATCACAATTCAGCCTGATAAACCTTTAGAGC[A/G]TTTCACAGGAGACCCAGGGCCAGAATCCCTGGAGAGGCGCGCACGCATCTATCGGGAGTGCTCCTAAGGAAGCCCCAGCCTCTGCAGAGGCAGACCCGGT
16	5594050	SCAFFOLD221404_5772	0.07	0.18	ATAAAAGGAAAATAATGATATTTGTCATAAGCAACACTATATATACATACACACATATTAAAACAAGGGAATCAAGTACTCAGGAGTGATGTATATGATT[A/G]AAACATGTAATGGTGAAAGTTGATTGGATAAGTTTTAGCCTTTTGACTAGTTCCTTTGCTGAAAGCTTGGGTATTAATTAAACCAAATATAAAAGATGTG
16	7693001	SCAFFOLD349064_338	0.34	0.43	CAGTTACATGCTGGCGATGGTGGTGGTGACCATAATACTAAGTCCAGTGAGTAAAAGACCAGGGTTTTCAGGCTTTATGTCTGTACAGCATTTGCCTATG[A/G]TTGCCTGTTTGCAGAACTGCCCAGAAGCCAAGAAGCTGGTCTATCAATTTGGGGGGAATTTATGGCCACAGAGAGTAAAACTCTAACCTGAAATATTCTC
16	8166330	SCAFFOLD121393_5256	0.16	0.10	GAGCAAACTCCTGGAGATAGTGAAGGACAGGGAAGCCTGGTGTGCTGTAGTCCATGGGGGTGCAAAGAGTTGAACACTGAGAACTGAACAACAATAAAAC[A/G]TAGGATGGAGTGAACAGAGGAACAGGCTTTTAAGTCACACATTATTCAAGAGGTACATTTAATTTTTTTTTTTTTAATTGAAAGATAGTTACTTTACAGT
16	8166692	SCAFFOLD121393_4894	0.22	0.26	GAGTTATTGGATCCAAGAGTTAGTGCTCAGTCTTCAGGGCCCATAGAGGGACCAGGGCAGAGGTTTGGGGGCTCAGTATGTAATTGTATACAGTGCTAAA[A/G]TCTCATTTTAGGGCGAGGGAAAAATAGAATTCTATGGTTCCTAATAACTACAGTTGGCATCAGAAGGAAATGTTTACTTTTTTTTTCATTAAAAAAATGC
16	9676226	SCAFFOLD142117_3231	0.28	0.20	AATAATAGTCACATTATTTTTGTTTCATTTATTATGGGTGTAAATATCATTTTCTTCATTAGAGGGAGAAGTAATTGCTTGATATTCTAATAAATGCCCC[A/G]AATCAAACATAAATACTTTACAAGATTTCATTATGTCATAAAATGTAGACACAAAATGTGAGGAAGCTCATTGCGTTTGCATCTTAGAAAGCTACACAAG
16	10493245	SCAFFOLD255638_2218	0.38	0.28	AGCAAGCTCAGTCAATACAGGGATGGGATTCCATTGGGTCCACTCGCCCACTCAAATGGGAAGGAAACGGATTAAGAGCAACTGAGCAAGCAAAGTCTGC[A/G]TTCATCCACACATTCTTAGAAAGTGCCTGGACATCCTATCTAAGCATTAAAAGTATATCTCTTTCTAATTCTGGGGCTTATTTTCTACATAATGAAAATG
16	11523618	SCAFFOLD311814_12304	0.28	0.18	AACCCTGGAAAGGGAAAAGATGCAAGTTTCTCTGACCAATGCAATGGACAAACAAGAGGTAGAATTAAGTTATTTATCATCAGTTATCATCTCTGATTTG[A/G]TTTTAATAGAAACAATGGAGCCTGTGGTTGTGATAAACCCGTATTTGAGAAATGCTTATTGATGTCTCCACATACTTTTAAATTTCAGCTCTTTCGAATG
16	12247408	SCAFFOLD280189_8316	0.46	0.03	TAGTATTTCCTTTGAGGAAAACAAAGAATCACGTTCAGTATGCATTATGTTTGAGATATCTATTAAACATGCTGGTAGAGCTGTAAGTGAGCAATTACAG[A/G]CATTTGGAAGTAAGATGAATGTGGAACTGTACCTGCTGGCAATCTTATTTGTCACTTGTCACCACAGACAAGATCCATGACTTGCATGTGGCTGAAGACC
16	13043273	SCAFFOLD150734_1074	0.24	0.40	TAGTCATAAATAGTCATTTAAATATTTGCTTTGACATTCTGTCACTGAATGTTCTCTAATACACTTTTAGATTTTAAGGTGTATAGGTTATTGATGAACA[A/C]ATTGAAATAAAAAAGAAATTTGAAAGCCAAAGAATATAAATATAATACATAAGATATAGATTCTAGGAAAACTAAAAGTTACTTTGTCATTAGAAGAACT

16	16080764	SCAFFOLD110541_12701	0.42	0.47	TTCTAATGATACTGCAGAATTACATGTATATTATGCATATTGTTTTGTATGCATGAAACACTGCAAAGTAGACTGTTTAAAGTTCAGTGCAACTTTTTAT[C/G]CTAGAATCAGTTCTTGTCCCAGGTACACATGCTAATGAACAGATTTGAAAAGATCCATGGTCTCCCTCTGCTTAACCCCTTGCTAGAGTACTATAGTGTT
16	16080875	SCAFFOLD110541_12812	0.44	0.49	TTCTTGTCCCAGGTACACATGCTAATGAACAGATTTGAAAAGATCCATGGTCTCCCTCTGCTTAACCCCTTGCTAGAGTACTATAGTGTTTCTCTTTGGT[A/G]CCTCTTCACTTCTTTGCCTTGTGAAGACTAAAACATTAAGCACCCTGGGCAGTTTTCTTAGGAATATCACTGAGATATTGGGCTGGAGATGTGTACATGC
16	16080987	SCAFFOLD110541_12924	0.44	0.50	CTTTGCCTTGTGAAGACTAAAACATTAAGCACCCTGGGCAGTTTTCTTAGGAATATCACTGAGATATTGGGCTGGAGATGTGTACATGCATCAGGAGAGC[A/G]CATAGACGAAGTTCATGTGTAAAGGACAGAGGGATTAAGGTTCCAAAATAACAGGAAAAAAGTTAAATTGATCTGAGAAATCTTTTGAGAGAGGAGAATG
16	16081016	SCAFFOLD110541_12953	0.45	0.49	CACCCTGGGCAGTTTTCTTAGGAATATCACTGAGATATTGGGCTGGAGATGTGTACATGCATCAGGAGAGCGCATAGACGAAGTTCATGTGTAAAGGACA[A/G]AGGGATTAAGGTTCCAAAATAACAGGAAAAAAGTTAAATTGATCTGAGAAATCTTTTGAGAGAGGAGAATGAGAACTGCCCAGAACTTCAAGGTGTCAGT
16	16081119	SCAFFOLD110541_13056	0.44	0.49	GGATTAAGGTTCCAAAATAACAGGAAAAAAGTTAAATTGATCTGAGAAATCTTTTGAGAGAGGAGAATGAGAACTGCCCAGAACTTCAAGGTGTCAGTGG[A/G]GAAGAAGACAAAGGATGTTTTCACCAGAGTTGCCACAGACTGTTTACAAATTCAATGTTGCCAATGCATGTTTAAGAAAAGCATAGTTTAGAGCATGCTC
16	17303527	SCAFFOLD115762_9415	0.44	0.07	AGTGACATTGAATACAATTTTGAGAAAAGTGATATCATTCGGGTGGCTGTAATTTTTTGGAGAGAGAATATCGGTTTCTCAGTTTATTTAACAACAACAA[A/C]AAAAAAAGGTCGTAAAAGGAATAACATTCGTATTAGCTAGTAAAGACTAGTTCAAACTGCATTGACCAAAGGACAAGATACCTAGAAAAACACTTTTGGT
16	18743547	SCAFFOLD604_14041	0.17	0.15	TAATCCAGGAGGACTTTACCTGTCAATCAAATCTATATTCATTATTTTTTTCAAGATTAATAATGGTTTGTTAACTGAGCCAGCTGGCAGACGTTAGGGC[A/G]ACGAGATGGGATCTTGGGGCTTCTATGACCAGCTGATGACTGCCTGACCAATTGCAAAAGATGCTTTGAAGGATCATAAAGTTTAGGTCCATGTGTATTT
16	19421840	SCAFFOLD115541_15491	0.12	0.20	TTTTACCACTGAGCCATTGTGGAAGCCTGCTGTGGATCATTTCACATCCTTTTCTTCCTCTTTATCTGCTCCCACTTGAATACTGTGATTGGGCTGTGGC[A/G]TTATTGTTATTATTTGCTGTTGACTACCCAGCACCTAGAACAGTGCTTGAAAGTGAAAGTGAAAGAGCAAGTCACTCAGTCATGTCCGACTCTTTGCGAC
16	19425194	SCAFFOLD115541_18845	0.41	0.42	GCTTCATGCTAACAGTGATCATCAGAAAGAACTGAGCGTTGTCAAAGGTTATCTTTCCATTCAAAGGTACCACATGAGAGGCGGAGATAGAACTTCATAC[A/C]AAATTTCGTCCTGTGAATAGAAATCATTGAATGACTGTCCAAAGAGGCCAGGAGGGTTTTAATAAAATATCCAAAGACCAGGGCAACAGTGACCTTGAAT
16	19483953	SCAFFOLD155072_21178	0.10	0.05	TGGCTCTGTTCCATACATTTCCTGCCAGGAGCTCACCTTATTCCTACAGTCAAGCATGGCAGTGTCTGAGTACTGAAGGCCATGGTCATGGTACCTCCTC[A/G]GTGGAGTTGCATTGAGGTTCAGTTGAATCAGTATATGGGAAGATGCTCTGGACAGTACCTGGTACACAGCAAGCTCTTCATGAGTCATAGCTATGATACT
16	19821482	BES8_Contig588_1285	0.47	0.07	GTTTCTGTCTATAAAAGGTGGCTGAAAGGACTGTTTCACTCTCACGTATTTTATAGCTGCCTGACATTCATTTACTGCCTCTCTTATTTGTCTGGTGTTA[A/G]TATTCATGCTAATGGGTGTTCCAAGTCTGGCCTCGGTCTCTGTGCATTTGGAGCCCTGCTATGCACAGCATATAAATGGTAATAAAAGAAAATTATCAAA

16	19821617	BES8_Contig588_1150	0.11	0.09	GGTCTCTGTGCATTTGGAGCCCTGCTATGCACAGCATATAAATGGTAATAAAAGAAAATTATCAAACTTGCCCCAGAAGAGAGGACATTGTGAGCAATAC[A/G]GTTTTCACATCCCTTAAAATGAAGGAAGAACAGGAATAGAAATGGGATCACACAGGAGAAAAATGGACTACATTCCTCAAAGGCACTTACCATAAAAAAG
16	19821722	BES8_Contig588_1045	0.20	0.21	ATCCCTGTCATGTTCATGGATATGGTTACTCTGTCTTAACCTCTTCCTCTGGTCCCCATCCCTTATTCCAAATTTCCCATTATCTAAGCAAAGACAACTT[A/G]GAATCTTTTTTATGGTAAGTGCCTTTGAGGAATGTAGTCCATTTTTCTCCTGTGTGATCCCATTTCTATTCCTGTTCTTCCTTCATTTTAAGGGATGTGA
16	19905494	SCAFFOLD99808_2825	0.29	0.38	GCCCTGTCTGTGACTCACAGAAGATTTCAAACATGTCTTATTCTATAGGAGTGTTTACGGAATACATGAATCAATACTCAAAGCAGAGAAGTGGTGACTT[C/G]CCAAGCGTTCCCCCTGCTCTCACACAGACTTCCCTTCACCTTTCAGTGAAATTTAAGAATGATCATTGCACACCTGAAGTTCTGCTCGGACCTGCACTTA
16	19905746	SCAFFOLD99808_2573	0.30	0.38	TTTTCACTTCACCATGGTCTATCTCTCACAGTGAGGAAGAGCAGACCTTACATGAGCTGGCACGTGAGACTCCTTAGCTATATGTTGCAGCTTTATAAAT[A/T]TTTTTTCTTGTTCTCCTGGACAGGCATCAATCTGCTGAAGAAACTTTCTAAGATATCACTGAACTCAGGTGTTCTCCAACTCCACCTCCCAAAATAATTC
16	19944216	SCAFFOLD305863_948	0.36	0.43	ACACTATGGGGAAGGCTGAGCAAACTGAATCCTTGAACATCTTTGGATCTTTAGAAGATCAAATAAAAAACAGCTGCAATTTTATTTGTTGCATTTCCAA[A/C]AAAACACAGTGACTTGTATCATGATGTGAAAACAAATATGCTCTGTATTAATGTAAATAAGAATATTTTTTCTAATTTGATGGAAGAAGGTTGACATGAA
16	19944281	SCAFFOLD305863_1013	0.36	0.42	AAAAACAGCTGCAATTTTATTTGTTGCATTTCCAAAAAAACACAGTGACTTGTATCATGATGTGAAAACAAATATGCTCTGTATTAATGTAAATAAGAAT[A/G]TTTTTTCTAATTTGATGGAAGAAGGTTGACATGAATGTGTGTGTGTGTGTCTGTGGCTGTGATGGGATTTTTGATCCTCTGATTAAATCTATTGCCAT
16	19944445	SCAFFOLD305863_1177	0.38	0.43	TGAACTGTACCGAACTAGTGGTAAAGGACATAAAATTCCCTGGATGCTGTGTGCTCTTAATTTTTTGTGATATCGATTAGTAATGAGGAGGGGTAGTGAA[A/C]TTTTTTTTTTTGCTGTCACCTTCTTACCCAATTAGTAGTAAAAAGAGTCTTCTAAAATGAAAAATGGCAATAGATTTAATCAGAGGATCAAAAATCCCAT
16	19944514	SCAFFOLD305863_1246	0.35	0.44	TGGTGATGGGCAGGGAAGCCTGGCGTGCTGCAGTCCACGGGATCGCAGAGTAGGACACAACTGAGCGACTGAACTGTACCGAACTAGTGGTAAAGGACAT[A/G]AAATTCCCTGGATGCTGTGTGCTCTTAATTTTTTGTGATATCGATTAGTAATGAGGAGGGGTAGTGAACTTTTTTTTTTTGCTGTCACCTTCTTACCCAA
16	21492142	SCAFFOLD141338_5396	0.47	0.41	TTCTACTTGCATGTGGTCTGTATTAAGAGTACATAATCTGTGTTCTCCTTCTGACGAACAGCTTACGGACTACTTTGATGAAAATAGTGTCTATATTACT[A/G]TAGCAAATGCTTGCTATCATCATATCAAATGTCTTTTTTCACAATGCTTATATTCTTGGGTAGGGTTTCCAAGCAGTGAAAAATAGTGTTATCCCTATAT
16	23741046	SCAFFOLD45030_15894	0.10	0.17	TGCGTGCGCGATATAATTTGTACTTGGTGATTGAAAGGGAAAGTAGATATTCCCATTATCCGTTATTTACTTCACACACAATTAGTTCTCTCTGGGAAGA[A/G]CACCAGCTCTGCAGCTCTCATTATTCCCTCCAGTGAAACTGCTGAAGCCAATAAGAAACTCCATGATCACAGCGGATGCCACACCAAGAGGGATGCTGTA
16	23741123	SCAFFOLD45030_15971	0.24	0.21	TAATGAGAGCTGCAGAGCTGGTGTTCTTCCCAGAGAGAACTAATTGTGTGTGAAGTAAATAACGGATAATGGGAATATCTACTTTCCCTTTCAATCACCA[A/T]GTACAAATTATATCGCGCACGCACCGCAGCGAGTGATGGAATTCCTCTCCTAAGCTGACTTTGGGGCATATTACAAGTGATAGAGAGGCTCTGTGTTGAG

16	25030837	SCAFFOLD141901_20810	0.40	0.50	AGGCTGAACAGACCAGGGAACCCATTCTGGAGCACTGCATGGATATGGAGGCAAGTCATCAACCTAGCCCACCTGCTGAGATTAGCTTCCAGCCCTGCCA[A/G]ATGAGGATGCCTGGCCAGTCAGTGGGCTGCTATGAAGCATATCCTACCAGGACAGGAAGCAAGTCAGCAGCCCCAACCAGATTCTGAGAAAGGCAGAAGG
16	25595042	SCAFFOLD35864_6131	0.12	0.10	AATAACATAAAGAATATAAAGAATGTTTCAGATGTGAGGTCCAAATCAAAGACAACAATAAAAAATACTGGCCTGCATGGAGCAAATCCCTTTCAACTGG[A/C]AAATTCCATCTACATGATGGCTATCAATTTCCAGAACAAAACAGGGGTAATCTACTAGTAATAAATTTTTTTTCAACATCTCCAAAAAGTTTTCCTTTCT
16	25821107	BES10_Contig546_154	0.21	0.25	GTGGTGGGTTGGGCTGCGAAGAGAGAACAATTACACAGTCACTCCATTAACCCAATTAGTAGTCACTAGCAGGAATTCTGTTCATCACCCGAAGAAGGAC[A/G]GTGGGAAAGGTGGGAAAAGGGGACAGCCAACCTGTGGAGGGTGACTGGGGCTGGGGTTGGGGAAGGCGGGATTGCTGTCGGAACAAAAGGTCTAGAAGTA
16	26399362	SCAFFOLD321742_20835	0.38	0.42	AGATGAGAGTCCTGCTGTGTTTTCTTCCAGAAGTGTGGGACTGTTTCAAACCCAGTACAGGGGTGGGGGCTTGGCTTATCTCTGCCGTAAATGAACAAAG[A/G]GTCTTCTCTCACAGACTCCCATCCACTGCACTGGGGGTAGGGCTGCAGAGCCTGTGGAATTGGGTCACCAGGTCTCCTTAGGAGGGCCAAACAGACTGTG
16	26907451	SCAFFOLD160090_5989	0.21	0.30	CTATACCATGATAGGTATCAGGGGTCAGGCTTCTCCTTGTTCGATGTCCTCAGTAGGTAGCTTTCACCTCATGGTCCAAATTGGCTGCCAGGCTCTAGTC[A/G]TCAAGTCAACACTCCATCTGGCATAAAAAGAGGATGAGAAATGGATGCACTTTCTTCTCTTGAACTTGCCCACAGCACTTCTGCTTATATTCTGTGGCCA
16	29309627	SCAFFOLD260075_12097	0.08	0.13	TCCTATCCCAGAGCCCAGTGTGTGCTAAGGACTCAAGCAGCGTTTGTGAGGTGTATCGGCAAGCCTCTCTGCAAAGGCCCCCATGTATACTCTGAGAGCC[A/G]TTCGCTCACTAGAGCGTGTCAGTTCATCACTCATGCCTCTGATGTGCCTGGAGAGCAAGGAGGGAGCACTAAAATGCCAGAACCCAGCCAGAGCTGAGGC
16	29384331	SCAFFOLD154713_4209	0.07	0.03	ACTCCCACTTTACAGAAGACCAGACTGGAGGTAAGTCACTTTCCATACAGCTCTCAGAGGAGATAGTGGGACTAGAACCTCACTGCCAAGTGTCCTGATT[A/G]CTGACCATGTGACCTTCCACTCTCAGCTCCCAATGACTTTCAGGGCCACTCTCTAAAAACAACTCCTGGGGATTTTCCCTGGTGGTCCAGCAGTTAAGAA
16	29384755	SCAFFOLD154713_3785	0.49	0.41	TCAGAGACACAGGGAAGTCACTCTGTCCCAGAGGATAACTTACAAGGGCTGTTTAGCTGATTTCATTTGCACCCAAATAAAGCAGAAAATGAAGAAACTC[A/G]GTAAAGCACTAGAGAGGTGACGCTTAGTCCAAGGTTAGGGAAGTGAAGTGAAAGTCGCTTAGTCTTGTTCAACTCTTTATGACCCCATGGACTGTAGCCT
16	29387200	SCAFFOLD154713_1340	0.09	0.03	TTTATATGTTCTTTGAAGAGTTGTATAGTTTTAGCTCCAATTTAGGTCTCTGATCCATTTTGAATTAAGATCATTCAGTTTTCCTGCAATAAAAATTTCT[A/C]ACAAACTCATTGAAGTTTCAACAAGGTAGATTTAGAATACCATTGTGCATTTTCTTTTCTGTACCTTATTTTTTAGCAACACTTAACATGGTGGCCATTA
16	30083257	SCAFFOLD296643_2746	0.17	0.33	TAAAGATAAGCTGAGGGAGGGGAGCTGTGGGGTGCATGCTCAGCTCATGGAAATTCTTCTGATTGGTTGGTGGTAAGCTAATTGGGAGTCAACATCATTA[A/C]TCTTTTGGTTCCCAACTGGCTTGGTTTCTATGTGCTTATGAGCAAAATATAGCTAACTTCATCCATTTGCAGGGGATTTCATTAGCTGCAGAAAAACTCA
16	30350211	BES10_Contig689_868	0.40	0.47	TCATCCTTTGTGCACTCAAGGTCACACCCTATTTAAGCGGGAATTGTAATATAGCCTGAGAACCCCAAGCCACCATAACTCAACTCTGCAAGCCGGAGTC[A/C]TCTTGGGTACTCAAAGCTCCCTGCCTCTTGATTAAAAGTCAAAGTGAAACCTAGAATAAGCTTGTCCAATCAAAGTATTTGGTTTTACCTTAGAAACTTT

16	30983359	SCAFFOLD125489_6932	0.05	0.13	ATTTTTTTTCATAACTCATTTCATACTGAACACAGTATTAGGCTATTCTTCCTCTGTCTTCTGAATTTAGGATCAAGGGCATGGGGGTTGTCTGTGGAAA[A/G]CATAATTTTGATTATCTACCAATTGGAAGAAGCTGCCACTTCAGTTCCTTTTACCATCCAAGTGTACCCTGGTCCCAAATTTGTCAGCATTAGAATTTCT
16	31157062	SCAFFOLD155509_6872	0.27	0.38	GCCGCCTTCCTTCTCTAACTTTATAATATATTGTGTATACTTGCAGGCAGCAACTGTGTTTTATTTCTATATCCCTGGCTTCTAGAAGAGTACTTGGTCT[A/G]TAGCAGACACTCAGTAAATTGCTGTTTATATGACAAGACATTTTGGCGTTTATTGTTAACATCAGTTCAGTTCAGTCGCTCAGTCATGTCCAACTCTTTG
16	31302860	BES10_Contig233_397	0.31	0.34	AATGGTACATTTGCTAAATGTTTAAAAAGGTTAGTTACATTTACATGAGCTACCACTGCCCTGTTTTCATCTCCAGCTGTTACTCCTATTGGATGTTACC[A/C]TATCATTAAACAAGTTTCTGTCATTATTGAACTTACCTATAAAATTAACTTAAAATACTATTTTTTGCCTCTTAAGTTACTGATAATGCTCACATATTAA
16	31465572	BES4_Contig468_902	0.47	0.37	TTGGTCTGCCTTAATTTAGTGAAGCAGCTTGAAAATATTTCATCTCACATTTCAAATATTTTGTTCCAAGGATACTGAGTAATAAAATGTACTCCCCAAC[A/G]TCTTGAATAATAAATCCATTTATTCATGAGGCAGCTAATTTTGTAAATTTCATGTAATTTAGGTTTTTCTGATCTGATCTAAGATTTTCCAAGAAAGAGG
16	35286684	SCAFFOLD101070_18932	0.46	0.14	ATGTGCTGTGGTTCATGGGGTCACAAATAGTCAGACAGGACTGAGCGACTGAACTGAACTGAACTTTCTACCATCATCTAATAAGACACTAGACCTTAGA[A/G]AAGGAAGACCAGAATCCTTACTTCCTTATCTCTGTACTTCTTTGGATCCTGAATTCATCACCCTGATTGAATAACTCTGGTGGCAACCTGCCCCTTGCTG
16	36760274	SCAFFOLD145525_4841	0.33	0.40	AACACAAACACACACAAAACGCAATTTTCAACAACATCGAAAAATATTCTAGAAATAAAACTTTCAAAAGTTTGGCTTTTTGGAGTACACAGAGATGCTA[C/G]GTTCTTCCCAAATTGATACATAGAATCAATGTAATCAACCAAAAGCCTAATAATTTTTCAATGAAACACTTAATAAATGATTCTAAATTAACATGGAAAC
16	37885962	SCAFFOLD125447_2382	0.06	0.14	AATGGCCTGTCTCTCTTCCTCCACCATTGAAGTCCCCAAGGGCAGAGGTCTGGTGGGTCTTGCTCCTCGCTATCTGCCCTGAGTGCAGCATCAGGATATC[A/G]TAGGTGCTTAATAAATGCTTGCTGCGTGGGTGGACATTGAATGACATGACATTCCCCGGGTGTGTCCTGTGCATCCTGTGCAGAGACAGGAGCTCTGCCG
16	43246647	SCAFFOLD143346_5166	0.44	0.03	TTTGTTGTACCTCTGAGATCTGACCCCCAGGCAACTTTCCAAGTCCCAGAGAGCATCCCCAGGTTTTAATCATCCTGAGATTCTCCCTTGCTGAAGGTCA[A/G]ACATGACCTTGAGACATCTATAAGAGGAATGACTAGATTCTGAGAAAGAGAGGCCAAGGGTGAGGGTGTTGGAGGGATTTTGGACCTGCAGGTCAGTGAT
16	43490232	SCAFFOLD152016_5856	0.25	0.29	GCAACTTCTGACTCTGATGATAACCATCCATTTAAATGCTCTTCTCTGCTAAGTGTTGTGACATGAACCCCCATAGTTCTCTCTCCCCCTTCTCTCTGAA[A/C]TGTGTTTATCCAAGGATCATTTTTAAAGCTGAGAAGGACATGAGACTTCGAGCCATCACCCTGCACAACCACATAAATAGAAGCAGGGAACAATTGGCTG
16	43490316	SCAFFOLD152016_5940	0.25	0.29	TGCTGTGAAGTGATGACATGTTATTGGTGTTTTTATTATGACTCTCTCACCAGAACCCAGATGGCCCTGGACTCTGCACACACACAGCCAATTGTTCCCT[A/G]CTTCTATTTATGTGGTTGTGCAGGGTGATGGCTCGAAGTCTCATGTCCTTCTCAGCTTTAAAAATGATCCTTGGATAAACACATTTCAGAGAGAAGGGGG
16	44613879	SCAFFOLD170764_5897	0.16	0.13	GCACCTTGACCTCATGTCAGATGGTATCCCAATAGCTGCTCAGCAGAGGCACCGAGGAAAGCCCTCTCTGGCCCTGGCATGGTCCTGCCTGGACATTCTC[A/G]GGTCTCCACTAAGCCCCTGATGGGACAGGGGACATTAGGCCCGCCAGGCCTGCCTGTGCCCACCTCAGGGGAGAGGGCAGCATGAGCCCCATGGGGCTAG

16	44614076	SCAFFOLD170764_6094	0.39	0.37	CTGCTCGGCTCCGGCTCTCCGCACGCGTCAGTGAGGCCCATGGGGCTGCAGGCTCTGAAATCAGGCTGGGCTGTAAGGAATGGAAAATTCCCCAAGAAAG[A/C]GACAGAGAGGTTGGAGTCCAGAAGCCTCAATGGCTTGGCGGTGTCCTGCTTTCAGTCCTCCCACGGTCCCCTGGAGGGAATGGAGTGGGCTGGTCACTAG
16	44984159	SCAFFOLD115010_51853	0.07	0.08	CTGTTGTCTCCTGAATTGATAGGTGGATTCTTTACCACTGAGCCACCAGGGAAGTCCCTCTGGTCTCTCAAGTATGTTATTTTGTTTAATGTCACAGCAA[A/C]TTTGGAGGTGGCAATGATGAATGCCATTTCTGCTTTGTGGATGAAGAGTTGTACATGATTTATCCACACTCAACCAGGTTGCCTGGGGCAGAGCAGGGCT
16	46152641	SCAFFOLD170182_27865	0.45	0.43	TGTGAATTAAGTTGACTGATGGCGAAATGTCAGTTTTATTAACGTTCTCCCTTCAGATGATAATTTTATATCTATAAGGATGAATGACAAAGGAGTTACT[A/G]AATGTGTCAATTTGGAATGATGGAGCCCTTTTAATAAACTCTACTTATTTTCTCATTCCTTATTTTTAAATATTTCACCTCCATCCAAGGAATTGTTTCC
16	46992465	SCAFFOLD266784_939	0.33	0.35	TCTTGCAGATACAAGTGTCCCTCCAGCTTCTTCCTCCTCGGCTCCGGGAGCAGGCTGCCGACACCCTGAAAGCCCCTCCCTTCACCATCAGCCCAAAAGG[A/G]AGCTGCCACCCCATGTAGGAGGAGGGGCCCCAGGCTGGGGGTATCCCTGTGCTGGGCAGCGAGCAGCCCCAAATGTCAGCCCTGATGTCAGCTGCAGGCC
16	48365146	SCAFFOLD278360_2768	0.50	0.03	GTTTCCCAGCGCAGGCTCCACCCTGGCCTCCATTAGTAGAAGCCAGGCCCTCACTAGCCTCTGGTCGTTTGTGGGTCTTGTGGGCGCCCAGGCATCACTC[A/G]GGAACTCCTCACAGGCGCTTGAAGCTGGCCCAGTTTGACTATGGAAAGAAGTGCTCGGAGATTGCGCAGCTGACGGAGGGCATGTCAGGCCGGGAGATCT
16	49123907	SCAFFOLD311704_43238	0.28	0.31	TTGGGAGGAGGCAGGAGTGTCCTCCCAACTTAAGTCGTGGTTTAGATGGTGACTTGATGGCCATTTTCCTCAGCAACAATCTGGCTGTCTGGGTGGGGGA[A/T]GTACTGCTGAGGTCACGTTGCAGGTACAGCAGGGGAAACGGGAAGTGAAAGATGAACCCAGAGCCAGCGAAGGCTCCCAGGCTTTGAAAAACTCCTTCCT
16	50369445	SCAFFOLD15094_34505	0.49	0.48	ACCACACTCTCTCACTGTAGGAAATACTCACAGAATGTCTGATAGATGCCAGGCAGTGTGAATGCCATGGGAGAGGTAGGGGCAGACATGTCCTCGTCTG[A/G]GTCAGACATGGAACTGGCTATCATTCTCAGCAAATGTAGCAACAAGTGAAAAAATAAAAGTGTTAGTTGCTCGGTCGTGTCTGACTCTTTGTGACCCCGT
16	51552296	SCAFFOLD111164_4522	0.32	0.47	GGGAAATTACAAGTGCCTGACTAGCTTTGCTCAAGCATGAGAAATAGTTCTGGAGGCCATGAATCCCATGTTAATGAATCAACAATACATAACCAATACA[A/G]TGTCTTTAGGCAAAAGCATACATAAAACAAGGTTATATAGGGATTGATTAACAAAAATGTGACCCGAGGCTCAAGAATCCTAACCCTGCCTTTTCCCTGG
16	52492126	SCAFFOLD55004_54933	0.49	0.49	TATAGTCGAAGGGATCTCTGTTCTTGGGTCAGGCCTCAGGAATTACAAATACAGGAAATAAAAAGGGTACTCGGGGCTGAAAACTTAATTTAGGACTCTA[A/G]AAAATCCTGGGACAGGAAAAAAGGACTTTGGCATTTGTGGTTGAATAACCAGGTATGGAATGTTTGAGTCATTTAGGCATCCGGATTAATTAGATTTTAT
16	53745273	SCAFFOLD261939_3134	0.28	0.19	GTTCTTTTTTATCTAGTGTGTGTTCTCAATAGCCTATCTTATTCCATGGTTCTTGGCACTTAATTTCCAGATGTACCCCACAGAAAATAAAAATAAACTA[A/T]TTCTCTTTGGGAACTGTCCAACCCCAGATCTGCTCTTTCCTTCAGATAGGATTATCGCTGTCCAGGGAATCACTTTCAGGAGGAAACCTGACAGTGGTCG
16	53747962	SCAFFOLD261939_1026	0.15	0.06	TTACTGTGTATGAGTGCCTATCAGAAACACTAATAGGAATGTCACGTAGGCCTATGGCCTATCCTTCCATGCAACCAATAATTCTCTTTCCCTCTCCCAA[A/C]TATTAAGTTCGTGGTTTGTTTTTCATTGGCTTTCTTAAATTTCCATAATAAAGTAGAAAAGTAATCTTTGTACTGATTTTGGGATATTTAATAGTATTTA

16	53748113	SCAFFOLD261939_875	0.17	0.06	AAGTAGAAAAGTAATCTTTGTACTGATTTTGGGATATTTAATAGTATTTATCTGGTTTCATTGTTTTCAAAACTTTCATCCTCTTATACTATGTATTAAT[A/G]TTTATGTTTCTCAAACCCTTACTCCATTTTCCTAAAATACTTTCCAGTTGATAACGAGTGGCTTCATTTCCTAGCTGAAGTGAGAGAAACAAGAGAATAC
16	54248312	SCAFFOLD81723_9670	0.15	0.20	AGTTCTTTCCATTCATGCGGGTAAACAGTAAACACTATGTTAAAATATTCATCACGGGGCCTCTGCTTGAATGTTAATCTGGGGTGGGAGCTAATTCCTC[A/G]TCCTCAGCTGGCTGGACAGTGTTGCTGGAACTGGGTGGAGGGAACTGGGTGCTGGAACTGCTGGAGCAGGGTGGTCAGAAAAGGTCGACCCCAGTGGAAA
16	54301386	SCAFFOLD215098_29103	0.24	0.27	TTTAGGATGTTGACCAGGGCTGTAGTCATCTGAAGACTCGCTGGGGCTAGAGGACTGGATTCTAAGATGGTTCACATATGTGATCTGGTTGTTGCAGAAG[C/G]CCTCCCTACTGAAGCATCTGCTGGGCTGCTTGAGTATCCTCACAACATGGTGGCTAGCTTCCCCCAGACCAAGCTACCCGAGAGAAAGCCAAGTGGGAGG
16	55147206	SCAFFOLD70736_3033	0.40	0.31	AGTCATACCAAAAGGTGTTAGTCCTCTTTCTTCCCTAAGCTGATTGCTGGCAGTCATAATGAATGCTGCTGCTTTCCTTCGCAAAGAGTTTGGTTTAGTG[A/G]AAAGAACTGCTAGAGATGGAAGACTTGGAAAGCACTGTTAGAAATAGAAGACCTGGTTTATAAAGTCAGCTTGACCACAGAGACTTTAGGCAAGTCAATT
16	56903851	BES1_Contig219_833	0.36	0.38	ATTTTTAATATCATAAAATTGGCTGTTCTGTAATATTTTGATTGGAGATTTATACATACTGTTGCCACATCCTTTAAACAGATGTACGGTAAATTTGATG[A/G]AAAAAAAATCTCTTCCTCCTGAAACTAGGACACTGGCTCATTTAAATAATATGGAACAACAACAGTTGCTGTGGATGTTTTAGAAACACAGGATGTTTTA
16	60750766	BES1_Contig550_727	0.38	0.39	CTCTAAGAAGTTTAATAATAAATCACTAAATCTCATGAGCATAGTAAATACAACCAGGGAATTATGTTCATATAAACTCAGTGGAATAACCACAAATTCA[A/G]CTTCCAAGAAGCATGAATTACAAATATAGTACACATACATTAAACTTTTCTGGAAGTCAAATTTTAAAGAAGTAATTTTCTTAGCATAGATCTTGGGCCA
16	60858370	SCAFFOLD313486_6482	0.48	0.45	GACTTGAGATATCTAAAACCAAATTCACATGTGATCCAGGGCTCTCAACACTACAAGGACATGTAGTGACAGCCCCTGGGTGTGTTGCTGAACATGTTAC[A/G]TGCAAAACACACACCTGTTAGGTGCCCTTTGAAGGCGAGCTCTGACACATTTACTTACTCCTTTGAATCCTTAAATTCAGACCATAGGAAAGGTCACCCC
16	61835341	SCAFFOLD112150_2535	0.37	0.45	ATAATGGCTTTGGAATAATTATGTTTGTCAAAGTAGCCCTGATTATTCGAACTTGTTTAATTATTTTTGCTATTTTATGCAGGGCAAAAAATACAGTTGC[A/G]TGGTCCTTATTGACAGGTGCTTTCCCTGCTTTCTAAGCCTTAAACTCTATGATCAGTGCCTGCTTTTGTGGCAAAAGAACAAAAACATGGCTTCGTGGCT
16	62535023	BES9_Contig216_874	0.29	0.17	AGGCAGCTGCCACCTTCTGCTCCCCCAGAGACTGGTAAAGGAACACTTTATATCTCACCAGGAGACGAAGCACCGTGGAACTGTCCGTGGGATTTATGGC[A/G]GTCTGGGAAAGCTTCTGGGCTGTGAGGAAGTGTTTCCATTCAGAGCAGGCTTCATTCACAGGGAATATTGGGTCACACAGAAGCTCACTACCCCCTGCTG
16	62930515	SCAFFOLD106832_3258	0.10	0.12	AGAATTGCCAATTGAAAATGCTATTCTGGGTGAAAGCCATTTTTATTATTCATCAAATTTTAAATATGTATTCCCAGGGCCCAGGAAAAGTAGAATAATT[A/G]CGTAATTCCATGTAATGGTTAATTAATATGTCCATTTGCCTGAGTCATGAGATACCCAAGTATTTGATCTAACATTTTTCTAGGTGTCACTGTAAGAGTG
16	62931039	SCAFFOLD106832_3782	0.11	0.12	AATGTTTGAATGAATACATTTGTTATTTTAATAAGAAAATTAATGCTCTAAAGGAGTGTTGGACTGACTCAAGGTCACTCAAGATATTTATTACTTGGGT[A/C]AGACAGGCATTTGACAAATTTTCTGGTTAAGTTAGGAAACTCTTTTTCCTAAACAAGACACAAAACCCATAGTCATGAAGGAAATGTTTGTCTACAGGAC

16	63286937	SCAFFOLD110132_1856	0.07	0.12	AAACAAGGAATGCAACAGAATTTAATTTTGCATCTTTCTTATGTGAAAGAACAAACAGCTGTAAATATCCTGCTAGAACATTTATTTTTAAAATTGTTTC[A/G]GAAGCAGTCATTCTTCCTGTGCATGCAAAGTAAAAAATCAAAGCCTGTGCCTCTTTTATCATTATGTTCAAGATTATGTTCATTCATTTATTCATTCACT
16	63558950	SCAFFOLD15298_5461	0.49	0.44	CAAAGCAATATATACATAATATAATCCCATTTTTCTAAGAGCAAACAACCTAAAAAAATATATCCCACAAAACATGTGTTTTATATGCTATATGAACATG[A/G]AGAAAAGCACAGAATAGCGTATGCTAAGTTGTTCACACTAGTTGCTTGAGTTGGAGGAGGGGATGTGGGGGAAGGAGAGAAAGTTAAGTTTTAGAAGGGC
16	63664599	SCAFFOLD206528_14609	0.30	0.30	GCAGAGGCATTGCAGCATCTCAGTAGAAACAGAACATCCTTGAGGGAGGTTTGGTGATGTGGCAGGATATCAGGTTTCAAGGTTCTGTAGAACTCATCAT[A/G]CAATGACATCCATCTCCTTTTATTTAAATCCAAGTGCTTGCAAGTGTACAAAAATTCGCATCAGAAATTAACCAGCTGTTCTGCTAGACTAAACAGTGGT
16	63846686	BV104037-256-R	0.27	0.26	GAAGAGAGAAAAGTACAAGCTCACATCCACCAGGCCATGAAAGCTCTACCTACCTTACCATCCATAATATATGACATAAAATAACAAAAATCAAATACAC[A/G]CTGTACTAAGAGGAAAAACAGAACATAYCRCTCAACCCCAACAATCAGTTCTGAATCTATAGCAGCAAGAATACTGCTAAGAATATAAGCAAATTTCTTC
16	64818695	SCAFFOLD61260_10267	0.28	0.43	TTGAAAGAAAAGTTTGCAGAAGATAGCCTTCTCTTCCTCCTCCTCTTCTTCCTTTTTGCATAGATTATTATACAGTACCCTCTGCCTTTTGAGTATGTGA[A/C]CAGGAAGTAAATAAATATGTATTGGTACTCATTAGAAGTATATACACAAATGCCGTATATGTTTGCTTCACATTCTGCCACTTGGTTTTTCTTGCAGGCT
16	65054477	SCAFFOLD251595_8246	0.27	0.38	TAACCATGGCCCTTTCTAGAAGAGCTGCCCATTCCCTTTGCAGCCCATCTCTTCCTTTCTTCCTATAGTTCAGTATTTGCTTCATGAGGAGATTACACTG[A/C]GGTAATACATTCTGAACGTTGAAACACATTTTGGGCTTCTCTGGGGGAGACATGGATTTCTGGCTGTCCTTCACTTTCCTCTTTTGTTCTTTGTACAGTG
16	65943749	BES8_Contig327_1499	0.03	0.10	AATGTTTATAAATAGAGACTTTTCTATGCGATATATTTTGTCATTTTTCAGAAAACACAAATATAGTGAACAGTTCAAATTTTACTTCTCTTGTTGTAAT[A/G]TCCAGGTTCTTCCCATTTAATACGTTGTATGTGCTCAGGTTTTGGTCGCTTCAGTTGTGTCTGACTCTTTGTGACCTCATGGACTGTGGCCTCCCCAGTC
16	68613275	SCAFFOLD150056_3359	0.20	0.21	CTATAAACAGGCTAAAGATGCACAGCTGGGAGTCATGAGCAAGTGCACAGCTACTAGAGACAATAAAGGATGAGAACACTTAGGGAGAATGTAGGAGGAA[A/G]TCCCAGAGCAAGGCTAATTGTCAGAGGGAAATCTACCTATGAGAAAACATTTCTATTTACTATTCATGAAATAGATAATTCGTAGGTAAGGAGTTGGTAT
16	69890274	SCAFFOLD81635_857	0.21	0.15	ATTACTTTGTAAATCATGACTGTTAAGTAACCATAAACAGATTTCTAAAGAATAATGTCTTTATTGTGATTTTAAAATTTATAGTGCAAAATATTTCATA[A/G]TGTGATATGGGCAATCTTTTTGAGGATTATACCAGAAAATGTAGGCCATGTCATTTCCATACTTTGTCCGCATACTGTATGGATGTGTGTGTGTTTGCGT
16	71401068	BES8_Contig485_491	0.30	0.28	TTGGATTTTTGTCTGCTGTTGGGGTGTTTATTATCTTGATGCTGCTCCTTTTTCTCTATATTAATAAGAAGTTCTGTTTTGAAAATGTTGGCGGGTTTCC[A/C]GATCTTGGTTCAGGATACAGTGCAAGGAAGAATTCACAAGATAAAATTTGTAAGTATCATATCGCTGCTTCTCTTGTTTAGTCTTTCTATCTACTGTTTA
16	72012419	SCAFFOLD102735_1234	0.46	0.48	AAAGATGGGATTGGATTTAACTGCCCAGCCTCCTGTTCCTGGAAGGGAAGACGTTCCCAAATGGATTGTCCTAAAAGCCCTGAGAGCCCAGGGCATCACT[A/G]CTGCTGACTAAGAGCTTTTTATGCACAGCACCTCATTTACTCCTCACACCATAATCTTGAGGGATAAGCTTGAGTAGCACTCACATTTTACTAATGAGCC

16	72012489	SCAFFOLD102735_1164	0.18	0.19	GACTCCCAGCTCCCCATCTTAACAGCCAAGTGATTTTAGGAAAGCAACTTGGACTCTCTGAGTCCTCACTGGCTCATTAGTAAAATGTGAGTGCTACTCA[A/G]GCTTATCCCTCAAGATTATGGTGTGAGGAGTAAATGAGGTGCTGTGCATAAAAAGCTCTTAGTCAGCAGCAGTGATGCCCTGGGCTCTCAGGGCTTTTAG
16	72055315	SCAFFOLD205344_9789	0.42	0.42	AATCTTCCCAATCCAGGGATCAAACCCAGGTCTCCTGCTTTGCAGGCGGATTCTTTACAGTCTGAGCCACCAGGGAAGCCCTAGCTTCTGGCTGTGGCCT[A/G]TAATTCTTGGCTTGCAGCTGCAACATTTCAGTCTCTGCCTCTGTTGTCATGATCGCGTTCTTCCCATGTATCTCTGTCTCTCTTATCTCTGTAAGGATAT
16	72055590	SCAFFOLD205344_10064	0.43	0.43	AAGAGAGCTCCCCCAAAGGCCAATTTAGTGATCTGAAGCAACTCTATGAATGCCTTAATGCCTGCTGCGTACACAGCCACCTTTTAAAGAAAGGGGCCCC[A/G]ATCCATCCTTTCCCACTTGCTGTTATGGGCTGACTTGTGTCCCCAAAAGATATGCTGAAGTTCTAACCCCCAGCGCCTTGGAACATAACCTTGTTGGGAA
16	72290010	SCAFFOLD210157_10565	0.22	0.29	GTCAGTCCTGGGAAATATAAAACATGGAGACGTGAAAGGTGCAAAGGTTCCACCACCTGCCAGCCCTTGCCGACATCCGGTCCAGTTAGTGAGAAGCACA[A/C]GATGTTCAGCGCAGGCAGAGGACTGAGCAACGGCCCTTCCTGTGCTCACCGGACCATCCACCCATCTCGTGCTCCAGGGAGCCTCTCACTGCCATGGGAA
16	73019259	SCAFFOLD150103_16092	0.24	0.35	AACTCTGCGTGACTTCAGTGACTAGGGAGCCACAGCCACCTGGAGAACTGAATGCAATTCCAAAAGATGGTAACGTTACCAGGGGCTGAGGGCGAGGAAA[A/T]GGGCACTCCCTATGGAAGGAATCAGTTCAGTTCAGTCGCTCAGTAGTATGTGACTCTGCGACTCCATGAATCGCAGCACGCCAGGCCTCCCTGTCCATCA
16	74258948	SCAFFOLD235979_4524	0.48	0.45	AAGACGTGTTATTTTTCCTGCTAGAACAAAATCCACTTACTACTTACATGCCACATCTGAATTCGAATTCAGCAACTTTGCTATTATCCACAGCAAAGCT[A/G]GTCAATTTATACATTTATTAAATATCAATAAACTCATTTCAAGTATGTGAGCTAAAAGGATTCTTTCTTCTTTGAGAGCTTGAAATGAGGAAAGCCTACT
16	74959635	SCAFFOLD20156_27190	0.24	0.23	GCACTATTAAAGTAATATTGCCAGGTTGTTTACTAATGTTTAAAATTTAAGCCTGAAATACAATTGAGAATAAGTAGATATGAAGGCAATTCACAATGAA[A/G]AATTTGAAGATTAAAAAATATCCTCTTATTAGCTGAAAACATTGAACTTATCAACATATGGTACCAGCTAGCCTCATCTCCACCCGTTCTTTTGTTATCT
16	76260197	SCAFFOLD15002_48007	0.04	0.08	GAGTTATGGGAAACACACCAGTAGAAAAACAAATGTAAAATTCCTTTCCTCCAAGCTCCCGTCCGGTTTAGCCACTACTAAACCAAAGTCAAGAAGAGTG[A/C]AAAATTAAATACACTCTCTAACCCTGCCCCATCCAGCTAGTGGGAGACTGAACTTGTATATCTTTGTCAAGATTTGAGAGATGTATGAAAATCTAGATCA
16	77338921	BES3_Contig484_1938	0.49	0.48	AGTCGCTGTAAACTTCCTGAATATGCTCTTCCAAGACATTATGACATATCCATTAGGAACTGATAGTCTTGTAAAATCAGTTTTCTGGTCAGCACTGTGC[A/G]TGAGCACATAGCAGAAAGCCCCATGGGTCTGTTTGTATTTGGATTTACTAAGAACAAGCACCCTACATGACTCATCACTCAATGGGACTGATTGTACCAG
16	77339298	BES3_Contig484_2315	0.47	0.47	TTGTACATGTGAGTCACTGCCGGTCTGCTCTATGGTGGTATCTGATGAGTTCGGTGGGATAGCGAAGGTGATTAAATGCCCACATCTGCCCAAAGGAGAG[A/G]GTTAGACTGTGCCTTGATTGGGAAAAATAATTACTAAAAACTGAGGTCGTAATTCCTCTTTTGCTGTTCCCCCAGCCTGGTTGGAACCTAAATTGTTCAT
16	77701428	SCAFFOLD135011_42532	0.07	0.19	GGCGGTGACTAGTCATGGAAACATTCTCGGGTTGGGTGCTGGGTGGATGGTCAGCGCCCAGCAGCAGAAACCCGCAGGCCAGGGAGAGCCCACCTTCTGC[C/G]GGGGGGAGACAAACAGTCCAGCCAGGCCCTCCGAGTCCCCAGACACCCCCCGCCCCCCTCTTCACTCCATCCCTTGTCCTGTTTTTCCACGTTAACTGGT

16	77701732	SCAFFOLD135011_42228	0.04	0.16	TTCTTTAAAGAGCTCACTAAAGTAAGTCATTTTTTAGTCAGCAGCTCTCAATAGGAAAAAAGGTTACATGATTCCTCATCCTCAGGATGCCAAACAGCTC[A/G]TTTAACTTGAGTCTCCCCAGTGGTCTGACAGCCCGACTGCCCGCTTCAGCAGAATCTAGTCCAATCTGCTGGGGAAATGGTAAAATTCTCTCCACCAGAA
17	1076439	SCAFFOLD125799_3125	0.13	0.24	CTTAGTTTCCCCTCACAGGAATATCAGCTGGCAACTATCAAACATCACTGTGAAAATACCAGAACCTGGGAGTAAGGCTGAGTCAGCCTTCTGGAGCAAT[A/G]AGACTGATGAAGAACTCGTTAGAAGGGTAAATAAAGCAATTTCACTGTGACCATATTGTCCCTCCTCATGACTGGCACAGTATTTCACCAACAGGGTGGT
17	1076512	SCAFFOLD125799_3052	0.14	0.25	AGGGCCCAAGAGCATCCTCCAGCTAAATAGCCACCTCAGGTTTGCTTCCTACCAGAGAAACTATAGACCCAGGACCACCCTGTTGGTGAAATACTGTGCC[A/G]GTCATGAGGAGGGACAATATGGTCACAGTGAAATTGCTTTATTTACCCTTCTAACGAGTTCTTCATCAGTCTTATTGCTCCAGAAGGCTGACTCAGCCTT
17	1076845	SCAFFOLD125799_2719	0.12	0.25	TCAGCAGTTGGGTATTGGTTTAAATTTGCTTCTCTACCCAGGCAGGGACTTAGAATGGACTCCATAGCTGATACGGCCATTGGTTGGGAGCTAAATCAGA[A/C]AATGCTTCCAGCTGAGCTCTCTGGCCAGACAAGGTCACCAGTTTGGCTCTGCAGATAGCCTGAGCTGCTGCCTGGGATCTCTACTCAGACAGCACTGTAA
17	3302271	SCAFFOLD261036_2325	0.21	0.24	AACCACTAGGAATCAGTTACTAGAGCCAGTTGGGGGGAGAAAAGGGAGTGAGATAGAGAGAGAGGTCGGGCAGAGAGTTCTGCCAAAGCCTCCCTTCAGA[A/G]GCTGATGACTGAACCAGATGCTCTGCTCTTCCTCTCTCTGATCTCCTGCTCTGCCTCCTATTGGCTGAGCTCACTGGGGGTCCAGAGAGTGAGGAAGCCT
17	3302287	SCAFFOLD261036_2309	0.21	0.25	TTACTAGAGCCAGTTGGGGGGAGAAAAGGGAGTGAGATAGAGAGAGAGGTCGGGCAGAGAGTTCTGCCAAAGCCTCCCTTCAGAAGCTGATGACTGAACC[A/G]GATGCTCTGCTCTTCCTCTCTCTGATCTCCTGCTCTGCCTCCTATTGGCTGAGCTCACTGGGGGTCCAGAGAGTGAGGAAGCCTCAGGATGTGATCCATA
17	4055580	SCAFFOLD75252_14584	0.10	0.06	GACTGTTTGACGTTGAGTTCAGAGAACATGACCAGGGCTGGTTTCCATCTGTCATGGTGATTAATGGGAGTATTGTTAGGTAATAATAGTTTAATAACAT[A/G]GCTGGAGCCAAAGAGGCACACAAAATGTGTACATGTAATTATAAAACCAAGAGCACTGTGGATAAAACCACAAGAGCCAGCAGATTGAGGTCCTGGTCTC
17	5067743	SCAFFOLD225965_1602	0.46	0.45	GGATTCAAAAAGAGATGGGAATTTTGGATTTCAAATGAAGAGAGCCAGGCTGGCCCTTGGAAAGACAGCCTTCTGGTTCTGCCACTGCTTCTGGAAACTC[A/G]TTTTGTAGGCCAGGTGTACAAGGTCCCAAAGAGGCCACTGAGAGGAAATGAGAGGCTGTAACGATGAAGAGCCAGCACACTAGAAATGAAGTGATTCATG
17	5068172	SCAFFOLD225965_1173	0.46	0.46	CGACTGAAGCGACTTAGCACACACGCATATATATATATATATATATATATATATATATATATAAATAACCTGTCTAAACTCACAAGGGAAGAGAAAGTATTA[C/G]TCTTCATAGGAAGCTCTAGTCCTAACTTGCCTCTTTGTTCTTTAAAAACAGTGACATCATAAGAGGCTGCTGTGCTGTCTTTGGGTGTTCAAGCTTTGGA
17	6604484	SCAFFOLD275031_14464	0.09	0.07	GGGCTCCGGCCTCCAGGGCTTCCTGCCCCGATCCCAGCCCCTTCTGGGTGGGACCTCCAGCCTTTGGGAGGAACAGAATGGATGAAGCCTTTTCGAGGCT[A/G]GAATGATGGAGAGGAAGCTGAATGCTCCTAAGACCAGACTGGGAGCTCTCCACCACCACGGGCATCTCAGAGCAGCAGGTGGCTTGGACGTTCCATTCAA
17	7074360	SCAFFOLD206949_1268	0.49	0.35	TACCTAAGAAACAAATTAACAGGGAGGGAGCTATGTCTTGTTATACTTTTTACTCAAGACTTGAATGAATATATTAGTTTTAAGACTTTAAATCAGAAAG[A/G]TCAGCAGTGATAAAGTTCCTTAGCAGTTCAATTATTTTTGTATATATTTTAATTGTGCTTAACAGAAATTGCTCCTGTTCTGTCTTGTTTGTTATAATTA

17	8073983	BES10_Contig518_1595	0.37	0.32	GGATTATTTTTGTTACACTCTGAATTGCAGCCGTGTGCAATCTTAACTTATTGTATTCAAAATTTCCACTTTATTCTCTTGCTACAACCATTGTAACCCC[A/G]TTGTACTAATGTTCTACTCTTAAATCTCACCGCAGTCACAACAAAGATTCCTGCTCCCCCACCTGCTTCAAACAGGCTTACAGACCATTCCCCTAACTAT
17	9216088	SCAFFOLD10080_18161	0.47	0.25	AGGTAAAAATGTTTTCACAATACTTTGGAATTTTTATAATTGTCCTCTGTATTGTTTGACTGTCAGTTAAATTAGAACGTGTGATAATTGTAGGGAGCTG[A/G]TAAGACTTTCTATTAAAGGTATGTCATTATGTTAATCCTGGTTCAAATATAGTTTGCTCAAATTTTATTTTGATTATATAATATTAGAATAAATTAACCT
17	10648570	SCAFFOLD271783_136	0.27	0.20	GCTAAACGTTACTGCGATCCTGGGAACCACCTGGTCTGTTGAATTTAGGACTAAGACTTACAGCATAATTATATTCTCACATTGTTCCTGTGGAACTTGT[A/G]CTCACTGCTGTCCCAGCCTACTGCGTGGTTAGGACTGAGCAGGCAAGCTTAAGTGAAATGCAGGAAGAAAGTCCTTAACTGAGGCAAAAGTATTGGATAT
17	10681450	SCAFFOLD190171_16889	0.47	0.44	GAAACTTCTCGCCTTAAGCAGGATCAATCCAGGACCTAAAGCTTCAGGAGACCTGGAAGTAGGAGTCCTGGAGGATCAGGGGAAGACCCGGACAGTCCTG[A/G]AAATAGAGACAATCATCCCAGGCCTCTTTACGTTCTCTCAATGAGCTAAAAGTCAGCTGACATTTTCCAGTGACCTAGGCTACTAAGATGAATGTGTGTG
17	12619658	SCAFFOLD120123_18380	0.25	0.19	AAATTAGCATTGCTGTCTCTAATTGGTCTAACTTGTATCATGTGATGTTTCTGAACTAATCACCATCCCTGGTCTGGTTGATCAGACTTGGGTCACATGT[A/C]TACCCTTAGAATAAATTTCACCAGAATCACAAAAGATTGAGGATGAGAAAGAGAAAGAAATCAGAGGCCATTTCCAGAAAATATTGTGATTAGATGTGGA
17	14696059	SCAFFOLD256628_2945	0.01	0.07	TCAGTGGGACTCTCCAGAGGTCCACTGGGTCTGAAGTCACCAGTCTATAGAGCCACCCACACCTGAATACATGGTGAAGGTGACCTGAGCAAGCCAGGCC[A/C]GCTTGCTGGTGTCAGATGACTTATTCTGGGACACCTTATCCATGGCAAAGTGGAGAACGGCGATGAGGGCATTTTATATTAGGCTAATAGTCTTTCCCTG
17	14696477	SCAFFOLD256628_3363	0.17	0.16	AAGAAGTTCTCTCTGTGTTGACCGAAATGAACCTGCCTTATTTCACTGCCTGACTAGCCTCTTCCTGTTATCTTAGATTTGTTGCCTTTCAGACCTGGAC[A/G]GCTGAATGGCCAACCTTATTCCTAAAGGCCTGGCATCTCTGTTTTGCGCAGTGCACAGCAACAACTGAACTAGAGGTACAGCTAGAGACAGGAGGGCCTG
17	19900726	SCAFFOLD110535_1540	0.47	0.38	ACCCAACACAATAAAAAATTAATATATTTTTTTAAAGCACAAGTCTTTATTCTTAAGCATCTTAAATTCCAGTGTGGAGAGAAGAAAATAAGCAAAATAT[A/G]TATTACACATGTGCAGATGGTGATAAAGGCTAAGGAGACAAATGAGCCAAAGGGAGAGAATATTCAGCAAGGAAGAGGGTTGCAATTTCAAACTGGGTGA
17	19901383	SCAFFOLD110535_883	0.30	0.23	CAAGAAAATATCTAGTAGACAAACTGGCACCCATAAAATGGTTTAGACTGGAAGAGAGATTTGAGTGAAGCCAGGGCAGTGTCTAGGATAGTTTAGAGAG[A/T]GTGTGAAGATCTGAAGAGGACTAGGGAGGGATGGCGTCCTGGAAATACCTGCCAGGACTGACTAGAGAGTTACTCTCTCCTTAGAGTTATCCAGGCGGGA
17	20813237	SCAFFOLD35509_12491	0.07	0.10	CTTGCTGTCTGGTTTTGCATCTTTTCATGCATTCTTGATCTCTCCTCTGCGTGGTTATAAGTCCCTCTGGAGCAGGGTGACGACAGTGATACCCCCCTTT[A/G]TAATGTTGCACATAGTGGATGCTCACAGATAGTTTGGTGATAATCTTTTGTAACACTGACTGCAAAAGTGACTTTCAACATGTGAAAAATGAAAATAGCT
17	23373633	SCAFFOLD101118_2815	0.38	0.39	AGCAGCAGCAGCAGCAGCAGCAATGCAATTCATCAGTGACTTAAGTGCCTCCTGAAAGCCCCATATTCTAACGACCCCACCTTAGAAGGTTAGGATTTCA[A/G]TATATGACTTTTGGGGATACACAAACATTCAGACCATAGCAATGATATAAATTGCAGGTGTAAGAGGTTACAAAAGCCTAATGGTTGAAAGAATCCCTAC

17	23757713	SCAFFOLD262092_7301	0.26	0.20	AGTATCTACATATTAATAAGCTCTTTATAGTTCTCTTTAAAAATTTTTTCATATGTTCTATATGATATAATTGGGTTGATAGCTTTGGTAAAATTATTGG[A/C]ATTTTCATAACAAAACATTCCTTTCAGGTAGCCTCTTATTCACCTAAGCTCTCTTCTTCTAAAAGGCAGGTGGTTTCAGGCAGCTGGGATTTGATGACAC
17	23758017	SCAFFOLD262092_7605	0.49	0.43	AAAAATGCCACTATCAAAATAAGCCCAGTTATTAAAAATGTATAATATGATTAAAGTAGGATGATAGGTTTAAAACATCAACCTAGTAACCAAAATGATT[A/T]TTGAATAATTATTAATGAATAACCAAAATCTGCTATTTCTAGTTATAGAATGTCTTTTTTCAGAACTAATGTCCTGATTCATTAAAAAATGACCAGAGAA
17	28276190	BES2_Contig369_543	0.20	0.31	CATTTGGAAGAATTTTTTTTAATACTTAGTAACAGTTTTAGCAATTGCTAGTCTACAGCCCTTTCTGCTATTCACTCCTTCCATCTCTAACATGATTTTC[A/G]TAGTTTGTAACCTGCTTTCAGCATTTAACTGCAAATCCAAGTAGGGGTAGAGAGAGTTCATGTTTGAAAACTTGATAGTTTTTATTCTGACATTTAAGCT
17	28276318	BES2_Contig369_415	0.20	0.30	ACTGCAAATCCAAGTAGGGGTAGAGAGAGTTCATGTTTGAAAACTTGATAGTTTTTATTCTGACATTTAAGCTGATTCTACTTTTATGTAGCACACATAA[A/G]CTTTCTTGCAGAGTAAATCAAGTGTTGCATAGCATTTACAAAATTCTATTTTAAAGCTAGTTCTCTTATTATAGCCTAACATAAAATGCAAGTTAAACTA
17	29424073	SCAFFOLD152367_581	0.01	0.02	TGCAAAAAGTTGGATATGACTTAGGGATTGAATAATAAGAAGAAGTAAGGATGCTTAAGCAAGGACTGGTTGGTATCTGAAACAGCTGGATAGTTACAAA[A/G]ACAGACCTTGAGAGCATGAGGTTGCAGCTTAATATGACCAAGTCACATTGTAGTGACTGAACAGTGACAACAGTCATATAGGAGAATCTACCCACCCCTT
17	29424195	SCAFFOLD152367_459	0.01	0.02	TTGCAGCTTAATATGACCAAGTCACATTGTAGTGACTGAACAGTGACAACAGTCATATAGGAGAATCTACCCACCCCTTTAGTAGTGGACATGGTGATGC[A/G]TGCCCAGAAGTCTCCTACTGGAGAAAAGCTCTCATCTCCCCAGATACTAATGGTGCTGCTGCTGCTGCTAAGTTGTTTCAGTCGTGTCCGACTCCGTGCG
17	29623635	SCAFFOLD105974_12364	0.22	0.08	CTCCTCAAAGTTCTTTGTAAACTATAAAAAAGTTCATATAAAACTATTCTTTGAAAAAAATCAGTAAATCCTAACGTAAGAAATATAAACTTATTTAAAT[A/G]TTTTCTAACACCCAGTGGTTTTCGAGAGTTTAAAAGATAACTTTTTATTGCCATGCTGATAAAATCTGTAATTGTTCAATTTTTAAAAAGCTGTTGTGTG
17	29792256	BES4_Contig230_1042	0.05	0.18	TTTTGAGTTCACATTTATATGAGGGGGATGCTGTGGATCATTGGCATTAATTGATGATGACCTGAGATCAAAGGCAGAGCCCTATGGAACTCCAGCTTTC[A/C]AGGTAGCATAATAAGTCTGAAATGTCAAATGAAATCTTCCAAAAATGAAATTTAAGCTACAATAATGGCCCATCAAAACTGTCAAGCTGGCTATCCAAGT
17	30117855	BES10_Contig428_792	0.21	0.35	CTCAGGCTGCACTTAATTCTGCTATAATGACAGCTGGCCAACATTCTAAGATGAAATTTTCAATAAATGCTGGCTACTAACACGATTCATTATGGTTTCT[A/C]CTGATAAAGGATGTGGGCAAAAATTGCTTTGGCTCTAAAAAAGCTTTCACTTCATTATGAGACTGGATCAATCACCATCAGTAGAAGATGCTCATTGGGC
17	30117923	BES10_Contig428_724	0.21	0.34	CCAAAGCAATTTTTGCCCACATCCTTTATCAGTAGAAACCATAATGAATCGTGTTAGTAGCCAGCATTTATTGAAAATTTCATCTTAGAATGTTGGCCAG[A/C]TGTCATTATAGCAGAATTAAGTGCAGCCTGAGAGCCTTGCTATAATGTACAGAATCTCTCTTCATCACTGTTCTGGAGTTGTTTTGGGTCTGCCTCCAAA
17	30118373	BES10_Contig428_274	0.48	0.10	GAGTTATAAGCTTGTAGTTGGGAACTTCTTCACAGAGTTTGTCATATGTTGCTTTGTCAAACAAGACTAGGTTATTGAGCTTGTCCCAGACTTTACATTT[A/G]GACCAATTGTTTTTGGCCATGCCCCCAGATGGGTTCACTGGATCTTTGTCTTTCTTGGCCAACTTTCTGGCATCTTTCTTCTTCTTGTCATCCTTGGGCG

17	34593955	SCAFFOLD151462_2935	0.22	0.26	AAGTAAAATATTAAGAATATTCTAACATACTTTATTATATTTTCTCTTCTAATATTAAAAATCTGCCTTCATTGAACAGTAGGAAATATCAGGCCATTTG[A/C]AACAGATGAGCACATGATCTCTATGGAGAAAGTTTACAACTTCAATAAAGATGTGTGTGCGTATGTGTGCACGCGCTCAATTGTGTCCAACTCTTCATGA
17	34626712	SCAFFOLD75748_13763	0.24	0.28	CAGCTAAGATGGTGAGTACTTCAGCTCTTTAAATCAGTGGCAGACCATCTTGATTCTGTAAACACATTATTCCCTGTTTTGACTATGTTGTCTGCTTCCT[A/G]TGTTCAACTTCTACTGATGTACGCTCTCTTTCTCACTTAGATCTTTAGGAAATGTCATTGTCTCAGTGAGATCATCATGGGCCACCCTATGTAAAACATC
17	34953337	BES4_Contig331_734	0.20	0.30	ATTGTGGATATTTCAAGTATGCAGATGAGACAATACATCTGGAAAACTGAAGAGAAAGCCCAATCTTTCAATTTGTATAAAATGCTTCTCCAGATTTCCA[A/G]CGTTAACTCTGTATCTGAAAATTAGATGCAGAAAAACGTGTCCATTACAGTTAAATGCTATCAAAGGGATTACATTTATACAGCAGATTATTAGTGCTAT
17	36158296	SCAFFOLD261139_4435	0.10	0.13	AGGAGCCTAGCGGGCTACAGTCCATGGAGTTGCGACAAGTCAGACATGATGGAGCAACTAACACTAACACTTTCCAGATGGTGGGTTGACACATTGGAGG[A/G]GAAGGCGGTTGAGCCCTAAACTGAACCACTTTATGAAGCTTATCCTATATGCCCTTAAACTTTGGGTCATGTGAATTACATGTAAGTAGGATTCAGACTG
17	36428597	SCAFFOLD130884_3966	0.20	0.32	ATAAAACATTGTCACCAATTATTCTGTTCCAGAGAAAAGTCCACATTCTGTGTATTAATTTGTTTTCTACCATAGCCATTTCATATGGGACTCAGAATTA[A/T]GAGAGGAAGTGCATTTATTTTCTCTACTCTAATTGTACATCATCTTGAAAGCCTGAAGGTATGTCGTGTTCAAAATAGCAGTTTGTGTTGTTGTTCAGTA
17	37507731	SCAFFOLD150329_1981	0.10	0.29	CATTTCTGTTTCTCAGAAAAGAAGGGTCCAGCAGGATTTTCAGGACTACCAGAAACTAGTTCACAATTTGAGAATGCAGACGTATTTCTACTGCTGTGTC[A/G]CCATGTGAAGCATGGAAATTTCTGTAAAGCTGTCCATGGCCTATGTAGCTAGAAGCTCATGCATTTATTCCTTACCTGGGTTTTTCTGCTGCAACCATGG
17	37776523	SCAFFOLD220408_18100	0.13	0.12	CAACAGATCATACCTTCTTCAGAGGCCAATTTAATTCTGTTATCAAAGCAGCCCAGACACCCCCGGGCAGGATGATCTGTGTATTGAAACCTCACAGATT[A/C]TGCGAGGGCCATTTCTGAAGTCTGGCACAACACTGAATCATTCCTCATTCTGTCCTGTACTCAAAGCGGGGCAGACCAGATCTGCACAACTCACTGGAGT
17	41375934	SCAFFOLD281900_1804	0.12	0.08	AATAATTGGTTATTTTTTTAAAAAAGAAACTCTCAGGAAGTAAGGAAACAGGAAATGATGTACACAGTTTTGGAGAGGCTGGACTTTCTTCCAGGTCAAT[A/G]AGGATAGATTATCATTCCAGCATCCAGATAATGAGACATAGGAGGGTGAGGAGCCAGAGCAGAAGCCTTTGTAATAGTTCTGGGGGAAGTGACGGGCTGG
17	43048422	SCAFFOLD306734_7254	0.11	0.14	GCAGCCTGTGACGTTTGCTATTATAAGCAGATTTAATAGAAAAGAAAAGAAGAAAATGCGAACCAGAAGTCTAAAAGCTATAAGTCTATGATCCAAATCC[A/G]CTTTGCTTTGGGGAACAATTAATCAGTTAGAATGTAAGGGTCTTTGACGATGAGTTCAGAGCTGCATGGAAGTACATTAACCCTTCATTAAATTCCAGTT
17	44213230	SCAFFOLD260395_9150	0.34	0.20	ATGAACTGTTTATGATCCTCTTTGCTAGCAAGAATGGAAAATAACAAATTCCTCAGATCAGAAGCCATACACAGAGCACCTGTTATTCTACTCTAGCAAA[A/G]GCAATCACATCTGGACGAGCAGCTGCAACTTGGATGACGACGTGGTGGACTTTCCAGTAGTCGGCTTCATCTTCCCTCCAGGCAGCAGCCAGCCAGCCCT
17	44684844	BES5_Contig520_1140	0.45	0.39	ATGAGTTTGAAGTCCTAGCAGTAGGAATCATAAATTTCAAATAACATATGCTAAATTTAAAGTCCTTCTTTAGCCCAGAGTAGACTGCATTCATGTCTTT[C/G]CGTTTCTTTATTTTCCCTTTCTTCCTCCAATTCAGTGTATTCATTCATATCTTCCTACTACAGAAAAGAAAAACTCATTTTTTTTTAAGTCAGGCTTAAC

17	49348120	BES2_Contig398_735	0.04	0.03	AGAAGTGACTGTTCACAATGTTTTTTTAAAGCTGATTAAAGATATCCTCTTTTTTTTTTTTTTCTATAAAAATAATATCTCTGTCCAGGCTCGAACGGTA[A/G]AGGTCATCTCTAGGATGGCTTCATCCTATGCATTTTGTAAAGGGAGTTCGCTTTGTGAGCAGTTCTGATTTAAAATAAATACCAGTCCTGCAAAACTTCC
17	54312135	BES3_Contig407_1134	0.13	0.18	TTTCTGTGGATTCTAGAATGAAGGGAACGATGATTCCTTGAGGTGCAGTGGAAAGAAAGACCTATCCCTTGTCACAGATTATTTCGTTTGAATTTAGTCC[A/G]GACTTACATGCTTTGATGGAATTGTGAATACATGGTGGTGTTTTGTGGTTTGTTTGCTTAATTCACCACTAATTATCTTTTGAACTTTTTTAGAAAATGG
17	55282217	SCAFFOLD170267_2930	0.22	0.24	CAGCTCATTCAGTCTAGGCCACAGAGAATCCCCACAAATAATTGTCCAAGGGCAACGAACAATATGGTTGAAGAAAACCTAGCTCCCAAGGGAACAGAGC[A/G]CTATGAGCAAGAGCCGGCAGAAGCAGACTCACAGAAACCTGGGGGACTGGATTATCCAGATTCTAAGGCGCTCATGCTTATTGACCCTGGTTAAAATTCC
17	55750037	SCAFFOLD55194_30265	0.30	0.38	CTGTGTGCCTGCCTGGGCATGCACATATGTCTGTATATGTGTATGGATATGCACACTTATGTCAGTACATGCACATGTGCGTATGTACATGTGGTGCCAC[A/G]TGTATACTTTTTCTGCATGTTGCTACCCTCAAGTAGCAGAACTGCTCCAGAAATCTGCCTGAGACAGCAAACAGGGTTCAGAATTTTTTTTAATATATT
17	55750526	SCAFFOLD55194_29776	0.24	0.29	TGTGTTAGACATATTGGCATCTAATAGTAGGTATAATGTAGAAGGGGATAAACTGAAAATAAACCCGTATACATTTCATACTGCTCACAAAAAAATAAAA[A/C]GAATTAATTGGAATTTGAGGCTTTAAGAAGCCAAGGGAATGTAGTATTACTATGCACTGCAGGAACTTTATTTGAGCTGAAGACTGGCTCCCGCTTGAAG
17	57681272	SCAFFOLD150220_9590	0.31	0.20	GAAAATAATGCATTTTCCTTGAGAAAATGCACTCAAGTGTTTGACACCTGACCTAACTCATGGGAGCATGTGCTAATTTTATCATTAATCAGAGAATTCT[A/G]TCCATTTCTGTATGTATTGCTGTAGAGTGGACTTCACTCCAAGTTGTCTGCACAATTATTCAGTTCTGTGTTACCTGAGAGGCTTCCCTGGTGGCTCAGA
17	57684765	SCAFFOLD150220_6097	0.28	0.22	AGTGACTCCTTTGGCAACAGGTGTGCAAAGAGAGGCTGGGAGTGGCTCCAATTAGCTAGGACACGGCACAGCCAGATACAGACCCCAGCTTGTTGGACTC[A/C]GTCACCTGTGCCTCTAACCCAGAAGATAAAGAAGGCAGTAACGTGTGGAAAGGCTGTGGCACACAGCAGGAGCTCCATAAGTACTGGCGCTTGTTCTGGA
17	57813556	BES1_Contig394_854	0.08	0.18	TGGCCTTGTGCGAACGTTGTAAGCTTTCCGCCTGCAGAGGAATGGGCCGGCGTCTGGGCCGAGCTGGGAAGAGTGATCGCTTCAGCGGCATGGCGGTAAC[A/G]TTCCCGCACATTTAACAACAATTAGTCTGTGCTGATGCCATGGTAGGCTTTCGTGTATGAAAATTTACAAGCCTTTTAATGGACTGCATTATGGAAGGCC
17	58290353	SCAFFOLD200391_3673	0.34	0.43	CACACCTTCATTTCCTCATCTGTTGAGCAGGAATCACTGTAATACCCCTCACAGAGGGGCACATGAGAGGCTTCCAGGTGTTGCCTTGAGCCTCACGGAG[A/C]GAGCATGCAAGGCAGTGACCATAATGGTAGTGCGTGGGTGACACAAGAAGAACCGGGCAGTCTAGGTCCACTAGGGGTCACTTTCTTAGGCTCAAATTTG
17	59162058	SCAFFOLD281013_11223	0.16	0.17	GCCCCGCCACATCTCACCCCCACGTGGCTTTTCCCAACCCAGGGTGTGGTCAGCTTTCAGCCCTTGTTGCCAGAATGGTCTCTACTCAGTTTGGTCTCAC[A/G]GATTATGTCCACCACCTGTCCAAGCATGCCCACTGATGCTGTCTGAGTTTGTGCCCAGTACAGGTGTGAAAGTGAAAGTCGCTCAGTGGTGTCTGACTCT
17	59607794	SCAFFOLD121139_3191	0.30	0.27	GCCTTGGCTCAGATACAATTTATACCATTGATACTGTATAGCCTGGGGACAATTATAAAAATAATCTCATGTGATTTTTGTGGGTTTTAAATGAGATACT[A/G]TACATCTTTTAGCTCTTGATGGATACTAGGTGTTTGTATTATATTTGTCAGAGTTCTCTAGATAGATAGATAGATGTGTGTATGCTTAGTCATTCAGTCG

17	59609660	SCAFFOLD121139_5057	0.42	0.33	TAAATCAGTTACAGGCACGTATTTTTTTTTTTGAAATTGCATTTTCCCTTTATGTTCACATTCTAAGCACCTAAAAATCAGAGAGAGACAGAATACTTGT[A/G]TTGGATGAGGGTTGCAATTTCCTACAGAGAGATGGCAGGGGAGCAGAGTTGACAGCTAGGTCTGCAAAGGCAAACAGTTTGGAGCAAGGAAGAAAGAGGA
17	59609774	SCAFFOLD121139_5171	0.32	0.27	CTCTCTCTGATTTTTAGGTGCTTAGAATGTGAACATAAAGGGAAAATGCAATTTCAAAAAAAAAAATACGTGCCTGTAACTGATTTATTAATAGTCCATA[A/G]TGCTGGTAATAGGCAATCTATAAGTCTGTTCTTAAGAATTTATAGGGCAAATATTCCAGCACCGTATTAAAAACACCACTCATAAATGCCTGCAAGCTTC
17	62120771	SCAFFOLD150802_4132	0.49	0.45	AGAATAGTGAAGGATGGGAAAGCCTGGTGTGATGCGGTTCATGAGGTCGCAAAGAGTTGGAAACAATTGAGCACATGGGAGCATGCAAACAAGCATGAGT[A/G]CTCTCTGAAGGCTCCAGGGAGGATCCTTCCTTCCTCTTCCAGCTCTGGGTGTTTCTTGGTTTGTGGCTGCATCCCTCCAATATCTGCCTCTGTCTTCCCA
17	62120885	SCAFFOLD150802_4018	0.26	0.18	CCAGGGAGGATCCTTCCTTCCTCTTCCAGCTCTGGGTGTTTCTTGGTTTGTGGCTGCATCCCTCCAATATCTGCCTCTGTCTTCCCATGGCCTCTTTTGT[C/G]TGTGTCAAATCTCCTTTCCCGTCCTCTTCTGAGGACATGCTTATTGGATTTAGGACCATCCCTAATCCAGGATGATCTTTCTTGATCCTTGTCTTAATTA
17	63033323	SCAFFOLD132028_9406	0.50	0.33	GTTGCCATGGGAACAAGGTCACTGCTATCAGCCCCTGGCTCAGGAATTACTCAGGGCTCCCCTTCCCTCATCCCATGTGTCCCCTGGACATCCCAAGCTC[A/G]TTCTGATCTCAGGACTTTTCCTTCTGCCACCCTCACTGCCTGCCATCCTCTTCCTAGCTCTTGCATGCGTGTGCTCCGTCGCTCAGTTGTGTCCGACTCT
17	64500048	SCAFFOLD130095_6623	0.12	0.23	GGTTTCCTCGACTGCGGATTGTGATCATCATGAAGGCCCCCTTACACGATGGGGGCTGAAAGGGCATGGTGGGTGTGAAATACCTAGCGTAGTGCCTAGG[A/G]TTAAGCCTTGCATATGTGTTAGATAGGTGCTAAGTCTGGAGGGGAAAGAAGCGGGAAGGGGACTGACTATTCCCCATGGACAGCGGAGCCTGGGGGGCTA
17	65363913	SCAFFOLD311721_12856	0.35	0.29	AGGCACTTGAACCATTTTTCCCAGTCACTGGAACATGCCTTTCCCAAGGCTGATGCTTCATCATTCAGGTCAGATGGCGCCACCTGAGAGGTCCTCTGGT[A/G]ACCCGCCTGTCATATACCCTGACTTATATTTATCAGTCAGGAATTAATTACAAACTAATATTTACCTTTATTGTCTCTCCCCCCACTTCAGCCACTAACT
17	65586507	SCAFFOLD121344_2150	0.44	0.41	CTCGGACGCGTCCATGCGGCGCCAGGGCGAGCCCAGCTGGAATGCGGCCCGGGCAGCCTTCACTGCTCTGTCCACGTCTGCCTGGAAAACACCCACCCAG[A/G]CACCTCAGGCCTTCTTAGCAGTAACCTCCTCCAAATACCTCCTGACCCACTGCCAAACGCACCCACAGGGGCTCCAGTCGATACCGATGGAGACACTGCC
17	66954281	SCAFFOLD235937_19387	0.27	0.28	TGGTCGACGTGCCCTTCGCGTCCTTCTTCCTGAGCCAGCTGCTCGGGCACCACCACAGCGTCTTCTACAGCTCTGTGGATGAGCTGCCCTCTCTGGACTC[A/G]GAGTTCTACAAAAACCTCACATCCATCAAGGTCAGTGTGGAAGGCAGAATGGCCTGGCTGAGTACCCGTAGGGACTCAGGGAAGGGATGCCCACAGGCAG
17	70689066	SCAFFOLD130230_7734	0.13	0.08	AAAGGTGAAAGCATAGGTAAAATTAAAGACTGAGAAAAAGGCTTCTCTCTTTTTGTGCCTCTTCTCCACAACATTTTCCAACTGAGAGATCTAAGGCCCA[A/G]AGAGGGCAAGCAACTCAGCCAAAGTCACACAGCATAGCTGCACAGCAAGCTGAATTTCGGCTTCCATCTTCCCTTACTACCTCTTGACTCCTCACTTGGA
17	72629663	SCAFFOLD201176_11207	0.11	0.11	TCTACTCTCTCAGGAAACCCCATTGCAGAGGTTCGAGGGGCTCTGCGACCTGCTGAGTAACTCCACAAGATTCTACGTGTGCAGCTGAACCCGTGGGAAC[A/G]GGGCCTGAGGGGACGCACCCCACTGTTCACCGGGGTTACCTCTGCGCAAGGGACGAGGTTAAGGGCAGTGTAAGAGGAATACATGTTAAGTGCTTTATTT

17	72633906	SCAFFOLD201176_15817	0.37	0.38	TTCATCGCATCCAAAATAATGCACTACCAGCACCTGCCTAGTTGCTGGGCTTTCTTGCTGGTTTCTAAGCATGGTGGTATCTAGAATAAGCTGGCTTCAG[A/G]AAGGCAAAAAAGATCTAAATAATTGTTTAAATAGCCAGTTTCCTTTCAACTTCTAACTATTCTGTTGCAACTGTTTTCCCTTTGAGCAGGTGTTTTGTG
17	72666312	SCAFFOLD85606_7256	0.15	0.13	TCTTTACTTGGGTTCTGTGTCCTATTTCTCAAGTAGAGTTTGGTTTTAAGAGTTCTTATCATTTAAGAATTTTTAAATTTCAAGTATGTCTCTTTTCATC[A/G]TTTCTGATCCATGACCAAATTATGGTCTGAAGTATTTCAGATACAAAACAGAAATGGTAACCAAAAGGATGTGAGAAGTAAGAAGAAAATAGGAGCTGAA
17	72956333	SCAFFOLD35329_4916	0.11	0.11	GGATCTCCCTGCATTGCAGGCAGATTCTTTACCATCTGAACCACCAGGCCTTGGGTTGATTTTTAATCCCTTTGACCCTTACTTTTTCCCTCTGTAAGAT[A/G]GGGATAATTCCAGGCCCTGAGAGGTCTGTGAGGATTCAACGAACCCAGGGTCCGGCGCTTGGCCAGTGCCTATTAAATCTTAACAGATACTAACTAGCGC
17	75657503	SCAFFOLD36904_7228	0.14	0.15	CGAGCCAGATGCACTCAGATGCCCCACGGTCACGAGGCACGCAGGACTGGCAGATTCACAGAACAGAAGGTGGGCCAGAGGCTGCTGGGGCAAAGGACAC[A/G]GCGGGGCGCTCCTGCCTAACGGTCACAGTTTCTGTCCAGTGCGATGAAAGGTGTCGGGAACAGACGGTGGTGAGGGCTGCTCGGCACTGCACGGTTAATG
18	1152821	SCAFFOLD186240_12002	0.43	0.50	ACCAGAGATGGAGGCAGGAAGTACAGACCAGATGGCCGACTTGATGCCCACAATACCAGGTCCTGCCTGTCAATAATCAAGTTGCCTCGCATTAATTAGT[A/G]TCTACCCTGCCCCTGTCAATGTCTTATCAGACAACCTTAAATACAATCCTTATCATCTTGTACTTGGCAGCAAAGATTTAATATAGTGAACTCTGCTCAA
18	1153028	SCAFFOLD186240_11795	0.48	0.43	GAACAAGGTTGGAAGATAAACCAAGGTTACCTTGATGTGTATTTGTGGATATGTAGTAACATCCACAAAACATGAGTTTTATGCTAGAATTAATGTTCAT[A/G]ACCTTGGGGGAATGCAAAAAAAAAAAGCCTTAGGAGAGTCTCTCAAAGAGTAGGCCTTTTGGTAGAATTCCAAAAGGCCGTCTACAAAATGCTTGACTGG
18	1153220	SCAFFOLD186240_11603	0.45	0.41	CTTGACTGGGAATGCTGTCTAGCGGGGTCTTAGGTTCTGTCTCTTACAGGTATGTGTTGAGACGGATTCTCCGCCGGGCTGTTCGATACTCTCACGAGAA[A/G]CTCAACGCCAGCAGGGGTTTCTTTGCTACGTTAGTAGACGTTGTTGTCCAGTCTCTGGTAGGTGTATTTCCTGCTCACCTCTTCCGTACTAAAGTGCATA
18	1153305	SCAFFOLD186240_11518	0.44	0.48	GGCCAATAAAGACAAGGATTATATCATGACTCCTCTAAAAATTCTCATTAACAGTGTGACATCGCCCACCCTACCCCAATTAAGGTATGCACTTTAGTAC[A/G]GAAGAGGTGAGCAGGAAATACACCTACCAGAGACTGGACAACAACGTCTACTAACGTAGCAAAGAAACCCCTGCTGGCGTTGAGTTTCTCGTGAGAGTAT
18	1153379	SCAFFOLD186240_11444	0.22	0.26	TAGGTGTATTTCCTGCTCACCTCTTCCGTACTAAAGTGCATACCTTAATTGGGGTAGGGTGGGCGATGTCACACTGTTAATGAGAATTTTTAGAGGAGTC[A/G]TGATATAATCCTTGTCTTTATTGGCCTAAAATCTGTGTTTCCCATTTGCCAGGGAGATGCGTTTCCTGAGCTGAAGAAGGACCCAGATATGGTGAAGGAC
18	2280203	SCAFFOLD210495_5591	0.06	0.15	TCTTTTCTTTCCCCTCGAACAGCAGGGAGATAGGTCTGTTTCCGTGTCTGCCCTGCTATGGAGTATAGTGAAAGCGATAGAAAATTTAGAAACAAAGGGA[C/G]CCGGTTTGGTGTTAACCCGGGACCAAGTGTCTAGGACCACGGCCACATTTGTTTTCTGTTTGCCTCCAACGTACGGTGTGCTGTGATTGATTAGACTTAA
18	3441708	SCAFFOLD305410_14210	0.07	0.12	TTAATTCTGATTAAAACTAAATCTTTGGACTGTTCACCGGGTCTAAACATTTTCCTAATGATTACTGGCTATTTTTGAGAAATGAAAAGAATGGTCTTCA[A/T]TGGTGATGGAGTTCTACAAAATCAGTCAGGTACATCATCTATCTCCTTGCAAATACATAGAATTCCAGGTCATCCAGGGGTTAGAATTCCATGTTCTCAC

18	4020525	SCAFFOLD30203_14583	0.49	0.48	CATCCCCTTTGACTTCTATCCCCTGTGACTCTATCCCCTTTGTCGTATTACTGCGATGGTGAATGTGCAGCAACGTTTTTGTTATGGATCTCACGGGTGG[A/G]GAGTGGGCGCCCTGCTGATTTCTGGTGGAACTGCTCCCAACACAGCTGAGTTGAATCTACTGGGCACCATCACGGGAGCAGAGCTGGAAGCCCTCAGGAG
18	4900355	SCAFFOLD295271_7339	0.17	0.29	TTAACTGAGTATCTGCTGGTACCGATGGGTGTCTGGATCCACACCTTTGTCCTATGCTCCATCCTTGGGTGCAGCTTGGTCCCTTCCCCAGGCCAACCAG[A/C]GGGGCAACCACTGGCTACGTTACTTCTGGTAATCAGGAAGGAATCCCCAGTGCAACCAAGGGTAGTGTGTGGAGCACAGCTTACCTGTCTATCAGGTGGG
18	5552328	SCAFFOLD286127_5518	0.26	0.33	AAGTCAATTTAATAGTCTAGTCTGCAAACAATCCTCTCCCTCCGTGTCCCAGGCGCTCCCCTTCTCTCCCCTTCTTGGCTCTGAGCCGGGTCCCCTCTCC[A/G]CAGCTGTGCTCACTTGAATGGTAAATCAAGCACCCTAAAAACCTGGTGGGTGGAAGGCTTTTCAGCACCGCCCGGGCCCTGTTTATCCCAGGGTGGCAGA
18	5552375	SCAFFOLD286127_5565	0.20	0.25	ATAATACTCCGGGCGTCTCTACAGGAGAAAGAAGAAACGAGCCGAACTCTGCCACCCTGGGATAAACAGGGCCCGGGCGGTGCTGAAAAGCCTTCCACCC[A/G]CCAGGTTTTTAGGGTGCTTGATTTACCATTCAAGTGAGCACAGCTGTGGAGAGGGGACCCGGCTCAGAGCCAAGAAGGGGAGAGAAGGGGAGCGCCTGGG
18	7209264	SCAFFOLD110018_29027	0.47	0.45	CTGTCTTCCCAGCACTTGCTACTTGCTCATTTTGATAATGGCCGTCCTAATGGGTGTGAAGTGGCGTCTTGGTGGTGATTCTGGTTTGCATTTAGAGAGA[C/G]AGGGTGGAATCATGGGCACCCCAAGGCATGATGATGGGGGCAGAGGGTGGGGCAAGGGGGGTGCCTGGCGCTGAATGAGGAGTGACCGTGTCATGGGGGC
18	7459380	SCAFFOLD11117_4437	0.16	0.29	GATTCCTCAGCTTAGGAAAGCTCATTCATTTTGTACCCATGAATTCCCAGAAGAGTTAACCCATTTTATGGCATATCTGGCCATCTTTTGGCCTACCCCC[A/G]TCATTTCAACTAGTCCAAGAAGGCAAAACCAGAGAAGGGTGGGGATTGAGAGAGGAGAGATGGGAGAAGCATTTACCACTGGCCACCCCGTAATTGGGAT
18	8413797	BES10_Contig401_1067	0.50	0.47	TCCCAAACCCTTCCTGCCATGTCTAGCTCCAAGGTCACCAAAAGGTCTGGCTTCAGGCAGTGGGGAACTGAGAACCCTCCCTAGGTTCACTCCGGCGGTA[C/G]CACAGCCCACAGTCTCTCGATTATTAATTGTTATTAACTTGCTCGGCTAATGTCCGGCTAATGATCTTTCTGGAGCACTTTAAGCCCAGGATATGGCTTT
18	8552751	BES10_Contig586_1594	0.35	0.43	TATTTGGATGTGAGGAAAATAAAAGGCTTTAGAACAACTGAAGGAAAGACGTATCGGAGTGAGCGGTGAATAAAATATTATGGAAATAAAAGGAGCGGAG[A/G]TGACCATTAGTAACAGTTAAAACAGAAAAAGATAAGTCATTGAGAATTGAGAAGAAAGATGAAAAAAGATAAAAGGAAATATTACTGTCTGAACACAGAC
18	9192211	SCAFFOLD50006_60621	0.27	0.28	AAAGCACATTGAACCGTATCAGCTAAGCTCAGAACAGGAAAGGAGACTCGTTGATAACTCCATGACACGTGCACACACTCATTATGCTCAGAAACCTCAC[A/G]GATGAAGTGCAAACAAGTAATCATTGGGTTTGGAAAGACCCAGGATGAAGGATCAAATTTGGGTTCTGTTGCTCCTCCTAGGAGATTAGGGAATCTTCTC
18	9315027	SCAFFOLD10898_5735	0.33	0.49	GTGGAGAGACTGAACTAGTGTTTCCCAAACTGGGTACCCTATGGTTCTGGCCCCATAATCACCAGCAGAATTAAAGATACAGATTCTCAGGTCCTGACAC[A/G]GGAGTTTGGTCCTGACACTGAGTTGGTAGGTATGCTGCTGCTGCTGCTGCTAAGTCGCTTCAGTCGTGTACGACTCTGTGTGACCCCATAGACGGCAGCC
18	9510832	SCAFFOLD282025_3342	0.43	0.43	AGCGGTGCAGGGCAGTGGACACTGGAGACTGGGGGATGCCACCTCCCAGTTCCAGCCGCCGGGACTGAGCTCACTTGGTGTTGTCAGAGTCGATGGTGTG[A/G]AAGAGATCCTCCAGTTCCCTCTCGTTGAGGACGCCATCTGCAAAGAAGAGCTGGAACTCCTCCAAGGACAACTTCCCGTCATCTGTGAGGGAGAGAAAGG

18	16486961	BES7_Contig430_975	0.50	0.24	GGAGCCTCAGGCTGAGCCCAGAGGCTTGGCCAAGGTTCTTCAAAGATCCATAAGTACTTGATTAAGATTTGTCCCACTTAACCCTCCATGTACATCTTCC[A/C]GTTGGTCTGCTTCACTGGGTTTGCTTTTAGAAGCTGCTTTTAATTGTCTTGAAACAGAATCCCGTTCCAAGCCATCAAGCAACAGAAATAAAGGTGTTTG
18	16487153	BES7_Contig430_1167	0.28	0.38	CCACTGTCTCACAAGAGATCCTTAGCCAGAACAACTCAATTAAGCCACTCCTGGATTCCTGACTCTCAGGAGATGTTATTACACAGAAACAGCTAATGCA[C/G]CCTCATTTGATCTGGGCAGGGATGGGGGGATCTGTAGGGTTGCAATGAAGGTCTTTTGGGGCCTCAGGCCAAGCCCAGAGGAGTTACTTTTGGAGCCTCA
18	16639190	SCAFFOLD205017_9206	0.14	0.13	ACACATGTGGGCAAATGAAAATAGTTTGCTGTTGCAGCAGCTGAGCATAGGGCAGGCAGTGGGGATTTAAAGGGTGATAGGCCATCTAAGTGGACCCCTG[A/G]AATTACCCTATAAGATAGGCACTGTTGTCATCATACTGATATTACAGATGAGGAAAATGAGATCACAGAGGTTTGGTAGCTTGTCCAAGTCCCACAGCTG
18	17996726	SCAFFOLD136864_3774	0.25	0.23	ACAGAAGATGAATGATACACGAACAATTTGTATGCCAGGCAACTGTGTGCTAGATAATTAGACATTAAAGTAAGCATAATAATCTCAGAGATAAAGGATG[A/G]TGTTAGAAGCATGAAGCAGCAACAGGCATTAACAAAAGAAAAGCAATTGGAAATGTTAGATCTGGAAGATATAATGGGTAAAATTAACAAGCTTAGTAGA
18	19611816	SCAFFOLD305172_7104	0.06	0.11	AGTCAGCTGCCCATCTCTTGGCTGCCACAAGATCATCAACACATGGGAGACCTGCCCTCTGCAAAAAGAAGAAAGACATAAGCAAACAAAAATAGCACTT[C/G]CCCATGCTGGGCCCAGCCATTGACTCAAAATGTTCTTGAAGCTCATAATCAAAGTGGCTATTTATATTGCTTATACCCCAGATCTTGTGGTAGGTGCTTT
18	19860432	SCAFFOLD256179_923	0.23	0.25	CCTTCAACCACTTCAGGGTCAGCTGAATTTTCTTCCATTTGGGTGTGACGCTGCCCCAGTCCTCAGTGGTAGGGCAAAGACTGGCTGTGGTGCCCCTACC[A/G]AAGGAAACAGCCCATGACTGAAACAGGAGCAAAACAGGATAGCCATAGCAGGGTGCCCTGACCCAGTAGTGGCACTAAGACTTTTATACCAGCTTGGAGC
18	20708836	BES5_Contig425_820	0.38	0.20	TTTACAAACGTTATACCTACCTGATTCACCCTCTGGTCTTGATGTTCCCAGAGTGCTGGCTTCCCTGTTGCTGTTGTTAAGCTCAGTTCTGATATGGTGT[A/C]TCCCACAGTTACCCCTGGCAAGTTGAGGAGCCAGCGCGAGACATTTCTTACCAGCATATTTCTTGTTTACAAAACTTCTCAATGGCACGCTATCCAGTGG
18	22223347	SCAFFOLD71818_2294	0.15	0.18	TCAACAGCACTTTTTGTTAGTTTCCCCATAGGAGGGGCAGATGGCAGTGGTCTGAAGATTCTTTGGAGTCTCACCTGGGAGGAGAACAGTCATCTCACCT[C/G]GAGCCTTTGCTGTCTCAGGGGACCACGAGTGAGTATCCCCACGGCCTTCTGGCAGATCTGTAGTGCTCTGTCTTCAAATGAGCCTCCATCAAAGGAAGGA
18	22583517	SCAFFOLD82588_4972	0.48	0.06	CAGGAAACCAGGAATGTCCCCTGTGCCTGATAATTTTTGACCCAATTACTGCACTGATTACCACCTGCAGAGATGTGTGCCTATGAGAAAAGGCATTTTC[A/G]TTTATGAGGATATGGGGTTTAAGAAGTACTCTGTATCTGGTGCCTTATTACTGGAAGCATGGTCTGTGGGCCAGCCACATGGGCGTCACCTGAGAGCTTG
18	22737614	SCAFFOLD305581_21897	0.27	0.25	AAGAATGGTAACCATTATCAGTCCTCAGGATACTCTGCTTCCTACAGGACAAAGGGAAAGAGGTTCCATGCTTTCTGTGTCCTTAATAGACCCCTGTGAG[A/G]ACCCTCTCAGCTCAGCCATTGCAAACCTCAAAAGGAAGCTAGGGATGAGGTTAAGTTCATGACTGAGTGTGGATTCCACTTCAGCCTCCGCATCCAAGAG
18	22795118	SCAFFOLD145048_1393	0.44	0.25	TGTTGTGTTTGTGTATCAGGCCCATTAGCCTAGATAAACTGTTGGCCTTCTCTAATGATTAATGACAGAATAGTGCAGTTGTTGCAAAAATAAGTCATGC[A/G]GTGAGGAAAGAACTGAAGATTATATCTAACACCAGGGGATGGCTTATTAATCTCACTTCTGGAGGAATTCCTGAGTAAATCAGTCTCCTTTATTAATGAT

18	22817673	SCAFFOLD145048_23190	0.23	0.47	CCATTTTCCCACATACTCAGAACTTTGCCTGGGCACTAGTAGGTGCCCCACATATGATTGTTAATGAATAGATGAAACAAAGCAAAACAAAGGCTCTAAC[A/G]GTGGACTGCTTTGTGGCCAGGAAAGCAACATGTATGTGGAGGAATGTCATATTGGTGTTTGATTGACTTTTTGCAAGTGGTTTTAGAACAACACAAATGA
18	23197183	SCAFFOLD135093_34368	0.34	0.36	ACATTTTAATGGAGTAGAATCTGTAAAAACACTGAATCACTAAGCTGTACACCTGAAACTAATATAATTTTGTAAACAGATTCAATTTTTAAAATAATCA[A/G]TTAAGCCATGAGAAAGAAGAAACGGGGGAGGCAAGTGGATTACTCAACACCTGCTGATTCATCTGTCTTATTTTTATCTCAGCATACAATTATTAATTCT
18	23239653	SCAFFOLD105248_1992	0.25	0.21	TTTTTCTGAGTCTTTATTGAGATAACACACAGAAGATACTCAGTGTGGTATCTGGAATATAGTAGACGCCCCAGAAAATGTTTGTTGCAGAAACAAATGT[A/G]GTCCAGTCCCGAGTTCTTTGAAGACGTTGTTCTTCCCGCCCCACCATTCCCACCAACATCCACAAGACTGTCTCCATTTCTTCCCTGACAACATTATTTC
18	23239756	SCAFFOLD105248_2095	0.28	0.37	CGCTCAGGAACTCAGTTACTTGGGTATCAGCTACTTGGTGATCTCTCCAAGCCTGAGTTTCCCTATCAGTAAGACAAATTTACAGTGTTGCAACCTCAGG[A/G]TGTTTTTCTGAGTCTTTATTGAGATAACACACAGAAGATACTCAGTGTGGTATCTGGAATATAGTAGACGCCCCAGAAAATGTTTGTTGCAGAAACAAAT
18	23275745	SCAFFOLD235447_22611	0.36	0.25	TTGGATGCGTAAACCTCATTGACCTTGAGGCCAGAAAGTTACCTTTCTCAGCTGACTCACCTAGAAAATGGAGAGCCCATTTCTGCCCCACCTGCCTTGC[A/G]GCGTGGCTCTGGGGAGCCAAGTGAGGCTCAGACACTGGTGTTATTTGGAAAGGAAAGGGCTGTCCAAAGGCCTTAGAGTAATGCTCCAATATCCCCAGAC
18	25348686	SCAFFOLD149767_1770	0.21	0.34	GCCTGGCTGGCCAGCAGTGCACACGTGGCCTCAGTGCCCAGCTGCCACACAGCTCAGTGAGTCCACGGAAGGTACAAGGGTCCTCACACCTGTGGACTTT[A/T]GAAGACAGCAGCTCTGAGGGGAGGTTTTGGAGCCAAAACAGTCTCAGATATCACTGAAAACCACAATTTACATTTGGAGACTGGGTGAGACCCAGAGAAT
18	25622532	SCAFFOLD151378_4382	0.41	0.44	AACCCTCTCCTGGTGTAGAATGGGTCACTCCCCCAGCCTCTGTTAGCTGAGTACCCACTATCTGCCAGCCTGTGCTGACCCAGCCTCCTCACCCCTACAC[A/G]TATGCTAATCCTCATTCCAACCCTGTGAAATAGGCCCTACCATTCGACCCATTTCACAGATAGGGAACCTGAGATCCAAGGAATTAAACGACTTGCCCAT
18	26024683	BES8_Contig374_1433	0.04	0.07	ACGGCCAGGCAGATTGGTCTGAGGGTCATGAAGAAAGGAAGTAAAAATCACCAGCTGTAGTTTCTTTTTGAGGCCCTCTAGGAAAACCATGACCACAGTA[A/C]ACAAGTGACTACAGTTAGGGTCAGCTGTCTACTTTCCCTGTGCGCTACGCTTATGTGAAGTAGAAACCAACACTGGAGACTGGAGAAATAACAGTATCAT
18	26318990	SCAFFOLD270472_16786	0.08	0.13	AAGGAAAAAAAATTACCCACAGCTGTTGCATTGTGGAGTGTAGCTACCCTTTGTATCACATCAGCATGGCATTTGGGAATTTTTTTAATTCACGAGGTTG[A/C]TTTAACCTTCATGTTTGACATACTAAAGGAATTTACTTGGTGACCTCATGGTTTGCCCTGTTCTTTGTAAAAAGATTTTCTAAAAATTTGAAATGTTATA
18	26319049	SCAFFOLD270472_16727	0.08	0.06	TTTAATTTTGAATGTTTCTGTCAGTTAATCTGTCACTTGTGCCCAGGAGCTTAAGTTACAAGGAAAAAAAATTACCCACAGCTGTTGCATTGTGGAGTGT[A/C]GCTACCCTTTGTATCACATCAGCATGGCATTTGGGAATTTTTTTAATTCACGAGGTTGCTTTAACCTTCATGTTTGACATACTAAAGGAATTTACTTGGT
18	28847686	SCAFFOLD155119_7123	0.31	0.35	CTCCGCAGAAGGGTGGAGGCTGTGCTAAACCAGCTCCCCCAGGAAGTTCTCACTGTCATCTCTATTCCACATTCAGCTCTCAGCAATTGTTAAAAATCAC[A/G]TTTAAATGATCTTACCATTTGTATGGCTCTAGCAACTTCTGATCTAGGTAAGCAGATACTAGTTGCTATNNNNNNNNNNNNNNNNNNNNNNNNNNNNNN

18	28847786	SCAFFOLD155119_7023	0.11	0.14	ATCTTACAAGGATTACTCTTCCCATCATGGTGCCAGGACCGCAAGGGAGTCTTCTCTGGTTCTCCACTGTGAGGACCTGGAGCGGTTCCTGGAGATTAAA[A/C]TCCGCAGAAGGGTGGAGGCTGTGCTAAACCAGCTCCCCCAGGAAGTTCTCACTGTCATCTCTATTCCACATTCAGCTCTCAGCAATTGTTAAAAATCACA
18	29396265	SCAFFOLD115008_35565	0.33	0.44	CCCAGGATAGCTGAAAGGGCAAGTCAGATTTGGGAGATGTTCCTGGGAGGGCCAGTTATGCACACCTTTCTGAGTTTCAGCTAGTGTGGAAGGTTCAGCT[A/G]TTTTATTTTTCACCTGCTATTGAAATGCTTATTCTCTGAAAATGTATTGCAAATAAGTCTTTGCAAATGGGGTCTGTGTTCTTCTCATCATAAGGGTTTT
18	29425151	SCAFFOLD167149_2583	0.50	0.46	GATTCAGATCGTATGGTCTTATTATTTCCCTTGCCGTGTGGTCCCCCAAAAGGCTCCTCACAGTGCACTTGCATCAAGTTGACCAGAGCTGTGCGACACC[A/G]TAAGAGCAACTCTTAAGATGGATACTGGCACTTCTTTCATTTAGCTTTGACATTGTAACTTGTTACCTACACACGAATGTGTGAGACATGTTTGATATTA
18	29600313	SCAFFOLD20017_21254	0.36	0.39	ACACAGTGAAATAGGGGCCATATGCAAGCCAAAAAGAGAACTCTCATCGAAAACTGAAGCTTCCAGCACCTTGATTTCAGGCCTCCCACCTTGCAGAACT[A/G]TGAGAAATCAATGATTATATTTTAAGCCACCCAGCTTATGGTATTTTGTTATAGTGGCTTAAGCCTGGACAAGTGACATTAGGCAAAAAGGTAGGTGGGA
18	31238166	SCAFFOLD68226_7882	0.46	0.43	ATCAAAGTTGATTTAAAAAAAAAAAAAAAAAAGAAAGTAAGAAAGAAAAAGCTTCCAGGATCTCACCAAATAAATACATTTGGAAAAGAATCCATCTCTC[A/G]GCTTATGTGAAAATAATGAAAAGATGCACAGTGATGACACATTTCAGGAATAAATTTGAAGGGAATTGTTTCATACAATTTCACAAAGGGACCTTAGGGA
18	31997039	SCAFFOLD130342_21732	0.03	0.14	TTGCTCACCTCTTCTCAACTCTGTGGTCAGTGATGTCTCCTTGGCAGCTTGAAATTGGCCACAGTGGGAATATTTACACTATGGAATTCAGCAAAGGTGG[A/C]AAATCACCCCATCTGCAAGGAGTCAGTTGTTGAATAATTCCAGCACACCACTGCTGTGACCACTTTCTGAACAGAGCCTTGGAATAAATAACCTTCTCTT
18	33054011	SCAFFOLD342982_446	0.49	0.49	TCTGCTTTCCACTGTATTTTTCAGTATATTTTACAACTGATATCTGCTTACATAAAGTTAAGCTTCCACATTTAAAAATATTTCCCCCCAGAAAGCAAGA[A/G]GGAGAGTCTGTTCTTGTGGAGATCTTAAGGGACAGGACAGCTGGGCTGGGACTGGGAGAGTCATACGTGATTTTGTTCTCTTCTTCCACATTTGAGGGCT
18	33899467	BV105480-254-R	0.19	0.18	GTCCCCRTACTCTTAGGATAAGATAAGGTTCCCCAGTGAGGCCTGCCCGGCCCTGCACTGCGTGGTCTGGCACCTCCAGACTCCAGCCGCCTTGTGGTCC[A/G]CTGTGACTTTGACTCTCGGCCTGCCAATGAAATGAACCCTGTTTTAGAAACTCAGGACCCACYATGCTCCCCCTGCCCTGGCCACTGGGACCTCTGCACG
18	33924254	SCAFFOLD153273_2901	0.11	0.19	GGTCACTGCCGATCTGTCCAATGGACACAGACATCAGAATGGGTGGTTGACAGCACTGTGCATATGGGCTGGAGGTGACCCAGGTGCTGCCCATGGCCTG[A/G]TCATAGCAGATCTCCAGGGAGATGATGCTCCTTGGATCTAAAGACTCCTGGATCCACTGCCCACCTTCAAGCCCAGGCTCAGACCTGTGGTACAAAGCCA
18	34483057	BES7_Contig335_1167	0.45	0.37	ATGGATCACCAGAGCCAAAGAATGAGGACCAGGAAGGCAGCAATAACTATGATGCCAGGGGCTGTGTTGGGGGAAGATTTTGAGAATGACCGCATAGTTG[A/G]CAAAATAGGACTCCCATGCCTTGGAGTTCTGGTTAGTTTCTGGTCTTGAGCCAGATTCTCTCTCTGCTGCAGATGCGAGAGGACGGAGCGGCAGGAGCTC
18	35368494	SCAFFOLD170085_24810	0.14	0.17	TCCAGGTACAATTGGTTGCTTGAATTCAGCTCAGACTGGAATTGTGACTGGCCACAATGTAACTTCACCTTCCTATGAACACAGGGAAGCAAGTGTAAAT[C/G]GGTGCAAGAGGAAATCTCGGTTTGGATTACCAATAGTTGCATACTGACCAAAAGTTGAGAGGCATTGGGGCTGGCGGCCATGCTCCAGATGGGCCCTCCA

18	36361028	BES7_Contig368_825	0.46	0.43	CTGTGCTCTTGGTTTTCTTTCTTCCACCAAAAGGCATCCCAGGACCTAACGGAAATTTACTGCTAAGGAGGAGAAGGAAGAAAAGGGGTTGTTGATTTAT[A/G]TTTTACATAATATTCATCTTATGGTTGGGACCCACTGCAGGGAAAATGTAGCATCCCATTTAATCTAGAATCGTGTAAGCCAGTTTGTGGGTGCGTGTGG
18	36403044	SCAFFOLD52080_7447	0.07	0.06	ACTACTGTCAGAGGTAGCTAATAAGGACTTGATTTTTTTTCTTTTTAAAGACAGACTAGTGGAAGACTACCATAAGCTTTGTTATGCTGTCTTTTGCAAA[A/C]GATAAACATTTGCTTTCCATCTTGATGTCATCTCTTCAGGTTATTATTTGGTCACTAATTTATCTCACCATTAGCGAATTACAGTAGATTATTGCTTGGA
18	36952361	SCAFFOLD251179_6739	0.34	0.47	AAATTTACCATCATCACATTATGAGTACAAACATTTAAGGAAAAAACGAAACTATGAATTTTGACATTCCTGAACAGTAAGAAACCAAAAAGACACCATG[A/T]GAGGAGGAGAGATGGCCAAGGTGAAAAGGGAAGGAAAGAAGGCTCTTGTAGCCGGGTTTCTCTTGAGAATGATCATATTCCTCTGGTCTGTCCATCTGTG
18	36952520	SCAFFOLD251179_6898	0.35	0.48	TCTCTTGAGAATGATCATATTCCTCTGGTCTGTCCATCTGTGCAACTCAATGCAGTAGAGGTCAGAGGGGAGCCCAGAGAGGACCCCAAGACAAAATGAC[A/G]TTGGTTACCGGCTTAAGAGACTCAAAGAACTGTCCTAGAAGAGAGCAAATTCTATTCTGAGAATAGGTTTTTCAAGAAGAAGCAGATTTTCCAGTTTCGG
18	38869710	BES9_Contig440_780	0.37	0.42	TTCCACTTATTCCACAGGAAGCTTTTCAGAACGGAAAACTGAATTCTCTACACCTTTGTGAAGCTCCTGGAGCTTTTATAGCTAGTCTCAATCACTATTT[A/G]AAATCCCATCGATTCCCCTGTGATTGGAATTGGGTAGCTAATACTCTGAATCCTTACCATGAGGCAAATGACAATCTTACGATGATTATGGATGACCGAC
18	38869829	BES9_Contig440_661	0.11	0.11	TGTGATTGGAATTGGGTAGCTAATACTCTGAATCCTTACCATGAGGCAAATGACAATCTTACGATGATTATGGATGACCGACTTATTGCAAATACCTTGC[A/G]TTGTTGGTACTTTGGTCCAGATAACACTGGTGATATCATGACCTTGAAATATCTGACTGGACTTCAGAATTTCATAAACAGTATGGGTACCATTCACTTG
18	40524778	SCAFFOLD70087_15055	0.49	0.46	CTTGGAGCAAAAGCCCATATGCCTCCTATCCTTGTTAGAAGATGCAAACCCAGGAAGCAGCAGTGAGGGCCAAGGGAACTGAGGGAAGCGGGAGGCAAAG[A/C]CAACAGGAACTGCACTCTCTAGCTACTAATCTCTGAGTGCAACTGATTACATAGCCTTGGAGGATGGTCATCTGAGAACCATATACAGGGGTGGGATGAC
18	41075131	BES9_Contig355_788	0.46	0.09	CCTCTCCAGGTCACCCTCCCTGACCGGCTGCCTTACTGGGTATCGAGTCTGGTGCCTCACCCAGAGGAGCCGTGGTGAGAAGGGGAAGCTCGGCACACAC[A/G]TGCCTGCTGCCTCTAAAGTCCATGAACATCTTCTTACACATTCAGAAATGAAAGAAAACCGTTAACTTTCTGTGTGCATGCATGAGAGAAAAGCTTTTAA
18	41705003	SCAFFOLD300664_1947	0.33	0.37	TCCCTTCTTGAGTCAGACTGTCAGGCTGGGCCCCCAAATGGGATTCTTACCCCATGCTGCCAGACCCGGCTGCTACAACAGCAACACCTTGGCTCCTAGA[A/G]TTGCTACAACCTTTTGTTGTTGTTCAGTCGCTCAGTCGTGTCCAGCTCTTTGCGACCCCATGGACTGCAGCATGCCAGACTTCCCTGTCCTTCACCATCT
18	43389201	SCAFFOLD26162_6221	0.07	0.14	GATGGATTTGAATTAACCAGGAAGTTCTTTGACTGCTATTTTAAAACTTCTCTCTGCTACCTGGCCTGACATAATAATTACCTGCCTGTGATTTATCTCC[A/G]GCAGATTATGAGCGATGATAGGGTTTGGGGTGATAATTGAGTGTGTCAAACACCTCACCTCCTCCCTCGTCATTCAAGCTGAAACAGCGCTGGTTTTCTC
18	46112016	SCAFFOLD135219_11361	0.07	0.08	ACTGGTGGACGGTCAGTTTGCGGGGTGGCCGAGGGTGGAGATGGCAGTCCAAGAGAGCTGAGAGTGGGGACCCAGGGTAGCGCATATGGGAGTCCGAGGA[A/G]GCTCAGAATGTGGGATTCTAGAAGACAGAGTCGGGGGATCCAGGGGGTTCAGGGCGTGAAAGGAGTCAGACTCAGGTCCGGGGGTGCTGCAGTGAAGGGG

18	47384477	SCAFFOLD260171_17048	0.33	0.32	TAATAAAAAACAAACAAACAAAAAACCCCAGACAGAAGTAGATTATCTGCCAGTTAAAACTTAAAGAGACCTAATCACAAGGGTGCCCCGAACAGGCCTC[A/G]TTCCTTAGCCACAGCTGACCTTTATTCCAGGGGAAGACTTGGACCAAGCCGGGACAAGGGGCTCCTCTATTCTGAATGGAAGGAGCAGGGCTGGGATGTT
18	47861332	SCAFFOLD98325_1119	0.17	0.28	GCACTGGAGGGCCATGCAGACAGACATGGACTCCTGGGACCACCAGAAGGTGCGAATGGCAAGGAGTGAGCAGGAGGAAGTGAGTGTTTGGAGGAATAAC[C/G]CTAACAAAGAGTGTGCCCGTGGAGTGCATTTGAGGGGCAGAGGTGGGGGGCTGGAGCTCCCACAGCAGGTACCAGAGTGTGAAGGACCAGTGACGAGCCA
18	47966036	BV105445-237-S	0.27	0.31	GCCAGGACCCCCGCCTCTGGCCTCTGGCGGCCCCTGGGGCTGACCGTCTACCAGCCCCTTGAGGCCGTTCTCAGAGAGAGGAAGTGGCCTCGTCTTAATC[C/G]CATTTCCCACAGCCTTGGCCTTTCCGGTGACCATGSGGGATGGAGGGGCCRAGGGGCTGGCCAAGCCCACTGGAGTTGCCCAGGGTCATGGAATCGGCTC
18	48063612	BV105352-933-K	0.46	0.49	CCGCCACGGGGCTCTTGCTGGAAATCTCAGCTGCCAAGGTGAAAGCCGCATCAAGCATGCTCTCCTTGTCTGGGAAGAGCCGGCTGAGGATGAAAGGAAT[A/C]GGAAATCAGGCAATCTGAGAAGAGCTCTCCTCTCCCTCCCATGGATGAAGAAGTGACCAATCGCAGTAGAAAGTGGAAGACAGCACTGGTGAAATGGACC
18	48063813	BV105352-732-R	0.46	0.48	GCCTACACTGCCCGCAAGATGATGGCTGACGAGGCCCTGGAGAGTGGACTGGTCAGGTAAGGGCTGAGGCCTGTGCTGGATAAGAATTCAGGGACAGGCA[A/G]ACTTGGGTGGGGCTCCCTGGATTCCCAYGCACCTTAACTCATGGATTCCAAGTGGCCTCCTCTCTGTGGRTGGTCTGGAGTCTACTGCCTTGCTCCGATT
18	53830565	SCAFFOLD140034_9410	0.29	0.28	CCTCTGAACCCTGCCTTCCCACTGATGAGAACAAGGGCTAATAAACCTTCTCAGGGGCATCAAAATATTTTTTTCCCAATAAAATTTTAAACTGCACCTT[A/C]TGGGTGAAGGACCCAGAAGTCCTAATGGCCTGCCCTCTGGTTTCCACACAGAAGCACATCCTTAGAATCCCAGGGAACCAGGACAGCGCGTCTGAAAACC
18	55914460	AY092412-246	0.12	0.09	CGGACCCCTGCTCCCGGGCCGCTCATCCGTGTGTGGGCACCCCAGGCCCCTTCACCATGCAGGGCCTGTTGGGCTGCGAGTTGGGCCCTGACAATGTCTC[A/G]GTGCCGGTGGCCAAGTTTGCCCTGAACGGMGAGGAGTTCATGATGTTTGACCCCAAGCTGGGCATCTGGGATGGTGACTGGCCTGAGTCCCGGACTGTCA
18	55918324	AY092413-314	0.38	0.38	GGGGCTTTCTAGATCTAGAACATCCTGGGAGTGGCCTTCCCTTTACCCACACCCACTCCCCACAACCTGGTGCTGGGATCTCTGGGGTTGGGAGGGACTG[A/C]GAACCCCCCATCCCCTGTCCTGACTCAGATGTGGCGGAGGGTCCCTTAAATATCTCACAACATTGTCTGACTGCAGAATCACCAGCCAGGACCTCGGTGC
18	55918813	AY092413-804	0.40	0.38	ATCCTAAGATTCACCATCCTTGGACAGGCCGGGAGTGGGCAGGAGGGCCCCTACATCCTCCCCACGGAAAGAGATCCAAGGAGCTGAGAAAAGAGGGACA[A/G]GGGAGATCATTAGAAACTTTGAAATCCTCCAAGTCTCTGCCTGCCCCTCCCCACGTGTGTGTGTGTATGTGTGTGTGTGGTGTGTGTTGTTTGTGTGTGT
18	64492300	SCAFFOLD75304_7015	0.35	0.23	GTTCGATCCCTGGTCGGGAAACTGAGGTCCCACATGTCCTGAGCGACTAAGCCCAACTTCCACGACTAAGGAACCCGCAAGCTCCAGCTAAACATTCTTC[A/C]TGCTGCAGCTAAAACCTGAGGCAGCCAGATACATAATAAGCTACAAGGATACATAAGCTTCTTTAAAAAGGACGGCATCTCTCTGGAGTTCATTTTCTTT
18	65715221	SCAFFOLD130265_24210	0.15	0.10	CCAAACACATTGAACCTCACAATGTCCATTCTAAAGGCTTTGTTCCCCACAAAGAGTAAGGAGACTGTTTTATTCAGTTTTTGAGTGGATATTTATGTCA[A/G]TTGTTGGATATGCATTGTTTACCTTTTGGGAGCATCATTTGAAAATGGAATCTGTAGGAGAGTTTTACTAAGATGCCTCTGATGAGTGCCAGTGTTAAAA

19	3367364	SCAFFOLD210001_43773	0.23	0.12	TTAGAATATCACAAGAGGGCTACTGTCAGGAATTTAGAGGTTTGCTGTCAGGGATTTAAAGATTCTACCTCACTAAAAAGCCCATCACTTGCCTTCCTAC[A/G]TCTTTAGTAATCAGAGTTGTGTTTGTGCTTTGTCAGGGCTCAATGACTTCAACAGCAGTGATAAGAGAATGCCAGAAGGTAGATTCAAATGACATTCTGC
19	4173324	SCAFFOLD142102_6580	0.46	0.07	AAACTGGCAACTCATCCAGCAACTGCAGGGGTCACACAGCCAAAGTTGGGGGAAAACGGGAACATATCAAATAAATGTCTACACCCATATCAAGAAACAC[A/G]GTGCACAGAATTGTTTCTATCAACAAAGAACTTAATTCAAATTACATCAGGCAGCAGAAGTTGGCTGGACCCTAGACAGCTGCCAGCACCTCAGAATTCC
19	6355505	SCAFFOLD226442_3035	0.16	0.17	ACACTCACCCGGTGCCTTAGCTAATGAATGGAATTTGGCTAGACTGCCCAGCCCAGCACCAGAGCATTATGAAATCTTCCCTTGATCCATCTAGTTGTGC[A/C]GTTTCTAACCTTTCAGGCCAACCATAGCACAAGAGGAAGAGAGAGGGAGAGAGAAATATGCCTAGAAACCTGGAGAAAGATCAAATATAGTCATTTCTAG
19	6355596	SCAFFOLD226442_2944	0.49	0.44	GTTAGAAACTGCACAACTAGATGGATCAAGGGAAGATTTCATAATGCTCTGGTGCTGGGCTGGGCAGTCTAGCCAAATTCCATTCATTAGCTAAGGCACC[A/G]GGTGAGTGTGGCCAGTAAAAATGTTGTTTTGGTTCCATAGAAGCGCTGATAGGGAATAGGAAATAAACTTTAATTTTGGGGGTCCTCAGAGGCAGTATAG
19	6471345	SCAFFOLD245001_53297	0.14	0.10	AACCTAGCTCAAAACCACTTCTCTGCCAAAAGCGCTCTCCAGGAATTCTTGCCTGTGAATATCTTTCTTACCCAGTTATTTTATGGCATGAACACTCCCC[A/G]TGCCCCATCCTGAAACAAATTGAACTGGGAAAAATTGGAGGTCTGGACCATCTCTCTAGTCTGTCTCCAGGGAGCTGTAAAGAATGTTGGGGACATAGTG
19	7502282	SCAFFOLD321751_22824	0.35	0.43	CTGAGATCTAGCGCTGCGCGCATTGCCATTCATCAGAGGTCTGTGGCAGCAGCCCGATTCTAAACAGAGCAGCTTGTCTTTAGGATGGCCAGCTCTCCAG[A/C]GGAGCCCCCTGGCCTTCAGCAGTGGGGTGAGCACCGCCTCCTCTTAACCTCTCTTAGCTCAGCCGATTCAGCCGAGCGCAGAGAACAGGCCCAAGTGTGC
19	7816104	SCAFFOLD313690_1625	0.22	0.19	GCTGCAGTCACACCAGGGGACAGAGAGAGCTGGAAAGGCAGAGAAAAGCAACCCCAGGGGCTTGAGGCTGTGTCAGCCCTAAACAAGGCCTGGCCAGCTC[A/G]CTGGGCAAGAACAACTTCCAAATGCCAACCAGGGGCTCCTCCTTGAGTCAGAGGACTCAGCCCTGCAAGTCCTTGAGCTTTGCCAGGGAAGGACACTCCT
19	7819211	SCAFFOLD313690_4732	0.23	0.19	ATCTGAATTGAGAGTACTAAATATCTCCGGAAAGTTAGAGGTGTGTTGCTACCTAAGGTTCAAAGCTTTGGGTGTCCTGATAGAAGTGACTGAATGAGCC[A/G]ACATTTTTGAGAAGAACAATGCAAGTAGGAGGCACTTCTGCCTTTTTAACAAAGAGCAGAATGTGAGTTAGTCTTATAAACGGGCCATGTTGAGTTCACG
19	7902990	G73111-168-W	0.46	0.10	CTCCCCACTCCCTGGCCCTGGCCTTGGCCTTCTACCTCCCAGCCAGAGCTMAGGAGGCCTCCCTCATACCAGCCTTATCCCCTGACAATGCCCCAATACC[A/T]CCCACAGGCTCCTTTTATGGAGGTCAACTGAGTAGCCAGGCCCTGACTCTCTCACCCGGCTCTCATCTCTGGGGACAACTTTCTTTCTGTTCTGCTTTGG
19	8717810	SCAFFOLD23408_767	0.08	0.10	AGGTACACAAATAGCCATTTGTGATTGAAACCAATGACTGACTCCTACAAGCCTACGTGCACTCCTGGGCCAAGAATGTGTGAGAATTTATGATTTGAGC[A/G]ACCTGGATTTTCACCCCACGGCCAACCAAACCACCCCCAAAACCTGTAGAAAGCTGGGATTCTATTTTGTGACTTATATTCAGTGCAGCTCCAAACCAAA
19	11009898	BES5_Contig238_570	0.09	0.18	ACACACCAAACTTTTCTCAGCGTCACAGGGGAAAAGGCCTTCTTTCTGGGAAACGGGAAAGGCACTAGTCCGAGTGAGGGCAGAGGGACCTACTCCCCCA[A/C]GGGCCTGAGTTTCCAGATCCAGTGGCGTCGGGCTGCCTCCTACCCCGGGGAGGGGCCTTCTCCTTTCAAGGCTGGGCTTTGCCTGACTCACAGTTCCAAA

19	11385079	SCAFFOLD105007_21421	0.32	0.30	AGGACAGTACTCTCATAGCTCATTAACTTTCCAAATAAGGCCTTAGGGTGTAGAAGAGGGGTACTGAAAGCTTTCCACATGAGTAAGGACCTTTACCTTG[A/G]GGAGAAGTTTGTTTCCATGTTAGAATCACACGTACCAATGAGCACTACTGTCCTCACAATCTGAAGAATATTACAAGAATACTCACAGAACTAATCTGGA
19	12005510	BES1_Contig631_717	0.48	0.41	TTAAAATTCATGGTCTGAAACCGCAGTTACATTTGCACCAACCTAACAATAGGACAGACAAGCAAAAACAAAGATTAACCGTTATATTCTGCTATAATTA[A/G]AAAAAAGCAAAAGGAGAACCTGTAAGAAGGATTTATGCTCTGGACCCACAATAAAAGACAAAAAACAAGAAAGAACTTTACTTTCCAGTGAGTGACACCT
19	12199133	SCAFFOLD110615_4785	0.46	0.46	GGGTGACAGCTCCCCAGCAGCCCTTGGCAGCCACCAGTCCGGATGGAGGAGACATGAGAGCGCTAGCTAGGGCATCACCAGGGGAGAAAAAGCACCTGAT[C/G]TACTGCCAGCTGGGCAGGTGTTTCCCAGTTCTGGTGACAAGTTTGATAATAAAGAACAGGTATGTAAAAACTATACAAGTGAATATATGTGGGAGTTATT
19	12259028	SCAFFOLD101904_18157	0.36	0.10	CCTCCCGCCGCTGTCACCGCTCAGAGCTCTCGCCTCTGGTGGCCATCCTCACAGCCTGACCTCTCAGGTCAAAGTCCAGGGGTGAGAGCAGTCCCTGAGT[A/G]AGGGTGGGGGAGGCTGAGATCATCAGTGGAGGAACCTCCCAAATAAACTCGAGTCTTCCGTTCAAGAAGGGAGAAGTTTCTGGGGCTCTGGGGTGAGGTG
19	13241459	SCAFFOLD85112_22280	0.39	0.43	ACTCTGTAAGTTTAAAAAAAAAAAAGCCTTTATTATTTAGCTGTTAAATAATTATAATGCAATCTGCTGCAAACACCCTCTTGGAATCAGTGTGCCCCAG[A/G]AGTATATTAGCATTATTTTTAATAAATACATCTGCTTAACATATTGGAATTTTTACTGAGAATTTCGGGGGGAGGGGGTGTTTGTGTTGATGGACAAAAG
19	14478666	SCAFFOLD276848_2797	0.32	0.48	TTCTTTCTTGTCAAGTGAACACACAGGGGAAGCAAGGACTTCATCTTTTGTTTTCTTTGTCTAGCACCACTGCCAGTTCTAGCCTGTCAGGGCAAATTCA[A/G]TAATAATTAATAGCTGTTGATGAGCCAGTTGTTCTAGAATCCATTTTTTTTTTTAATAAGCTGACCAATAAATGAGACTTGCCATAAATTTAAGGATTCT
19	14626504	BES10_Contig468_537	0.48	0.38	AATGAATGGGGGTATACCAGCCCTTAGTGCCCAGGTGCCAAGAATGTGCAACATTCCACAGTGGAGAGAACAATACCACAATAAACAGTGCCTCCCAGAC[A/G]TCAACAGCACTGCCACTAACAAACACAGCACGGCAAACTGGCTCTTGTACGCAGCTCCAGGGAGGTAGGAGGAGCAACGGGAAAGAGCCTCTGAAGCTCA
19	24489943	SCAFFOLD321708_25778	0.06	0.11	CCCTGATCTTAACCAAATATCCCTGCTTGTATGGATATTAGAGAAAATGCACGGAATAACACTCTAGTGCAGATCATTTTAGACTGCTGGGCTTGGCTGA[A/G]TGGAACCAAAATCTAACTGGGCCTAAACAGTTTGACCAGTTTATTACAGATCGATCCCTGAGTTGGGAAGATCCCCAGGAGAAGGGAAAGGCTACCCACT
19	31862191	SCAFFOLD151511_3373	0.35	0.37	TTAAAAGAATTCGTATTTAGACAGAGGGCATGTTGCATATGCAGGACTTGAAGAAAGTATAGAGCAACGAGAGTAAAAGCAGCGCTATAGGTCTGATGGA[A/G]AGATGGCTCTCAGGGTTACCAAGGGATGCTGATTGCTAAGTAGGGTTCAGAGGATTCTACTGGGAGCCAAGAGCCCACTGTAAGAAGGTGATACAGAAAG
19	32341595	SCAFFOLD37470_667	0.36	0.37	CTCTGGTAAGTAACGGAACTCCTCTGTGATGACGGCAGGCCACTCTTCCCTAGTGTATTGGCTTTGCCTACAATGGAGCATCGCTGACGGTCTTCTTTTT[A/G]TACCCAAGTTGGTCATTTGGCTTTGTCTCTGATGGGGCGTTGGCTGGAAGGCTAGGGAACTGTGACCCTGACAGATGGTTTTGCATTGCATTGGTTTGCT
19	33263855	SCAFFOLD315630_442	0.40	0.43	TTTCTCCATATAATGAGGTTTGAGTGATTTTCAATTTTCTTCCTTTTGCTTCTTTTTTTAAAAAATGTTTCCAGAATGAACTTGTGTGACTTTTGTAATC[A/G]AAAGAATAACAGTTGTGTGGGGGGAAAAAAAAGTATAGACTAGTGTATGTTACAGGAAATACCTGGTTTTCAGCACCTAAAATTAATAGTTTTCCAGCAT

19	36775177	SCAFFOLD300078_3267	0.38	0.47	TTTGGAACCAGCCTTGAGAGGGCTGGGGAAGGCAGGGGTGAGGTTTAAGGGAATATTTGCACCTGTCTCTGGATCACAGAAGAGGCTGAGGGTGTGTGGC[A/G]TAGAGAATTGGCCCTGAGGCCTTTCTCAGAATTCCCTGTACAGTTATTCAAATACTCCTTTTGCTCAAGTTTCCTGGGGATGCCTAGGCTGTGGGGCTGA
19	37366165	SCAFFOLD35382_7020	0.14	0.20	TGACACAGTGTGCGACTAGGAGGAGAATCAGACCCCACGGGACCTGGGTCCCTGAGGTCCTCACCCTTCCGCTGAGTCCAAAGGCCTCATCCCAAGCCCC[A/G]AGGATTGTGAGGATCATGCTGCACTCTTAAACCCTAAAAGCCAGATCCCTTTGTCATCAGCCAGAGGTTATTCTGGATTCTAGCCTGTATATCCCTTCTT
19	38185871	SCAFFOLD290056_19398	0.47	0.45	CCCGTCTTCCAATTGTTTAAAAATGTCCAACCCAGCAGTCAGTAGAACTAGAGAATCAGTTTTAGACTCTAAGGAGCTCTAGCCACACAACCTGCTGGAA[A/G]GGAAACTGACTAGGACAGCACAAGTCTAATTAATTTCACTCAGAGTAGGCACCATGTGCAAACACATGCTCATCAGCAAAGTAGTCTGGTGGTACATGAA
19	39870292	G73147-486-S	0.49	0.00	AACCTCCTTGAGGAAGCCTGTGAGGGGTGGGATGGTGGGTGGTGGATAGAGCCCYGGGCTTGGCAGAAGCAAA[C/G]TCTCCCCTACCATCCCTAGAAGGGAGGAGACAAGGCAGGTTGGGAAGGACTATTCTGGCCCTGGGACCACCTCCCCAGACACCCCGTGTCTTAGCTTCTC
19	40020618	BES1_Contig469_977	0.45	0.08	TTCCCAAATACCCTACCCCCATCATGGAGGCTTTGGTCCAGAGAACTAGTCGTACACAGTTACACCAAACTATTTTTCCTCATTCTTCAAGTCAACAGAC[A/G]AGGTAGATACTCCTTAACAAACACAAGTATGTATACATATATGTAGAAGGACAAGTGGAATCTTTCCTTTAAGCCTATGATATCAGAAGTGTGAAGTACA
19	40100049	SCAFFOLD49643_12002	0.14	0.22	AATCTTGTTAACATAGCTTACAAGAAATTTCTTCCAGGGTGCGGGAAAACTCCGCAAATGAGGCAATAAAAAAAAAGAGGGCTTATAAGAAACTAAGAAC[A/G]CTAACATTTGTGCTCCCATTATGTGTCAAGTACTGTGCTGGGTGACTAGGTAGTTCCATGCTATCCCATCTAATCAGCAAACAAAGTATGCTAAATGCTT
19	43319410	SCAFFOLD220003_30109	0.42	0.33	TGTGCCACTGCTCCTCCACCAACAAGGCCACGGCCTCCCTCCACTGCCCGAGCTCCAGGCAGGGGTAGACATGAAATAGATGTGGCGACAGCAGGCCCCC[A/C]GGGGCCACATGTGTGTCATACTCAGCCCACTCCAGGAAGGAGAATTTGGGGACATGAGTGCAGGACCATCACATAAGCTGTCAGTTACACTGTGATGGGT
19	43492519	SCAFFOLD281250_1419	0.42	0.33	CCTCAAATTTTTTCATCTCTTACCCAGACTTGAAAACTAAGGCACTTGTAACCCAGGGGAAGGCAGCATAAGCCAAGGGAATCTGAGCTTCAACAGAGCT[A/G]CATATCTGAAAAAACAGGGAACGAGGTCATGAAAAAGGAGCTAGAAAGAAAAAAGAGGAGGGGAACTGTATGGTATAATGGAAGGAGCCAGAAAGAGCAT
19	44753088	BES4_Contig492_558	0.48	0.39	CGACGGTGACCTGAGGCTGCTCCTCCTCACCAGTAGGGGTCTGCACACTTGGACAGCCAGGCGGAGCCTGTGGCCCCTCATTCAGCCCCAGGGTGCAGGA[A/C]CTGTCCACCTCAATGGAGGACCAAGAAGCACAACAGGGGTCCTGTTTGGGGGCAGCATGGGGGCGGGTGACGGGAGATGGAATGTGTTCTCACATCTCTC
19	47869681	SCAFFOLD146_27046	0.27	0.30	GTATTTCTTTAGTTCTCCCTAAAGCATTCTATGCTTGCATCCCAAGATATATGTATTGGGAAGCTTGAGAAGATTTCAGAAGATTTGCTTTCCCCTCATG[A/G]TCCAGTTGGACACTGCCGTGGCTGTCACCCCCTACCCCCATATTTAGGCTGTGTGTTAATATTAGTTCATCCAGGCTCTGAATTCCCTGTGTAGTGGCTA
19	49864900	SCAFFOLD290149_8228	0.06	0.10	TTAAGGTTCAGGGTGGGGAAAAGAAACACCACAGAAATGGACTCATATTATGTGGGAGGAGTTGGTGACTCTCAAGTACATGTCCTTGCCATAGACTCTC[A/G]AACCAACCATCTCCTAATCTGAAAGGTAGATCTCTATTCTTCTCCAAACCACAAATCCGATAAGTGATTGAACTTCACCCCAGTACCAAAAGCCCCAGAG

19	49864903	SCAFFOLD290149_8231	0.06	0.11	AGTCTCTGGGGCTTTTGGTACTGGGGTGAAGTTCAATCACTTATCGGATTTGTGGTTTGGAGAAGAATAGAGATCTACCTTTCAGATTAGGAGATGGTTG[A/G]TTCGAGAGTCTATGGCAAGGACATGTACTTGAGAGTCACCAACTCCTCCCACATAATATGAGTCCATTTCTGTGGTGTTTCTTTTCCCCACCCTGAACCT
19	50972227	SCAFFOLD195062_2149	0.06	0.01	AGGCTTAAGGGGGATTATTCCATTTGGACAACTAATAGCCTAATTACTATTTTTTAAGAAAACAACAACCAAACTCCTTTTCAGCCAATCTTGGCAAGAA[A/G]TCTCTAAGAAACACCCATCTCTGCAGTACCATAAGTCATACTGCTAAAAAACATAATTACACTCAGCTGCATCCAAGTTTCCATCAGACGCAGTCCAAAA
19	51542877	SCAFFOLD256301_5813	0.36	0.28	GGCTAGGACACTCCCACATGCTGTTCGTCAGAGGTTGTCGGCAGATGGCCTGATGGTACTTGGTGTCGCTTTAGATGCCCTGTGTGTCCTTGGAGCCTGA[C/G]GTTTACAGATGATGCCCATGCTGAGGAGATCATTGTTGTCTTTAATTGGGGACTTTGGGAATAGGATACAAAGGAAGGAAGTCCTCCGTGTGATGGTGCT
19	54405535	SCAFFOLD316730_30253	0.21	0.25	AAGGGTACAACGAAGGTTCTTGAAATGGGGCATCCTAATCCTGAGGGGGCCTCCCCCCACAAGATGATCAAGACTCACAGGGCACTGACGGGCAGTGGGC[A/G]GCATCTTGTTATCAATTGTGGAGACACACAGACCAAGCGCCAGGCACAATCGTGCCTGCATTTAAAATTTGCAACAATACTGAGCTGGAGGCTCTGACCC
19	54919331	SCAFFOLD250290_12600	0.12	0.09	TGCCACGTGTCCCCCAGAAGGCAGAAATCCTCCCAGTTGAGAATCAGTCCTCCACCCCCAATGGAAGCACCCTTCTGCACGTTGGTATGTCTCTTTCGAA[A/G]TCTAGAAAGGGAATTTTTCTGTACCTGGGTCAGAAGAGACGGAGACCAGGATGCAGCCCGTGAAGGGGTGGTGATATCGGCGGGGGTGGAGCCGTGATGG
19	55171760	SCAFFOLD105423_15698	0.35	0.42	GGTGAACATTCCAACAGCTACTTCAGGAGAAAGTTAACTAAAAACAGCTTAGATCTAGTCTGAGCAAAAACCACTCTGAGAAACAACACAAATTCTACTG[A/G]TTCACACCCCATTCTAGAGAAAGCAAGCCTAAAGGGAGTTTATTCTGCTTTTTCTGGACTTAACAAACCAGAAAAATTTCTAGTCCATGAAGATGAGAAG
19	55229010	SCAFFOLD188448_3370	0.43	0.41	GGAAGACAGACACTGACATCCGCACACTGAGCGCAGAACTTGAACAAAAGGACAGCAACCGTCACGGATAAAGAAAGGAGTTAATAAAGAAGCAAACACA[A/G]AGGAGCAGATCTCCCCGAGCCCAGGAAAACTCATCCTAGATCTGAAAATGAGACTGACAACTAGCAGTTTGAAAACATCAGCGAGACACACACGCGTGGG
19	55411554	SCAFFOLD154062_3866	0.23	0.24	AAAGGTCCAAGGGTTGTGTAGCAGGTTGACCCCGACCTCTCTCTCTTTCCGGCTTCAGACAATGCCAAGGGACAGGCTGGGGAGGCCTGGACAATGGTCT[A/G]CAGCCGGAATCGCCATTCCAGTTTGCAGGCCTTTGTTTCTGCTGGGAGGGGCCCGAGAGCTAATGAAAAGGTTTGTGGCTCAGATGTCTATGAACAGTGG
19	56193816	SCAFFOLD161280_6022	0.38	0.36	GGAGAGCTTCAGCAAAGGGTAGATCCCGGCCAGGGGTGGTGGGCAGGGAGGGGGCCCTTGGACGCTTTCTCATGTTATTCCGTGGGGTTATCTCTGAGAC[A/G]GTGTGAATGAGAGCACGGTCACAGTGCCCTGACCCCGACCCTGGGCCACGGCCCTTCCTGCGGCAGCATGACCTTGGCTGAGGTGCTTGGTTCCCCTCTG
19	56232065	SCAFFOLD81049_10623	0.25	0.42	AGACACAGCCTTGGGTCTTTCTCCTGCAGCATCAGGTAGCCTGCATCGCCATATATTCTTGCATTCGTTCTCCAAATTCCTTTCTGGTTCTAAAGTTCTG[A/G]TTCCAGATCACTCTCAGCTCTAGCTGACAATCAGAATCATTTCCAGAGCTTCTTAGTAGACAGATTACTGGTCCTCGCTTCCTAGAGCTCCTGTCAGGAG
19	59441396	SCAFFOLD166325_461	0.45	0.37	GCTCATTGTCCTGGCCAAGGAGAACGGGCAACCTACACCACGGGTGGGGATCAGCCCCGTCTCTGCAGAGAATCTCAGCTTCTGTGACTCCAACCATTCC[A/G]GCCACATCCTTTAGAAAGAGTGGAATTTCCAAACCGCAAGAGCCATGATGGGCATGGCAAGGTGGTCATCTCCCCAAAACATATTTCATTGAGTGGTGCT

19	62524811	SCAFFOLD245220_10416	0.37	0.41	TATGACTGTTATGCTTTAATCTGGAAAGAGCAGAAATCAGTCAGATGAAATTTGAGACTGTCTACTCTTGGTCCGTAATGGTAACTCAGATGGAACGATT[A/G]TTTTGAATCATGAAAAGGGAATTTCTTGGTGTCAGCTTATAAGATCCTGAAGTCAGTCAGAACTCACAGATACAAGCCTATTTAAACGAGGCCTCCCTGC
19	63029103	SCAFFOLD212069_21366	0.46	0.43	ACCTCCTCACCGAGTCCAAGTGGGACCTGAGTTCCCGATTCCATTTTCCTTTGAAAATTCTCATTTCCCTTGAGGTGGTGGGAGGCGGGCCAGAGCGGTC[A/G]CCATAGGAACCACATAACCATCACGAGGTTCGACATCTTACCATTTCCTTGGTCAAGACCACAGATTCAAAGAACTATTTCGTGGCACATAAGGAACTCA
19	63357121	SCAFFOLD125568_5025	0.26	0.41	AGAGCCGCGCGCGCGCGCGCGCGTGTGTGTGTGTGTGTGTGTGTGTGTGCACGCGACAAGCAGCGGTGAAACAAAGGAAGACTCTGCCCCTCCCACAC[A/G]AGTGCATTCATTACTCCAAAGATCTGTCTCTTCTGCTGTCTTCCATTAGTCACTCCTACTCACACATAACCTGCTGCTGCTGCTGCTAAGTCGCTTCAGT
19	63614676	SCAFFOLD245209_18582	0.24	0.45	AATCACTAGGGCCTGACGAGCATTCCAGGGAACCTCTTTCAGTGACGGACACTCCCACTCGTATATAAATAAGAAGCCCCAATGCAATTTAAAGCAGCTT[A/C]CACTCATCTGGTGCTGATGCGGGAGATGGGACGACTGGCTCCTCCCAGGCTCCCTTGGCATTGCCTAAGCAGACAGGCATCCCATTCTGTCGCTAATTCT
19	64695847	SCAFFOLD138153_833	0.44	0.47	CAGTGAGCAGCGGGGGAGGAGGCTTGGGGGGGGACACGTGGTTCAAGAGCTACGTTTTGGAAGGCGGGCCACTGCGGTCTTCACACCCCCCGTTGTTTGT[A/T]GTTTTAATTTCTCGCTGTCGAAGTAGCTCCTGTTTCCGGAGGGACATCTGGGAAGTGAGAGCCAGGGTTCGTTATCCAGAAGCAGCCTCTGAGCTGTGGG
20	762879	SCAFFOLD90561_9460	0.36	0.29	GCTACGATAGCAGACTGTCTTTGCATGAAGAGCATTGATAGTCCTGCTCCCCAAGTCCCGGATCTAAGCCCCCTACTGTGAAACGTGGAAGGAGTCAGCG[A/G]TACAGGAAACCTTAGTTCAGCTAGTTACCAGTAAACATATACAAGGTGCTGCTTCTTAGAGAGCTGAAAATTATAAACTCAGTTAAGAAGAGTCCTGGTC
20	903521	SCAFFOLD65247_3863	0.48	0.50	AAAGATGGACTCGAGAAGACAGAACACAGAAGCAAAGGCTTTAATGGGTATGCAGGGCAGAGACTACCAGGAAAGGAAAGAAGACTCAGAGGCCTAGGTT[A/C]CAGGGCCAGCTTTGCTTCCCACTGCGTTGTCCCCTCCTGAGCATCTCTTCTGCCTGGTGGCCTGCTCAGTTTCTCTGGCCCAGGTGCTGGGAAAGCAGCT
20	1000502	SCAFFOLD100226_13722	0.31	0.24	TGTGTATAAATTGTCACCTGATCCTTAGAGCAAATCTACGCAGTTTAACACATAATGCCCATTTTACAGGTGAAGAAATTGAGACTCAAAAAATTTAAGT[A/G]ACTTGCTCAAAATCATACAAGATTTGCATGTAAACCTGGCTGAATCCAGTTAACTAATGCTTTTATTACATTGAAGACATAGGGTATTACTCTGGGAACT
20	2353627	SCAFFOLD275311_14169	0.37	0.35	AGCTCCGCAGCAAGAAAAGCCACCACGGTGAGAAGCCCGCACCGCAGCAACGAGCAGCCCCTGCCCCCTAGAGAAGTGCTGTGCACAGAGACAGACGCAA[C/G]CAGCCAGAAGTAACTACGTAAAGAGACATGTTTAAAAGAAGGCTGAGTTCTCCCGGGTTTTTCCCCTTGATTCAAGTATTTCCCCATTCAGAACTAGTGA
20	2388152	SCAFFOLD145774_16111	0.13	0.16	AAAAAACTAAGGGGGCAGAGAAGGAGAGAGTGGGTACATATAATTACATTTCTTTTACAGTGCATTCATTCTCCACTTTATTTTTTCATTAGCAAAAATG[A/G]GAAAATTCTGACTTGCTTAAAGAGTCCCACCCAGATCACACGACTAATAGATTTTCATGAAAGGCAACTGTGAACATGATCAGCAGAATATTAAAATGAA
20	2388212	SCAFFOLD145774_16171	0.33	0.49	TGTGATCTGGGTGGGACTCTTTAAGCAAGTCAGAATTTTCTCATTTTTGCTAATGAAAAAATAAAGTGGAGAATGAATGCACTGTAAAAGAAATGTAATT[A/G]TATGTACCCACTCTCTCCTTCTCTGCCCCCTTAGTTTTTTCTGCTCCCCCATCCCCTGCTGCCTCTCCTGAATCCTGTTCTCCAAAGGAAAGTCCCATTT

20	3527172	SCAFFOLD240282_2931	0.13	0.23	CAGTGTCTGCAGCATCCCCTTGTCCCAGGGGCACGGGGCTCCTGCTGCAGACTCTCATCTGGATAAGCATCATCCTCAAGGTCTGACCCCGCATTCATTG[A/G]CAAGTTTCCCGCAGCACCCAGCTCCATCCTGCCCCCCACAGAATTTCTGGAAGATTCTCTGAATGTCCAGGCCTTCTCAATACATCAACGCCAGGGCGAA
20	3527615	SCAFFOLD240282_2488	0.06	0.13	CTCTGAGCACCGCAGCATCTCCCACGAGAGCCTTCCCGATCGCAGAGCACGATGGAGCTAGGAGAAGCCACTTCTCAGCTTCCTAGCTGATGAACGGTGC[A/G]GCTAGAGGTTTTCTTCCTGGGCTGTAACTGACAATACCTGCCCAGCTTCTAATTCTCGAGCTCTGTGTGACCTCAAGCGAGAGTGCAATTCTGAGCTTCT
20	3544207	SCAFFOLD105178_9371	0.18	0.16	GGGGCTCTCCCACCCCTGGGTTACATTAGGAAGAAATTTTGGTTCCTCGGTCACCCAGCTCACGCTGATTGCCAAGGGGACACTCATTGCCTTGGTCAGT[A/G]TGTTCTTCTCCAGTCTGTTGCAGGAAGGGGGACCCCTTCCAGGGCCCGAGAGTGGGCTCTTGTCTAACACTCGGTAATGAACTGAGAAGACACATGTGCT
20	3888519	SCAFFOLD205088_415	0.21	0.17	AGGAGATGAAAAGGGTAGAAGCAGCTGCAGAATAGAAGCCCAGGAGTGACGGGGAGCGTGCATCAGAAAGCAACGCCAAGCCTCCTTCCTACCCACCAGC[A/G]GGGGTCAACGTGAGGCTTCCAGTCCACTCCTCTCTCCAAGACTGCTGCATCTTATATTTTATATTCCCCTAAATGTGTAAGATTCAGGCCCCACCACAAC
20	5017140	SCAFFOLD65168_22156	0.07	0.08	TCCATCTCCTGGGAGAGATGCTTGGGGCCTTAAGCCATCAGCTGGTGGTTCAGCCTTCCAGATTTCTGCTTCACCCAGTGAAAAGTGTACACCGAGAAGT[A/G]AAGAGGCAAGCTCAGCTGGACTGCAGTTCTCTGCACCTCGGGTGGATTTGGCAGTCTAGAAAGTTCTCTATAAAAAAGCGTTGGCTTGGTTTTGGGCGAC
20	8676291	SCAFFOLD275478_11846	0.19	0.21	CATTTTTATGCCTTCTGTCACCACCAGGTCTGGCCATCACGGACACCCAGCTCTCCTTTTCTCTGAAACTCAGTGACAAAACACAGGAAACAATATAAAA[A/T]TTTTTCTTATCCCCTTAACCCAGCTGAGATGGACCAACTTTTTTTTTTTTTTAAGAAATTTAAAAAACCCCAGGGGATGGGAGGCAGGAACCCTGAATCT
20	10512024	SCAFFOLD266330_825	0.31	0.32	TGGCAGGAATGCCCTCCCTCACTTTACAGGGGGCACCTGGATACCACCCCAGGCCCAAGCTGATGGGAGCAACCCACCACCAGTGGCACAGGTGCCTCTA[A/C]ATCATCCCACAACACATGCCATCATCCATGCCTGACTGTATCTCCACACAGGTTAATTGGCAACTTTCAGTGTGGTATTTCTAAGTGGAATTGCTTCTCT
20	11905970	SCAFFOLD100002_29562	0.33	0.24	GTAGTAGTTTGAGTCACGTATATGGGGCATTTTTTTCTGGGTCTCCTTTGCCAATGATCTTGCTTTGCCTGGTTCTGAGTCTGTATTTGGTTATCTCAGC[A/G]CACTCCCATGTCTGCATGCGCATCTCTTAGCCAAGATGATGTTGTTCAGTTGCTCAGTCATGTCCGACTCTTTGCAACCCCATGGATGGAGCAGCATGCC
20	13276680	SCAFFOLD120004_10180	0.27	0.26	AGTGGCTCAGAACCACAGCAGTTACAGTGGAGCTGCCTTGGGGTTAGGGAGTGGGAAGGGATATTTGGAAATGAGGTTCACACTCCGCTATTCCACCTTT[A/G]CTTCAGCCAGAGAAGATCAAACTCTGTCACATTTTGGGCTAGAAAGCAGTCTTTGGCTTGAGTGAAAGATTTAGCATGTACATATGTTTGCACATGCATT
20	19170732	BES1_Contig298_937	0.36	0.37	ATGTAAATGGTGGGTGAGCTCCAGAAGCAGATTAAAAAGTTGGGTTCCATTTTTTTTCTCCCTGAAATACTCACCAGAAATACATCAGTAGGAACCTGAG[A/G]GAACTATTTGCTATCTCTTATTGGTTCTGGTTCACATGCTTTTAGTCACTGAGGGTGTGTCTCAGTGTTACCTTTTTTTGAAGCTGGGATGCACAGATAT
20	19170831	BES1_Contig298_838	0.37	0.36	CCCTCAGGTTCCTACTGATGTATTTCTGGTGAGTATTTCAGGGAGAAAAAAAATGGAACCCAACTTTTTAATCTGCTTCTGGAGCTCACCCACCATTTAC[A/G]TGGCATGGTGACTAAACCCGAACTCTGCCAGGAGCCAGTTTAACGATGCAGTTAAATGAGGAAAATGTTGCCTCAAGTGAGTAGCACCTGTTTTGCTTCC

20	19510164	BES11_Contig509_1565	0.44	0.44	GACCATTTTCTTATACTTTGTATATCAGTCTCACAACATTTTGGGAAATCCATGAAGTACTACATGTTCTGGAATACACTCACACCTGTAAATCCATAGC[A/G]GTAAACCAAGGTTGAAAAGCAAAAGTACTGTATAATATAACTGAATTATGTATCATTGTCAAAAGGAGAATAAAAGTAGGAAGAAAAGAAGCATGAATAA
20	22068656	BES11_Contig492_674	0.15	0.12	CTCAAAACATCCTATTAACCATTTTTCTAATAGGGATGTCATTTTTTTTTTAATGATCCCTTTTCACTCTTTAAGGTTCAAGTTAAACCTGACCCTGCCT[A/G]GGATCCTAAAATAATGGCTCTCTCATCTCAACTGATATGAGGCATTTAACCTCATTCCTTACATGGAGTTAAGTCATCAACAACTATTTTCAGATTGGGA
20	22322841	BES5_Contig242_347	0.28	0.22	AATTATCTCAGAGCAGGGACAATGGGAAGAGTGTATGAGGACATATTAGGCACTTTCCACTGGTAAAAGAGATGAAGAGATACAGGAACTCTACACTACC[A/C]ACCACAGGGTGCCTTTGTAACTTACAGCTCAACACAGCTTCAAGCTCCCGTCCTGCAGGCCCAGACTCATCTTCCCAGGACCCTGGGACCACTCTCCTCT
20	22715561	SCAFFOLD141483_12923	0.19	0.44	CTTCACAGTCCTCCTGGGGATATTGTGTTACCTCTGCACCATCTCTCAATCTTTTTTCTTTCTGAATCATTTTAATTCAATTTATGAAAGTCACATACAC[A/G]TGTGGTCTGAAGCAATGGGTAGCTTTTCACTGTTGAAGCCTTTCCTTGGCAGGGGAGAGACAGCCTCTGATAATGGCTTCCCAGATAGCTATTCAGGAGC
20	22715783	SCAFFOLD141483_12701	0.04	0.17	AAAGTTGAAGAATTAAGTGAGACCGATGACTTCAAGTGGAGAAACTGAAAGAAACGGAACAGAGTGATAAACAGCTCACTCTGGGTAGCAATGAATCCAT[A/G]GGCTGCACTTGATTCTAAGCAGGAGTGACTGGTGATGCTAAAGAACACTGAAGGGAGAGCTATTGCAACCAACCCCAGTAAATCCACTCTAGTTTATTTA
20	22885733	SCAFFOLD67085_7878	0.08	0.14	TAACTGAAATGAAACATTTTTTTAAAAAGTCCTATCTGATTCTCACTGATATCTCAGAATTCTGGAACAAATACTGGGCTTCATTACTTTTCACGTCAAT[A/G]TATTTACTTTCAGGTTCAGTAAGAATCTGTTCTCCTATTTACCAGGATTTAAGCAGACAAAATGATTGTGCAAGTGCTGTAGTTGGTGTGTCATATTAGA
20	23450783	BES10_Contig679_1007	0.03	0.08	TCTTCCAATCAGGGAATCAGTCCAGAGAAACCACAACACAGGAAGAGAAAACTAACAGATGATTCTGTTTTCCTCACCACCTGCATCCAACACAAATAGG[A/G]GAAGGCATCAGGAAGCAAAAACCCCGTTAAAAGTTATGAGAGTCTATGGCTTCCTTGAGAACAAACTGGATTCTGCTTGAGTTATAACTCTTTCATCTTT
20	23807388	SCAFFOLD127505_336	0.43	0.37	CTGGGAAGTTGGGTAGATGACAGTGCCACTCACCAAGATGGAAAACACAGCAGGAGAGAGTCTGTCAGGCAGGGCGGGCTTCAGGGGCACATGAACTGCA[C/G]GGTCAAACAGGGCTCTGCTCAGAGGGGACGCGCGACTGGCTCAATGATCTGCTGCTGCTGCTCTAAGTAATATTGAATAAGGAATTTGGCACTTTGGATT
20	24608540	SCAFFOLD108264_1584	0.49	0.38	CCATTCCACAGTTAGCATCTCTATTGGACATGGGGTGGGCAGTAGTGCTGGGAAATGGAATTTGCTGTTCTAAGAAGCATGTCTGTGTTGAACTTCAGTA[A/G]TGATGAATATCTAGAAAGCATTTTCATAAAAGGAACTTCGTTTTAATAATGTGCTCAATTAGTCCAATCGTGTCCGACTCTTTGCAACCCCATGGACTGT
20	25849454	SCAFFOLD155700_12715	0.48	0.04	GAGCACTGAGGGTTTTCAAGTCAGGAATAAAGAGCATCACCAGCTTTTCTGAGCCCCTTCTCTGTCCACCTGCCCTTGACCCCGTGTGTCTGGTGCCATC[A/G]CAGGAAGTAGAAACAGCCAGATGTTCTGTATCTTCATATGGTGTTAGTCCAGGTAATGGGCTAGTGACAGCTGACTGTGGAGGAGACTGCAAGTGAGCAG
20	25849654	SCAFFOLD155700_12515	0.47	0.43	CTGGATCTGGAGGAGTCAAGTGGCTGAGGGATCTTTTTAGCACTTAGTTTTTTTCAAGTCTCACCCAACTGTGGGGTGAAGGTGGGAAGGAGTCGGCCAC[A/G]TGTGTAACTTGTGCAAGAGGGAGGCCAAACGCAGCACTCAATTGTTCTTGGGGAAGCAGAGTTCAACAATTTAAAATCAACTTTTGTTCTCAGATAGCCA

20	28092109	SCAFFOLD130042_46665	0.33	0.29	GCAGTTGCCGGGCTGCCCTGGTGGCTCAATGGTAAAGAATCCACCTGCCAATGAAAGAGACATGGGTTCAGTCTTTGATCAGGGAAGATCCCGCATGTTG[A/T]GGAGCAACTAACTAAGCCCGTGAGCCACAGTTACTGAGCCTGCTCTTTAGAACCTGGGAGCTGCAACTACTGAGCCCACGTGCTGCATCTGATGAAGCCC
20	33897071	AF140284-257	0.28	0.18	AGCTAACTTCATCGTGGACAACGCTTACTTCTGCGAGGTAGACRCCAAAAAGTACATTGCCCTGGCCCCTCAYGTCGAGGCTGAATCACACGTAGAGCCA[A/G]GCTTTAACCAGGAAGACATTTACATCACCACAGAAAGCCTTACCACTACAGCTGGGAGGTCGGGGACAGCAGAACATGTTCCAAGTTCTGAGATACCTGT
20	33897128	AF140284-200	0.06	0.07	GAATAAGACTGGGAACCCCCAGTGTGACACGCACCCAGAAGTGGTCACACCCTGCCAAGCTAACTTCATCGTGGACAACGCTTACTTCTGCGAGGTAGAC[A/G]CCAAAAAGTACATTGCCCTGGCCCCTCAYGTCGAGGCTGAATCACACGTAGAGCCARGCTTTAACCAGGAAGACATTTACATCACCACAGAAAGCCTTAC
20	33897128	GHR-AF140284-200	0.12	0.08	GAATAAGACTGGGAACCCCCAGTGTGACACGCACCCAGAAGTGGTCACACCCTGCCAAGCTAACTTCATCGTGGACAACGCTTACTTCTGCGAGGTAGAC[A/G]CCAAAAAGTACATTGCCCTGGCCCCTCAYGTCGAGGCTGAATCACACGTAGAGCCARGCTTTAACCAGGAAGACATTTACATCACCACAGAAAGCCTTAC
20	33938999	GHR-AY643807-300	0.10	0.06	GGATCTGTACAGATGTTCTATATCAGAAGGTATGGGCTTCATGCTTTTCTGATTTCTSTCCATGAATTTTCTGATGAAAATCCATTGAGTGTCATGCAGT[A/G]GTGGGAATGGAAATAATCTTCTTTGGTGATCTAAATGCATTCACCCATTCATTCATTTAAATATATTAGTTAAGCCCTTACTATATGTTGGG
20	34086084	GHR-AF126288-149	0.28	0.41	GATGAGCCTTGTGAGCCTCCAGCCCAGGCCTGGCCTTCACTTCAGTTGGCAGAACCCAGCCCTGGCAAAGGTCGGGGGGGTTCGTTATGTGAGGCAATGC[A/G]TTGTGTGCTCTAATCTTTTCTGGTACCAGGTTGTGTGTGTGTGTGTGTGTGTGTGTGTGTGTGTGACTGGGAGGGAGGAAGAGAGAGAAAATGTAATTGA
20	36202145	SCAFFOLD165003_41555	0.28	0.32	ATCAATGGAGTTATCATTATCATCCTCATTAAATATTTGTGAAACATCTCCATATGCATAGTGGCAGGTATTTAAGCATGATGAGAAAATGAAAAGAAAT[A/G]TATAATTATGATGTGTGTCCTCAGGGGCCTTGAACCTGAGTTGTGCAGACAGATACATTATTTATGAAACTGTGAATAGCTGTCCACATAGGATACTGTG
20	36394404	SCAFFOLD108770_1519	0.21	0.35	TCCTTTGTTCTAAACCCACTCCTTGTTCTTCCAGCTCCAGAATTCCAAACCTGCTTAACCCACATCCTTCACTTGGAAACAAAAGAATTTTATCCTAGCC[A/G]TAAGTATGAAGAACTGTGAAGAGCTGGGGTCAGTTATGGGGAATGCCATGAATATTTAGGACACTAAATAATATCTTTATCACAGAATCATTGACCTGAA
20	36955575	SCAFFOLD11088_1144	0.23	0.23	AGTCCCTCTAATCTTTGACTCCTTGCTTTACTATTGTACTTGTGAACTATATGATCCTCTGAATCTCAATTCCTTTACTAGAAAAATAGGGCTGTCCGTT[A/G]TGCTTCTTCCACAGGGTTAGGTATCAGTGCTTGAAAGTACTTAGCACAGCACCTAGCATCTACTACAAGCTCAGTGAAAGGTTATGCCTTCTTATACCAA
20	37333792	SCAFFOLD140021_29827	0.30	0.26	GACAGGAACAAATAATCCAGTTGAATGCAGCAGGCCTAACTAATACTAGAAAAGCTAGATTTGTAGATTACAAAGATCCGTAAGTACATTAGTAGGTAAA[C/G]CCTAGATTGATTCCTAAAACCAGTGATAAACCAATGCAGTGACTCAGGTCAGTGGTGATCTCAAGCTTGGGTTATGCAATGTAATGGACACCCCCTTACA
20	37928023	AJ505155-BULGE113-137	0.19	0.31	GTTTGAACTAGAAATCCCAAGATTCTAGACTCTGAGCCTAACACTATAGTTAAATGAGACTTTGGGGGGCCTTCAGAGGGAGAAAGTATATTTTGCACAT[A/G]TTTTTGTTTTGTTTGTTCAGTTGCTAAGTTGTGTCCGACTCTTTGTGACCTCATGGACTCTAGCATGCCAGGCTTCCCTGACCTTCACCACCTCCYGGAG

20	38797757	SCAFFOLD135010_59089	0.43	0.48	ACCACACAGTAAAAAGAGGTTTCTGTAAAAGCCTTTTCCCAAGCACACCAATGACTCCTTGCCCCATCACATAAAGTCATTTCTTCCCAGCTGCCAGGCT[C/G]CTTCTGAAAGAGCTAGAGCCCTGCCAGTTACAGTAGCTGGTATAAAAGCTAATCTTATAAAACACACGTCACTTCTCCAACCCCATAATTAAGACCAGAA
20	38814738	SCAFFOLD6393_4411	0.50	0.44	AGTCTTTGAAGCCACCCTTGGGTCCTTGCCAAGCAGGGCCACATTCTTAAAACATAACTACTTACTTCTTCAAAGCCAGCAGGAGAGTCTCATCCTTCAC[A/G]AATGGCCCAGTCCTGCTTCAAGGGCCTTTGCCTGATTAGGACAGGCCCACCCTATATCTATCCCCTCCTCTTGATTAACTCAGAATCAACTGATTTGGAA
20	39871699	SCAFFOLD125283_9837	0.05	0.12	GGTGCTTTTAATTTTTTTAACAGTTCTGCTGAAATGTAACTCATACACATACGATTTACCCCTATTGTGTATAATTCAATGGTTTTTAGTGTATTCGTGA[A/C]GTTGTGCATCCATCATCACATTAAAATTTTAGAACATTATTATTACCCCCTGAAAAAAAACCCACACTCCATAGCCATCACCCCCGCCAATTACCCCATT
20	41612944	BES2_Contig210_1321	0.18	0.16	ATGTGTAAATACTTTTGTTATTTGCGATGACCTTACATCTAATAGTGTGACATTTAAAAGAAAAAACTTAAGATTTTGTGACTTTGGTCTGGGTGGGTCT[A/G]TGTTTTTCTAAATAAAATATCACCAGAACTCATCACCTAATTTCTGTTTTTCTTTTTAAAGCTTTACATATTACTAAAAATTTAGCACACTTAAGGTTTA
20	42753545	SCAFFOLD115711_487	0.27	0.32	TTTCCAAGCTGTGATAGCTTATTATTCTGATTCCCTATGTACCAACTATTCTGCTTGATGGTCTCTTTGTAATTCTTCCCTTTTGCAGTATTTTGGTTTT[A/G]AATTTCTCTTCTTAGAAGTTTGGGTGAACTCTCTGCTGGATGGCAAACTCCTTGAATGTAGGGGTTTGCTTTAATGCTTCCTTAATTAGACCAGAGCTTG
20	44651005	BES8_Contig309_741	0.50	0.45	TGACCTTTACTATAGTACCGCCTCTCCCTCAAGACCGCTCACTGCTACTAAACAACTAAATGAACTATTTAAAGGTCAGTTAGGTAAAATTCAGGAGCAG[A/G]GGAAGTTATTAAGAAGCTGGAAGCGCGTCGAGTTTGTCACTGCCGCCTGAGGGTGGTGGTTGAAAACATAAGCCATTGCTTGCCTAGATGTCCCTGTCAG
20	45114461	SCAFFOLD230335_23211	0.15	0.21	ACCAATTGGCATTAACAAGTGTGATAGGTATTTGAAAAATTGTTGGCAAAACAACTTTCTTGGAAGACAGAACAAAAGAATTAGGAGGTTTAGAAGTCAG[A/G]CAACTTCTAATATCTTATACCATTGACAGTGATTTGTTCCTGTCATAGCTCTTCTCTGACCTGTTAGTGAAGGACAAAATTCTAAAAAAATGTTTAGTCA
20	45377221	SCAFFOLD70871_6268	0.14	0.22	TACTATGAATTATAAAATATTCACTCCTAGAAAAAAATAGAGGAGAAAACTACTCTACATCCAAGCCTCCATCTTCCTCGAAAGAAAAAGGCTTGTAAAA[A/T]TTTTTCTGGACCTAAAAATTCCTATAAATGAATAGTAGGCCCAAGTTATATGAGGAGGCACAGGGAATTGATTCATGCTTAATAAACCCTGCAATTAATA
20	47684622	BES7_Contig376_1523	0.32	0.15	ACGTTTCCTGCTGATCTCCTATTATGTCACTTAGTCTTCTCAGGAGTTACCTGTGTTGAAAATATGAGAAAGAGAGACAAATGGAAACTTCAAAGAGAGA[A/G]GCATGTTTGTTGTGTTTTCAACTCAAGACATATACTGGATATATGGACACTAAGTGAGGAACATAAAGCTTATCTCCTTTCAACTCAGAGCACAAAGAAA
20	47929842	SCAFFOLD130061_3178	0.15	0.06	CAATTTGCTTTCATGGGGTGAAATTTAAGCTGGGCCCAGATTTCTTGTGAGTGCCGTTTAAAGCCAGAAGATAAAGGAGCCATCAACATTTTCTTCAGGT[A/G]AATAAAATATGACCATTACAGTCCATGCAGTCACAAAAGAGTAGAAAACAACTGAGCAACTGAACGACAACAGCAGCATATTACGCCTAATTAAGGTCTT
20	48663198	SCAFFOLD260326_4201	0.43	0.32	TTTTGTTATTTAAGTATCCATCAGCATTATCTCAATTATGTTATTTCTACATAATCATATCTCCAAATTTATGGTGAATCATGAAATTAAGTTCAATGAA[A/G]TTTCTTCTGCAATTAAAAATGTACTTTTTTATTGTTTACCTTTTGTTGTTTTGAGGTTTGTAGATACCTTGAAATAGGCCAAATTGCATAAAAAAAAAAA

20	49534749	BES3_Contig374_1476	0.18	0.19	TCATATAAGCTTTGAATGTACTAGCTACATTTTATTTTTCTATGGCCATCTACTACACCACTGTGGAGGTTACAGGAGAACACAGACAAAATCCTCATTC[A/G]CTTATCTTTAGCACATAGTACATACCAACAGTCTAGAATTAGTTTACATACGTTTATCGGTTACCTGAACGAAAATGTTTGGTATGAATGTTCCTTGTTT
20	50934131	SCAFFOLD20130_2652	0.09	0.15	GAAATAAGCAACATATTTTCAAAGATTTTATTTGTGATATTCATGAGAATCCACATCAGGATCAATGGAGAAGGAAAAGCGTAGTAGTAGTCTAAAGAAT[A/T]TGGCCCACAAGTTCAATGCAGTTATTTAACACAAATTGTAACTTCTTTCTTGGTGAGTGTCTAAGTATCTTTCTGTTAAGAAGAGTATGTGAATATACAT
20	51342406	SCAFFOLD270550_10562	0.35	0.33	GTCGTGGGCAGTGTCAGAGAATCATAGTACCTAATGGATAGCCTCTGAAGAGAAGTAAGCAGTAGTTCCTTCCCCAGAAGAAGTGCTCCAGTCCTGCCTA[A/C]GTCTCCTCCTAGTTTGGAGCTGATCTGGGGTAGACAGGTCCTGAAGAGTTCTATCAATGGAAGCCTTCCAGGTTTTAGGTGGCAGCACATCCACCTTGAT
20	51402608	SCAFFOLD15454_18507	0.46	0.49	GTATTCCTGCCTGGAAAATTCCATGGACAAGAGAAGCATGGAGGGCTACAGTCCATGGGCTCACAAAGAGTTAGACACTGCTAAGCATGCACACATACAC[A/C]CATAATATATGCATAATTTTATATATGTATGCTAAGTCACTTCAGTCGTGTCCGACTCTGTGCGACCCCATATGGCAGCCCATCAGGCTCCTCCGTCCCT
20	53350430	SCAFFOLD180565_9260	0.26	0.32	GAGAGATGGCCTGAAAAAGAATTATTTAGCAAAAAAGTAACCACAACTTAAAGATTTGGAAAATTCTCATCCTATCCGTATTGCAAAAAGAGAGAGAGAG[A/C]GCTTAGTACTGAAGAGAGTAGGTTGAAGGGTATGGTTGAACCACTCTTTGATAAAAAGATTAGTATGAACTCCAGATACAATTTTGAACAGTCACCTCAG
20	54534060	SCAFFOLD48045_564	0.16	0.24	TGATTGCTTCTTCTAGCTAAAATAGCTTTTTTATTTCATTTTTTATTGGTTGAGTTCAATTATATTCAATTTGGGGGCCAAATCAACTCAAGATTATGAA[A/C]TTTCTATTCTCTTTATCAAGGGTTATGGATGGTGACTTTGGATAAATATATATTTATGCTTCTACTAGGGGAAAGGAGAGGGCTGCCAAACTTGAAACCC
20	55818955	BES7_Contig314_1151	0.09	0.27	CTGCCTCCCTCCCACCCCGCACTCTGCGTCCCCCGCTCTCCTTTTCACCCTGAGGCTCACTTAGCTCCGAGTCTCCGCTCTCGCTGCTGCTGTATAATCA[A/T]TCTAGCCTGTCTGAAGCTAATAGTCCTCTATTTAACCAGCCAATCAATAGGAAGGATTCCACTGCTAGTCCCAAGAGTCTGTTTCCAAGTGTGGTACAAA
20	56587233	SCAFFOLD150062_850	0.38	0.25	CCAACCTTTTAAAGTGAAAAGGCAACACATTAACTCAATGAAAAGACTAGATTGTTCAAATCAATAAACTCTACAAGAATAGTTTATTCTTAGCTAATAC[A/G]TGCTAATGTCTCCATTCACAACAAAACATTCTTTGAAGAAACTTTACTTTCCTGGACACATGATATTTACAATAACAATTGCTTCAAATCAGATTGTGTT
20	56612844	SCAFFOLD26178_16622	0.44	0.24	ATTGATTGTTTTCCATTTACCCCCCCTCACACCCTACCCAACTGCACTTAACCATAGAGACAAAAATCAAAGAGTCAAGCATTTCCATATTTTGACTATA[A/C]TCTAATGCTCCAATTCTGCTTTGGTGGCAAGACATAAAGCCATGTCAGTATGACATGAAGCAATATACTGATTTTCCATGCTATTTTCAAGTCTGCAAAA
20	57251001	BES10_Contig414_999	0.47	0.44	TATTTTTTTCCAACTACTGTGCTTTTCTTAGATTAATGATGAAACCAATTGTTCATCTCTTGGACTTTGACAGCATACAGTGAGTTCTGACAACTTTCTC[C/G]CACTCCAGTTCCTGAATAACATGTAATTTCCTTGATATGACAAAGTATCATTTTAGCATTCATGTCATGCCATGGTGTACATTTTTCATTTTTCTGTAAG
20	58927206	SCAFFOLD115952_737	0.42	0.33	GTTTCTATCAGATACCCTTTTAGCCTTAGGTTCAACGAACTGTGTTCTAAATCGATAGTTCAGCAGCCCACTGAACACTAGTCACACCTAACAGCAGGAA[A/C]AATAGGCTCCATCTTTTCTGGGTTTTTTTTTGTCATAGCTAATTACAGCAAAACCAAATTGTTAAGAAGGATTGTGTACTCCTAGCTTGTGCACGTGCAT

20	59419997	SCAFFOLD256529_6850	0.43	0.39	TTTTGAACCGACCTTGCTCCCACAGTGGACACTGTGCCCAGTGCCAGGAGACTCTCCCTGCTCCCTTCCCGTGCCCTCCACCCACATGGAGAATCTGTCT[A/G]TGTGCTCCAGCCACAGTTGCTGGTCTGGACCTCATTATTTTTCCTTTTTTATAAAAATGTTTTTGCAGAATTATACTAATATGGTTTCTGCATTTGCAGA
20	60977129	SCAFFOLD61039_4469	0.21	0.24	TTCCTGCCTCATTTTTTGAGCTGGGATGTAGCCAGCTGCAACTCCTGGTTCTCAGGTCTTTAGACCTGGACTGACCTGCACCCCCAGCTTTCCTGGACTC[A/G]AGGTTATAGTTGGTAGATGATGGGACTTCTCAGCCTCCATAATAGAATGAGTCAATTCCTCATAATACATCTTTTCATATAAAATATCTCCTATCGGTTC
20	62321001	SCAFFOLD80682_16346	0.36	0.45	AGGCAATTCAAATTTATCCTGCATTCCCTTATGAGAATCTCTGCCCAGTTGACCACATCTGTTCTTACCACAAACGTCACGGATTTTCACAAACTCTGTG[A/G]TTTCTGTGAAGACCTCCTTAGTGGAACATCTCACGGTCTTGGTCTCCCACAGGAGCTGCTCACGTGCTGCGAGGAGGGGAAGGGGGAGATCAAGGATGGG
20	62321130	SCAFFOLD80682_16217	0.35	0.44	TCATTTTTGAGGCTCAGTAGTTAACATTACACTAAACTCACCTTCAACTATGACTGCATTTGGTTTCCTCTGAAGCCTAAGAAACATCCTGACTTGAGCA[A/G]GACAAACCCTTCCATCCACTGGCTAAAGAGGCAATTCAAATTTATCCTGCATTCCCTTATGAGAATCTCTGCCCAGTTGACCACATCTGTTCTTACCACA
20	62330948	SCAFFOLD80682_6272	0.19	0.17	GCACACAGGAAAGATCTTTAAGTGAAGAATAACTCTTGCTTTTTAAAGTTAAGGATAACCATAAAAGAAGCGTTCTCCCTCCGTCCCTCCTTCTCTCTCT[A/C]TATATATATATAACATGGACTTCGCTGGTGGGTTAATGGTAAAGAACCTGCCTACTAATGCAGGAGACACAAGAGATGTAGGTTCGATCCCTGGATCAGG
20	62653406	SCAFFOLD132982_3250	0.45	0.33	GGCACCATCTTGCAGCCCTGCTATCAGAGTAGAAGTGTGAAACTTCCAATGTGTTTTTCTGAAATCCATTCCTTTCTTTCTGGTCCCTTTGCAAAAAGAA[A/G]AAAGTCCCCAGAGAGATGTTTCTGATTCGATTTTGCCTCTCCAACTCCTTTCCAGGCTTCATGATAGGAAAAGGGCTCATTAGCTCTGGTGGTATCCACA
20	62653437	SCAFFOLD132982_3281	0.45	0.33	AACCAGGTCATTTTGCTCAAATTGCCAATATTGTGGATACCACCAGAGCTAATGAGCCCTTTTCCTATCATGAAGCCTGGAAAGGAGTTGGAGAGGCAAA[A/C]TCGAATCAGAAACATCTCTCTGGGGACTTTTTCTTTTTGCAAAGGGACCAGAAAGAAAGGAATGGATTTCAGAAAAACACATTGGAAGTTTCACACTTC
20	63516165	SCAFFOLD130370_1515	0.36	0.40	ATGTCAGGAATCAGCATTAGGGACTGAAAAATCAGGATTATTAGTAGAATTTAATAATAAAATATCTGAACTAGAGAACTGAGTGATATTCAAGTAGGGG[A/G]GATGGCCAGAGAGATGAGGTGGGTTCCTCACCATTTTATGAAAAAACTGATAGGATCTGACAAGAGCCAGCGAGCAGTATTTTAGGTCTACAGCCTCTGC
20	63516236	SCAFFOLD130370_1586	0.36	0.41	AGGTGGCTGAAATGACATTTTGAAGGCCGACTCATTCAGTCCTGGGGGTCAGGTGACAGTGTCCCCAGGAAGCAGAGGCTGTAGACCTAAAATACTGCTC[A/G]CTGGCTCTTGTCAGATCCTATCAGTTTTTTCATAAAATGGTGAGGAACCCACCTCATCTCTCTGGCCATCCCCCCTACTTGAATATCACTCAGTTCTCTA
20	65221094	AJ496641-007.SP6-415	0.22	0.30	AGCAGAAGGAGGAGAAGTCTTGGCTGTAAGAGGAAAGAAGGGGAGGCTTCCTGAAGACCTGGAGGGATTTTGAAACACAGGGAAGTAAGAAATTGCTACT[A/G]AGAGTTGAGCATCTGTTGAAAGTAGAAGTGAGAGTAGAGTGGGTGGAGTTGGGGGGARGCCATTGGACCTCAGAGACTAAAACAGCTCTTCAACCCTGTC
20	65221152	AJ496641-007.SP6-473	0.32	0.16	CTGGAGGGATTTTGAAACACAGGGAAGTAAGAAATTGCTACTRAGAGTTGAGCATCTGTTGAAAGTAGAAGTGAGAGTAGAGTGGGTGGAGTTGGGGGGA[A/G]GCCATTGGACCTCAGAGACTAAAACAGCTCTTCAACCCTGTCGGGAGCAGTGCCTACTCTGGGGCTTTAGGATGCTGGACTTTCTCACTAAGAAGTTCCT

20	67357191	SCAFFOLD297109_1203	0.47	0.04	TCATTTGCGGTAAAACCGCCATAACAGCACCACACAGTCTTCTGGTCACTAGAGCCTCATTTTCAGAAGTCACAGAAGACACTCTATGGCTACAGACAGC[A/G]TCCCACAAGCCCTTATGCTCTCTCTGCTCTCTGTCTACATCTGTTTTTCCGGAACAGGTTACCTTGGCTACATCCTCACGCCTGCAAAGTCTGTCCCCTG
20	67538813	BES8_Contig583_1103	0.35	0.34	TCTAGATAAACCATCAGCAAGAAGTCCTACTGCGTCAGATGGCATTTTCTTTCCAAGACTGCTCACATTCCTTCTCTCCAAGGAACTAGTTAACATGTAA[A/T]TCTGCCCCAGATATTCTGGGATGAATTTAACCTGAAAGTGACTTGCAGATAATGAAGACAAAGATCTGCAGGTCTGGGGCCATGGTAAAAACTCAACCTC
20	67977148	SCAFFOLD150139_6440	0.43	0.38	TAAAAGCATGCAGGTGCACAGCTATACCATTGAAGCTCAAGGTATAAACAGCACACTACATACATAATTTCTAACACAATTTCAAATGAAAGCTCCTCTG[A/G]GAAAATTCTTAGCATATTTCATATTTCCCTACTTTATTTTCTGAAACTTAAAAATAATCAAAATCAAAATAAAGTGATACCTCAATGTATGATATAACAA
20	68097790	SCAFFOLD10964_8800	0.18	0.19	GAAGGGACTAGTGGCTGACAGGCTACTGACCTGAACATTTGCGGAAGGTAGGACTGTTCTGAGAAACGTCTGTTTTCTTGTTTCTCTTAAGGGAAACAGG[A/C]AAATGCTTCTGAACTTTCTGTAATAAAGTTGATGTCTTTTATTTTTTTCTTTTAAACAGCCAAAGCTTTTCCTTTGGGGAAGTTTGAAAGATTTGATGGA
20	69111189	SCAFFOLD146743_398	0.08	0.12	CTCTTGAGTGATCAAACCTGGTCCCTGGTCCTGAAGCTAAATCTTCTTCGGAGAACACGAATCTGACATCGTTCACCATAAGCTATCATAACTCAATGAA[C/G]GTATGAGCTATGCCATGCATGGCCACGCAAGATGGATGGGTCACAGTGGAGACCCTGACAAAACTTGATACACTGGAGAAGGGGTGGCAAACCCCTCCAG
20	72269003	SCAFFOLD151_26514	0.46	0.40	CAATTTATCATCATATTCTCTTAAACTCTGGGGTGTTGCATTTTAAGGTCCTATTAAATAAATCAAAAGAGCATATTATCTGAAATTAGGAGCCAGACAG[A/G]ACAGCAGCTAAAATTAATTCACCATAGTAATCTCCTCCTAAGACAAATTCCCCTGGGGGAAAACATTATATGCCCATTTCCATGGGCAGAGGTTTAAATA
21	1016751	BES3_Contig284_789	0.39	0.45	ATTCATCATCTCCAAAAACACTGCAGTATTCAAGATTAGTGCAAAAGGGGCAATAAACTGTTGCTGGATTTAATTAAAAACAACAAATCATTTGGTCAGT[A/G]GTAATGGATGCTACAAACAACCCATCAGGTGCATGGCTATTTAAGGAGCCAATATTTTAATCTCAAAACATCCAATTAGATGAGATCATGAAGCTGATGA
21	1017277	BES10_Contig479_1315	0.40	0.46	CATCTCTCCTTGGAATTTTCACAGGGCACAGCCTCAAAGGCATTCTTCTTGTCCCCTTCCTCTCTCCTCCTCCGTTGGCTCAGAAAACAGCTTAGGATTC[A/G]ATTATCTCAAGAATGAGCAGGGTGAAGGACGCAGTGAAGAAGGGAACCTGCTCACACACGTAAAATGGCCTTAAGAGGAATATCTCACTTTGAAATTAAT
21	3827974	SCAFFOLD211668_11002	0.48	0.43	CCTTATCAGTCATCTGAGAAACACAAGTTTTGAACACTGAACTCTAGTGTTTTGTTTTTCCATTAGATTTGCAGAGATTGAACTCTCCACAGCAGGGATG[A/G]GGGAGCAGGCATGGGGCACAGCATTATTGTGGACAGGCATAATCTATGCTAATTGCTTGATTCATCTCGAGCTGTCCTTGATGCATTTCATGATGACTTT
21	3874230	SCAFFOLD250149_26247	0.42	0.41	GAGCTGCAAAAGTCTGTAAAGCTCTGCTAAAGGAAATAGTGCCTACCTTTGGGCTGCCTTGATCACTTCAGAGGGACAATGGGCCCTCATTTACAGCCAT[A/G]ATATCCCAGAACCTGGCCATATGCTTAGGAATGAAATATCACCTCCACACATCATGGCAGCCTCAAGCCTCAGGGAAGGTATGCTGTGCTTAGTCACTCA
21	5702403	BES1_Contig646_1624	0.17	0.19	AAACCCAATCAGTGAGTGGCAAGTGACAGTCAAGCTGCACCTGGTGGCAGGCTATATAGTGGCAAGTGAGTTGATGTACTTCCTTGTACAGCTGCATCTG[A/G]GGCCAGGCTTTAAGGCAGAATCCAATATTATTTTACTCTGTGCATGGCCTGCCCATTATTTTATCTACCTCTTCCATCTGCATCTCTTTCCTGCACTGCA

21	6296337	SCAFFOLD271702_37443	0.46	0.47	ATGTTGTGGAAATCAGCTGGGACAGAAAAGGAACTTTTGGGGATCAAAGGAGAACACAGCAGTGGACTGTGGAAGGCAAGACAAAGTAAGAATTGCGTGC[A/G]TGATCTGTATTGCAGCCCCATGCACCCCAACCTGAGCTGTGGGCTCAGGGAGAGGACTGCAGCTGGTTGTGCAGATACAAAAGAGGGGACGTGAGTGAGG
21	6805542	BES3_Contig435_1235	0.34	0.35	GCAGATGTGGGACCTGTTGTAAGCTTTCTTGACAGTTTACATAGGCGTTTGTTTTTATTTTTGGTCATTGTAAAAATTCCAATGACCAGAAAGATCTCAC[A/G]GGCAAAATCCACTCGCTTCCCCCTGCGTGTATTTGTTGGTTTATAGCACCTTCCGTTTTCCTTTTATTCTTAGAGGCAGAAATGCCTTTCATCACGCCAG
21	6806087	BES3_Contig435_690	0.34	0.09	GTTTGTCCTCCAGCAAATGGTCCCCTTTCTCTCTCTCTCTCCCCATCACACCCTGTTTTTATTCCCCCAAAGTATTTGATATGTGTTTGTGCACAGTGGC[A/G]TGATGTACCGTGGAAGATCTATGGTCGCAATTTGTGATCCCTTTAAGAGGAATTGTTCTGTCTAGGAACAGGATTTTACCTAATTTAAGCTTTACCGAAA
21	9103919	SCAFFOLD266745_3555	0.24	0.19	TGCTAATTAGCATCTAGAGATATCATATTATAGATGCTTCTTTAAAAGCTATCAGCCCTCCAACTCTGCGTAGTGACATGGTATCTATCAATGTCTCAAG[A/G]TGCCTGCAATTCTGACAAAAGGCACTTTGAAGAACTGGCACGCACCTCCATATTTGCATTTTTGCATCTCATTATGGACATAATTGCATACCAACACAGC
21	9478201	SCAFFOLD140237_11719	0.27	0.29	TGAGCAGCTTTCTCTCCTAAATCCAGTGTGGAATGGAAGCTGACTTCCAACAAGTTGGCCCGGTTCCATCCAGGCCTGGTCTTTTGCTAGTGGGCTTAAC[A/G]TAAGGTTCTTAACTTCCCTGAAGCTCAGTTTCTTTAGCAGCAAAGAGGAGACAATGGCAGTTCCTAAGTCCGTGGGGAGGTGGGGTGGGGCGGCTTGTGG
21	9837926	SCAFFOLD150332_12151	0.09	0.13	TTTCAAATATTTTTTTTCCACAGTAAAACCAAATTCTCAGACTTTTGATTTGGACACATAAGGCACAGAGAGACTGCCATAGTAAACACAGAGCAAATAC[A/G]ATAGGTCCAGAGAGGAATGAACATCTTTACAAAATAAGAGGAGTTTGCAAAGAGATTAGAGCAGGGATTGCAAGAGGGAATAATGGCAGAGAACTGGGCT
21	11820092	BES4_Contig386_566	0.39	0.34	CACCCATGATCATAAGTAGCTTTCGGTGACCTCTATTCATCAAAGGTTTTACAAAACACTGTGTACCAGGCGCAGGCATTTATTAAGTCTCTGAGCCCAG[A/C]GCCCTGAAGTCAAAATTCTAGAAACTACAATGAGAGGGAAAGAAGAAGACAACAACGCAGTGGTAAGACGCCACTTCGCAGCACAGACATAAATACTGCT
21	11820201	BES4_Contig386_675	0.40	0.34	AGGACAGGAGAGGTTAGGAAGAATGGCCCAAGGAATGAAGAGGGGAGAAAAAATGTTGCCGGCAAGGATAAAGATATCAAAACTCTGAAAGGAACAGAGG[A/G]TCCTGGAGAGCAGTATTTATGTCTGTGCTGCGAAGTGGCGTCTTACCACTGCGTTGTTGTCTTCTTCTTTCCCTCTCATTGTAGTTTCTAGAATTTTGAC
21	12595937	SCAFFOLD75135_26559	0.23	0.13	GCACAAGCCACTTTAACATGAACCAGATGTTGAGTGCACGGCTGGGGGCTTTGAGCGGAGATTGCACATTCTGGAGGTTTGCTTAATGGGGTGTCCCACC[A/G]TTTTAGAAAGGGCAGCTAATTTCCTTGGGGCACTATGCAATACACCACGGAATGCTGGGGTTCTGGGTGGTTTTTCTTTCTCGGCCATCCTATGTCCACA
21	13254954	SCAFFOLD290205_6811	0.50	0.46	CAGACAAGTGGGAGCTGATGGTTTTAGTGCCGTGGGATACACCCTCCCGGTGAGAGGCTGGACGGGAACAGGAAGACAGGGAAGGAGAGTGCAAACCCAT[A/C]CTAGGCGCCTGCCCTGGGAACTTTCACCCATGTTAGCTCACTGAATGCTCCCTAAAGCCTGGTGGTCCAGTGATTAAGAATCCGCCTGCCAGTACAGGAG
21	14564149	SCAFFOLD247079_11949	0.14	0.14	GGCCAAAGGCATCAGAAGATGCAAGTATTAATGGCTCCTTGGGTGTCTGTAGGTCAGGGTGTCATCACATGGTCTTCTCAACACAGGCTCCTGCAGTGCA[A/G]GGACGGAATACGTGATGGAACCTCTGGAAGCTTCTGTTCAGTATCTTCAGTGTCAGGTGTTAAGAATGACATTTTGACAGGGGCAGATCAAACATTCTCT

21	14702969	SCAFFOLD35134_27036	0.34	0.37	CAGGCAGAGAAGAGCATCCACAACGAGTAAGCCACTGGTCCACCCCAGCTTGTTGCTGAGCCGGGTGAGCGCAGCATAAAGCCAACCAGGCCAGCAGCCA[A/G]GCCTCATGGAGTGTTGCTGTGCCAGACCCTTTGAACATGGAGTTTTAAAAATGACTTCTCTGCTCTGAGGTCCATGTATGATTTGAAGAGAAGGACAATT
21	18129749	SCAFFOLD140041_44992	0.02	0.06	GTCACAGAGTCGGACACAACTTAGTGACTGAACAACAACAACATTCATTCATTCATTTACCTGTTTTATTTGTTTTCAAAAGTACTCTTAAACATTGTGT[A/G]CTTTCCCAGGGCCCCGTTGTCCCCATCCCCTACCAGGTTCATTTGCTGTGACTCTGCATAATCAGCCTGGTTCTTAGAAGCCTTTGAACTTCTTTTGCTT
21	18130029	SCAFFOLD140041_45272	0.12	0.16	AGAAGCAAATAAAATGAACAGAGCCACAAAATTGTGTTGGAGCTGACAAGAGTCTCAGAGACCCTCTGTCTTAGGCGTTGCAAAACACTAGCCGGTGAAC[A/G]TACTTATTTGACACTTAGAGTTTTATTTATCATTCATTCATCATTCATTCATTCAAAAGGAGAAGAGGTGGCAGAGAATGAGGTGGTTAGGTAGCATCAC
21	18130172	SCAFFOLD140041_45415	0.48	0.05	GAGGAAAATTAAGGCTCCTCGCTTATACAGACATGCAGTCTGGTAGGCTGTCTCTTCTATTATCCCTTAGATGCAAATGGATTCATTCTCTCCTGGAATT[A/T]AGAGGGTGCCCCTAACTCACATTTTAGAAGCACATTTATTTTAGAAGCAAATAAAATGAACAGAGCCACAAAATTGTGTTGGAGCTGACAAGAGTCTCAG
21	19103797	AJ496767-048.SP6-493	0.39	0.47	GTATTCCTAGAAGTGAAATTCATGGTCAAGGATGGACACATCTAAGATTTTGCTAAGGTACCACCAAAGTGCCCTGCAGAACATACTTTAAAAATTTTTC[A/C]CTGTCCAGTCAGTAAAAAAAAAGATCTTTAGATTAGCGGTCTCCTTTCTTTTTTGTTAGGTAATGTGTTTATTTGGGTTAGTGGCTACTGGTTAGAGTGG
21	20586553	SCAFFOLD155320_17634	0.50	0.39	AGAATCTCTCTTCTCAACCAGGCTGAAACCAGGGCCAGGGTCAGCCTGGAACCGTCCCAGTTTCCAGGGTGAATGTGGGACTTGAGGGCTAACAGGAGCC[A/G]ATGGCAGATTTCTGAGCACATGCAGGGTGAGGACAGGTGAGTGTAGATCAGGAAATGACAGCAGGTCCCTCAAGGAGCCACACACACCAGGCTTCAGGCA
21	20789000	SCAFFOLD275035_19294	0.31	0.37	TCTTCCTCAGTTTTAAAATCTGTGGGGGGAAAAAAGTGAAACTGATCACCAGATCCAATGGAAGGCGTTGTCTTTTATTTAAGCCAGTGGTTGTCAACCG[A/G]GAGGTTTTGCTGCTTAGGGAACATCTGGTTCTGTCTGGGCTGCCGTGACTGGTGGGGAGAGTAAGGATACCACTGGCATCTCATGGGTGGAGGCCAGGGA
21	21884591	SCAFFOLD317495_539	0.45	0.49	CAGCTGTGTTATGGTTGGATGAAATTCAAGGTGGAATCTGGCAGTCCAACAAAGACACCCAAGAAGCACAGAGGTGTATGTATCGGTTTGTTTTATTTCT[A/G]TTCTGATTAAGTGGGGAGTGGCAAGGTGTTCCATAGCTCAGTACTCAGCTTGACCAAGATAAAGGGCTGGGGATTCTCTGATTGCCTAGTGGTTAGGATT
21	21884788	SCAFFOLD317495_736	0.43	0.23	GCTGACTCATCCTGGGTTTCCTATAAACCATTAAGAGCAAAACAGTATTAGAGGCATGAAATCAGGGTACATGCCATGATGGTGGCTTCTGACACTTAAT[A/G]AGAAACGGAGGAAGAGGTTTCAAATATACAAGACATGTGGAATAATCAACTGGAAGAAGCCAGCATCAAAAACTGGTAAAAGGGTTACACGAGAAATACC
21	24409362	SCAFFOLD316799_27843	0.30	0.27	GTGTCTTCTGTGTGTTAGGCACTATTTTAGTTGCTGGAAACACAGTGGGTGAGCCAAGCAGACAAAAAAATCCCCACCCTCAGGAAGCTGATATTCTAGC[A/G]GTGCAGGCAAATGAGTTCGCCAGGCACTTTGCCCACATTTTCAGTCCCCAGTTCTTTCCCTCTTTTTCCTCAAGGTTGGGGTGCTTATTAGGTGATTCCT
21	24827395	SCAFFOLD141275_7008	0.43	0.39	ATATTCTGGCATGTGGAGGCTGTCGGCAACGTCCATTCCCACCAACCTTGTATTGGGGTTCAATCAGCTCCTGGCTCTCTCCCACATTTACTGTTTGTAG[A/G]CTTTTGTGGATGTCAACTCTTACTGGTGGGATTTGGTTTCGTGCTATCGTTTTGATTGTCTTTCCTCCTGTAGTAATTAGGGATGCTGAACATCTTCTCC

21	25147257	SCAFFOLD141409_4380	0.48	0.48	ATTTAATTGACTCGAACCAGGTGTGGCCTGTGCCCCTCCAAGTGGCCAGGGCCCAACTCTGGGAGCAGAAGCGTGGGAGAGATAAAGGACCAGCGACCA[A/G]CAGACACTGAGAACCATTATTGCAATGGTTCAACCATGAAATCATGAATGCAGAACAATGGCGATGATGGTTAAAGTAGACAAGAGAAGACACAGGCAAG
21	25247691	BES2_Contig241_586	0.39	0.41	GCCCACCTTCTCCTGGTCGATAGGGAGAGTGCCTCTTCTGGCCAGACGTGGATGGGGAGCCTGAGTCTTCTCGTAGGCTGCCTTCCATGAGGCCGATGCC[A/G]AAGAGACCGGGATCTTCACAACCTGTTCCGTGACAGTGTGGACGAGGCTGGCATCTTTGCCATCAATGCTGCTGGTTCTGGAGAGGGGGCCCCTTCTGGG
21	25897834	SCAFFOLD125212_13312	0.49	0.49	GTCTCCCCACAACCACAAAACAAAACCAGAGCTCCACATCAGAAGTAGACCACGCCCCTTTCCTTGGGGCTGGTTATAGGAAGAGCTGCATTCACCAGCA[A/C]CTGACGGAGGAGAGTTTCCTAACCAAATAAAGATGAGCACAAGGGACTTCCCTGGTGGTCCAGTGGTTAAGAATTTGCCTTCCAATGCAGAGGATGTGGG
21	26401305	SCAFFOLD86821_4075	0.23	0.34	AAGGGCAGCTGTTAGAGAGGTTGGCTGCATTCTGAAATATCACCCCCGAGGCTATCAAGGACAGAGGGCTTGGGATTCTGGGTGGCGGAGCCAGCCTTGC[A/G]GCCTCCAAACACCAGACAGCGTCTATGTGACTCATAGGTCTTACCCACAAGGGAGCCTTCATTTCCTTTTCCAGTCCGTCTAAAATACGGCCTTGAGGTA
21	29098079	SCAFFOLD126032_4723	0.33	0.24	TCCATTGGTGGTTTATGTCTCAAAATGCAACAGATTTCTGTGTATTAATTTTGTATCCAGCAACTTTACCAAATCAATTAACAAGCTCCAGTAGTTTTCC[A/G]TGTCTTTAGGATTTTCTCCATACAGCATCATATCCTCTATAAAATGGTATTTCTGTGGTGAGTGCAGACAGGAGAGGGGAGCGAGGCCAGGTTAGCGTGC
21	30693086	SCAFFOLD317015_11758	0.05	0.21	CACCCTTGGCTCCTGCCCAGGGGAGGAGCCTCAGAAACAGCTGTGTATAAGTTATAGTCACTGTCAGGTGAGGAAAGAGACGTTCTGAGACGCGACGGCC[A/G]TGTCCAGGGTCGCACAGGCTGAAATCAAGTCATGCTAGCTCTCAGCCCCTTGCCCATCGCGGCCCACTCTCCCACCCTCAGGGGCACCAGCCCAGGCAGC
21	33209686	SCAFFOLD205022_32716	0.27	0.22	TCCCCGCTTCCACCACACAGACGTCCGTCCCAGCCTAGAGACCTCAGAGGCTTGGCCCGTCCTTCCCTTCCCTTACCTCACATCTCGATACTTTTCCTGC[A/G]TGCAGCCCCTCCTGAGAAACGTGTCAGACTAAGCCCCCTCACCCTGAGGACGGAAGGTGTGGGAGGTGGTCACCCCTCTCAAGAGACACAAGAGGCGACA
21	35150184	SCAFFOLD311908_14984	0.49	0.50	GGAGTATTCCTCCCTAGTACCGTTAGATACCTATGTACAGGAAGTAGTGATTCAGGTTAATGTTTCATATTAACGAATGTATTTGTAAAATGTAACAAGA[A/G]CAGTAGTTAACTTTTTCTAAGCTCTGAAAAAAAAAATAAAGATTATGTGTTAGACTTAAAATTTCAGTGAAATCTGTCTTAATACTACTTTTGTGGCTA
21	36102176	SCAFFOLD5077_14499	0.41	0.15	ACATCTTTTAATTTTTGTCCTTGTGTGGACCCCAGGTAACTTAGCCAACTAGAAATGAGAATGAATTGTTTGCTCCCTGAGTGTTCTCCCTGAGTTCAGC[A/G]CCAGTGATTTAGATCTGGGCACTGCTGTCTCTTTGTCCGCTGATTGTACTGTTTGCAGTGCTCAAAAAGAACGATGCTTCAGTATGAACCAAAATGGTGA
21	36295396	SCAFFOLD231073_11510	0.49	0.49	GATAGGATTGGATAAACAAACCTTTGCCACAAAATTTTACTGAAACAATGAACCTCTTATTCTCAAAATATCTGCACACAGTTAGTTGGGTTTAACATAA[C/G]GTTTCTGACCCAGTCCTGCTGCCTACGGTTCTAAAGGCAGCTGATGAAAACTTGGGACTGAGATTGGGCTTGAAGGAAGTGACAGGATGGACTTCAAGAA
21	40229689	BES8_Contig395_800	0.30	0.38	ATATAATACTTAGGTAATTGACTAGGGCTACTCATACTACTAAAAGACATTTAATCTATGAAAGGTACCCCTTATTTGGTTTGATCAAAATAACTGAATC[A/G]CAAGGGTTGACTAACAAGCTTCTTGATGAAGGTGAAAGAGGAGAGTGAAAAAGCTGACTTAAAACTCAACATTCAAAAAACTAAGATCATGGCATCCAGT

21	41873570	SCAFFOLD131201_14506	0.11	0.15	ACACATGGGAAGTTGTGTGAAAAAAAAAGGAACTTAAAAATCAGTGTGTGCCAGATTAACTTTTTCAAACATGCACAGAAGAAGCCTGGAATAACGAGAT[A/C]TAAAATCAATAACAAAATAATTTCTCAGAGATAGAATCATTGACAATTTTTTCTTTTATGATTGTGTGTGTATTTGAATTTTTTTACAATGGACCCCAAA
21	42582881	SCAFFOLD153044_4148	0.37	0.25	TGAACGATTGATCTGATCTGATTGCAGGAGGTGGAGCTTAATTCCCCACCCCATCTCCCATCCATTTGAGTGAGCTGAACCCAGTGATTCACTTTCAAAA[A/C]ACAGAAGATGGGAATGGAAACTAGTAACTTTAGGAACTTCCCTGGCCGTCCAGTGGTTAAGACTTCACCTTCTAATCCAGGGGGTGTGGGTTCAATCCCT
21	48174745	SCAFFOLD280454_16730	0.20	0.12	GGTCATATTTTGACCATAGGTGGATTCCTATGCAAATTCATCTTGAAAGAACTGGTTTCCTATTGCAACCCAGGAAGAACTGGGAAGATCACTTTGAAAC[A/G]TTGGGCAATGTTTTTTTTTGTTTTGTTTATCAAGCAGCACAGCTTTCTACCACTCCAACTTTTTGACTTACCATTCCCCTCTTCTCTATTTCCCCTTTGT
21	50527043	SCAFFOLD125069_19183	0.19	0.18	TACTTTTGAATTTTATCCTTCAAGTCATTGCTCTCAGAGGACCCTTCTAAGAGACTGTGAGTGACTGGAGGAACCCATCAACACTAGGAAACAAAATACA[A/C]ATCCTCTTGAAAGTCCCATCCCTTTTATGGGGAAGCCAGCGGATTGCTGATCAACTCCCTGCTGCCACAGGCCAGGGTCTTGGGAAGCAGATTCTGAGAT
21	50773070	SCAFFOLD200171_10366	0.38	0.40	AGGAAGAAATGTATATTTTGGATTAAATGTTCTAGTTTTAGAAGAACATTAGATGTGGGACTCTTTGGTTTGTAAACTCTTTCCTATTTTACCTAAGTTT[A/T]AAAGTGTGCAGATTTATCATCAGTAATTAAAGGGGGTAAGACTATTCTTTGTTGAATATGGCCAGCTTTTTGAATTCTGGGAGCAAATTTTGCATCTTTC
21	50773122	SCAFFOLD200171_10314	0.43	0.39	ATATCATTAACACAAATGTTATTAGACTTCTTGGTCATGGGAGAAAATTCAGGAAAGATGCAAAATTTGCTCCCAGAATTCAAAAAGCTGGCCATATTCA[A/G]CAAAGAATAGTCTTACCCCCTTTAATTACTGATGATAAATCTGCACACTTTAAAACTTAGGTAAAATAGGAAAGAGTTTACAAACCAAAGAGTCCCACAT
21	52424030	SCAFFOLD145703_5705	0.31	0.42	GGGCTAAAGTCCACAGGGTTGCAGAGTCAGACATGACTGAAGTGACTTAGAATGCACAGACGTATTTTGGGGGCAGAATCACAATAAATGTCTAGTTCAA[C/G]TCTCAGGCAAGAATAATGTACATCCAGTTTCAAACATTACATGAAAGAGACTTCACAGCTTTCCCCTAAATGCATTTGAAGATAATTTTCTTGTTGTTTT
21	55708557	SCAFFOLD141316_865	0.28	0.29	GAAGCTGCTTACCTGGAAACAGAGAGGTAGCTGTGGGAAACAAGAAGCTGTGGGATGAGGGGGGAACGAAATAGAATCCTCGCCTATTTGAACACTGACA[A/G]GGCTTGCCATGCTCCCTTCTCTGGCTTATTCCCTCTCCTTATAGAAATATTCAAATCACCACATCCTGGAAGCACCTCCCCTCTACTCTTCTATCAAGCT
21	55751392	BES5_Contig592_1191	0.37	0.35	GTTTTTCTACATTTTCCTGCTTACTTGTTCCTTGGGTAGTAAACAATGATTACATCTGCCCCAGAAATAAGCTTTAAAGAAATAATTCTTAACGAAAGAT[A/G]TATGTCAGATTTAACTGGGAGTGTTTTAACATCCCACACCTTTACTGTAAATCTATAGAATTAGGATTTCTGAAGATAGAACCTGGAAATATGTATTTTA
21	57138502	SCAFFOLD135896_7763	0.12	0.18	TGCCTGGGACATCCCATGGACGGAGGAGCCTGGTGGTTTACAGTCCATGGGGTTGCAAAGAGTTGGACAGGACCGAACACATGATGGATTGGTTAATTGG[A/G]TCAAGCATGTGACAGTCCAGGGTAGCGGGTATTTGATGACTTGTCAATTTGATTCAGTTCAGCTAATATTTGGGTAGTACCAGATGGTGATGGGCCAGGC
21	59304091	2279-422-W	0.14	0.24	AGATGCCGTTTATTCAGGGGAATTACAGGTGCCCAAGAAAACAACTGCGGTTCAGTGTTTGTGAACGGAGTACTTGGGTGGGGACAAGGCACATGAAAGC[A/T]CCCCTCAAAGTCCTGCTCCCCAAGRAGCTTAGTATCTGGTTCCACAAAAAAGAGGGGAGCCAAGGAAACAGAAGGCAGGAAAATCTCTCCCCTGAGGTGA

21	60133932	SCAFFOLD133_10823	0.18	0.21	ATAGTCCAAAGGATTTCTTCACCTTCTACTGGATCAAAGCTCAGAGAAGCTATTTGCCCAAGGTTAATAGTAGAGTTGAAATTTTCATCTTGAACCTATT[A/G]GAGAAGCTCTAGGAGACCTTTTCCTTAGCGCCCAACAAAGAGACTGGCATAGCACATATGCTAAATAAATGTCTGTGATAAATAAATTAATAAATGAATG
21	60133991	SCAFFOLD133_10882	0.17	0.21	GAGTTATAGCTGGCAAATCACTGGCATCATGGAAAGTCCATTGAAATCCAGAGATCATTCATTCATTTATTAATTTATTTATCACAGACATTTATTTAGC[A/G]TATGTGCTATGCCAGTCTCTTTGTTGGGCGCTAAGGAAAAGGTCTCCTAGAGCTTCTCCAATAGGTTCAAGATGAAAATTTCAACTCTACTATTAACCTT
21	60437862	SCAFFOLD145027_16638	0.38	0.35	CCAGCTGAACTCTTGATTACCCTGGATACCGTCTGCCAGTAACCGCATGAGAGACCTTAAGTGAGAACCTCTAAGCAGAGAGCCCCGTCAACCCCAGCCT[A/T]GTTGGAGAAAAAGCATCGCTGTTGCTGTCTTAAGTCACTAAGTTGTGTTATGCGGCACTAGATAACCAGAGCGCCAGTTTCTTGTCTCTGCCTCCCCAGG
21	60753739	SCAFFOLD85234_10372	0.43	0.41	CATAGAAAACATTTAGTTTAGGGTCTGGCATATAAGAAATGCTGGATAAATGGAACGTATTACATTATTACACAGCGTAACATGACTACATGATCTATAA[A/T]AACTTGTGCATAGCATTATTATGCTTGACTGTTAGTCACACTCAGCATCTCATTTATGATGAGGCTCATAATCAATTCTGGCTCAGCACTGGGGATACAC
21	63769906	SCAFFOLD255533_15525	0.44	0.45	TAGAGTAGTTTGCCATTTCTTTCTCCAGAGGATCTTCCTGACCCAGGGATCAAACCCTAGTCTGCTGCAGATTCTTTACTTGCTGAGGCATTGGAGAAGA[A/C]CTTTAGTTCAATTGAATTCCAAGCCCTGCCCAATGCCAAGTCATGACATGGGCTAGAGACAAGGCCTCTGAGACTTGGTTTCCTCAAATATAGCACAAGT
21	64230124	SCAFFOLD311324_1275	0.31	0.39	AGCTGTTTTGTTAAGATCTGTCTCCCTGGACTCTGAACTCCAAAAGGAAAGGGGACATATGAATGTGGTCACCACTGCACACCCAATCTCACGCCTGGCA[C/G]GTGTCCTACAAACGCTCTGTAAACACAGTCAACATCACTTGGCTAATGAGCGAGACGTTGAACCCAATTCTGTCTGTCTCCATAGACCAAGGCTGTGACC
21	64432198	SCAFFOLD382156_497	0.17	0.26	GGGCAGCCCCTGACATCCTGGCACTCATCGGTGGCTGAGCCCTGTTCTGGAGGGAGGTGTCCGTCACTGATGGATTTTTTTTTTTTTTTGGTGGTGGGCTG[A/G]GGGGAGAGGATGCTAACACATTGGCTGCTTGCTCTCCCTCTGCCTGTCCAGGGTGGCATTTCCCCTTTTCGAGGCACAGAGCTCCCTAGGAGCCCCGGAT
21	64675970	SCAFFOLD35342_23666	0.17	0.22	CCCAAGAATGCACCTAAAGTGGGCAGGAGTGGCCACCTTGGCAGCCCCGCCTTTCGGGCTTTCCCAAGTATAATGGTACCAGCGGCATGACACAATTCAC[A/G]TTATGAAAAGATCTCACAGGTTCTACTCAGACCTAGACAGGAGAGATCAAAAGTGCAGAAACCCTCTGAAGGGAGGACGGTGTCTGTCATCACTGGAAGG
21	64678203	SCAFFOLD35342_21433	0.10	0.14	TAACTATAGCAGCAGAAACATGCTGCTCAAGAGTTTCCGTGATGGTAAAAGATGTGGTCCACCTTGTCTAACCCACCAGGGCTAGAATGACGCACTGAAT[A/T]CTCACATCAGACTGTCTGCCAGCATCAGTCCCAGGCAGAGTCAGCGCGCAGAGCAAAAGCAGGAAGCACGCAACCCCAACGGCACCAACAGCAAAACCCC
21	65511048	BES10_Contig553_546	0.43	0.39	CACCCTAATGAATTGGGCCATTTCCATGCATTCTCAGGAAGTGTACAGAACAAGGGTTTTCATTCTCCTGGAAAAGTTTGTCTTGGGGAGGCAAGGTGGC[A/G]TGTTGGTGGGCACGCAGGTAGTTCTGCAAGGGTCGGCTGAGCACTCCGGCACTTCGCTCCTCCGGGCTTCGACGCCGCCCTCCGGGGTGAAGCTTCCTCC
21	66337412	SCAFFOLD255104_14760	0.28	0.23	GATACCCTGAGTTTTACTTATTTATTTTTCAACAAGGCTGTGAACTATGCCAGGTTGAGGCTCAGAAAATTGTCAAATAAAAACTCTGTTATGCAAGCCC[A/G]TTTGCACTTAGGAAGGCATCTCTGAAGACCACGGTCAAGAACCCCTCTAATCATCCTCTGATCTTTGGGGTGTGTGATCATGTATCACCTTAATGCTCGA

22	2945540	SCAFFOLD103644_2942	0.30	0.26	CTGTCTAGTCCCCAGAGAACCCCATCCACCAGCAAGAAACAGGACACTAGGAATCTTTATGTCTTGAGGTATCAGAAAAAAGATCTCGGATGTAGAATGA[C/G]GGGGAAAGGCTAGAAAATGCCGGGTTGTGCTATGTTTTGTTCATTCCCTGTCTTCTATTTTTAGTTTCCTCCAGGAGCTAAAAGACCACAGGCAACGTTT
22	3945360	SCAFFOLD225316_11410	0.40	0.40	GCTCTCATAAAAATGATCTGGGATTGTCCTTGTCAGCATTCATCGGGCATCTCTCCATTTTATTCTATTAAGGGGCATGAGAGAGCAATGCCAAGCTGAT[A/C]GATGTGCTGGAGAGTCTTACGGCTGTATTTTAGCATGCCCTGTATTGCAGGAGTTAGGCAAATGTAAGAGGAATTTACTACTCACAGGGTGATGGTTCAC
22	4471280	SCAFFOLD135781_9137	0.42	0.42	CATGATTGCTAAGACTTCCTTTTTTTCTGTCATTGGTTCTAACCTCAAATTCTCAAGTGATCCCATCTTGGAAATAAATCCAAGTGGTGAAAGTTGAGGA[A/C]TGAATTAGAGATCAACCAGAACTTTGGGGGAATGCTTTGGGTTAAACCCAAGTTAACTAAGTCTGTGATTTTTCTCTCTTGTAGTAACTGGTATGGTTAC
22	5747280	BES4_Contig438_715	0.22	0.19	CATGTGGGGGATTCCCTTTGATTTAATCATGGATTAAGATCGACTTTGCAAACTTTTGACAAGAAAGAGCACACAGAAATTTCTGGAAGAGATGTTTGTG[A/G]GGAGGCTGGTCTTCAGATAAAAATAGAAACAAGGCGGAACGCTGACTAATTAACAAACCTAATTATAGGGATTTCTTACAGACAAATGCTCATGTCAGCT
22	5778362	SCAFFOLD146281_2664	0.23	0.27	TTCAATTCTGTGAGCAAATGTGGTTGGAAAAAAACTTATTAGCTTAATGTATAAGAAAAAGCATCCTTTTTGGTGCCCAAATTCTGCCTGTGTAGAACAA[A/C]CAACCAAAGGATACATAAAACTCCAGATGATGCTGGGAAACACAATTTTGGAGAAAATTCAGGCAAGTCTTCAGATCTCAGTGTTTGAAAAGAAGCCCAT
22	7347863	SCAFFOLD115443_13923	0.13	0.10	ATGGGCATCAAGGCAATTATCTCACCCAAAGGTTTTTGAGATGATGGGCAATTTCCTGTTTTGGTGACACTTGTTCTCCCATCAGCTTAACTATAACATG[A/C]GATAACCATGGAGCTTCTGCAGAGTTGTCCTACGCTCATGAACCAACAGGCAATGATAATAACAGTCAAAAGAGAAGAGAAACTTCCATCGTTTAATTAG
22	9799397	SCAFFOLD20754_2588	0.01	0.07	TACCGTGAAAAATGTTTATAGCTACTGTGAATTCTGGAGCTTGATGACTGCTTGGGAAGACTGGATGGGAGGGTTAGGGGCAAGTGGCTGGACGATGATG[A/C]AGCCTTCTTGCTGGGCATTAAGGTATGGGTAGAATTTAACTTCTCACTGTTAATAGGGAAGGCTATTCTTGGTACAAGAAGCAATTTACAGAAAGATGTG
22	10529810	SCAFFOLD181989_3968	0.33	0.37	AAAATAATTATACCACAGAAGTTCTCCCAGAGGACTGAAAGTTCTGAACCCCACATCAGGCTTCCCAACCTGTGGGTCTAGCAATGGGAGTAGGAATCCC[A/C]AGAAAATCTGGTTTTGAAGTTCATGAGGGTTTGATCACAGGAATTCCACAGGACTGGGGGAAACAGAAACCCCACTCTTGGAGGGTGCACACAGGATCTC
22	11039315	SCAFFOLD190049_10845	0.16	0.42	CAAAGAATGTGAGTTTTTGATCCAATAATGTAAAAACGTACCAAGAACCACTGTGATAGGAGTAGCCCAACACTGGGGATTGAGGAGAGCTCCAGATTCC[A/G]CTCTCTGGCCAGGAGCAAGTCTTGTTCCCTCTTTTCTTAAAAAAATTATTTATTTTTAATTGGAGGATAATTGCTTTACTTGTTCCCTCTTGGGGCATCA
22	12964817	SCAFFOLD120466_4911	0.47	0.39	AAGTCTGCGCATCCCCAGAGTCATTGGCCAACAAGGTCAGTATTTGCTGACTGAGTAAATAACGAAAATATATTGAAAAAATTATTTAGAGGCTTATTGA[A/G]TAGTATGGAAGTAAAGACTCAGAAATGAAAATGCCAACCAGCGGGCAGAGGGTCCAGATGACAACCTGAGATGCTCAGGGTCACAAAACTCTTTCAGAGA
22	13373452	SCAFFOLD295799_12595	0.18	0.35	AAAGACAAAGAAGAACCTCAGTTCTGCCAGACCGATGACAGGAACTCATTCATTGCCAGGATGGTTCTACAAAGACCCAAATTACAAACAGGGTGGAAGC[A/G]CTGATTCACAGTCTGTTTTCAGCAGCAGAGCATTCATGACACATGGTCTGCCTTTATCAGGACAAAGAATACTTGTCCAGATTTTCCCTTTTAAAGATAA

22	13373801	SCAFFOLD295799_12944	0.42	0.42	TCTGATGACAAAGGAATAAGTTACCACTTCTTGATTTGAGGCAGTTGGGATTCTGCTCCCCTGGCCTCCACTTTGTCTTTGATTGCCGGGGAAAATGGTT[A/G]TCTTGGCTAATGTTAAAAGACAGATCTAAGTGTGGTTGGAAAGTCTGTTCCAGGCATTGTCAGAGCTGGACTGTAATAGTGTATGTAACTCTGGACCACA
22	17988809	SCAFFOLD115034_36047	0.04	0.07	GAAAGGTGAGGCTCACACAAGAAGTGATTTGCCCAAAGTTCCTGGGATCCAGGCTGCCTCCGGTTCAGGGGCCTGGCCAAACACAGTGCACTCGGGTGCA[A/G]TCCAGGGAACAAGGCACCCAGAACAGAAACACATTTACCTGCCCCCAGTTCAGCACGGCCTCAGCCACACCTCCACACAGGTACCAGCTTCCCTTGGCCC
22	18468369	SCAFFOLD316779_17258	0.32	0.45	ATCCTGTGGACAGAGGAGCCTGGCAGGCTACAGCCCATGGGGTCGGAAGAGTCAGACATGACTCAGCGACTAAACCATGGAGACATTTTTGATTGTCCCA[C/G]TGGGGGGCTCTGGTGTCTGGGTTACTGCTAAACATCTTACAATACACAGGACAGCTCCCTCCACAGCAGACAACCATCCAGCCCCTACTGTTGGTAGTGC
22	20915854	SCAFFOLD153099_2229	0.22	0.25	TTCTTATTTTATTTTTCTTAAATACTTTCACTATTATTTAACTGTACAAGCATTAGCAAATCCGAGGTTCAGTTTTCATTTGCCCAATGTTGAAATAGAA[A/G]TCGATTAATTTTTAAGTTTTGCTTTATTCTTATCTTTCCTTTTAATAATATTTGATTGAAGTTAACTTCCAAGTATCCCATTGCTCAGATCACACTTTGT
22	22225308	SCAFFOLD135112_7636	0.33	0.27	TCTGGCCCTTAATCCTTGTCAGCTGATTGCTCACAAGGCTCCTGATAAGTCTTTATGATCAAGACAGAAACCCAAAAGCACTGTGGAGTTTTCTGTGACT[A/G]TAAAGTAAGGTGATTAACAGAAAACTTCAAAACTGGCCCCCAAAAGATGGGGCACATTCGAATACTCTAGAGAGAAGCAGACAAAGTGGGGTGGGGGAGA
22	22366608	SCAFFOLD216765_5028	0.32	0.17	TCGGAGGAAGGAAAGTATATTTTCAGTTTCTGTTTTGTAGGTGGTTCTGAGATGGAGGAGGCTCCTCTCCATTAGCTCTCAAAGACTTCTCCTTGGACTC[A/G]GAAAGGTTTAGTCCTTGGGTTTAGGTTCTTTGCCTCCTCACTTTATAACTTCTAGAACTCAGGTCAGAGTAATCTTAGTTGGCTTCATATATATGTGTTT
22	22706393	BES4_Contig512_385	0.29	0.40	CAATAGGAGATGGAGGGCAAGCTGCAACTTCACTCACACCTGATGTTGATGGAATGCATGTTTGCATCTTTGACAGTTTGGAACTTGAAGGTTTGCATGT[A/C]GAGTACTTACTATGTTCTCGATAGTCACATACCCAGCTAAAAACTGAGAATTCTGATAGGAAGGGAGAGAGTGTTGCTGGACAATCTGTAGTCTGTGCCA
22	23047099	SCAFFOLD190836_5512	0.45	0.43	ACTACAGAGATAGAAAGCAAACACCAAGTAAAGGGCTGATCATGGTGTCCTTAGCAAATAAATGAATGAATAAATAGAAAACCACATGAGAGAAGATAAT[A/T]GATTTTAGTATTGTTTGTTTGTGAAAGAATGAATGTAATACCTTTTGGCCAATCACACAGGCAAATTAGAAAATTTGATAATCAAGCATTGCATTTGTTT
22	24032252	BES4_Contig382_1604	0.34	0.13	TTGCCCTCAAGCCTCTCCTGAGCTGAAGGTTGAGAATCAGAATAAATATAGGGGGCAGACACAGAAAGCGTCATCCCACAAGCTTGAGGGCATCTCAGGT[A/G]TGAAAGATGGCCACAGAACGGCAGAATATTAAGAAATCCTTTGATGGGTCAAGTATACAAAAACAGACGAAGGTCTGACAATTTTCCTGCGAAAACACTG
22	24272041	BES10_Contig779_1471	0.18	0.17	TTGCTCAAAACAGAAATCACACACCACTCAGAGCAAATAACCCTGTCCTCCCACCAACAGCAGAGAGCTATGTGCGTTTGATGTATTTCTCTTTGTGTGT[A/G]TACACACATTTGCAATTATTTTAATTCCATGTATTTGGGATGATAAAATATAGCGTGGGTAATCAGTAATTGGCTGGTGTTGAGACCGGCATGACTCATA
22	24416891	BES10_Contig320_647	0.13	0.15	ACATTCCCACCCATTCTGTTATCACTGCAGCGAGTGTGCATTAAGAGAGCTGGAATGGAGATCATTGTTATTAACACAGGTTGATCTTATGAAAGGTCAC[A/G]GCCAGATTGTGCATGTGAACATACTGTCGGACATGCCACGGTAAATCCGTACAATTCATTTTAGTCTCAATTGCTCTGGGAAACCTATTTTTTGCCAGTT

22	24510032	SCAFFOLD22355_816	0.06	0.08	CTTCTAATTCAACAAAGACTGATGACTTACAAGTTGAACATTTATGAAGGATAATCACAAGATGAAGGGAGCATTATTCCTAGAATTAACTATTTCAGAA[A/G]GGAAATGCTAATTTTTCCTCATTAAAAAGAGAGAACACAGATGCCACTCACTTCCATGAAAAAAAAAATTACCATAGCTCTAGAGCATGCCATTCTTTTA
22	24510032	SCAFFOLD130917_10918	0.06	0.17	GCAGATCTTCCTGACCCAGGAATCGAACTGGGGTCTCCTGCATTGCAGGCAGATTTTTTACCAACTGAGCTATCAGGGAAGGAGAAAACTGAAGTTGTGG[A/G]AAATTAAGGGTTCTGTCTGAGGTCACGCCTCTGAAAATGGCACAGCCAATCCGGATTGCCTGATGAGGATGGTACCTCCCTCACCATCCTTCCCAACTCT
22	24510190	SCAFFOLD22355_974	0.33	0.35	TGAAAGCCAGTCCTAAATTGAGGTAGGAGGGCATGCAGAACATGAATGTGTTAATTAGCCAGATAGCATTTGGAGAAAAATGTACATCAGTCTATCATGA[A/G]AAAGGGAAGATGGCAAGTGGCTAGCTTTATGCTTCCCACTTCTCACTTTATGTTCCCTAAAAGAATGGCATGCTCTAGAGCTATGGTAATTTTTTTTTTC
22	26347249	BES7_Contig485_1361	0.20	0.29	TTAACAAGTTTTAGGGATTAGGAATTGAGTTTGATTCTAAAATTTAGGAGCACGGATGTCAGCTTTGAATATTTTCCAAACATCCCATGACCATAGATTT[A/G]TAGAATGGAAGTGCTATTCCTCATGCACTATTCCCCACTGTCAGTCAGTCCCAGTCATGCTTGTCTTTAAGAAAAAAAGCATCCCAGTCATTTAGAACTC
22	28176365	BES3_Contig309_899	0.24	0.42	ACTCACCCAGCAAAAGGGGTATTTCAACAAAATTATGAGTCAAAAACAACAAAAACAAAAAGTTAAATCTGTCTTTAAGGTTGAAAATGCCTCTTTAATT[A/T]TTGTTTCAAAAGAAGCTGAAGGACATTTGATAATTGATTCTCAGGAAAGCTGGCCACACTATGAGAAGGATATGAAGCCTAGAACTTGTTAGAGCATCAC
22	29138273	SCAFFOLD25420_21809	0.11	0.21	TGCACTGCCTAAGAATGCAATACAACTTGTTTTTCTAACCAGCTAGATTGGTTTACGTGGTGAGATAAATATTGTGCTAAGAGCAACTGCAGCACATCAC[A/G]GAGAGAGAGATGTGGTCCCCATTCAGGCTGGCTGGGAGGGCTCCCAGCACCCCTCCTAGGATGCTCAGGACGCAGTGGCTCGCGTCAGCGGCTCCTTTGT
22	29837317	SCAFFOLD61390_3094	0.43	0.42	TAGGACCAGAAGTGTGTGCAGGCAGTCTTGGGGCCTTAGAAAGCAACATGGAAGGCCTGCAGGTTGAATTCATCCAGCTGATGCACCTAAAGTGTATCGC[A/G]TTTCTGAGAGAAGCAGCTGTTTAGACCCGTACCTAACCCTGTCAGTTTGTGGTGATCTCTAAGGAACCCTGCCAGCCCCGGATGGAGACAAGCCAGCTGT
22	29837722	SCAFFOLD61390_2689	0.27	0.24	GCCAAGGAAGGAGCCTGGCTCTGAGGTCATTGCCAAGTATGTGACCCTTGGCAAGTGACAAAGCTTTAAGAGCCTCAGTTTCTCCATTGGTAAAGTAGGC[A/G]GCATATATTTAACTGTATCACTATTAGGATAACTGGTTAAGGTAATGATTAAAAGAGTTACTCATAACCGAAAGGTAACAAGAGGAAGACTTCTTATCAC
22	29900388	BES9_Contig373_1108	0.23	0.27	ACTGAGATAGTGAATGAGTGCTTCAGTGGCTTAAGTGGAAAATGATAAATTATATTGTGAAACATCTGCATAGCTATGATACCTGATAGGAAAGTACTCA[A/C]AGCCAATAAATATCATTTTCATAGTGTAATATCAGAATGTAAATACACTAACTTTCCACTGCTACCCAAATTACCCCAAACTTAGTAACTGAAAATAACA
22	30866166	BES2_Contig475_1208	0.49	0.02	AGCCATCGCTTGCTTCCCACCCAGGTTCACTGGTTCACAGTGGCTGTCAATCACCAAGGTCCTGGACTACAAACGTGATACACAATGAGGACACTGTCAT[A/G]AAATGCAGCTTCCTGCCCCCAGATTCCAGAGTCTCAATCTGGAGTCCAAGGAATCTGCATTTTGACCAGCTCTTCCAGGGAACTCTGATGAAGATGCGCC
22	31775633	SCAFFOLD305629_22295	0.32	0.30	GTCCTTTGGTTATATACCCAGATGTAGAAAGGCTGGATTATTTACCTTTTGAGTGCCCTACATAACAAGTAAACACAATATGTGGAAAACAACTGAGCAG[A/G]AAGATCAGTGTGCTAGGAAGGAAATAAACTACCCATAAATTCTCAAAATCTGGATTCGTCAGTTGCTAGAACTCCAGTCAGACCTGAGCTGCTTTGAATC

22	31861006	BES7_Contig247_1147	0.20	0.13	TAAAGTTTTACATCCACATGTCTAATTTAAATTGTGCATTTTGTAACTGAAGCTATCAAAGATACACAAGTTCTGAAAGAAAGTGGTCAGTGGAAGAAAG[A/C]ACATCCTTCAACTCAATTACGCTGGTGGAAACCAGCATGTGATATGTCAAGATTATGTAGAATTTAAATTCCACAGATGGCAGAAAGTCATTTACTTCTT
22	34667325	SCAFFOLD75128_26545	0.21	0.33	TGCTTTTTTCATTTGGAGTTTAGTCAACTGCACTAGTTATTTTAACAAGAATAATGTAGCAAGTTGATGAAAGAAATGTTGGGAGGACTGCAGGTACAAA[A/G]AGAGAACCCTGACTAAAGCAGAAATTGTAACTGTAGGAAGCAGCTACCACCCCTGGAATAAACTCTCCAAGAACTTGGAAATGGGAGGAGGAATCCTGCA
22	35532306	SCAFFOLD280392_13668	0.48	0.04	CTTTGACCTGAAAGTCAAAGATGGCCATAAAACTAGACCAAACCAGGTGAGATTTGAAGACTGGTGGGGATACAAACAGGGCTGAAATAAAGGTGATGGG[A/G]ACAACAGGGCATCTTGGAAAGATACCAAAATCCTTCTGTCTCTCAACTCTGACAATTATTTGACTCTATGTGGACAGTTTGAACTTGAAGCATTCTTTTT
22	36640521	SCAFFOLD260699_13209	0.20	0.31	TTTTCAGTCCACAGGCTTCTCCTTACCTCAAGAAAGCAAATTATGCCCGTGTCCTTAACACAAACTAGACCTAATATGTGTCCAGATAAAATCAGGTCAA[A/G]GATCAAATCAATGACTATTAAGGACAGCATTTTATAAACCAATTCATTAAAAGGCAATGACATCTTAGCTCTGACATATGAACAATCTATCTTTAGAAAA
22	38395929	SCAFFOLD225781_14086	0.28	0.25	GACATAAGTTCCATGAGAACAGAGTTTTGCTCACTGCTGTATCTTCAGCATCCTGAAAGAATGCTTTGAATATAGTAAACACGGGAGAGAATTATGGAAT[A/G]TTGATTGCTTTCCTTTATTTGGTCACAGTTCCTGCAAGTGTCTCACAGTGTAAGCATCTCATTGTCCTGTGATTGTGGTGTTTAATTACTTGGATTTTTC
22	38526951	SCAFFOLD6161_13827	0.38	0.35	TTAAAAAAATTGATGGCACTGATATGAACAACTCCTGCCGCTGTTCAGAGAAGGTTGGAGCTGATGGTTGCTAAGCAGCCAGGGCCATCACTGGGAAAAC[C/G]GCCTAAATGAATGATCCCCAGAACAGGAAGGCAGTCCAAGGGATGAGAAATTAACTCTCTGTGGGACCAGGCAACTTAGATTCATTTCTCCCCATTTTAT
22	40139379	SCAFFOLD36354_2181	0.36	0.42	TAGAATATATTTGACTGTGGATACATCTATCAGATGAACAAAAGCAAAAAGAAAAAAAGGAATGAAAAATATCTATCTGTAATGCCCTCACAGCCTGGGA[A/G]TTATAAGATACGAGTATTAGCAGTTACAGTATTAGACTATGTTGACAAGACTTTGACTTCTGTTTTATTTGAAGAGGCACTGCATCGGGAAGCCTTTAAA
22	40639537	SCAFFOLD55042_15876	0.31	0.41	CATAAAAGAATACCAGTTCCATAACTTCCATGGTGGTCCAGTGGTCAGGGCTCCATGCTTCCAAAGCAGGGGTCCAGGTTCGATACAGATCGGCAAAGTA[A/C]GATCCCACGTGTTGTGCAAAGCCGCCAAAAAACAAATAAATACTCTTCTAGATAAGTTTTTTTGTTTCCCTATTTGCACACAGACCAGTGGCTGGAGAAG
22	40883081	SCAFFOLD96282_2582	0.20	0.28	TCCCTGTAAGGAAACCCAGTGGGGAATACTGGATGGTACTGAAAGCCACCGAGGGCACACATCCCATCTACACCCTCCAGGCTGCTCTGCTGTCCACAGG[A/G]GCCTGGTAGTCTGTGTTACATTTTAAAATGCCTTTGTGGGCTTCCTTGGTGGTCCAGTGATTAAGAATCTACCTGCCAATTCAGGGGACACGGATTCAAT
22	40957965	SCAFFOLD305862_2157	0.48	0.46	CTGACCTTTGAAACAGAGCAGATGATGTTAATCAGATTTGTACAACTTTAATCTAGCTCAGCCTCCATCTCTCCATGTAGTTTTTAAAATATCATAAAGA[A/C]GAGTCTCACAAAAGGGAATCTTTCAAATCAGAATAGATTAAAAAATGGAATAGGATGTATGACCTTGCTTCAAACAATGAAGTACAATTCAGACATGTAA
22	40958460	SCAFFOLD305862_1662	0.44	0.41	ACTCATAAATGACATCTGAACTCTGAATGTTGGTTTCATGGACATTTTGTTGACAAAGATGTAGAGGCATCTTAGTTGGCTAGAGTCTTATCTGGAAACT[A/G]AAGTGAAGGTCAGAAAAGTTAGGACTCCTGGGTTTTATTAACGTTTAATGGTAGGGGTCCTGAAGTTTTTTAATTAATGTATGTAAAGTCATTGTACAT

22	40994039	SCAFFOLD35454_8906	0.29	0.33	AAATGAACACTGCAAAAAGAATTGAGCAGGATTACAGGACAAGAGAGCTCAGTTCTACAAGTCTGAAATGAGCTGGAAACATAACAACAGACTCAGAGGC[A/G]GAAACAACAGCATGGGGGGGGTGGGGGGAGTCACACAGAAAGGGGACTGAGCCCGGCAGCTTTGGGGATTTTTGATGCTGGGACACACTCGGGCTAGAAC
22	41559358	BES9_Contig418_574	0.37	0.35	AAATAAAGATGAAAACTGAACAAAAAGTTTCCTAGTGCAATTGCATCTTCTCTAATTTTCTCAAGATTGCTGCCTCCTGGCTGTTTCATATAATATCCCC[A/C]AAATGTATACCAAGTACATATTTTAATACCTAGCTAGGTACATTTCCCAGAATGTATTTTTAAATAGGCTGGTTGTGGGAAGGCTGGCAAGATACTAGTG
22	41649041	SCAFFOLD235305_8110	0.19	0.19	ATATTTAATGGGCAGTCATAAGGCATTGAATTGTCCCTGTTATTTTTGACCTGCAGGAGACCACGGGGCACCTAAGTGTAGGGGAACAAATTAAAGGGAG[A/G]GGATCTGGCTGGATAGCTTTTAGACATTTGGGATGCCTATGCCCTTGATTTTTCAAATTGCTGCTGAAATGCAGCTCTAAAATTTGGCAGATGAGAGCTC
22	42415179	SCAFFOLD130705_13273	0.47	0.38	AATGGAAGCAGAAGGGCTACATCACATCGCATTGAACATGGCAGTATGGTAACAGTTGAGTGTTAACCATGGGTCAGCTTCGGCATTCACGTCTTTTTAC[A/G]CTCATGATTTCATGTCATCATCCCAGCAAACCTGGAGTCTAGGCCTTGCTGTCCCTGTTTTACATGGAAGGAAACTGAGGCACGGGGGTCGGTGGGAGCG
22	43302142	BES4_Contig493_2956	0.27	0.33	ACTGTGCTTCCTATCACAGTTTTGGTGAAACTCTAGATATTCAAACAATTATATTTTTCCACTTCATAAATCTTAAGCTCTTTAACTGGAGGGAAGAGAA[A/T]ACATCTATTCATTTTTAATACAATCATGTAACCTAACAAATGCTTCATATACAGCATAGGCCCAGAATTCATATTTACTGAAATGAATTTAATATTTTAA
22	43435178	SCAFFOLD140014_29121	0.03	0.08	CTATAGCATTAAAGCTGCTGTTGTTTATGCAAATGAAATCTTGACAATTTCCAGGAACTTTACCAACATTTATAAAGACCCAAAACGTTCTGCTTTCATC[A/G]CTGAAGTTGATATTTCCAATGGGGAAGGCTGCTAGGATCAGCTCAGATGGTGTGTGTTGGCATGAGGAAGGCACCCCATTTTCTTCCTCCTCCTCCATTG
22	43985505	SCAFFOLD145052_20697	0.20	0.34	AGCCAGATCTTAAGTTTTGTGGAGCAGGACCCTAGGACTCCACTGAAGACAGTGGGCAGGTTGAGATGGCTAAGCTGTGGCATTTAGACCCAGGTGTTGG[C/G]GGGGTGTGGGAGAGGTGTGGTGGCTTTTGGATCCCATTGTTGGGGAGTTGGGGCTCTCCTGGGATTTCTGCTCGGGGCACTTGCCTTGTCTGACCAGTCA
22	44203664	SCAFFOLD312433_15807	0.28	0.35	GGGAGAAGAATAGAGAAATTATTTCAAATATTGTAAAAGTGTAAATTGAAGGAGACCAAGTGACATACATCATCAGATAATGGGATATACAGAATTACAT[A/G]TATTATCCTCCTGACTGGAAAGGTAGAATATGAAAAATCAACTTTAAATAAATGATAAAGAAACTATATTATACAGAGAATGAATGCCCCATAAAATTTT
22	44896040	SCAFFOLD155592_8515	0.22	0.30	CCCGGGCTGCAGCCGGGACGGGTTAGCCGAAGGAACTACTTCCGGAGCAAGTCGGGCCGCTCCCTGTACGTCGCCATTTGCAACATGCACCAGTTTATCG[A/G]TGAGGAGCCTGACTGGTTTGAGAAGCAGTTCTCTCCTTTGCATCCGCCTCCTCTCCGCTACCGAGAGCCGGTCTTGGAGAAGTTTGACTCGGGCTTGGTT
22	46120882	SCAFFOLD211113_6232	0.18	0.27	AAAAAATTTTTTTTCCTTACCTGGATTATAATTGTAAAGAAACCAGATCTGCCAGTGATCTTTATGAATTAGGCTTATGATGCCTTTGTAGCTATTGTTA[A/G]ACATGGCACTTAAAAATGTTTTATTGTATGTATATGTGATTGTTTTATAAAATGGGGATTATGACTATGACATTTCTATCGCTTGACTCAAGGAAGGTGA
22	46690904	SCAFFOLD146363_8633	0.25	0.47	CACTTGAGGCAAAATAGAACTTCTTTGTTCAAAGGAAATAGACTTTTTAAAGTCTTGTAAAATAGACCCACAATGCTTTTTGGGACTCAAATAACTCTGA[A/C]CCAAAACGTTTTAATAAATTAAACATAAGTGGATTAGAAACAGACAGGTTGTCTTTTCATGAAATACACCCAGGCCAGACTCAGAAGGAGAAGCACAGAG

22	46691150	SCAFFOLD146363_8879	0.33	0.22	ATCTTCCTTTTCTTTCTGATAACGGAAGGGAGATGAACTCTGCTATTATTTCTGAGTCTATTTCTTTGTGCCTTTCCAGTATGCATCTCTGAATGGGGCC[A/G]TGTGTAACCCCTTGTGGTACTGAATTGTGTTATCCCCACCTTCAATGGGTAACCTTTAATCAGGCACAGGAGGTGGGGGTGATGGTGTGTGACTTGGCTG
22	48807460	SCAFFOLD40179_14392	0.49	0.44	CACACAGGCTTCTTGCAGACACACCATCCCAACACAGGGACACAGACTTTGCTGTGAATGCACACAGGCACACATGTGCATACGCTTGCGCATACAGACC[A/G]GGCCGTGGAGACACTTACGCCTGCACTTGTAGCCTTGCTTGTTGAGACCCCTGAAAGGCAAGTGGCCAGTCTGGGTGAGTGTGGGGAAGGCCAGGGATGG
22	51040567	SCAFFOLD70173_981	0.21	0.23	TGTGGGGTGGGGAAAAGGCCTGATGAGATCCTTTGTGGAGGCCCCTTGACCCCTGAGCTCCACCCCATCCCCCAACACCACCAGCAAGGGTAACTGACCC[A/G]GTGGTTCTGAGCCCTCAAAATGGATTAAGAGACCCTGAAGAACAAATCTCAGATGGGAGGCCAAGCTGTCTGTGCACATGGCAGGCAGCCATCTCTTAAA
22	51502019	BES6_Contig390_784	0.26	0.39	CTCTCCTACCACACTGTGAGCCCCTTGAGGGCAGAGCCTACCTCCTGAGCTTCTTTCTTCCTCTCATTTCACCCAGGGATAGGCATCTGATAGGTGTTGA[C/G]CAAAGGTTTCCAGAATACATGGATGAACTTCACTTTAAAGTCCTCCTACTTCTAAGTAGAATTTAACTGTAGAAATCCCTCACATATATGACTCCTCATA
22	54075034	BES9_Contig467_946	0.07	0.21	AGCATAGTGTTGCTTGAGAACCTGAGATCCACCAGATGGGGCTCTGTGTCTCCTGTAGCACAAAAGAGTATTCAGTGATTCTTGGCACCTCCAAGCTGAA[A/G]CCCCCGAACTCCACAAGAACCCTCTTGATCCCTGTGAGGGACATCATTATGCACCCCAAGTACTGGGGCCGGACCTTCATCATGGGTGATGTGGCCCTTC
22	54090671	SCAFFOLD65385_6621	0.29	0.22	AGGTAGAGAGACACAAGCTGAGCACAGGGCTACCCAGCCAGGCCCCTACTCCTGGATCACCAGATTCAGTGGGGGCTCCCAGGCCAAAAAGCAGAGCCCC[A/G]GGGACCGCTGGCCACCCTGACTGACCTCTGTAAGCAGTGTGCAGCTGTAAGGACCCACTGGCGGTGGATGAGGGAGCCACTGCAGATGCTCATGCCCAGG
22	55134751	SCAFFOLD255895_2064	0.31	0.49	CTATCTTGCTTTGCCAGCACCTTCAGTTTTGAGGTCACCACGTACCAAAATCTTGTGAGCAGAAAACTTGTGGCCCTCAAGCTTTTGAGAGCATGGAAAG[A/G]ATCAACCTGTCCTGCCTTACGTGTTCTAACTGTCCTCATTCCAACTGTGTTTCAGGCTCTCCAGGTTTCATTCAACTGCTCTTGGCTTTTGGAGAGGGTA
22	55536152	SCAFFOLD91128_4487	0.17	0.18	AGGAACATCTCTTTCTAGGGCTGCACGAATGATGAGGATCATATGTCAGCTTGCTGAAGCTGACAGCTAAAATGAGTGTGGAGAGGACGCGGAGTACAGC[A/G]TCTTCAAATTTGACACAATTAGCTAGAGAACTGTCAGCCCACAGGAAAAGACAGCCAGCTTGCTGAAGATGAGACCATGCCAAATTGAGCAGACTATAGG
22	56165120	SCAFFOLD115037_43056	0.26	0.31	ATGGCCTTTGAGGGCCCACCTAAGACCAGCAGACTGTGGAGTGAATGGGAAAGGGGAGCCTGTAAACTGGCAAGGAGGGCCTCGAGGAGCTTGGGGCTGT[A/G]CAATGAGGGAGCAAGACATCCCTGTGGAAGAGGTGCTTGCAGCAGGATTCAGGGTGGGCCTGAGGAGCCATGATCCTTGGCATCAGAGCTGACCTCACAT
22	57362459	SCAFFOLD105204_16908	0.40	0.43	ATCTAGAGTCAGTTGTTGAAAGCATCTTTCAGGCCAGCTTCGCAGCTATCATAGTATGTTCTTTTCCCTTATAAACAACTTTGTTGGTGGTATATTTTAC[A/G]TATCCTGAGATTTATTTATTGCAGGAATATACTTCAGTGATTTTTAGTAAATTTACATAGTGATACAACCATCATCATTAATCAATTTTAGAACATTTTC
22	60301983	BES10_Contig680_824	0.20	0.18	GGGACGGGGCATACCGTGTGCACCTCTAAGAGCTGAGGGGACCTGAGCAAGCTTCCTAGAGCTGCTCAGAGGAGCTGGAGGGGATGGCTCAGGTCCTGGC[A/C]GCTGAGGGTCTCCAACACAGCATCAGGGTGTCTCGGAGCTCGGAGCAGCCGGGTCTGGGCTCCAGCCCGGTCTGTGCACAAGCTGGGCCTTGAGCTCGGC

23	101057	SCAFFOLD59442_4840	0.01	0.03	TTGTTCATTTTTTAGTGCTAACAAACAAGGCTACACTCAGCAGCAAGAAATAAAATGACCAAATGCCCAGATATGATTTCAAGGGGCAGGAAATTTCTCC[A/G]TCCCTGGCTCACTGGCAGCAAAGTACCGTTTCCAAAGTTGTTCTCCCATGATCATAACTATATTTGAAGAAATAAGCCAAAGAACGTTAATTTCAACAAC
23	1310558	SCAFFOLD135282_16356	0.42	0.32	TGGAGCTGTGCTGGCCGGTTGCCACGCCGCTCCTCTGCTAGACCACAGTCCTTCAGGTCGGAGGATGAGCTCATTTGATAAGCTAACCAAGGTCAGGCTA[C/G]GGTTACTTTGCAAGAGAAAATTTCAAGGTAAAATAGGTGCTGCCAAAAATGCTTTTGCCTGTTCGGGGGGTGACTCACTGGATAAATATCAGATAATTGT
23	1507400	SCAFFOLD131821_4175	0.49	0.04	CCACCTGCCAAACTGATCTGTTTTATTGCTGGCATTTTCATATATAGTATTGTCAAAACTTTGATGCACCTGCCTGAGGGAGAGAAAGTCCAATCTGTGG[A/G]GTCTGTGCATGGCTGTCATCTCTGCCATCATGTGTTCAGTGCACAGGCATTGGGTGGCTGAAGAGGAAATGGTCAGCCTCCTCTGGGCAGGTCACCAGTT
23	1507468	SCAFFOLD131821_4243	0.48	0.04	CCTGCCTGAGGGAGAGAAAGTCCAATCTGTGGAGTCTGTGCATGGCTGTCATCTCTGCCATCATGTGTTCAGTGCACAGGCATTGGGTGGCTGAAGAGGA[A/C]ATGGTCAGCCTCCTCTGGGCAGGTCACCAGTTGACCTGGTTGCTGATGGTCTACTCCATGGTGGGTACTTTCTTCTAGGCATTAACCTGAACTGGAAAGA
23	3314163	SCAFFOLD15117_27903	0.14	0.13	AGGTAGCTCAAAGGTTTCCTGATTCTGCAGCCACTGCAGTGAGACACCACATTCCCATCCACTGTGCAGGGGTCATTGAGGAAAAGTCTGGGAGCCTGCC[A/G]TGAGGGGCACACCAGAAAAGGAAGAATTCCATATCCCTTTGCTTCGCAGAGGCACAATGGAGTAATTTAAAGCAACAACAAAACGGAATTAACCTTTTGC
23	3314578	SCAFFOLD15117_27488	0.17	0.23	TTGAGCACAACTGTTATCATCCTGAGTCTTACTGCAGCCATTTGTAAGTTTCAAGTGCACACAAGAACATCATTTATCTCTTTTGGCAGGCAAATACTTT[A/C]CCGCCCTGCAGTTAAGATTATAGATACTTCGTTTTAGAGCTGTTCATTTCTGTTCTTTGCATCAAAATTTCAAGGATACTAATAATAAGCTAACAAAATC
23	3314692	SCAFFOLD15117_27374	0.19	0.23	AAGATTATAGATACTTCGTTTTAGAGCTGTTCATTTCTGTTCTTTGCATCAAAATTTCAAGGATACTAATAATAAGCTAACAAAATCGTTGAAAGAATTT[A/G]GTATTTAGGTTCTATTCTTATTTTCTGGCTTGTTTGTTGCTAATTTATCATCCCCTTATCCAAATGATGGCATGTTATTAATTGCTTCTTTCTGTGTTGT
23	6883797	SCAFFOLD362148_210	0.27	0.38	ACGTCACTTCAGGAAGAAGCCTCCCCTACCTGCATCACGGCAGCCTTCAGACCTGCCGGGGTCCTCGCTCTTGCCCTGCATTCTTAGTCTCACGACGCCA[A/G]TGCAGACCCTCACGGTGTGAAGCGAAGGACTACGATAGGGCTAATGCCAGCAGCCGACCATGTTCACTGCACACAAGGCACTGTTTTAAGTCCTCTGCTT
23	6896529	SCAFFOLD96069_11607	0.31	0.41	AGATAAAATAATAAAATGTTTTTAAATGTCTTTTAAAAATTATCAAGGACTGCACCAATGTCACTTTCCTGATCTTAATATTGTACCACAGTCATGCGAC[A/G]ACGTAACCGCTGGGGTAAACGAGGTACACAAGACATTTCTGTGCACAAGCAACACTTATTTCAAAATTAAAAAATTTTCACAATGAACAAGTAACTCAGT
23	7339117	SCAFFOLD102055_10178	0.41	0.47	TGTAGGAAAAGGTAGCAAAGTCAACCGAAGAGAGAAGGCAATACCAACCTGGGAATCTCCCTTACCGGTGGCCTCTGAAACAGCTTGAATAAATGAAAGC[A/G]TCAGGTTGGTCCAATCAGAAGCTCTGTTGTTGAGTCGTTTTCTGTGCTGCTCGTTACCGTCCCCAGCGGGAGGATCTGGGTTTCCCAGGCTCTTCAGAGA
23	7458121	SCAFFOLD75350_9632	0.49	0.42	CACTTTGCTTCAGAATTCCCTGGGAGGCTTTCCAAATTGTCCCATGCCTGGGCCCATCTTAGACCCGGGACACCAGAACCTGGGATACAGAGGCACAAGC[A/C]CTTTGGGGAAAGCCATTCTGGTTCTTCTGACAGTTCAACCCCTGGCTGAACTTGGATCTGTCTCCTGAGATGCCCTGAATCGAAGGAAAACATTTTTACT

23	8187274	SCAFFOLD115214_5943	0.26	0.44	TCATTGGGGCTGGGACCAAAATTTCCAAAGGGGAAGACCAGCCATGGGACGTGAGCAGTTCAGACATGCTCGCTACACTCTTCAAGCTTGTAATCTTAGG[A/G]ACCGTCTTTTGGATTTTTTGTTTGTTTTTTGAATGTTTTAATATTTAAACTCAAAAGGCGCTTGTAAATAGTATTTAGTGAAGTCTCCCTCTCAGCCCTG
23	8498505	SCAFFOLD131392_3007	0.24	0.20	ACCTGATGTCATCCTCGTTGGCGAAGATGATGACCGCCCTGGCATTTGACGTCTCCAGGAGCCGCCGGATGATCTTGTCGAACTCCCCAGGCTTGGGCTC[A/C]CGAGGTATCTTGACGGACTGGGCAATGCACACGCCCCCTGCAGGAAGGGCAGCGGTCGGCCTGGGGCAGACAGGGTCTTCCCCTGAGACTCACACTCCTG
23	8630436	SCAFFOLD305882_9044	0.45	0.45	GTGACACCCAGCTAAGGAGGTACCTCAGCATCTGCGGATGCAGCCACGAGGCTCTGTGTTCTGTGGCCTGAATACCAGCAATGTGCTCAACCCAGGACAC[A/G]CAGTCCTCACAGAGACAGCGGCTGGGGTGACAGATGGTATGAAGTGGAAGAGATGACAGCCTAGGGAGGTAAGCATTTGGAGGTAGGGTAAGAGAGGCTG
23	12071453	SCAFFOLD85366_314	0.15	0.29	TCGTTTTCCTGGATAAGTTGTGGTACCATATCTCTTCTCTTGGGGAGGATGTCTAAAGGTGAAGGGTGAAAAATTAGTGGTGGTAGAAATAATACCTCTT[A/C]TCTTTACCCATTACATGAGTCATTAAAGATAAAATTGAGTTGGGCTGCTGTTAAAACCTTGGAATCCCTGACTCAGAAAGGCTGCCTAGGGCAGCCCCTG
23	12729291	BES4_Contig286_900	0.14	0.24	GCAGATCTACATTTCATTGCAACAAATGTCTCTATTAAACAAGTACTGAAATTTGCCCTTAACATGCCACAGAGTTAAGGGTTTTTCCTGAGATGTTCTG[A/C]AGACATGCAGGTGTCTGAGACTAAGGGCCTGGTGTGGGGCGTTTTTTAAATCCCCTCACACCCAAATTTCTACATGGAATATGAGACTCTCGTGGATCTG
23	12740444	BES2_Contig212_569	0.42	0.38	CAAAAGTCACAGTGACTCTGACTCAGTTGCAAAATTAGAGTATATTCTGTCCTTAAATCTCACCTTAAAATTTTCCTTTTTCTTCTAAGCTTGCAATTGA[C/G]TCTTTCTGTAAAGTGTCTTGTGATACTGTGAATTAGGTGATATAAAAATAAAGCACATTGCATTATATAAAGTTTTACTGCTTCTGTGCTATAACATCAA
23	12741013	BES2_Contig212_1138	0.14	0.23	TGTGTACATTTAATAGCTCCTGAATGATTTCAAATGTCACAGGAAGATAATGCTTGAATGTGGTGGCCAATTCAATAGAAAAACAATGCTAGGATGTTTC[A/C]CAGGGCTTCCTAGGAAGGCTTCCAGGCTTCATGGGGAATTTGGTCTTATATGAAACACACTGACAAAAATATACTTGTTCCATATAAAACCTAGAAGCTT
23	12851853	BES4_Contig320_1060	0.13	0.21	ATTCATTAGTTCTTGTCACCAGTCTTAAAGACGTTGACTGATAGTTGTGTTGAAGCCACTAAGACTTGGTACTTCAGTTAAGATGACTGGGTGTCATTAA[A/G]AAAACAAAAACAAAAAGCCAAGAGAAACAGATGTGTGTGCTTCTGGGGCCTGCGGTTCTCTTCACCTGTACTTCTCTTGCCAAGAGCAATAGATGGGCAG
23	13608773	SCAFFOLD45473_5592	0.08	0.20	GTGAAGGAGGCCATGGGACAGCAGGTGGTGACAGCACCTGCCTAGGTCGCAAGGAAAGCCACACCCTGTGGGCTCAGACCACCCTGAGCAAGGAACCTGG[A/G]CAAAGTTACTTCACTTCCCTGAACCTCAGCGTCCCTCACATGTAAGATGGGCCAATAATCCCCATGGTGTCACTGCAACCATCAAATGAGTAAATAATGA
23	13608912	SCAFFOLD45473_5731	0.10	0.21	GTGGCTTTCCTTGCGACCTAGGCAGGTGCTGTCACCACCTGCTGTCCCATGGCCTCCTTCACCTTCCCTGCAGCCTCCTGGTTTTTCCTTCTGGGCTGAA[A/C]ATCCTCACCCTGCTGGCTCAGGTCCCCTATGCACTGTGGGGCTGACCGAGAATGACGGGAGGGCCTGGCGGCCCTGTGGGGATACCTCTGTTGTCTCTGT
23	14911353	BES1_Contig463_878	0.38	0.33	ACCTCTCCCACCTTTCTTCCCCTCATTGACTCCCCTACTTCTGACACTGCTCCCATGAATGACAGTCACTTCCTGAAAGGGCAGGCCCTGGGGCTGACAG[A/G]TGCAGGAGTCAGTCTTCCCCAGTCACATGGTCCCACTTGTGGACTGTCTGTGTCTAGTATGACCCAGAGCTGCCTCCAGGGCTCAGTCATGGGTACTTAG

23	15541042	SCAFFOLD251761_4620	0.28	0.30	TTACAGATAAGGCAGTGACCCACGAGTGAAACCACCGGCCCAAAGTCTCAAAGTGAACAAAGTCCCCCTTGGTGATGAGGGCTGTAATGGCGGAACATGA[A/C]CAGGTGGGGGCGCTCAAGAGAAAGCGGTCACGCCGCCCCATAAGGCGAAGCAAACCGTCCAGATGGAGATGACTGATGCTTAGGCTGAGTGAGCCTGGAA
23	16667462	SCAFFOLD290983_14884	0.38	0.40	GCAAGGTGGGGCTGGTTTAGAGAAGGTGAGAATGATTCAATTAGTCAACACTTAATCACACAGCCCTTAATATAACAGACGCCAAGAACTGCAATGGAGG[A/G]AGAGAGAGAACTTACCAGACAACTACAAAACCACTGTAAGGACGATGATGGTGATGCAGGGGCACCTCCTTCGGTTACTGAACCAAATACCAGTGCACTT
23	17213379	SCAFFOLD290165_19324	0.09	0.14	GCTTGTGTCTAGCTGGTTCCCCAACCCTAGGACAGATGCAGTAGGAACGGGGATCCTATTCCCAAAGCTGGAACAGATCCTGGATTCTCATCTCATTTCA[C/G]CCCGGTCCCTTCCTGCCAGGCTTCCCCTTTGATCCTCCTCAAGCCTCCTCTAGGAAACAGGCCCTCGAGGACTCCTGGGAGCTCTCTCCAATTTTGCGTA
23	17300967	SCAFFOLD130115_22826	0.06	0.12	CTGAGTGGGACAGGCGAGGGACCCACAGCGGGTGGTAGCGTGGGCTTCCCCGCCCAGCAGCACAGCTGCACTGCGATCGAAGCTTTGTGGAGACAGCTGG[A/C]GCTCATCATTCTGCTTTGTGCTACCCAATCATGGCGGGAGGGGGAGTGTGTGCCATTCCTGCTCAGGACACAAGTGGCCTGAGGCTGGGGAGTTCTTTCT
23	17651249	SCAFFOLD220683_1125	0.09	0.07	CTCTCGTCTCCCTTCCTGCCCCTGGCCTCCTCCCCTCTGCTTACACCGAGCCATCTCCTCTGCGCGCTTGTCCTTACCACTGGGTTCCCTCCGTTTGCCC[A/G]GTCTCCTTGCCGTAGGTGGGGCTGTGCACAGGTGCCTGTCCACCTACCAGCCGGCTGCTGGGCTCTGCCCCCATTCCTCCCCTCAGTCAGTATGTGCCTC
23	17653167	SCAFFOLD172924_2966	0.07	0.11	GGAGAGCCTGCCCGGGCCCCACCCGTGTTCTGCTGGCTGCTTGCCTGCCACGCGCCCAGGATCGCTCAGGGCTTTGCTCAGGTATCTCGGGAACAGCTGC[A/G]CCGGAAACTGGGGGCCAGGGCTGGGAGGACTGAACCCAGGCTGCCTCAGTAGGATCAGCTCTCTGTGCGGCTGTTCACTGACAGTCAAGGGGGAGAGGAG
23	18237013	SCAFFOLD215757_14509	0.29	0.26	GGGTCACAGATGCCAACACAAACACCATTCCTATGGAGGCAGAAGAGCCCGTGGGAATTATTTTTAACCTGTTGATCAGAGGCCACAGCTCTGGCCCTGG[A/G]AGGCCTGGAGCTGCCGGTTCTCAGGGGGAAGTGAATCGTCAGGCTTCCCCTCAATGACCCTCAAGGGCACTGGGTGGTGGAAACCCCAGCCCTCACCCCA
23	18658929	SCAFFOLD115359_9329	0.13	0.13	TTCAATCCCTGGTCCGGGAAGATCCCACATGCCTTGGGGCAACTAAGCCCATGTGCCAAAACTACTGAAGCTTTGGGGCTAGAACCCGACAAGAGAAGCC[A/T]CCGCAATGAGAAGCCCACGCATGTAACTAGAGAGTAGCCCCTCCTCGCCACAAGAGAAAGCCCGAGTGCAGCAACAAAGACCCAACTCAACCAAAGATAA
23	19956369	SCAFFOLD105090_4815	0.15	0.20	CTGCAACCTCGCCCATTAGATGAAGGGATAACTGGACAATGTCTAGTCCTGAATTCTTTGTTTCTCTCCATGCTCACTTGTTCAGCAGTATTAATTGGGC[A/G]CCAATTATAAATTATAATTGACCCTTGCTTGTGAAGAGCTGTAATGCAAATTCTCCACTACGTGCCTAGCAGAAAATGTTGATAGTGGCCATGAAAAAGA
23	19980120	SCAFFOLD105090_27305	0.44	0.41	CTGCAACCCTCCCACAGAGGAGGGGTCAGCAGATCTTGGTGAAATGAATTGTTGACCCAGCTCATTCCTAACAGGCAGCTTTTTCCAGAGAATGACAGAG[A/C]ATATGGATTATCTGCATGTGAAGATTGTATGGCGTGTTAACTGCAATGTCTGCTAGTTAAAATGAGAATGGCCATTCAGGCAGGAAGACTCTTAGTTGCA
23	19980296	SCAFFOLD105090_27481	0.46	0.41	GAGTTGAGAAATACGGAAATAGAGCAGATTTACTGACACATGGACGTGGACATCCACATGTATACTACACCCACACGTGTGACTATCAGGGGTAACACAT[A/G]CTACAGCAGATTGACCCAGAAATTTGACTTGGTGGCAGGAGCAGGACTGAATTCTGTGTTCTTTGTGACATTTTCTGCAACTAAGAGTCTTCCTGCCTGA

23	20800011	BES10_Contig539_997	0.28	0.36	ACTGAAAGTGAAGATGAGTTCAAATATCAAAACGCCACCTAAGCCTGGTCCTGTTTCAACTATCTTTTCTCCTCTTTTCAACTGTGCCTTTTATATCCAC[A/G]TTTCCAATGCAGGGGAGAGCATCACTTGCCAGGATCCTATCCTAGGTGTTGGGGAGCCTGGGAAAGTCATCCAGAAACTTTGCCGGTTCTCGAACATTAC
23	22188230	SCAFFOLD1747_667	0.41	0.35	AATAATAAAAATAAGAGGAATATTAATGAAACGCACAACACAGTGCACACATACAAACACACACACAAATTCCTGGTTAAATCTAGCAAAGAGAAAAGCC[A/G]CCTCTTAGTAATTTGCTAATCATTCCTTTTCTCTTAAGGAGTTTCAATAAACAGATACAGATTTATTTAACTTCTCTCCATTCTTCCTCCACATTCAAAG
23	22188607	SCAFFOLD1747_290	0.43	0.40	TCATTCTAAAGAAATGTCTGAAATATCTGCAAGAGTTAAGAGCAAGATAAGCACTAATTTACCATCTCCTTCATCCCCATAAGGAGGTACTTAAAAGAGG[A/G]CAAAATGCATCACTAGGTATTGTCAGGGTCAAACTAAGACTTCAGTATTTCTTTGAATAAAAGTACTATGCTCCACTGCCTAAGATGAAGAGTGTTTTTG
23	22575044	BES7_Contig424_1473	0.04	0.12	CATCAGCCCTTCAAGGTAACTGCCGAGACTGACTCCTTTCACAGTAATTACGGCTTCTTGAGTCAAAATAGTTTTTTCTGGGTCTTGAGGATGTGAAACC[A/G]TCTACTGAAACCATATTTGTAAATGAAATATTTGTAGATTTAAGTTCCATTGTTTTCTCTACGGGATCCACTACAGAATGTTCTTGCTCATATGTTTTGG
23	22575178	BES7_Contig424_1339	0.04	0.16	GCTGCTGCCGCCATTGGTGTCTGTGTGCTTCCTCTTGCTGAAGCTGTCAGTTCCTCAATTTCAGCATTTAATTTATGTATTACCCACTCCATTGCTTCTC[C/G]GCCTTTATTAGCATTTGATGATATCGTACTTGCCATCAGCCCTTCAAGGTAACTGCCGAGACTGACTCCTTTCACAGTAATTACGGCTTCTTGAGTCAAA
23	23137143	SCAFFOLD115511_4737	0.23	0.21	TTGTTCCCTCCACCACACCCACCGCCAGCCCCATCACATCCCCGAGTATCTCTCTCTGTAGCATGCTGGGCTGGGTGTCAGGTGAACACATATGAATGTG[A/G]CTGGTATTTACTCACAATGAATCATCCTCATGTTGGATGGACCAATCACAAAAGGTAGGCATAATAAGACCTCACTAGACTTTGTAAATACAGTTTAAAA
23	23595534	BES2_Contig395_725	0.16	0.13	TGGAGTAGTAGCTGCTTCTGCAACAGTAGGGACAGGGCAGTGGAGAGTGAAGGATGAAACTGGAAATGCCTGTCCTGGAAATGAGAACTTAGGGTGACTA[A/T]CTTCATCCCTTCCATCTGCTTTATGAATGATAGGCATTGCTAATCAATCTTGGAAGTCTTCATAAATGAATTAGGAGGCAAAGTCAGAACTGTTTTTCAT
23	24171139	SCAFFOLD141184_3477	0.06	0.08	ACTTGAATTATCCAGGATAATCTCCCCATTTTAAAATATTTTACTTAATCACACCTGCAAAGTTGCTGTTGCCAAACCTTCATGAGTTCTAGGAATTGGG[A/G]TATGAACATATTTGGAGGCATTTTTTCAGAATAGCATAATTGCATTGTTGAATTGATAGTGTTGAGAAAATGGACTAGCCATTTGAAAAGAAATTAGATA
23	24975294	SCAFFOLD81570_11372	0.05	0.07	TTTTGTATCTCATATTCCTGATACAGCAATTTAAGTATAATGTTGCCTTTCCTAACATGATGTAGGTAAAGAAATTGTTGGTCAGTGTCCACCATACAAT[A/T]ATTCTACATCTGTTATTGGATCATTTAAGCAGATGTGATTGACTTAGATTATCATGGAGGCAGATGGCAGAGCAGAAGGAAGTTTAGGAACGGATTTGCT
23	25718358	SCAFFOLD146149_8302	0.25	0.37	GACCTTTATGTCTTCTATGCTGGTGGGTACAGCCAGCTCTAGGCTCACAACAGGAGTTCCACTCCAGCACTTCACCACCCAAATTGTCTTGATCATCACT[A/G]TCAAATTTCCAAGAAGATTGTGTGGATTACGACAATGTATAAGGGTGTTAGTCGTTCAGTCGTGTGATTCTCACAGATGTGGGTTCAATCCAGCAATCTT
23	27076772	BES2_Contig347_1219	0.27	0.18	GCTGGGAAAGGTGATCATTCAGGGGAAGAAACAGGATGCAGGGCTGGCTCAAGGACTGCGCAAGATGTTTGGCTGATTAAAAGTTAAACCTTAAAAGAGA[C/G]AGGAACCTGTGAAGTGTGGTCTATGCTGTGAGATTTGGTGGGGACCAAGGGAGAGTTGCACCATATCCCACTCCAAGCTTCTGCCTTCCTAAGGGAGGTA

23	27286587	G73136-248-R	0.33	0.33	TTCAAACATTTATTGAACACTTACTATATACTTGGCATTGTTCTAGGTTCTGTGAATACAGTTATGAACAAAACAGAGCAAAAATCCTTGCCTTCATGGG[A/G]TAGGGAATGGGGAGACAGACAATATACATAATAAATAACTAAATGTATTTTTTGTGTTAAGAGGTAATAAATACAGTGGAAAAAAAMCAAAATAGGGCAA
23	27913734	BES10_Contig768_1175	0.08	0.21	TTTCTAGATTGTTTAGACATTTGTTCTTGCCTTAGGGCAGTTGTGAAATCTGTTGAATTATCTTAATTATCTGGACATGTGCCTTACTCCCTGACTTGGA[A/G]GCAGTTAGGCAGCCTCAGAAGGCCTGGGTTTACATGAGTTCTGCTGTTGGCCCCTGGTAACGTCACTCAGACCTGCCACTCCACCTCTCTAGGTCTTAGT
23	28338172	SCAFFOLD185040_54480	0.45	0.41	AGTGACGCCCACCCCCTCACCGCCCCCTCCCACTGGGGGTCCAGAGTTGTGTTCCATGACTGGTGTGCGGCGTGCACTCTTCTGATCCAGCCTCCAGAGA[A/C]GTCTCCAGGACCATCATTGCTCCATTCAACTGCCTCCTGGCTCCTCTTCCCCCCTCGCCAGCTGACTGCGGAACAGGAAACCTCTTTGGTGCTGTTTCTT
23	30238291	SCAFFOLD155216_10397	0.17	0.34	AATTTTGAGATGAAAAACCAGCAGGCCCCGCTGGGATTCGAACCCAGGATCTCCTGTTTACGAGACAGGCGCTTTAGCCAACTAAGCCACAGAGCCAGCT[A/G]TTGGAAAGCTCAAACTTCCTTTCTGATCAAATGCTACTCAATTAGGTGAAAAGCTGTGTTAATTAGAGCTACTATTCACTCCGTGGATAGAAGAGACGGG
23	30241062	SCAFFOLD3944_744	0.14	0.27	GGAATGAGAAATAAAATGTTTGTTTATGACCTACTAAGACAGAAGCTCTTGTGACAAAGGTCAAAGAGTTTTTTGGGAAAAAAAGACGGGAGTGGAAACC[A/C]ACTTAGTCTTTGCCTCAGGCTGATTTGCTCTCTGACCCCTCCCACTTCTCACCTCCTCCAAAAGAGTTACAGGATCAGTCCGATGGGACCTTGGCAATCG
23	30241510	SCAFFOLD3944_296	0.12	0.16	ATGGTCCTGCTGGGGTAAAAGCCAATTTTACAGTTGCTTGTTTTAAACCGGGCCTTGTCAGATTCAAGAGAATCTGAAGGCTTTAATGAGATTGATTTAC[A/G]TAAAACATCTTTCCGACATTTAAGTCCTCTAAAATACTTGCACAACAACGTAAAATACACGTAAATGTTTTTTATGCGGAAAGTTGACCGCATATATTCA
23	30758253	SCAFFOLD195518_6712	0.07	0.10	GAATGGAACCTTCAAGACGTGGGCGGCCCTAGTGGTCCCTTCTGGAGAGGAGAAGAGATACATGTGCCATGTGCAGCACAAGGGACTTCAGGAGACCCTC[A/C]CGCTGAGATGGGGTAGGGAGGAGGCTGGGGTGGAGCTTCTTCTCAGATAAAGCTGGAGCCTTTCTGGATCCCTTCAGCAAGAACAGGATTCAAACCTGAA
23	30758291	SCAFFOLD195518_6750	0.04	0.07	AAGGCTCCAGCTTTATCTGAGAAGAAGCTCCACCCCAGCCTCCTCCCTACCCCATCTCAGCGTGAGGGTCTCCTGAAGTCCCTTGTGCTGCACATGGCAC[A/G]TGTATCTCTTCTCCTCTCCAGAAGGGACCACTAGGGCCGCCCACGTCTTGAAGGTTCCATTCCCTGTAGGCCTGGTCTCTACAAGCTCCATGTCCTGGGT
23	31601937	BES7_Contig253_1064	0.38	0.34	AAGGTTTGTTTACTGTGAGAGCTTCAGTGATCATCAGGGACAGCTCTATGAAGAATGTGTCCTGCTGCATCCGGAACCTCCTTCTTGGCCAGGAGAAAGA[A/T]GTAGAGGTTTCCATACCAGGTCAGTAAAACAGGTGCTGGGTTCCTTTTTGACACAGTTTCAGGGCAACATCACATGGGACATCTGCCCAGCTACAACCTC
23	31602377	BES7_Contig253_624	0.38	0.44	GAGAAAGAGAAGCCCAGAGGAATTGGGAATAAGAGGGACCCATCTCCATGTTTTACTACACAAGGGAACTGAGGTATCCATAAAAGAAGACATCTTATCA[C/G]AGATGAAACAACTATTGGCTTAAAGTCATATTCCAAATTGTGTATATATGTGAGAATATGTTTTCTTTTCATGAAAAGGGCCACCATTTTCAGCAGATTC
23	33027223	SCAFFOLD96303_4158	0.37	0.26	AGCAGCTACCTTCTCCTAGACTGAGGCTCTTTATGCGCTCCCCACCATCCTGGGGGAGGGGGACAGGAGGTTACGGTCTCGCGGTAACAGAGGTGCAGAT[A/G]CTTCTTTTTCCTAGACCATCTCCCACGCCACAGAAACTTGCACCGGTGCTCCTCATCAGGTGCGTCGACACTCCTTATCCCTTCTAGACTCTCATCAGCA

23	33670009	SCAFFOLD185066_42127	0.45	0.47	CCATCCAGATCGGTAGATGGCAGGTCTAGTAAGCAAAGAGAGTCACTAGCAAGGCTCATCTGATGGATGCACCCCACCCGCCCATACACACCAGAATCTT[A/G]AACGTTTATATAGAGCCCTTAATTGAGTTCAGTCACATAGTCACTCCAGATGTTGATTCACTCATCAATTGCTCTCTTGCGGCTATGTCCTTGAAAACAG
23	33670262	SCAFFOLD185066_42380	0.44	0.47	GACTTCATCTAGGGTAACTCCTGAGGTTGCAAACGAACACTTGGCCGCCAACCCCCTCATCTTAGCTGTGACTGGATCAGAGATTTGCGCGCACACACAC[A/G]ATCAGAAAAAAATGTTGAGGTTTCATTCAAGTGTTAATAGCTTTCTTTAATTACTGGGGATAATGCTTCCTTTTATTATATTTATCAAAGCTTCTTGTTT
23	33670373	SCAFFOLD185066_42491	0.45	0.47	AATGTTGAGGTTTCATTCAAGTGTTAATAGCTTTCTTTAATTACTGGGGATAATGCTTCCTTTTATTATATTTATCAAAGCTTCTTGTTTTCTTTTGTGT[A/G]TTCAGTAGATATTGATTACACACGTACTACATGCAGGCTTTACGATGCATGCTGGAGATGCATCTGTGAACCAGACCAGACCTGTTCCTGAGTTCAGTGT
23	33670488	SCAFFOLD185066_42606	0.44	0.47	GTCCTGATCCCAAGACGTGAGTGAGGCACTCGGCACCACTGGCCAACATCCGCCACAGCCCTTGTCACATACTCCTTTCTAATGCGCTCTGATTGCTTGT[C/G]GGGGGGTCATGGCCACACTGAACTCAGGAACAGGTCTGGTCTGGTTCACAGATGCATCTCCAGCATGCATCGTAAAGCCTGCATGTAGTACGTGTGTAAT
23	35900238	SCAFFOLD35003_41225	0.30	0.29	TCAGAGGACTGATAGGAGATTTCATGAGAAAAGAAGCCCGTTTTTCTTTCTTTCTCCAAGCTGAAGCCAAAACAGAGATGGCAATGTGAAGTTCAGCAAA[A/T]GTGGCCACATGAAATTCTCCCTGGAGTATAGTCCAGAGGAAAGTCAAGTTAGAAAATGATTCTTTGGTTATGTTTGAGAGAGGTTTTAATCTCAGCAATG
23	37198979	SCAFFOLD31957_2263	0.48	0.48	CCTGTATTACTTTGCAAAGGACCATGACTTGTGTTGTGTTCATACTCTCTCTCTTGCCATCCCCTTTGCTTGCTTGATGAAGCCAGCCACCGTGTGGCAC[A/G]CTGCCATGTGAGGAAGCAAGGAATAGAGTAAGGCCTCTGGCCACCAGCTCATGGGGAACCAAAACTTCTCAGTCCAACAGCCTAGGAGGAAATGCCTCCT
23	37558866	SCAFFOLD248940_3447	0.33	0.21	AATTCCATATGCGACTTGGAACCTGCAATGAAAATATAGCGTGGAAAAGCTATATTTCTAAAGTGTGCAAGGGATATTGCTTAGTTTCAGCCGCTCTTCC[A/G]TCACAGTTGAGTACTGTGTCTCATAACACAGGCTTGATTTTTTGACACCAGACATTTGGTGGCAGTGGCTTTCTAAGATTTGCAGAAGAGAATTAATTTA
23	37696587	SCAFFOLD153095_8152	0.31	0.32	CCAATGAAGAAGCAATTAGCAGGAAATAACACTAGAATCAATTATTCACTCAAGTTGTGATTAAGAGCCATGTGTATAACCCTTAAAATTTAATAAAGCC[A/G]ACACTAAGCAGGAATCCATCTTTTTCATCACCAGGAATATAGATAATCCAATCATAGGAACTACCTTCGATTAGTATCAACACCGTTTATGTACTCTGAA
23	39450899	SCAFFOLD75154_18798	0.01	0.04	AACGCCAGATTTGGTTTCCAATGAGCAGAAAGGAAAAATATCTTACTAACTGAAATAAAGTTGGCAAGAAAATTTTGAAGACTCCTGTAATATCCCTCGT[A/G]TCTCTGTCTAAATCATTTTTAATAGGAAATTTTTTATCCCCAGCGTATGACAAGGGCTCCCAAGAATGGTGATTTGTCTCTCAGACTAAGAGTGTGAATC
23	40253865	SCAFFOLD102173_483	0.47	0.02	TACCAATATTTTCCTGCCATATGATTCATGGTTTAACATTTTCAAGTTGGAAGAATTCCATAGAAATACTTAAAGTTTAATCTTTTAATGCATTTCTGCA[A/G]GTCTCTAACATCCTCAAGTACCACAAGATGTCTATTGCAGTTATTTCTCTATGAGCCATTATATTTTTTAGCAAGTTGGTTCTATGGAAAAACATTACAT
23	40284986	SCAFFOLD255402_24210	0.42	0.17	GTTCCCCTCATATCATTTTCACAGTAGCTGAGAGGTGAAGGTACTGCATGGCTCACTTTTACACTTAGGTTTAATTGCTAGTTGAAAATAAAACCTTCGG[A/G]AATCTAAGATGTACATTTTTACCTTTTAGGCAATTTCCATATATATAGTAGTAGTATGCTCTAAAAAGGTATGATTTCTGTTTTCTTATTTTTACCACCT

23	40983286	BES3_Contig418_1154	0.39	0.43	AATTTCCCCGTGTAGGCTAAATTTCCTTAAGCTTTTCTGGACAAATTCCCTGCATTCAACATCATAAATTCCAATGTTTAGTCAGAAAAGAGGCTCTGGA[A/G]CATTAAGATAATTACACAACTTGAACTTCTAAGTATATATTTTTTAAAAAATTAATAATATACTGTACTTCCTTGCTTAGAAGGAAATTTAAATTACAAA
23	41404196	BES6_Contig252_799	0.19	0.22	TATGGTATGAGACACTTGATGATATTTAGCAGAGACAAGGAGCCGACACCCTATGGCGTTGATCTGGATTTTACTTTGTATAGCGGAGCCACTTCTCAGA[A/T]CTTCGCAGCTGCTGCTGTGCTTTTCCCACTGATTCTCAACTATTCTTCTAGAAAGAAGGCACCAAAAGCTCCCAAGAGAACACAGCTCTCCCAACACACC
23	42589585	SCAFFOLD86180_8791	0.39	0.47	GCTGAGATTTCTCACCTGGGGACCGGACTGTGTGGGAAGGATGAAGAAGGCTAAAGGGAGAGGAGGGGGTCTCAGTTTACTCTGCCGCAAGGCACTGCTC[A/G]AGAAAGGATCTTACTGTCTATACCGTAATGTGAACTCGCAAGACAGCAAGCTATGAACTTTTTAAAGTCTTTCTCGAATTTGTTACAATATTGTTTCTGT
23	44574226	SCAFFOLD130166_763	0.25	0.46	GCAGCAGACACTATACTAAACCCAAGATAACAGTCAGTTATAATGAAATCTCAAGCACAACAGAATTCAAGAGGAAGCAACTGACCCACTAGGTGTAATG[A/G]CCTCAAAGGTATGATCATAAACAGGAGGTAATCAAAGTGTTCCTTAACATGGAAAGATAATGAATGTCTCTGTACTTCAGTTCCCTTATGTGTAACATGG
23	44577296	SCAFFOLD130166_3832	0.15	0.12	CACCCTTGAAGGAACCATCTTCATCCAATTTTTGTTTTATTGGTGATAAGTAACCTGATTTTCTTATTCATGCCATAAATGATGATATACTGAACAGACC[A/G]ACATGCTCAGTCAAGAAAAATCTGAAATGTGGAAATGTCCAAAGTGCCTTCTTTAGGAAGGCAAAGAAGATATGGTTTAATTATGAAAGCCCATTTCACA
23	45550461	SCAFFOLD21426_3200	0.25	0.22	GTCACTAAAAATATGTCGTGTAAATTTATGACTCACCCTAGACAAATTTTTGAAAACAGAACTCATCGACATTCAGAGGCCAAAAAAACTGAAGTGCTTT[A/G]TTCTCACAGTATTAAAATTAGTGACTACCTTGGGAATAATAGTAAGACAAATAAAACTTGATTTCCTTTCTAAGAACAACACAAAGGGATTAGAGTTAAA
23	48193360	SCAFFOLD140402_6904	0.21	0.25	GAGGCAGCAAACCATCTCACTTGGATTCATAGTGAGTGGACTCTGCAGGGAAAGGGACCCGAAGTTGTCAACGCAACACCAAGCACAGCCAGAGACTCAC[A/G]AGCCTCCAGGAACCCTGGCCAAGACCCTGTGTCACCTTTCGAAGTCTGACTTGACCCAGCAGAGCCAAATCTTCCCCCTTCTATGTCCCCACACCCTGTA
23	48491515	SCAFFOLD195870_2156	0.47	0.41	GTAGCCAAAGATTTCATGCTCTGTGTTGGAAAGTTCGAGTAAGCTTGTTACTCTGAGGCTACTGACCAATTACAATCAGTCCGTGAGGTGGTGAGAGCGC[A/G]TGAGTGAGGTCAAGAGGTAATAATTAAAGATGATGTAATGAGATGATCCAACCTCCCCCGGCTGCCAGAAGGCCCGGCGGTGACCACGCTCCTGCTCTTC
23	50100754	SCAFFOLD141061_5804	0.29	0.23	CTTCAGAGATGAGCGACAGGGAGGTTCGAGGGCACACAAGTGGCTATTGGGCAAGACGCTGGATCGCTGAGGAACTGCCCTTGGGTTCCCAAGAGGCTGC[A/G]GTGTAGCTGGGAGTGTCTGCAGGGCTCAGACGACTAGACCCGGCCGAGAGGGCGGCGAACTCGGGATTCCACTCGTATCTGGAGTCACATACCGTGTTCC
23	50236310	SCAFFOLD180254_14008	0.19	0.18	CCGGGAAATTAAGACAGTCAGTTGGGAAGAGCGTGGCACGTGGGGAACAGGGGCATCTGAGCTGATTTCCGGGAAATTAAGACAGTCAGTTGGGAAGAGC[A/G]TGGCACGTGCGGAAACGCGGCAGGCGCTCTTGTTGGCACCGGAGCTGCCCTGTCCAGCGCAGCAGCCCTGGCCGCGTGTGGCTGTCATGCATGTGGAATG
23	50641815	SCAFFOLD155467_15287	0.43	0.41	CAGGTTTTTTTGTTTTTTGTTTTTGCTTCCCCCTGCCCCGCCTTCTTTGGCGGTTAGAATATTTGGTTTCAGTGTCTTCAGACTTCCATAGTTTCTTAAC[A/C]AGAAGTCAGTGTATGTTCTTATTTTTATTGTCTTTGTTTCCCTGTATATAATGAATCATTTTTCTTTGGCTGATTTCAAGCTTTTATCTTTGGCTTTTAG

23	50641904	SCAFFOLD155467_15376	0.41	0.41	CGTGATAAATTGTTTTCTGTTAGAAAACGTCCTCGTTTCACCCACAACTGTAAAGAATAGTTTTGCTGGATACCAGAATTCTTGGTTGACAGGTTTTTTT[C/G]TTTTTTGTTTTTGCTTCCCCCTGCCCCGCCTTCTTTGGCGGTTAGAATATTTGGTTTCAGTGTCTTCAGACTTCCATAGTTTCTTAACAAGAAGTCAGTG
23	51841473	BES9_Contig534_1084	0.19	0.16	CTCCTAGAGATCCTTAATAATCTGAAATGGTTGTGAGAAAAGCCACTCGGAATAATTAGAACACCTGAATTATGAATTATGAAACATGACTGAAGGAGCC[A/G]TGAGAACAGACTGTGCCCTGTCGACAGCTCAAACAAGCCTAGTTTAATGAATAGAGAAGACGCGGCCGTTCACGACCGCCCAACACTCTCACTCACGCGC
23	51845887	BES3_Contig338_843	0.34	0.43	ACGATCCCGGAGAGGCGGCGGGCAGGCCCTGCCGTCACTCGCTGTGGAGGCGGCTGGTCGGCCTCGTACACCCCCCTCCCCTCCATCTTTGCTCCTCCGG[A/G]GTTTGGAGTGCAGCTTTTCCACCTACCAAGCCCTGGGATGACTGTGCCCCTGGTACTCATTTTTTCCACATCCAGTTTTTATTGAAATTACTTCTAAATT
23	51966382	SCAFFOLD225086_14961	0.49	0.47	GAGTGCCAGGACAATGACTCTAGTGTGTGACAGCCGGAGCCAGACAGGCTTCCCAGGAGGCCCCCGTGTCAGATGGAGGGCCTGAGGGACAGCAATGAAG[A/T]GTCAAGGCTGCAGGGGAAGGGAGGTGGGGGAGGGCCACGTTAGGAGTGATGGATAACAGTTAGAGGGGTCCAGATGTTTTTATTACTTTAAATTTTGCTT
23	52549627	SCAFFOLD181521_3012	0.27	0.14	CTCCAGGCAGAGGCAGAAGCTTCGGCCAAAGGCCAGGAAGCCTTGGGCTGCAACAGTACACGGAGCGCAGCATTCACCAGCGAGACGCAAGGGCAGACCC[A/G]CCCTAACAGGTGACTCTGCGCAGAGCGGGCCTGTGCGCGGCTGGTGGGGAGTGAGGATCCCGCTGGTCGGCAGGAGCGCATCCTGCTCGCTGGGATGGCG
24	116843	SCAFFOLD215715_8569	0.37	0.08	CTGGGTGACGGCTAATTAAAGTCCCTGTGTGAGCTTCTCAACCCGTGGGGTACGCGTCACTGAAAGGTAGAAGGATGTTCTCGGGCCACTCCCCCTCCCC[A/G]CTGTTCCCCTCCGCGCTCTCATTTCTGTCTGGAATCCCTTTCATTTGTAGAATTCTTTTACGCAGAAGCCAGCAGTCTGCTTGCACCACTTCCTGTGCTG
24	1271471	SCAFFOLD150018_4530	0.22	0.22	GTGCTGGTTGGTCATTTACCAGCAGAGGCTGGTCGGTTGTTATGTCCTGCCCACACAAACGCAGATGCAGCCGGAAATACCGCAGAGCCACAGCCCCCAC[A/G]AGGCGAGGCCCGGCGGGAAGGATTCCTTTATGAGTCTTCCTGGACAACACACAGCAAGACAGCCTTCCACCACCACCAGGGGTGAGGGCAGCTGGTGGGT
24	1450431	SCAFFOLD222971_1856	0.47	0.49	GCCCTTGGTGACTCCTGGTGATTGAGCTCCTTACGCTTCTATTCGTTTCATGAAAAGGTAGCCCAGGTGTGCCTGCCCTGAGGCCCAGTACCTTCCTCCC[A/G]ACCCCGTGGCACCAGAGTCTCAGCCCCTCGCCGTGACACCTGGCAGCCTCACCCTTGTACCTGCTGGGCTGGCACCCACGTGGTCACCTCCTCGCACCAC
24	2538214	SCAFFOLD60223_13396	0.23	0.22	GAGCACAAGGGGCGGGAGGAGGATCACGGTCTTTCCTGCAACTCTCCAGTCCCTCCAGCACTGTCGCCTTCTCTATGCTCGGCCCCCAGGCAGGAGGAGC[A/G]AGACGAATCCTGTTCCCTGTGGAATGTCACATGGCCTGGCGAGCTGGGCTCTCTACTGGGGGTGTCTGGGCACCAGGCTCACTGGGCTGACTGAGTACAG
24	5176344	SCAFFOLD41706_5862	0.50	0.00	GCTGACGAGGGCAGCCGCAACAGCCGCTTCCAGATGCTCTTCACGTCCTTCCAAGAACGTAAGTGTCTCCGCTCTTCGCTGCTCGGCCTTCCAGTGGGTC[A/G]GAGGGTGCAGATCTCATGCACTGTGAATGGTGTTTCCTTCATGCAGCTTGAGCCTTAATAAGTTGTTCAAACCGCTTAGATTCCCTTTCCTTCTCTTTGA
24	5966664	SCAFFOLD236442_391	0.16	0.16	AGTTAAATTAAATCATATGAAATTGAGAGTTTTAAATAGAAATGGATCACTATCGTGAGGAAAAATTGAGAGCGAGGCTCTGATTTGTTATTCAACAGTA[A/G]CTCATCTTCCAAAATCTGTACTAATGTAAAAGTATAAATATGATGAAATAGTCTTTTAATTTTGGTGTTGTTATGTTGTCAACACAAGGCTGTTTTAACC

24	6349478	BES10_Contig566_1390	0.46	0.49	GTCCTTTGAAGATTTGAATGCAAGCAATGACATCTCCACTCTAGACTTTGAAAGTCTTAGATGGCATCTTCTTCCAATAAAAGACTGTTTCCTCTACATT[A/G]AAAGCCTCTTCTTTACTGTAGCCACCTTCATTAATGATCTGAGCTGGATCTTCTGGATAGCTTGCTGCAGCTTCTACATCAGCTCTTGCTGCTTCACCTT
24	6349942	BES10_Contig566_926	0.46	0.49	ATGAAAGCTTTTCATGGCATTTAGAATGATCAATCCTTTCCAGAAGGTTTTTAATTTGCTTTGATCAGATCCATCAGTAGAATAACCATTTATGACCGCA[A/G]TAACCTTACAAAATATAATTCTCAAAAGTAAAAATTACTCCTTTGATCCATGGGCTGCAGAATAGATGCTGTCAGCAAGCTGGAAAATATTAATCTCACT
24	7029519	SCAFFOLD221584_792	0.08	0.08	GCCACCTGCGAAGCCCCCAAGGGCTTAATATCCTTCCAGAATTTCTTCCACAAAGGATAGCTCCATCCTTGACACTCTGTCCTAAAGCATTTTGAACTTT[A/T]TTGAAACAAGATGGATGGTATCCATTTCCCACCCAGGTCATAAATAGTATATTATGATGAATGTTCAGGGAAATAATGGAGGGGGCTGTATGTAATTTTC
24	9443510	SCAFFOLD171254_10574	0.47	0.07	TTCATAAAGCCAACTCCATTAATGTATCTCTTTCCTCTGACCTTATAAGTAGCTTTTTTTGGGTAAATGTAATGTTGATAGTTTGATTACATGTTGTGCT[A/G]TTGATTTAAGGAGGTGCTTCTATGATGACACTGACTCTAGAAATGCAAGGCTTTGCAGATCCTATTTGCAGAGCATAGAATTAGGATGGTACTGTCCAGT
24	9870624	SCAFFOLD115964_5023	0.46	0.36	TGCTCCTTCACTGATGAAAGGAAATCCAGAGGACCAAAGGAAACCACCACTAATGAATGAATTAAGGTGGAAAAGGGAGAAGTAGGCTCCTCTGAAGTGC[A/G]TTTACAGAAATACAATCGGTGAGAAAGAGGCTGTTTTCTGTGTGGCATTCAAAAACATCATTATCCTAGAAGAAAATTATCATATTTTTAAAGAGTTCCT
24	11092077	SCAFFOLD106552_3434	0.14	0.15	TTGATTACACTTCCTATTCTTAACTGACAAGATAATCTATCTGCTGGTCACATAGAGAAGAGGACATGTATCATATAGGCATATAACATAAGAGTGGTTC[A/G]GGAAATCCACAGTAATTATGTCAAACTCCAGAGTCAGTTTACAAAATGGACATTTGGGAGAGAGTAAACTATATCATACCATTTCTGTCCTTTATCGAGC
24	12830897	SCAFFOLD61795_5222	0.37	0.27	ACCGAAGCTGCCTGGCTCACTCTCAGATACCTTTTATGGCTTTTACTTATTTTATATATTTGCTTCCTAGATATCTTGAAATCCTTAATCAGAACCCCAG[A/G]TGCTGCTGCACCAGCCCTCACTGTACCCTGAAGACCAGATTGAGATGAACTTTCATTGGTCCCAAGTACAAGTGAATCTGGGAACCTCCAAATTGGTTGG
24	12899221	SCAFFOLD255245_11962	0.12	0.07	GATTTCCCCAAATGATTGTGCTGCGGCAATAGAAAAAGAAGCATCTGCAGCAGACTGATTGTATCACGGTGTTTACCAAGAATGAAGGACTGTCACAGGC[A/G]GGCTGATCGGAGAAATAAGCTGGAAGATTATTATTATTAAACAACCAACTGCTTAAAGACCCACACAGCATATTACTGGGAAAAACAGAATAAGTTGGTT
24	15870015	SCAFFOLD150215_5759	0.31	0.34	GTTTCTTTATAAATTTGCATTACATGAATAATGCCTTGAAAAAGGTGACCTCCTCTGGCAACCCTGTATAAGGCCACTATTCTTAATCACTCTCTTCTGG[A/G]TATCCAAGTTCACAGCCTTTAAATGACAAAAAGTATACAGGTTTTCCACAGCTATGTCAGAATCTGTTCTGGGCCCATTAAGAAAAGAGTGAGTATTTGG
24	17185247	BES10_Contig500_955	0.20	0.22	GAAATTTCCTTCAGATAACCTCAATTTACATTTTAGAGGGGAAGTTTTAAACAAACCAGGAAATAAATGTAATCATTATTGTCACATTAATTTAGCTCCC[A/G]AAGCTGTGGCATTAACACTCATAAAAGCAGCTAAATCAGCCCACAAATTGCATCATGTGATTTGTCATGCAGGGAATTCACATGCAAAGAAGAGGTGTCG
24	18434881	SCAFFOLD264527_1185	0.15	0.11	TGTTTTCTTTTTCAAAAGTTCTTACAGCAGAAGTAAGAGAGCCCCAGGCATCTAAGAAAGGTCTCGTTTTTTTTGCTATTTCCTTTTCACTTAAAAAAAA[A/C]ACAAAACACTTTTTTTAGACAATAAGGTAACAATAAAGCAGACTGTATCTGCATGGCCTAAAATAGTGAGGCACTGCCTTGCAGGTGAAGGTGGCAGAAG

24	18434911	SCAFFOLD264527_1215	0.25	0.31	AAGTAAGAGAGCCCCAGGCATCTAAGAAAGGTCTCGTTTTTTTTGCTATTTCCTTTTCACTTAAAAAAAAAACAAAACACTTTTTTTAGACAATAAGGTA[A/G]CAATAAAGCAGACTGTATCTGCATGGCCTAAAATAGTGAGGCACTGCCTTGCAGGTGAAGGTGGCAGAAGCCCCTCCCTGCAGACAGGATCATCCATCTA
24	18492634	SCAFFOLD163379_620	0.44	0.18	TTCTTGGCTATTCTTTTTATTCTCTTTTTAACTATTATCTTTTTCTGCAAGTTTCTATATATTGATTCTGTGTTCAAACAATTCTCATAAAACTATGGAC[A/G]TTCAAAAATGTGAGTAATCCAGATGCAGTGAAGCAATTTCCCTTAGCTCCATCCACTCAAAGTCATGTCATATGATTAGGTAAACCCAGATTCTGCCTTG
24	18493192	SCAFFOLD163379_1178	0.07	0.18	CAGTAACTTTTTAAAAAAGAAATAAACAGCCACTTTTAGTATATATATAACTTTTTTTATTTTGTGTTTTACATGAAGTACCAGTCAATGCTGCCTGTAT[A/C]TTAGAATCAAGTGGGAGGGCTTTAAAGAACATTAATGCCTGGTTTCCAATGCTACAGAGGTTGTTTTGATTGAGGTAATACTACATCATGATAAAATAAA
24	18753500	SCAFFOLD137719_496	0.36	0.49	ATACATCCAACAAAGTAAAAAGAAAAATAAGCATCCTTTCCTTGTTTATGTGTTGTTTTAAGTGATTTCCTTCTATGCTTCGAGCACCGTATTTGTTTCC[A/G]TGGGGCAGATACGTGCCCTCCCCTTTGTCTTGATTTGAGATAGAGACAGAAGGATCACTGCACTCTCCTTCTTACTCACATAGCAGACGATCCACATGTC
24	19254677	SCAFFOLD246697_4076	0.30	0.43	GAGTGACAGCCTTATGGCTTTGTGGTTACATCCTGAGGATAATTAACTCTCCTAGGCCAGCTGTGACCCCTGCTGGGAGTCAGCCTGTGCCCAACATGAT[C/G]AGAGAATTGGCCCCAAATGAGGCCAGTACTCTGGAGGCTCTCTAGTGACTTCTGATATTTTAATGAACCTAAGTCTCTGCCAAATATGAAAACCAAGGCA
24	21551826	SCAFFOLD734_797	0.39	0.29	AAAAAAAACAAGAAAGAAAAGTGTGCTCGGGCAGAGCCTGGGGGTGCCTGGTTTAAGGTTAGTTATAATATAGTTTTGCTAAAGTGACAAAACAAAGGGG[A/G]CCTCCTGCAGAGAGCTTTGTCCTGCTGAAAGCTCCTCACAGAGGCTTTGGTTACTCCCTCCTTGGAGCAACTGGGTATGGTGGTTTCTTCCCTCTGCTTC
24	22207910	SCAFFOLD111047_8389	0.32	0.17	GCTGGACACCCAATTTGGGAAATACCAAATTAGCTGACCTCCATGACCCTGAGATAGTGTGATTCTTTAGTAACTGGATAACATTTTCTTAGGGATTAAG[A/T]CTAAAAGGCACACAGGAGATGAAAATAGGCCAAAAGGTAAATGAAAATTTCAAGAAAACGTGATTAAGTCTTACTTCTTAGGGTAAATATAAGATGAGTA
24	24095221	SCAFFOLD35926_1498	0.48	0.41	GGGCCTGTGCAGATTTGTACTCAGTTGAACACAAGCTGCATATTTTCCTTCCTTTCCACAAACTTAAAATACATCAGTGTTCATAACAGCTCTACATGCA[A/C]CTGAGATAGTGTTATGGCACTTATATCTAATTTACAACTCATTTATTCCTCCTGCTTATCTCATATTTCAGGGACCAGTGTCAGTGTTAGTATGGTGGAA
24	25720917	BES2_Contig374_1412	0.40	0.37	GACTCAGCTAAGAGGGCGCTTAAGAAGCTATCACAACAAAACCCAGAGGTGCAAAAACGAAAACAAACATGTCACATGAATGGAACCGAAGAGTTGAGAC[A/G]CGAAGTCAGAGATGAAGCAGAGGTCAGATCACGGAGGCCTTTATACAACCATGCCAAGGCGTTTAGGCCGTATTCTTCGTAACATGACAGAAGGAATCTG
24	25721034	BES2_Contig374_1295	0.45	0.39	GCAGAGGTCAGATCACGGAGGCCTTTATACAACCATGCCAAGGCGTTTAGGCCGTATTCTTCGTAACATGACAGAAGGAATCTGCTGTGGGGTTGAGAAC[A/G]CACCAAGTTAAGAAAACAGGTTCTGGTGTCTCTTTCCCTGTTTAAGGCTTCCAAACCAAGCCTGCTAATACCAATCGCGTATGTTATGGAGGACACTGTA
24	25721111	BES2_Contig374_1218	0.26	0.38	GAATCTGCTGTGGGGTTGAGAACACACCAAGTTAAGAAAACAGGTTCTGGTGTCTCTTTCCCTGTTTAAGGCTTCCAAACCAAGCCTGCTAATACCAATC[A/G]CGTATGTTATGGAGGACACTGTATTCCTAATTATTTTAGGGTAATGTATTTAGCTACACACTAAATTAACACTAATTGAACTTGGAAGAGGTTCAGAAAG

24	26845535	AJ505157-BULGE100-78	0.48	0.42	TACCCTGCAACCTCACCAGTATACAATATTCATCACTATTGCATCTACCACTAAACACAAACTAGAATCAGAGAAGTCCTKGACTCCATAGGAACTGTAG[A/C]TGAAGCTCCAGCGCAGGATCGAGGCTCACACAGCGCCAGCTCACCCCTAGTMRGTCTCACTCTGTTGCCACTCTCTT
24	27745470	BES10_Contig784_2526	0.44	0.36	GTAATTTCAGTGTCACATGCAATGCTCTGGGCATATATATGAACTAAATGCCTGCATCATAAGTAGAATACTTAGAAGATTCCATGAACAGCATTATTTT[A/C]AGAGCAAAAAGTGAATAATTATATAATGTTATCTTTCTTCCCAGTATTACATTTTTCTGCCTTCAAAGAATGTCCAGAGGAAGGTATGATTTGCCTGCAG
24	28071362	SCAFFOLD10637_3690	0.43	0.49	TACAGTAGTTCCAGAATAAACATTTCTAAATTTTTTGTATGTATTTGATCTATTTCCAGAATCTTAAAGTAGTTGTCTTTGACAATTATGTCCATTTATC[A/G]TTGTTTTTCAAGGACAGTTATATTGAGCTCCTTACTCAGCCATTTCAGAAGTTTCCCCTGCTCATTCTTTTGTACTGTACCTTGCATCTCTCAATTCCCT
24	29624478	SCAFFOLD105096_24896	0.44	0.47	CTGAATTAACAACATATGAACAATTCAGGAAATTTTAATACTAACCTTATTAATTATTAACAATGTGGATTCAATGAATGTTAGACCCATATGTGCATCA[A/G]AGGTAAGAGATATCTTTAGAGGGACGATATATATTAACAAGGTCTCATATATGACTAGGATTTCAACTAGGTGAGAAGAAGGAATGGGAAAGTTTCAGGC
24	30578158	SCAFFOLD115575_19820	0.13	0.24	ACTGTATTACACGAGTTTCAGGATTTTCTGAAGCCTCACTTGTCAATATTACCTGATACAAGGGAACGAGAATACAATTTATTTTAACAAACCTGAGTGC[A/G]TACTTTGTGAAATGCTCCTTGAACAGCAGAACACGACATTTAAATTTTTCTTTCTATTGGACTTTTAATAACTATTATCATTTTAAAAGCTCATCACCAC
24	32277266	SCAFFOLD270318_11891	0.26	0.47	TCTGCCGTCCATTTCGGAGTTCTCCATGCCTCCTCTCAAGGCTGCTCTTGATGGCCACTTCTAATGCTATTAAAAATGTTAGGGGATGAGAACACTCAGT[A/G]GTTTGCCCCGGGCCCCTCTTCAGGCTCTGAGAGCACCAGTATTTCACCTTGATTGCATTTGTTCCATGATAATATTGGATGAGTTTGTTAATTTTCTATT
24	32277736	SCAFFOLD270318_12361	0.23	0.46	CCACCAACAGCAGTGAACTAGAAAAAATATATAGTTACTATTTTATTCTATGTGATAAAGTCTGTCAGATTTTTTAGAGTATAAAATCTGTTCTTCAATA[A/C]CCTCTGATTGCTTTTTAAATAATCGCTGATATCCATAAAAAAGAATGTCCTGGCAGTGCTGAGAAAATAGCATTTGAGAGTTGACTAGAAATAATGAGGA
24	32302760	SCAFFOLD126059_2034	0.33	0.50	CCTAGTTGAATGCACTCCATTTGCCCTCACAGTCCAGAAAGGGGACTCCCTTTTGTTATGAGGGACGCCTCAGGAAAATCCCAATGGGGCTTTGGACTAT[A/C]TAAAAATTAGATATAGAACAAATCTTTCTTCTTACAGAAATAACCCAGTGATCAATGCTTTTAAATAAAATAGCTCAAAGTGGCTGCCTTCCTGAAAAAT
24	32302914	SCAFFOLD126059_1880	0.32	0.50	AGTCCCCTTTCTGGACTGTGAGGGCAAATGGAGTGCATTCAACTAGGAGCAGATTCCTTCCTTCCTCCCCGTCTGTTGAAGCCAACTGTCACCTCTTCGA[A/C]TCCTCCAATGTCTGACAGTTATAAAACATTTTTGTTCTTCAGTTGCCAAGTCATGTCTGACTGTTTGCGACCCCATGGGCTGCAGCCTGCCAGGCTTCCC
24	33101881	SCAFFOLD176137_10399	0.43	0.23	GTGTGTCTGTGTACACGGGCACTCTGCCTGCCTCCCATCCTGGGAAGGAAGGAAGGCTCCTTCCTTGTGCTTAGGCAGCAGAATGCCAAATGTGGGGGCA[A/G]GGCTGGAAGAGCAGGGCACACAGGTAAGAAAAGAGGCCAGTGCCCCTCAAGTCTCATAGCGTCCGCGCTCCTGGTGTCACATGCCACAGCGTCCAGCACG
24	33101980	SCAFFOLD176137_10300	0.44	0.24	CTCGTAGCATTCCTGGCCATCTGGGCTGGGAGACCGAGACCACTGTGGCACCGAAATAACCTTGGCCAGTTCTCCCGAAACTTCTGCTTTAGGAACTGTC[A/G]TGCTGGACGCTGTGGCATGTGACACCAGGAGCGCGGACGCTATGAGACTTGAGGGGCACTGGCCTCTTTTCTTACCTGTGTGCCCTGCTCTTCCAGCCTT

24	33760156	SCAFFOLD216926_3725	0.42	0.47	TGCCCAAACATGTTTGTGCCTGTTGCCTTTATTAACTGCAATTATCGAATTCATTGTTTACTGTGTAAACAGATGAAACAAGACTCCAAAAAGTGTTATA[A/T]ATGAAAAATAATTATGAGCTTTGAAAAGACTGATAAAGGCATATGACTCTGAAAGCTCCTTTAGGATTAGATTAAAAGCTCCTCAGAGTGCTCTGCCAAT
24	33760646	SCAFFOLD216926_3235	0.43	0.50	ATACCAAGTGTTCTAACTGTCTTGGGAAAGCAATGTATGTTGTACTGAATATCAGAAAAGGAATTGAAGGAGCTAAGTGAGTGTATTGGAGGGGCCTCTT[A/T]CGGACCTTGATGAATTACTAGTGATTATTTAAAAAGTCAGTTAGGGACGTCCCTGGTGGTCCAGTGGTTAAGAATCCACTTTGCAATGTAGGGAGCATGT
24	34453326	SCAFFOLD155891_775	0.30	0.24	CGGGCTACAGTCCATGGGGTTGCAAAGAGTCAGACACGACTGAGCGACTAACCCTTGCTTCCACTTCCTTCAAGTCTATCCACTCGATGTCCTTTAGGTC[A/G]GAGGTTTTGCTGACAACTGGTTATAAACACAGATGATGACACTGATAAAGATGCTTCTCTTCCAGCCTACTGCTGCCGGGGGATGGAAGGGTCTAGTCTT
24	36156804	BES2_Contig463_697	0.08	0.13	TATACTGACTCCCAAGCTTGTATGTTCTGTAGAAAGAAAAGAAAACCTGGCCTGATCTCAAGAGCATTCCTGAGTAAAGGAACATATTCTTCCTGATTCA[C/G]GTCACAGATCTAAAAAAATGTGTACATATAAACTACTTTGTGGGAAAATATTAATCATATAAAACCAACTTCCTAAAACAGAACTGAAAGTAAATTTTCA
24	39061144	SCAFFOLD322397_15135	0.49	0.44	AGCAGGAATCTGGTCTTTCTTGCTTGCTGCTGTAACTCCGTTAACTAGAACAGTGCCTGGCATACAGTAATTACTTGAAATATTTATCAAATGAATAAGC[A/G]ATCGTTCCCAAGTATTTCCAGGTGTCAGAAAAGAAAATTTGATCTGGCCTAGCAAGTATAATTCAATAGTGGATGTCACTGAGCAGAATACTAGATTTTA
24	40979414	SCAFFOLD90052_4931	0.25	0.32	TTTACACTGGAAAGTAGAGATAATTCTTTGGTCTGGGTCCTGCAGAAAAGCAAAATATGAGGGTCTAGCATCGATGTTGCAACAGCTTCTTTACTATCTT[A/G]TCTTATTTCTTTGCCATAATAGTCTGTGTAAAATCAAAATCTAACCTTTTACCAGCATCTTTCGACTCGAGGCTCCTATGCACATACCAGGCCAGTCATG
24	42134360	BES9_Contig478_702	0.37	0.42	GGGTAAAAAGAGGTGAAAGAGAGAGGACTTTGAAAGCCAAGGTCAACTGTATAGAATTGCAGTGTTGACCCATGGACATGTTGATGGCTTCTCAGAAATG[A/C]CCAGGAAGTAGTTTTTCATATTGTGACCCTCCTTTGATTTTTTTTTTTTTTTGCAGGCTCCTTCATGTTGGTGAACACATCCGGGAGACCCGAGGGACAG
24	42188487	SCAFFOLD300392_11877	0.15	0.19	CCTCAGGACCAGGACCAAGTGTGCTGGTGAGTCTGGGGATCCTGGGGCCGCTTGTCCGTTTCTGATGGGGTCTGACCTGCAGCCTCAATGCAGGTGGCTC[A/C]CGCTTTTCTCAGCTCTGCTGGGGCCCTGTCTGGGCACAGGAGGTCCCTTCACCTGTGGGCAACCACCTGTGGATTCAGACCTCAAGGCTTGGTCTCACTC
24	42425150	SCAFFOLD150108_10044	0.17	0.13	AACAAATCTGATGTGAAAATGTCCTGGTTTATTTGCGACTATTCTAAGAGATGGGAACAAATAAATTTCTAAATCTCAAAGCAAATCCAAATGGAAAAAG[A/G]TCATAAAATCAACTGACCACCTTTTTTATTTCGTCTTTGTGTCTGTAGCCACCTTGTAGTGATCAGGTGCCATTTCAGCAGCAGCGCATAGCTACACACC
24	43270638	SCAFFOLD166152_3237	0.13	0.24	CATCCCAGGAGCTCCACGCGGCACAAGCCCTCCAGGAAAGACACCTCTGCCCTCCGAAGCCTCTGTCCACTCCGAGCCCAGCATTTATGTTTCAAATTCC[A/G]TGACCCGTGGCTACTCTGACACCCAACACGTACCCACCCTAGCACAAGTAATATGGCACCGTGTCTAGCGTCACCTCCTATGGCCACACACCAGTTCATC
24	43337200	SCAFFOLD61272_2778	0.07	0.19	TGGGGTTTGCGTGTGGGTGACCTGGAGCGGCTGGAGTCGTGTGTGATGTGTGTGAAGGCACTGGACGTCTATCCGCCCGCTGCCCCAGCCTGGGAACACC[A/G]GCGACGTGGTCTGTGGGCCACACAGGTGTGTACGCTTCATAAGGGGGCTTTGTGGAGAGCCGTACTTTCTGACTATTACGGCTCCTCAATGGGTTACAGG

24	44245710	SCAFFOLD105690_6637	0.31	0.37	GCAGAGAAATTGAAACTGTTGAAGGGGAGCAAATGGAAATACTAAAACAGAAAAGTGTAATAACATAAAAAATTCATTGGATGTACTTAACAGCATGTTG[A/G]ACATGAAAGAACAATTTGAAGACTTAGAAAGCAGATGTATAGAAATGATTTCATCTACAAGGTGGGGTGGGAGGAAGCAATTGAGGTGAAATGAGCAGAT
24	44245753	SCAFFOLD105690_6594	0.31	0.38	TAGATGAAATCATTTCTATACATCTGCTTTCTAAGTCTTCAAATTGTTCTTTCATGTTCAACATGCTGTTAAGTACATCCAATGAATTTTTTATGTTATT[A/G]CACTTTTCTGTTTTAGTATTTCCATTTGCTCCCCTTCAACAGTTTCAATTTCTCTGCTGAGATTCTTCATCAGTTCATTTACCATGCCTACATCTTATTT
24	44502612	SCAFFOLD265613_7256	0.43	0.45	ATATGACTGACCACAGCTTAGAAGCCTTTCAATTTTCTTTTCTGACATGTTTGAACAAGGAGATGAATGAAATGTATTTCACAGAGACCACAAAACCCGC[A/G]TCAGGTGTGAAAGTGCTTTACCTGACAAGTCAAAACTGTAAGACATGCTAAGAGGCCGCGTTGCTGAAAAAAGAAAACAAACAATAAAAGGAAAGTAATT
24	46427029	BES5_Contig460_1314	0.47	0.38	ATTCTAGATGTTCAGGTTACAATCAATTTTAGCTGCTCCCTCGAGTCCTTAGAGATGACTTTTGAAAGTTCGTACAAGCTTTGGGTTTTCATTACTTTTT[A/C]TTCTACTCTCAATGCTTAGATTGGCACCAATTTACCCTCAAGAGGTTCTTGTGAGCCTTAATTGAGGACCACAAAAGATAGCAAGATCTTTAAGTGAGTA
24	47738919	SCAFFOLD205142_480	0.28	0.45	TGACACCAGCTGGTTAAGAAGTGGAGGGAGGGAGAAACGCTGCTACGTGGTGGCCAGCAACCACCTCGACTGGAGTCTGTGGATCTGCTGAGTTAGGTGC[A/G]GCTGACAGAGCGAGTGGGGACCCCAGGCCAGGCCTGGGCTGGCGTGGCCGGTGAAGTGGCTCCCTGGGCAGTGGGCCCATGGCTCAGGTCCACGCTCTCA
24	49539218	SCAFFOLD268943_393	0.37	0.38	GAGGCCAGATTCCACCAGGAGGCTGACATCACGGCCTGGTCTGGGGAGGGCAGGAGCCAGCAGTGTGTGCAGTGCAGCAGGAGGGAGTGGGGCTCAGGGC[A/G]GCTCTACCACAGACGGTTCACTGGGAACTCGCTGCAGCGGCGATAAGCTGGAGACCCCAGGTATCTTAACCCAGCCCCCCAGACAGGGCCCAGGCTGGAG
24	50386868	SCAFFOLD205003_54121	0.47	0.47	TTACTCCCTTAAAGCCCAGCCCACTAATACCATCACCCTGGGATTAGGGCACAGTATGTGCAGCTGAGGTCAGAGGATACGTTCAGTCGTAACAGTTCAC[A/G]TTCTCCAGGGGGAAGGCACACTTGCTAAGCACTGGGGCCACCAGCAGTTCCACCTCAATCCACTTTCAGGAACTGAATGAAGTGATTGAGAAGCTGGATC
24	51649884	SCAFFOLD200633_9674	0.48	0.43	CAAAGCCCTTATCACTCCCGATCTTAAGGATGGAAAGAAAGAACCAACAGCACTATCAAAATGCAACTGACAAAACTCTCAGGGGCTTTTAACCTTTCAC[A/G]GCCAAACGCTCTCCCGTTGGTGCTTCTGTCTTAGGTCCACTCCTTTTGTTGCTGTTGTTTAGATGGTGAGCCGCGATCGACTCTTCTGTAGCCCACCAGA
24	52527489	BES5_Contig611_2321	0.34	0.38	AGCCTAAAAGACACATTAAGTAGAAAGTCTCTTTAATATTTTCTTAGTGATTGAGATGATGGATGAGCAAGTGAGCCAGTTGAGTTAGCCGATGATGCTG[A/C]AGCTGTCATTAAGTAACAGGTGAATCATTAACTAGGAACGCAGACAGGGGGCCCGAACTTCATCATTTTTATTCCCACCTTTGGGGAAGCCTGGCAGCAT
24	52527942	BES5_Contig611_2774	0.43	0.38	GCAGTTGCAAGCATTAAAATGGATAGAACCTCCTTCTCTCCTTCCCTCTCCCTTCTTCTATGCAGCTGTCATTCAGGGCTGGGAAAGACACAGGACCCAA[A/G]TGAGGGGTGTTGCGAGTGAAATCCTTCCTTTCTAAGCACACTGTTTCCACAGCACAGAGCAAATAGGTGCCTGATAAGAGGGACTATGAATGCTGAGCAC
24	53162369	BES1_Contig612_1163	0.12	0.10	AGGCAGGGCTGGGAGTTGGCTTCTTCTCTTCCTTGAGAAGAGGTTCCATTTGTCACCCTCTCCCTCTGTCTTTCTGGGTAGCCCTCTCCCTCTCAGCTTG[A/G]CTCTTCTTTATCATCACTGCTCTTGATATCCAGAGCCCTTTGGAATATTGCTCCCTGGAAATTCTAGACTTCCAGGTTGCTAAGGGAGGTTGGTAGGGAT

24	53162573	BES1_Contig612_959	0.27	0.37	GCTACGAAGCTCCCCAGGGCCTCAAACTCAATTTCTCCCCCAGCTTCTTCCTAAAACTAAAGTAAACACACTGGAGCACTGTTTACCAGAAGCGGCCAAC[A/G]ATGCCCCAAGGAGCAGTATCCCTGAACTTGTAATGCTGTCAGCAGAGAAAATGCTGATGCGATATTTGACACCCAGAGTTCAAGGGAGAGAGCAACTGGG
24	54778858	SCAFFOLD290859_1103	0.43	0.38	TTCAAGTTTCTCATTTGCAATGCCATGCTCATCCTTCCTCTCTCCCTTCCCTTTGTTATTTGGCCTTAAAAAAAAAAAAAAAAAGGACAAGCTCCATATC[A/G]CTGTCCAAGTGCTGAACTCTCACCAGTTTAAAAGGCAGTATTTTTATAGGGGAATCGTGAGGCTGTCACATTGATTTCAGGAAGCCTCGGTTCCATTCTG
24	55034641	BES2_Contig394_660	0.10	0.10	GGAGTTTAAGGTTCTAGCTCACAATGAAGATGCAGTTTTTAAAGAGCATCCACCAGATTTAGGGTGATGTGTCAGAAGTGTCAAGTGATGTCTTCCCCCC[A/G]TTCCTCTCTGACTCTGGGCAGGTGTGGTACAGAAGGTTTCCTTGACAACAAGCCTGGCGGTCGCCACTCACCAACACTGCCTGCTCTGAGTCAGTGAGGA
24	55034747	BES2_Contig394_554	0.04	0.03	CCCTCTGTGGATGTGGCAGACCCATACCATCCGAGGAAGGGAAGGAAAATGGAAGGCAGTTTCAAACAGTACCTTCTCTTGCGTGATGTCTCTTCCACCA[A/G]CAGGGTCCTCACTGACTCAGAGCAGGCAGTGTTGGTGAGTGGCGACCGCCAGGCTTGTTGTCAAGGAAACCTTCTGTACCACACCTGCCCAGAGTCAGAG
24	55552331	SCAFFOLD46051_988	0.45	0.40	TTCTTTAAATTGGAGTCTCTACACAAAACGTTTTTTAAATTAGCCTTCGTTCCTTCATTTCCAAAGTCTAACACACATTACCACTGTTTTACACTAGCAT[A/G]TTACATCTGATTTTTAATTTGGAATAATGTGTCTTTCAACCCCATTTATTTTATATGATTTGTTAAGAACATCAGTCTCATCAAACTAATTATCGAAGGC
24	56094597	SCAFFOLD141291_440	0.27	0.40	GTTAATATACAATATTTGTTTTTCTCTTTTTGACTTAACTTCAGTCTGTGTAACAGGCTCCTGGTTCATCCACCTCAGTTTAACTCACTCACATTAATTC[A/C]TTTTCGTGGCTACTAGTAGAGTTTTAAATCAATTCATTTCTATTGTGTTTTTGATTTATATGCTTGTGAAGAAGAAAGATTGAATTCCCTAGTTTCAGAT
24	57963324	SCAFFOLD240536_8598	0.13	0.13	TTCTTTGCCCACGCTTCCCAACAGGGGGCGTTTGCTCAGGCTGCGGGCTCTCTCGACACCCTGTCCTTCGGTGGTGATGACCAGGGTGGCGTCTATGCCC[A/G]TTAGTCCTATCTGACAGCCCCAGCCCTGGTCTCCTGCTCAAGTGCTGTGCCTGCCCACGGGGCATCTCTTCTGCTGATAAGGCAGGCGTTTGGTCTGTTT
24	58185821	BV10477-364-R	0.49	0.39	GCTGCCTTCATACTTTCTACCCACCTAAATGAGACATTCCTTTTATGTCTAGCCCTCACTCCCAGTGTAAACCTGTTGTGTTTCCCTGTATTTTCCTGCA[A/G]TGTTGTCACAGAGGAAGTGGGGTAGTGTAGTTCTGCCTTGATCACGCTKTCTCTRTCTTAGCTGTTATTTAAAATTTTATTGTCTTAAAAATTTTTTTCT
24	58588510	SCAFFOLD190177_28325	0.44	0.07	GCTCCGCCCCGAACCCTTCCAGGCCCTTCACTTACCTTTAAAGGCACCTCCTTCTCCCTGAACACCCACCCCTCCCTGCCTGAATAGCTGGCCCTTCCTC[A/C]GCACCACCATGCCTCAGCTCAGGCGGTTCTGCCTCTGTGAAGCCAGCCCTCAACCTCACTCCCCGGTTCCCCCAGGACTGTAGGTGCTTATTCACTTGTG
24	58635593	SCAFFOLD265_2802	0.42	0.49	GCGCCAACAGCCTCCTGGGGTCACAAGGCAGAATCCCATGAGAGCTGGTTGGCAGTGACCCCTCCCAGGCCCGGTTCCTTTCTCCTGGGCTCATCCAGTC[A/G]CCCTGTGAAAAGCGTGGCTTCGTCAAAAGATGCATTCAACCTCCCCAACTCGGGAGCCTTGTTTACCAAACAAAGATGCTGCAGAGTTTGGACCGGGGCG
24	58635793	SCAFFOLD265_3002	0.49	0.47	GAATGGCCACTGCCCAGAACAGTCATCGGACACTTTCATGTTTGGGGACCAATTAATTATCCCAACCTCTTGATTTGAAAAAGAGGATATTTAATCTCCC[A/G]TTTTCCCCAGAGTCTCAGCCCTTTGACCCCACTTCTTGACCCCTGCCCTTCCACCTAGCGTGTTCCCCAGAGTCCCTCCCGCCCCCAACCCCCTCTCCCC

24	59493016	SCAFFOLD195793_4445	0.03	0.03	TTCATGGAGAGTATCTTCTGGTTGGGGGCAGACCTTAAGGGAAAAAGGCAGATGGGGTTACAAAATAACACAGGGCTGTCAAGGCAAATCTCTCTGCGGG[A/G]GTATGCTGGGCGCTCGCACGCTGGGTTTTGCTTTGTGTTTGGCAGGGGATGTGTTGGGAAAGACCTCCGTGAGGAAGTGACTCTTCAGCTGAGACCCAGA
24	59589744	SCAFFOLD211059_826	0.32	0.33	GGAGATAGTTAAAGGCAGGGACACCTGGCATGCTGCAGTCTATGGGGTCGCAAAGTATATGACACAACTCAAAGACTGAACACCAGCAACATTGTGGAGC[A/G]CGACCCTGCTTTCTGCTTTAGCTGTGTCTGTTTCATCTGCCCGACAAAACTATAAAGAGCATGGAGACATTGTCTTCTGTGTCTTCCGTGTCTATATTCC
24	59961935	SCAFFOLD150363_3665	0.47	0.47	TTGGCAAGAGTTTCTTTTTAATCATGAAGGATACCGAATTTTATCCAATGCTTTTTCTGCATCTATGATCGCGTGTCTTTTGTCTGACACTGACTCGTTT[A/G]CATATGTTGAGCCATGCTTGTGACCCTGGAATGACCCCAACTTGATCATGGTGCCTGATCCTCCTGACAGCTTGTGGGATTTGCTTGGCTAGCATTTTGT
24	60163442	SCAFFOLD42023_828	0.21	0.18	ATTTCCTATCAAAAGAGCAACCTTGTCCTTTGCTGTTAAAAAAAAAAAAGAGGAGGAGGGGGAGACATTTAAAGGAGGATATAAAGTTTAAAAACAAAAC[A/G]CAAAAAAAGAAGGCTTTACAGATAAGATTTCTTCATTTTATTTCATTTTAGAGCTAGGCTATCACTTTATGTAGTAGACCAATGCACTCTACTGAGGAAT
24	60576236	SCAFFOLD142089_7609	0.16	0.15	CGGTTCTGAATTCTGTCCATTCATTAGGGATTTTGAGCTGGGAGCAAAATCTGTCTGTTGTTTACAAACTAACTCCTCCTACTAATAAAGTGCAATTTAT[A/G]TTGCATTTAGGAGGAAGAAGCACAAACACAGTCGGTGCCTTTTTAAATTTCTCGTTCATTCATTCCCCTGGGTTAAATTAGTGGCATGCTGTTTTGGGTT
24	61730315	MC4R-AF265221-1069	0.44	0.35	CAACAAATGATCTCTTTGAAGGTTTTCCTCAGTTCTTGGCTCCGCAGGGCATAAATCAGAGGGTCAATGATGGAATTGCACATGATGAGGATGAGGTACA[C/G]GTTAAAGTGAGACATGAAACACACACAGTATGGGTTCTGGGGACAAGAGATGTAGAATATCAGGTGCAGGAAGAAGGGGGCCCAGCAGACAACAAAGAC
24	63929826	SCAFFOLD272215_4435	0.32	0.26	TGCTCGCCAGGTCTTGGCAGCCTTCTTTGGACCTTGGGGCTCTTTGAGTACTTACAGTCTTTGTTCAAATAAGCAGCCTCCTGATGGCTGGCCCCCATGC[A/G]AGGCCCACATCTGGAAACCATCCCTGGTCTTGGCCCAGGCGTTCAGGCCTAGGGGGTGTGTGTGCGTCAGGTAGAGCCCGCCCGGGGATCCACACGCCAG
24	64091396	SCAFFOLD305155_18811	0.45	0.16	TTCCCCTCCCCCGTCTCCCTCCAAGGAAAAGCAGGTGCTATTTCCAACCAGCAGTCGCAGGCCAGCCTTCTATCAGCCCCAAGTCCCCGCCAATGGCCAT[A/G]TCCGACATGACTGATGAGTTTCTGGATGACCTCTGGGCTTGGGGAAACCCTCAAATATCTTCTGACTCATTAAGACACATACGTATGTCTGGTGTAGTTG
24	64920071	SCAFFOLD292811_537	0.49	0.43	TAGATTTTTAAAAATGGTGACTAGAACCTTAACTATAAACACTCTAAATAAAAAATAAATAAATATTTGGAAGTTTCCATTTTGTACAAAGTCCTGAGAT[A/G]GGTGCTAGCAGATGCACAAAGCAGAATAAGAAAATTTTGCTCTCAGGGAACTTGTTCTAGAGATGAAAAGATACGAACATATGAAAACAGAACTAGCAGT
25	1082329	SCAFFOLD210524_11945	0.39	0.34	AGGGACGGGGGAGCCTGGTGGGCTGCCGTCTATGGGGTCGCACAGAGTCAGACACGACTGAAGCGCCTTAGCAGCAGCAGCAGATGTGGTCAGGCTGTAA[A/G]CCCCACAGAGCGAGCCGTGGGTCCCATGCTTTGCAGACGGCAGCACCTGATCAGCAGAAGCTGGTTGTCATCTCGGTCTCGCCCCAGTCCAGAGCGTCAC
25	1413336	SCAFFOLD126527_1474	0.19	0.21	GCCCCACTGGGCACAAGACCCAGAGGCCGTACCGCCTGTCTGGCTGCTGGGTACTGGGGTCCCAGCCCAGACCATGGTGCAGGCCTCCTGCCACTTACAT[A/G]GCAGCTGAGGATTTGCCACTGCTTTTCGGGGTGGCGGGGGGCGGGTGGAGACGAGCAACCCTGTACCAAGCCGGGGTGTGATGGGCAGTCAGGCTGAATG

25	3625175	SCAFFOLD31537_636	0.23	0.38	GTGGGGGCTGGGCGGGGGGCGGCGGGGCGGGCACCTGGTTGGTGGGCTTCCCGGTGGACACCGTCGTGGGTGGCTGAGTCCGGGCCACGCTGGGGAAGCC[A/G]GCTGGTGACATGCTCACAGGCGAGGGCTGGGGCTGGGCGGGGGGCTGCGGCGTCTGGGGCGTGCTGGGCTGCTGCGTGGGGGTCCCGGGCGGCGCTGAGG
25	3627910	SCAFFOLD31537_3371	0.08	0.16	CAGCAGGGGGAGGAAGGTGTCAGAGTCTGAGCCGATGTGTCGTGTTTGAGATGCTCAAGTAAGAGCTGATGGTGGTTTAGTTGGACGCGAAGCCGTCAGC[A/G]GTGAGAAGCGTAGGATGTTTTTCAAGACTAACAACCCTAAATTTAGGGCGTGTAAGGACAGTTCTGTTTGAAGGCCGCCTGCTAGGTCTGGGCCTCAGAG
25	3698438	SCAFFOLD60020_10226	0.18	0.32	CCTTCAATGAACCCACGTGCGGCAGGGGCACACCGAGGCGCAGCCACACCCTGACAACTGCCAAGCTGGCCAGCACCTCCCATTCTATAGTCAATCTTCA[A/C]GCAAAATGTGCCAAGTTCTATGGGGAGAAAACAAGACTCCAAACCTCCGCCTGGTCTGTCCCTTATCGCCACACCCAGCGCTTCCCCACGGCCCTATCGG
25	4144489	SCAFFOLD55591_510	0.16	0.31	CTCAGCCACCCCCTCTCCCCAGCCCCTGGGGCCCTGGCACAGTGAAGGGGCTCTGAAGCCCCGGGGCTCCAGAGTCAGATGGACCGAACCTGCCTCTGCC[A/G]TGAGCTGTTGTAACCCTGGGGACAAAAGTGACCAAAGGGCTGACTTGACAGGATGTTCAGGAGAGTGGAAGGAGGTTGTGCACACCAAGGGCCTGGGACA
25	7447448	SCAFFOLD115006_8551	0.32	0.44	CAAAGAGTCAGACACGACTGAGCAACTCACTTTCTTGAATGTCACTAGATTCACAGCTTGGTTAGAAATAGTCTCTTCCTTGGAAAGAAATGAGTCATCT[A/C]GGTAAAAAATATACATTAAAGCATGTAATGCCGATACAGGGATGCATTCTGAGCAGTGAGTTTGGTGTTCCCAAAGTAGACATGTTTCTGAACTTAATAA
25	7783367	SCAFFOLD105089_26770	0.40	0.44	ACGCACAATTGACACCATCTATATTTTTAAAAAGGAATCTCACTTTTTTCGCCTATAGAATCTCTTCTATGCTTTCTCTTCTACCTTCAAAACTAACACA[A/G]CATTTGAAAGAATGTCCTTAGGATACATTTCCAGAAATAGGAATGATAATTCACAGGTTATGTATATTTTGAAGTCTTGAGTATTTTACTAGGGCTGGAA
25	7783417	SCAFFOLD105089_26820	0.40	0.44	GTGTGGATCTTAGCTGTTCTTTAGGACTAAGGATCAACAACAGTGGACAAACGCACAATTGACACCATCTATATTTTTAAAAAGGAATCTCACTTTTTTC[A/G]CCTATAGAATCTCTTCTATGCTTTCTCTTCTACCTTCAAAACTAACACAACATTTGAAAGAATGTCCTTAGGATACATTTCCAGAAATAGGAATGATAAT
25	8289512	SCAFFOLD210508_16484	0.20	0.36	CAATGCCTTCTCCAGATTACAGTTTTGGAGAGAAGCTGGTGAGGTAGCATCTTCAGAAAGCTGCTTGGAAATGACCTTTCTCTTTGCACTCCTGCCCTGG[A/G]GAAGACAGTGTTGAGAAAAAGTACCTCTCTCCTCCAATGAAACCGAACTCATAGGTGCTTGGTCTTCTCATCTGTGTGTGTCCTAATCCATGCCTCCCCT
25	8995368	SCAFFOLD300283_16679	0.13	0.21	TATCCGTGGCAGACATTTCTAATCAATCCTGACATTCCATTTCACTGATCCTCACCTCGACTTCAATCTTCTCTGTTTAGTTAAGCTCCAGGCATCAGCT[A/G]CCAACCCAAAGAAGTCAATTTCCCATCCTTTTCCTGTAGCAACACTCCATCCAACTGTGTTTCCAAATCAGCCTTCTGCATTGGAAGGATAATACAATAC
25	11691944	SCAFFOLD100400_31720	0.04	0.09	CCTCAGGAGGGCATCATTTCCATTCGGTTTGCCAAGACACATGGATCAGCAGAAGCCAGGCCTGGACATCAGCAAAGAGGAAGACTGACAGACTTTGGCC[A/G]CCACCAGTGTCCTCTCCCATGAGAATCTGGTTCCAGGCGGAACCCCAACTTTATTTATAGCTCTCCTTTCTCCCAGCTGGAGAGGGAGCTCTGTTTATGT
25	11692145	SCAFFOLD100400_31519	0.46	0.09	TCATGGACAGAGGACCCTGGTGGGCTACAGTCCATGGGATCACAGAGAGTCAGACACGACTGAGTACAAGCCAGCTGTAAAGTATAGTTCCCCACAGTGA[A/G]CCATGTGGCCCTGAAACTATTATCTTCCTCTCTGAAAAATAGGACCCTCAATTCTGCCCTGGGTCATAGGCACAAAGCGTCTGCTGATCTATGTTAGAGG

25	12270018	SCAFFOLD35284_17529	0.38	0.40	ACTTCTCAGAATGATCATCATAGTAATTACTGTGATACCGTTTATTGCCAAGCTTCTGTGTGCTGAGCACTATGTGGAGTGTCCCCCTGAATTACCTCAA[A/C]AGACCCCTGCGACAGCTCTATGGACTAGATACTAATATTCCCATTATGCAGATGAAGAAACAGAGGCTCCCAGAAATTAAGTACCTCCCCTACAGTGACC
25	12571870	SCAFFOLD210415_11596	0.45	0.38	GTTGGTTTCAGAGTCACAGGAGCATGAATACCTCTGTGGTTTTCTGCCTGACGTCATGGTAGGAGGAAATGCTGAATTTCGGTTAGAGGTCAGTGGAAAT[A/G]TTTTTTTTTTTTCTCCCATGTAGGTGCACAGATCCCTGTGGATCCCAGATTGAGCCCTTATCCATGTATACTTGTGAACATACTCTTGTCTGACTGGTCG
25	12715260	SCAFFOLD163726_1130	0.23	0.31	GCAGCGACTGTTGCCTCGGTAGAGAAATTCACCCAGGGATGGGTAGGGTGCCTGGCAGATTTCGACTTAACTCCTCTGCTGAAATCTCCTTGGAGCCTTT[A/G]CCTTGCCAACCAGGGCTTTGGGAGCTCTCCTCCTGGTTCGGCTGGGATGGGGGTGGAGAAGCAGGAGGAGGGAGGTGGGAAGCTGGGGAGGAGCACTAAC
25	12828875	SCAFFOLD105457_745	0.27	0.38	CTGCCACAGCCAGATAAATTAAAAAAAAAAAAATTTTTTTTTAAGAGTAGCCTGAGCTGGACGGTCTGAAAGGTGGAAAAGAAACAGAGAAGAAAGCATT[C/G]CATGAAAACCAAGGAAAAGGTGAGTTTCAAAGACCGGAGAGTGGCCAGCGGAGTCAGACATGAGGTGTAGCTGAGTAGGCTGGAGAAGAGAGTGTATGCC
25	12876648	SCAFFOLD130045_33647	0.46	0.38	CCTCCAGAAAGCCACTCACAAAGCTAATCCCTATGTCCTGGGCAAGGAAACTGACCATAAACTTCTCTCTGTTGCCTAAGAAAGCTTCTTGAAAATAACA[A/G]CTGCCCATGTGCCACCAACTGGGACTGGATATGGTCCCCTAGGCTCCAGGAAGTCTTCTTGACTTTCCCCATGGGCTGATGGTCTGATTGATGGAGGGAA
25	13342187	SCAFFOLD35263_5214	0.23	0.18	AGCATGTGTGGAGAAAGAGTGAAAGAATACTGGTGGCCGCGTGACACAAACAAAGAACGAAGTGGGTTATGTGGGATGGCAGCAAGGGTCTATACAGTCC[A/C]GTCGCCCTGACTTGCCCCCCTCAAACCCTGGAAAATTCAATCCATCATAACATGAGTTATATAGCATGAGCTTTTGGTTATAAAGACTGAGTTTGAATGC
25	13556647	SCAFFOLD146466_8003	0.47	0.45	TCTCTTATGTCTCCTGCTTGGCAGGTGGGTTCCTGATCACTAGCGACACCTAGGAAGCCCTTATCAGCTATAAAAAATTTAAAAATACTGATTCCTAGGC[A/G]TCACTTTGAGATTCTGATCTAACTGGTCAGGGGTGTGGCCTGGCATCCTGATTTTTAAAAGCTCTCCAAGCAGCTCTAATACGCAACCAAGTTTGGGAGC
25	14047149	SCAFFOLD140031_10945	0.49	0.01	ACGGAAGAAAGGATTCCTTCTCGATATGCTTTTGCAGCACTGTCCTTTGGCTTCCTCCTCTTCAAATGAAAGCTGTGTTTAGTTGGTGCTGACAAGTCAG[A/G]TTATTGATTAAGAAACATGACCGTGGCGGTTGGAAGAAATTCACACCATTTACTGTGAGCAACTGGATTAGCACCTTTAGGGGGTGGTGTGGGAGAGAGA
25	15192897	SCAFFOLD171134_7987	0.21	0.17	CGGAGGAGGAGTCCCAGCGCATCAATGCCAACCGCAGGAAACTGCAGCGTGAGCTGGACGAGGCCACGGAGAGCAACGAGGCCATGGGCCGCGAGGTGAC[A/G]GCGCTCAAGAGCAAGCTCAGGTGAGGTGCCGGCCCCCAGGGTCCTCGGGTAGCAGACGGGGTGCGCGGGGTGCCCTGAAACACCCACAGTGGCTCAAGGG
25	17823838	SCAFFOLD75765_18884	0.38	0.38	CTTCACAGCCACCAGGAAAGGGAGAGGGCAGAAGTGTCTCCACAGCATCACCAGCACCGAGTATTAACATGTGACGGTAGGGTGTGGGATTAGTGTGGCC[A/G]CCAAAACAATTTTTGCTTGCGTTTCTTTGATTGACTATCTGGCCACATACTGAATTACATCTCCTCCTCTGTGTGGAAACACTGACTTTCTTTCTATGTG
25	18572839	SCAFFOLD206554_1248	0.12	0.24	AAAGGCGCTTTCTGTTTGGTCATATACTTGAAGAGTTGGAAGATGTCTCCTAGACTGAGGGATTTCTGTCATACCCAGGGTGACTCCCTGAGTGAAGATC[A/G]AGCTCACAGAGGGAGGGCCATGGGAAACTGCTCCTGCTGTGGTGTCCAGCCTACAGGATAGCGTTGTCCTTACCCTTAGCTGCCACACCTTGGTGTCTGC

25	19691933	SCAFFOLD45092_17416	0.04	0.03	GGACAGCATAAAAGCAGGTCTTTGCAATGTCAGAAGCTGTTTATCTCTCTCTAATGCTACTAAGTCCTGGACTTCTCTGGGGCAGTTGCAGTCTTCCAGC[A/C]GGAATACACCTATGCAGAAGCAAATAAACCAACATCTTGCATTTAATACCAATTCTAAGTCTTTAGCAGTTGGTTCAGAACTGGTACCTGTTTCAACTAC
25	20020871	SCAFFOLD234225_9665	0.18	0.14	GGTCTTATTCTCAGGTGATGGGGTTCTTTCTTCGCTAGGTGAAGGATACTATGGCAGAGTGGGGTGGGTGGCTGTCAGAAGCAGGAGCTCAAATCAACCA[A/G]CCATTCTCACTCCAGGTAGAAGTACTGTCTGTGGTAGCTCAACAGATTCTCAGCATTCAACAAGCCATTATCCGGAAGCTAAAGACATTCATCTTTGAAG
25	20949306	SCAFFOLD292164_3242	0.35	0.31	GGCTCCAGGGAAATAAGTGAACAAGAGAGATGAGGTTCCTGCTCTCGCTGGGCTTATCATTCTAACAGAAGGAATTGAAACTTTTCTATTCCCAGATCTA[A/G]GTCTCACCGTATATTGTTCTGGCTGAGAAGAAACACACTTTTAAAAAAATTAAAGGCAAACTTGAAGTATTTCTAAGTGTAATATGTAGATAAAATTAAA
25	20949514	SCAFFOLD292164_3450	0.35	0.30	GTGCTTGACTCAAGCACCCAGAAAAGTAAAAAATGACTGAATGATTGCCTTCTATGACTTGTGGGGTTTGTTTAAGTTTACTTTGCATGTTAAACGAAAT[A/G]TTTCTACGTAGCAGACTACAGCATAATGCTGCAGAGTAGGACCCAATGGAATTAAGTGCTGGGATGACCCACTCAGAGAGAAATAAGATTTAAGGGGTGT
25	21852821	SCAFFOLD215107_4516	0.08	0.13	CGCTTGTGGAAGAAGGGAGGCTGACTGGCGAGGGGTGTTGGTTTTGGAAGAGAAAGCTGAAGTGGCTGCAGGGAGAAGGGGGCATCACTAAGGAGAAGAG[A/G]GTGGAATCTTCCCAGAGCTCTGGAACTGGAGGCACCAGGGACCCAGGCGGGGTGATAGATGTGGGCAGAAGCACAGTCAATGTTAGAACTCTCTGGAAAC
25	22943705	SCAFFOLD30035_27012	0.23	0.27	TGCACTGCAGGTAGATTCTTTACTGTCTGAGCCACCAGGGAAGCCCTCCAACTTCAGGGATCATGGACCAAAAGCATGACTTCTTGTGGTGCCCTGTTTC[A/C]TCCTTCCCCTCTCAGAGCAAAACTGGGGGCTCATCTCAGCCTCCATCAAGGACATGCCCTAGAACAATTTTCCCCAGACTGGCCCCAGCAAGGCTCTTTC
25	23512347	SCAFFOLD166019_6545	0.45	0.43	ATAGTATATTAATTAGTTTAATTTGATCTATATATGTGTTGCTGGAATGCTGTTAGATTGAATAGATATTTCAACCAACATTTGGAGCAGGAGGGCAGAA[A/G]GTCACTAACTGTTTCTGTAGGATATTCAAGACTAGACCCTACATTACTTTATTCAGAATCTCTGAAACTTAGTGCTTGGCTCTCAGTAGTTGTCAAAACT
25	24240627	SCAFFOLD140507_1982	0.04	0.05	TCCCCTTGCAGCACATTCTCTGAATGGTCAGGCCCTTTTCCCTTGTGAGGAGCAGGTACTCAAACTTGTTTAAGCAGAGAGGGAATTTCTCGAGTCACAT[A/G]GTAAGAAGACTGTGGCCCTCATGGATAGAACCATGGTGGATATCGGAGCTGAAGTTCTGTCCTGCATTTTGTCTGTCTTCCATCTCTCCTTACCTTAACT
25	25204090	BES2_Contig416_1031	0.39	0.33	CCAGTGAGAAAAGATTCAATTGTAATCCGACTAATGCATCTCCAGAAGGGTTGCTATCCGCTGCAGAGCACTCCCCATTAAAGAAATCGATTTAGCATTC[A/G]ACTTCTCCTGTGGCACATATTGAAGTGAGATCTGACAAGGATGGAAAACAGATTTGTGTTAAATACTGCCAATAAAGCCAGTAGCTCAAGGTTAGAGCAG
25	27710783	SCAFFOLD164843_274	0.38	0.28	CTCACCCATTGGCCTGGCCTTCTACAAGGCCCGCCCACTGGATGGAGTCTTTTTCCTGGTTGATGGAAGGGCGAGGTATCCTGGGAATTATAGTCCGCAA[A/G]CCCTCAGGCTGGCGTGGAGGAAATCGGGCTGGGAATAGCTTTTAGACACTGCGGATCTCATGGGTATCCGCTTCCCCTTTCCCCCAACTGCGAGGTGGGC
25	30549649	SCAFFOLD240305_16371	0.28	0.24	CTGGCAGTCCAGTGGTTAGGACTCAGCCCTTTCAATACTGTGGGTGTGGGTTCAATCCACGGTCCGGAAACCAAGATCCAGCAAGCTGGGCAGCACCGCC[A/G]AAAAAAAAAGCTGAAGGGCTAACACTTTATTGGGAGACAGAGTCCCAGACAAGCAAGAGAAAAGCGATGGGCAATGAGGCAGGGAAGGGGGTACAGATGC

25	30581870	SCAFFOLD130222_4417	0.03	0.06	CCTTGCATCCCAGGAGAAAATCTTACTTGATTATGGTGTATGAGACTTTTAGTATATTTTTGAACTTAGTTTACTGATATTTTATTGGGGCTTTTTGCAT[A/C]TGTGTTCATCAGGGATATGAAGTTGTAGTTTTCTTTTCCTGTAGTGTACTTGTCTGGTTTTGTTATCAGGGTTAATACTGGACTTGTAAAATGAGTTTGG
25	32705398	SCAFFOLD245044_41239	0.34	0.46	TATCATATTAGAGTGGTTAGTTATTGAGCATGAAGAAATAAATAGGGGAAAATTCACATATTGGCTAATCTCTGTTGCTCTGCTTGTTTTGCTAGTCATC[A/G]TTTGGTTGATTGAATAATGATGATGATAAAACTGAATGGGTAGAACAATTTGTGATTAAAAGAAAACCTTCCATTCTGCTTAATAGACTGCAGCTATTAG
25	32724242	SCAFFOLD130246_31612	0.37	0.19	ATCCAGGCGGCACTGGGTGTGGCTTTTTCCAAAACTCCCAGCGGCCACAACCACTTCTCCATGAGACACTGGTGGGGACCAATGAGGGCATTTCCCACCT[C/G]AGCCTATCAGGTAATGGGGGAAACTCTCAGAAATCAGGAAATCAGTAAAGCTCAGTAGCAGCTGGCCTGAGAAAGAGCTGAAGTGAAATGGGCATGGACT
25	32831383	BES9_Contig346_679	0.34	0.33	CTAGACTTAATTCAGAAGAAACACAAAGAAGGTGAAATGGAGTTCAACCTGAGGAAGCTGTTTAAGATTGAGGAGCTAAGACTTTACCAGCAGCAGCGAG[A/G]GGACCAGTGTGTTGTGGGCAGTATTTGCAGTCAGCTGGAGAGCGTCCAGTTGTCACAGTGGATGGCAGCAGGGACCATTAACAAAGGAGATTCAGGATGA
25	35314575	SCAFFOLD1888_1505	0.43	0.36	TCAGGAAGAGAGCTGATGTCTCAGGCTTCCTCCATCTGACTTCGGTGTCTTATTTCCCAGTGGATTCTGCACTGTCACCATGACTGCCCTCTCGCAGAAA[A/G]GGGAAGGGCCCTGGCATGGGAGACCTGGGGGCACGCCAGAATCAGGGAGGGGCTGGCGCTCACCAGTACAGCTTGGAGTGCTCCACCAGAGTGTAGGTGT
25	36050067	SCAFFOLD60809_8909	0.25	0.42	GCTGTCCTTCATCCCTCGACCCCCACTACATGCATGAATACCAATACCAAACCCTGATGCCCCCTTCCTGACTTTCCCTCCATCGGAGTTCAGCTGCTGG[A/G]GTAGGAGATTCCAGGGTCTTATCACAGGTCTCTCAGCTCTTCAGATGTTAATAGCATCACACCCTGTCAGCTGGGAGCTGGGTAGATGTGTATCCCACCT
25	36733123	SCAFFOLD216780_1194	0.49	0.39	AGTTGACTTTGGGGCCGTAGTCCTGGAGGATGAGGATGAAAAGGAAGTGGGGGATTGGCTGAGTTTACGGAAATGGCATCACACGCCAGAGCGGAAGAAT[C/G]CAATCACAGAGACCAAGACAATCCACGATGCCTGACTTCGTGGATCTGACATTTTGGCAGAAGGAGACAGACACCAGAGAGAGAAATACGAACAGCACAC
25	37957831	SCAFFOLD154571_307	0.26	0.34	CGTGCCCCAGCCCATTATGTACCCAGAGGTGACCATCAGGGCACCCAGGGTGCTTCCTGCCATTGGCACTTGCTGCAAGAGTTTAGGGGTGCTTCTCAGG[A/G]TCCCATCAGGAGGGCTTCAAGGAGGAGGCAGCATTTCAACAGGATCTCAAAGGAGGAGAAGGTGGCTAACTCAGGTGGTGCCTGGGAGGCCGAGCAGGGG
25	39197170	SCAFFOLD10375_2467	0.41	0.27	CCAGGCTCCTCCGTCCATGGGATTTTCCAGGCAAGAGTGCTGGAGTGGGGTGCCATCGCCTTCTCCGTACTCACTGGGTGGACCCTAAGTGCAATGGCAG[A/G]TGTCCTGATAAGAGACAGAAGACAGACTCACGGGGGAAGAAAGCCATGTGACAACAGAGGCAGAAATTGGGGTGATGCTTCTACAAGCTGGGGAAGGAAC
25	39403764	SCAFFOLD206036_438	0.43	0.32	CCCGCTGGGCTCCAGCCACATCCGCCTGACTTTGCCCCATCGCACCAGCCCTTGGCGTGTGCTGTGCCCACTGCCTGGAATCCAACGCTCTGATCTTTGC[A/G]CAGCTGGTCACGCTCATCATTTTGGTCTCCATGCAGATGCCCCTCCTCTGGAAGGCCTCCCTTGACTTCCTGGGTTAAAATAATGCTCCGCGACCTGCAG
25	39809856	SCAFFOLD216862_6835	0.40	0.47	GACAAATTTTCCCAAGAAAATATGGAGTCTCGGGAAAAGTTACCTGAAACTGACCATCGTCTCTCAGGCACAAAGTGCCATTTAAACCAAGAAGAAAGCA[A/G]CTCCACATCTGGAGTCTTGCCGCAGCCCACAAAGCTCTCATCCCCAAATGCAAAGCGTTTTACAAAAGAAGGGATTCCTTTAAATCCCAGTGTCCTTGCA

25	40234964	SCAFFOLD100231_10874	0.48	0.42	GATGAGTTCAGTTACAGCATCAGTCACTCTTCCACCAGGCTGCGGACACGCTTATTCACTCGTGGGTTGCTGGTTTTTTTTCCAGTGAGGATATACTGTT[A/G]TCATCCAAGCAGAGTTTATAATGTACATGAGACTGGAGCACACACACTAGACCACATCCATCTCACACTCACACACACACTCACACACACACTCACACAC
25	40235065	SCAFFOLD100231_10975	0.47	0.42	CAGCCCAGGTGAATCAGCCATGTGACCGCCTTGGTTTATGTCCAGTTACCACGCAGCACAACAGTTTTCCAGCCTAACAGGCATTCGGAGCTGTGCTTGA[A/G]GATGAGTTCAGTTACAGCATCAGTCACTCTTCCACCAGGCTGCGGACACGCTTATTCACTCGTGGGTTGCTGGTTTTTTTTCCAGTGAGGATATACTGTT
25	40235114	SCAFFOLD100231_11024	0.15	0.26	GCGTGTCCGCAGCCTGGTGGAAGAGTGACTGATGCTGTAACTGAACTCATCCTCAAGCACAGCTCCGAATGCCTGTTAGGCTGGAAAACTGTTGTGCTGC[A/G]TGGTAACTGGACATAAACCAAGGCGGTCACATGGCTGATTCACCTGGGCTGCTCTTTTGTCCTCTTGATTAACGACCTGGTGTTTCTCTACGTGGTAAAA
25	41675215	SCAFFOLD131294_7825	0.12	0.10	GCCACGACTCCAGCATTATAGCCAACACCGGCTGGATGGACTTCCAGTGTGAGTGTCACGAAGCCAGAATTAAATGGCATGTTATACTTATGTCCCAAGC[A/G]TCATACAATATTGATCTTTTTATTGTAACTATAATTTTCTGAGGGCTGTGAGTAAATAGATTTTTTTTTGTCACAATTATAGTAATCCACTTGAAGCTT
25	42471441	SCAFFOLD295392_4784	0.33	0.27	CTCCTGCATTGGCAAGTGGGTTCTTGACCACTGTGCCCCCTGGCAAGCCCTGAGGCTCCGCCCCCCCACATAAACCCACAAACTGAAATGGTCTGGGAAA[A/G]AATAGTCTCTTCAGAGCGTGGGTGGAAATTGCCGTTATGAGTTGAGAGTGTACCCCGGAAGGAAACGTACGCTGCAGAGCTGAGTGTTACAAGCCGCGTT
25	43343119	BES9_Contig459_684	0.36	0.27	CTGTCCTCCATTCTCCAAAGAACCTACACTCCTAAGCTTGGAAGTAGCCTTTCTGAACCCTGGTGCACGGACGAGGCAGGGGCTGTGCTCTCTGGGGGAC[A/G]TATAGAAGCTGTCTTTACCTTCAATCGGGACCACGGATGGCTCTTTAATGGAGGGAGAGATGGCACGCTTCTCTGCAACCGCAGCCTCAGCCAGGCGTCC
25	43638141	SCAFFOLD300541_6351	0.20	0.17	GTCAGCTAGGGGACCAGATCGCCTCTCCCTCTGTCACAGCCGCTTGTAGAACCCTGGGGCCCCCACCCTCCCCTTGGCCCTGTTGCCATTACAGTAACCT[A/G]CTTCTCACCCCAGCGCCGTCCTTGTGGCAGTGGGTCGCCAGCTGTGGAGGACCGGCGGCACAGGTTTCTGTTCTGCGCCCTCAGAGCAGCCTTTCAAGGT
25	43751222	SCAFFOLD52001_2575	0.12	0.11	GGACCTGCGCCTCCAGGGCCAGGCCTGCAGGCTGGGCCCACCTTCCAGAAGAAAGCCTGGGCCGGGGACGTGCTGAAGGGAGGTGGGCACCTCATCGGCC[A/G]AGTCAGGAAAACAAGCTCCAGATGGAGACCCAGCTGGGCTGCTCCGGGGACTCGGGGGGCAGGGGGCCCTGAGGGGCGGCTGCAGGACCCGAGGGCCCCA
26	837210	SCAFFOLD246909_16859	0.47	0.41	GCTGCAAGGTCGTCAAAAGACCATACATTCTGGCAGCTGGCTGGGGTCAAGTACTACATTTTGCCTTCACACCAACACCAAGGACTGACCCTGCAGCCAC[A/C]GCAAACAGCAGGTATCTTTCCTTTAGAAATGTTCCTGTAGCACCCTCTACTGGAAATGTTTTAAAACGGCACTCACTGTAAAAGAGAAATGCATTAAGAA
26	2896324	SCAFFOLD105131_5601	0.50	0.00	CATTTTCCTAAACCAAACGGACAATTTCAAAATTTTTGAAAGGAGTTTTTGCTGAAAGGAATTCTGACAAGTAGGATGGTTAAGAATTATTAATATATCC[A/G]AAGCAAGTAGGGAATTTTGAAGACAGCATAATCAATGACAAGAATTCAAACCATGGCTCAGAAAAGATGAAATGATTCAGGATTGAAACATTAGTGCTTT
26	2896452	SCAFFOLD105131_5729	0.40	0.49	CTTAATGGATTTGGTACTAAAATTCTTACCTAACTCATTATATATCATTTGTGATCTCCTGTTCTTACTCATTATTGTGCTTAATCTTATAAATCATCCT[A/C]CTAATTTGGGGCTAACCTAGTGCCTTAAAAGCACTAATGTTTCAATCCTGAATCATTTCATCTTTTCTGAGCCATGGTTTGAATTCTTGTCATTGATTAT

26	7484705	BES8_Contig245_678	0.41	0.40	TAATGTTAAGTGACATTGGTATTGCTTTTGTTCTTCCAAATTTGCTTCACTCTATCTGATACATAATTTAATCTAATTTCTCATTGTGCTTGTTTATTCA[A/G]CTTGAACACTTCTGTGCATTGGGTAGTATATTCATACGTCTAACTTCATATTTTGGCTGTTTACTTCATTTGTATGTCTTCAGAGCAAATGTGTGGGCAC
26	8434009	SCAFFOLD109398_2406	0.25	0.31	ATTCCTGTGTTGAAGCTCCACCCTCCCCATCTCCAAGCATGTGAGTGTGTTTCGAGACAGGGTTCATAAGAATGTAATTAAGTTTAAACGGGGTTGTGAA[A/G]GTGGGGCTCTGATGTGATAGAATTTGTTGCCTTGTGGAAAGAGATGCAAGAAAACTCTCTACTCTCTCTCCGTCTGCCATGTGAGCAAACAGAGAGAAGG
26	10258006	BES10_Contig542_925	0.48	0.41	CTTAGCCCTGTGGAATTCCTTTAACAGCCTGGGGATTTCTAGGTTTCTAAGAAGCTTGAGGTGGTTGTTAACCTAATACACAGTCGGACGGAGCCAAGAC[A/G]GCCTCCCCATGCCAGCTGTGTGGTAGGATGTCTTCTTAAAAGGCTCTTTAAAATCAAGCGTGTAAGGGAATCCTTCATTCCTGAACTAGGGCCTCTGAAA
26	10258450	BES10_Contig542_1369	0.47	0.41	AACTCTTTTTTTCAAGTCCCTTTCAAAAGTTCCTTTGTCCTGCGGTGAATCCTGCCACTTGGATGGTTATGGCAGGAGACTGCTCCTGGCAAAGGGTTCT[A/G]GTATGTTCTCCCTCTGATCTGGGTTTTGTCCCTGGGTAATGCCATCTGTAATCTGGTATCCCAAAGGTTTAAGCGCCAGGCAGTTGTGAAATAATTACCA
26	10403830	SCAFFOLD266100_4197	0.15	0.24	AATTCATTCTTAATCTTTGACCATCTAATTCTGTAGGTACATGAAACCAACAGTATTTTAGCTGAATAGCTGGCTGTTCTTTACTCAAAGTGGATGTAGA[A/G]ATAGCGCTGAAAGATCCATTTTCCATATATAATTCTCCTAGCAGGCCAGTAGAGTCTAAACTTCTACCAATCATGGGTCATTCCTTTTGTGTAAAAGCAT
26	11500215	SCAFFOLD5102_12208	0.18	0.19	AAAAGAAAAATAAAATACAAATGTATGCCTGTATAAAATCAGAATATCCCAAATATTTTATACAAATTTTTAAAAGCTCTGATCTCTATGCTCATTTTTC[A/C]TGAATATTTATAATACTTTAGAGAAATTACATTGCAGAACAAGATAAACATAATTTTACTATGACCATCATAGGAAAAAACATGTATAAGAATATTTATA
26	11500747	SCAFFOLD5102_12740	0.49	0.46	CATGCTGAATATACTTAGTCAACCTGGGTGCCCCTCATGGCTGTGTTTTTGCTTTCAGTACCTGTGTCCAGGCATTTGTGAGACCTCTCTGGAGACAGTG[A/G]ATTGAATGACCTTCAGGGTCAAAATATTTCCTTGAGGTTCAAGACTTAATTTTTTTTTTTTGGCCATGGGGCTCAGCACGTGGGATCTTAGTTCCCCAAC
26	12204007	SCAFFOLD110120_25106	0.39	0.49	AGAATGTAACTGCAAAAGAATATATAGAAAAAAATACGTCCCTCCTCTAGGATCCCAAAGCCCTCTCTGGGTGTCCTTATAGTGTATCATATTATCTTTT[A/T]TCACTACCAAACTGTGAGCCCTTCAAGAAAATTCCTCTTCTGTTCCAAACACCCCCCAGGAACCCAACGTATTGTCTGACACTTAATTTACACCTAACAT
26	13512895	BES10_Contig658_1482	0.27	0.28	ATAGGGCAGGAGAAAAAACATTTATAATCAAAGTAGTATCTACTCAAATTAGCTTTAGAAATTACAATGAGATTGGGAAAAGAAGTAAGTAGATCAACAT[A/T]ACCCCACCAACACTCTAGAAAATAATTTAAAGCCACCATCAGCATCACTTTCATATGCAAAATAAGGAAAAAAAAAAAGACCGGCACTAGAAGTATTCCA
26	13610649	SCAFFOLD90532_852	0.42	0.46	GACAGAGAGGAATAGAGACGGTGTTCCAGAAGGGCACCAAGAAAAAAGGCAGGAAGTGGCAATAACCACGCGGTGGTGGGAAAGCAGAGAGAGAAGTGTG[C/G]CGGCCATACGGCTGGGGTCTGCCATCAGATGAGGTGCAGTGAATGGAGGCTGAAGAAAGCCATCCTTTTCTATCTCTAGGTAAGAGGGAGATAGAGTGAA
26	14595084	SCAFFOLD1162_6502	0.48	0.04	CCATTTCCTCATCTGCAAATCAAGGGAATATAATAATCCTAGCTGCAAGGATTAAATGATCCAGTGGTGAGAAAGTACCTTGCACATGGCCATAATAAAT[C/G]ACCGATAGGCAGTAGCAACTCTCGTTAGCCATCAAGACATGAAGTCTTAGTGCCAGCTACTTTCAAACCCTCAGCTCTTAAGCCACGCCTTTAGTCCTTG

26	14595383	SCAFFOLD1162_6203	0.15	0.22	TGCATGGTACCTCTCAGATCTCAATTAGCCACCAAAGTCGAGTAGCTAGAAAAGGAGTGTGATTGGAGGGCATGCCATAGTTTCCCTTGAGAGGCTCAAG[A/G]CTTCCTGGGGAGACCTTTGTTTTTCTCCTTCAGTGGACATGTGTAGAGGGGCAGCTTCTAAGAAGGGATATGGCCCCTTTTCAAGTGGCTTTGGAGTGGC
26	15807244	BES1_Contig548_1195	0.32	0.28	TCCTCCAAATGAGTCACTTCACTATTAGAAACCAAGGAAAGTCTAGGTCCGTTAGGCAGGAAAGTTATCTGAGTGTGATAATCAATAAGCCCCCATTCCT[C/G]GAAAAGCCCTTCCCCAATTTTTGCAAAGAGATCAGGTCCAGATCAATGTCCTTTGCTCGCCCCCTCCATCCCTCTCTAAATGAGACTGGAGGGAGCCGT
26	15977147	BES10_Contig580_1086	0.22	0.33	CTCTCCAGCTAAAGCTTTAAGTAATTGAAAAGTTCTGAATTTCTGTTTGGAAAGCATGATGATACTCAGTAAGACCAGAACAATCCAAATAATCCTAAAC[A/G]TACAGAGACTAGATTCATAAAACCCCAGAGTAATTTTTTTTATCCCCATCTAGAAAAATCGTATTTCATAGCAGTTCTTACAATGCCCTGACTCTGGATG
26	17335235	SCAFFOLD155200_32871	0.22	0.31	AAAAAGGAGCAGGGTGGGTGGGAATGTAATATGATCTGTCTCTAACAAGGTTTCATGTGGCCGAGGGACTCAGAGCATCACAGAATTTCAGGGCTGGAAA[A/G]GACCACAATTTCCAAATGTTATGAGTCTTGAAGTCCAGACCAATCCATCAAGATGAGAGAATACTACAACCAGTCACACACGCTCCCTGAAACAAAGAAT
26	17599641	BES8_Contig453_1098	0.17	0.18	AAAAATAGTCAGAACCATAGAGTTGAGAAGTTAAAACACATTTGGAGAGATCAAATAAATCTCCCCTTTTCTTGAGGAAAAACACTGTGTACTAGAAATT[A/C]AAATCAATCTTATCCATTCATTTATTCAACTGACATATGGAATACTGACACAGACCAGATCCTTTGCTGATTTCTGGAGATCTAAAAACGTGTAGTGTTC
26	17599784	BES8_Contig453_955	0.18	0.17	TGGAGTTGAGGATAATTAGTGTACATGTCACCACTTCCAGGAAGTCTTCCTGAAAGTCTCTTAACCCTTCTCTAATGACAGATTAAATACCCTCCTCGGC[A/G]TCTGTCTGTGTCCGTCTCCTCTATGAGACTAACTCACTTTGAGAACACTACACGTTTTTAGATCTCCAGAAATCAGCAAAGGATCTGGTCTGTGTCAGTA
26	19537212	SCAFFOLD134029_802	0.07	0.13	TCTTTGCCCCCCTCCCAGTCTGTTAGGCTAACCCCAAATTTACAAAATTACCCACACAGAACAGGAAATGCAAATGTCAAAGTGCTCATAATACTTGACC[A/G]CATTTTTCATGTGATTCACATTAAAGCGTCTTATGTAAAAGTTGAACAAGCATTGAAAATCTATGTTAATTACACACAATATAAATGAATTTTTTCAAAT
26	20122895	BES11_Contig361_1110	0.01	0.06	AGAGCAAATAGACTTTTGCCCCGTGGGTACCCGTGATCCCCAGTCCTCTCCACAAGGAGTGTTGGGAACTCTGTGCCCTGCTGCCTCATGCCACCACCCT[A/G]TTTTGAGGGCATCTGAGTCACGTGGTGATGGCCCCGGCTCCACACTTGTCTCTGCATCTCGGTCCCTGCCACCCTTTCAGTGTAGAAATAAGTTGCAGGT
26	21118484	SCAFFOLD240765_1255	0.34	0.19	GATGTGAAGGGTTACTCTGCAGAGGGAAAAACACAGGTGAAAACGGAGTCATGGTTGTGACTGCAGGGAAACCCTTTGCTTGGTCGCTGCTTCCAGTCCT[A/G]AAACTCCCATCTTTGATGCTTTACAAATAAGTAAACGAGGTCTGGATTATGGAATCTTGTGATTTGAATGGAGTTCTGGAACACTGTCTCCATCGGCTGC
26	22129599	BES11_Contig457_1355	0.19	0.24	GCTAGACAAGAGGAGGATGTCACCTGTCTGTGGACAGCTGCTCATGACCCATCATGAACTGCAGATTGTCGATGACCACATGACAAATGTCGTAAACATA[A/G]ACTGCATGTTGCATTGTGTCTATTACAGTCCTGGGAAGAGGAAGAGACAATGAATTCAAGAGTCAGTTACAAGTAATCTCTAGACCACGGCAGTCTGAAG
26	22206226	BES1_Contig564_1210	0.33	0.26	TCAGGGAGGCCCCAAGCCCTTCAAAGTTAATTAAAACAGGAACTAAAATCACAGGATTGCTTACTAACCTTTTCCCCATCTTCGCCTTAAGGGGAAGACA[A/G]CCCACAGGAGCCCTGCCTCTGGCCTGGACCCTGTTCCCAGGCTGTTTCTTCCGGAACCGACCCTCATCCTGGACAGGCACCCTGGCTTTCCTGCAGGCAC

26	25330025	SCAFFOLD265399_2401	0.21	0.34	AGATGCACTGGTAATTTCACATCAGCGCTTGGCATCCAGCACTGTGTGGGCAGAACGTCCCAGGGGTGATCATCAGGTCCCCTCGCCAGGCCACCCCAAC[A/G]CTGAGCTATTTGGGGAATTGCTTCCTTTTTTTGTCACCGATGAGCTTCACTGGGCTTTAATGTGTTCAAAGTTCAGGTGGCCCTGGAGCCCCCGGGCGGC
26	26085394	SCAFFOLD290023_4337	0.29	0.25	GGCTCTGGAAACAGGGGCCACTGCAAGCCCTGTTTGGTCCTGTTGGGTCTTCCTATTTGGAGGAGGGAAGTAGTGGGCCATCTGGGGAAAATGGTGAATT[C/G]TGGGGGTGGGATTGGAATTCACACACCAGGTTGAGCAAGGGCATATGATATTTTGGAACTGCTGTGCACCCTGAATCCTGATCTACTCATTGAAATCACC
26	26085495	SCAFFOLD290023_4236	0.34	0.38	TGGGGGTGGGATTGGAATTCACACACCAGGTTGAGCAAGGGCATATGATATTTTGGAACTGCTGTGCACCCTGAATCCTGATCTACTCATTGAAATCACC[A/G]AGCAGCCTGGAAGAGAGGCAGCAGTGATGACCTGGGGTTTTAGTGCTACCCGTGGCAAATGAAGCCCAGAAAACCAGCACACCCTCTAACGGTTTGATCA
26	26085713	SCAFFOLD290023_4018	0.37	0.38	AACAGTGGGAGCATTTCTCAGGCCATCCTGAAGTCAGGGCCCAGGAGCCCTGGGAACAGGACACCAAAGAAAGCGGAGGGTGAGGGACTCTAGCAAGGCA[A/G]ATATTTAGTGCTGGTCAACACAGGCAGAATTCTTTTGTCTTCAGAACCATTGAGTCCTTATAATTCGTACCAATTTCTTCTCTTTTTGTTCTTGACTAGG
26	26203049	SCAFFOLD295529_6923	0.22	0.24	NNNNNNNNNNNNNNNNNNNATTTTCTAATAACTTTACCTTCCAAGAAGATTCTAAGTATGTACAGAACCATGTATGAAAGCCAATAAGCCAGAAAGGAAT[A/G]TCCCTTCCTTAAGTGTACCCCAGAACCATGACAGTTCACATTGGACCTGGAGACATACTACAAAAGACACACTGACATGCACTCTATACAGGTAAACTAG
26	30057135	SCAFFOLD90026_17834	0.25	0.32	CCCTTAGAGGAATCTCATCAGGATTCCAAGATTCTAGTGACACAGAGTTTGGAAAAGGCTCCCATGTATTAGGGATTGCAACCCCTTTTCCTTCTATAAG[A/G]AGGCCTAAATTTTACTTACCTTTAATTCTGATCATTCTGAGTCCAATGTCAGTATTCTAGCATTAGGAATAGTTATTGACTGTACATGTGAAAACTCAGC
26	30406680	SCAFFOLD245260_13971	0.23	0.30	TCACAGAGAAACCGATTAAATTTGACTTCCTGCTACAAACATCTGATGCTACTATCCGAGGCCCTGAGATGGAGGCTCTGAAAGTATCGCAGACTTCCCT[C/G]GCTGGTGTTTTCCATGAAGGACAGCAACTTGCCTATGATGATCCCATCTGCACTAAAGATAGGGTGCAGACAATGTGGGCAGTCCCATTCTTCTTCAAGG
26	30406944	SCAFFOLD245260_13707	0.23	0.30	TGCCTCTCCTCAAAGACAAGTTAGTGAAAAAATTCAACAGAGGAGTGTTTAGCAGGTGGAAGCTCATAAATCTGTCAGCAAACTTTACTGACCATATACA[A/G]GAAATTAGGTTATGCTTTTTTTTCCCTTACCTCCAGTGTCATTTTGAATTATGTACTCTGCATTATGGAGAGACCAGCTTCTTCCTGGCTGCTAGCCAGG
26	31356545	BES3_Contig288_1358	0.18	0.17	AAAGGATGGAGAGAAGCAAACCACTCAGCCCTGGACATGTGCTTCATCCAGAATAAGAATCAGCCCGTGAGCTCTCCAGCAGGTTAACTACCTTGCCGTT[A/C]GGGGAGGTATATGGGGTTAATTACTAATGAGGCATGGGCTGGTGGCTGCACACAATATCCTTACTCTGACACTAAACTCTCTGGGTCTTTAAAACATATC
26	31432263	BES5_Contig208_579	0.29	0.31	AAATTTACTTTGATATTATTTCTAATCCTACATTTTCTAATCCAGTTAGAAAATGCTGCTTTTATATTCTGTAACTAAGATATCGGTGTTAAAGGTTTAT[C/G]CCTAGCTTAAGAAATAAATGGATGCTTTCTATAGATGTATGCAAACAGGCAGTTTTAAATTTTAGGTAAGATTAAAGATCATTGCTTTTTAAATTTTTAG
26	33686222	BES11_Contig363_1256	0.11	0.12	ATGTAATACTTCTAGAATCTCCAGAGTAATTTCAGCATTAGTTACAAATAAAAGTTGGCTCTCTGCATCTCTTAACCTTGGGAACATGAACCTTTAAGAC[A/G]AGGAGATCAAATACCAACATTCCCCTAGGACTGCTGTGCACATCTGCATTTCTGGACATAGGACTTTTTTTGTTCCTAATCAAGTGTAATCTCACTTCAT

26	33782997	BES11_Contig288_1189	0.43	0.48	TTATAGGAAGACTCAAAGCACAGCACAGGGTGAAAAATAAGCCTATAGCAGTCAAAACTGTTGTCTGAAATGTCTTCAGACTGGAAGGCTGAATTTATTG[A/C]ACAAAACAAGTACTAGCGAGGCGGAGGTGTGCAGTCCTGCTCATTATCACTGGAAAGGAGAACCAAAAAGCCGAGTAAAAAATTAAATTTTAATAATGCC
26	34478468	SCAFFOLD271555_1329	0.47	0.48	GAGGCCTGCGGTTATTACACAAAGAGCACAATTGTTCACTTTTTGCTTGAGATAAAAGGAAGAGTACATGTCAGGGAAATGAATCCAGATTCTGTTTATA[A/G]AGGTGAACAACAGACTCACCAGGTCACTTCCTGTTGACACAGGAAGAGAAGCTTCTGGTTTGTTTTCTGGGAATCTCTCCACAAAGTCAGAAGGTCGCTA
26	34481827	SCAFFOLD271555_4466	0.26	0.34	TCATGTTGGTTGCTGTTAAGTGGCCAGTTCCATGAAGCCATGCAATTGACTTGAGTCATCTTGGAGCAGTCTCCATGAACAACTTCCAACTAAGGGAATC[A/G]GAGCCAAAAAATCCACTAAGGCAGCATGTTTTGTCTGATATGAACCCAGAGATCCCTTTGGGAAGCCACTGAAAGCTACCAACTCTTTCTCTGTACATGC
26	34481960	SCAFFOLD271555_4599	0.04	0.07	TTGCGAAGCTTCTCTCCAAATTCGATCCACCTAAGTAAAGCTGTTGCCAGCAAGATTCTCCTCTAGGGTCAAAGTAGGGGGATTCTTACCCTATTGTCCC[A/C]CAAAGAAGCTGGAGAACTGGGTATGCGTGTCAGCATGTACAGAGAAAGAGTTGGTAGCTTTCAGTGGCTTCCCAAAGGGATCTCTGGGTTCATATCAGAC
26	34760234	BV105383-115-R	0.37	0.34	TTCCAGATTCTGTTGAAAAAGCAACAGATGGACAMCACCAGTCAAAACGAAACAGAAATAAAAAAAGGACTGTAGAAACTAAAAAGAAGGTCAAGACCCC[A/G]GAAACGTGCCTTAGAGAAACAGACAGTCAGACATCTGTAGCTTCCAGTCAGAGCTCCRTATGTGGAGATGGTGTTCAACAGTGCCAAGACACGGAKACCA
26	36806927	SCAFFOLD106742_1008	0.34	0.32	TTTCTTTACATGCGGTGCAAGGCCATGATTGGTGGGATATGTTAAGCAACATTTGGGGGTTAAACTGAAAAGGAAGTCTCCTGTTAGACAGAACCATGAA[A/G]ACCATATGGGACAGTACTCACCATTTTTCCTCCTAGATATAGAAGCTCAGGAGTCTTCTCATCTTAGCATTTCACACTCATTTGCACGTCGTCGTGGTCG
26	37632424	SCAFFOLD15483_5985	0.39	0.25	ATAGAATAGATGGAGATGCATTTTCCTCATCTAGAAAATGGGACTAATAATGTAACCCTTCCTTACCCATTTGTCATGAATGAGAAATATGATTGCCAAG[A/C]TAAGTGCAATATAAATAACTGTAAATGAAATGCTAAGTAATAAAAGTAGGAATGATTTGACTAATAAAAAATGTTTTTCCTAACTCAGTTTTTCATTACT
26	38247254	BES2_Contig152_914	0.16	0.11	AGACGAGGTTTCTCTATCAATGGAAATAAAACTGATTAATGCAGCCTATCAGGATAAGAAGCAAATGAAGCATGCAAAAACAACCTCGTACCCCCAGAAG[A/G]GGGAAGGAGGTGGGGGGAGCTAAGTTTCCTCCAAGACTGGTTACAACTTTTTCTTTACTGTTAAAATTGAGAGTTGAAGGTTTCTCAGAATGCTTGCCTT
26	38309322	BES9_Contig272_406	0.39	0.47	TCAGGGATGAGGCACCCACTGCTGACTCCTTAAAAAGTATTTGGATTTATCAACTCTGTGCATTTCATATTAGTGGTTTAATCTAATCTCAGGAAACCCT[A/G]TAAGATAGATACTATCAGCATTTTACATTCACAAGGATAAAGCTTTGAGAGGTTATGCAACTTGCCCAGGGCCATCTGGATAATAAATGAGCAGAGATTT
26	38309860	BES9_Contig272_944	0.39	0.47	AGCCTAATAAATAAATACACTAATTGGGCTTTGCAAAGATAGATCTTCCCCAACAGTACTTATTACCACATAATAAAAACCCATCACAAGCTTCAGCTTC[A/G]TGGATACTCTGAGAGTAAATAAGAGATTATTTGCAAATTCAAATGTGCTGAACAGGTTGGCACTCTTTCAGGTAGCAGTGAGTTTAGGCCAGATGCCAAG
26	38353877	SCAFFOLD301963_2538	0.39	0.26	CTCCTGTGTCTTCTGCGTTGCAAGTGTATTCTTTACCCACTGAGCCTTCAGGAAAGCCCCCCTCTGAATAGTAAGTGTAATAAATTTGATAAAGTGTAGA[A/G]CCGTGCCATATAGGCCCTTGCCATTGTGCTGTGCTCAGTCACTTCGGTTGCGTCTGACTCTTTGTGATCCCATGAGCTATAGCCTGTCAGGCTCCTCTGT

26	38545913	SCAFFOLD145009_3607	0.45	0.06	TTGCCAGCACACTGGAATGAGATGGGATGGATGCTCCCCTTCCCTCTCCCTGTGACATCCATCAGAAAGGGCTAGCTTTTGAGCACTGAGAGAGGGGGGG[A/G]AAATAAGCAGAAGTAATTTATTCGGGATCTTTGAGAGGATAATAGCCAATTATTTGTTTATAACACTGGTTAAATTTTCATTAGGCAGATTAATGGAATA
26	40150542	SCAFFOLD131266_3327	0.08	0.09	CTATTCATAGCAGGTTCTGTTCTTGTGAAACGTTATATACACACCCGTGGACCATGGAACACTGACAGATTGCTAACTTCTGAGGGCCCTGTTCTTACTG[A/G]GGAAGCAAAGGCCTTCTTTGGCACACAGGTGGAGACTAGCTCTTAGCTTAATTTAGCAATCCTCCAACATGAGCAAGCCCAGAGCCACCTGGAGGGCTCG
26	40947322	BES4_Contig360_877	0.29	0.42	AGTATGAGGATTTTACACACTGAGGTCCTGCCCAAATACCATATCACTAACTCTCTCTTGTATCTTTTAAAACTCTGTAACTTTTATTTCTATGCAGAAC[A/G]AGTCATTTCTTTTCCAGAATCCTATCTCCTATGGACCCATGCGAAAGTGATTATATTTTAATCAGAAATTCTGAAAGAATAATTGAAGTCATAATGGAAG
26	40947555	BES4_Contig360_1110	0.29	0.42	TCTCGATTGTATTTTGGCAACTTTCCTTTCTCAGCAGATGTAATTCAACCTGCTCAAAGAGCCATCATTCTGAGGCTTCTTCCAGCTCTGAGGTGTCTGG[A/G]GACAAAGATCTGCAAACCTGACATCTTTCTCGAACTTCAGAGGGAAGGTATTTTAGAAATCTGGAGGGCAGTTTCAGCCTCCCAGCAGTAGTAATTTCAG
26	41817433	BES2_Contig539_2285	0.40	0.45	CTTGAGACAACCCTGATACAGGCTTAAAAAAAAAAAATCACATCTCAAGTTACAATATGAAAGCCTAGTCTGACTTTTTACTCAATATTATGGTTGGTTT[A/C]GTTTCAGTATGTGTTCAGAGACTACTCATTCTCAGTATGTGCTTAAGGAAACATTGCACTCGACCTTAACTGAGAGGGTGGTCACACAAAAGTTATCCTG
26	43697698	SCAFFOLD170285_15108	0.02	0.03	GTTGGGTTTATTATGTGCTCAGGGAGCTCAGCTGTTCTCATCTGAAAAGGGCAGTGCTGGGCATTTTCTTGAGTTTCTTGGCAAAAGCTGTAGGCGGAAA[C/G]TTGAAGCTCATCTGGAATAGTTTCCACTTTGCCTCCTTCTCTTCCTTCCACAATCCAGGTTGAGCAGAAAAGGCTTCACAATTAGAGAGAAGTGCTCAAC
26	43697998	SCAFFOLD170285_15408	0.47	0.03	CTTACCAGATTCAAACACCAAATAAGCAGCAGAGATGCAAATCCTAACTCAAATCCATCGGACTCTCAAGCCAGTGCTCTTTCAGTTTCACTGGGAAGCC[A/G]AGGTTCCCAGAATCGTGTTGAGAATCTCTGCTTTAGATGCCACCAGACTGTCACCTTCTCTCTGCCCGGCTTGGAAACCGCATCTGGGTGTCTGAGGGCT
26	45494871	SCAFFOLD10_46467	0.39	0.39	TCAATAAAAAGCATCAGACGGGCATGTCAGAAAGAGCTGAGATCCTGCTGTAAGGACTTCGTCTTCACCCTCTTTTCTCAGCTGCTTAAATCGCTGTCAC[A/G]GCCTACCTGTAGGAATCTTAGACAAGGGGGTTTTCTCTGATATAATTATGCTGGTAGTAAAAATCTTGGCGCCTTTTCTCCACTTTCCTTCTTAGCCACT
26	46311650	SCAFFOLD105216_6935	0.19	0.06	GGTTTTCCCCACGTACCCTCTCCGCTTCTATCCCCACCCTCCTGCTCCAGCTCTGTGTCTTGCCTTTAGCTTCTGCCATCCTCTGACATCCACCCTCCAA[A/G]CATCCCTCTTTCTGAAACTGAGACTCCACCCGCTCAAGACAGAGCACAGTTTTTGCACATCCATTCCTCAATTCAAAATGCAAGTACACTTATACCACCC
26	46456796	BES1_Contig647_1466	0.28	0.43	ATTCTTGCTGGTTGGCCCAGCTGTAAACGTGAGACAAATGTCCTGTAAACGTTTCTCTTACCTCTGGTTCCCCACAGTCACATTCCTCTCCTTCTTCAAC[A/G]TATCCGTTCCCGCACTTCTGGCCCCCGAAGGACTGCTTCACTTCCGGCAGGTTGAACAGACACATGCCCATGCCCTTCTGCAGGCTGGCCTCCAGGTCCT
26	47322086	SCAFFOLD130770_5013	0.37	0.38	GGAGGATGTATTCTGCATTGTAGATGCAAGCGTGGCTGTGGATTTGAGTTCTGGCATCTGTATTCGGGAAACAGAGCTTGTAATGTAGAAATCTCCCCCC[A/G]GTATAAGAAGGCTATTCAGAAGCTGGAGCGTGGACCTGGCCATCCTGCGTGTGGGCTGTAGTAGATGGCTGATGGCATCAGTGTCTCAGCCCCTCCGGAG

26	48596900	BES2_Contig541_978	0.49	0.01	CCACTTGGGATGCCAGACAAGGTGACAAATGTGACTCATAGTAGCCACTGCAAACAAATCTAAAAAAAGGACACCCAGAAGCACCTCCCTGAACGCCAC[A/G]GCGAAAAATGCCTTACATTAGCCGCAAAAGCTGTGTGAGTCCTTGAGGGCGTAAGCTCTCCTTGGTGTATTCTGGGAGGAAAATTACGTGAGTCAGGGCT
26	48629195	SCAFFOLD316731_49215	0.27	0.31	GAACCTGAGCAGTCAGGTAAACACCCTTTTGGCCCGCTCCTCACCACCAATTCCAGTCCAGTCTGAGCTGGAGCTAAGTGACAGTTCAGGCTTCTTGCTG[A/G]CGTGGCCAGCTGACGAGGGTCCTCCCACCTCTACTGCACCCTCCAGCCACACCTCCCCAGGCCTACTTGCAAACAGGTGCCTCCCGCTCACCGTGGAGAC
26	50027686	SCAFFOLD105036_27060	0.09	0.12	CCAGAGTGATGTTCCAGACGCAGGCGTTCCGTGTGTCCCACCACCTGTGGAGCCTGTTTGGTTACACTAACCAGGCAAGTGAGCTCCTGAATTTACTGTC[A/G]TTGGCTGCTCCGCTTTTCTGGCTTCATTAAAAACAGGGTGAGACAGAAAAGGACGTGCTTCCTCCTGTCTCTCACAGCCTCCTCGGCACTCAGAGGGGTG
26	50057793	BES2_Contig346_959	0.44	0.44	TAAAACAGATCTGACAGGTAAACTATGGTCTTTCCGACAAAGGCGGTGGAGGCGGTGCGCCCTTGGATAATTATTTCACTAGGATTCAGTGAAGCCTCTG[A/G]AACTCCGGCTCCGCGTGGACAGGGTGCTTTTATTTCCTTTGTATATAAGGGCTAAATGTTTTGGGAAATCGCTGTGTTCTAACGAAGAGGTTGATGCAGG
26	50963631	SCAFFOLD15206_2572	0.38	0.09	GATCTCCTCTTCCTGTAAGGACACAGTAACACTGGAGAAGGGCCGTTCCATGACCATGTTTAACACCCATCTCCTCTTTAAACACCCTGTCTCCATGTAA[A/G]TCACGTCTGAGGTCCTGGGGTCAGGGCTCCAATCGGGGGACACAGCCCAACCCTCAACACCAGCCACGTGCCCCGTCTCCTGCTCCCCCAGCTCAGATCA
27	1051	SCAFFOLD65094_19683	0.47	0.41	CGTCTAGTCATGGGGGCCTCGGCCGGCCGGAGTGCCCGACAGCGAGCTGCCCCGAGGGACTTGAAGATGAGACTCATTCCGCCGCTGGGCTCCGTGGTGC[A/G]TAGACCGCTCAGCCTCCCAGAGGCAGGCTCCCTTGCATGTCTTAGATGTGTTCTCTTCTGGGAAGAGTCTTAGCAGATGGCAGAAGCGGGGCAGACAGTC
27	9333426	SCAFFOLD101424_2510	0.35	0.23	TGTGTATTTTGTTATGCATGTTAAATGGAAATTTCTATTACACGAAAGTAATGAAGCCACATAATAAAAGTTTTCCTCCCTCCTTTCTCTTTGTAGTGGA[C/G]GAAGTGGAATTCATAATTCATTTGATTGCCATTATATGAAGGAATGAGCTTATCCACAGAGATTAATTTTCTAGCTAAATGAAGCTTATTCGTTTAAATC
27	9333597	SCAFFOLD101424_2340	0.43	0.41	TCTAGCTAAATGAAGCTTATTCGTTTAAATCTAGAAAGAGGTTTTTTTAACTTGTATATAACATGTATAATAGTATTTGCTTACATTCTTCCATGGATCC[A/G]GGTAATGGTTTCATCTTAATAACTCAGGATCATTGCTACCAGCGTTCAGCATTACATGGAGTCATAGAGCAAGAGTGAACAGAGAACGGTTAAGGAAGCT
27	12355411	SCAFFOLD130356_3115	0.09	0.16	GAAGTTATGAGAACCAAATTTCATAAACATTTTTCTATTGATACTTCCTCAATGTCTGGTGATTCTTGGTTCCCGTTATACCTGAATTAGGCAGTATTAA[A/C]AAGTTCTGATCTTTATAGGCGATAAAAATCTTGTTATTAAACAGACTTTGAAAGAGCTAAATGTTATTTTCTAGAAATGCAAAAAATATCAAAATGAGGC
27	13614715	SCAFFOLD10151_22515	0.29	0.20	ACTATACTTTGATGATCACAATTGCTTTAAAAACTTGCTCATGTCCATAAAGTTTGTACACTACACATAGCATGCAATGTTCCCTAAAAAAATCGGTTAT[A/G]AGCAATAACATTAAACATAAAACATAAGGGATGTGACAATATCTGCTATGACTAGCCCTAAAGTAGTTTATAAACCAAAGTTGTAAAAATGATATGGAGC
27	15608079	SCAFFOLD155074_18588	0.18	0.24	GTTCTGAACGCAGTGCAGCAACTGACAACCAGTAAGTAATCTGTATTAATCACTTCCCCCACCCCAAAAGAGACCAGTCCTGTCAATATGGATTCAAAAG[A/G]CAGAAATAAACTGCCTTTAAACTGTTTTTTAATAGGTTAAGAAACAAGACACAGAGACAACTAAATATTCTATCAGGCTGCAGGCCCCAAAATACCACCC

27	17397398	SCAFFOLD105280_12735	0.35	0.42	CCAAAATAGATACGTCTTCAAGTATTCTCTTTCTAGTCTGAAATTCCATGATCCTGGGACTGGGTTGTTTTTATTAACAACCTTGATCTTTTTACAACAA[C/G]CTTGATCTAATCTTTGCCAAAGTTGCAACATCCAGCTATAAGCAAAGAAACACAAGAAACACAAAGATTAGAAAACCGGACTACAGTGTCCCTTTTTCTC
27	17397484	SCAFFOLD105280_12649	0.37	0.42	TCTTTTTACAACAACCTTGATCTAATCTTTGCCAAAGTTGCAACATCCAGCTATAAGCAAAGAAACACAAGAAACACAAAGATTAGAAAACCGGACTACA[A/G]TGTCCCTTTTTCTCTGGGCAACTTAAAAAATCAGTACATTTTCTCTCCCTTTAACTTTGGGAAAAGAAAAATAATACTGTATCATTGCATTTGGTTGCTT
27	17397652	SCAFFOLD105280_12481	0.35	0.42	ACTGTGGCTTCTCTAAGGTAACATATTAGAATGGCAACTCTGTTGACTGGATTATCAAAGCATACTTTCAAGAAAGAGTGGAATATTGAAGATGATCTGT[A/G]TATAGAAATTCCACTGGAATGGGAAAATGGTACTGCCCAACTTTTTTTTTCCAACTGAGATTTAGTGAAGCAACCAAATGCAATGATACAGTATTATTTT
27	17888381	SCAFFOLD190739_1625	0.44	0.46	CTGTAATAGGTAAGCTTTCTTTTGACCAAATTACATAGTTGATGAAGCCAAGGTATGTCTTTTTGTCTCAACTAATTGTTCAATTTGCAAGTGTTAGTTT[A/G]TCCTTAGGCGCTGGTAAGGAGTGTTTGAAGTGAGCGATGCAGATGATTCATTTAACTAACATCAGTAAGTTGCTGTTTTTTCCTCTTCTCATGTCTGAAA
27	18172661	SCAFFOLD252389_477	0.09	0.10	TGCAGAGCAGAGAGAATGTGCTGGAAGGTACCTGGACAACCCTGCTCACCTTTTCTCTTCTATAAAACAGATGTAAATTAAGCCACTGTCAGGGCTGTGC[A/G]GTGAACTCGGTCTGATCAAGGAAGAAGGCTTTTCCTTTCTGATAAACTACATGTGTACACACACACACACACACACACACCGAGCTTTCCTTTTTTCCAA
27	20180620	SCAFFOLD11246_10200	0.38	0.26	ACTCAGAGAGACCTCCTCTGACCACTCTATCCAAGCCCCAGTGTTTTTCTCTATCTCAGAGCTGTATAAGGGCTTTCCGGGTGGCTCAGACGGTGAGAAT[C/G]TACCTCCAGAGCAGGAGTTCTGGGTTCAATCCTGGGTCAGGAAGATCCCCTGGAGAAGGAAATGGCTACTCACTGCAGTATTTTTGCTTGGAGAATCCCA
27	24330273	SCAFFOLD303739_1450	0.36	0.44	TGAATTGATGCTATAATAATAGTACTTTCTCCTCTACTTTCTTCTTCATGAGAAATCAGCCCACATATCATGCAATTCACACTCACATATATCAGTTGAA[C/G]GAGTGTGAGAAATCAGAAATCATATAATTTACAGTGTTTAAATACATTACACAGCAGGTACTTCAATATCCTGGTTGTAGCCTTCTGGTAACTAGTTCAT
27	24813229	SCAFFOLD158097_907	0.38	0.49	ACAAAAAATGACATCTAATTTAATTATTGACATCTTAGCCCCAAATTATATGCACATATTTTTCATTGAAATGGAGAATGTAAATAAGCGCAATTAAATC[A/G]GCATGTTGTAATTCTTTCATCAGATTATGGCTTATAAAAGTTTGTTTCTTATTGAAAGTTAATCCACAAAATAATTCAGTAAGTGCCATCAGCTTATTTT
27	25239917	SCAFFOLD201083_3831	0.39	0.50	GGAGTTGCATCCACTCTAAACCAACCATTCTGAATCACACAATCTCAAATAAGGGTGCCTCTAGTAAGACTCCTAGGATTGTCTTCTTCCATAGCGGCGC[A/G]ACCATGTTAATGAACTTACTCTTTTCCACGATCATTTAGGACCAGTTCCAATTGTCTCTCAAATCCATGTCCCATGTTCCATTCCTACTGGCTCTCCTCT
27	25421465	SCAFFOLD150371_1216	0.42	0.43	ATCATCATTCCGGAAGGGCCTGTCCAACATAAATATATTACCATTTTCAAGAATGTTTACAGACTAGTTTCTCTGGGTTCTGGATCCACATTCAAGCTTG[A/C]AAGTCCCCTTCCTCAGCTGGAGGGAAAAGGTGGTATCCATGCTCAGGGGACCCTAGTAATGATCTCTGTTCCCACTTGCCAGCATTTGAGACTGTAGTTA
27	25421924	SCAFFOLD150371_757	0.20	0.28	AAATGATATAATCCTATAAAGTCTTAGGTACATAGTGAGTGCTCAATTATTTAATAATTATTTAAATTATTTCAAAAATAATTTTAATCATTTTTATTAC[A/G]CTTCATGGAATCTTTAAATGTGGTCACTGATGTCAAATGCCTAGTTCAGAGCATAGGACATACTAAGCTTTCAAGAGTTGGCTGTTACTGCTATTACTGT

27	26813974	SCAFFOLD102044_6244	0.30	0.42	CTAATAAATACTGTGACTTGTGAATGCTGAGTGCTGACAGAAGTTGGAAAATGTGCAATTAATAATAATAAAATGATATGGTAAATTGCGATGACGCATA[A/G]TGCTTTAAAAATTGATACTAAAATGATTTATTTGGTGGTGTGTGTGTGCATGCTCCGTTATGTCTAACTCTGTGACCCCATGGTCTATAGCCTACCAGGC
27	27201937	SCAFFOLD291887_1748	0.16	0.10	GCCCAGAGCTGAGAGGATGAGGGTCAATAACCAGGAGCCTCCTCTCCCAACCACGAACCACAGACCCCATTGTGACAACTCTTTCACCCACTGCAGCCCC[A/G]TGCCCCAGGTTGTGACAAAGCCGGAGGGGCCAGCAGATCTCTAACTGCCAATGTAGCCACTCAGCTTGAACAACTGTCACAAAGCAGGGATGAATGGATT
27	30833960	SCAFFOLD152064_3794	0.12	0.20	TTATCTCAGCTTTCTTTACTTCTGTAAAGGTTTTCTGAGCTCTGAGGACTAGTGGGTGCATTTATTAGCAGTGGGCAGTTTTAGAAGTAGGTGCAGTGGA[A/G]AAAGAGGTGGACAAGGGACGAACAGTAATGAAGGCTCACGAACCCTCAGCTGAATACAGAGACGGGAGAAGAAAGGAAGGAGCTCAGCGATGGGAAACGA
27	30834278	SCAFFOLD152064_4112	0.49	0.10	ACATTAGAGTACCAGTTCCCTGCTATAATGAGCTAAACGTGAAAAACAATTAATTCCAAACAAGACAAATACGCACAACTTTCTAAATGAAGCCCATGCT[A/G]TGTAGCTTATTTTCATGGTCACATTTAATAAAATAAATCAGAGCATAAATAAACAGACAAGCAAACATACGTCAGCCCTTATGTTCGAGTTTCAGCAAGA
27	31346748	SCAFFOLD11077_3047	0.04	0.09	GACTAGATAGAGAACGCTCTTTAAGGACTGTACCTTCCTTCATTCAGCATGTGCTTGAGTACCTCCTGTGTGTCAGGACTTTGAAATGTTAGTGTCTTAC[A/G]TAACTACTAAGTGGACATTAAATGCCGAATGAATATAGAGCACAAGAGTTAGCAGAAGATAGATGATTTTCCTTGTCCTCAATGAGATAAAATCATGGAA
27	32489608	SCAFFOLD430_17076	0.31	0.33	TAAGGCACAGATTTTAGGAGGGCAGCTTGTGGCTTAGAAATCAAATAATGGATTTCAAATTCTAAAATGTTGGAGGACACTGCCTTTCTCCTTCCCCATG[A/G]CAGGTAAAAAAAAATAGCCATCTTGGTAAGAACAAAATGGATGGACTCTATTTTGAATGGCTCAGTGGGTCAGAAGCTTCCAGGTGGATTTTAGCAATTT
27	33169944	SCAFFOLD127325_1517	0.05	0.08	CATACATCTCCGCCTACGACAGACCTGATTTCACGCTGACTGTCCTGGAATGATTATTAATAGTGCACCATTTAACCATCAATGCAGACTCGGTCTGAAT[A/G]ATACCTTTTAAGATTTTCCTAATCATGGGCACTATTGAGGCACTGTGAAGGCACTCTACAAACTAGCTTCATTTAATCTTTTCGTTTTCACATGAGTTTT
27	33170067	SCAFFOLD127325_1394	0.03	0.05	TGGTTAAATGGTGCACTATTAATAATCATTCCAGGACAGTCAGCGTGAAATCAGGTCTGTCGTAGGCGGAGATGTATGGTCACCTTGTATACAGGAAACC[A/G]TAAATGCCTTTACGCTGATGTATGCTATCAAGGATCTTTCTCTGTACATATACATATATATATGTATATGTGTGCATGGGCTTAGTTGCTTAGTTGAATC
27	33601432	SCAFFOLD125063_15484	0.26	0.29	CAGGGTCACGGTCCTCTCAACCAGGTAATAAAGAAGGGGCAGGGTGGGGTGCCTCTGTAGAGCCCCTTCCTTCCAGCCATCAGGAGAGTTCCACGAAGCC[A/G]TTTTACCTCTTGAGTAATGTTTGCAAGTATCTTGTAAAAGCTTTCTTTCCAAATATCCTAAAATTGGATGTGACAGAGGGCAAAACTCTGAATCTATATT
27	33601535	SCAFFOLD125063_15587	0.23	0.27	GAACGAAGCCACCTGAGATACCCTGAGAGGCATTTTGAGAAACTGGGTAGTTGAGAGAAATCGAAACAAATGTTACAACCACGAACTCCAGGCAAAAGTT[C/G]CACAGGGTCACGGTCCTCTCAACCAGGTAATAAAGAAGGGGCAGGGTGGGGTGCCTCTGTAGAGCCCCTTCCTTCCAGCCATCAGGAGAGTTCCACGAAG
27	36527406	SCAFFOLD271203_2920	0.19	0.22	CCCTGCCAGGGAGATGGTACCCTAGCAAATATCCCAGGCTTTGTGTTGGGACCCAGGGGCAAGGGTGAATCAGACCCTCGACTTATTTCCTACAGGTTTG[A/G]TTGTTTTCTTTCACTGTTTGCCTCCAATTTAAAATAGCATAATTGGTAAAGAATCCTGCAGTGCAGGAGACGCTGGTTTGATTCCTGGGTCATGAAGATC

27	37076812	SCAFFOLD111293_9353	0.49	0.49	CCCAAAGCACAACAGCCTGGGATCATTCAGGTCAGGAACCCTGGGAGTCATTACTGTAAAATAAACATTAATTAATGCATTACAATTTATCACTCTTTTT[A/T]AAGGAGGTAAGACAGGGTGTTTGGAGACTGTGGAAAGCATAATAAAAAGTTATGTTTCTGAAAGAAACAAACTCTTCTGTCACATGGAAAACGATTCTAA
27	37434175	SCAFFOLD120838_12714	0.38	0.43	TGCAATTAGGGTGATTTCCTATTTTCGGCCATTGCGTTTCTGTGTGTTGAGGTCTCACGTGAAGCTTGGGAAGAGGGGAGGGGCACACATATGGCTTATA[A/C]CCGCTTTATCCAGCAATGACCCTGGTCCCAGAGAACATGACTGATCTTGGCTTCTTCAGTTCTTTTCTGTGGCCAGTGAAGGGATAGGTTGTCCTAGGAG
27	37434252	SCAFFOLD120838_12637	0.31	0.38	CCCAGAAATTGCCAAGTGATGTGATCACCAGAATAATACTTAAGCAGTTGGCGAAGGGTGTGACCTTTAAGTGGCCACTCCTAGGACAACCTATCCCTTC[A/G]CTGGCCACAGAAAAGAACTGAAGAAGCCAAGATCAGTCATGTTCTCTGGGACCAGGGTCATTGCTGGATAAAGCGGTTATAAGCCATATGTGTGCCCCTC
27	39733164	SCAFFOLD10105_7056	0.48	0.44	TCACATTTTTATTGAACTTGCATTATACTCGGAGTAAAATTGGCAAATAAATATGTAAGAAGCAAGATGTCTATTTATACACATAGTTCCATTTTCAACC[A/G]TGGCTGAAGGGAAAGTATTTCCCTAATATAGCTCCTGGGACTTGGCCAAGCAGCCCAACACTTCAGGGCACAGAATAGGAACAAGTGAGCCTTAGGTTTG
27	40240121	SCAFFOLD295848_7080	0.50	0.46	CTAGATCGACTCAGATAGAGAATCAGCATCAAGGTAAGTGGGAATAGCTTGCAATTTATTTTTTTGTCCTTTTTCATTTTGTAAAATTTCCAGCTCAGAA[A/G]TCCCTCAGTGTTTTGATTATTCTCCTCTGGATATGTACCAGTTTGATAGTCTTTTTAAAAGAGGGTTCCTAGAAAACAAACACTACGCTTAAGAGAGAAG
27	41294262	SCAFFOLD220321_4637	0.49	0.46	TCCCTGCTTTGTTTCCAGGGCCTCACGCTATTTACTATTCTATGTGTTATAAGTTGAACCCTTCCTTCAACCACCAGAAATGGAAACTCCATGAAGATAC[A/G]GCCCTTGTCCTTTCTATATACTTCTATATTTTTGATGCCTAGCGCATGGCAGGTACTCAAAGTCATTTGTTGAAGGCAGGAATAAATAGATGAATAAGAG
27	41294408	SCAFFOLD220321_4783	0.47	0.43	AACTTATAACACATAGAATAGTAAATAGCGTGAGGCCCTGGAAACAAAGCAGGGAGGGACAGAATTGTGATCCAGGCACACACGCGGAGCGGGGCCGTCT[C/G]TGGAGGAGGGACAGGTGTGTGTGGGCAACATGTGTGCTTCACCCTGGAAGACGGAAGGAGGCCCATGTGACAGGACTGTCCGCTGACGTGTGATGCTGGG
27	41294441	SCAFFOLD220321_4816	0.49	0.44	TCTAGACACAGACATAGTCCATTCCACCTGGACCCCAGCATCACACGTCAGCGGACAGTCCTGTCACATGGGCCTCCTTCCGTCTTCCAGGGTGAAGCAC[A/G]CATGTTGCCCACACACACCTGTCCCTCCTCCACAGACGGCCCCGCTCCGCGTGTGTGCCTGGATCACAATTCTGTCCCTCCCTGCTTTGTTTCCAGGGCC
27	42235106	SCAFFOLD190156_7905	0.16	0.16	CTATGGGGTCGCATAGAGTCGGACACGACTGAAGCGACTTAGCAGCAGCAGCAGTATAAGAATAATAAGATAAAGAAGAGGAAAGGAACAGAGCTACATT[A/G]GAGCAAAGTTTCTGTGTTTTATGAAAATTGAGTTAGTCTTAATCTATAGGAGATTATGATAAATTAAGGTACATTTTATAACTCCTAGAAAAACCATTGA
27	42710278	SCAFFOLD147190_1021	0.04	0.12	TCTTTCATAATAATTCTGCAGTTCTTGGAGACTGGAGATGGGCATGGATGTCTGTAGAAGGCTACGCCATGTCGCTAACACCAGTCCATGTTCTTTCACC[A/G]TGCACAGTCTATTTCCTTCCTTCCCTAGTAAAAAGTTCAAATCTAATTTAAAGAGTAGGCTTTCTCCTAGAAACCCAGCTGAAGCCTGAACAAAAACTGA
27	43308627	SCAFFOLD120249_11968	0.46	0.05	CCCAATTCAGTTTCCATGAATAAAATAAGAGCAATAACTGCCCTATACTGATAACCTACTCTGTATTTGGAAGTCACTGAGCATTCTAGCATTTGGGTAT[A/C]CTTTCAACAATTATCCAGACAAAGACAGAATTTCACCTAAATTTCTGCCAAGATGTCTTGGCATACACCAGGTAGATGATAAGCATGGCGTTCAGCTGTT

27	43903046	SCAFFOLD281908_2988	0.24	0.26	AAGTATAATCAGGACACTTGAGAGTGAGTTCTACCCAGTACTCCCAATTAGCATATAATAACGTGCAGTGATTTACTGATTTCCTTTACTTAACTGCTAT[A/G]ATGGCTCAAAGAGCCAGCAGGTAACAGAGGAGACCATCTCTCCAACAGAAGAAGAAGAGACGGCTGGTCCACCCCCTCCTCAGAGCCCTTCATACAGGAA
27	43906621	SCAFFOLD120249_17244	0.34	0.29	GCCTCAGCCAGGCCTTTTGTCCCCTAAACAACAAACAAAGATCGTTAGGATCCATATTCAGGGTAGGCAGACTGCAGGCGGAATGAAGAATGACTTCACC[A/G]GCCTGCCCATCGTATTTGAGGGCAGAAGGTAATCCATAGGCTGCCCTCTCTGTGGACAGCTGGTGCAGTACAGGTGATCCATGGCCCCACACACCTGGTC
27	48275366	SCAFFOLD15536_13684	0.49	0.00	CCCAGCCCACTCCTCAGGGTAAGGTCAGCCTGTTAACAAAGCCCCGGCCAGCTGTGACTCCAGAACCGGCGTTTCTCGGCCAGCTCCCCAGGTGGGCAGC[A/G]TCCCCAGGCATCTCCAGGTGGGTAGTGCATTGGTTTTAGCTGCCTCCCTTGTCCTCCTTCCCTTTCGGGGCTGTCGGTCAAGGAGGCCTCTCTGCTGTGA
28	1653077	SCAFFOLD270101_29656	0.06	0.11	CCAGGATATTTGCCTCCATCCTTTTGCATTTCCAACACGCAGTGGATTCAGTAGGACCACCAGCAGGAATCCAGGCCTCCCCTGAGCTAGGAATGGCTAC[A/G]GCCTGCATTTCCCAGTTGGCCGTCAGAGGGCGACAGTCCCACCTCTCTTACCAGTGCAGGGGCTCCGGATTCTGCCCCCAGACTTGGCCCTGTGCACAGC
28	1653144	SCAFFOLD270101_29589	0.06	0.11	AATCCAGGCCTCCCCTGAGCTAGGAATGGCTACGGCCTGCATTTCCCAGTTGGCCGTCAGAGGGCGACAGTCCCACCTCTCTTACCAGTGCAGGGGCTCC[A/G]GATTCTGCCCCCAGACTTGGCCCTGTGCACAGCCTACAGGACACGGGCCTACTCAGGACCTACCCGGGGATTCACTTCAGGACAACACAGGGCTGTGTCC
28	2765207	SCAFFOLD20062_414	0.11	0.16	GGTCTCTAAAGCACAGTCTAGTTTGGGAAACAATATTCACAAGTGGACCAAAAGCAGAATAACCAAAAAGGAAGCAACTTGCCCAGCCTTGAGTCTGGCC[A/G]GTGAGACACAGGAAATGTGACACTGGCTACTGAAGCAGCACGTGGGGAAGTCAGAATTAAATTAATGGCTTCCAGGTCTCCATCCCGCCCTCTGCTCAGC
28	3553188	SCAFFOLD148171_1957	0.15	0.12	TCTGTGGTGGAAGGGAAAGCAACGCCCAGCGCAGTGTGGCAGGGCCTTCCCCGCTCCTCCCTGTTAGTCCTCCATGCTACCGCTATTTACAGTTTCTACC[A/G]TGATTATCCAGAGAACAGACCCGTGGGTCTATTCTCTATGTTGATGGAGATGGTGGACAAAACCCTCCAGGTGTGACTACATCTAATTTAATTATACTTG
28	4150241	SCAFFOLD282541_2701	0.47	0.50	ATGCCTGTTTCATGGACCAAGAGAAGTCCGCACATCAAATAATCTTAGAAACAGCCTGCCAGGCAGATGGTTTTAACACACGGTGCAGAATGTAGACTCA[A/G]TAATGCCAGAATAGCCACGTGATTCATATAAAACCCAGCACTACAGTCTATATTATCTTTCAAAGAGAAGCAATTGTGTTCAGCATCCAAATGTAGAAAA
28	5784816	SCAFFOLD81850_7662	0.31	0.44	CTTTCAAATAAATACTTAAGACTCTGGGGTATTCTTACTCATAACCTGAGTCACTGGTTATTAAAATGGATTCAGTCACGTATTAGAATGTATTTATAGA[A/C]TCCATGCTTACTATAAACACCACAGGAGGGGAAGGGACACAGAATATACCCAGGGCCTGCACTCAGGCCAGAGCTTCTGCTGGGGAGCTCAACTCAGGAA
28	6513403	SCAFFOLD82626_1783	0.28	0.34	CCGGGTTTGAAGAAAGAGACCATGTATCAGAACCAGAGTCAGCTGTGGCAGATGCTGGAATTATAGGACAGGGACTTTAGAGTAACTGGCAGGCATCAGC[A/G]CAATTTCTCTTAATACTTCAGCATACAAATATTGCCTTTTTAGTTTTCAAACTAAAAACCATATCACTTGCCCAGAAATCTGAAACTGAAAAAGCAACTG
28	7156245	SCAFFOLD225832_5380	0.36	0.30	GCCCCAAGAAAGGGCATCAAGGGAGGAAGGCAGCCCACCCACCGCCCTGTCTCCCTGGGAGAGGGAAGGACGGCTTACCTCACCCCATCTATCTGTAGGA[C/G]GACTGGGGAGCCGCTGTCCGGGCAGGGCAAGTGGTCAGGAGGAGGAGGCTACAGAGTGACCTGTGAGGATCAGGCGACCAAAGATTCTGAGACCCACTGA

28	8034260	SCAFFOLD322259_2911	0.35	0.36	TAAGATAGCTTCCGACTATACTATGCATTAATTCAGAATCCATTATCAAGCCGACGCTCTCAGTCAATCCTCATCAGCGCTAACCGTTGGCTAGCAGGCC[A/G]CCAAAGGCCTCTGACACTGTTTCCACTGCTTCCCCATCTATTTCCCATGAAGTGATGGGACAAGATGCCATGATCTTTGTTTTCTGAATGTTGAGCTTTA
28	8736461	SCAFFOLD170266_1965	0.12	0.20	TAGGGGAAGTGGAAAAGGATGTGCTAGGGGTCAGCTCGCCATCTGATTCCTCTGTCTCCCACAGTAACTTACATATGCATTCAGTACATCTTGACTATAA[C/G]ATTAAGGGGCAATAATAGGCTCATGTGGCATTTTATTACTTCAAGTATAAGGCCACTGACAAGAAGAGGAGACAGACTGGCACAAAGCCACAGAGAGAAC
28	10466344	BES8_Contig562_678	0.40	0.29	CTAAAGCTCACAGAATCCAGTGAAGGTGTCCATAGATGTCATGACTTCAGCAGCGATTGTTCTTGAGCACAAAGATGAAGAAGTGTGAAGCAGTTAATGC[A/G]CCTTGATGCAGACTCCATGGGGCTGGCTTTTGGGGCAACCACAAGCTTCCAAGAAATTTTCATTAAGCAGTCTGTGTGCACTCCCAGGAAGACGAGATGG
28	11137573	SCAFFOLD127004_3536	0.33	0.12	ATAACTGGGGGAAGAAAGACATGATCTTACTTCAGAATTTTGGACTTTAATTTCAAAAAGCATCTAAATGGGTAAAATCACTCCTGTTGGTGAATATAAG[C/G]CAATCCTTGAAACCTGGTGGTATTTTCTAGGTAAGTAGTGATACACCTTGTTATAGATTCTGTGAGGATGAGTGTGGGTTTGGGGGCTCCAAAAGTAATT
28	12120513	SCAFFOLD76808_7487	0.20	0.26	GAGCGCTGCCTTCCACTTGAAAGTGTGGATGATTAAAGCCGAGGCTGGCGGTGATGGAAGTGGCTCCATTAGCTCCGGGGGAAAGTGGCACATGGTTTCT[C/G]CCTTCTGCGCTTGCTCTAGGCCCTCCCCGGGGTCACACACACACACACACACAGGACCTTGTGTGATACAGCCGTTCTGTGAGGTCGGCAAGGCGGGGCT
28	13110587	SCAFFOLD26572_3283	0.16	0.15	GCCCCAAGCATCCAGTATCATGCATCGAACCTGGACTGACGACTCGTTTCATCAAGCACCTCATATCCAAGGCACATGAGACCAATTAACTCAACTCTTG[C/G]GACACAACCCAGACCTTAGCTTAAATTTTCCCAGGTGACTTCTATGTGTAGCCACAGTGAGAATCATAGGATAAAAACGTTTAGAATACATGAATGGGTT
28	13117400	SCAFFOLD313323_2560	0.22	0.30	AGCATTTTACTCTCTGGGTGCTGGAAGCACAGGTGTGGGAACTAGAGTAGAAGCCAAAAATCTAATGATTTTGAATTTCCACAGAACAGGGCTCTGGGCT[A/C]TAAAACCCTGAGCTGAAGACACAGTGGGAATAATGAGAAGCAGTTGCATTGTTTTCTATCATTTTTAGGTTTTAGAGGGGTGCAGGGAAAAGGGGGTCAC
28	13117448	SCAFFOLD313323_2608	0.23	0.30	AGAAGCCAAAAATCTAATGATTTTGAATTTCCACAGAACAGGGCTCTGGGCTCTAAAACCCTGAGCTGAAGACACAGTGGGAATAATGAGAAGCAGTTGC[A/G]TTGTTTTCTATCATTTTTAGGTTTTAGAGGGGTGCAGGGAAAAGGGGGTCACTGTAAATAATGTATTTTGTAAGTCACGAGTGTATTAGAAAAGACTTTT
28	14268867	SCAFFOLD35872_19407	0.44	0.44	TCCATCAACACTGCTTTTTTACAAAACCGGAGTTTGCAACTGCCCATAAACTAGTATCTCTAATCCATTTTAACCTTAAGAAGGCTTTGTTTTCACAGTT[A/T]CCATTAAAGAGTTAAGAAATTTGGTCTATCCAATAATGTCTGTGTAAGGAAAGTGGGTGGGCCCCGGAGTGTAATAGGAATAGATGAAAAAACCACCCAG
28	14733008	SCAFFOLD316873_553	0.38	0.29	CCCCTTCCACTGCCATTCTCTGTGGCCAAGTATGAAACCTGATCACTCAATTAGTTAACACTCTGGACACAGCATTGCGTCTAAAAATACCCGCCTTGGG[A/G]AATGTAAAGGTTTCCCCTGACTCCTAGTAAGGAGGCCCAAACTGGTCAGAATGTCCCTAAGTATTGAAACAAGGGGTGGGTGGAGGCAAGGATGGCCGAT
28	14988013	SCAFFOLD15286_17074	0.49	0.48	TTAGCACATATATGATTAGCCCTTTGTTAGAAACTGGAAACACAATGAAAATGAAACAGAGGGAATATAAGAAGGTGATGGACACAGAAATAAGGGAGAG[A/G]AAGGTGATCAGAGGGAGCTTCCCAGTTAAGCCACTTGGCTCTTCAGTATGTTTGGTAGTTCTTTCCCTGAACTACAGATGGTAATACAACTTGACGATAC

28	15385295	SCAFFOLD312111_5581	0.29	0.25	TCTGTTTGTGAATACCTCCTTTATTTTGTGCCATTAGGCACACTGAATTATTGCTGTTTATTCCTTGCTTTTCAGCCTTGCTAGGAGATATTCCAAACAT[C/G]GTAACACACGAGACATTTCTCTTAAAAAACAATATAAGAGATAAGGCTGAAGTTCTTTGCCTTTTTCCTTTATACATGAGCCAGCTATTCCATTGACAGA
28	15469221	SCAFFOLD192017_9820	0.23	0.21	AAAAAAAAATCAACAGCTTTTAGTATCATTAAATTGACCTCTGAATAGAATAAGCCCGAGCCTCCACCACACAACATCCCAAAACAGGTATGGAACTGTG[A/G]CCTTAAGATGCTCTGTGTGAGTGCTGTAACATTTTAATTTACAGAGAAGCAGTGATTGATGGCCTCTCAAAAGCCCCCCTGCTCAATACTTTCTATTTTA
28	16777366	BES10_Contig435_1219	0.03	0.01	AACTGTCCACACAGGCCAGCTTGAAAGGAGACTGGGGCAGATGACCATACGTGACCTGTCCTTAACAGGATGCCCTGAGAAGAGAGGCCTTGAGAACAAG[A/C]CACTGCCCTTAGGCTCTTTTCACTCATGCCGCAGCCTGGTCAAGTCTCCGTGAAGAATTTGATGGGAAAAACCCTCAAGGACAAAATACAAATTTCTTAC
28	17372092	SCAFFOLD215976_11111	0.44	0.41	GTGAAATTATTGAAGTCATTTGCTCCAATGGGTGTGACAAAACATACAAAGAGGATGGCCAGAAAGATCATGATTTGTTATTGCGTGAGGCAAATCAAAT[A/G]AGAGAATTGTGATACTGTGTACCTACTGTTGGTGCTGACTAGTGTGTGTGTGTATTTGTAGAGATGTTTGTGAGGACGATTACCAAATGTAAATGGTGAT
28	18024346	BES2_Contig400_1246	0.42	0.35	TTCCCTCTGTTCAATGGGAATGATGAGATGGTGAGTCACTTTTACCATTGAGACTATTAATTGTGAATAACTAAAAATCCCTTTGCAAGCAAGGGCTTCC[A/G]CGGGAGCCCAATTTTGCTAAAACAGTTGGGAAAAAATTGGTAGTGTACACATTTCAAGGCAGTAATAATAAACTGTAGATTTTGCTGTACAAGGAAATAC
28	18024848	BES2_Contig400_744	0.42	0.35	TTCGGACAGCATTTTCTTGTTGGCTAAAGTCCCACCAGTAATGAACTGGAATATAAAAACAAAGACTGCTCTCATGTCGTTTCCCAACCAAAGAGGGCTA[A/G]GCCCTGGGTGAATATTGTAGTGTCGGCATGTTTATCTGAGTCTTGAGCTTGACTTTTTCCTACCTAAATATTCCATCAAATCCCCACCAGGATGTGCTCA
28	18554359	SCAFFOLD171671_5714	0.31	0.29	AAAAATAAAGCTCCATACTGACAAATCTTACAAATGATCTTTTTAAGGCAGCATAGATGTGCATGCTGAGGCAAATGTGCAATTTAGTTAAGCTGACAAA[A/T]GTGATATTTTGCTGTTTGGTAGACTTCATTCAAATACTAACTGGAAATATGAAATGCATTTCCAAGTTGAATTTCTTGAAGCACGACAGACAATGCTGTA
28	19292152	SCAFFOLD11052_910	0.42	0.47	AAGACTATATCACCAATGTATGCATCACATTCTCAGTTAATGAGAATTAGGACTGCAACATATGAATTTGAGGAGAACATAACTCAGACCACTGGCTAAA[A/C]CACTGGTAGTCTTAGAACAATGTAACAAAATCCACCATCTCCACAAAATAAAATTCAAATGTCCACTTCACATACCAGAATTACTTGACATATGAAGATC
28	19392216	SCAFFOLD100563_498	0.14	0.16	ATAACATCTGTAGAACCTATACCTCCAAAGCTACACCTAGCTGTAATATGCCTCACAGAAATTACCCTTTTCTCAACAACACACTCCTACAAATCTGACT[A/G]CTGTATGTTTAACACAATGCCTCTATCAATTTAACTGAATTGTTTTCTTAGAAACGTTTGTACAAGGGTTTTATATTACATCTATGTTATAACTTTACAC
28	21868538	SCAFFOLD205696_20838	0.13	0.21	GGTGCCTGCAGAGACCATTTGCAAGAAAGTAGCATCAGCAACAGTCACCAAGAGAAGACAGAAGTCAGATTGCATAGCAGCCAGAAGGAATCAAAAACAG[A/G]CTCAGCTGACCCAAAGGCTACAAGGCCACAAATGTCAAGTTCTTCCCTCAGTGCCTGCTGCTGTCAACTACAAAACAGTCTTTCCTGGCAAGGACAGCAG
28	24327679	SCAFFOLD150989_2786	0.09	0.17	AGAGTTGGATACAACTGAGCTTTAACTTTCACCTGATTTCCTATCTTTTATTATGCAAATCTGGATATTAACATTGTTAGGTCTTAATTATGAAAAAAAC[A/G]TTTCAATTCTCACATACACTTTTATTCTGCAATAATGCATCTGCAGTTAGAATTCCTATATTGGTGTTTCTCCTTTAAGAAAATATACTTGTTCTTTTTT

28	24504312	SCAFFOLD151783_559	0.43	0.12	TGATACCTGCTCCCAAAACTTCCAGGGGTAAGTAAGCGAAAAGAACGAAACTTTCAGAAAGAGAATTTCAACAGGGAAACACAACTCAGAGTATTAAAAA[A/G]AAGTACCTGCAAACAAGCATGATACAATGCATTATTAAAGACATGCAAATGCCAACTTAAACCACAGGTATCCCCTGCACTAGTCAAACGAGTCATAAGA
28	24504481	SCAFFOLD151783_728	0.06	0.13	CTTACCCCTGGAAGTTTTGGGAGCAGGTATCATTTACAGCCCCACAGCGCTTCTGAGTGTTAAGCTGCAATAGCTGTTGTTTTCAGGTCGTTGTTGCGGT[A/G]AGTGGCCACGAACAGGATGTGCAGCTCTGGTTACCCAGTTTTGCCAACACAGCCTTACTGCAAGTCCTGCTTTGGGGTTGTAAATTAGGGTGGAATGAGC
28	26871628	SCAFFOLD176855_4155	0.26	0.30	GTTTCAAGGGGACATAGACTCCATCTTTCAACGGAAGGAATGTCAATGAATTTGCGCGCCTCTTAAAACCAAAGTGCAGGCTGGACAAGCGAGAGGGAGG[A/G]TGGTCTCAGGCAGCCTAGGGGCTGAATGCCAGCTGCCCCCTGCCCCGCCTGCCTCCTTGCACCTGCCCAGCACACAGGGCTATGATCAGTGTCCCAGCAG
28	27350489	SCAFFOLD115347_5344	0.27	0.28	GGGGACTTGTCCTGGTTCTTGTGGGATCTTTGAGGGGAGGATCACCCACAGGTGCCACAGGCAAGGGGCGTTTTTTACCACCCTCAACCCCCAGTCCTGT[A/G]CCAGGTACGGCCAAAGAAACAGTGGGGGCCTGTTTCCCTCAAAGGCATCAGAAGTGTCTGTCCACTCCAGCCTTCCTTTCTCAGCCTTCCCCCAACCCCA
28	27354033	SCAFFOLD115347_1975	0.27	0.27	GCGCTGGGCACCAGGGTGGGCACGAGCAGGCAGGAGGCGCCACGAGGGCCCACACACGCACACAGGCAGCCACAGGCCCTCCACAGAGAGCTAGCAGGGC[A/G]CGTTCAGCTGAACTGGTTTTTTCCCCTCCCACCCAGAACCACAGAACTCCAAAACCACATCCATCAGGAAAGACAAGAAAGCCGAACTGAAGGTTGACCC
28	27364335	SCAFFOLD5171_7397	0.28	0.33	GCCCAGAACACCCTGCGATGCAGGAGGCAAGGCTCACCATGTCGGACATGCCAGGCCCCAGGCGGTCGCACTCCTCCTTAGCGTGGTCCACCAAGGCCTC[A/G]ACGAAGGTGGAGTTGGTCCTCAGAGCTTCCTGGACGTCGGTTACCAGCTGAATGCAGTCCTGGCAGACGTTCCCATTAGCCTAGACAGACGGGGTGGTTA
28	27364565	SCAFFOLD5171_7627	0.45	0.45	AGCCACCCTTCAAATGGTTGAGCCACGGAGATTCTGAAGAACTGGGATGTTTAACATGCCCCCTTTCCTGTTAAGTTTCACTGTGATAAATGCCTGCATG[A/G]TTTTAAACTTCAGTCACTTTAGAGCCTATAAAAGCACGTTAGGAACTGTTTAGGGACTCACTTCTAGTGCTATAGTATGAGCATGTAGGAGTCTGAATAC
28	27364768	SCAFFOLD5171_7830	0.45	0.46	GGTAACTGTGGAGTCAGGTGAAGGAATGAGGCCCCGGGTTCAGAACTGTACACATTGCCCCTTCTGTGTCTCAGCTCTGGTTCCACCCCCAACCCCAAAC[A/G]GGGCCCTGAGCAGCCTGGCCCGGCGCCTCACCTTCTTGGGCTGGGGCTTGCTGTGGGAGCCGTCCTGAGGGTACAGGAGGAAGGGGATGTTGGCCATGAA
28	27550224	SCAFFOLD105516_9425	0.46	0.46	GGATGCTTCAATAAAGTTCACCCAATGCTAGATATCTTTTATCTGAAAATGAAGTAATAACCCACATATGTGTCCAAGCAGCCAAGTGTCCCCAGCCCTT[A/G]GATCCAGGAGCAGCAGGAGGAAGGCCACAGGAGGGGCTTCCAGGGAACCTGGCCTGGGCGGCCCCCTGGGCACTGGACAGGCCAGCCCACACTCCTGGCT
28	27699906	BES1_Contig680_1223	0.15	0.18	TGGGTGGTGCTGGCTGCTCACAGACTCCCCTACTGTTTACTCTCTGGGCTTACCTCTGCCTCCAGGCTGGCATCTAGCCTCTCTGCAATCAGTTGTGACA[A/G]CTCACTAAAAAGACTCTTTTGCAAGTCTCATGCAACATGCAGGAGCAACATGAGTTTAAAAGCCTTGCTCTTGCAGTTTCAAAAACAAGGAGGCAAAAAT
28	31375347	SCAFFOLD90022_6472	0.24	0.24	TAAATGAGCACCATTCAGCATTAATTCTTTCTTGTTTGGGTCATTAGTACATGTACTGGAAAGCTGAAAAGGAGAAACCTAGTGGGGATATTAGAAGAGC[A/G]AATAGGGTCCTGTCTTGAGACTTGCAATGCATTGACTTGAGAACTAGCAATGGGAGAAGATTTAAGAATGGAACAGTGTGACCTCTATACCCAGAACCAC

28	31495528	AJ496776-090.T7-622	0.46	0.42	AAAACGGAACCCACTATCCAACAGTGAAATTCTGAGTCATCACCAACACCAACAAAAGGCCTCCACATCCAGAAAATTCTCCCTGTAAGGTCTTTTAGAT[A/G]TCACATACTTTAAACTTTTCAGGCTTTCAACTGGCACCAAAGAGCTTTTAAAATT
28	31495721	AJ496776-090.T7-429	0.01	0.01	TCCGTTTTGCCCGAAGATGGTGATGAGATGTTTTGAGTGTCTCGAGTATGTAGTGAGGGGCTACAAAATATTGATGTAGGAAGTTGTCTGCTTTTGAAGG[A/C]GAATTTGYTCTTTGGGCCTATATGGTGTCCATTACACTGTATTCTTGAACAGGTTTATCTTTRAGGAACCATCCACCTACCTTTAATCTGTGGTACTCTG
28	31495784	AJ496776-090.T7-366	0.28	0.27	CAAAATATTGATGTAGGAAGTTGTCTGCTTTTGAAGGMGAATTTGYTCTTTGGGCCTATATGGTGTCCATTACACTGTATTCTTGAACAGGTTTATCTTT[A/G]AGGAACCATCCACCTACCTTTAATCTGTGGTACTCTGTGTGGGCCAGTTACTGCCCCAGTGCTAGAACTGGACCCAGATCCAGGTTCATTTGAGCAGTGT
28	31861798	BES10_Contig554_738	0.11	0.14	CTTGCTTGGCCTTTTACATTACAGATTTTTAGAACATAGTTGAAAATTTCTTTTTGTCAACAGGGGAGATCTTGCTCTAACTTTCTTTGCTTTCCTTTCT[A/G]CAAAATAGCAACAAAAAGGGGATAAGCTTATGTAATATTTAGTTCAAGGCACAAAAAATTCTCTAATTTTCATTCTCTCAGCTACTTGACTAGACTAGAA
28	32380107	BES10_Contig743_1320	0.43	0.39	GCTCTCATGGGCCAGTTTATGAAGCCCATGTCATCCTCTTCTCTGCTGGTGTGTGTTCAGCCTGGCCACCCTTTATGCTGAAGACCTTGAGCAGACCTTG[A/G]CTGAAACCTCAAGGTCTACTCCCTGTTCTCTGTCAGCAGTGATCTGTGGAATGGATTGGTGTGGGCCGCGTCGCCTTGGGCAGAGTTCATGTGTCATTCA
28	33779696	SCAFFOLD50661_8723	0.37	0.49	TGCAGTTATTATAAAGTGGTACTGGTGTAGGAAAAAAAAAGGTTAAGTCTAATTAAATTCTGGCAACCACTTTAGGGCTCTGTAGCAATGAGGATGCTGGG[A/G]TTTATGTTAATTAATTCTATATGAAAGGTTGCTGAAGTGTTTTAATATAATTCAAAGAGCCATGATTTTACTACCAGCGCGTGTTTCGATTTTTTTCCC
28	33783073	SCAFFOLD50661_5397	0.27	0.27	ACCAGACCCGAGCCCAGGAATACCTGGTCCCTATAGAGAAAGATAATCAACAGACCTTACAAAGTCTTCTGTGGCTGCTGGAGTGAAGGACGGATATGCT[A/C]TGTGCCCACTGGGCTCACGGCTAACCCACCAGTGGTAAAGTTGCCATAGGCTCAGGGGCACTGGTGGATCACTCTCATCTTCCAGACCTCTAATGGAAAA
28	34691253	SCAFFOLD106623_11509	0.32	0.45	CAGCATCCTGACCCCCAGAATCTGTGACTATGTTGCCCTAAACGACAAAGGAATTTGCAGATGTGATCAAGGCTACAAACCCTGAGATGGGGAACATACT[A/G]TAGATTATCCAGGCGGGCCTATGAGTCCTTTCCTAGCTATGGTAAGGATGAAAAGAAAGAGATGAGAAGCTTGAGAGGGATTCCATCCTCCCAGGAAGGG
28	34691475	SCAFFOLD106623_11731	0.14	0.22	TGACAGACCTAATCCAGAAACTTCATGGTGCCTTGATCATGGGCTTTGAAAAGCACTCCTTTCTTTCCCTTCCCAGCTTTGTCTTGAACCATCTGAGACC[A/G]TAACGATTGGTTAGATACTCCCCTTGCTGTGTGGGCTGGACACCTAGCAGACACTACATAAGTTGGGATGTCTGTTCAGGATCTTTGATGACCCTTTCGG
28	34691500	SCAFFOLD106623_11756	0.13	0.22	TCTGAAGTCTGGAGACCCTTTCCCCCCGAAAGGGTCATCAAAGATCCTGAACAGACATCCCAACTTATGTAGTGTCTGCTAGGTGTCCAGCCCACACAGC[A/G]AGGGGAGTATCTAACCAATCGTTACGGTCTCAGATGGTTCAAGACAAAGCTGGGAAGGGAAAGAAAGGAGTGCTTTTCAAAGCCCATGATCAAGGCACCA
28	35347393	SCAFFOLD35490_8458	0.10	0.12	TTCATTCTCCCTCTTGGAATCAAAACAGCATGTTTCTTTTCTGGTCAAGCACCCCACGCCTGGATTCCCCATCTAACCATCTCTACAACAGACCTCTGAG[A/C]GTGTACATGCAAGGCATTCCTTGCTGCTTGGCTTGTAGCAGCTGAACACTGAGAGCAACCTCAATGTCCATCCATAGGGGGCTGATAAAGTGAACCATGA

28	36209906	SCAFFOLD11756_13885	0.45	0.09	CTTCGGTTCAGGAACTGTAAGATCTTTGGTCTTTTTTGTTTCAGTTTATCCTTGTAACACTAGGTACGCTCTTTCAAATGACCCCTACTTTTTGCATGAC[A/G]GTAATGAGCCATGGAAAAAACAAACAAATAAATACATAGTTGACCTTTGTTGGCAAAGCTGACAAAGGTCCATCTAATCAAAGCTATGGTTTTTCCAGTA
28	36210456	SCAFFOLD11756_13335	0.23	0.30	TGGACAGGGAAGCCTGGCATGCTGCACTTCATGGGGTCACAAAGCGTCAGATACGACTAAGTGACTGAACTGAACTGACAGTTGACCTTTGAACTGTTGA[A/G]CTTTCAACTGACAGTTGACATGAGAGTTAAGATCACCTACCCTCCAGTGTTGAAAATTTTGTATTTGGCTTTCTGTATCCAAGGTTCTAATCCACAGATT
28	36866417	SCAFFOLD111828_22983	0.49	0.47	GGCACTTGCCCTCACCAAGAACTACATCACATCTAATATTATGTATTAAAATGCTCCATTTATGTGTCAACCCCTCTCTCTCACAAGGTGTTTAGTTTAA[A/G]CATCTCCAAAATTCTTTTACTCTACCAAACTCTCCATTTCCTGCCGGGGTCCAGCCCCGGTGGATCCCCAGGGAATTCGAAGTGGGGACGGCGTCTGCGA
28	36875801	SCAFFOLD172016_4142	0.46	0.48	GTTCTCTCCATTTCACCAAATGGTATTAGGAAAAGAGTAAATATAAAAGAAATAGTCTCAGCAGAATTGGGGGAATCGGAAATGACTGTAAAAACAGTAT[A/G]TATTAGGTGTTATCTGATGTTGTGCACTCCTCATGTGCTGATCGAGTTCTATGAAGTAGGTGAGCAGTGATATCTGGCGACAGCAAGGTCAAAGTGAGAT
28	36875848	SCAFFOLD172016_4095	0.45	0.47	GAAACAATGCATGCTACTCCCTACCCCCATTTAAATAGTCAGCTCAAATCTCACTTTGACCTTGCTGTCGCCAGATATCACTGCTCACCTACTTCATAGA[A/T]CTCGATCAGCACATGAGGAGTGCACAACATCAGATAACACCTAATATATACTGTTTTTACAGTCATTTCCGATTCCCCCAATTCTGCTGAGACTATTTCT
28	37860949	SCAFFOLD166988_3548	0.33	0.39	AAAATCATGATATTTGGAGGAAATTAAAAGATTGACAAGAGGCTATGCTGTTCAACTATGCAAAAGTAATTGAAGACCCAGTGAAAAAAGGAATATTTTT[A/T]AAAATTTTCAATACATTAAATTAATTCCAACACAAACAGAATATTTATAGACCATTCACTATGGAAAAACTGAGTTGTGAAAATGCTAATCCTATGTCAA
28	37861196	SCAFFOLD166988_3301	0.16	0.13	AACAGATTAAAACACTACAAATCTATTTAAAATGCTCAATAACATAAAGAAAACCAACCAAGCCTCTTTTATGAAGCTAATCTTACAATAACATAAAAAA[A/C]CAACAAAGAATTTGTTAAAAAATGCAAAACAAACAGTATTATGATATTATGAAACACAGTTCAGCCACATATTAAAATGATAACCACATAGCAAACCAAG
28	40041354	SCAFFOLD205332_29182	0.15	0.13	CATATGAACTCATCTTTAGACCCTCAGTTAAGCAAAGTGGTGGATAGCAAGCAAATCAAGTCCTTGCATCTTCCATGTTTACATCAAAACACAAGCTTAT[A/G]TGGCAGCGGTGTGTTCGTCAGCCAGTGTCGACCCTGTCAGCATGCCACACCTCAACTGTAGGGCCTCAGCCCACAACATACTTGGGTGCTGACGGAGTGC
28	40252272	SCAFFOLD265551_632	0.28	0.40	CCCTCTGTTAGTTAGCTGGCCTCTCTGAGCCTCAGTTTCCAAGTCCTTAAATAAAAAACAAAACACAACACACACAAACACAGGATTAGAAGTAGAGCCC[A/G]TAGCAGAGGTTGCTGTGAAGATGAAATGAGGTGACGTGTGTTCAGTCCTTAGAGCAGGGACTGATGCAAACTCAGTGGCCGTCACCAGGAGCAGGTGTTC
28	40326167	SCAFFOLD241720_921	0.36	0.30	AGCTGTTATCCCCGCTCTGGTGTTCAGCCCTGGTTCTGGATGAGTGGTCCTTGTCTAGAGCAGGCCAGCGACCTGCCATGGAGGGTCTCAGAGGCCCTGG[A/C]GCAGAATCTCCAATCTTACTCTTCCTTCACTATTCACAAAATTCTTTGAGTGAAAAGCCTATGAAAGTTCAAGCACTACTTAAGATCCCCTTGTCTCCAA
28	40379026	SCAFFOLD275553_9017	0.47	0.40	TAGTGGGGGTGACCTGATTGGACCCCATTTGGTGGGCACAGCAGATCTGCATTAGTTCCATGTTGCCTGACCTCAAGAGCATGGCACACAGAAGGTGCTC[A/G]TGAGATGGCAGCAGGAGGAATGAACAAATGCTGAGTGAGTCCTAAAGGGCTGATATGGCAGCCTCTCCTGCCCTTGGGGCATGCGGTGGGAGCCAGGAAA

28	40686825	BES11_Contig401_365	0.46	0.47	TCTAAGTCTAATGTCTGTGGAATATCATTTCTTCAACAAGTGTTTGCTGAGCACAAAGACCAAAGAATCACTTTGGATACTGAAGACACAGCAAAGGACA[A/G]CTCAGATAAAGCTTTCTTCTCTCTCAGCGTTCATGTGAGTGGGAAAGTGAACAACAGTCCCATTTGCTCAGTTTCTTCAACATAGCCAGGATCCTGCCCC
28	40686884	BES11_Contig401_306	0.11	0.08	CCAAAGAATCACTTTGGATACTGAAGACACAGCAAAGGACAGCTCAGATAAAGCTTTCTTCTCTCTCAGCGTTCATGTGAGTGGGAAAGTGAACAACAGT[A/C]CCATTTGCTCAGTTTCTTCAACATAGCCAGGATCCTGCCCCATCCCCTGCTCAGTACCCTGGCCCAGAGCAGCCCCATCCTGGCGCGGCAGCATCTTCTA
28	41026692	SCAFFOLD96764_11685	0.33	0.37	TGTCAAAGGGCAGCAAGAGTGTGACCTGAAGTTGACGCTCACTCTGCTTACCGCACAGTAGGCCAGTTAACCAAGAGACAGTGTTGAGGCAAGAAATATA[A/C]CTGTATTCGGACAGACAGCAGACCAGAAAGATGGCACAATAACCATCTCAAAATAACCATCTTCTTGGGGTCTGGTTGCCAAGTTCTTTTATAGAATGGA
28	42698889	SCAFFOLD125974_10424	0.12	0.13	TCTCGAGGGCCCTTCATGATGGGCCTAGGTAAGGGTCCTGGGAGGACTCTCCAGGGGGTGGGGCCCTGCTCAGGACCAAGGGGATATTGGTACTGACTCC[A/G]TTTATCACCCACAGATTGGAGCCTGGTTACCTGGACTCTATTCTAGTGGGGCATGAACACAGTTCACACCTGGTCCATTATTCCACCAGCCAGGTTAAAC
28	44007257	SCAFFOLD186736_5402	0.43	0.36	ACTCAGTGATAGTTCTGGGACTCAGATTAAGTCCTCAGTCTCCTCCTTTATCACAGCTTTTCTCCTATGATGAAGCAATAAGGCACTGATGAATTGTGGT[A/G]AAGGGCTGGGCTCCCAAAAGTTTGGAGGTGATGAGTTTACCTTTAAGCACCAAGGAACTAAGAGCACAGCTCCTCAGCCTCCCCAGAGATTTGTTTCTTT
28	44950631	SCAFFOLD215469_500	0.28	0.30	CATCTAATCCTTGCCAGCAACACTTTGAACAGGCAAGTTGTCTTTGAGTTAAACCAAAATTGGGCCAGAGGACTGAGAAATTGTTCAAGGTCTCACAGGC[A/G]TGATGCAGTGCTGGCACGATTGAGACACAAGTTCCTCCAACTCCTAAACCTGTGCTTGTGAATAACACGCAATGTTGAGGATGTTTAGGGACCAGCCATG
28	44953601	SCAFFOLD121397_7042	0.04	0.06	TTTGGGTGCCTTCCACAATCTTTTAGGTAAGTTGAACACATCCTTTGGTTCTAAGGAAAAGAGCTTGTAAAATGTTTGGGAGCTCTTGGGACATCAAACA[C/G]CTCTTGAAGGAGTGCATTTTAACGGTTTACTTGTAAAAGAATCAGTAAAAGAAGCAGAAACATATGGTTTGAATTGCTCTGAATAATACGAGGTATTTTT
28	44954013	SCAFFOLD121397_6630	0.27	0.35	GTCATGTATAATTAGGATACAGTGGTAGCTGGCCCAAGAGTTCAGGTTTAAAAGCTTACAAACCCCGAGCAGATGACATGAAAGCTTGAACTGGGCCCTT[A/G]CAGTTTAAAATGGAGGCTTGATTGGGGTGAGCAACTTGAAACTGGTAAGGCTCTTGCTGGCCCTGGGGGTGTCCCTGGTGGCGCTGGTGGTAGAGAACAC
29	266311	SCAFFOLD322270_2452	0.31	0.23	GAAATATATTGAATCTGTTAATTCAATGTCTTCCCCAAAGCATTTCTCTTACACGAACCTTGAAAAGAAAGCTCTCAGCATGATAATGAATGGTGTGTAT[A/G]TCCACTTCATCTCCTACTCCTAACAGGTACCAGTTGGTCATTGTGTCTTCATTCATGACGAGTCCATGAAGGTTCCCATAAATCTTCCCATTAATAGCTA
29	588291	BTCN44447-2	0.05	0.06	TTTCAAGTCAACTGCATAAAAAAAGTACCATCTCTCCATGTTGGTATACCCTATAACTGTACATAAACTTTTGCATTTTAAAGCACTTGTCACTTATAAA[A/G]TGAAAGGTTTCAAAATGCTAARTAAACATTTGCTAATTATTACTTTTTAAAAACAATATTCCTTTAGAAGATTCCTCTTCTTTGTGCTTATTGTTGATCT
29	773943	SCAFFOLD245583_8667	0.11	0.12	CATCGAGGGGCAAGAGTACTTCAGTGGACCATGTTCCAAGTTAGAAACATGTTTCGTCAATGTGGATTTAGATTGTAACACGTGTGTGTGTGCTGTTAGT[A/G]TGCCAGTGTGTCCAAGTGCCCTAATAGTTGTATCTAGGATCACTCAGGGGAGAAGGCAATGGCACCCCACTCCAGTACTCTTGCCTGGAAAATCCCACGG

29	5434557	SCAFFOLD115406_9951	0.46	0.13	TTTCTTGAATTCTTTCTATATTACTTTCAGATCTGTCTTCTTTCCAATCAGTTCCTTTTGTTTCTTTCTGATTTGCTTACAAATCTTTTGGCCTTCTTTT[A/G]TAATAAACCCTTTGTAGTAATTTTCTCCCTCCTGAAGAATTAGAGTTACCATCTGATAATCATGTCTCCATATCCATTAACGTCAAACAGTTGGGAGAGG
29	7688436	SCAFFOLD51667_429	0.46	0.38	TTCCTGGTTGTGTATTTAGTGCATTTTTTATAGAGAAGCAATGAAACACTTTGCGGAGTGATCTGCTCATTCTGGAAGCCTTGTTAGGGTCCAGTATATG[A/C]TTCAGTTTACAACAGAAGTGAATTTTTGATTTCTGTTTCTTTTTGGTGATAGGTCAAGAAAGATTTGGAAACATGACACGGGTCTATTACCGAGAAGCTA
29	9372989	SCAFFOLD155699_9849	0.23	0.19	GATAAGGAAGTGCAGCTTGGGGAAGGGTAACCTGTCCACAGCCACTCGGCCAGTGAGGAGCGAAGGCAGCACCTGAACCCAGATGCTGTGGCCGCAGAGC[A/G]CATACATTTAACACCTCCTCTCTGCTACCTCCCCCTGCCATTTCTCTGAACTTCTCTGGAGTCGCTTGCACCCTCATCACTCTTAGTGATCTGTTTTATT
29	9597160	SCAFFOLD119679_516	0.21	0.19	TTAAAAAATCAAATATGGTAAAAACGTTTTTAAGCAAAATACTGTGGTTTTCTGCGCATGATCTATTCCCCAGAAGCAAGCACTTTCAACATTCTTAGCT[A/G]CTTCTTCTTCTATTTACCCTGTACCTATACGCAATTGTTGTTCTATCCTGATTCATTCATTAGTTTTGGCATATTTTGACTATGTTAGAAAAGGATTTTA
29	11434269	SCAFFOLD60825_4496	0.32	0.47	TCACTCTGGCATATAACCTCTGTGTGCAGAGAAACATCACACAAAGAGACGATTGCTGTTCCATATGGCACGCATAGACGGCTCAGAATCATGATACATT[C/G]GGTTCCAATTTATCTGTCAGTAGCAAGAAAACTCTTCCATGTGTATAACCAGTCCAGTCTTCCTAAAGTATCTGGAAATAGATCCAACATCTGTTCAATT
29	12129567	SCAFFOLD145299_12523	0.07	0.18	TGTCATTCTCTGATTAGAAAGTAACGCAGTTCATTGTAGAAAATATGGGAAATATAGATATATATAAAATAAATTAGGATTGCTCACAATCTTATCAATA[A/G]AAGGATCTATTTATTATGTTTCTTCATAGTCAGTGCATGTTTTTACATAGTTGAACTTTTCATCCTTCCTTTTTCATTTAGTATTACAGTATAGGCATTT
29	12129990	SCAFFOLD145299_12946	0.49	0.00	AGATTCCCTGGAGGAGGGCAGGGTAACCCACTCTAGTATTCGTGCCTGGAGAATCCCAGAGACAGAGGGCCTGGCAGGCTGTAGCCTATAGTGTTGCAGA[A/G]TCAGACATGACCAAAGTGACTTGCAGAGAAAGCGAGGTTAACGTAGGCCCAGATCAGTTGTCACACATTTCTTGCTTTAAAATGCTTGAAAATGAGCTCC
29	12682820	SCAFFOLD266585_14176	0.31	0.32	TACAGTCCTGGCTCCACCACTTACTAGATCTGAACTCTTGTACAATCACTTACCTCTTCTGAGCTTCAGTCAGTTTTCTCACACTTAAAATGAGAACGAT[A/G]CTACCTAATATGCCCCCTGTTTATCAGATGCTGGGTGTGTGTCAGGTGCTTTATTTTATTTATATCCTCTCCCTAATTCAATGAGAGATAAAGGTAATAT
29	14498603	BES2_Contig389_1070	0.17	0.21	ATATAGGAATTTTTTTCTATTCTTCTTACTAACAGTGATAATAGAAAATTTAGTAGTTTTGTCTTTGAATCTGAAACTAAACAGAATTGTATAATCCTTT[A/C]AATTTGTTCAGTACTGTCAGCGTGAGTAAATTTCCTGGTGGAAAGATAAATGTTAATAAAATTTGACTTTATACCTTCCAGCAATACCTAGTTTGATATT
29	15536124	SCAFFOLD82824_594	0.20	0.32	AAAGCATAACTAAATCTGAGCATTTCTGGCTGGATTTTTAAACCCCTATAATCTGGATACACCCTATCAACTCACTGGTAGTTACTGTCTAAAGTAATCC[A/G]TCTGCTCAGCCCACACTACATTGTTATCTACCACTTACTGTTCATTCTGTGGCCTCCACCTTGGATACTGTCTTCCTTTTCCCCTACTTCAGTACATTTC
29	16888647	SCAFFOLD100520_11158	0.01	0.01	ATGTACAAATTTATGCTTTTAAGTGTTTAGTTTGGGACAAACATAGTGTCATGTAACTGCATATAGTCAAGATATAGAACAGTTTCATCATCTCCCCCCA[A/G]AATTTCCCTTGTATCCTTTTGTAGTTTCCTTCTGTTGCCACCTCTAGTCTCTGGCAACCATTGATTTGTTTCTGTCCATCTAGCTTTATATTTCCCAGAA

29	17382133	SCAFFOLD175305_6177	0.22	0.34	ACATAGAGTTTATGTGAGAATTAAATTATGTAAAGTGCTTAAAGTGATGATTATTATTTCAGTTTTTAAAATGGAAGCTAACTGGACTGGAAAGGCCTAG[A/C]TGGACGGCAAGGAGCAGGGGTCTTATTTAAGGCAATTCTGTATCCCCTACCACATGCAGCCTGGGGGCTTGGCATTCATTCATGCAGAAATAAATCAGAT
29	17757674	BES5_Contig608_1742	0.05	0.20	CCAGTTGCTTGACAAATAAATGCAATTATTCTTGCCAGGCTCTGTAGATGGTATAGAGGGGTGGAGGAGGCATTCAGGGCAAGCAGGACCCCAGAATCCT[A/G]TGTCCTCTCTGTTACCCAGAGCTCCTCACTCTGAGCTACAACTGCCAGCCTAGACACTGAAGCTATTATTAACTTAGCCAACTACAAGAATGAAGCTTGG
29	18258032	SCAFFOLD90229_1703	0.02	0.05	GAAGGCAGGAGAGCCTCATACAGTGTAAATTTAAATCCGTAACCTCATGAACTCACAGATACTTGAACATTGCCTGTTCTCCCTCTAGGCTGTAAGCTAC[A/G]TGAGGACAGGAATTCTGTCCATCTTTTCCTCTGCTGTAACCAGGGTGCCTGGTACACAGGAGCACACCCAATACTCGTTTGTTGATTAATTCATTAGTGG
29	20935926	SCAFFOLD145192_9213	0.42	0.38	TTCATTGAAAAAGCACCTTATGGTAAAGTTTGGAAGTAAGGTGATGTAAGACAGAAGGTGCAGTTTGGGTGTGTTAAATTGATGAGGGAAGATATAGTGG[A/G]AAGATACATGAGGGAAATCTATGCTTGGCTTCTAGCTTCAGAAAAAATTCTGGAATTCACTTTTGGTTTCTAAAATTCCAGAAATCTGAAGTAAATTAAAA
29	21244296	SCAFFOLD260989_933	0.21	0.28	TATATCATTCCACAGGGCCAAAAAAACTCCTAGAAGTGCATGGTAAAGATATTTGGCATGAATGAGGGGGAAATACGAGAGAGTAGGTGGCAGCCAGGGT[A/G]GAAGTGGCTTTGGATAGTCACAGAGGCGGAGTGTTGAGCCACGCTACACCTGCCATGCCCACCAGCCTGCGTGAATGGGCTAACCACGGACAGCAAAGG
29	25939788	SCAFFOLD25027_24456	0.27	0.28	GGAGAAAGAGGTGGGGGGGTGGGGACGCTAGGGTGGGGGCTGTGAGGAAATGGTTCATGGAGAGGGGGGCGCAAGGAGTAATTAGGCTGACATATGCACC[A/G]CCCAGGACCCAGGAGCCAAGCCAGGGAGGGTCACTTCCTGATTTACCCCTGGCGTTGGGGCACACCTCTTTGCCAGGGTAGAAGAGAAGTGTGCACTGGG
29	29323428	BES11_Contig430_1266	0.18	0.23	ACCAACCCACATATTGTGTTACTGAAATTTGCCATTATTGCTGAGGACCAGCAAAGGGATTCCCAATTAGAGTCAACTTTGCATCCCATCTACGGCTCTG[A/G]AAGAGGGGCTCCTGCTTCTCTTCTTGGATAGCAGGTATAGAAATTGTGGACAGAAGTCTAAATGAGCTCTGTTTGTGAAAACTACATTTTTTCTGGTTTC
29	29741733	SCAFFOLD50303_4748	0.31	0.28	TTCCACAAGTCCTGATTGAACCATGAAGTCTTGAACAAGTATATCACACAACTCCTAGAAGCCAACACCACGGTCACATGATCCTGGTGGCCAATATGGC[A/G]GTGCTTCTACACTATAGAATGGAATACGTATTAATAGCTTTTACATTATTGGCTTCATTTTACCTTCAGGATGTTACTGGAAGTTTTATTTTTGATACAA
29	30186139	SCAFFOLD160379_10089	0.09	0.14	CAGGGTGGCATTTCTTAGTCGTGTCTGCCTTGGACACCATCTGGGTTCGGCCTCATCTTACTGGTGCTGCTGACATATTGCGTGGCTTTGGAAAAGTCAC[A/G]TCATATCTCTGACTCCCAGTTATCTCCCATTTATTAAATGGGAATAAGACCATTTGAGGAACAAATGAGATACTAGTTTTGAAAAGGAAAAGGAATTGTT
29	30453999	SCAFFOLD208955_20939	0.12	0.26	CTGTGTGGACAATGGCAGCGCCACCAGAACCCACCAAAGGTCACAACAGGTACCACCCCATCCCCTAACACCCCACCGCTGCCGTGGCCAGGAGCCTTGC[A/G]TGGAGGCAGCGACACGTCCTTTCTCTGGAGGTTTGTACCAGAGCGCCGAGGGACACCACACGCCAAAGCACATGAAGCATACTAATTAAATGAAGGTTTA
29	31890059	SCAFFOLD280067_19358	0.49	0.03	TCAAAATAAATAAAGAGGGAAGTTTTGCAGTGTGGCAAATTCTCCTCTTCTCCTCCTAAATCATGTTTATTTGCTTACTTGAAATTCACTACATGCCCTC[A/G]GCATCCAGCAATGATTCTGAGGCTCAGCTGGACACACACTGGGGAGAGGGAACAGCCTTATAAGCAAATGAATGACAAACACCTCTTTGGCTTCAGCATC

29	31890131	SCAFFOLD280067_19430	0.11	0.09	AAATCTTCACTCCTTACACTAAAACTACTTGGATAGTCTAAAGCACTGTCATAAATATAAGACACCAAATTGTCAAAATAAATAAAGAGGGAAGTTTTGC[A/G]GTGTGGCAAATTCTCCTCTTCTCCTCCTAAATCATGTTTATTTGCTTACTTGAAATTCACTACATGCCCTCGGCATCCAGCAATGATTCTGAGGCTCAGC
29	33284439	BES2_Contig422_801	0.28	0.27	AACTCAGTTTCTCCTGAAAATCTTGGCAGAAACACCTGATATTTCTTCTTTAAACCACACACTGGGTGGACTCGGGAACGACAAGCAAATTATAATAAGC[A/G]TGCCTCAAAATTCTCATAAGCTTGTGTTTGTCATGATTTTGTAAGTTTCCCAAGGCCTTCTTCCTGTTTTGTTTTTTCATTCATCTCTCTCAAAGGCTCA
29	33284835	BES2_Contig422_1197	0.42	0.50	TGAGGGATCTCAGAAGATCCTGTCTGACTTGGAGCATTTCTAAACAAATTCAACCTAAGAATTCTTTTCTGCATCTACTTTTCTGTGTGTGTTTTTGTGT[A/G]TGTTCTCAGCACAGATTTTGGCTACTATTTCTCTAAGCAAACATGTGATTCTAATGAAATTTGCTGAATTTCAAGCAGCCACACGTGTATGAAAGCTGCC
29	33284894	BES2_Contig422_1256	0.50	0.01	TTTGCTTAGAGAAATAGTAGCCAAAATCTGTGCTGAGAACACACACAAAAACACACACAGAAAAGTAGATGCAGAAAAGAATTCTTAGGTTGAATTTGTT[A/T]AGAAATGCTCCAAGTCAGACAGGATCTTCTGAGATCCCTCACTGCTATTATTATTGGTAATATAATCTGAATATCTCTCTTAATACTCTGTCTCAAGAAA
29	34324645	SCAFFOLD125425_2197	0.42	0.37	TGGGGCATGTTGTTTAATGATAGGGGCATGTTGTTTAATGGTAGGAGCTTGGAGAGACAGCTCCAGTTGTCACAGCGTGGCCCTAACAACACAGACAACC[A/G]AGTTCCTACAGACTGTTTTTAGACCTTCTTCCCAGGGAACACAGTCCTACCTCGCTTCCCTGGAGCACTGCAGTGTATGACTGGCTTCCCTTACAGTCTT
29	35007501	BES1_Contig509_596	0.28	0.35	ATGCTAGCTTATTTTCAAGTAACGTTTACTGAGCATTTATTAGGCATCCCTGCACACAACGGAAAGCTGGGCCCAAACAGAACATTGAGTCCCTGCAGAA[A/G]GGACCACAGCCCTGCCGTCCTGCCCTGCGTCCCTGGAGAAGGGAGGCGGCAGATTAGGAACACACGGGAAAACCCCTGGAAGAGGATGCCCAGTCGTACA
29	35115470	SCAFFOLD265215_4647	0.40	0.43	TGCCATGTGAGGATACCGTCATCATAACGTATCGTGAGGATTCAGGGCTGGGGCGGAGTACCCTGGGCCAAAGTGAGAAGCTGGGGCTTAGCTATGTATG[A/G]AGCCGAGGCACCCACACCAGCCACAGATGCCTGCAGGATAGTGGGCTGTTAGCGCAGCCCAGTGCAGTGAGAAACGGTGACTTTTCAAATATGCAATCTA
29	36374464	SCAFFOLD165127_16206	0.18	0.21	TTAATTACTGCCCCTTGCATTTTCAGGCACAGAACCACTAGCTTCTAGCCTAAGGATTCAAGCTTGCCAAGAGATCCTGGCGTTCCTCGCTGCGGGCCGC[A/G]TGATGACAATGAAGTGGGGCTCTCATCAAGAGGTCCTTATAACCATGACAACATGAGCAGCACCGCTCTTCTGAGGATGTCATTGCCATGACAACACCTG
29	37418951	SCAFFOLD141082_7705	0.10	0.20	CCAGCCAAGCTAGACAGAAATCCTGTGAAAGGATGAGATTAGAAAGACATCCAAAGTCACCTCTTCCCCCTCCTTCCTGGCAGACTCTAGCCTCTTTACA[A/C]CATTTGGTTTTGTTTTTCTACAGAGCACATGCCAGACCTGGCTGAATCCAGGGCTCAGCCCCCTCCGATATCCCTCCCATCTCATGACATTCTTCTCGTT
29	37728774	SCAFFOLD170015_32126	0.33	0.32	CTCACCCAGAAACCAGGACCCGGGGTTTCCATGTGCAACGTGCTGCAGGGAGTGACACTGTGCAGACGCACCCTCCTCCACTCAGCTCAAAGGCTGTTTT[A/C]CCTCCCTGCAGGCTCCACCTCTCCCCATCACCAACCTCCACTCTGCGCCAGCCCTCAACCACCCGTCACCAATCTCCTCTCCGTGCTAGCCCTCAACCAC
29	41985033	SCAFFOLD273170_4202	0.29	0.29	GGCGAACACTTCAAAGTCCCACCACACCTAGAAATGCCAAATGAAAGAAAGAAGTGACTTGTCCAGGGGCACATAGCAGGCAACTCAAAGGCCATGCAGG[A/G]ATGTGTCCTAGCAGAGAGCAGGGCTCTCCCCTCTACCACACTCACTTTTGAAGGGGGCCCTGGCTGCCCAGCCCAGCACTGGGGCCAGAGGCTGACACGA

29	44221429	SCAFFOLD115515_7081	0.13	0.07	ATATAGAAAAAAAAAAAAAAAGTCCCTGCACCAATTACCTGTCTTGGAGGAAGCACACTGCACACAAAGTAACTCTTGATACACTGCATCTGAGGTGTAG[C/G]GGGGAGGTGAGGACGCGGGCGGCCAAAGAGAGCTCTTCTCGACATCAGAAAGGAGCTCGGCCCAGCCCTGGACACCCTGAGATAGCACTGAGCTGAAAAC
29	44392572	SCAFFOLD100157_5531	0.08	0.13	CTTGTCCTAGACGGAGCCCCCGACCTACCCCTCCAAAACCTGCAATCTCATTTGTCCAGCTGCTCAAGCAGAAACCTAAGAGACATTTATAATTCCTCTC[C/G]CACCCCAAATCTAAGCCACTGACAGGTCCTGGTGGGCTTTACCTCCTAATCCGTTCAGAAGCCCACCCCTTCCTCCCCTGGCAACTGTTTCTACCCTTGA
29	44771349	SCAFFOLD115786_4123	0.46	0.11	AGCCCCACAGGAGAAATTCCTCTCAATATTTAGTGCCTGCTCTTGTAAGCTGAGTGTAAACTGGTACCAACTTTCTTGGAAATGATTTGACAATGTGTAT[A/G]AGGGGCTTTAAGAATGCATACTTTTTTGACTCAGAAATATCCCCTTCTAATAATTATCTTGGGCTTCCCCAGTGACTCAGCAGTTAAGAATCTGTGTGCA
29	45220632	CAPN1-AF252504-5082	0.23	0.28	CTTCCCACCACCTCATCCTCACAGARCCCAGCTCAGCACCCAGCACCCACCGTTGGAAGCACAGCTGAGAAGCCTGTCTGGGCATAAACTGCTGAGAGA[A/G]GCAGCTCTACACACAAAGTACAGTAAATCAGGGTGGGACAAGATAACCATCTTTGGGCGAGAGTCCCTGGGAGATAGGAAGGTGCTGCTGAAGGTCACGG
29	45237834	CAPN1-AF248054-4558	0.18	0.14	CCCCTGTCTCCCCCTTTCCTCCCACCACACCCTTGCTGCCCCAACCCCCGTTGACTGGCCCCTCTCTCTCCCCACCCTCTGCAGAGAGCTGGATGACCAG[A/G]TCCAGGCCAATCTCCCCGACGAGGTACGTGCCCTGCCCCCACCCTGGGTGCACGACGGGGACCCGGGTGTCCTGTGTCTTGGTCTCTAGCCAGCAAGGCA
29	45237961	CAPN1-AF248054-4685	0.20	0.19	AGCACAGACCCTGCAGAGAGCAAGAACCCAGGGGCCCTGCGCCATTACTATCGATCGCAAAGTCCTTTGCAAACTGTAAAGTGCTTGGCAAGTGAGGGAT[A/G]TGTATGGCTCCTGCCAGGAGGGGCTCTGCCTTGCTGGCTAGAGACCAAGACACAGGACACCCGGGTCCCCGTCGTGCACCCAGGGTGGGGGCAGGGCACG
29	45238181	CAPN1-AF248054-4905	0.49	0.03	AACCCAGTCCCTACTGTATTCCTGTCTAGAACTCTTGTGGTCTAAATATGGAGGTGGAAATACAGCCCAATGATGAGGGGTGAGCCTGGATCCCTGCTGC[A/G]TGACCTTGGGTAAATTCCTTAACCTCTCTGAACCTGAGTTTCCCTCTGAGAGGAGAGACGGTGACAGCACCCTGACACGGTTGTTATGGAGGAAACCGGG
29	45240643	CAPN1-AF248054-7367	0.20	0.14	CCAACTCAGGGATCGAACCCACGTCTCCCGCATTACGGGCAGACGCTTTAACCTCTGAGCCACCAGGGAAGTCTAGTGAGGACAAGAGGAAATGCTAAC[A/G]GTTACCAGGAGAAAAAGGCAGTAGATGGCTGTTTGTTATCACTTCACCACCACTGCCATCAACAACCCCACCCCACCCACTGACAAGGCCCCATGGGTGG
29	45241089	CAPN1-AF248054-7813	0.21	0.18	CAGCCAGGGAGAAGCGCAGCCAGCCTCCAGGGCCTCCAATGGGAGCTGTGCAGGGCTCACCCTCACAGTCCCACTTCAGGATGAGGCCAGGGCTTTCCGA[A/G]CAGATGAAACTGGACCTGTGGGCCTGATGGCCAGCTCCTGGGTGGGTCGTTCTGGGTCCTCAAGCTTTCTCTGGGCCAGCACTTGCCCAGGACTGCCTC
29	48271992	SCAFFOLD296812_510	0.25	0.28	TTACTGACCAGGCTTCTTGGCCTCCTTCACCAATAGACATTGATCAGAGGCCAGACAAGAAATTCGGGCAAGGTTTTAGCGGGGACCCTGCTGCTGCGGG[A/G]GGGAGCGAGAACGAGTAACAGGCTCCCGGGCTCCCTCCCCTGGGGCTGGGAGGGACTAACTGTTTCCTTACATGGGGTGAGGGTCAGGGTGTGTCCAGGG
29	48956564	SCAFFOLD126896_5229	0.16	0.25	CAGGCTTGGGGATGGAGACTTTCCTCCAAGCCTCTCAGTGCATCACATTAGAGCTCGATTGGCAATTAGCAAAAGTCACAACCCCTCCTCTCGTCGCCAC[A/G]GCTGTTTCAGATGTCAGATTCCTGCCAAGCTTCGCATCTTATTCTAAAACCTCTTTCCAGGAGACTCGGGGGAAATTAACATTGTGCCAAATGAAGGAAA

29	49118396	SCAFFOLD110435_21192	0.11	0.10	CACTGCGCGCTTTCTGCAGGCCAGGTGAGCTGACCTTCTGCTTTGCCCGGGAAACATCCCCTGAGAAAGACTCTCTGGCTTTGCCAAGGAGGGGAGCTCC[A/G]GAAACCAGTGTGATGGTCACTGAGTTCAGCGCACCAGGGCCCCCGGGGGATCAGCCTGGCCAACCTCCTCACTTGACAAGTTGAGAAACCGAGGCCCTGA
29	50676260	SCAFFOLD90580_12258	0.05	0.06	CTGTTGGGTGCACCCAGGTGGGCCACATGTTGGGGGGCTGTGATGCGGGGTCCCCCCACCATCCCCAGACATGCTTTGCCTGGGCTGCGCCAAATTCCCC[A/G]TCTCCAGCACTACTCTGAGAGCGGCCAGGTGACACAGGTCCACCCTGGCATCGGCATCTCGGGCCAGGGTCTCACACCACCCCAAGAAGCCCCCGCCGGG
29	51109326	SCAFFOLD252706_2287	0.44	0.43	GAAAAGTGGATTCTTAACCACTGCACCGCCAGGAAAGCCCTTCATGATCATTTTGCGTCCTTCAGCCATCAGGACAGCTCCAGTGGGTGTCTGATAACAA[A/G]GCAAACGCAAGCGGGTGTTTGATGACTCCACGCAGATAGAAGTTCTCTCGTCTGAGGTCCCGGTGGTCATCACTAGGAGGTGCCCACGGCTGCTGCCCTG
X	420053	SCAFFOLD286772_8345	0.25	0.18	TATTGTTCCCAGTTATTGCTCCAGAGACTAGTGAAAGGCTGGCACATTTCTTACTTGAAAATACAGATTTGCCTTGCAGCTATTCATTCTTTTGGGCACA[A/G]TTCTAATAAGCATAATTATCTGATTTATCAATCCCTGGTAAATTGCAAGTGAGGAGGAGAATTAGGGCAGTAAGAGGCAAAGAAATGGAAAACAGAAGTT
X	420108	SCAFFOLD286772_8400	0.28	0.21	CATTGAGCTGGCAGCCAAAGTTGATGTCACAAAGTCCATAAACTCTAGGATTGCATATTGTTCCCAGTTATTGCTCCAGAGACTAGTGAAAGGCTGGCAC[A/G]TTTCTTACTTGAAAATACAGATTTGCCTTGCAGCTATTCATTCTTTTGGGCACAATTCTAATAAGCATAATTATCTGATTTATCAATCCCTGGTAAATTG
X	420541	SCAFFOLD286772_8833	0.26	0.20	AAACAAATTCAAACTCCTCTCTTTCCCAAATTGGGTATTTGACCCAATTTGAAACTAGTGAAACGACTTTAATAAATACTGCCAATAAGGCTTTCATGTA[A/C]GACAAATATTCTTAGATCAAAAAAGCAATTCCACTAATACAAAGATCTTTCTAGATGCTGTCATTTACCTAAAATGTTCTCAACTAGTATGTGAAGTATT
X	1111368	BES9_Contig308_1451	0.10	0.12	AATCAAGAGTCACAGATGAGGAATTTTGATATAATTGAATGTGAAGCATCTCATCAGAGGAAAATGGCTTTACTTTAACATACACAAATTTAAGAGCACA[A/G]GATATTTTGAATTGCAAAAGCAATCACTACAAGTATATGCAAAATTAGTTACATTTTGTCAACATACAGTAACTTTTTCTATTCAAGAGAGATTTTTAAA
X	7641750	SCAFFOLD68962_9331	0.23	0.30	CTGCCAACTCTAGCCAACAGCCTGGAAGGTTCCCCACTGTGGGAATATTGGGAGGCGCCAAGCCTGGGAAGTGAAAGAGACACGATGAGAACTTTGCTCC[A/G]AAGTTCTTCAGGGGGCGGCCTCTGGGAATGCCCTTGCATCCACTATTGGTCTGGTTTTGAGAATCTGATTTGGTTAACTGTTGACCCTCTAGTGCTTGCG
X	11023343	SCAFFOLD235262_6861	0.24	0.37	AGTTTGAACCCTGAGATATGAGCTTTTTCCTGGCTAAATCTCTTGGTACCAAAAGTGTGGAAAGTGAAAAGTTCATTAACTTGAAAAATCTTTAGCCAAT[A/G]GATAGGCAGTTTGTCTCTCTGAGAACTGCAGTGCCCAGAACGTCTCTAAGATTGACTTTGGAATCAAGCTAAAGAAGCACTGGTGAACTATCTCCCCTGG
X	11028013	BES4_Contig343_1134	0.44	0.34	TTAAATCTAGTGCCTTGAACATAAACGTTGTGTTTCACTGGAACATTCAATGGAAGAAGCAGCTTCATTTCATATCCTTGATATACCCACAAATCAGGTT[A/C]AAAAATCATGGAAGGCTCAGAAATGATCAAGGAATAGACGCCTGATGGAACAGATGCACAGATGCTGTGCTGGAAGAAAAGACAAATGCTGAAGTGCATA
X	11734519	SCAFFOLD130170_10110	0.33	0.26	CTAGCATCAGCATTGCTCAATGATTAAAGCCCCAGAGTGAAATCAGCCAATTGGAATGATACATCACAGAGTACATCACAGAGTTGTGTCACCCATCCTC[A/G]TTTTAGGATCAGAGGTATTTCACAGGTTTAATGTAGCAGATTAGCTTGTTTGGGGAAATAGTTGATGAAACTGCTCTTTGTTCAGCTGTTAAAAAAAAAA

X	12238724	SCAFFOLD15641_12871	0.34	0.39	ACATCTCCATCATCAGCAACTCTCATGGGACAAACCTTAGAGCACTTGGTCCATAAATATTTCTTGAAGATCCACTAGGGGTGCACACTGGCACTTTATT[A/T]AACACCAAGTATCTCGTGTGCATGCTAAGTCACTTCAGTCCTGTTTGACTCTTTGCGATCCTATGGACTGTAGCCTGCCAGGCTCCTCTGTCCATGGGGA
X	21834369	BES11_Contig506_3137	0.33	0.34	GCCAATGAGTGGCAATGGTGGGATTTGAGATTAAGAGCTCAAAGGAGAGACAAAAAGAGTGGAAGAACAGCTTGGATGTATCTGGCTACTCTGGCCTCCA[A/T]CATCAGAGAAATGTATGGACTAGCTTAGATGGGCTTGTGTTTCTTGCCCATCCCAGCTTCAAGGACCTGCCAACACTCCCCCACTCTTTACCGCCTCCCC
X	28161885	SCAFFOLD180540_8495	0.27	0.21	CATCTTAATCTGTGTTGTTTCACTAAAGTATCAAACCAAAAGATATTGTAACTATAGTTTTTACTTTTTTACTCTTGGTAGGAAATTTTTTGATCCATGC[A/G]CACTTTTAATTGCCAGATCTATTTTGGCTTTACCTTTAAAGAAAAATCACTGCACTTTGCTGTATTTGTCAAAAATGACAGGCTAATTCTTTGACTCAAA
X	38492716	SCAFFOLD147422_737	0.33	0.48	TATTAAAAGGAGACCAAAAATTTGCAGTTTGATAGTAGAGACCAGGCAAGGATGAGGTTTGACAAGTAAACCAAGAATGTGGATTTTAGTTTCTGTTGGG[A/G]TTTACATAAGGGAAGTGAGACTGGAGGGTTATCTTTGGCTCTTGAGTCCAGTCTACTGAGCATATATTACTGTCACCTCAAACTCAGTGTATTAAGTCAT
X	46531887	SCAFFOLD4869_4182	0.13	0.12	TCTTATCCTTGCTGGAATCCCTTTGCTCTTTCTTGAACATCCTTTGATGCATCAGAGGGACGAACAAGAGGATTACTGCCCCAACGATTGGGGGTACACC[A/G]GCAAAGTAGAAGGCCACATGATAGTCCCCAAAACAGTTGCGGAGGAGGCCTGAAAAGTTAACAGAGACAAGTCAAGTTTAGCTTTGCTCTACTATACAAA
X	46532144	SCAFFOLD4869_4439	0.13	0.13	TAGAAGTTAACTGTAGCTTATGAAAGTAAAAAATGGAGAACTTTGAGAAGTTTCATTTGCCTCTTTCATAATAAGCATTTGCCTCAAGAGCCTACAGTCT[C/G]AGTGGCTCAGGGAGGAGCTGCTTGTCTTTGTAATAGAGGTTTTGTGTCTTCCATCAAGGAGTGTTTAAACTGAGTTGGGCTCCCAGGTCAAGGCAGTACA
X	47330491	SCAFFOLD318366_7525	0.08	0.13	TGTCAGATATTAAATTTTATTCTCTCAGGATGCAAAATTACAATAAAGTGCAAATCTCTGTTAATGAATTGTGTATTCTTTGGTGCATCTTCCTTCTTCC[A/G]TCCCTGCTTTCATCATTATAAGGTAACTAAGGATTCCTTTTAAAATGTTTGTCTTGCATATTGCCCCCTCCCACATTTGCTAGTGTTATGTCTAATACTG
X	47330745	SCAFFOLD318366_7271	0.08	0.14	CTAGGATTTCTACTTTGGGGCAGAGGATTCCTGTTGAGACAGTTTTACCAGAAAAGGGTAAAAGTGAATATGTCCATTTAAGCCAAGAACTTTCATGCAT[A/C]TTGTATTGTTATATAGCCTGTGGAATGAATCATTTTTTTCTCCCTTGAGTTCCGGAGTGACTCTCCTGGAAAAACTCAACCTATATACCTGTGCCAAAAG
X	48746914	SCAFFOLD15133_2787	0.15	0.33	CTGTCATTAAGAACCTGTCGTTAAGATTCTAAAGAAAGACTTCTCAAAAAAATACAAAAAGTAGACAGAAAGAGGGAGGATCCAATACGTAGGTGTGGAA[A/G]GAAAATTCTAAATGTGGAAAAAGAGTCACAGGGGAAGATGAAAAAGGATATCTGAGAGACTACAAAAGTAGCCACAAAAGGGTAGGAACTGGTAGAAAAC
X	48749434	SCAFFOLD15133_267	0.15	0.33	GATTTCCAGATGAAGATTGATGATGAGGTGAGATTTTAATCTTTATTAGTACATCCCATTTATCCTTAAAAAAAAACAGGCTTATTGAGATGATTGACAT[A/T]CAGTAAATGGCACGTGTTTAAAGTGTACTATTTGGTGCGTTTTGATGCATGTTTATACCTGTGAAATTGTCATGACAAGATAATGAGTGTATCTGTCACC
X	55259065	SCAFFOLD155018_9068	0.11	0.27	CCAAAATCACATCCAACAGTGACATCAAAGTTCACCTTAACCCGCCCCAGTACACCCCAAGCTGAAGCCCTGCCCTAGCTCCTGACTCACCACTGGACAT[A/G]TCAACCTTTCCCCCTCCATCTCTGCTGCAGGCACTGGGCATCTCTGAGCAATTTAGTGGCGTCTCTACTCAATTCAATGAAATCCATTGCATCCTTGCTG

X	61365643	SCAFFOLD110371_6693	0.16	0.17	CTTGTGCCTGGAGACTCAGAAATTGAGTTTCAAAGCCTCAGCAATTCAGAGGTCCTCTGCTCCCTTTTGCCACTCAAGCAGGTTCCTTCCCCTTTTCACC[A/G]TTTCAAGTGACCATAGATTTGTAAAAGGTGATATACCCATAGTATCTGACGCAAGACGTCATTCTTAGACATTTTGGAACACTTACCACTGTTTTGGGCT
X	64139050	SCAFFOLD129123_765	0.19	0.13	CAAAAGGCATTAAAGATTAATCAGCAAAAGGCACTCTTCTGGTTTTCATATCAGCTGAAGACACAGGTAAAAGTTGATTGAGAACTTATCTGACTTGGGT[A/G]CATTCCTCATGAGTGGGCTAATGCTCAGTAAGACCAAAAGGGGAGGGTGTAGGAGTGCAGACCCAAGATGGAACTGGCAAAACAGAGTCAGCATGGTTTA
X	68962673	BES2_Contig513_1168	0.30	0.16	GGCATTCCCATCTTGAATTTAGAAGATTCATTTGTTCTTGCACTTCAGTTTCTTCATCTTCTGATAATTTCCCTGACCCAATCAGCTGACTCCCCAATTG[A/G]AGAACATTGCCAACCCGTCCCTGATGGGATGTCAAATCCATCATGTATCCCTGACAAAGAAAGAAGGTGATAAGAGTCAACATATTATGGGAATCATTAT
X	69423521	SCAFFOLD281693_10844	0.44	0.39	TGATTCCCTTTGATAACCTTTCTCCTCAGGCACTGTGAAGACAAATAATAATTAGTAGTTCAGCTAGGATTTGGACCAGAAATTTCAGCTTCTTTTGTAA[A/G]AGACACTCCACATTCACTAAAGCTCTGACAGTTGCAAATACTCCCTGTGGCCAATCCTGCTTATTTTCCCCTTTGGAATGAGTCTGTTTTAACTGAGCCC
X	80441126	SCAFFOLD305897_2469	0.42	0.24	AAAGGCAGCTGGAGGTTGTGTGGACATAAAGGAAATGCCATTTGTAAAATCCCTGCATTAGCTGGGCCCTTCTGGGATGTGAGGATGCTTAGATTTCAGA[C/G]GTAAATGTCTGTCTTTGCAAGGAAATGCACATTTATGTCATGTCAGAAAGCCCCTCAACAGTAGGTGACTGAGGATGCCTTCCAGTAATCGTCTTTAGAG
X	84409286	SCAFFOLD300450_30284	0.49	0.00	GCACATCCAATGTTATGCTTTAAGATATTTTCTGAAGCAGTCAGTGTGTGTGATTAATATTTATTTAGTTTTCTACATAGACTACTAAAATCTCAGCATA[A/G]CAAACTGATATATTGAGCTAACACATCAATAATAAATGAATGAGATATGATAATGAATTCGATCTGTAGTAAATAAGAAAAAATCTATGTTGTATTTGAG
X	85510888	SCAFFOLD285727_12117	0.36	0.49	TTGTTTTTTTTTTTTTCATTTAATAGACCGTGGATGTCTTTCCAGGTCAAAAAATACCTACTACCGGATATTTAATTCTTCCACTATACCTGTGTCTTAC[A/C]ACTGAACCCACCCTCTGGTTTTGGCCACTTAACTTGCTTCTGGTGCTTTGAGGTCATAAATAATATTGCAGCAGTTTTTCCTTTCACAATTCTTTCCATT
X	85532795	SCAFFOLD145281_2616	0.37	0.46	TGATTCCACTCCTAGGTGTATACCCTAAAGACCTGAAAACAGGTGTTCAAATATTTGCACACAGATGGGTTCACAGCAGCCTTATTCAAACAGGTGAAAG[A/G]TGGAAGGAGTCCAAACCTCCATGGACGGATGAGTAGATAAAAAAAATGCCCTGTACTCATACAAGGGATGAGAAGGAGGGAAGTGCTGACAGACAACGAC
X	86199840	SCAFFOLD70166_10737	0.43	0.49	GGAAGGTTCGTAGGTGACCCTTAAGGGGCGTGATGCCGGTTTGAGAGGAGAACACAGTTCTTCACAGAGCCTTGCACACCCATTGCAGGTGCCATTCCCA[A/C]GCCCTCCTGCTATGCTAGTATCTGTTTTAAAAAATTAAAAAGAATGTCTGTAGATCCACTTAAAGGCAGTCTTTCATGCAGAAAATACTCTCAATTGAAC
0	0	BTCN14808	0.48	0.06	TTTTCCCCTGCAAACCATGAGTATTTCCCCAAGAACAA[A/G]GAGAACTGTTTCAGTTGTCCAGGTAGCTGTTCCAAGTACCGAGGCTTCAGCTTTT
0	0	SCAFFOLD135102_4467	0.18	0.06	TGGGTAAAAAATGATAAGTTCCTAATCAGTCTTGGTCCATTCATTTTGGCTTTTGGTTGGAAGAGACTCTAACTTAAACAAGAAAGAAATTTATCAAAGG[A/G]ATGTGGGGGAGGTCACAAAATTGGGTTGGAAGCTGGAGTTTTCACAAGGACAGGAAGCAGGACAGATGCATAAGCACCAAGAGCCAATAGACGGATTTTG

0	0	AJ580801-115-T	0.02	0.07	CTCATCCTCACAGGTTATGTCTGTGCTCTCAGCCTCAGAGAATTGAGCACTGCTGCATTGGTCACTAATACAAATAGAACCCTTTTCAAGATCTGGAGTC[A/G]TCAAAAGTAGAGCATCTGTAGTTGCTGGCACCATCTCATCTTTATTTTTCCATGATGTATCAACACTGATATCTTCTGAAATGGTTTCAGGCTCCAAAAG
0	0	LR20_149F1-SNP1	0.02	0.07	CTCATCCTCACAGGTTATGTCTGTGCTCTCAGCCTCAGAGAATTGAGCACTGCTGCATTGGTCACTAATACAAATAGAACCCTTTTCAAGATCTGGAGTC[A/G]TCAAAAGTAGAGCATCTGTAGTTGCTGGCACCATCTCATCTTTATTTTTCCATGATGTATCAACACTGATATCTTCTGAAGGATGTATACG
0	0	BES10_Contig414_595	0.04	0.07	AGAGTTCTGGATGTCAATGACAATCCACCCGAGCTTGCCAGGGAATATGATATTGTTGTCTGTGAAAATTCTAAGCCTGGTCAGGTAAGTTAATTGTTTC[A/C]TTCCTATAATTTGAATGGAAGAAAAATAAGCAGCTCTGTTTCTATTATTCTTAGGTATAATCCTCAAAAATCCCTCAGTGTTATTTTTGTGCTTAATACA
0	0	G73121ML2	0.16	0.08	AGCCGGCCCTGAGGAAGAGCTGCTGCCAGGGGTGGACATACTCCTGGAGGTTTTCCCCACCTGTTCAGTGGAGCAGGCCCAGTGGGTATTGGCCAAAGCT[C/G]GAGGGGACTTGGAAGAAGCTGTGCAGATGCTGGTAGAGGGGAAGCAGGGGCCTCCAGCCTGGTATGGCCCCAACCAGGACCTGCCTAGGCT
0	0	SCAFFOLD135102_4826	0.26	0.09	CTCCCCCACAGACAAACTGGTTCTGTTACTGGGCTCCACCAGCACAAATGCTGAGGGAAAGACACCAAATATGTCCCATCTATTTGATGACCACTCATTC[A/G]CGCCTTCTCCCCTCTTTCCCATGTGCCTATGCATACCTAGACAGGAGAGAGACCAGCCAACCCAGCCCTGTGGTGGGTGAATGGCAAAGGGGATGAGGTG
0	0	SCAFFOLD318094_6588	0.10	0.13	TATAGGTGCTATATTCCATTATAGCAAAACTATATTCCATTATAGTGACGTCTTACACAACCAAAACCTGGGAATGCACACTGCAGATGACCAACTTGGG[A/G]GCTTGAAAAAGAAAGTGTAACTCCAGTAAAACAGGGTATTTGCCATCTCTCTTAATATTCAATCTCCTGTTGGTTAGATTTTGAGAAAAAAATAAACAAA
0	0	SCAFFOLD318094_6639	0.08	0.13	GAGATGGCAAATACCCTGTTTTACTGGAGTTACACTTTCTTTTTCAAGCTCCCAAGTTGGTCATCTGCAGTGTGCATTCCCAGGTTTTGGTTGTGTAAGA[A/C]GTCACTATAATGGAATATAGTTTTGCTATAATGGAATATAGCACCTATAATGGAAGGTGTGGTAGTGGCCCTTATGTACTGAATATTAGATATTGAAGTG
0	0	SCAFFOLD110576_2863	0.42	0.14	CTAGGCTGTCTTAACATTACTGTTTTAATCACAGATCAGATATAAAGGACAATATGAATTACAACCTCCAACTAAAATCCTGTGTAGCCTAGACAGTGAA[A/G]TGATACAACATTAGAAGACTTTAAAACTGCAGTTCTTTCTGGATCCCCCAAAGTGTATCTGCACTCTTCTTCAAACAGGCCTCTTCTTCATGAGTCAGAG
0	0	SCAFFOLD40955_7411	0.12	0.18	TAATGTTTGTCTCTCTCTAGATGAGTCCATAAGATCCATGATGTCAGGGATCATGCATATTTATTTCTCCTCTCATCTCACCATGTATCAAATTCCCTGG[A/C]AGTTATCAAGTACTTAATAATATTTGTCTGAATGAGTAAGTGGAATAGGTTTAATGGAATTTGATGAATGTGGGAATATGAGGATGTGATTTTGAGGATG
0	0	SCAFFOLD260546_17298	0.13	0.19	ACTGCCACCAAGTCAGGTTTAGAAATGGACTGCCCCAACAGTAAAGCGTTTAAATTTCTCTCCAAAAAAAGGCTCTTGGTTTTGAAACTGTACTTTCCCA[A/G]TAGTAATCTCTTGTGGAAGTTCTGTGGGACACAAGGCAGATAAAGTAAGCTTAACCTTGTTGCTGTCAGTGGGTTAAAAATACCTTTTGTTTTAAGCTTC
0	0	SCAFFOLD65831_1165	0.18	0.20	AAATTACATAGATATGAAAATAATTGATTCTCCTACCTACTAGCTAATTTTAGTTTTTGAACCTTGCTCTACATTACTGATTTAAATGGAAAATTTCCAT[A/T]ATGGAAATGTTCTTATTTTTTTGTGCCTTGCATGAGTGTCTTTTCCTTCTGAGTAATGAATGAGTTAAGTCACTCCTATGAATCTGCTTAAGTTTTTATG

0	0	BES10_Contig558_349	0.14	0.23	AAATTTAAATGAGTAAATAAATGCCTTGAAAGAAATTAAGAGAAATCATCTTAGGCCAAAAGCACACCTGGAACATAGATCTATGAAGGTACAGGATTCT[A/C]TAATCTGCAATATATCACAAGCTTATCTGTGAATAGAAGTAGCTTTAGGATTTTAGGGCACAGGGAAAGGATTAAGTTATTGAAAATCCAGCTTCCCTGG
0	0	SCAFFOLD265091_15493	0.32	0.23	AGGCGACCAGTTGACCGCCCATCACCAATCCTGGCACCAGCACATAAAGAAAGCAACGCCTTCTCTCTGTTTTCCCAAATGTCAGGCTCCAGAGTTTCCT[C/G]CCTCATGGTACCCGGCCCCCCACCCTACCCCTGCTCAGGGGAGGTATTCATCTCAGCTCAGCGTCAGGAGACATGGGGCAGGCGAGGGAAGTTGTCAGTG
0	0	BES11_Contig273_978	0.19	0.23	GCCCTGTACTTCATTCATTCTACAAATTCTTAAACTGAGTATCTACATACTCCGTCCTTTGCTCTTGGTGCCGAGGATCCTGAAGTGACCGAAGTCCTTG[A/C]GCTGTGGATATGCTCTTCAGGGACAGAAGAAAGGCTGAAGTTATAATTGGGCAAATCGTAAAGCCAAGTTGCCTTTACCCTCAGAGTTGTTCTGTTCTCT
0	0	BES4_Contig509_1194	0.36	0.23	CTTTACTCCTGAAGCCAAGTGAGTACAGATCTCCTAATTCCCAGTTGGTACAGAGGATCTGGGAAGCAGCCTGCAGACAAATTTGAACACATCTATCTCA[A/C]CTTTTTCATCAAGTTGCATTTTTGCTTCTCAAACACTGAGATGTATTCATGAACTCTTCTTGTGCAATGACACTGTTCATTCTGTTTATTGTTCTTTTCC
0	0	BES4_Contig509_1322	0.36	0.23	CAGGCTGCTTCCCAGATCCTCTGTACCAACTGGGAATTAGGAGATCTGTACTCACTTGGCTTCAGGAGTAAAGGAAACACCTACCCAAGGGCACAGAACC[A/G]TTTTCTGGTGCTGTCCTAAGCCTGCATACTGGGTCCTATACTCCCATTTTCTGACATCATTTGCCCATCTTCAGTTTAGTCACTCAGTCCTGTCTGACTC
0	0	BES2_Contig481_1550	0.16	0.24	TGTTTTTCCTTTACTTTCGTATATCTACCACACTCACAACACTTCTGACACCAGATATGTGGGTACTTTCCCCTACCCAAGAATTCTCTGCAACACTAGC[C/G]GGCGTCCTACAATTTAAACCAGGTCTGACTCTGTCTACCTGGAGACGGCATGGGATTCCCCAGGTTAAGTGCCCAGTCCTATAAAACTGGGAACATTTCA
0	0	SCAFFOLD139675_516	0.22	0.24	AAGTTAAAGAGAAATTATAAAATGCAAAAGTGAGCAATCAGCTACCAGAAAACACTTGAAGAAACTAAAACCATTAAAGAGACAAAAAGTTCCCCACTCT[A/G]AAGAACTAAATACTTGAAAAGGCAAAATAAAAGCAAACCAAAGCAGTCATTAGAATAAATACAAATATCTCCAGAGAAGTATGAAAGGGTTTACATTTAT
0	0	SCAFFOLD215815_8561	0.31	0.25	TTTAAATTTCAATTTTACTTTAAACTAGAACAGATATTCTTGACTCTTATGTTTGTGATGGAAATAGGAAGCATTTCTATTTCCCTGTCTCCTATCTCTC[A/G]TATGTAAGGTTTACTCAGTTGATTTTCCTATCTGCTTCATTCACATCTGTCTTGCTCTCTTATAGCCTCAAAATTTATTCTACTTACTGCCATCCCTATG
0	0	SCAFFOLD215815_8602	0.33	0.25	AGTAGATAAGAAAGAGAAATGAAGATTTAAATATTGGAAAACATAGGGATGGCAGTAAGTAGAATAAATTTTGAGGCTATAAGAGAGCAAGACAGATGTG[A/G]ATGAAGCAGATAGGAAAATCAACTGAGTAAACCTTACATACGAGAGATAGGAGACAGGGAAATAGAAATGCTTCCTATTTCCATCACAAACATAAGAGTC
0	0	SCAFFOLD69179_2924	0.36	0.27	AGGAGGAGGAGGGGAGGAGAGAGCTCCAGGCTCAGAGGAAGATAGGCAAGACCCTCATGTGTGAATATACCTGCAATGACCAAGAGACACAGTGAGGAAG[A/G]CAGCATGCTGATGTCCAGGGAGCAAGGAGAAGCATAATACCTGGTGAGATCATCATGGTGGGCAGGTCATCAAAGAAATTTAACATAGATGACGGTGGTT
0	0	E64H04-38783-1	0.35	0.27	AACCACTTTAAGCATACTAATGAATGTAAATGGACAGAATTGTTGGGGGCAATGTAGGTAGAASGTTATTGAACAGAAGTGTTTCAAGTGCTATGAAAAA[A/G]CAGTATATTTTAGCTGGGGAATGGTAA

0	0	SCAFFOLD195273_10714	0.18	0.27	GAGGCTCTGGCCCCAGGACAGGACTGACAGGTGGGCACGTGGACTAATTCTGGGGGTCAGCACTGAGGACCCTGGTTCCCCGGGAGGCATCCTGTTCCCA[A/G]CCTTTATCAGAAGGAAGATTACTTTTCACCTCACTTCCCCCTGACTTCCCCATCATCATCGCTCTCAAACGGCCCCTAACTCAGGTGGAAGGGGATGTTG
0	0	SCAFFOLD106077_1598	0.38	0.33	GACTTAGCAACTAAACAACAATAGTGTTTATCATCTGTTATATGGCATACTAATTGTTTTCTTCTGTTTTGTCATTCCTCATTAAATATAGGCTCTGTGA[A/G]TATAAGCATGTTTGTCTGCTTCTTTTTTCATTTACCTAACACCAAACAATACCTGATATATAGTAGTAGCTCAAATAAATATTTGTTAAATGAAGAGATC
0	0	SCAFFOLD115093_14103	0.23	0.35	AAATGTTTGTTTTTTTTTTATTTTAAAAAGACCAAAAACACTTCTGGGACAGATGTATTCTGGAATTTGGGGTTTTTTTGGATTTTAGGAGGATCTCATG[A/T]GGCACTTTTTAATCAAACACACTAGAATTTCTGCAGCGAACTGTGAATACTCACACTAAGTGAGATAAAGCTGACAGTAACCTCACGTTTGTTCAGGGAA
0	0	SCAFFOLD230210_3350	0.28	0.35	AACAGGTGTGGGCATTGTATAAAAAACAAAACACCCCACAGAACCTGGGGAGACATTGGGCATGGTGTGTGTGTGGGTAGGGGGCACTTTGGACAAGTAG[A/C]CCTGACCTGGCGCAGGAGGGTTGAGAGAAGCCTTGGCTCCTGATAACATAAGTTTCTCTTCAAAGAGCCTTTATGTGATTTGTGCATGAGAAATGCAACA
0	0	CC517527-273	0.47	0.35	GTTATAGGTTGGGGAACAAAGTTGTTGAGCAAAAGACACTAAGTACGCTGTCTTCACTTCATTTCTCAAGCTTAGTTACCAAAGGCAAAGTCTTTATAAT[A/G]ATGTTGCACATTATGTGGGTGATAGCTAAAGCCGCCCTTTGGTCCACTGGACCGGAAAATATTTTTAAAAGAAATGGCACCTCACCCCATGGCTAACTTT
0	0	SCAFFOLD105166_16070	0.30	0.36	TGCCCCTGCCCTCCAGTAACGAACATTCCTTCAGCTGGAGGGAGAAAGTGATGGGGGACCCCAGGGAAGGTAAAGAGAAATGGCAGGAAAGTATTAAAGA[C/G]AGGCAGCAGGACTTTTGGAAGACCATCTAGATACTTTCCACTGTAAAATAAGCAGGACTGGGTAGAGGTTAAGCCATGGATTTTGGAGTCAGGTGTGAGT
0	0	SCAFFOLD153002_7972	0.21	0.37	TGAGATGATTGTGGTAGAGTGGAAGAGCACGTGACATTTGCTGAGGTCATTGAGGTGAGTTATTAATTTTCTTAAACACTAGTTTTCTCATTTTGAAAGG[A/G]GTCCTAAGATCTCCCTCTTTCATAGGGTATTGTGGGATACTGATAAGATAACACACATAAAAGCATTGTATAAATTGAGAAAGTATACGTGAATTTAATG
0	0	AB070368-1759-UASMS3	0.37	0.37	TGTTGTAAAGTAATATTTAATGATGTAGGAAAATAACTTTATTTTGTGAGCTGGAAAGAACGGATTATAAAATGGTATGTGTTTTCTGATCACACACATT[C/G]CAATCAATACACACACTCTCACACCAAAATATACATTATCACTATTGGGAGTAGGATCAGGAATCTTTAAGCTCTTCTTTGTGCTTTTCTGCTTTTCATA
0	0	SCAFFOLD45155_17934	0.35	0.38	GGTGCCTAGCACAGGATGAGGCAAATGGTAGGAATTGAAAAAGAATTGTGTTGAGCTGAATTGATTCACATAACTCATTTCCTGGGAGTCTGGCTTAAAG[A/G]TTACATTATGCTCCACAGATATTTTCTTTAATGTGTTCTTCCATGTTATGACATTGCAAAACAGTTTGCATTCATTGTCTAGAATATTTAACACAAGAGT
0	0	SCAFFOLD136321_685	0.42	0.38	CCTGCCTTTGCACACCTACCCTCTGACTGGCCAGGTCCAATCAGGCCCGGTGGATTCAGCCAATCAGCACTGAGCAGGATTTGAGTTTCACTTTTCTAGC[A/G]CTCTGGCAGAGCAACAAGGCCTTCCACTTGTCAAATCCAGTGAAACCCTTTCAGAATTTAATCATACTGGATTTCTCTGGACCTTCTGATATTGTTAACA
0	0	SCAFFOLD121099_3123	0.46	0.38	AAAAAAACCTGCACCCACTTGTTCAAGTTGGTGCATAATTTCTAGGGCTGTGAACTCCTGGAAGCTCGCATGTATTGGAACATGTGACTGAGGCATTCTG[A/G]CTCATTATGTTTTCATTTGATTTTGTTTGGTTAAATTAATATATGCGACTAGTTTTAAAAGTTCAAAGATAAATGGCTGGCCCTGCTTCACCCCCACTTC

0	0	SCAFFOLD136321_1198	0.43	0.38	AAGACAGAACTCATCCCCTTTACTCCTTAATTTCACTCCACTTCCTATGCAGCCAAGCTACCCTTCATCCTGCATTCCCTGCTTTAGTAAAGGCACCACA[A/G]TTCATCATGAGTGCAAAACAGGAAACTGAGTCTCACTAAAAGCTATGTTCGATCAACTCAACCCCCAAATAACCCTCCATCTCCTCTCCCACACTTGTTT
0	0	SCAFFOLD136321_1119	0.44	0.39	CCTGAATTCTAGTCCTTTGTATCCAATGCCTATACTGAAAAATTCTATCTAGGTATTCATGAATAGCTCCAGGATGTCAAAGACAGAACTCATCCCCTTT[A/G]CTCCTTAATTTCACTCCACTTCCTATGCAGCCAAGCTACCCTTCATCCTGCATTCCCTGCTTTAGTAAAGGCACCACAGTTCATCATGAGTGCAAAACAG
0	0	SCAFFOLD10982_20909	0.50	0.39	CGACTGGTAAACACTGAAAAAAGTCAGCTGTAGAGCAGGATGTATATTATAACCGCTTTTTAACATACAGAAAGATAATCATTTGATCTGCAAGGGAAA[A/T]ACCCATACTCATACATAGATACACACACACACAAACACTGGTCTGGAAAACAAACCAAACTATTAACCTACACTTACGACCTTATAAAGTGTAAGCTGGG
0	0	BES4_Contig253_834	0.50	0.40	GCCGGCATAAGAAAACTCACTGTAGACTCTAGATGACACAGGATTATTTGTTGAGTGTTTACTGCTGAATTGCTTGCTACCTATTGTGAGCTTCCTCTT[C/G]ACTTCTTTGCAAACAGCAGGATTCCTCTCATCTCTCTAGTTTCAGATTTCACCAGAAATTCAGGAGCTGAGTCAGGACTACAAGTGGCCCCATCTGCCGA
0	0	G73114-264-R	0.47	0.40	CCCACTGCAGTATGCCCTGMCCCGTGGCTTCCTGCRACCTCAACAGCTAACCTGGCAAAAGCGTGGATCCTCCCAGCGGGATGTCACTCTCCTGGGATTC[A/G]GGAGTCCATAATTCCAGAGCCCTAAGGTAATGTCCACTCTCTTTAAGCCCCCAGTGGAGGGAGTGCCCAAGCAAGAGGGACAGACATGGCKTCTGACCCG
0	0	AJ496772-060.SP6-52	0.30	0.40	GGTTTTGTGGTTAAGAAACCTGCTCTCTTG[A/G]GTAATCATTACCTTATAGGGGCCTCCTATATATTTTTTACTTACAATCTCTTAGTGGGATTAATTATTTCATCACCTATTTTGTCCCTTTGGGCTTCAC
0	0	SCAFFOLD220897_2677	0.48	0.42	GGGCCTAACAGGTGGGTGCACATGGTGGGCATCTATGAGTGCCTCAGGAACGGTGTACCACGAACTGAGCAGCCCGACGACCAGCCGTGTACTGTCTCGG[A/G]GTTCTGGAGGCAGCAGGTCCAAAGTCAAGATGCTGGCAGGACCATGCTTCTTCTGAAGGCCATAGGGAAGGATTTGTTTGAGGCCACTACTAGCTACTAG
0	0	BES1_Contig454_797	0.44	0.42	ATGTTAAGATTCACTAGCCCGGATGTCTTGGAATCTCAAACTCTCCGTCTCTATTTTAAATTTTTATTGTACATTTCAAATTATCTTCTAGCACCACAGT[C/G]GCAAATAGATGGCCACATATCTTGAAAAAATTACAAATAATATAAGGGGAGGGCCACTAGGTCAAAAGATATTTTTCTGGAGATTCTTGCATTATACCAA
0	0	CART-SNP	0.45	0.42	AAAGGGGGCTGTCTTCCTGCTGTTNCCAAATAAAAAGAACACATTAGATGTTACTGTGTGAAGGGTAATGCCTTGTATGGTGTGGCTCTGTGTACAAAGT[A/G]TTCTATTCTTATTTTCTCTGTCTGACAAACTTTTAAAAGTGAATATTTAAGCAAAGAGGAAGAACTTCACTTTGAAAATTGTATTTTTGTAGGTGGCATG
0	0	SCAFFOLD306076_12244	0.41	0.43	TGTTTTGAATGCTGAATGTTTTATTTTTTCCTTCTGTTTCTAGAGCTGCCTCACCAAGCCCTCAGTCGGTAAGGAGGGTCTCATCTTCCCGATCTGTCTC[A/G]GGATCTCCTGAGCCAGCAGCTAAAAAGCCCCCAGCACCTCCGTCACCTGTCCAGTCTCAGTCACCCTCCACCAACTGGTCACCAGCGGTACCGGTTAAAA
0	0	AJ496767-048.SP6-638	0.41	0.44	AGCAAAGAATCTAAATAAGCGGTTCAC[A/G]AAAAAAGAAGAACCAATAGCCACGTGAAAAATAYGCAGAGATGGCCACTCTAACCAGTAGCCACTAACCCAAATAAACACATTACCTAACAAAAAAGAAA

0	0	SCAFFOLD90920_8323	0.46	0.44	CTACAAGCAATGCCCTGTCATGTAGAGCCCAGGTTTTATGTCTGGCTCCCACCAAGCATCTCAGACACAACCCCTGCCCGTCCTGGGAGGGAGGAGTCAA[A/C]AGAGGGCCATGCGGAGGTGGTAAGGTACCAATGGACCCTGCTGACTGAGAGGCGGAGGGCTTGCCAAGGGATGGGGGCTTGAGCCAAGGTTCCTGTAACC
0	0	IGF1-AF017143-198	0.27	0.45	ACATTGAAATAGTGATGCTGTCCTCTGATTAACCTTCTACCGGGTGCGAAGACACACACATCTGNTNATACACCTTACCCGTATGAAAGGAATATTCGT[A/G]GAGGATGGCGAGCAATTTTATATTCCAGAGCCAACACAAAATTTAAAAAACAAGAAATGAAAAAAAGACCATCTCAAGCCTACTCTATGATTTTAACCCT
0	0	AF017143-198	0.29	0.46	GACATTGAAATAGTGATGCTGTCCTCTGATTAACCTTCTACCGGGTGCGAAGACACACACATCTGNTNATACACCTTACCCGTATGAAAGGAATATTCGT[A/G]GAGGATGGCGAGCAATTTTATATTCCAGAGCCAACACAAAATTTAAAAAACAAGAAATGAAAAAAAGACCATCTCAAGCCTACTCTATGATTTTAACCCT
0	0	CC474638-434	0.44	0.46	CGTTTTGGTGAGCAAGTGCAGAAAGAGAATTAGCATAATGGCAAGCGAGGACTTCGAGAGGTCTACGTGGGATCAGAATTTATAAGGTGGATGTTTTCAG[A/G]TTCATGTATTGCTGAGAGTGAGCTACATACCAACCCCTATTAAAATNCCTGATTCCCACCGTTAAACCCCCCAAAAAATCCCCCCCCGCCCATGATTATC
0	0	SCAFFOLD155476_15944	0.38	0.46	CTCTTCTTTTTAACATCAAATCTTAAATTATTTTTTAATCTTTTATTATGCTTCATATTTGAATGATAAGACACCTGTAATTTTAATTTCAGGACTATTT[A/G]TTGGATACCAAGACTAAATTCTTCCATTGAGTGTTTCTCAATTATGAAGGCACAGACTAAGTACTTGGAAACATGGTTAAAAATGAAGATTCTCATTCCA
0	0	SCAFFOLD5952_3608	0.48	0.47	CCTTTATCTGAGACCTTTATTTCTCCCTGTGGCTTGGAGGTACTATCTAATGTCCCTTCACTTAAATCTGAAGGGACACCGTGAGTATTTCTTGTAGGGC[A/C]GGTCTGGTGGCAACAAACTCCTTCAACTATTGTTGATCTGGGAATGCTTTTGTTGTTGAGTTGCTCAGTCACGTCTGACTCTGTGACCCATACGCTGAAG
0	0	SCAFFOLD15114_27297	0.33	0.47	GTCCATAGGGTCGCAAACAGTTGGACACGACTGAAGTGACTTAGAACGCACATGAAAGTCAAGTGGATTTAAGAACAAGAGGGCTCTGGAGTGATGGTGA[A/G]GCAGGAAGGGAAAGACAGTAGATGAGTGTGAACTGCTGTTTTGAAAACTGAGCTCTGAAGGGTCGGACAGAAATAAGAAGTAGTTGATGAGGGAAGGGCC
0	0	SCAFFOLD121256_6611	0.47	0.47	TTGGGGTTCAGCGTTGGTGTCCAGGGTCTTGCTTGAATGCCCTCTTCTCCCCGCTTGTAACAGTTCTCATACTGACAACTTGAGCTTCCCCACTCAACTG[A/G]GTGTTTCTGAACCCTGAAATTCTTTGTGTAATTAGCCTCTGATTGCTAAAGATTATCCCATGTAGAGAATGGAACCAAAGCAGTTGTAAAATGAGAGCTG
0	0	BV105448-337-Y	0.44	0.48	CTTTCCTATGAACTTTGTGATGCTGAATAAGGCATGC[A/G]GTTTGACTAAAAGCTTTTCCACTTTCACTAGATTCAYGCGGTTTCTCTGTGTGAATTCTTTGATGCTGAATAAGATGAGAATTCAACCTGAAGTCTTTTC
0	0	AJ496773-064.SP6-339	0.49	0.48	CCTTGTGCCTACATGCTATGTGCTATTCAATCTCTGTGAGCTCAGGAACATCAGCCAGGGAGCCGGGAGGAAAGGCCTTTCTAGGGCGGCCTCACTGGGC[A/G]GGGCCACTGGCAGCCCTCCATCGAGGCAGGCTGCTTCTCCCGGTATCAAACACAGCAGCACATCAGCCCACTGCTTCAGTGGGAGGCACGGGGGCACAGA
0	0	SCAFFOLD281506_2307	0.31	0.48	GTGTGTGCGTGCACGCTCAGTTGTGTCTGACACTTTTGCATTGTGGGAGTTTATCATAAACAAGAAGCCGGACTTCACTATTGGCTGGGGATGGACCCAG[A/G]AACAGAAAGAAGACAAAATTGTCTTAAGTGTTTAGTCCCTCAGTCGTGTCCAACTCTTTGCAATCCTTTGGACGATAGCCTGCCATCTGTCCATGGAATT

0	0	SCAFFOLD281506_2428	0.31	0.49	CATCTAGTGTTGTTACAAAACAGGACTAAAGGCATTCTGATGGGCTTATTAGGTGAACAAACATACCCTGTTTTCTGGCAAGAAAATGGTACCAAAAAAA[A/C]CCAGAGCTGCTGGGGGGTGTGTGTGTGCGTGCACGCTCAGTTGTGTCTGACACTTTTGCATTGTGGGAGTTTATCATAAACAAGAAGCCGGACTTCACTA
0	0	SCAFFOLD271090_5919	0.35	0.49	ACTGTCACACAGCTTTTTTCGGGTTAAACTTCCTTCTGATGGCTGCGGGACAAGGCACACTGACAGACACGTTCTTGGTGAAGTGCCGGTGAGTTACAAC[A/G]CTTCGGGCGTGCAGCCGAGTGACCCAGTTTACAGATACCTACTTTTTCAGATTCTTTTCCATTACAGGTCATTACAGGACGCTGAACACACTTGTTAAGA
0	0	SCAFFOLD66496_9339	0.44	0.50	CATGTATTTTTTCCCAATCTTCATCACTAAATGAATCACTATATTTAGAACAGGCACTACAGCTGTTTACTCACCACTGTACCCACTGCATTCTGATGCC[A/G]AGCACAACGCCTGGCACAAAACAGAACTGAAAATATTTACTTTAAAGAAAAAAGTGTGCAACATTTTTTTCTAAGACAAAGAAGCAGTAACTGTCATTTT
0	0	SCAFFOLD128106_6123	0.28	0.34	TGATGGCCAGAAGCCACATCAAGAAGATCTCGAAGACTGTCTTGCTGCCTCAGTTGCCACTGAGCTGTTCCAGGTGGTCATGGTTCTTGACCAGGAAAAC[A/T]GCTACTTTTTATACCAAAGAGTGCTCTGCCCATGTTTTCCTCTGGGAGTTCTATGGTTTCTGGTCTATATATGTCTTTAACCCATTTTATTTTTGTACGT
0	0	SCAFFOLD302326_838	0.39	0.50	CTTGGTTCCCCGACGAGGGACTGAACCCACACCCACAGCAGTGGATGCGCACAGTCCCAACCACTGGAGCACCAGGGCATTACCTATCACAGAATTTTAA[A/G]GGAAACGGACAGCCCGCGGCTTGAGAATCTATTAAGAGCGCCAAGTCTAACCACTGACGTGCTTACCTACCATGAGTTCATCTATAGTCGCTGCACCTTC
0	0	SCAFFOLD302326_754	0.46	0.38	TATCACAGAATTTTAAAGGAAACGGACAGCCCGCGGCTTGAGAATCTATTAAGAGCGCCAAGTCTAACCACTGACGTGCTTACCTACCATGAGTTCATCT[A/G]TAGTCGCTGCACCTTCCAGCCCCCCGGCTGCCCCGAGGCCATGGGTGAGGTGTTGAAAGGGCCTGACCCAAGGGCAGAAGAAGGCTTTCTGAGAAGCACC
0	0	SCAFFOLD164825_5856	0.36	0.36	GGGAGCATAGGAAGCCGTCATGTATCTAGTTCCCATTTCACAGAACTCTCCACCTTTGTCTGGTGACAACTTTCCAGCTTTCTTCACCTTTCTATTCCTA[A/C]CCAAAACCTCCCTCAGTTCTCCTCTTCTCCACCCCCTGACACAGAACTGTATCTCTCTTCCATGGGACTGAATTGCACTGTTTTGCAGTTATTTATGTGT
0	0	SCAFFOLD164825_5726	0.35	0.36	AGTTGTCACCAGACAAAGGTGGAGAGTTCTGTGAAATGGGAACTAGATACATGACGGCTTCCTATGCTCCCCCTGCTTCCACTCTGGCCCACAGTAAGCA[A/G]CCCAGCAGTCCCACAGCTGTCACCACGATCCTTTTAAAATCACAATCTGATTCCATCACTCCCTAACCTCAAACTTCCAACCACTTCCTATCACATGTAG
0	0	BES8_Contig500_1139	0.21	0.16	CTTCACAGGCTCTGAGGTCCAGCTTCATCCATGGTTCCAGTACAACACTGCCTTTACTCATTCCATTCTGGGACACCAAATAGGGTTTGGACTGGTAATT[A/T]CACTAGACCTGCTGCCTCTTGAATCTGTGTGAGAAAGTTGCCAACTCCAGGAAGGAAAAGACTCAATGAAATATCAGCAAGAGGTCCTCTTGCCATAACT
0	0	SCAFFOLD240996_1000	0.36	0.20	CCGCGGCGCCTTCCCTGCGGGGCCCTCTTTCTGTTGTGTGATTCGTTGTAGCTGCCCTGCTCAGAATTGCCTTTTTGAGAGCTGAAGCCAGGTGCGCCTC[A/G]CCACCTGCGGCCACATATCCCGTCCGGGGCGGCCTCTGCGCGGCCCCCGTTGTTCATATCGGCACATGCATCTCCCCGCCCCGTCGTTGTCTGCAGCTTC
0	0	BES1_Contig537_1232	0.10	0.10	GGACAGGTCTCCTACTGGAGGGCCCTGTGCCCCTGCCAGGCCAGCTTGATTTTGGCCCCACACATGGGGGGCATCTCCCTGGCAGTGTCATAGCCCAGGG[A/G]CCAGCTGTGAGCGTCTCTGTCCTCAAGGAAGCCACTCCTGCTCCTGCCTATGGGTACAGTGTGCGGCCTATTCTTTAGCTCTAACAATGTCACGGTGGCG

0	0	SCAFFOLD151267_4200	0.26	0.45	AATAAAAAACCATGTTTTTGAATGATCAGAGTTACAGACTCAAATGCTGACAAAGCCAGGCTGGTACTACTAATATAACAGCAAGGAGCATGGGGACTGG[A/C]AAATTTGAGAAGACTGTTTCCAAAGAAGGTGGTCAGTAGATAGCGCCAGCTCAGTGTTGCTGGGCAGGAATGTTGGGCCAGGACTGCCGTATTTTCCAGT
0	0	SCAFFOLD105315_6308	0.04	0.11	TCACTGGAAACCTTTCAGAAATTGGGTTTAGTGGAGTGGGAGGGGCTGCTCTCTCTGTCAGAGGAACTTTATGTGCCAGACTTGTTAAAATGAAGATTCT[A/T]GAATTTGCTTAGACTCTGCACACTGTAGTGTCTGAGACTGAGGCCTAGAGAATATGTATTTTTGACAACTTCCCCAAGTGATCCTTATATATATCAAAAC
0	0	SCAFFOLD141146_9111	0.47	0.45	GAACTTTCTGATACGCAGAATTCTGAGGAACACCACAATTTGGGGAAAACTTATTTCAAGGAAACAGGAGGTGACCACCTGTTCTTGAAAAAAATGAGAT[A/G]TGGAAAATGAAGTTTTCTATTCATTGCCATCTATAATTCCACCGAATCTATTTATTGAACACTTTCATTCTGAAAGGCTTCCCAGGTGGCGCAGTGGTAA
0	0	SCAFFOLD76639_3861	0.40	0.18	CCTGTAGCCTGTTTTGCTTAGATAACAGAGAATTTGAATTGCTATTTAAAAATCAGGAGAATTCACATAAACATGGATTTCTGTTTTTATTCATATAAAA[A/G]CTTGGATTTCTGTTCTCCATTAAAATAAAAGCCTTGCTTGTTCAGATTGTTAACTCTGATTCAGCCTCACAGGTGGTTTTCTTGCTTCCCTACATTCTCT
0	0	SCAFFOLD130131_22718	0.19	0.18	TCCTCCAGGGGATCTTCCTGACCCAGGGATGGAACCCTTGTATCTTACGTCTCCTGAATTGGCAGGCGGGTTCTTTACCGCTAGAGCCACCCAGGGCCCC[A/G]GGAGCATCAACTGCTTGTCTTTGACCCTTAACGTGCAAGAAAAAGAAAAGTCCCAGCTATGACTGTGACTCTGAACTGTAGGGGCACCATGGGGCCTTGC
0	0	SCAFFOLD120113_7960	0.31	0.29	AGGAGAAAATATTGCCAGCCTAGCATCACCCTGATACTAACATGTGACAAAGACATTACAAGAACAGAAAACTACAAGCCATTTTCTCTTATGTATACAG[A/G]TGTAAAAATCCTCACCAATGTATTAGCACCACACTATGGGGAAGACCATGTGGTTATCTCAATAGATGCAAAAATAGCACCTGGAAATACTCAGCTTTTC
0	0	SCAFFOLD11084_11208	0.45	0.03	AGGCTTAGCAGAAAGCAGAGTCCTGGAACAGTGAAACAGAAGGGAGACACGTGAAATGGTTGAGGAAAGATTCCAGGATATTCCCCAAGGGCTGGGTATC[A/G]GTTATCGATTGCTGAATAATGATAATTATTTCTTTAAAAAACAACATTCTCATCATCCAAAAACTATGAACATTAAATAATGGAGAGGATGTGGAGAAAG
0	0	SCAFFOLD120979_8524	0.22	0.42	CCCATTGCAGATTTCTAGGCTTCTCAGATTTACTGAATCTATTTCTATTGAGTTCCTCAAGAAGCACTTCATCTTTACACTGTGCAACAAAAACAATCTA[A/G]TAGCTCTGCTATATGACCTATTCCACCGGAATTTCACAGTCCCCTTTTTCTAACCAAATTCAACTACTGCAAGGAAATCAGGATGCAGGTCCAAGTCATT
0	0	SCAFFOLD316912_15690	0.46	0.50	AGACTGGCGGCCTGGCCGGAAGGCGAGACACCTTCTTCCGTTCTCAGCAGCCTGTGGAAGCCCCGCTGTGGGCAGACAAACCCTGAAACCCGGAAGCCAC[A/G]TGGGGTGCTCAGCAAAGTGGCCTGAGCAGCTGGGGAGGCCTGCAAGGAGGAGGGCCCGGGTCCCAGCAGGAGGCTGGGAGGAGGTGTCACCCCCAAGCTG
0	0	SCAFFOLD316912_15446	0.44	0.50	CTGGCCCAGAAGAGTCCTGGGAAATGCAGACAAGCTGGTCATCTCCGGCAGAGGCGGGGACCGTTCCCACTCCCTTCCCCAGAGCCCAGCCCCACCAGGC[A/G]GCTCCCCATGCGTTGGTCTTGCTCTTTCTACTTCCCTCCTTCTTCATCAGACTGGGGAGCAAAGCTGTCCACTGTGACCAACTCAGGTCCCCCCATCCCG
0	0	SCAFFOLD3177_2791	0.20	0.24	AAATCTCTAGCACATTTCTCCAAGTTATTTCCACAGGGGCATCCCATGCCTTTCAAACCCCAGTGATATTGTCTGAGAAATACACATCTCACTCACAGCT[A/C]TGGTCTGAGCTCTTTAAGTGTGTTTTCCCCAATTCATGATTTAGAATAAGTCCGGCATAGACCAAGCACTATTCACATCTTTTCAAGATTCTGTGTCCAC

0	0	SCAFFOLD181546_419	0.32	0.42	TGCTGCCTTTGCTGAAGCTCTCTCAGTTCACTCAGCCATCATCAGATTCTAATTCCTGGGATGCTTCTTCTGAGGAGCTGTTCCTGTGGATCCGGCCATC[A/G]CTCTTCATACTGTGGAAAGTCTTCTTGAAGGCCTCCATCATGGCGTGGGCCAGCTTCTTGGCCTCCAGCTTGCTCTCGCACTCGACAGCATGGCAGTCCA
0	0	SCAFFOLD55505_8699	0.24	0.25	TGGAACAAATTTTATGAAAGGGTTCATAAGATCAATGGGCTTTGTTGTTTTACCTGCAAAGGTGTGACATAATGTTAACTGATTATCAGCGTGCTCAGCA[A/G]GAGGAAAGGCCCTGAACTATATACCTGACTTCGCAAGTGTCTTGCCTCACCTGGTGGTGTTCTGGCATGAATATGAATAAAAACAAACACTTTAACACTT
0	0	SCAFFOLD131537_4695	0.22	0.22	TGAGTTTGGGAGAGAGGTTGCTGTCAAAGATACCAACTGAAATTATATCAGGGTAGTTGAAACCTTGAAAGTAGATGAGATAGTCAGATGCAGAGTGAAG[A/G]ATGCTCAAGAATAAAGGAAGGCTGAGGATGCAATTCTGGGGAAGACTAATGAATGAATGGGGAGAAAGGAGAAGCCAAAGGAGGAGGTCCAAAGGGAGGA
0	0	SCAFFOLD131537_5113	0.37	0.32	ATCCTGTCCCTTCTAGGTCAGCACTGTCCTTTGTGCCCATCCATTCCTCCCCATCCCCGACGCCCCTCATTGCATCTTCAGCTGCTGCCGGAATATCACC[A/G]CTTGGTTGTCTAAGAAACCATTCAAACCAATGAAGTTCAAAGCTGTTCTCAACTCCCCCTCAACCGTACACTCTCACCTGTGTTTCCCACCTCAGCTGCT
0	0	SCAFFOLD131537_8388	0.23	0.25	TCTGAAGACTTATTACCTAAATATAAATCTCACTACATGAAAGGAGAGTGATTGTTCTGAGGCATCAAAAACTGTCATTTTTGTTTAGTGAGAGATAGTG[A/C]GTCGTCGCGGTTAGGAAAGTAGACTATTCTGCCAGGCTGCCAGGTTCAAACCCCTTCTCTGCCACTCACTATGCTTGTGATTTGGGGCAAGTTCTAAAGT
0	0	SCAFFOLD160352_14290	0.24	0.40	AATATCCGTCGTGTGGATAGACTGTATTTTGCTTACCCATTCACCTGTCAGCTCACACTTAGGCTGCTTCCAAGTTTTAGCTCCTGTGAATAATGCCACC[A/G]TGAACCTTAGCATACTAACCGAATGCGTGTTTTTACCCAGTGGAGGTAACGCTGCAGATGTAATCACGTATCTTGCTTTTTTTCTCCTTAACATTCCATG
0	0	SCAFFOLD256192_8199	0.11	0.24	GAGCTGCTTCCTGGGCTCCGGACTGCGGTGTGAGCTCGCCGCCCCTAGGCTCAGGTCACAGAGACACTGAGCTGGACTCCCCTCGTGTTTCACAGCTGGC[A/G]GGGTCCACGGGGATGTGCGTGTTGGCGCCAGAGTGTCTTACACAAGCCCCTCTATGCGCAGGTGGAGTTGCTGGTAATGAAGGCCCTCTCGGTGGGGCTG
0	0	SCAFFOLD256192_8281	0.32	0.35	GCACATCCCCGTGGACCCTGCCAGCTGTGAAACACGAGGGGAGTCCAGCTCAGTGTCTCTGTGACCTGAGCCTAGGGGCGGCGAGCTCACACCGCAGTCC[A/G]GAGCCCAGGAAGCAGCTCCGCTGCAGCCCCGAGTCTAAGCCACATACCTCGTTCACAGTGAAGCTCTGGGCTGCAGCCCCGAGTCTAAGCCACATACCTC
0	0	SCAFFOLD1304_429	0.02	0.04	ATTTAAAAAATCAATCGGGTAAGAATTCAGTTGTGGACTGTAATTTTGTGGATTTAGGTTTTTTCTACAAGTAATTTCAAAATTAGACTGACATATAATT[C/G]GATAATAGGCCAGAGCTTTTGCTTTTGTCCCTTTAACCTTATGTGAACATATTCGCAGACATAGTGCTCAAAGGCCCTGTTGCTGATGATGGGTAAAAAT
0	0	BES4_Contig408_1085	0.26	0.42	ATGTTTTCTGAATAGCACTAAGTATACTGCTGTAATTTATGGTGAATCCTAGTGGTAACTTTCCCAAAGAAATCATATAAATTCACCAAAACATTAAAGA[A/G]CAACTTTATTTCTTTAAAGAAACAGAAAGGCACCAAATATATGTCAGTGATAAAATGTTTATGCTGATTAGCTTCTCCTTTAGCCTCATGAGCTAAATAC
0	0	BES4_Contig487_1232	0.47	0.48	CTTTTTAAGAAAGTGCATTCTGTTTTTACTTAATTGTTTGAAAATGGATCTAAATTGTATGTCCATTAACATGCTAATATCAAAAGTATTTTTCCTGACC[A/G]TTGTACAATGACACGAGTTCCTCAAATATCATCCAATTTACCTCTTGTGAGTAATGTCTTTAACTTTCATAACTAGAGCAGGAATATGTATTATTTAGAT

0	0	SCAFFOLD90277_18782	0.46	0.06	AAGGCCTTGAGCTTGAGAGAGTCATTCTATCCTAATTCACTTTCCATTTCCTAGTTGTTACTTTCATTACTCCTACACTTATTCATCATCAGGGTTGTTC[A/G]GAAGGTTACAAGTGAGTTAAAGAGCTAAGAGAGAGAGAAATCTCCTGTGATGATTAGACAAGCAAAGAGGGAAAAGAAGTTAGGAGAAATGGTGTGCTTT
0	0	SCAFFOLD10136_11539	0.49	0.42	CTTGTTTTGTGAAAGTCTAATTATGGGTGTTTTAGACAGATGGATATTGAGAGTTCATTAAACTTTTTCAATGTTTCTCTTTTCTTTAGCATAATCGCTA[C/G]TGCTATATTTTCAGGTTCAATTTTATTTGCTTCTATTGTGTTTAATTTGCTCATAATTCCATCCAGCATATGAGTCACTGGGGTGGTTGTGTTTTTTTTT
0	0	SCAFFOLD10136_12008	0.50	0.42	TGGCAGGGTCCGGCCGATGCCTCTGACCCCTGATCACCTTTAAGAGCCGAATGTGGTGGGCTGCCACCTGTGACTGCTGAACAGACCAGTTACATCACTC[A/G]GAGTTGACAAATGGAGGCTTTCTTGTTCTAACTTTACTTCTGAGCTAATGACTGGGATTTTTTAAAAAGAATTTTATCTCATCAATTTTGAACTGAATGT
0	0	SCAFFOLD85279_22429	0.40	0.33	AAAATGCAATTGTTTTTCAGAGAAAAGTATTTCCTAATAAAGTCCTTCCATAATTAATTCCCTCTGGCTAGTGCCCAAGGCCCTTCTGACACTTAGCAGA[A/G]CAAACAGCATTTATTGAGATCCCAGAATGCTTACTCTGAGATAGCTCTGTCCTTCCATACAATTAACAGCTCTGTGCAAGGGTAGTGAGCTAACTATCTG
0	0	SCAFFOLD125600_2589	0.39	0.38	TTCAGGAATAGATCTCAGTGGAGTAGAACAGCAGGGGTTCTGAAGTAAATAATAAGAAAGAAGAGTGGAGAGCAGGGTGGCCTCGCCCCAAAGGACAGCT[A/G]CCACATTCCACTCTTTGTTATAGACACAGAGTCATTCCTCCTCCATTCACCACCCACCCCGTGATCAGGTCACTATGTCTTCTGGGGGAATAGTCTCAGG
0	0	SCAFFOLD275079_22999	0.24	0.27	GGACTCGGCTTAAATCACTGAATTATAGTTTCTTGATCATTCTTCCTTTGTTTCTGCATTCTCTTACTTTCCTGATTGAATGTGCTATTTGGAACTCAGG[A/G]AAGGCCTAGGAGGCTGAAGCCCTTTTTCTGCAAATGAGAAATATGGGACAGGGAAAGGATTCCTACCCAGGAGGGCCCTCCAGGATCCCTCTGTTTCAGA
0	0	SCAFFOLD196839_438	0.50	0.00	TATTGCTGATATTCTTACTCAGGATGCAAAGAATTACCACGCCTGGCAACACCAGACAGTGGGTTATTCAGGAATTTAAACTTTTGGATAATGAGCTGCC[A/G]TATGTAGACCAACTTCTCAAAGAGCACATGAGAAATAACTCTGTCTGCAACCAAAGATATTTTGTAATTTTTAATACTGTGGGCTACAATGATCATGCTA
0	0	SCAFFOLD11377_3357	0.18	0.19	TTTGAAATAGCCTTTTGTTGCTGTTTCTTCTTTCTTTTGTCTTCTCTTGATGAAGGTATGCTCTGTGAAAGGTGGGGCCTTACTTGAGGAGGTTAAAACT[A/C]GTCAGTGTTAATTAAGCACTTTTCCTGGAAACTGTTGTAAAGTTGTCCTCCTAGAAAATATAAGCTTTTCTTGAAGCATTGTGAAAATCAGATGGATTGT
0	0	SCAFFOLD10775_4795	0.45	0.29	TGGGTCTGTGGAGCCCTCCAGTTGGCAGTTGGCGTCTGGGGCAAGAACCTGGAGCCAGATCTGGGCTGTCTGCCCCTTGAGGACCAAGAACACGAACCTG[A/G]TTTCCTGAAAACCACTCTGCTAGGTGACTCTGAACACAGCTGGCTTTCCTGAGGTTCCAGGATATGCTGGAGGGAGGGCATGGGGGCACCATTGTGGATT
0	0	SCAFFOLD10775_2055	0.45	0.29	AATGACTGATCTAAAAATTCTTCCTTTACTGATTTCCTTCTCTTCCAGGCCTTACCTCCCCACTTACCTACCAGTGAGTAGTTCCAAATAAACTATTTGC[A/G]CTTGAATCTTTGTTTCTAGGTCTCTTCTGGGGAAACTCAAGCTAAGACACAGACTGAGGTCCAGAGAAGACAAGGAACATGGCCAAGGTCACACTGCTGT
0	0	BES7_Contig507_2727	0.06	0.12	TTGAGGGATAGAGGGGGAAATGACTTCACAATTCACCCTGTGGCTTCCACTGTCAGGCACCTTAATAGGGGTGATGCCCTTTGCTGTTTATTCTTGCAGA[A/T]GATTCAGGGTAAAACAGAACAACTTATGTCTATCTTGCGTGACACATACGTGATCCTTTGCAGGACTAAAGAGAACTAGGAATTGCCAGCTGGCAACCCA

0	0	BES7_Contig363_1120	0.50	0.00	TGACTCCTTCTTCTGAAATATAGCCATAAAATAATGGCTCTGTAAACTGCTCTGGGCAATTATGGTGCTCCTGGAGTGAAAGTGGGCTGTCGTTAGCCCT[C/G]CTTATGGAGACCAGCTTGCCGCTGTTGGCTGTTAGCCAGGTCGCGAAACTGCCTTCCAGTGAGAGGACACAGGTTCAAGGGGATAATTAGGCAGAGTTTG
0	0	BES8_Contig212_428	0.35	0.34	TTTGGAATGCACATGTATACATGTGCCTGACATGCAGAGAAAAGATGAAGCAAGGTACAAGCATTGCCTAGGGATACACATAGACTCTTCTGAATTGAAC[A/G]GAAGGTCAGGAGGAGAATTACCTTTGTACAATTATCCTCCCTTATCAGTGGGGATTATGTTCCAAGACCCCAAAGGTACGCATGAAATTGCAGGTAGTACC
0	0	BES4_Contig133_337	0.29	0.31	GTGTTTGTGCCTGTAACATCCTTTAAAATTAAGAATAAAATTACATAGAATCCAAAATCATGCAAACTTTTATTTCTCTTTAAACAATTTACATGGACAC[A/G]GGAAGTGAACGTAACATAATTATTTTTTGGCTTTTTTTTATAACCTCAAGAGTTTGGCAGCTGAAGAATCTGAGCTGAGATTCCGACAATTAACAAAAGA
0	0	BES10_Contig697_766	0.48	0.36	AGGTTTACAGGGTACTATGGTCAGAACAGCAGTGGTATTTCCCTAGGAGGACAGGCTCTTCCTTGTCACTCACAAGCTTCCAACAAGCCTATAATAGGCT[A/C]CTCCCAAAACAACACTGAGTGACCTTGACCCGAAATCCCAACAGATGCATGGGTTTAATGCATACCTGGGCCAGGTGGCAACCATGACAAGGATCAGCGT
0	0	BES1_Contig527_922	0.49	0.38	CCCTATTTTGCCCCAAATTAAAACTGGGCCTGGGGTGAGGCGCTGACGCAGAGAGGAGGGACAGATGAGCAGCATAGCAAACATATCAGGTCAAAACACG[A/C]AACTATTCTCTTCCACGCCGAGAACACTGGGCATAGATGCTGCATGTCTTTGAGGAGCCAACTCATTACTTCTCTCTCATTGAACAGGTCTGCAGATACA
0	0	SCAFFOLD290938_3570	0.44	0.11	CTTTAGCTTTGCTCCATTTTGATCTTTGTAAATTATCTTAAAGATTATATGTACCTTCCTTTGGCTGTGTTAAGTGTGTTGAAATGACTTTTGGTCAAAT[A/G]TAATAAAATTCTACCTGATCGCAGCTTACTTATCTTGCCTGTCTAATCCATCTTGCACTTCCTGCCTACTCTACAGAAGGTGCCAGTGTGGTGATCTGTG
0	0	SCAFFOLD130087_22009	0.25	0.40	ATAGGCAGAAATTCTGGTTCTGTACTTGAGCTCCCTAGACTATCTAGATGAAGAGGCTCTTTTAACTGGTCTGGATTTTAATTCCACTGTCAGCAAAATG[A/C]GTATGTTCGTACAGCATTGGTACATTAAGGTCTCAAGGCAGACGCCGTAGACTTGTAAAATACTCCCTTAAAAGTTAACTGTCTTCAGAGTCTTCATAGT
0	0	BES2_Contig494_782	0.04	0.11	AACAGGTTCTTTGTATTTGGATATGATTTGGGTCTTTTAAATTTCCATAGAAAATGCTGGTACTTTTTTTTTAAGGCTCCCTTTTTAATACTATTTAAAT[A/G]ATTTTTCTTCCTACTTGGGTTCAGATAATAATTAAGGTCTCACTCTTAAATTTCAGGCAGTTCCTTTTAGTCTTGACAGCGTGGCAAATTCCATTCACTC
0	0	SCAFFOLD130277_4678	0.34	0.35	TCTCTCCCTTGATTAGCCTCCTTTATACTTCATCCTATCCATACACCTTTTCATTTTTTTAAAACTGGGTATGTCCTTTCATATTTGCCCTCATATCATA[A/T]GACAATGACAGTATTAACAACAATCAATGCTAATGGTTATTTAGAGCTTATTATGAGCCAAGCCACTAACTGCCTTCGTAAGTTAAGATACACAGAAAGC
0	0	SCAFFOLD130277_12275	0.49	0.43	ACCTATTTACTTACCATTCTGAATATGGGCATTAAGAATATGCTTCAAGTGTTCTATTGACCTAACAAAAAAACGTGCTTCCTTGCATAGAGGAAGCAAG[A/G]AGAGAGAAAGAAGAGGTATTATTCAATAGTTGCTTCAAATATAAATGGAGACTTCTTACATACATTTCTACATAAACCCAAGAATGTTACAAGAACATGG
