# Supplementary material for: A 2cM genome-wide scan of European Holstein cattle affected by classical BSE
Source: BMC Genet. 2010 Mar 29;11:20. doi: 10.1186/1471-2156-11-20 (PMC2853485; doi:10.1186/1471-2156-11-20)
Supplement: Additional file 4 — Graphical plots of the MAF versus position for each chromosome for the multiple family sample set and unrelated sample set. [file 1471-2156-11-20-S4.PDF]

Additional Figure 2: – Graphical plots of the MAF versus position for each chromosome for the multiple family sample set and unrelated sample set.

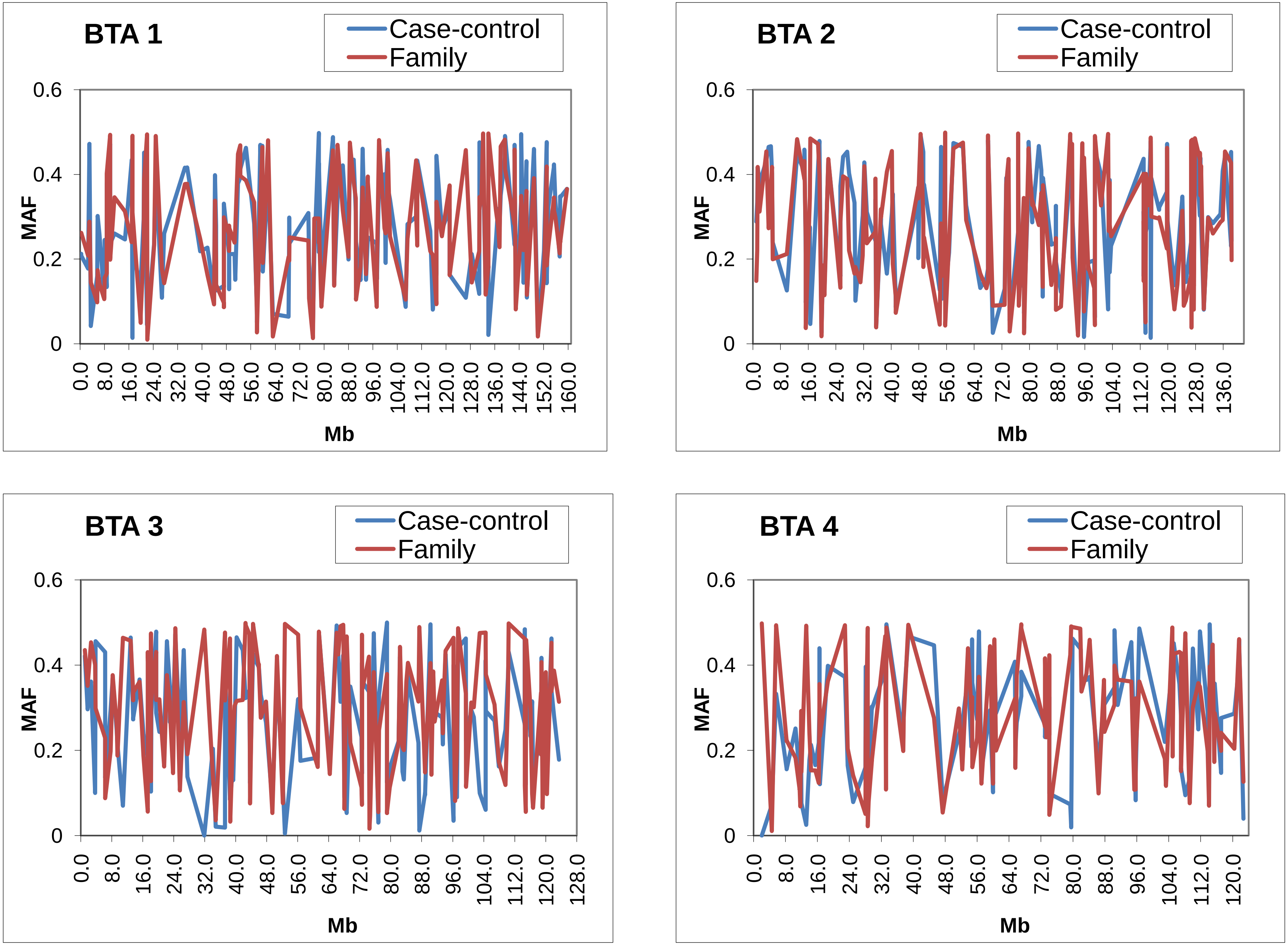

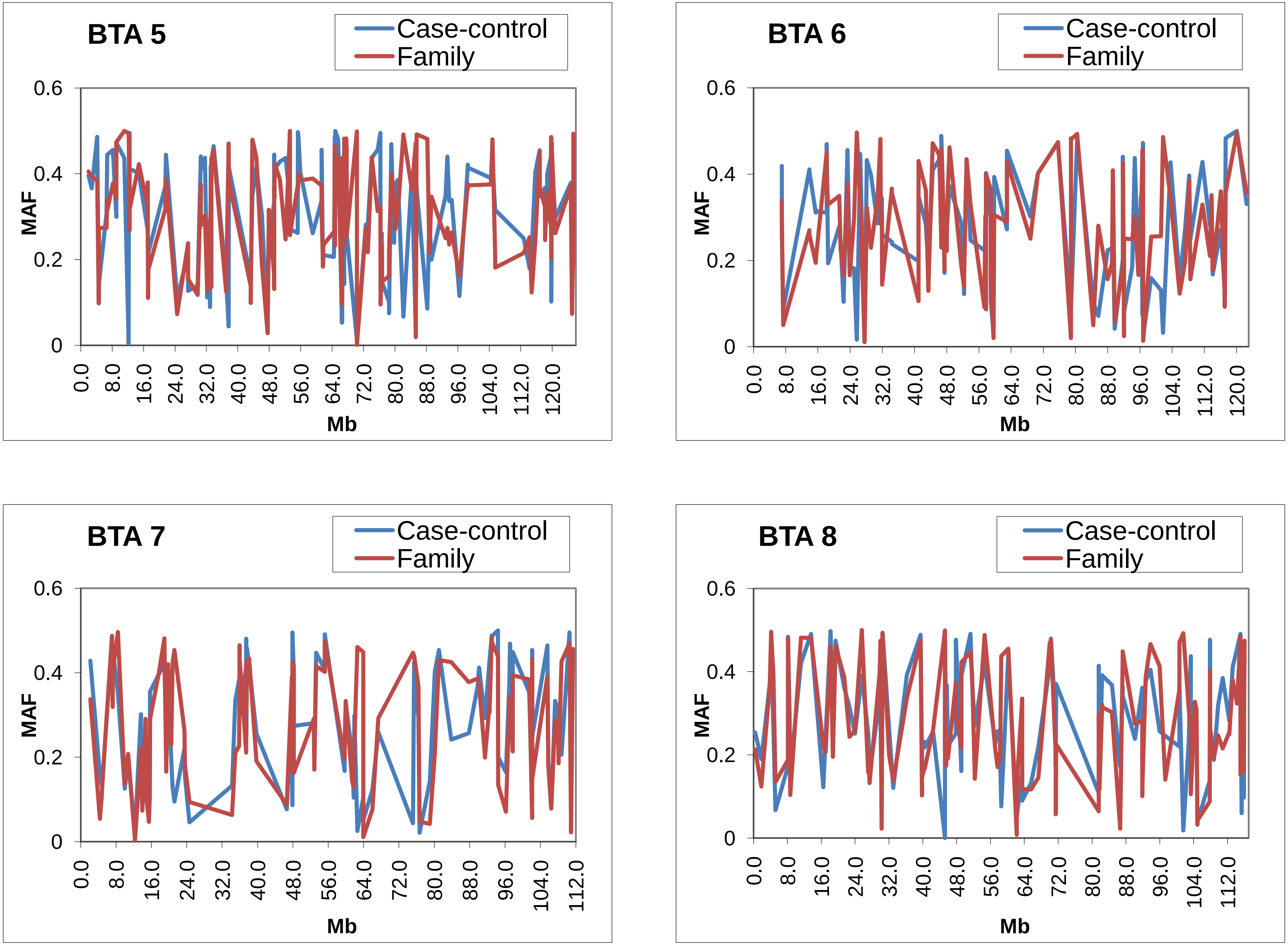
BTA 5
Case-control
Family
MAF
Mb
0.6
0.4
0.2
0
0.0 8.0 16.0 24.0 32.0 40.0 48.0 56.0 64.0 72.0 80.0 88.0 96.0 104.0 112.0 120.0
BTA 6
Case-control
Family
MAF
Mb
0.6
0.4
0.2
0
0.0 8.0 16.0 24.0 32.0 40.0 48.0 56.0 64.0 72.0 80.0 88.0 96.0 104.0 112.0 120.0
BTA 7
Case-control
Family
MAF
Mb
0.6
0.4
0.2
0
0.0 8.0 16.0 24.0 32.0 40.0 48.0 56.0 64.0 72.0 80.0 88.0 96.0 104.0 112.0
BTA 8
Case-control
Family
MAF
Mb
0.6
0.4
0.2
0
0.0 8.0 16.0 24.0 32.0 40.0 48.0 56.0 64.0 72.0 80.0 88.0 96.0 104.0 112.0

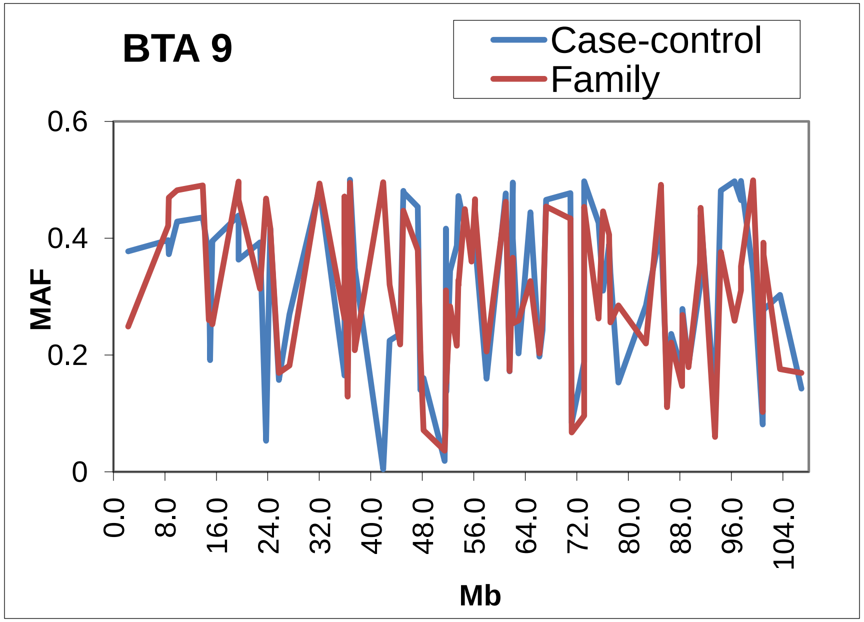
BTA 9
Case-control
Family
0.6
0.4
0.2
0
MAF
0.0
8.0
16.0
24.0
32.0
40.0
48.0
56.0
64.0
72.0
80.0
88.0
96.0
104.0
Mb

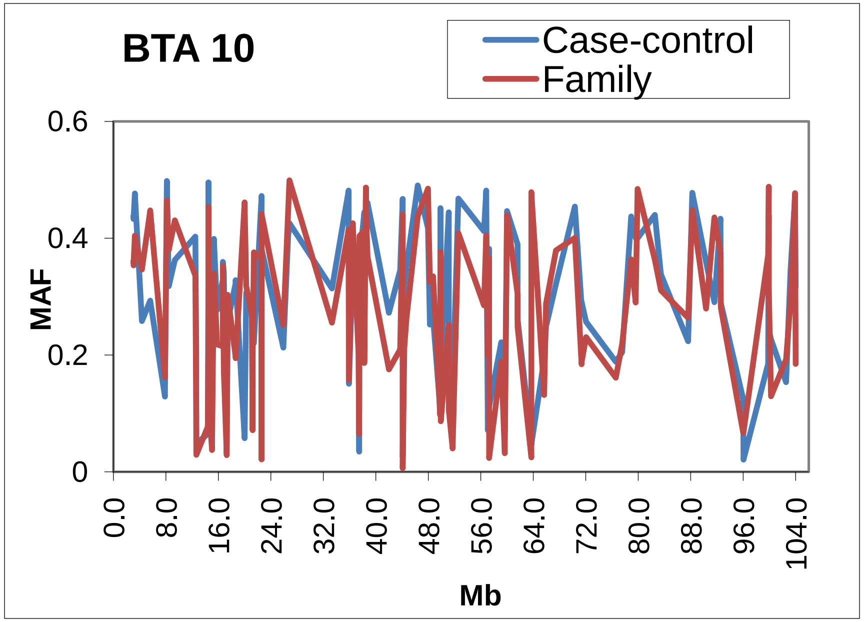
BTA 10
Case-control
Family
0.6
0.4
0.2
0
MAF
0.0
8.0
16.0
24.0
32.0
40.0
48.0
56.0
64.0
72.0
80.0
88.0
96.0
104.0
Mb

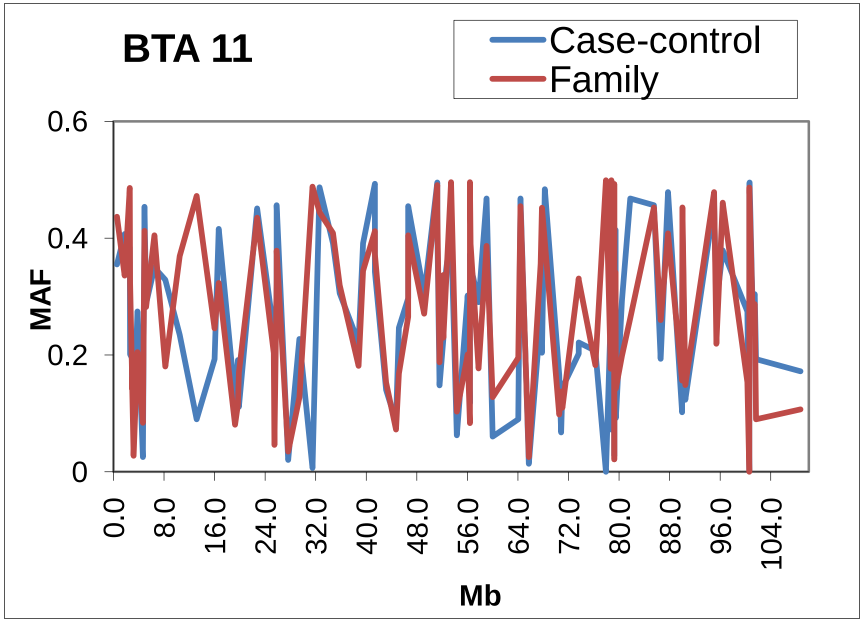
BTA 11
Case-control
Family
0.6
0.4
0.2
0
MAF
0.0
8.0
16.0
24.0
32.0
40.0
48.0
56.0
64.0
72.0
80.0
88.0
96.0
104.0
Mb

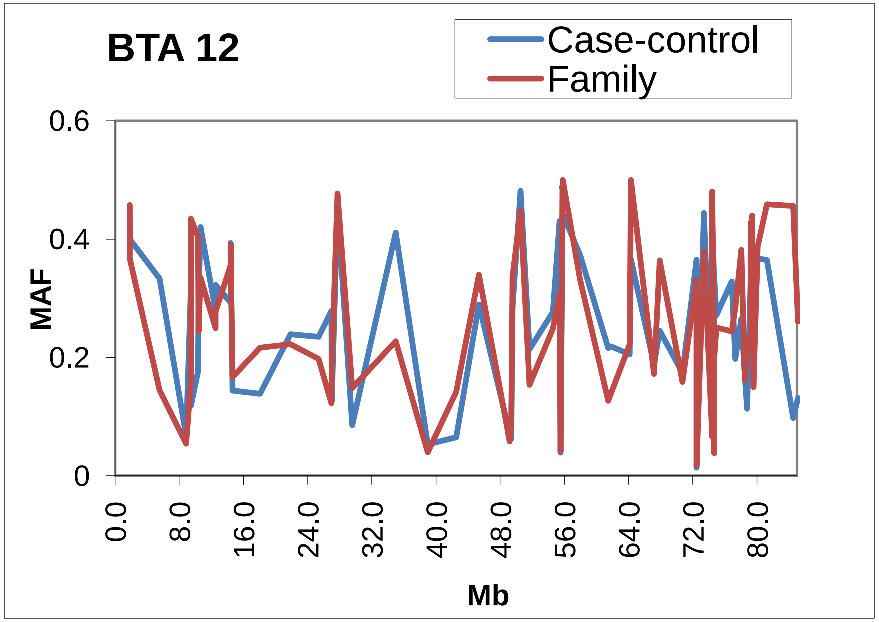
BTA 12
Case-control
Family
0.6
0.4
0.2
0
MAF
0.0
8.0
16.0
24.0
32.0
40.0
48.0
56.0
64.0
72.0
80.0
Mb

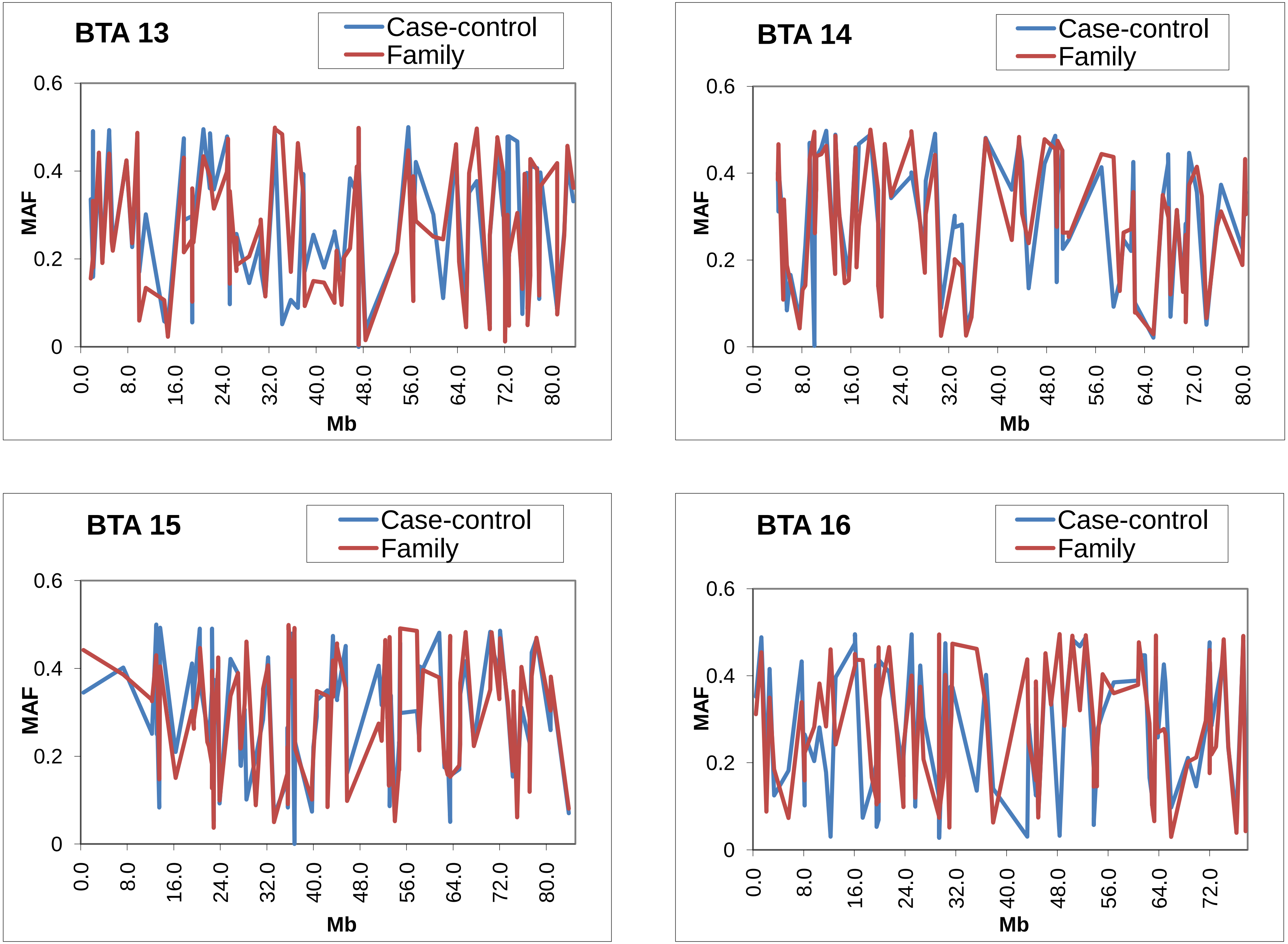
BTA 13
Case-control
Family
MAF
0.6
0.4
0.2
0
0.0 8.0 16.0 24.0 32.0 40.0 48.0 56.0 64.0 72.0 80.0
Mb
BTA 14
Case-control
Family
MAF
0.6
0.4
0.2
0
0.0 8.0 16.0 24.0 32.0 40.0 48.0 56.0 64.0 72.0 80.0
Mb
BTA 15
Case-control
Family
MAF
0.6
0.4
0.2
0
0.0 8.0 16.0 24.0 32.0 40.0 48.0 56.0 64.0 72.0 80.0
Mb
BTA 16
Case-control
Family
MAF
0.6
0.4
0.2
0
0.0 8.0 16.0 24.0 32.0 40.0 48.0 56.0 64.0 72.0
Mb

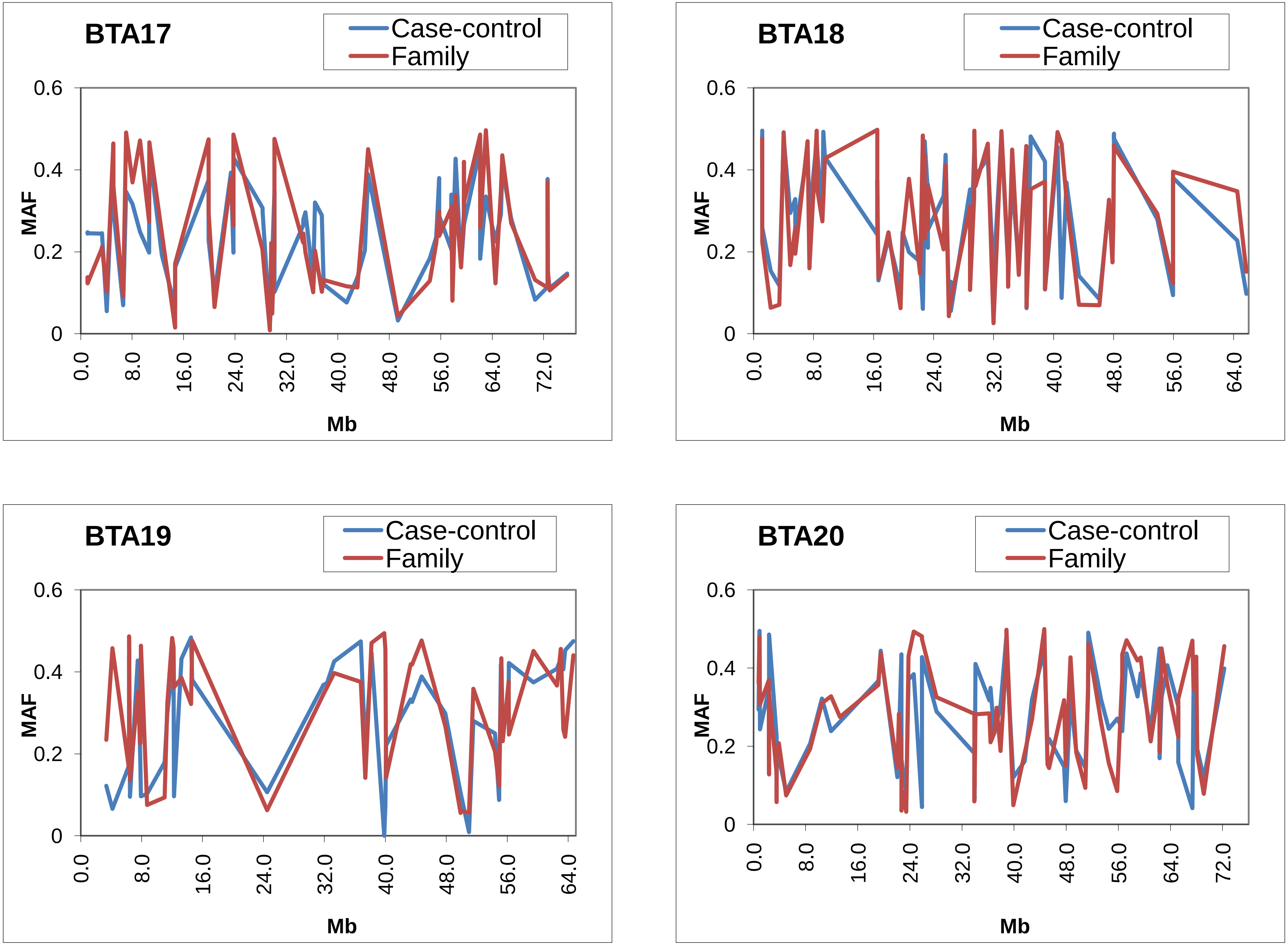
BTA17
Case-control
Family
MAF
Mb
0.6
0.4
0.2
0
0.0
8.0
16.0
24.0
32.0
40.0
48.0
56.0
64.0
72.0
BTA18
Case-control
Family
MAF
Mb
0.6
0.4
0.2
0
0.0
8.0
16.0
24.0
32.0
40.0
48.0
56.0
64.0
BTA19
Case-control
Family
MAF
Mb
0.6
0.4
0.2
0
0.0
8.0
16.0
24.0
32.0
40.0
48.0
56.0
64.0
BTA20
Case-control
Family
MAF
Mb
0.6
0.4
0.2
0
0.0
8.0
16.0
24.0
32.0
40.0
48.0
56.0
64.0
72.0

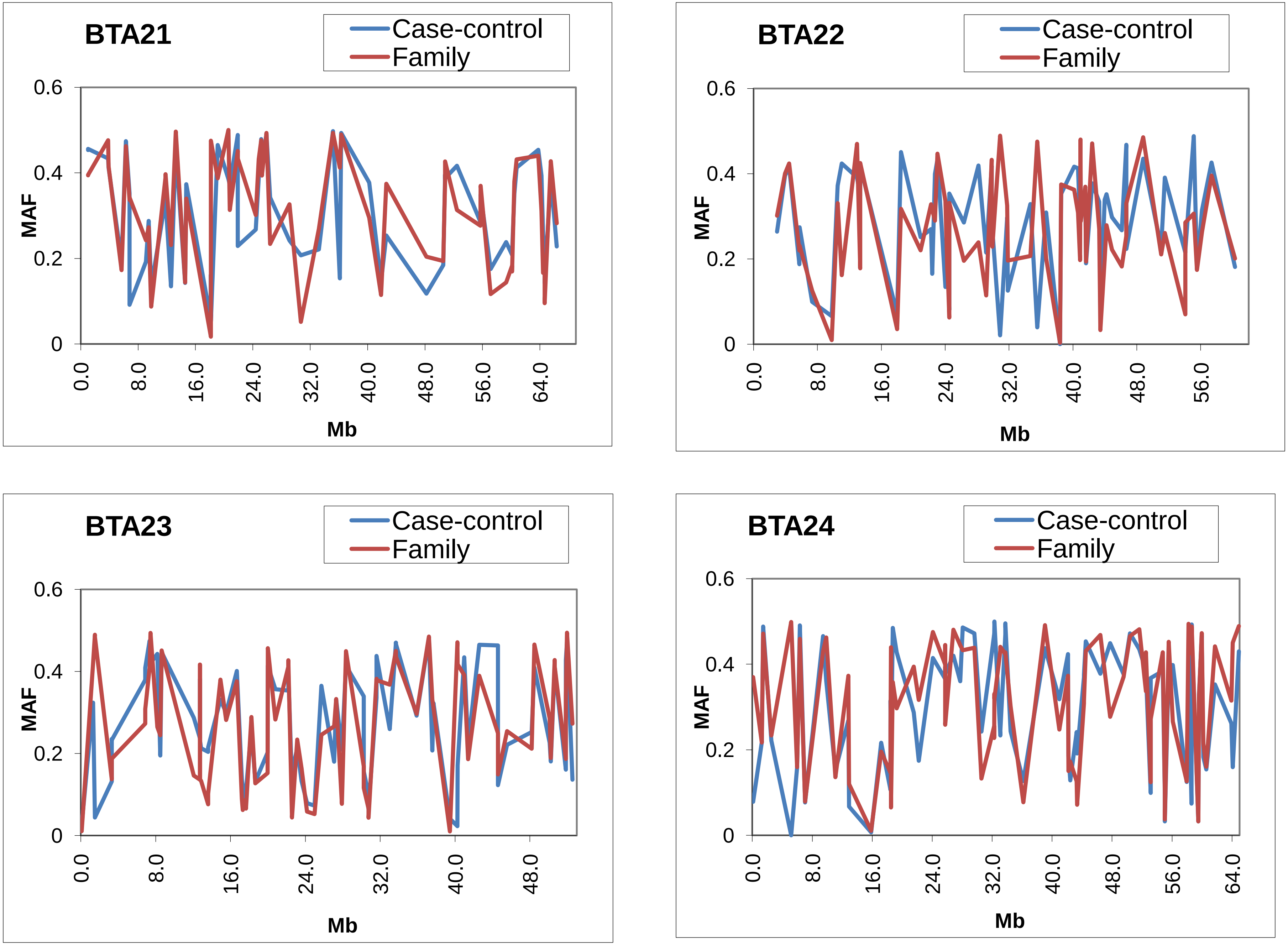
BTA21
Case-control
Family
MAF
Mb
0 0.2 0.4 0.6
0.0 8.0 16.0 24.0 32.0 40.0 48.0 56.0 64.0
BTA22
Case-control
Family
MAF
Mb
0 0.2 0.4 0.6
0.0 8.0 16.0 24.0 32.0 40.0 48.0 56.0
BTA23
Case-control
Family
MAF
Mb
0 0.2 0.4 0.6
0.0 8.0 16.0 24.0 32.0 40.0 48.0
BTA24
Case-control
Family
MAF
Mb
0 0.2 0.4 0.6
0.0 8.0 16.0 24.0 32.0 40.0 48.0 56.0 64.0

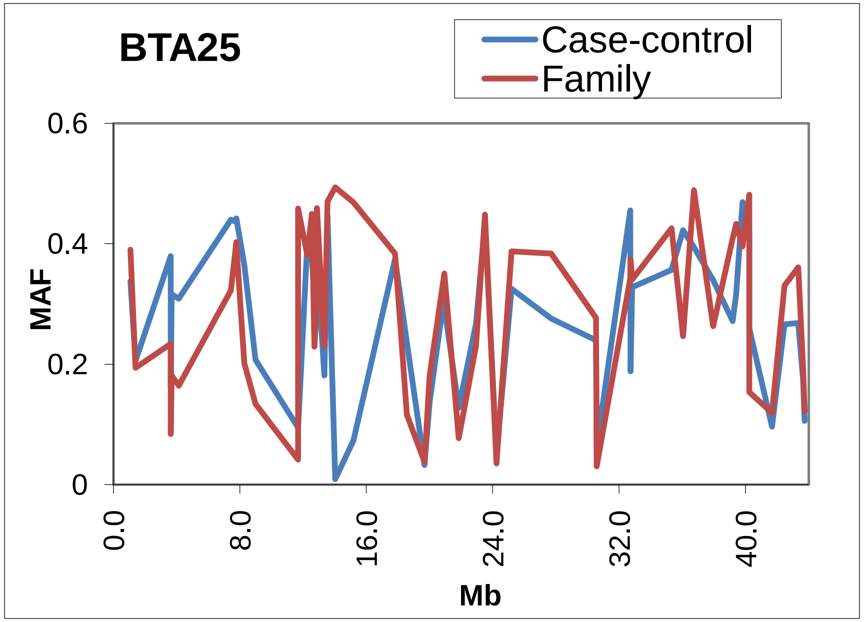
BTA25
Case-control
Family
MAF
0.6
0.4
0.2
0
0.0
8.0
16.0
24.0
32.0
40.0
Mb

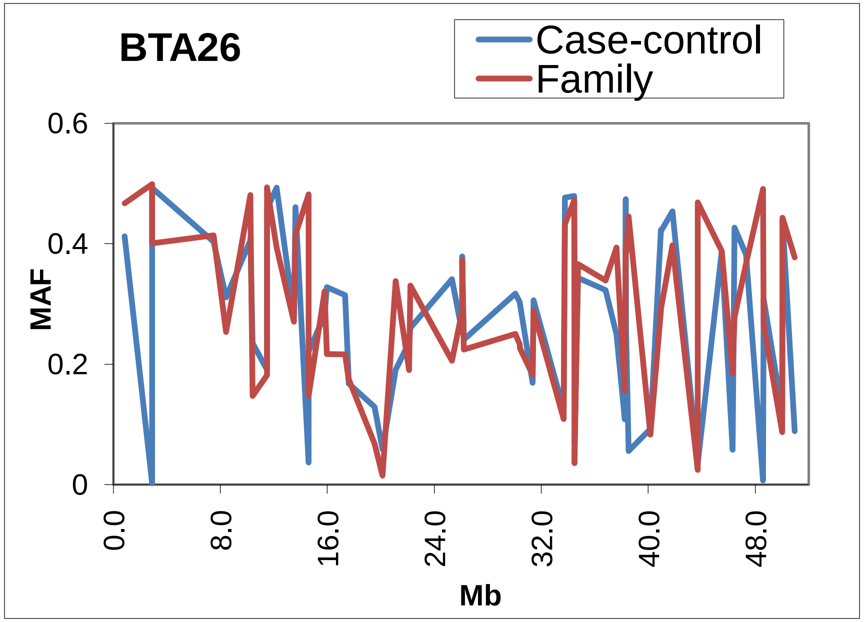
BTA26
Case-control
Family
MAF
0.6
0.4
0.2
0
0.0
8.0
16.0
24.0
32.0
40.0
48.0
Mb

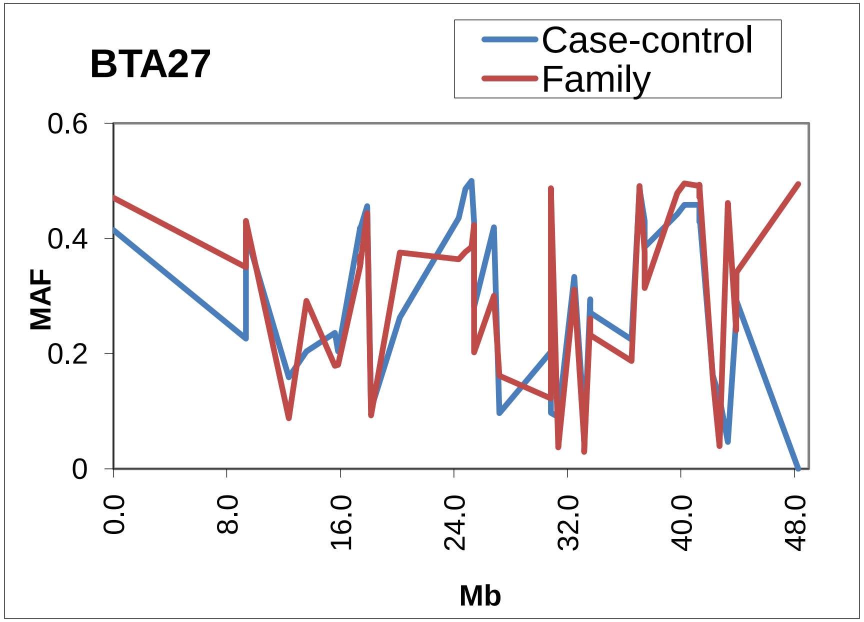
BTA27
Case-control
Family
MAF
0.6
0.4
0.2
0
0.0
8.0
16.0
24.0
32.0
40.0
48.0
Mb

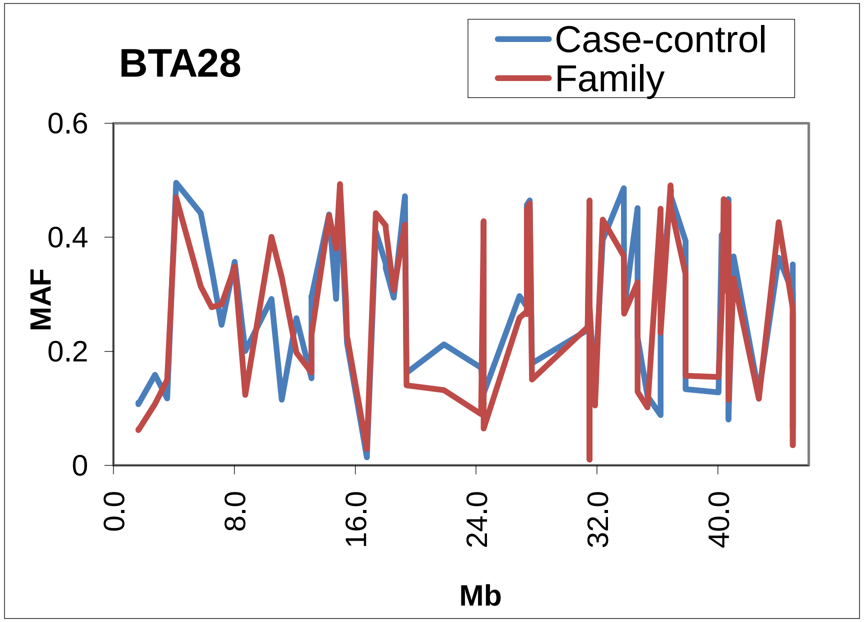
BTA28
Case-control
Family
MAF
0.6
0.4
0.2
0
0.0
8.0
16.0
24.0
32.0
40.0
Mb

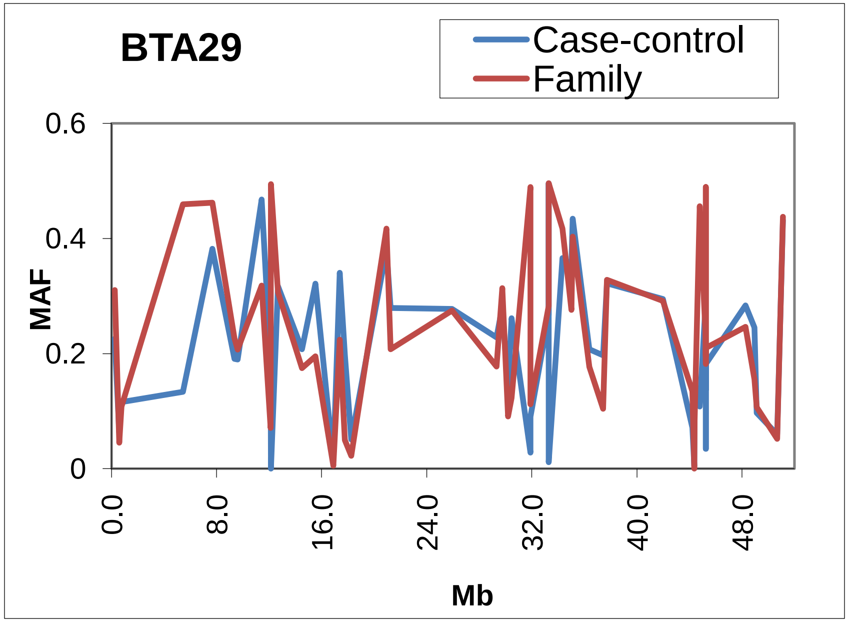
BTA29
Case-control
Family
0.6
0.4
0.2
0
MAF
0.0
8.0
16.0
24.0
32.0
40.0
48.0
Mb

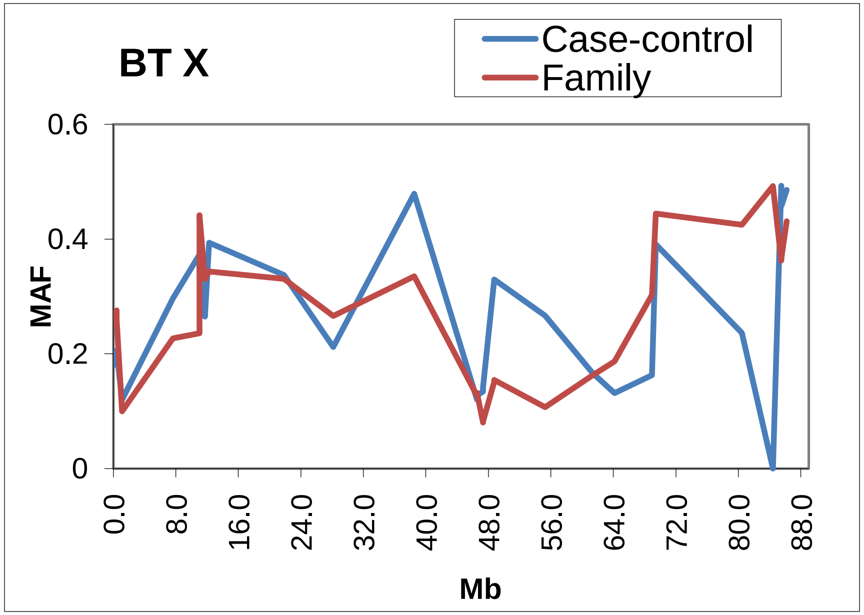
BT X
Case-control
Family
0.6
0.4
0.2
0
MAF
0.0
8.0
16.0
24.0
32.0
40.0
48.0
56.0
64.0
72.0
80.0
88.0
Mb
